# New Developments of the Principle of Vinylogy as Applied to π-Extended Enolate-Type Donor Systems

**DOI:** 10.1021/acs.chemrev.9b00481

**Published:** 2020-02-10

**Authors:** Claudio Curti, Lucia Battistini, Andrea Sartori, Franca Zanardi

**Affiliations:** Dipartimento di Scienze degli Alimenti e del Farmaco, Università di Parma, Parco Area delle Scienze 27A, 43124 Parma, Italy

## Abstract

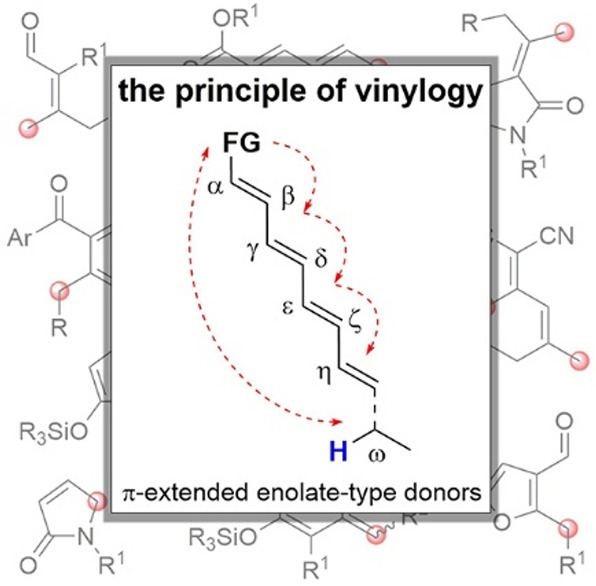

The principle of vinylogy states that the electronic effects of a functional group in a molecule are possibly transmitted to a distal position through interposed conjugated multiple bonds. As an emblematic case, the nucleophilic character of a π-extended enolate-type chain system may be relayed from the legitimate α-site to the vinylogous γ, ε, ..., ω remote carbon sites along the chain, provided that suitable HOMO-raising strategies are adopted to transform the unsaturated pronucleophilic precursors into the reactive polyenolate species. On the other hand, when “unnatural” carbonyl *ipso*-sites are activated as nucleophiles (umpolung), vinylogation extends the nucleophilic character to “unnatural” β, δ, ... remote sites. Merging the principle of vinylogy with activation modalities and concepts such as iminium ion/enamine organocatalysis, NHC-organocatalysis, cooperative organo/metal catalysis, bifunctional organocatalysis, dicyanoalkylidene activation, and organocascade reactions represents an impressive step forward for all vinylogous transformations. This review article celebrates this evolutionary progress, by collecting, comparing, and critically describing the achievements made over the nine year period 2010–2018, in the generation of vinylogous enolate-type donor substrates and their use in chemical synthesis.

## 1 Introduction

**Scheme 1:**
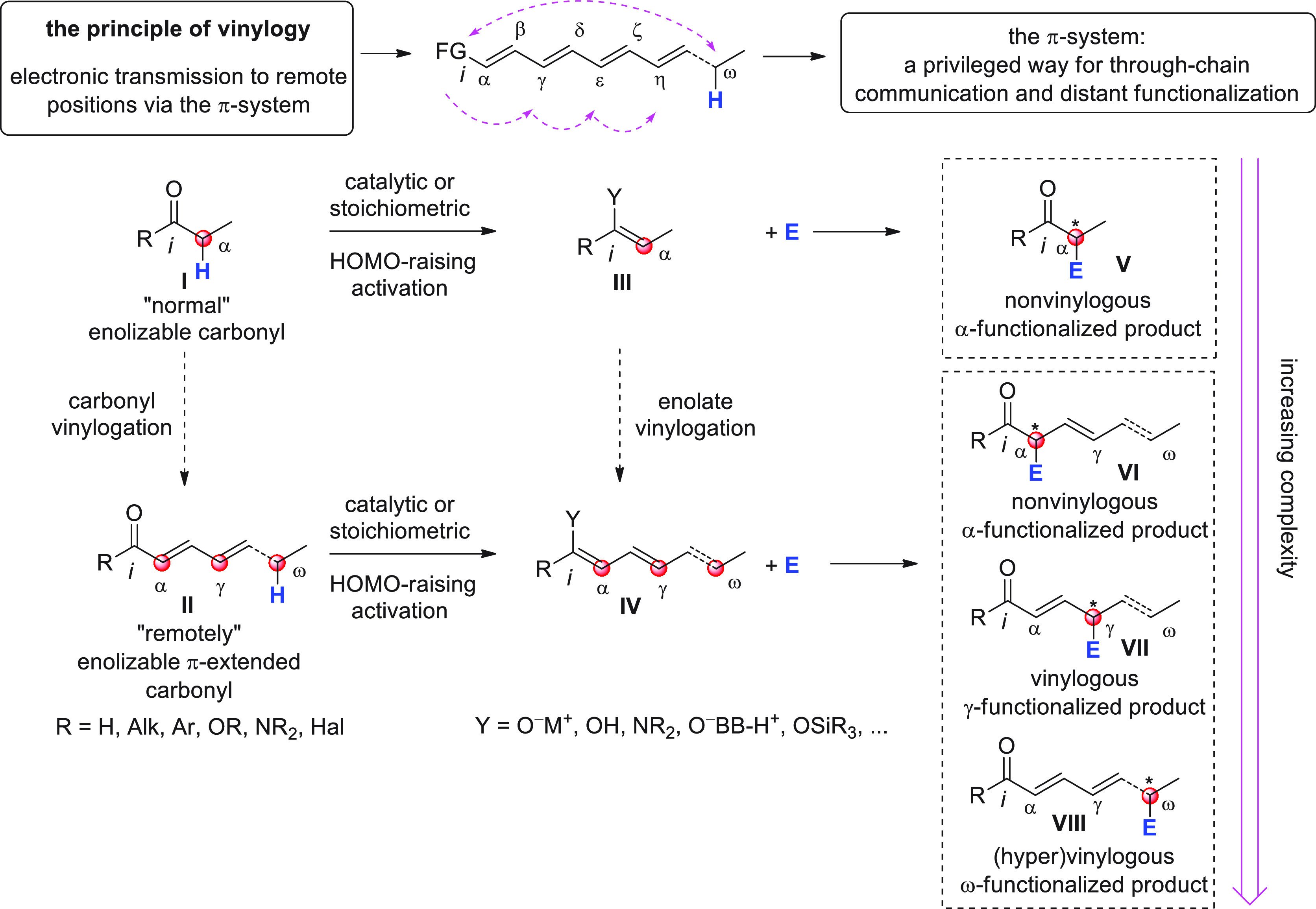
Depiction of the Principle of Vinylogy Applied to π-Extended Carbonyl Compounds **I**/**II** [FG = functional group; E = electrophile; catalytic or stoichiometric HOMO-raising activation refer to covalent or noncovalent activation strategies inducing the formation of metal enolates (Y = O^–^M^+^), enols (Y = OH), enamines (Y = NR_2_), enolates with protonated Brønsted base counterions (Y = O^–^BB^–^H^+^), or silyl enol ethers (Y = OSiR_3_). Red circles indicate pronucleophilic (compounds I/II) and nucleophilic (compounds **III**/**IV**) carbon sites.

The design and development of selective C–H activation reactions at carbon sites remotely positioned from a leading functional group has evolved into an exciting research topic of contemporary synthetic chemistry.^1,2^ The principle of vinylogy, originally formulated by Fuson in 1935,^3^ states that the electronic effects of a functional group in a molecule can be transmitted, via interposed conjugated multiple bonds, to a distal position in the molecule (Scheme 1).

Enolizable, π-extended carbonyl systems of general formula **II** (Scheme 1) (generally including aldehydes, ketones, and carboxyl-level functionalities such as esters/lactones, amides/lactams, acyl halides) can be considered to be emblematic examples of this principle, whereby the electronic properties of the carbonyl functional group are relayed along the carbon chain to remote carbon positions through the conjugated π-system, which effectively represents a privileged means of communication between distant sites. For example, the conventional electrophilic character of the C=O group (*ipso* position, *i*) is “usurped” by the conjugated β, δ, etc. carbon sites and thus a typical 1,2-nucleophilic addition to carbonyl compounds becomes a 1,4-, 1,6-, etc. conjugate addition reaction (not shown). On the other hand, the prototypical pronucleophilic character at the α-position of “normal” enolizable carbonyl compounds **I** is propagated long-range to the vinylogous (and hypervinylogous) γ, ε, ..., ω carbon sites, via in situ-formed or preformed polyenolate-type intermediates **IV**—the vinylogous versions of enolates **III**—using suitable catalytic or stoichiometric HOMO-raising activation procedures (here and throughout this review, hypervinylogous sites refer to those carbon atoms along the π-chain which are separated from the leading functional group by more than one unsaturated linkage). These inherently polydentate donor systems may be engaged in useful enolate-based chemistry with suitable electrophilic partners (e.g., C=O, C=N, activated C=C bonds and other electrophiles) ultimately providing, at least in principle, a plethora of diverse synthetic pathways and products of increasing structural complexity vis-à-vis their simple nonvinylogous counterparts.

While these concepts are generally a well established part of a chemist’s repertoire, far from obvious is how to simultaneously maintain chemo-, regio-, and stereocontrol of the multisite reactivity present in these vinylogous substrates. For example, which of the several possible competing regioisomeric products **VI–VIII** (α vs γ, ... vs ω site selectivity) emerges as being the preferred is a multifactorial issue depending on (1) the intrinsic electronic bias of the nucleophilic carbon sites (HOMO coefficient, in turn dependent on the type of metal/counterion such as Li^+^, SiR_3_, NR_4_^+^, ...), (2) the electrophilic susceptibility of the coupling partner (LUMO coefficient), (3) the presence of strategically placed biasing/bulky substituents along the chain (steric effects), (4) the thermodynamic stability of the products (when the reaction is thermodynamically controlled), and (5) the type and mechanism of the employed catalyst (if any).

Other parameters may add to the complexity of this matter: the use of ketones or branched molecular substrates with a conspicuous number of enolizable positions, the coupling of a vinylogous C–C/C–X bond-forming event to cascade processes of cyclization, and stereochemical issues, concerning the *E/Z* geometry of the emerging olefins within the products, as well as the simple and facial stereocontrol of the newly forged stereocenters.

Given this state of affairs, the advantage of vinylogous transformations over “normal” reactions, namely, increased product complexity with simultaneous formation of multiple functional groups and stereogenic elements, can be brought effectively to fruition, provided that two fundamental conditions are met. First, *suitable activation strategies* and/or catalytic modalities are selected to chemoselectively activate either or both partner substrates, while inducing maximal regio- and stereocontrol, and second, the *vinylogous substrates must remain coplanar* in their reactive conformations, in order to preserve the electronic transmission through the conjugated π-system.

One of the greater successes in the development of vinylogous enolate-based chemistry over the past decades has been the use of preformed silyl enol ethers (or silyl ketene acetal polyenolates) from the corresponding π-extended carbonyl precursors, whose innate electronic predisposition to react at remotely positioned carbon sites has been certified and widely exploited in synthesis.^4^ In the new millennium, the remarkable development of novel covalent and noncovalent, HOMO raising and LUMO lowering activation strategies using chiral organo- and metal-based catalysis has shaped the concept of and the way of conducting both old and new chemical reactions. *Merging the principle of vinylogy with activation modalities and concepts* such as iminium ion/enamine organocatalysis, NHC-organocatalysis, cooperative organo/metal catalysis, bifunctional organocatalysis, dicyanoalkylidene activation, and organocascade reactions truly represents an impressive step forward for vinylogous transformations.

A critical survey of the contributions published in the literature over the past recent years and our own experience in this dynamic field of research made us realize that (1) the palette of pronucleophilic species used as direct or indirect sources of vinylogous donor species has been greatly enriched and diversified since the pre-2010 era; (2) these electron-rich species trigger a spectrum of reactions including the “traditional” aldol/Mannich/Michael addition reactions, but also “new” connections such as [4 + 2], [3 + 2], [n + m] annulations, nitro-Henry additions, Rauhut–Curier reactions, amination and alkylation reactions, and others yet; (3) the activation of such pronucleophiles often includes direct catalytic modalities (e.g., HOMO-raising organocatalytic enamine, NHC activation), while indirect activation of these matrices (e.g., via silyl enol ether preformation) is losing ground although still used in target-oriented synthesis; (4) when strategies are involved that activate the “unnatural” carbonyl *ipso*-site as a nucleophile (umpolung), vinylogation transmits the nucleophilic characteristic to “unnatural” β, δ, ... remote sites.

*Our intention here is to celebrate these evolutionary improvements of the past few years, by collecting, comparing, and critically describing the achievements made, over the nine year period 2010–2018, in the generation of vinylogous enolate-type donor substrates and their use in chemical synthesis*.

## 2 About this Review

In 2000, a review article was published in this journal, dealing with vinylogous aldol addition reactions and chronicling their development and application in organic synthesis from the onset to the end of 1999.^5^ About ten years later, a sequel to this article was published, covering the topic of vinylogous aldol domain and related vinylogous Mannich and Michael reactions emerging from research carried out in the first decade of the new millennium (January 2000-April 2010).^6^

Given the interest shown in this topic by the chemical community^7^ and the continual, ever-growing number of papers published in this field, we have compiled a comprehensive and critical review article about the exploitation of vinylogous enolate-type donor substrates in chemical synthesis over the most recent period January 2010-December 2018.

In order to emphasize the structure and vinylogous reactivity of the pronucleophilic species focus of this article, this review article is subdivided into main sections, according to the *functional group responsible for vinylogous reactivity*, namely, vinylogous aldehydes, ketones, esters/lactones, amides/lactams, nitriles, and others (generally unsaturated and saturated acyl halides and carboxylic acids, nitro (hetero)aromatic compounds, vinylphenols). In this way, the reader can readily visualize the main classes of provinylogous substrates (which will also be presented collectively by appropriate figures at the beginning of each main section) and observe how they act in different ways to generate the active nucleophilic species and fare in subsequent homologation reactions.

Each main section is organized into subsections, according to the *nature of the electrophilic substrate involved in the vinylogous coupling*, namely, additions to C=O (aldol-type additions and related cascade cyclization reactions), C=N (Mannich-type additions, 1,3-dipolar cycloadditions and related cascade cyclization reactions), electron-poor C=C bonds (Michael-type additions and related cascade cyclization reactions), and miscellaneous electrophiles (alkyl halides, amination reagents, and others). A further distinction between *direct procedures* (in situ activation of pronucleophiles by suitable organo- and/or metal-catalysts) and *indirect procedures* (use of preformed and isolated silyl-derived nucleophiles) has been made. For each subchapter, the contributions are grouped according to the *acyclic vs cyclic nature of vinylogous donors*, where the term “cyclic donor” denotes those substrates where the conjugated π-system, responsible for the vinylogous transmission, is partially or totally included in one, or more, carbo- or heterocyclic ring. Far from being a fictitious subdivision, this classification reflects the profound differences between the two classes, attributed mainly to different steric and electronic properties, especially when aromatic compounds are involved. In the absence of pertinent studies, the relevant subsection is not treated.

As already stated, given that the principle of vinylogy, *per se*, includes practically all existing functional groups which are “vinylogated”, the field has been restricted here, by choosing vinylogous pronucleophiles containing carbonyl, as well as carboxyl-level and miscellaneous, functional groups which may act, upon suitable activation, as electron-rich donor components in polar, enolate-type reactions to forge new remote C–C and C–X (X = N, O, S, halogen, H) connections. Special attention is paid to enantioselective reactions yielding chiral nonracemic products.

For these reasons, the review will not cover the following topics: (1) vinylogous electrophiles (e.g., Michael acceptors and higher homologues, which are the subject of focused reviews);^8−10^ (2) condensation reactions where the newly created stereocenters are promptly lost;^11^ (3) examples where the vinylogous multidentate donors react at nonvinylogous positions (α-attack);^12,13^ (4) reactions involving reactive π-extended radical species; and (5) simple Friedel–Crafts reactions where the π-extended conjugate system remains within an aromatic ring and no leading “carbonyl-type” functionalities are present.^14^

On a technical note, conceptually similar articles are reviewed sequentially or under tabular format, and are often preceded by general, explanatory schemes. Throughout the work, reactive nucleophilic or pronuleophilic vinylogous sites in the structural formulas are denoted by red circles, electrophilic sites by blue circles, and the newly formed linkages are colored in red. For the sake of the reader, dashed lines connecting the electronically complementary reactive sites within substrates are used when complex annulation reactions are described. Particular emphasis is placed on the mechanistic investigations made by the authors, especially when vinylogous enolate-type donors are employed, as diene components, in cycloaddition reactions; most of the alleged cycloadditions under review either were not mechanistically studied thoroughly or they revealed their actual stepwise, vinylogous nature.

During the 2010–2018 period considered here, a considerable number of authoritative reviews, accounts, and highlights appeared in the literature that focused on particular aspects of vinylogy.^7^ Many of them accounted for specific HOMO-raising catalytic activation modalities of pronucleophiles (amino-organocatalysis,^15−18^ NHC-organocatalysis,^19,20^ noncovalent organocatalysis);^21−23^ some dealt with selected classes of vinylogous functional groups (aldehydes,^24,25^ nitriles,^26,27^ alkylidene carbo- or heterocycles^28,29^), while others were focused on either specific reaction types (Michael additions,^30,31^ Mukaiyama-type C–C bond forming reactions,^32^ organocascade reactions^33^) or selected target categories (polyketides,^34^ γ-butenolides^35,36^).

An updated, comprehensive and critical review article embracing all these allied subjects under one common underlying principle—the principle of vinylogy—was missing, and our intention here is to fill that void.

## 3 Vinylogous Aldehydes

**Figure 1:**
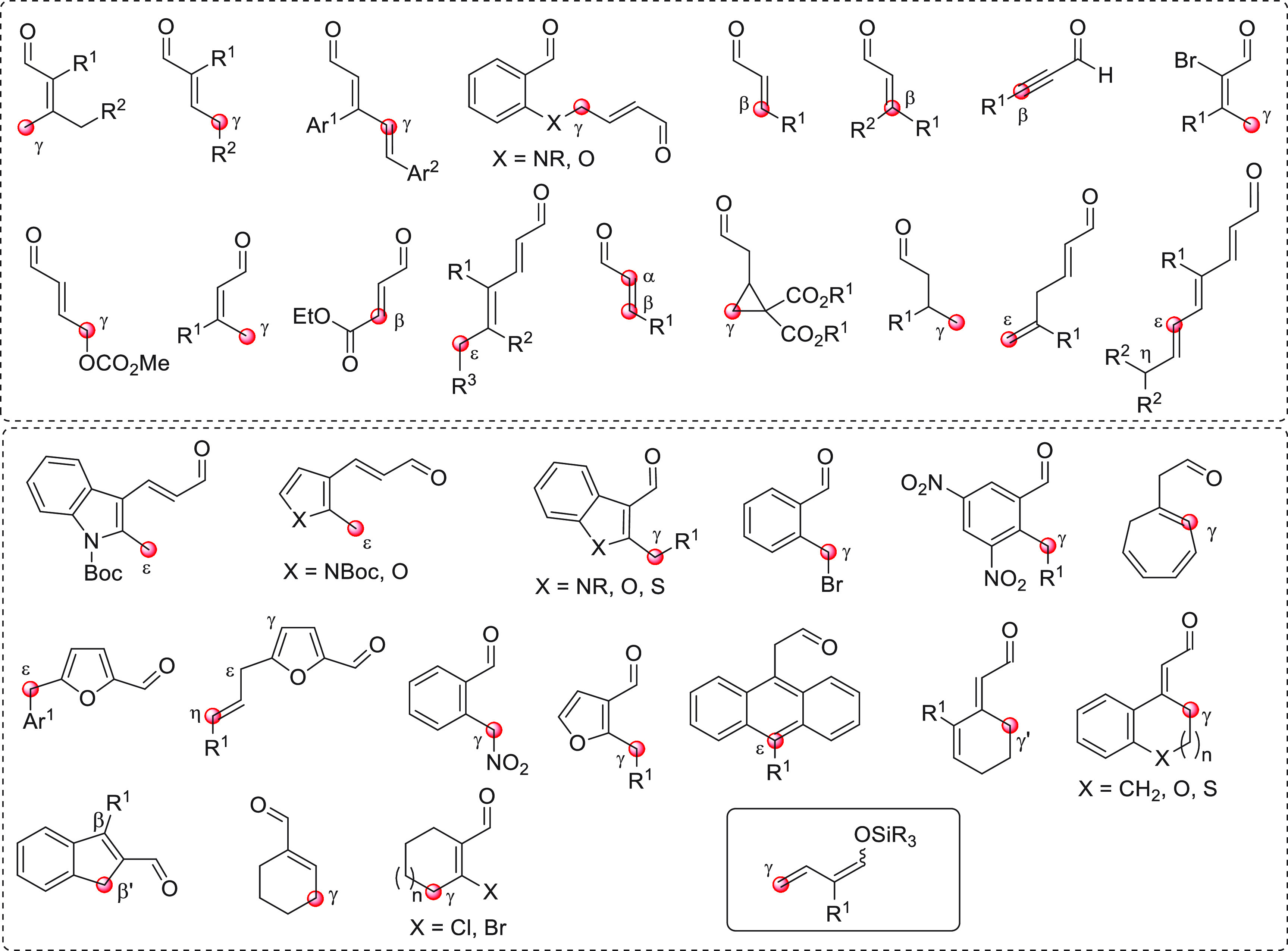
Collection of acyclic (above) and cyclic (below) pronucleophilic aldehydes at work in this section using the direct procedures. In the plain box the only type of nucleophilic aldehyde-derived silyl dienol ether used in indirect procedures. Red circles denote the reactive (pro)nucleophilic carbon site.

The aldehyde function occupies a cardinal position among the most popular polar functional groups that can be “vinylogated”. The possibility of readily obtaining remotely enolizable enals and higher-order homologues via synthesis makes this class of compounds a qualified source of carbon pronucleophiles for use in fruitful additions to electronically complementary partners, especially carbonyl acceptors or electron-poor alkenes. Due to the invention and powerful exploitation of catalytic activation modalities, particularly suited for the aldehyde functional group, the majority of the examples in this section command direct procedures, where conjugated aldehydes are activated in situ to unveil their remote carbon nucleophilicity at either “natural” γ, ε, ... sites, or “unnatural” β sites, according to umpolung polarity reversal (vide infra). On the other hand, indirect procedures, using preformed stable enolates (e.g., silicon extended polyenolates), are limited to a rather restricted number of examples, reflecting the general trend in organic synthesis toward the use of direct, catalytic, and stereoselective methods. Pronucleophilic aldehyde substrates, reported in this chapter under the section “direct procedures”, are depicted in Figure 1, subdivided into acyclic and cyclic representatives, and with their reactive pronucleophilic remote sites indicated. The molecular structure of one aldehyde-derived dienolate nucleophile, used in indirect procedures, is also portrayed in this same figure.

### 3.1 Additions to C=O Bonds

#### 3.1.1 Direct Procedures

##### 3.1.1.1 Acyclic Pronucleophiles

**Scheme 2:**
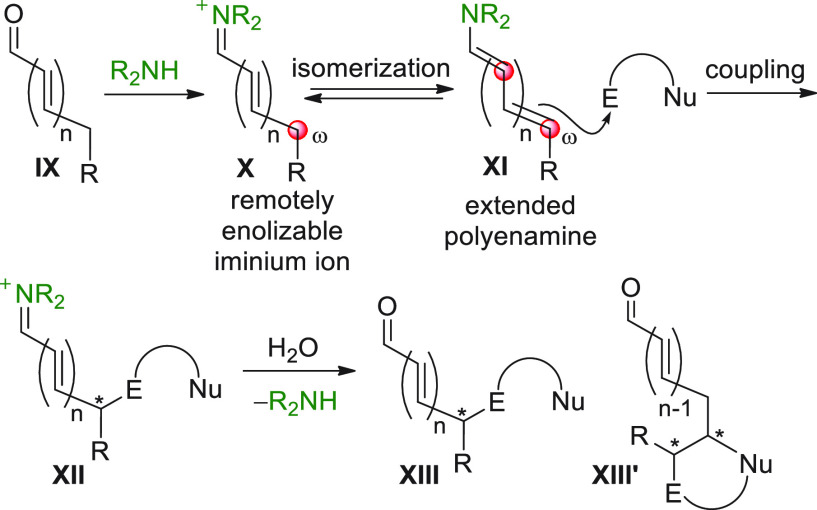


After the launch of chiral secondary amines as useful organocatalysts to be systematically exploited in either the α-pronucleophilic activation of enolizable aldehydes (according to the HOMO-raising principle) or β-electrophilic activation of α,β-unsaturated aldehydes (LUMO-lowering principle) via enamine and iminium ion intermediates, respectively,^37^ it was promptly recognized that such principles could be efficiently translated and exploited in the realm of vinylogy. In particular, the covalent activation of a remotely enolizable conjugated aldehyde **IX** (Scheme 2), through condensation of the carbonyl group with a chiral secondary amine, can give rise to an extended polyenamine species of type **XI** (dienamine, trienamine, tetraenamine, ...) by isomerization of the polyunsaturated iminium ion **X**, thus unveiling multiple and often competing nucleophilic carbon centers to be successfully engaged in, for example, aldol-type addition reactions (Scheme 2, E = carbonyl). In the event, the ω-coupling product **XIII** is formed after the original carbonyl restoration and catalyst recycling; alternatively and more frequently, variously forged cyclized carbonyl products (e.g., compounds **XIII′**) are obtained, when additional electronically complementary functional groups in both reacting partners trigger intramolecular cascade reactions.

**Scheme 3:**
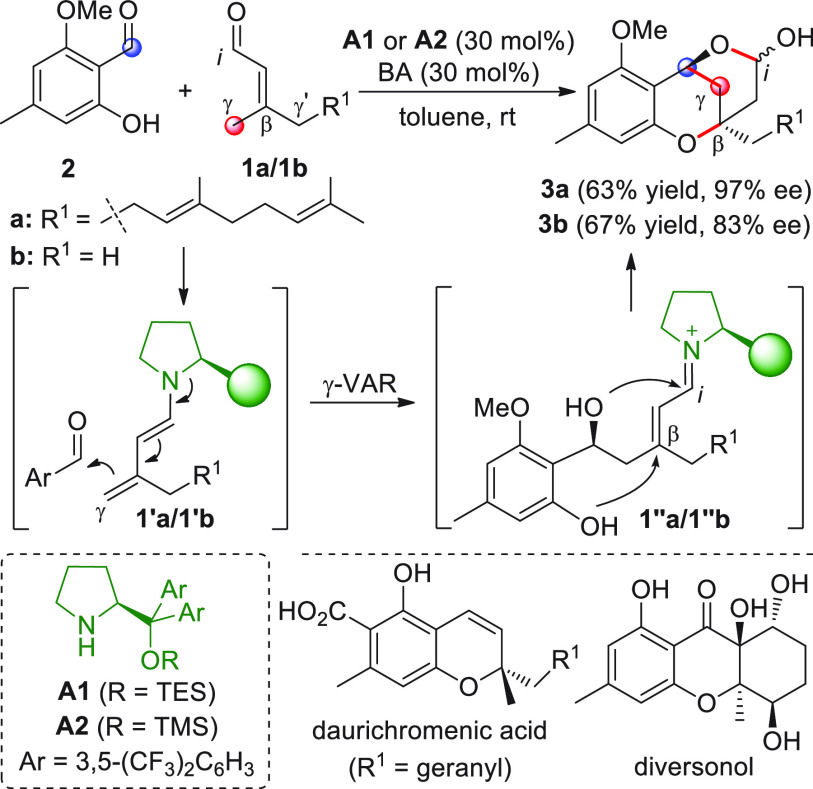


The Woggon and Bräse groups were among the first to recognize that merging the concepts of asymmetric amine-based covalent organocatalysis with vinylogy and cascade reactions could have enormous potential in the synthesis of natural products. By following the chemical strategies they had already adopted prior to 2010,^38,39^ these two groups independently exploited terpenoid-related γ-enolizable α,β-unsaturated aldehydes **1a** or **1b** as useful γ-donor precursors (Scheme 3). When farnesal **1a** (R^1^ = geranyl)^40^ or prenal **1b**(41) (R^1^ = H) was reacted with salicylaldehyde **2** in the presence of diarylprolinol silyl ethers **A1** or **A2** as the key amine organocatalysts (the same reaction conditions were used in both cases, i.e. 30 mol % catalyst and benzoic acid additive, in toluene), the tricyclic chiral lactols **3a** or **3b** were respectively obtained in useful yields and moderate to good enantioselectivities. Both authors hypothesized, but did not experimentally prove, that these products were the result of an organocatalytic domino sequence^33,42−44^ encompassing an initial vinylogous γ-aldol addition reaction (γ-VAR) between the activated dienamine **1′a/1′b** (formed upon condensation of the catalyst and enal **1** with subsequent iminium ion/enamine isomerization) with aldehyde **2**, followed by intramolecular oxa-Michael closure of the phenolic OH on the nascent unsaturated iminium ion and final spontaneous acetalization. Thus, the pronucleophilic aldehyde initiators **1a/1b** provided the γ/β carbon sites in a stepwise [4 + 2] annulation process. Lactol **3a** was subsequently used for the enantioselective entry to anti-HIV active daurichromenic acid and confluentin,^40^ while **3b** and related analogues served to obtain naturally occurring diversonol and other tetrahydroxanthone and chromone lactone families.^45^

To be precise, in both studies, terpenoid enals **1a** and **1b** possess two nonequivalent, remotely enolizable positions, namely, the γ and γ′ sites, which, in principle, could give rise to diverse competing regioisomeric products. Although this issue did not emerge in these studies, as only products **3a** and **3b** were reported, it was rebooted some years later by the Liu group,^46^ who studied the same reaction extensively and investigated how diverse substituents to the aromatic ring of salicylaldehyde precursors of type **2** could influence the γ/γ′ regioselectivity of the process, thus opening a doorway to novel non-natural chroman derivatives embedded with quaternary stereocenters (not shown).

**Scheme 4:**
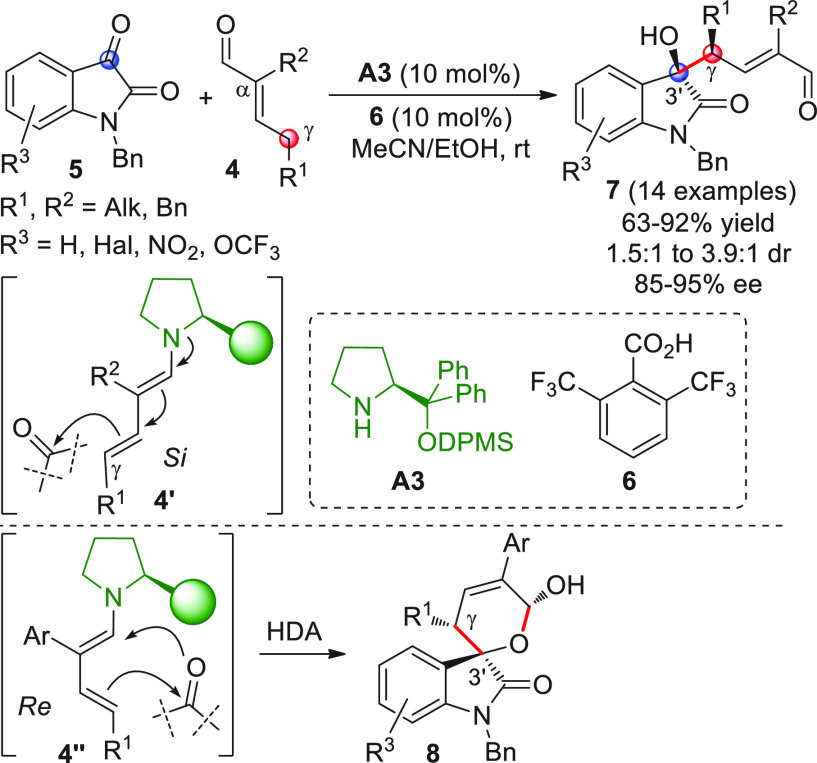


The first study of a direct, enantioselective VAR, involving acyclic enolizable enals yielding isolable and stable aldol products, uninvolved in cascade sequences, was reported by the Melchiorre group in 2012.^47^ It was found that different α,γ-dialkyl substituted enals **4** reacted directly with isatins **5** upon in situ HOMO-raising activation by the bulky chiral secondary amine **A3** catalyst through formation of dienamine **4′** (Scheme 4). Access to the enantioenriched vinylogous aldol-oxindole products **7** was secured (exclusive γ-attack was observed) as variable mixtures of isolable diastereoisomers at C3′ in high yields and moderate to good enantioselectivities.

Of note, it was observed that the chemical behavior of enals **4** strictly depended upon the nature of the α-R^2^ substituent: when R^2^ was aryl instead of alkyl or benzyl, coupling of **4** with **5** under the same reaction conditions gave bicyclic acetal products **8** with reverted stereochemistry at Cγ as a mixture of separable C3′ diastereoisomers. This behavior was probably the result of a pericyclic [4 + 2] cycloaddition (hetero-Diels–Alder, HDA) involving *s-cis* dienamine conformer **4″**, as demonstrated by NMR-based experiments and control reactions (Scheme 4). In this last instance, given the concerted nature of the HDA coupling, the transformation cannot be strictly regarded as a vinylogous procedure, since no electronic transmittal from a given functional group is relayed along a conjugated system to a remote site; however, it could be classified as a vinylogous process if an asynchronous cycloaddition mechanism was invoked; this was not proven nor excluded by the authors.

**Scheme 5:**
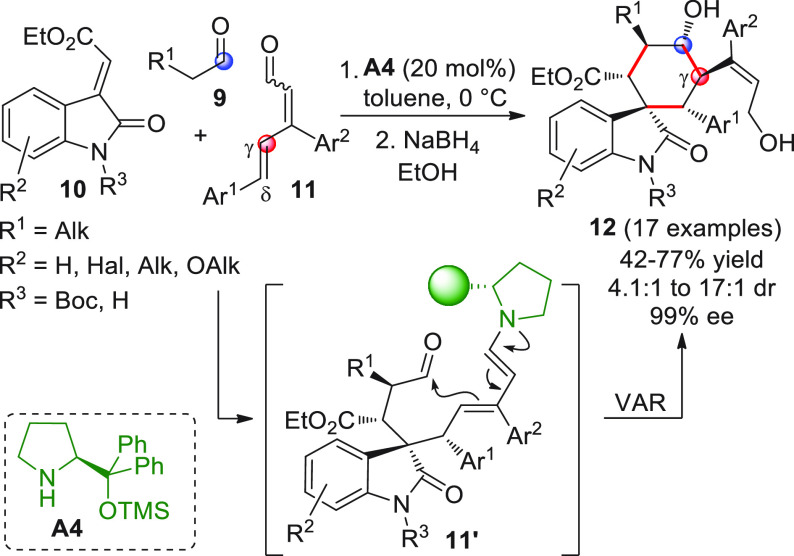


Nonenolizable polyenals of type **11** may well serve as γ-donor components in aldol-type additions, provided that suitable activation modalities are implemented with timely precision (Scheme 5). This did not remain unnoticed by the Melchiorre group when they exploited β,δ-diaryl-substituted dienals **11** in a three-component organocatalyzed cascade coupling reaction with α-enolizable aldehydes **9** and isatin-derived activated alkenes **10**.^48^ Using prolinol silyl ether **A4** in 20 mol % loading secured the prompt formation of highly valuable spirooxindole cyclohexane derivatives **12** bearing six contiguous stereocenters with excellent enantiocontrol (99% ee in all cases). Given the multisite, electronically complementary reactivity of the three reaction partners, different reaction pathways could be, in principle, operative. Indeed, a well orchestrated activation sequence was in motion, encompassing an initial enamine-triggered Michael addition of aldehyde **9** to acceptor **10**, a second intermolecular δ-selective 1,6-conjugate addition of the emerging enolate to iminium ion-activated dienal **11** to forge spirocyclic dienamine **11′**, and a final intramolecular VAR providing the final products (which were isolated as alcohol derivatives **12** after aldehyde reduction with NaBH_4_). Key to the success of the reaction in terms of both regio- and chemoselectivity were the judicious positioning of biasing aryl β-substituents within **11**, in preventing parasitic 1,4-conjugate additions; and one-pot, sequential additions of the starting materials (**9 + 10 + A4** at the beginning, followed by addition of **11**) to preclude the formation of unwanted aldol condensation products.

**Scheme 6:**
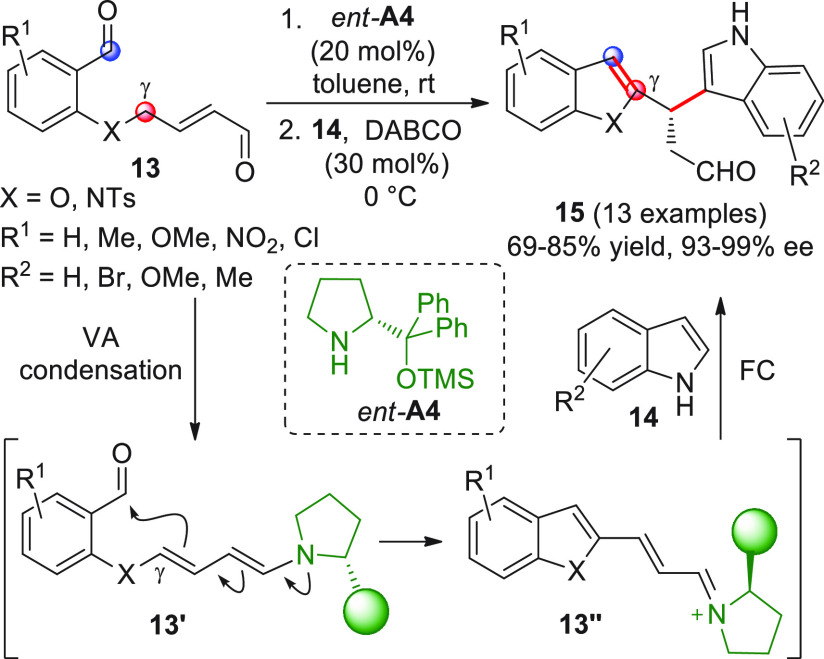


Ad hoc prepared γ-enolizable dialdehydes **13** served as the starting materials for the enantioselective entry to various diheteroaryl alkanals **15**, according to a one-pot sequential reaction involving indoles **14** under silyl prolinol organocatalysis (Scheme 6).^49^ To account for the observed chemical behavior, the authors hypothesized that an initial intramolecular γ-VAR occurs through the intermediacy of dienamine **13′**, formed after condensation of the Hayashi–Jørgensen catalyst *ent*-**A4** with the enal-aldehyde function, followed by subsequent iminium–enamine isomerization. In the event, the aldol condensation product **13″** is obtained, with no new stereocenters formed. An intermolecular Friedel–Crafts reaction then occurs between indole **14** and the formed iminium ion **13″**, under classical “steric shielding control” by the appended catalyst, to afford the targeted heterocycles **15** in good yields and notable enantiomeric excesses. Antiproliferative assays of the new products on cancer cell lines revealed interesting cytotoxic activity, in some instances, comparable or even superior to that of cisplatin.

The HOMO-raising activation of enals, via catalytic dienamine formation, could also be effectively applied to an unsual yet very interesting [5 + 2] formal cycloaddition reaction involving α,β-unsaturated aldehydes and oxidopyrylium ylides generated in situ from 1-acetoxyisochroman-4-ones (not shown).^50^ These dienamine intermediates showed exclusive γ,β-reactivity and provided direct asymmetric entry to oxabicyclooctane-containing products by using a bifunctional secondary amine-squaramide organocatalyst.

**Scheme 7:**
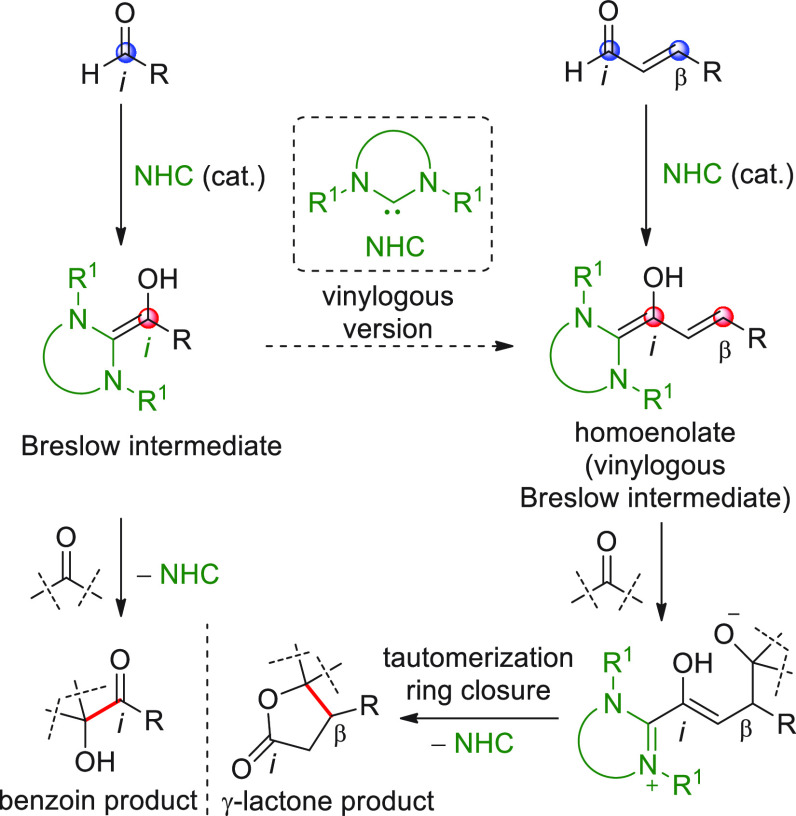


Besides the HOMO-raising activation modality of enals involving amine organocatalysts, another successful activation mode is represented by covalent N-heterocyclic carbene (NHC) organocatalysis. The classic NHC-catalyzed a^1^ → d^1^ umpolung reactions of aldehydes, where benzoin products are formed by the C-*ipso* (*i*) pronucleophilic site of the Breslow intermediate attacking a carbonyl acceptor (Scheme 7), have found their vinylogous counterparts in “homoenolate” chemistry introduced in 2004 by the pioneering and independent works of the Bode and Glorius groups.^51,52^ In this instance, the direct covalent activation of the starting enal, via NHC catalyst, generates the key homoenolate species–a vinylogous Breslow intermediate–whose pronucleophilic β-carbon site (d^3^) attacks the carbonyl acceptor providing γ-lactone products after tautomerization to a catalyst-bound carboxylate and ring closure by the formed alkoxide with concomitant catalyst recycling (Scheme 7). A formal [3 + 2] annulation is thus attained, where the β/*ipso* carbon sites of the starting enal close onto the C=O bond of the carbonyl acceptor. Complete β vs *ipso* regioselectivity was attained within the reactive homoenolate by means of the judicious choice of the NHC catalyst, whose electronic and, above all, steric properties can allow for complete depletion of the C-*ipso* reactivity in favor of the β-position, as well as the premature proton quenching of the catalyst-bound intermediate.

Following these pillar studies, intense research in this field was perfomed by diverse groups during 2004–2009, where assorted electrophiles (i.e., C=O, C=N, and activated C=C bonds, vide infra) were also used, and by the new decade (from 2010 onward), the time was right for extending the horizon of this process toward highly diastereo- and enantioselective variants for the asymmetric construction of a wide range of hetero- and carbocyclic structures. In particular, chiral 1,3,4-triazol-2-ylidene catalysts were used for this scope, often combined with additives.

**Scheme 8:**
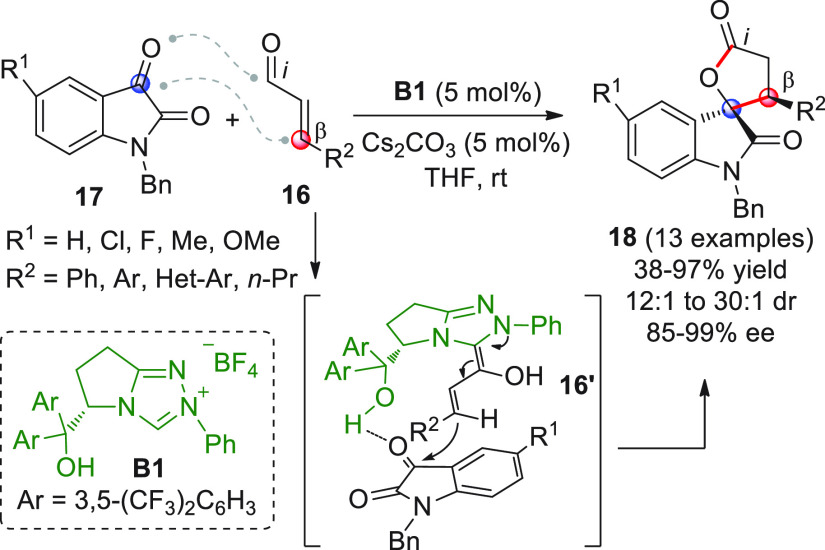


Among the first reports of efficient and enantioselective [3 + 2] annulations involving NHC-homoenolates was that by Ye et al., who employed enals and isatins as starting substrates (Scheme 8).^53^ It was found that L-pyroglutamic acid-derived triazolium salt **B1** (5 mol % loading), bearing a free hydroxyl group appendage, was the NHC precursor best to succeed in the transformation. Hence, a series of aromatic enals of type **16** coupled with isatins **17** to give the corresponding spirocyclic oxindolo-γ-butyrolactones **18** with very good yields and stereoselectivities, except for the single case of β-*n*-propyl-substituted enal **16** (R^2^ = *n*-Pr), which afforded product **18** in a rather poor 38% yield. One limitation of the procedure was that only isatin carbonyls proved to be suitable substrates, since other aldehydes, such as benzaldehyde or propionaldehyde, failed to react under the reported conditions. Given the strong influence of the free hydroxyl group on the activity of the catalyst, the authors proposed a plausible transition state where H-bonding operated between the catalyst and isatin and was probably responsible for enhancing the carbonyl reactivity and directing the homoenolate addition along the indicated trajectory (see **16′**).

Shortly after this study, three other works were independently published by the Scheidt,^54^ Coquerel,^55^ and Glorius^56^ groups, who assayed the prototypic reaction between enals and isatins exploiting the concept of dual organocatalysis: the first group applied an NHC/Lewis acid activation, the second exploited bifunctional NHC/thiourea organocatalysts, and the third group employed NHC/Brønsted acid catalysis.

**Scheme 9:**
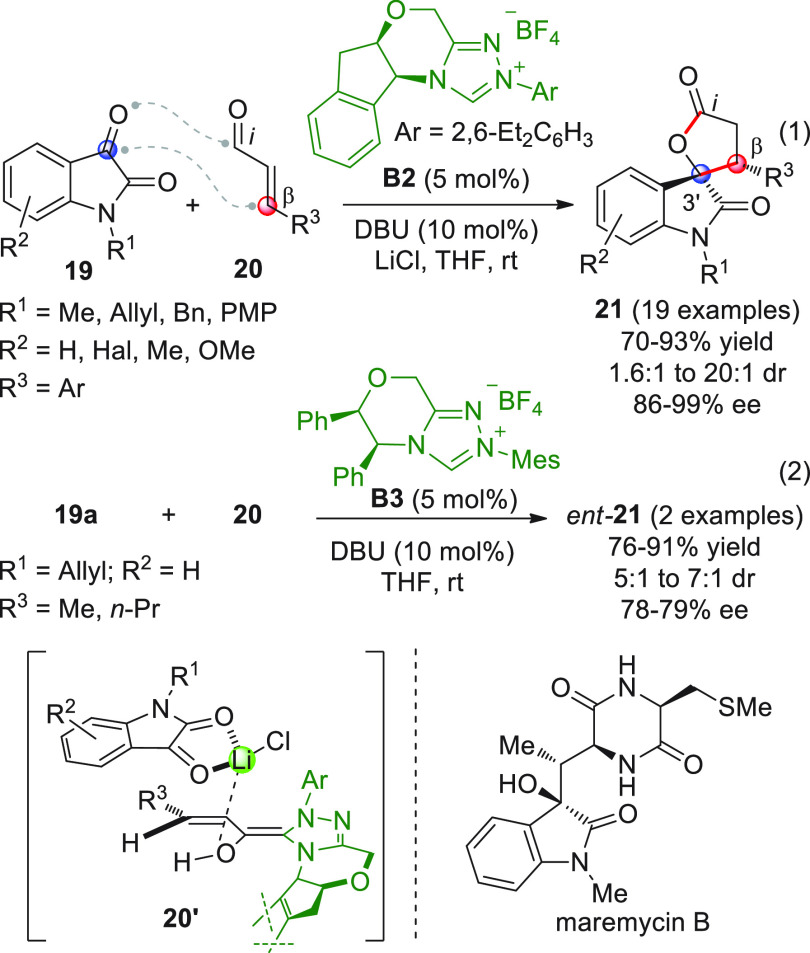


In the first contribution,^54^ the ultimate goal was to demonstrate whether the use of a mild Lewis acid additive together with an NHC catalyst would be able to enhance the reaction performance in terms of both yield and stereoselectivity, as had been previously reported for related additions to activated C=N and C=C systems (vide infra). After extensive screening of diverse Lewis acid/chiral azolium salt couples, it was found that treatment of variously substituted isatins **19** smoothly reacted with β-aryl enals **20** in the presence of triazolium precatalyst **B2** (5 mol %), DBU, and, importantly, lithium chloride (2 equiv), to afford the corresponding spirocyclic oxindoles **21** in good yields and enantioselectivities, as separable mixtures of diastereoisomers at C3′ (poor to very good dr were observed, Scheme 9, eq 1).

Curiously, the Lewis acid additive (LiCl) had a detrimental effect on the reaction involving less reactive β-alkyl substituted enals **20** (R^3^ = Me, *n*-Pr), and new optimization conditions were needed. In this instance, bicyclic triazolium salt **B3** was chosen as the best precatalyst, giving products *ent*-**21** in good yields and opposite facial selectivity (Scheme 9, eq 2). To account for the observed diastereoselectivity in β-aryl substituted enals, a highly coordinated model (see **20′**) was postulated, where the lithium cation coordinates both the enal oxygen atom of the homoenolate and the 1,2-dicarbonyl of isatin; for alkyl derivatives, on the other hand, no definitive explanation was given. Interestingly, one spirocyclic product **21** (R^1^ = R^3^ = Me, R^2^ = H) was transformed to the anticancer agent maremycin B via a five-step procedure, thus emphasizing the synthetic utility of the disclosed [3 + 2] annulation.

**Scheme 10:**
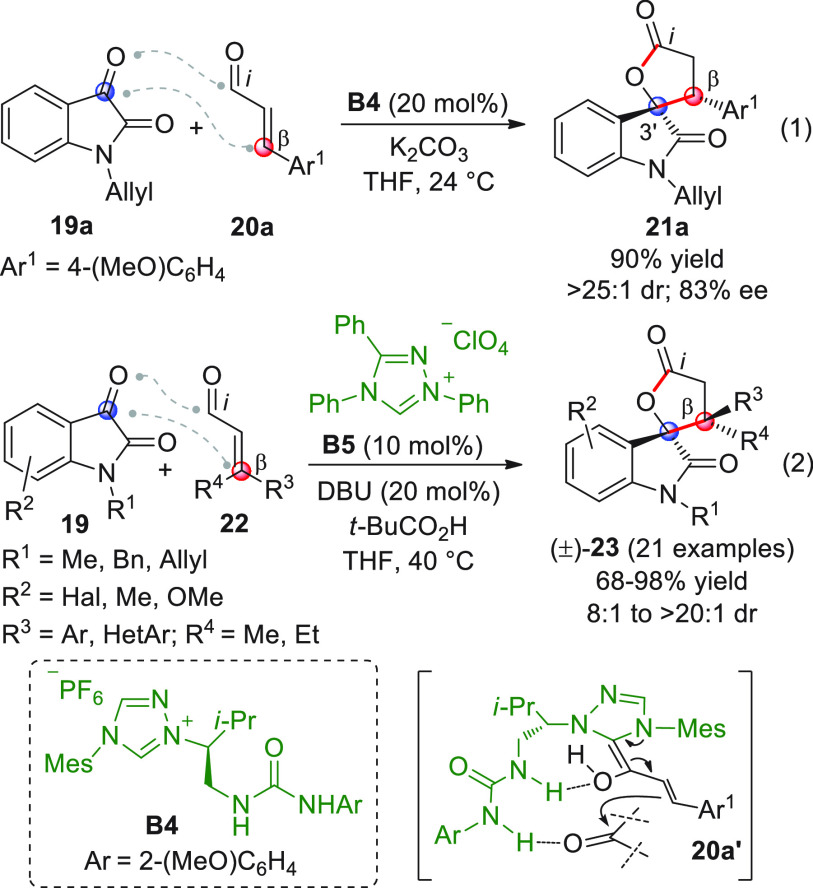


An alternative approach for the same reaction type was devised by Coquerel and co-workers, who synthesized a small library of bifunctional organocatalysts where a chiral 1,3-imidazolin-2-ylidene NHC core is covalently connected to a hydrogen-bond donor group.^55^ The H-bond donor would be able to control the approach of the incoming electrophile and concomitantly stabilize the *E*-configuration of the vinylogous Breslow intermediate, thus anticipating a good overall stereocontrol. Following this line of thought, an extensive initial study of diverse bifunctional NHC catalysts bearing either hydroxyl, guanidine, thiourea, or urea moieties was carried out on several model reactions, to demonstrate the viability of this concept especially in view of the challenging possibility of avoiding self-quenching of the active carbene by intramolecular acid–base reactions. While the reactions involving enals with nitrosobenzene or chalcone acceptors met with only partial success, the γ-lactonization reaction of enal **20a** with isatin **19a** (Scheme 10, eq 1) proceeded efficiently by using the NHC-urea catalyst from **B4**: spiroxindole **21a** was thus obtained in a high yield and diastereomeric ratio, though with modest enantiomeric excess, probably via the hypothesized highly coordinated transition state **20a′**.

A NHC/Brønsted acid dual catalytic system was exploited to perform similar [3 + 2] annulations, involving isatins **19** and scantly used β,β-disubstituted enals **22** (Scheme 10, eq 2).^56^ The delicate issue to be faced here was to activate properly the pronucleophilic, sterically congested β-position of **22** through the expected homoenolate-type reactivity, while suppressing a possible competing γ-site nucleophilic activation toward [4 + 2] annulation (vide infra). The presence of a Brønsted acid cocatalyst (*t*-BuCO_2_H) flanking the catalysis of NHC from **B5** was beneficial for the purpose, providing efficient access to a collection of racemic spirocyclic oxindoles **23** bearing two congested and contiguous quaternary centers with moderate to excellent diastereomeric ratios, probably via a postulated tight transition state where two stabilizing hydrogen bonds arise between the carboxylic acid function of the cocatalyst and the two reacting partners, namely, the isatin carbonyl and the NHC-bound homoenolate hydroxyl (not shown). A couple of examples involving the use of less active, fully aliphatic enal substrates **22** were also reported, but a change in the triazolium precatalyst was needed.

An enantioselective version of the reaction was also performed starting from isatin and β-methylcinnamaldehyde **22** (R^3^ = Ph, R^4^ = Me) and using bicyclic azolium **B3** and the *o*-fluorobenzoic acid agent (1 equiv) (not shown); the reaction gave the corresponding enantioenriched products of type **23** in promising though not excellent results (83% yield, 5:1 dr, 84% ee).

If, instead of being coupled to α-ketolactams such as isatins, an NHC-β-activated enal of the types previously mentioned (e.g., compounds **16** and **20**) is reacted with α-ketolactones such as benzofuran-2,3-diones, a similar [3 + 2] annulation will occur, and entry to interesting spiro-bis-lactones is secured. This strategy was devised and exploited independently by the Cheng^57^ and Nair^58^ research groups in 2013 and 2014.

**Scheme 11:**
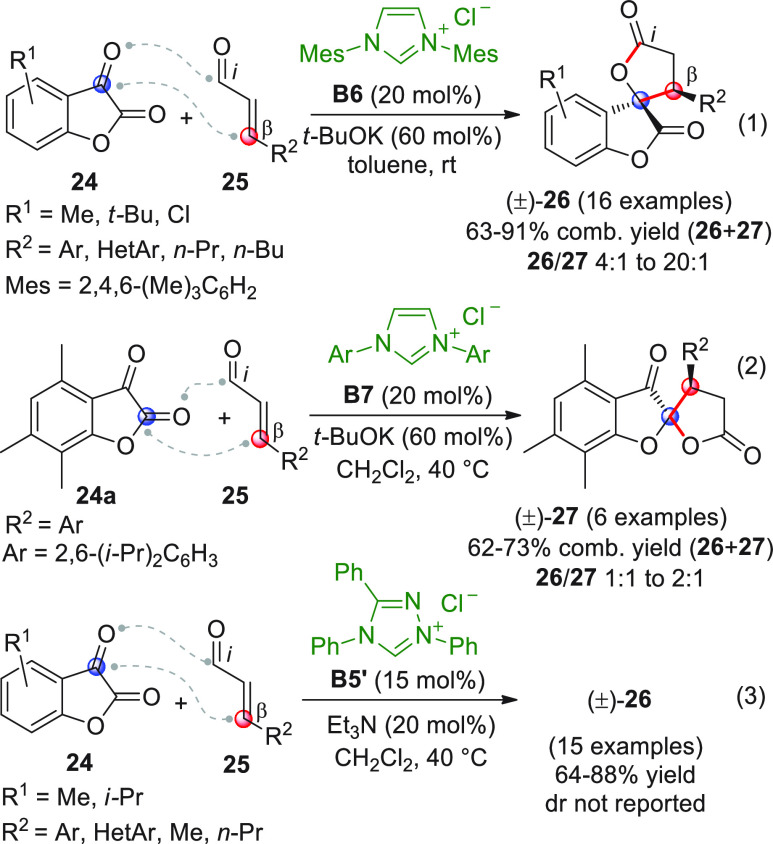


In the first report, β-aryl- or β-alkyl enals **25** were treated with achiral imidazolium salt **B6** (20 mol %) in *t*-BuOK and were reacted with diverse benzofuran-2,3-diones **24** (Scheme 11, eq 1) giving rise to the expected [3 + 2] annulation products (±)-**26** as single diastereoisomers, accompanied by variable quantities of the regioisomeric spirocycles (±)-**27**, which were the result of a rather unexpected attack of the NHC-bound homoenolate on the lactone carbonyl (Scheme 11, eq 1).^57^ Using different NHC precursors (**B6** vs **B7**), solvents, and reaction temperatures, optimized conditions were found to regiodivergently access predominantly either (±)-**26** or (±)-**27** (Scheme 11, eq 1 vs eq 2), though in the latter case the regioisomeric selectivity remained poor. Access to (±)-**26** was also attained using an alternative thiazolium salt precatalyst (not shown), in which case the regioselectivity in favor of **26** was excellent (**26**/**27** > 20:1) but isolated yields were moderate (40–62% range).

In the second work (Scheme 11, eq 3), the reaction was performed using NHC precatalyst **B5′**; in this case, however, despite similar reaction conditions, only spiro-bis-lactones of type (±)-**26** were reported, without any mention of possible byproducts of type (±)-**27**.^58^ Isolated yields of **26** were good, though no diastereomeric ratios were documented. In both examples, no attempts to translate the transformation into a chiral nonracemic format were made.

**Scheme 12:**
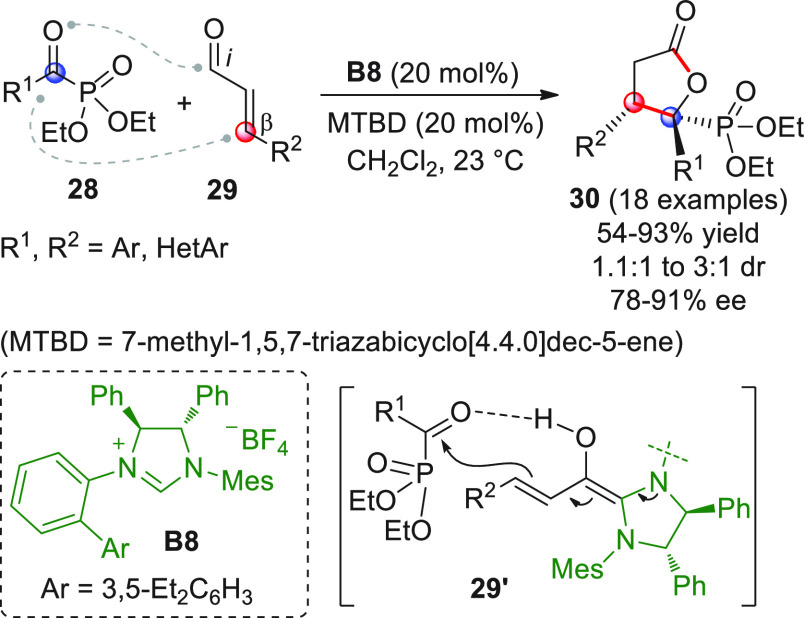


The asymmetric NHC-catalyzed formal [3 + 2] annulation of α,β-unsaturated aldehydes **29** with acyclic α-ketophosphonates **28** was documented by Scheidt et al. en route to the synthesis of unprecedented enantioenriched γ-butyrolactones **30** bearing a phosphonate moiety (Scheme 12).^59^ Initial DFT-based computational investigation of a model reaction guided the design of tailored *C*_1_-symmetric NHC precatalyst with the intention of selecting the optimal catalyst structure, capable of maximizing the stabilization of the transition state leading to the major enantiomer product, while maximizing the destabilization of the minor enantiomer. Azolium precatalyst **B8** resulted in the best candidate, which was used in the realization of the product panel **30**. The scope of the reaction was fairly broad, including aryl- and heteroaryl-substituents on both the donor and acceptor components. Many diverse products **30** were isolated in good yields and enantiomeric excesses, though *cis/trans* diastereoselection was poor or moderate. As a limitation, alkyl-substituted substrates did not provide good levels of efficiency. Based on computational modeling, a highly organized transition state was proposed, featuring H-bonding between the ketone oxygen within **28** and the enol function of the extended Breslow intermediate **29′**.

**Scheme 13:**
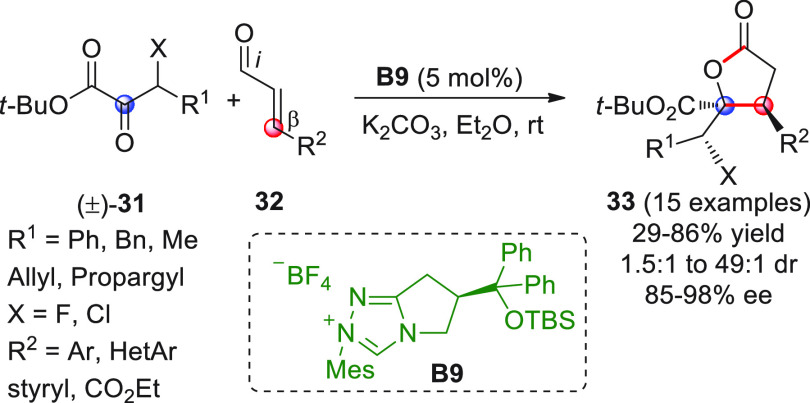


The asymmetric NHC-catalyzed homoenolate-mediated reaction of α,β-unsaturated enals **32** was cleverly exploited in the reaction with racemic β-halo α-keto esters of type (±)-**31** to produce the corresponding chiral nonracemic γ-butyrolactones **33** via dynamic kinetic resolution (Scheme 13).^60^

Catalyst optimization was performed in order to channel the reaction path toward the intended [3 + 2] annulation and prevent possible competitive side reactions such as cross-benzoin coupling. Apart from a few exceptions, deploying the catalyst from **B9** generally furnished lactones **33** in good yields and stereoselectivities.

**Scheme 14:**
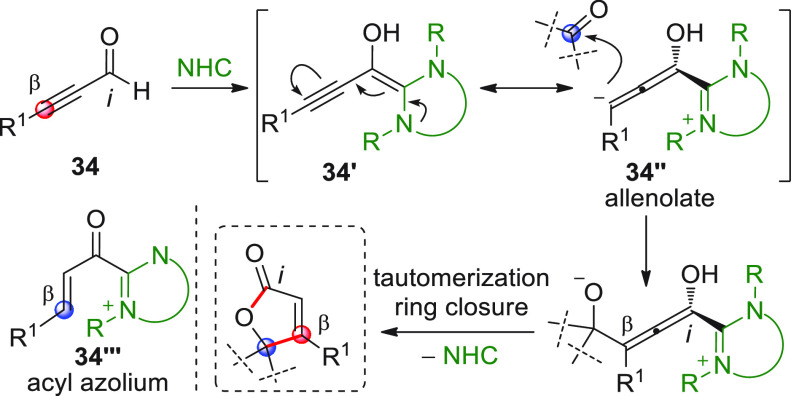


In strict analogy with the disclosed homoenolate-based [3 + 2] annulations involving enal substrates, replacement of the enal with a linear conjugated ynal of type **34** (Scheme 14) and subsequent NHC activation may lead to the corresponding homoenolate **34′**—an allenolate equivalent—which may conveniently be coupled in a vinylogous fashion (β-position) to an electrophile such as a ketone, giving rise to valuable γ,γ-disubstituted butenolide structures. Putting this concept into practice may encounter difficulties, due to the weak nucleophilicity of the allenolate and the competitive, facile redox transfer through protonation of the homoenolate/allenolate to forge an α,β-unsaturated acyl azolium of type **34‴** with reverted (electrophilic) *ipso*/β polarity.^61^ This issue was cleverly handled independently by three research groups in the 2013–2014 biennium, by employing cooperative NHC-Lewis acid or NHC-Brønsted acid catalysis in order to enhance the reactivity of both substrates and hopefully coordinate them in organized transition states.^62−64^

**Scheme 15:**
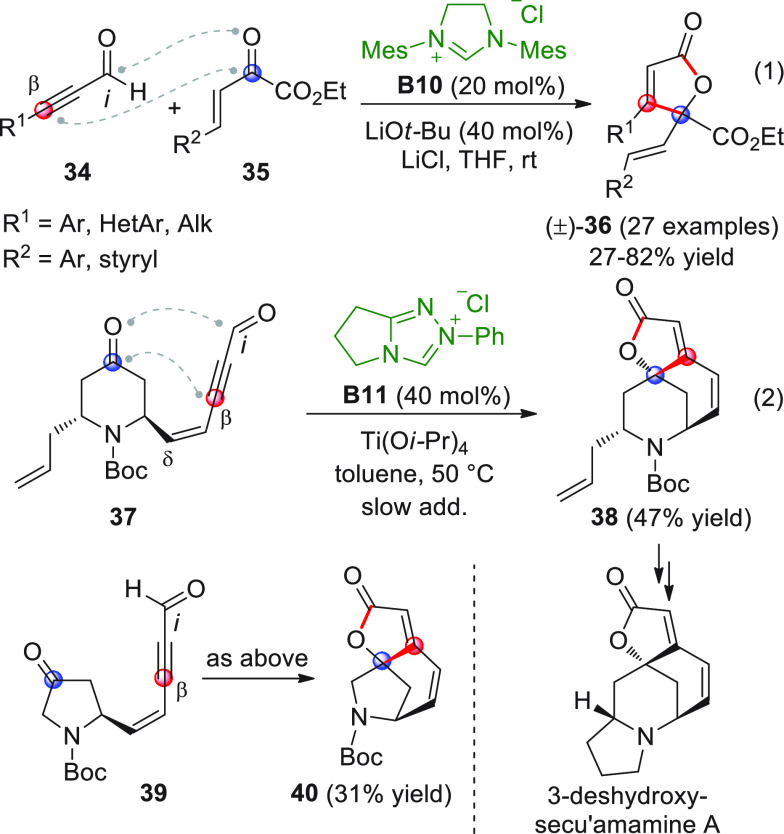


The first study, performed in a nonasymmetric setting, reported the reaction of ynals **34** with unsaturated α-keto esters **35** under NHC catalysis from **B10** (20 mol % loading) together with the mediation of LiCl (1 equiv) as an indispensable Lewis acid ingredient (Scheme 15, eq 1).^62^ The butenolide products (±)-**36** were obtained in generally good yields with both aromatic and aliphatic ynals, while the keto ester component was restricted to nonenolizable aryl (or phenylvinyl)-substituted ketoesters **35**.

The utility of this [3 + 2] annulation in an intramolecular, diastereoselective setting was soon after demonstrated by Snyder and co-workers, who successfully forged the fused polycyclic core, shared by *securinega* alkaloids, in one step starting from ad hoc-prepared enynal ketone precursors **37** and **39** (Scheme 15, eq 2).^63^ The challenge here was even harder than in the previous example, since an enolizable ketone moiety is present in the starting substrates (**37** and **39**), and competitive intramolecular addition reactions or even hypervinylogous versions involving the δ-site could, in principle, occur. Nonetheless, conditions were found to perform the transformation successfully, namely, slow addition (over 8 h) of **37** or **39** to a preformed suspension of precatalyst **B11** and Ti(O*i*-Pr)_4_ (2 equiv) in toluene, which ensured preparation of the corresponding tricyclic butenolides **38** or **40** in 47% and 31% yields, respectively. It was demonstrated that the efficiency of this annulation strongly depended upon the conformational bias of the enynal starter, since carrying out the same reaction on a simplified model (e.g., des-allyl compound **37**) produced the butenolide product in 91% yield (not shown). Compound **38** was easily elaborated in two steps to the 3-deshydroxy-secu’amamine A target, an analogue of natural secu’amamine A.

**Scheme 16:**
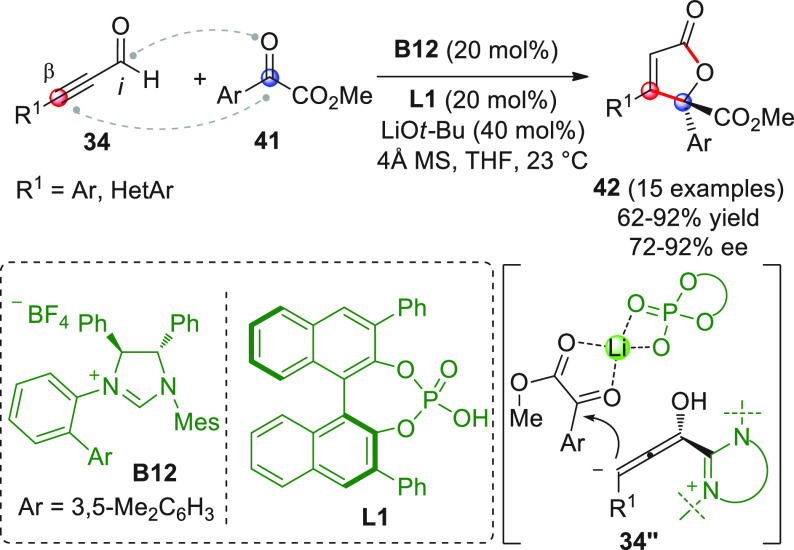


The enantioselective version of this transformation was developed by the Scheidt group, who coupled aromatic or heteroaromatic alkynyl aldehydes **34** with aromatic α-ketoesters **41**, to give enantioenriched butenolides **42** through chiral NHC/chiral phosphate cooperative catalysis (Scheme 16).^64^

Key to the success of this endeavor was the new mode of cooperative catalysis centered on the combination of three components: (i) the Hoveyda *C*_1_-symmetric biaryl saturated imidazolium salt **B12** as the chiral NHC-precatalyst, (ii) the lithium *tert-*butoxide base which activates the NHC precatalyst while furnishing the coordinating lithium ion, and (iii) a chiral Brønsted acid **L1** (20 mol %) serving as a cocatalyst. Though the specific roles of each component of the catalyst triad were not fully delineated, it was proposed that the lithium ion acts as a Lewis acid capable of coordinating both the BINOL-derived phosphate and the α-ketoester (as shown in **34″**), thus ensuring the observed yields and enantioselectivities of the butenolide products.

**Scheme 17:**
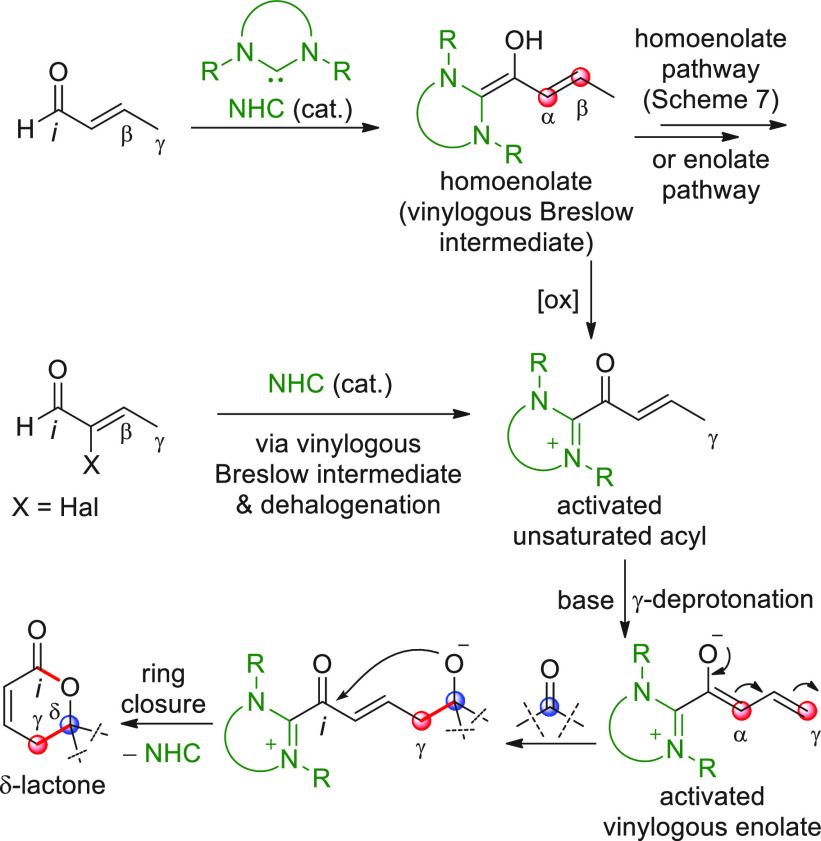


Besides the β-sp^2^-CH nucleophilic activation of enals (and ynals) via in situ generated homolenolates (Scheme 7), the inherently multifaceted chemistry, triggered by NHC catalysis, may well allow for the nucleophilic activation of γ-sp^3^-CH bonds starting from, for example, γ-enolizable enals or α-haloenals, via oxidatively generated vinylogous enolates (Scheme 17). Upon interception of such extended enolates with an electrophile—e.g. an activated carbonyl—and subsequent ring closure, δ-lactone products are accessed according to a formal [4 + 2] annulation, while the NHC is recovered for the next catalytic cycle.

Significant regioselectivity issues are posed here, which are seen to depend on the switching from the homoenolate pathway (unveiling Cβ- or even Cα donor sites)^65^ to the vinylogous enolate pathway, which unmask the competitive Cγ/Cα nucleophilic sites. In addition, pointing on the γ-functionalization may pose questions about effective stereocontrol, given the distance between the NHC chiral inducer and the remote γ-site.

**Scheme 18:**
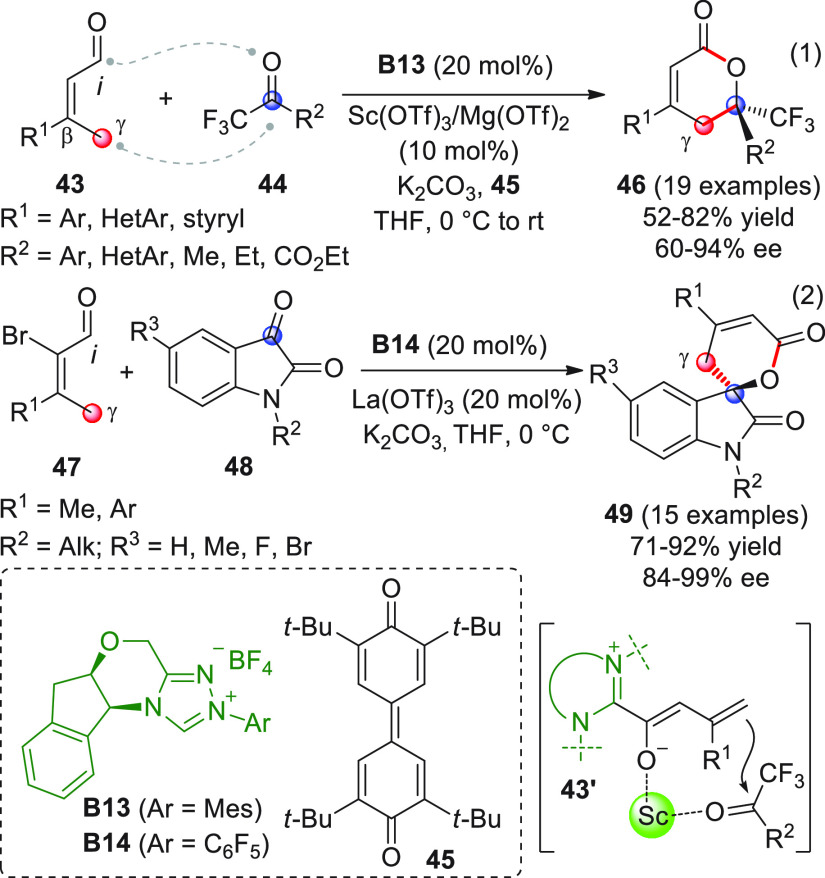


Capitalizing on outstanding precedents on the γ-functionalization of enals via dienamine catalysis (vide supra) as well as NHC-catalyzed γ-activation of α,β-unsaturated ketenes, Chi et al. documented, for the first time, in 2012 the enantioselective γ-addition of remotely enolizable enals **43** to activated ketones **44** via oxidatively generated NHC-bound vinylogous enolate intermediates in cooperation with Lewis acid cocatalysis (Scheme 18, eq 1).^66^ To skip the homoenolate pathway, biased enal substrates were used, bearing bulky and nonenolizable β-aryl (or heteroaryl, arylvinyl) substituents. The combined use of NHC from **B13** (20 mol %) and the two Lewis acids Sc(OTf)_3_ and Mg(OTf)_2_, together with potassium carbonate as the base and quinone **45** as the external oxidant, provided the optimal conditions to consign the δ-lactone products **46** in good yields and modest-to-good enantioselectivities. Mechanistic studies were not performed, but given the indispensable role of the Lewis acids in drastically improving enantiofacial discrimination, the authors proposed that a multisite coordination as in **43′** might occur, where the scandium ion is capable of bringing the ketone acceptor in close proximity to the chiral NHC dienolate.

A similar formal [4 + 2] annulation was carried out by Yao and co-workers,^67^ who utilized α-bromo-α,β-unsaturated aldehydes **47** as γ-enolizable starter molecules.^68^ In this case, debromination from the extended Breslow intermediate is operative (see general Scheme 17), providing the key dienolate intermediate without the need of an external oxidant reagent. Thus, treating enals **47** with isatins **48** in the presence of catalytic NHC precursor **B14**, catalytic quantities of lanthanium triflate as a cocatalyst, and the carbonate base, afforded spirocyclic oxindole-dihydropyranones **49** with good efficiency in terms of both yields and enantioselectivities (Scheme 18, eq 2). As in the previous work by Chi, a tightly coordinated transition state was proposed, where the lanthanium ion coordinates the carbonyl acceptor thus enhancing the chiral induction exerted by the NHC catalyst.

A similar reaction scheme was performed some years later by Yang, Zhong, and colleagues using β-phenylcrotonaldehyde donors and *N*-deprotected isatin acceptors (not shown).^69^ In this case, a cooperative catalysis between NHC and Brønsted acid (pivalic acid) resulted effective in producing the corresponding spiroindoline pyrans similar to compounds **49** in generally good yields and moderate to high enantiomeric excesses. A further study on this subject was developed soon after, focusing on the chiral NHC-catalyzed [4 + 2] annulation between β-aryl or β-methyl crotonaldehydes and isatins (not shown).^70^ In the absence of any additional cocatalyst and under oxidative conditions, the reactions proceeded successfully giving, again, the corresponding spiroxindole δ-lactones of type **49** in good yields and enantioselectivities.^71^

**Scheme 19:**
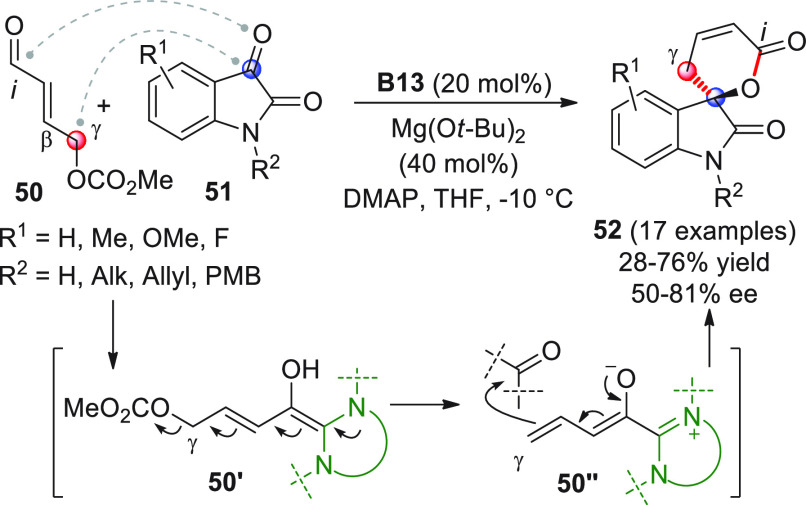


As already stated, the NHC-catalyzed formal [4 + 2] annulation involving the γ/*ipso* carbon sites of γ-enolizable enals proved viable when the vinylogous enolate pathway prevails over the homoenolate pathway, and this was attained by placing biasing β-substituents in the starting enals (e.g., Scheme 18). As a clever alternative, Ye and collaborators were able to perform this chemistry by exploiting β-unsubstituted-γ-preoxidized enals, which are able to generate the expected unsubstituted dienolates in situ, en route to the formation of the δ-lactone targets (Scheme 19).^72^

After careful experimentation, optimal conditions were found [NHC precatalyst **B13**, Mg(O*t*-Bu)_2_] to channel the coupling reaction of enal carbonate **50** with isatins **51** toward the desired spirocyclic oxindolo dihydropyranones **52**, while completely suppressing the competitive homoenolate-mediated [3 + 2] annulation. The efficacy of the reaction was quite good for many diversely substituted isatins **51**, with the sole exception of *N*-Boc and *N*-Cbz protected isatins, which proved completely unreactive under these reaction conditions.

**Scheme 20:**
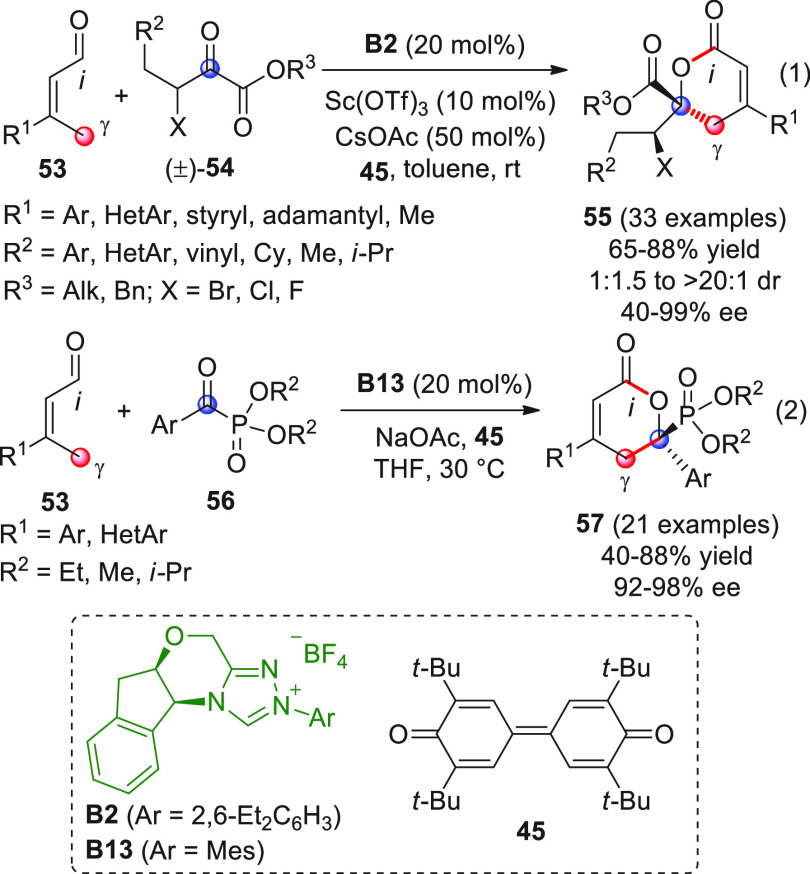


The cooperative oxidative catalysis by a chiral NHC and a Lewis acid was also exploited for the dynamic kinetic resolution of racemic α-ketoesters to forge enantioenriched δ-lactone products (Scheme 20, eq 1).^73^

The optimized reaction conditions (precatalyst **B2**, scandium triflate cocatalyst, cesium acetate as the base, and quinone **45** as the external oxidant) were applied to a considerable number of substrates, where both the β-substituted crotonaldehydes **53** and the racemic ketoester components (±)-**54** could tolerate several substituent variables. The corresponding δ-lactones **55** were obtained in good isolated yields, very high diastereomeric excesses, and good levels of enantioselectivity on almost all occasions. Just a few cases met with limited success, namely, the reactions using fluoroderivatives **54** (X = F) or methyl-crotonaldehyde **53** (R^1^ = Me).

The NHC-catalyzed [4 + 2] annulation strategy was also recently applied for the enantioselective entry to 2-pyranylphosphonates **57** by coupling γ-enolizable enals **53** to α-ketophosphonates **56** (Scheme 20, eq 2) (for the analogous NHC-catalyzed [3 + 2] annulation involving α-ketophosphonates, see Scheme 12).^74^ In this case, the use of NHC from **B13** and quinone oxidant **45** was sufficient for triggering the reactions with high efficiency and enantioface discrimination without the need for additional cocatalysts, probably due to the unique stereoelectronic properties of the ketophosphonate substrates. With the exception of alkyl derivatives (R^1^ = cyclohexyl or methyl within enals **53**, or alkyl phosphonates of type **56**, not depicted in the scheme), all other substrates **53** and **56** were successfully coupled giving very good results. The chiral δ-lactone products **57** were assayed in vitro for their antibacterial and antiviral activities and some of them showed promising results for potential use in plant protection.

##### 3.1.1.2 Cyclic Pronucleophiles

The set of cyclic unsaturated aldehydes for use in direct vinylogous aldol additions (VARs) is much smaller than that of their acyclic counterparts, and the reported studies are more recent. This may be due to the particular difficulties of catalytically activating cyclic substrates in situ, especially when they possess aromatic properties.

**Scheme 21:**
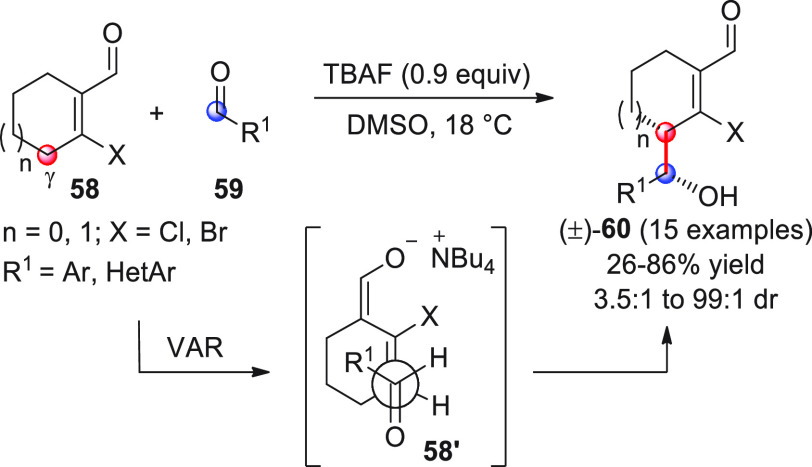


The only example dealing with nonaromatic substrates is the work by Gong and co-workers, who documented how cyclic β-haloenals of type **58** could undergo TBAF-triggered direct VAR with aromatic aldehydes **59**, giving racemic δ-hydroxy-β-haloenals (±)-**60** (Scheme 21).^75^

Among the tested bases (DBU, DABCO, Et_3_N, DMAP, and others), only fluoride ion (as in TBAF) or hydroxide (TMAH) proved competent reagents, and indeed almost stoichiometric quantities of TBAF at 18 °C were needed to afford the products in appreciable yields and *syn*-diastereoselectivities. Raising the reaction temperature to 28–32 °C led to decreased isolated yields of the aldol products in favor of dehydrated aldol condensation byproducts. The *syn* diastereopreference of the products was hypothesized as deriving from the transition state **58′** with an *endo*-antiperiplanar disposition of the two reacting partners. The minor *anti*-configured diastereoisomers were seen to derive from an antiperiplanar disposition in the transition state where unfavorable steric interactions between the X and R^1^ substituents occurred (not shown). No mention, however, was made by the authors for any possible, favorable synclinal transition states alternative to **58′** (with R^1^ pointing away from the cyclohexene ring) giving the observed *syn*-products **60**. It is worth noticing that complete γ-site selectivity was attained in all cases, as opposed to the α-selectivity observed in another study by the same researchers,^76^ where identical substrates were coupled to Michael acceptors under TBAF agency, indicating that the γ/α regioselectivity of the nucleophilic addition for these cyclic dienolates is strongly dependent on the electrophilic counterpart involved in the process.

**Scheme 22:**
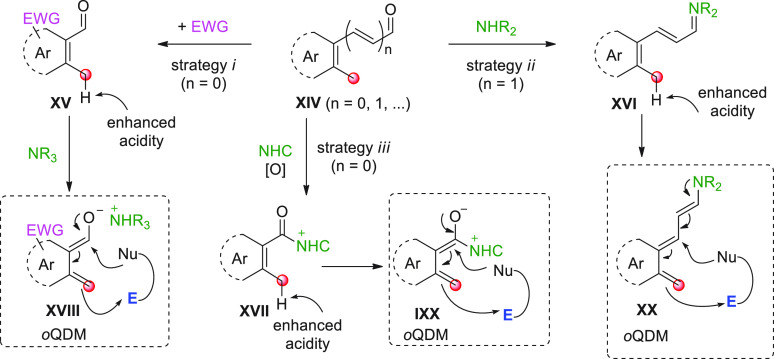


The benzylic C(sp^3^) sites of *ortho*-methyl substituted aromatic carbaldehydes of type **XIV** (*n* = 0, Scheme 22) or extended polyenals **XIV** (*n* ≥ 1) may be envisaged as remotely enolizable sites of the vinylogous aldehyde system so much so that useful functionalization chemistry with suitable acceptor components may be anticipated. However, deprotonation of such benzylic positions, especially when carbocyclic aromatic rings are involved, is far from trivial, since it generates highly reactive and unstable *ortho*quinodimethane species (*o*QDM), particular polyenolate donors, where the aromatic character of the original ring is temporarily lost. To enhance the acidity of protons at these benzylic positions, clever solutions were devised, either by strategically placing electron-withdrawing groups within the aromatic ring [strategy (*i*), Scheme 22], by activating the carbonyl function via covalent iminium/enamine organocatalysis [strategy (*ii*)], or by NHC-organocatalysis [strategy (*iii*)]. In either instance, the corresponding HOMO-raised polyenolate-*o*QDM dearomatized species (e.g., **XVIII**, **XIX**, **XX**) are formed, which can engage in remote benzylic functionalization with suitable electrophiles, often leading to cycloannulation products. The driving force of these processes is the thermodynamic stability of the products, which recover the aromatic properties, while further being stabilized within new 5- or 6-membered rings. In all cases, be they concerted or stepwise annulations, the regioselectivity of the processes is granted by the transmission of the electronic effects of the carbaldehyde through the conjugated π-system, exquisitely according to the vinylogy principle. The examples that follow in this subsection will deal with the exploitation of these concepts (strategies *i* and *iii*) for additions to C=O bonds, while additions to C=N and C=C bonds (strategies *i*, *ii*, and *iii*) will be treated in the competent subsections.

**Scheme 23:**
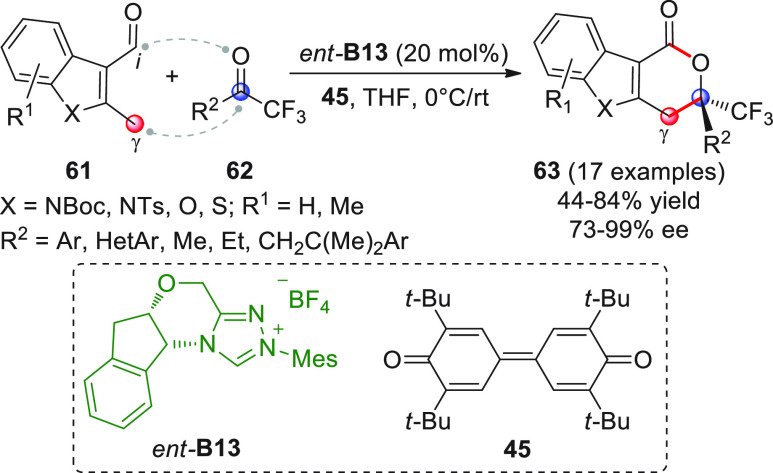


Capitalizing on precedent works on NHC-based activation of aldehydes and *o*QDM generation via trienamine activation (vide infra), Chi and co-workers successfully realized for the first time the functionalization of benzylic C(sp^3^)–H bonds of heteroaryl aldehydes through NHC organocatalysis.^77^ They started from 2-methyl-3-carboxaldehydes of indole, benzofuran, and benzothiophene of type **61** (Scheme 23) and coupled them with activated ketones such as trifluoromethyl ketones **62** (or isatins, not shown here) in the presence of NHC precatalyst *ent*-**B13** (20 mol %) under oxidative conditions. The corresponding δ-lactone products **63** were obtained, as emerged by the formal [4 + 2] annulation involving an NHC-bound acylazolium of type **XVII** and *o*QDM intermediate of type **IXX** (see Scheme 22).

The experimental conditions were slightly adapted depending upon the nature of ketones **62** (R^2^ = aryl vs alkyl), affording the heterocyclic products **63** in good yields (apart from a couple of recalcitrant acceptors) and enantiomeric excesses. It is worth noticing that this procedure could not be extended to simple aromatic aldehydes (e.g., 2-methylbenzaldehyde) which were oxidized under the oxidative conditions of the reaction, nor could it be adapted to prostereogenic indole substrates where the methyl group at C2 within **61** was replaced by benzyl or CH_2_CO_2_Et groups.

Almost the same concept was applied by these authors using indole substrates with “inverted” substituents, namely, starting from 3-methyl 2-formylindoles (not shown).^78^ The reaction of these substrates with ketones such as trifluoromethyl ketones and α-ketoesters under NHC-catalysis delivered the corresponding racemic hydropyranoindoles in useful yields. Unfortunately, any attempts to translate this transformation into a nonracemic setting using chiral NHC did not yield appreciable results.

As already mentioned, in situ formation of *o*QDM species, derived from benzene precursors, is much more difficult than that of corresponding heteroaromatic precursors, given the higher degree of aromaticity to be temporally broken.

**Scheme 24:**
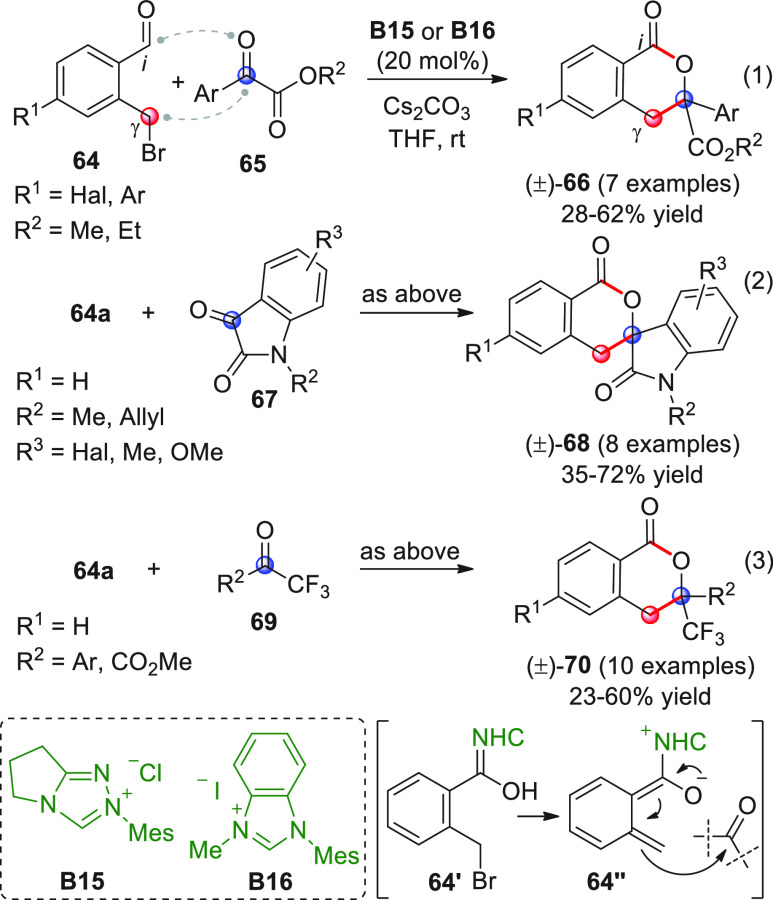


In 2016, Glorius et al. succeeded in the realization of this goal, by using NHC catalysis and *ortho*-bromomethylbenzaldehyde **64** as the starting materials (Scheme 24).^79^ In this instance, the presence of a leaving group (the bromine atom) facilitated the in situ generation of the NHC-bound dienolate *o*QDM **64″** through elimination of HBr from the Breslow intermediate **64′**, thus circumventing the need for oxidation to acylazolium species and γ-deprotonation.

Coupling with activated ketones such as aryl glyoxylates (eq 1), isatins (eq 2), or trifluoromethyl ketones (eq 3) under uniformed reaction conditions (NHC from **B15** or **B16** and cesium carbonate) invariably produced the [4 + 2] annulation products **66**, **68**, or **70**, respectively, in low to moderate yields in a racemic format. An attempt to use chiral NHC was performed on trifluoromethyl acetophenone (not shown), leading to a 48% ee of the δ-lactone product.

**Scheme 25:**
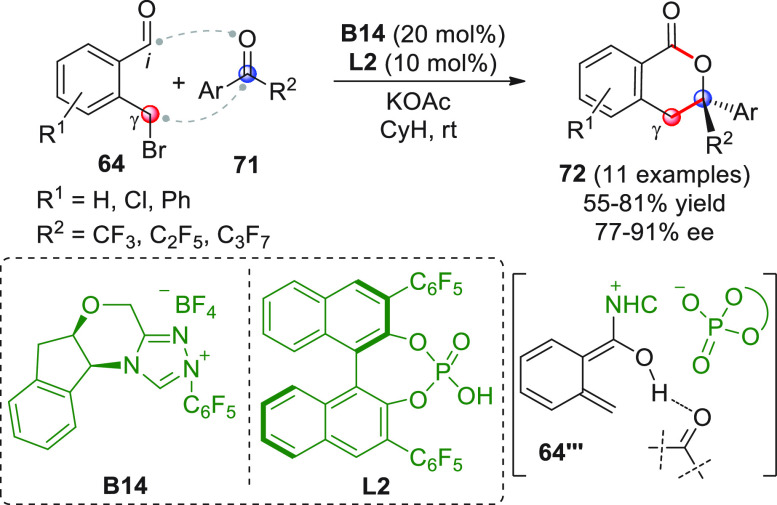


Almost during the same period, Rovis and Chen developed a chiral version of this reaction using chiral NHC/Brønsted acid cooperative catalysis.^80^ Thus, starting with bromomethyl benzaldehydes **64** and perfluoroalkyl aryl ketones **71** as the substrates, the combined use of chiral NHC from **B14** and BINOL-derived chiral phosphoric acid **L2** gave the desired products **72** with good results (Scheme 25). As for the ketone scope, electron-withdrawing substituents on the R^2^ aryl group generally decreased enantioselectivities, while alkyl derivatives failed to give any products.

Given the substantial role of the Brønsted acid cocatalyst in improving the enantioselectivity in a matched sense (a combination of *ent*-**B14**/**L2** was less effective), a mechanism was proposed, where the Breslow intermediate undergoes transformation into an *o*QDM-dienol **64‴** with the formation of an ion pair between chiral triazolium NHC and chiral phosphate counterion, which dictates the enantioface discrimination of the incoming carbonyl. In the absence of mechanistic studies, the actual role of the acid as either a Brønsted acid, chiral counterion, or phase transfer promoter for the NHC catalyst remained undefined.

**Scheme 26:**
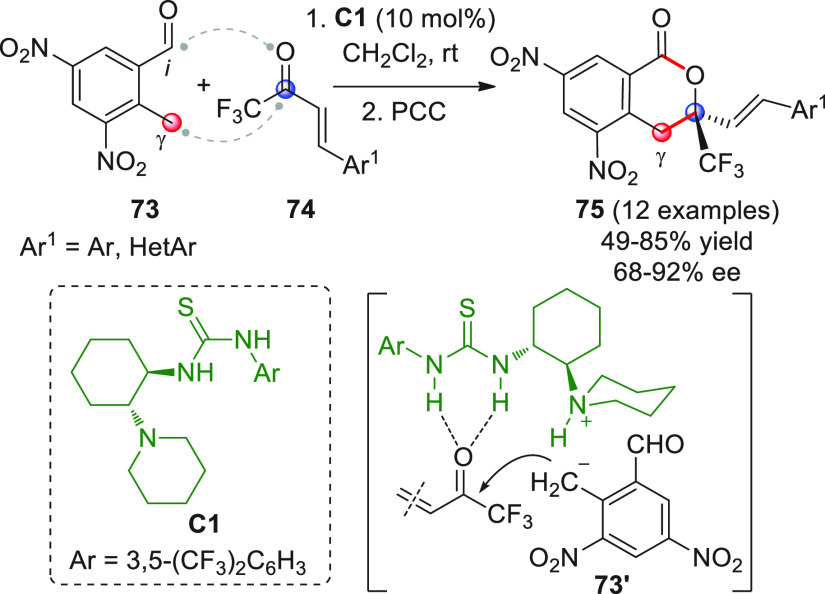


According to the concepts reported above (Scheme 22, strategy *i*), the introduction of electron-withdrawing groups such as nitro groups at *ortho*- and/or *para*-positions of 2-methylbenzaldehyde should enhance the acidity of the methyl protons, thus facilitating remote deprotonation and generation of highly reactive *o*QDM species. In 2017, Li et al. developed a domino asymmetric benzylation/aldol hemiacetalization reaction involving 2-methyl-3,5-dinitrobenzaldehyde (**73**) and β-aryl- (or heteroaryl)-substituted α,β-unsaturated trifluoromethyl ketones **74** (Scheme 26).^81^ The reaction was catalyzed by the tertiary amine/thiourea bifunctional organocatalyst **C1** (10 mol %) and provided the corresponding 3,4-dihydroisocoumarin products **75** after oxidation (PCC). It was found that both electron-withdrawing and electron-donating substituents on Ar^1^ were well tolerated, but yields and enantioselectivities decreased when bulky (e.g., naphthyl) or *ortho*-positioned groups were used. Based on the experimental results, the authors proposed a transition state model of type **73′** where the benzylic pronucleophilic site of **73** is deprotonated by the tertiary amine catalyst forming a chiral ion pair, while the ketone carbonyl of **74** is activated and positioned by hydrogen bond interaction with the thiourea moiety of the catalyst. Unfortunately, control experiments using benzylic substrates without either nitro groups or carbaldehyde groups were not performed, which would have furnished further information about the actual role of these groups in the reaction.^82^

#### 3.1.2 Indirect Procedures

The vinylogous addition of silicon-stabilized dienolates to C=O bonds, namely, the vinylogous Mukaiyama aldol reaction (VMAR), was introduced by Mukaiyama in 1975 using crotonaldehyde-derived silyl enol ether and cinnamaldehyde dimethyl acetal with TiCl_4_ as the Lewis acid promoter.^83^ Since then, research in this field has flourished, centered upon the application of stereoselective VMAR especially in the synthesis of polyketide-related natural products. Indeed, before the advent of direct HOMO-raising catalytic modalities triggering regioselective remote functionalization (see direct procedures), the VMAR represented the most common and orthodox way to carry out the aldol addition reaction in a vinylogous fashion.^4^ During the period covered by this review article, most of the silyl-based vinylogous reactions are associated with ester- or amide-derived nucleophiles, while a few examples deal with the use of aldehyde- or ketone-derived acyclic silyl enol ethers.

##### 3.1.2.1 Acyclic Nucleophiles

**Scheme 27:**
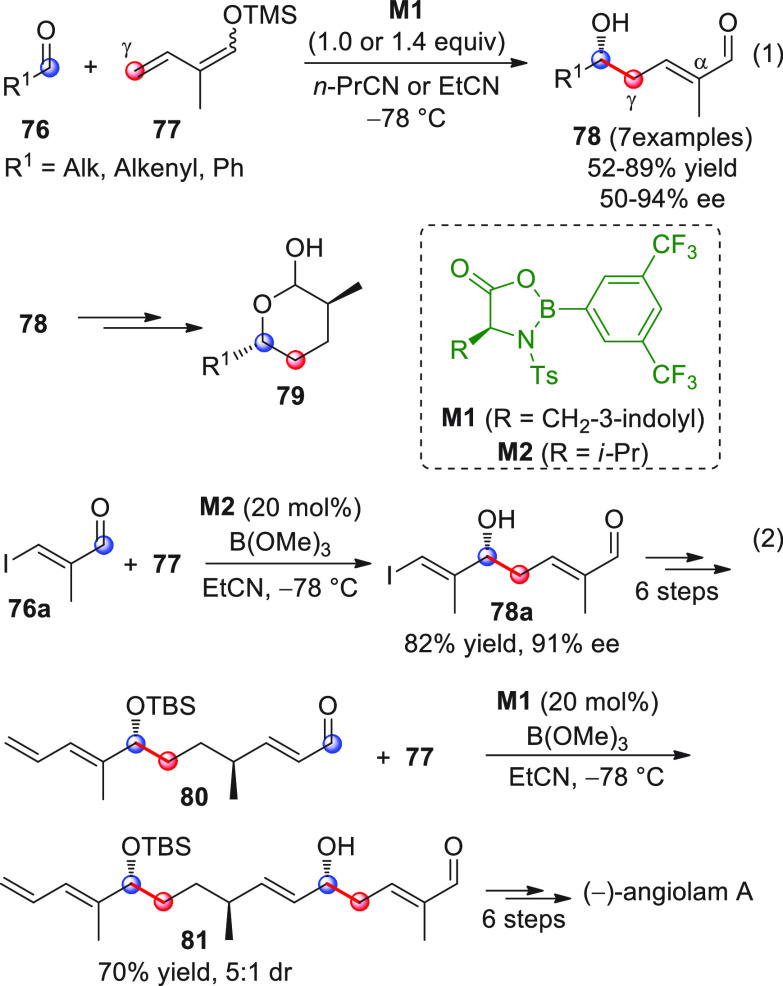


The first enantioselective VMAR of aldehyde-derived dienolates was documented by Kalesse and Gieseler in 2011.^84^ Based on precedent results on the use of amino acid-derived oxazaborolidinones (OXB) in VMAR with ester-derived ketene acetals,^85^ they carried out the VMAR between preformed silyl dienol ethers **77** and aldehydes **76** utilizing the chiral OXB promoters of type **M1** (Scheme 27, eq 1).

Various aldehyde substrates could be used successfully, including both aliphatic (i.e., cyclohexyl, iodoalkenyl, silyloxyalkyl, ...) and aromatic aldehydes, giving the corresponding δ-hydroxy-α-methyl enals **78** in satisfactory yields and variable levels of enantioselectivities. In any case, the chiral OXB Lewis acid had to be added in stoichiometric (or more) quantities (1.0–1.4 equiv range). The VMAR products **78** could be used as the precursors in various transformations. For example, the conjugate addition of hydrides followed by internal stereoselective α-protonation could give rise to hemiacetals **79**.^86^

The sequence of VMAR followed by hydride reduction and α-protonation was exploited by the same authors twice during the elegant total synthesis of the naturally occurring antiobiotic (−)-angiolam A (Scheme 27, eq 2).^87^ In this instance, the two key VMARs involving first **76a** + **77** and then **80** + **77** proceeded with two different OXB promoters (**M2** and **M1**) and afforded the corresponding aldol products **78a** and **81** in good yields and high stereoselectivities. The addition of trimethylborate as a competitor for binding the product to the chiral Lewis acid led to improved turnover numbers and allowed a catalytic loading of the OXB.

**Scheme 28:**
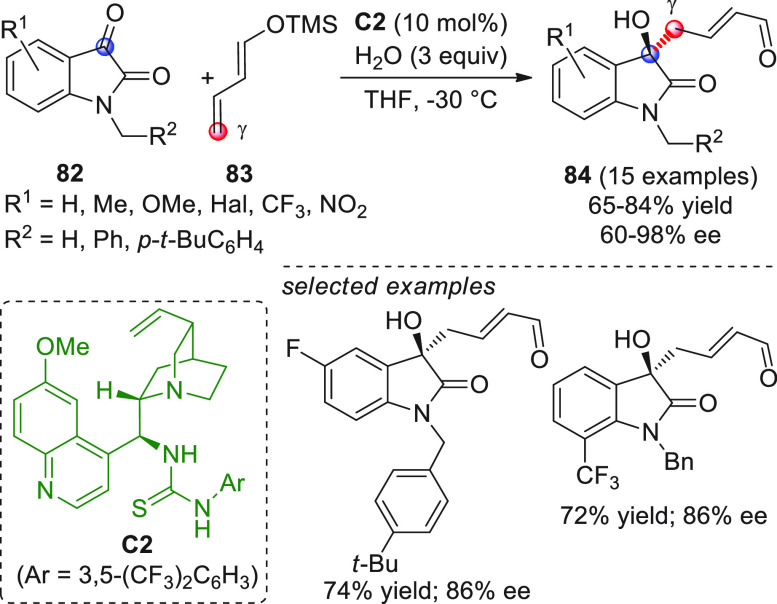


In the previous examples, the chiral metal-based catalyst (or promoter) acts as a Lewis acid capable of lowering the LUMO of the acceptor component, while not intervening with the silyl enolate substrate. Over the past decade, organocatalyzed asymmetric VMARs have been introduced and widely exploited, where a metal-free organocatalyst is able to activate either the acceptor or donor component, or both, as in the case of bifunctional organocatalysts. Despite the numerous reports in this field dealing with ester-derived silyl ketene acetals, we had to wait until 2018 to witness the first (and sole) enantioselective vinylogous aldol addition reaction involving aldehyde-derived silyl dienol ether donors and isatin electrophiles under bifunctional organocatalytic conditions (Scheme 28).^88^

Alemán and co-workers found that variously substituted isatins **82** could efficiently couple to silyl dienol ethers **83** using cinchona-thiourea catalyst **C2** (10 mol %) and a controlled quantity of water (3 equiv) affording the respective products **84** with exclusive γ-site selectivity and good-to-very good levels of enantioselectivity. The role of water was crucial: in the absence of water, almost no conversion occurred, whereas an excess of water (6 equiv) led to hydrolysis of **83** and overall decrease of reaction efficiency. The authors affirmed that water played an important role in triggering the aldol reaction and in the catalyst interaction, but they did not enter into the specific study of the reaction mechanism. They did, however, refer to a previous work they had conducted dealing with the nonvinylogous, α-regioselective coupling of silyl dienol ethers **83** with nitroolefins under bifunctional organocatalysis in the presence of water as an indispensable ingredient (not shown).^89^ In that case, experimental studies and DFT calculations corroborated the conclusion that hydrolysis of the silyl dienolate occurs in the rate-determining step and is followed by the C–C formation, due to the appropriate orientation of both reagents in the transition state by the catalyst. The reasons for the striking difference in α vs γ regioselectivity of the two reactions compared remain to be clarified.

### 3.2 Additions to C=N Bonds

In this chapter, additions of remotely activated aldehyde-derived carbon nucleophiles to aldimines, ketimines, hydrazides, hydrazones, nitrones, and azomethine imines have been grouped together. All the examples refer to direct procedures involving acyclic pronucleophiles, and the HOMO-raising activation modalities parallel those encountered in the additions to C=O bonds, namely, activation of the remote β- or γ-sites via NHC organocatalysis and activation of remote γ- or ε-sites via enamine organocatalysis, often in cooperation with complementary LUMO-lowering Lewis/Brønsted acid catalysis.

#### 3.2.1 Direct Procedures

##### 3.2.1.1 Acyclic Pronucleophiles

As previously disclosed (Scheme 7), the covalent combination of α,β-unsaturated aldehydes with *N*-heterocyclic carbene (NHC) catalysts generates homoenolate equivalents in which the electron density of the heterocyclic ring is relayed to the vinylogous β-carbon via the diene portion of the molecule. When coupled to C=N bonds, these substrates may give rise to precious γ-lactam products after intramolecular closure according to a formal [3 + 2] annulation. Despite the numerous reports on this chemistry since its introduction in 2005,^90^ the enantioselective versions of these transformations had to wait until 2010 for their debut.^91^

**Scheme 29:**
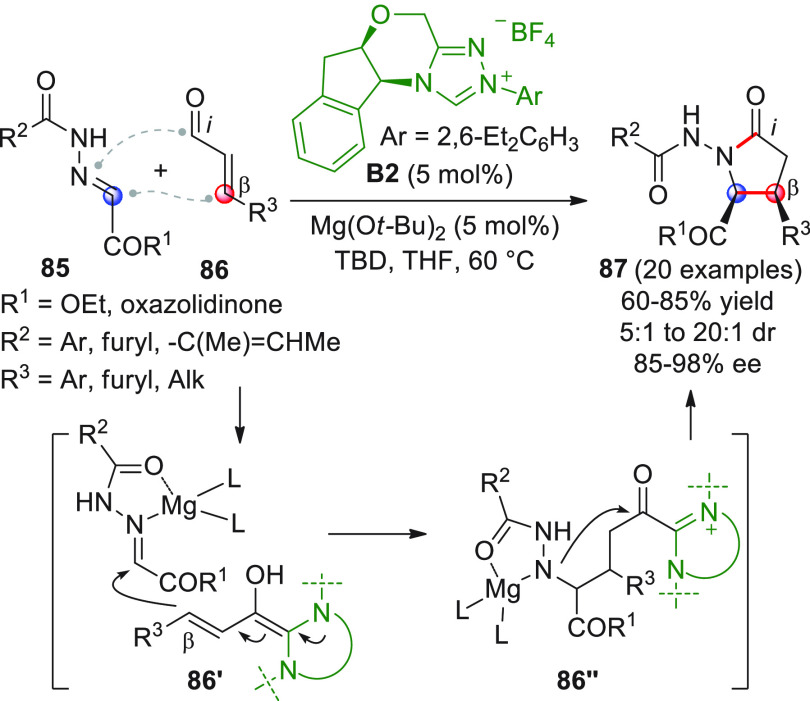


Scheidt and collaborators recognized that the addition of homoenolate equivalents generated by enals **86** to hydrazones **85** would furnish the desired [3 + 2] annulation products with stereochemical induction, if the simultaneous activation of both reaction partners, by distinct catalysts, was at hand (Scheme 29).^92^ Indeed, treating α,β-unsaturated aldehydes **86** bearing aryl, furyl, or alkyl moieties with aryloyl hydrazones **85** in the presence of azolium salt **B2** (5 mol %), a strong base (triazabicyclodecene, TBD), and catalytic magnesium di-*tert*-butoxide (5 mol %) effectively produced *cis*-disposed γ-lactam products **87** in good isolated yields and high enantioselectivities. When varying the imine portion of the substrate, glyoxylate-derived hydrazones and even an oxazolidinone-containing substrate reacted efficiently; whereas hydrazones from aromatic aldehydes (benzaldehyde) were still not electrophilic enough to undergo this annulation even with Mg(II) activation.

The presence of a hard, oxophilic Lewis acid such as the Mg(II) salt proved strategically indispensable for the overall good of the reaction, since it was able to enhance the electrophilicity of the hydrazone substrate, while not disturbing the catalytic activity of the NHC. After some preliminary kinetic studies, the authors proposed a mechanism according to which the vinylogous Breslow intermediate **86′** attacks the Lewis acid-coordinated hydrazone; subsequent intramolecular acylation of the magnesium-bound nitrogen closes the ring with regeneration of both the NHC and Mg(II) catalysts (via **86″**).

**Scheme 30:**
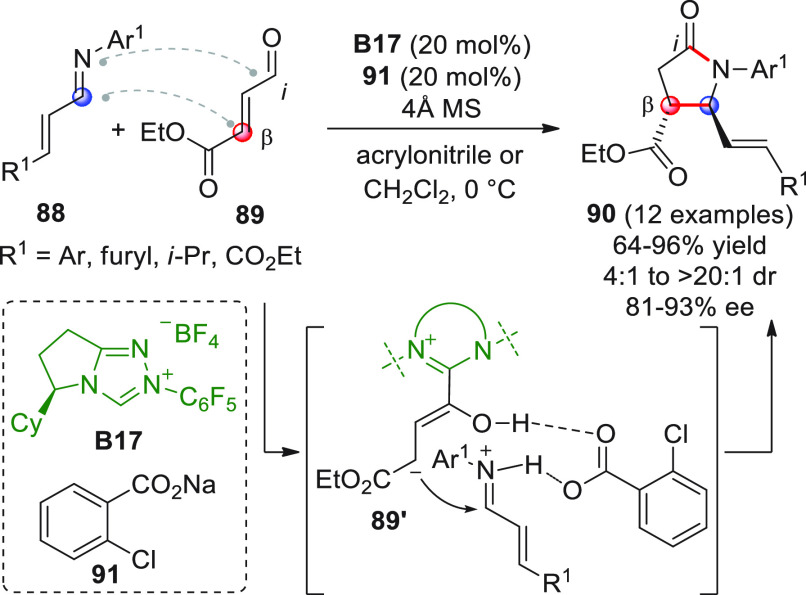


An efficient and enantioselective [3 + 2] annulation between α,β-unsatutated aldehydes and unactivated imines was developed by Rovis and co-workers, using cooperative NHC and Brønsted acid catalysis.^93^ The focal idea was that the conjugate acid of the base, used to generate the carbene species, could be useful in activating basic functionalities such as that of an unactivated imine. The judicious tuning of the electronic and steric nature of the catalysts (i.e., weak basicity of the carbene induced by electron-withdrawing groups, steric hindrance of the carbene, weak basicity of the carbene-forming base) led the researchers to find the right combination of catalysts for an optimal reaction. Thus, β-ethoxycarbonyl-substituted enal **89** was treated with preformed α,β-unsaturated imines **88** in the presence of catalytic, chiral triazolium salt **B17** and sodium *o*-chlorobenzoate **91** (20 mol % each), leading to γ-lactams **90** having an unprecedented *trans*-disposition in the predominant diastereoisomers (Scheme 30).

The best solvents were acrylonitrile or CH_2_Cl_2_, depending on the electronic nature of the Ar^1^ aryl group of aldimines **88**. It was found that under the reaction conditions, other β-substituted enals of type **89** (e.g., β-keto- or β-aryl-substituted) could prove to be competent substrates (not shown), whereas changing the imine component (e.g., *N*-Bn, *N*-Ts unsaturated imines or *N*-phenyl aldimine from *p*-bromobenzaldehyde) gave modest results, if any. Based on control experiments, the authors proposed a mechanism in which the vinylogous Breslow intermediate **89′** attacks the acid-activated imine via hydrogen bonding; subsequent proton transfer would produce an acyl carboxylate eventually affording the annulated targets.

**Scheme 31:**
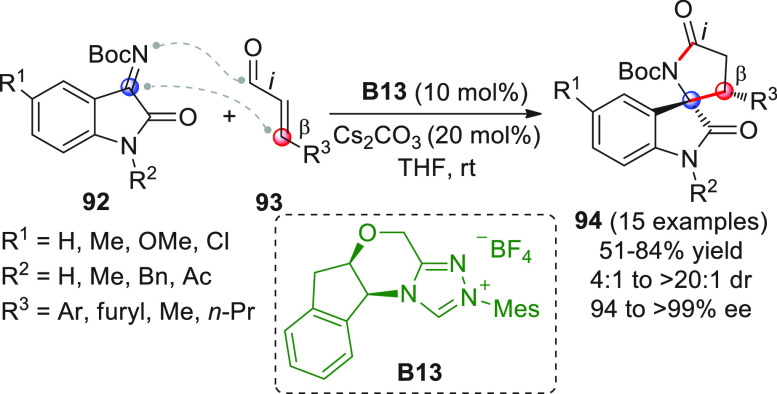


Compared to aldimines, ketimines are generally less reactive, and high stereoinduction is more difficult to achieve. The group of Chi developed an efficient protocol for the addition of NHC-activated enals **93** to isatin-derived ketimines **92**, to afford spirocyclic oxindole-γ-lactams **94** according to the previously disclosed formal [3 + 2] annulation path (Scheme 31).^94^ The aminoindanol-derived triazolium salt **B13** was chosen as the best NHC precatalyst, together with cesium carbonate as the base. A broad range of *N*-protected or even *N*-deprotected isatins **92** can successfully participate in the coupling reaction to diverse β-aryl or β-alkyl enals **93**, giving products **94** in moderate to good yields and very good enantioselectivities.

Almost during the same period, a similar study was reported, dealing with the NHC-catalyzed homoenolate additions of enals with *N*-aryl oxindole-derived ketimines (not shown). The γ-lactam products of type **94** were obtained in good yields in a racemic format, though some preliminary attempts using chiral NHC were performed, obtaining modest enantioinduction.^95,96^

**Scheme 32:**
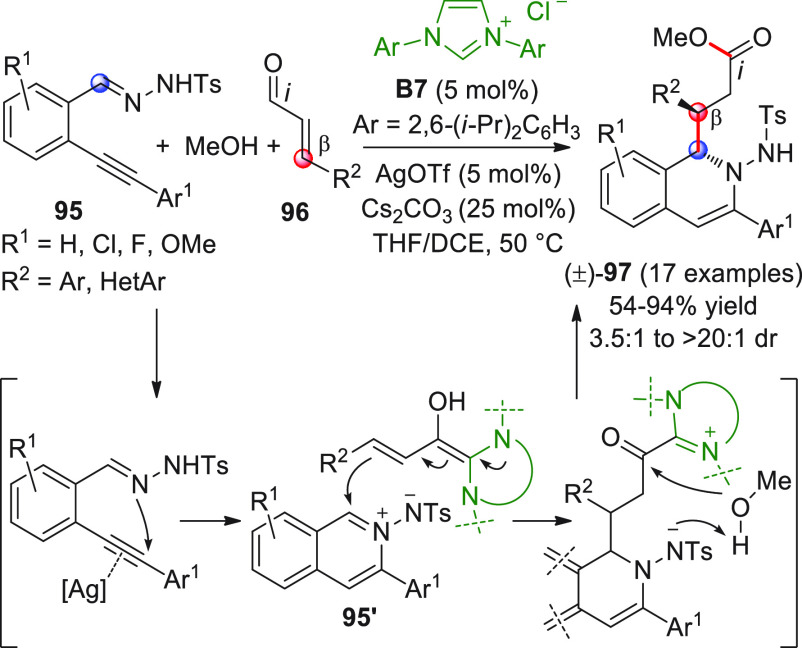


The concept of enabling both nucleophilic and electrophilic activation by combining metal catalysis and organocatalysis was exploited by Wu and co-workers in an inspiring work dating back to 2010.^97^ The researchers discovered that 2-alkynylbenzylidene hydrazides of type **95** could be easily converted to highly electrophilic isoquinolinium ions of type **95′** via 6-*endo*-cyclization in the presence of a suitable metal catalyst (AgOTf); in situ interception of these electrophiles by NHC-activated homoenolates from enals **96** could lead to 2-amino-1,2-dihydroisoquinolines (±)-**97** after the intervention of methanol which liberates the catalyst, as depicted in Scheme 32. After identification of the best reaction conditions (AgOTf/**B7**, 5 mol % each, cesium carbonate as the base), the scope of this one-pot three-component reaction was analyzed, by exploring differently substituted substrates. Aromatic groups attached to the C≡C bond within **95** gave good results, while aliphatic groups (*n*-Bu or cyclopropyl) proved detrimental to the reaction.

**Scheme 33:**
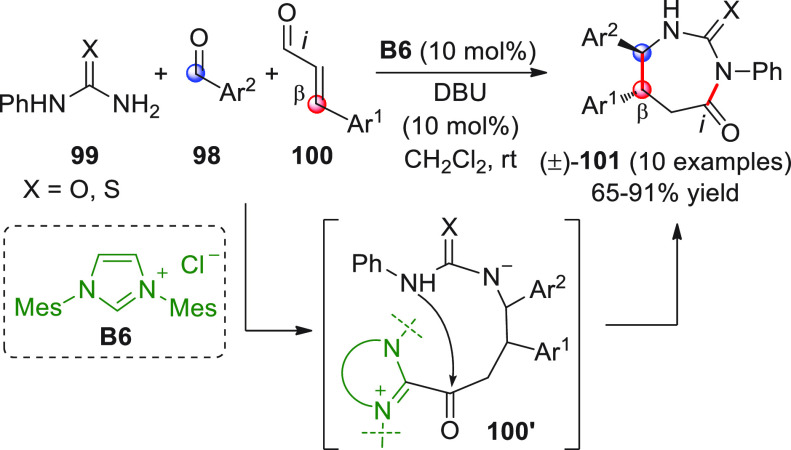


Another one-pot, three-component reaction was proposed by Siddiqui and collaborators during the synthesis of 1,3-diazepanes **101** (Scheme 33).^98^ NHC-activated homoenolates from α,β-unsaturated aldehydes **100** were coupled to aryl imines, which in turn were generated in situ by condensation of aryl aldehydes **98** and urea (or thiourea) derivatives **99**. A formal [4 + 3] annulation took place, likely through intramolecular closure (via **100′**), giving racemic (±)-**101** in good isolated yields.

**Scheme 34:**
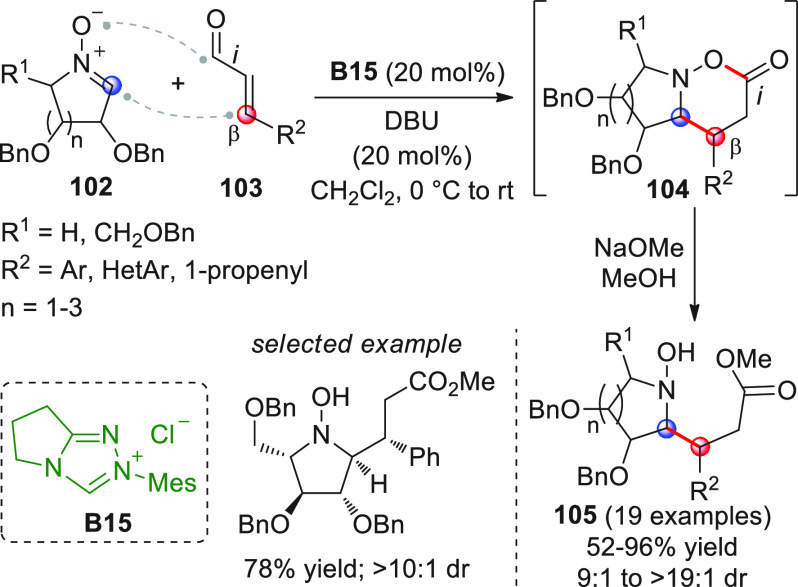


The NHC-homoenolate component unveils a remote β-carbon nucleophilic site which may well act as a “dipolarophile” in a stepwise 1,3-dipolar cycloaddition with suitable dipole components. For example, if a nitrone is used in combination with a β-carbon NHC-activated enal, a formal [3 + 3] annulation may occur. Along this line, sugar-derived cyclic nitrones **102** were found to react cleanly with enals **103** under NHC catalysis, giving polyhydroxylated pyrrolidine and piperidine (and even azepane) derivatives of type **105** (Scheme 34).^99^ In the event, NHC-activated homoenolate from **103** coupled to nitrones **102** according to a formal [3 + 3] annulation, affording intermediates **104** (otherwise stable and storable), which were quenched in situ with NaOMe/MeOH in a one-pot operation, to furnish the desired products **105**. The large collection of products **105** was then easily transformed to the corresponding pyrrolizidine and indolizidine alkaloids (not shown), which were eventually assayed against various glycosidase enzymes to give important clues to the structure–activity relationship of this new compound class.

**Scheme 35:**
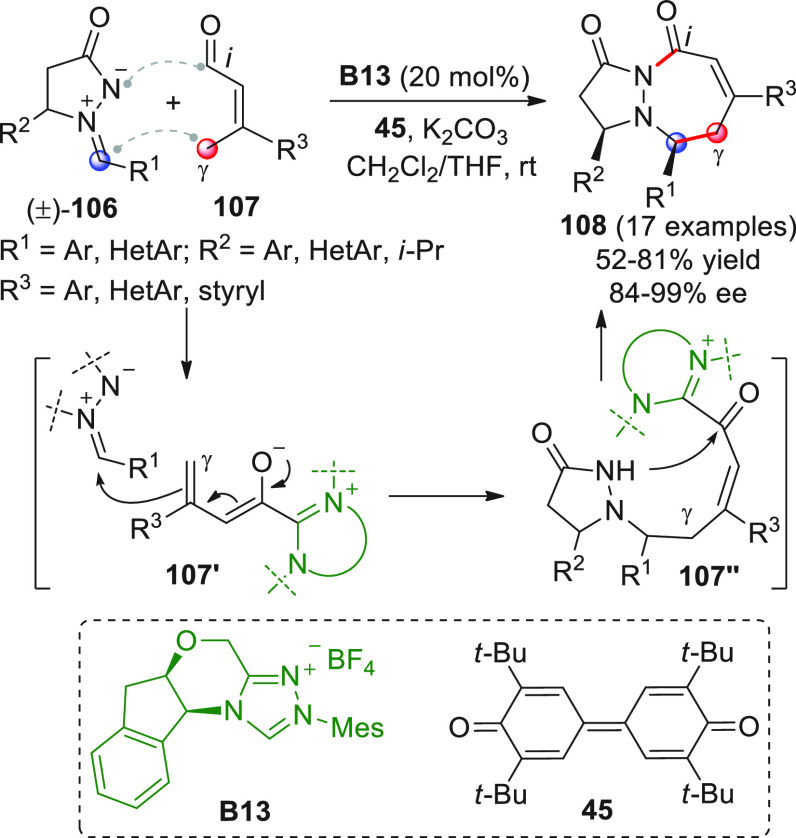


γ-Enolizable enals **107** served as the four-carbon component in the [3 + 4] 1,3-dipolar cycloaddition with azomethine imines **106** (Scheme 35).^100^ Activation of enals **107** at the remote γ-carbon via oxidative NHC-activation (for the general concept of γ-carbon activation via NHC, see Scheme 17) afforded vinylogous enolates **107′** as the reactive dipolarophile components, which reacted with racemic azomethine substrates (±)-**106** to forge the first C–C bond, as depicted in structure **107″**. Intramolecular closure then yielded the expected dinitrogen-fused seven-membered heterocycles **108** with high optical purities and moderate-to-good yields. The approach also provided effective kinetic resolution of the starting racemic azomethine imines.

**Scheme 36:**
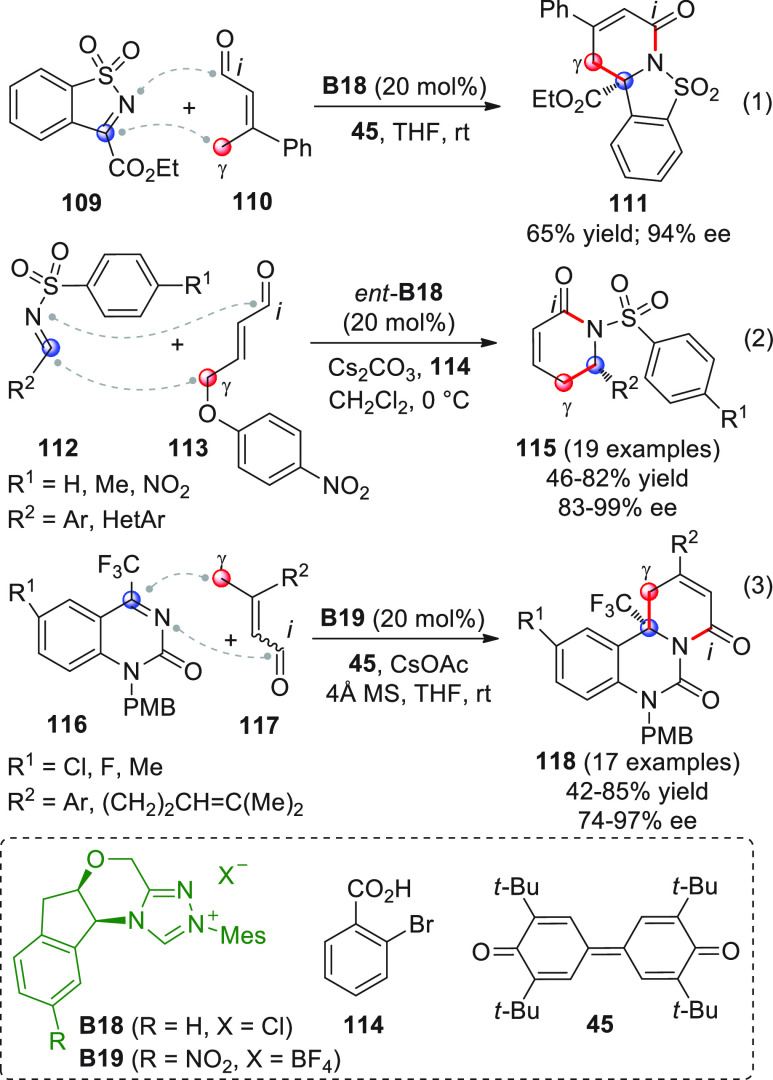


The enantioselective NHC-catalyzed [4 + 2] annulation reaction of γ-enolizable enals and aldimine or ketimine acceptors was developed by diverse authors to obtain highly enantioenriched δ-lactam products. As a first example, Chi and co-workers considered the reaction between enal **110** and cyclic sulfonyl imine **109** in the presence of the chiral NHC precatalyst **B18** and quinone oxidant **45** as the model reaction for studying the general reaction mechanism of these types of annulation (Scheme 36, eq 1).^101^ It was noted that the reaction proceeded effectively, producing the δ-lactam product **111** in a good yield and enantioselectivity, without having to add a base, since the counteranion of the azolium salt behaved as a weak base.

Experimental kinetic studies monitored in situ by ^1^H NMR, as well as deuterium labeling and kinetic isotope effect studies, revealed that the rate-determining step of this oxidative catalysis is the formation of the vinylogous Breslow intermediate between the enal substrate **110** and the catalyst from **B18**, whereas the subsequent steps, namely, oxidation of the Breslow intermediate, γ-carbon deprotonation of the unsaturated azolium ester intermediate, and the vinylogous addition of the γ-carbon to the imine, are facile steps (the depiction of these steps for analogous addition to C=O is given in Scheme 17). The reaction scope of this transformation was then extended to a series of diverse enal/sulfonyl imine pairs (not shown), producing a collection of sulfonyl amides of type **111** which were duly evaluated in vitro for their antibacterial activity.^102^

The cooperative catalysis using NHC from *ent*-**B18** and Brønsted acid **114** (2-bromobenzoic acid) was used for the effective dual activation of γ-enolizable enal **113** and sulfonyl imines **112** for the asymmetric synthesis of δ-lactams **115** (Scheme 36, eq 2).^103^ Instead of using oxidative conditions, enals **113** with an appropriate leaving group (*p*-nitrophenoxy) were utilized to grant the formation of the NHC-bound dienolate intermediates. All the Mannich reactions proceeded uneventfully, giving a diversified array of δ-lactam products in effective yields and good-to-excellent enantiomeric excesses.

The oxidative NHC-catalyzed [4 + 2] annulation reaction involving β-methyl enals **117** and cyclic trifluoromethyl ketimines **116** was developed by Enders and collaborators in 2017 en route to the enantioselective synthesis of dihydroquinazolinone products **118** (Scheme 36, eq 3).^104^ The optimization of the reaction conditions demonstrated that nitro-substituted NHC precatalyst **B19** (20 mol %) together with the cesium acetate base and quinone oxidant **45** were the best choice to furnish the expected products in high yields and stereoselectivities. Of note, besides the β-aryl-substituted enals **117**, aliphatic citral was also tolerated, delivering the target lactam in moderate yield and enantioinduction.

Besides NHC organocatalysis, aminocatalysis offers a powerful activation modality of remotely enolizable polyenals, and dienamine- or polyenamine-mediated additions of these donor substrates to C=N bond acceptors may trigger Mannich-initiated cascade reactions or cycloadditions eventually evolving to annulated products incorporating the γ/β, γ/β/*ipso*-, ε/β-, ... carbon portions of the starting enal (for a depiction of the general concept, see Scheme 2).

**Scheme 37:**
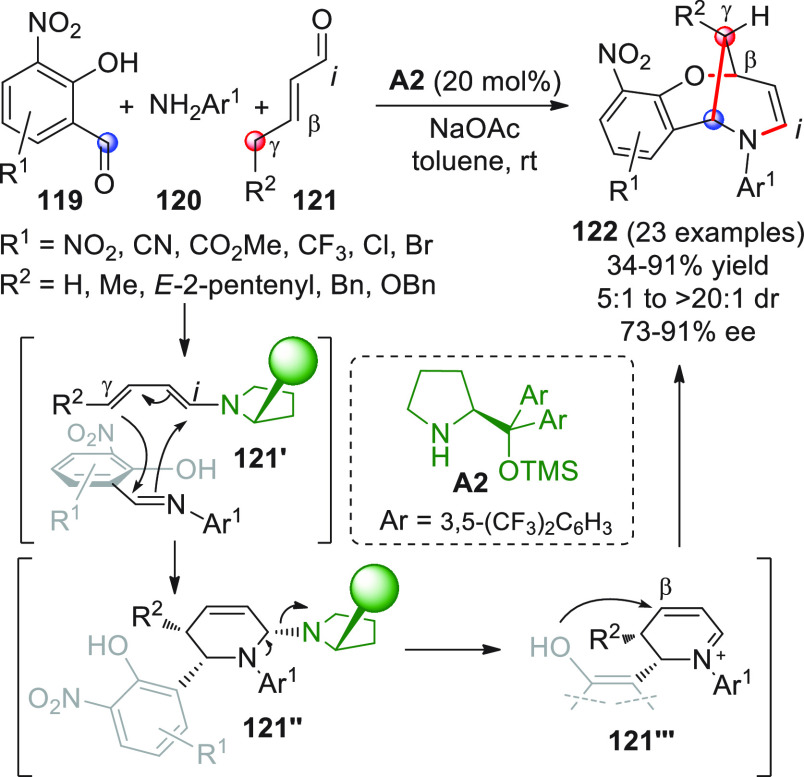


The first aminocatalytic, asymmetric, γ-selective, and Mannich-initiated cascade reaction was reported by Jørgensen and Albrecht in 2014.^105^ γ-Enolizable α,β-unsaturated aldehydes **121**, anilines **120**, and salicylaldehydes **119** were treated together according to a one-pot, three-component procedure in the presence of the Jørgensen–Hayashi organocatalyst **A2** (20 mol %), directly affording bridged benzoxazocines **122** incorporating the γ/β/*ipso* carbon skeleton of the starting enal (Scheme 37).

Tuning the electronic and steric properties by careful choice of the R^1^/Ar^1^ substituents of the salicylaldehydes **119** and anilines **120** was important to increase the rate of their initial condensation to forge an imine in situ, while not being detrimental to the subsequent coupling reaction with **121**. Thus, electron-withdrawing R^1^ groups of **119** and electron-rich anilines **120**, bearing variously substituted phenyl-, naphthyl, and anthracyl groups (including *p*-ethynylphenyl), were revealed to be good substrates for affording the desired products effectively in generally good yields and with high diastereo- and enantioselectivities. A plausible reaction mechanism was proposed according to which an initial condensation between **119** and **120** occurs, giving the corresponding imine in situ. The *s-cis*-dienamine **121′** derived from condensation of enal **121** with the catalyst **A2** and iminium-to-enamine isomerization then couples to the imine component, giving the catalyst-linked [4 + 2] cycloalkene intermediate **121″** and hence the highly reactive iminium ion **121‴** after elimination of the amine catalyst. Finally, an intramolecular oxa-Michael addition within **121‴** consigns the benzoxazocines targets **122**. Neither a truly pericyclic cycloaddition nor an asynchronous—or even stepwise—mechanism was proven by further investigations, though the authors affirmed that employment of electron-rich *N*-aryl anilines might favor a [4 + 2] cycloaddition pathway. Whatever is the case, the vinylogous transmission of the nucleophilic character of the dienamine functionality to the remote γ-site through the π-system is operative, thus accounting for the observed regiocontrol.

**Scheme 38:**
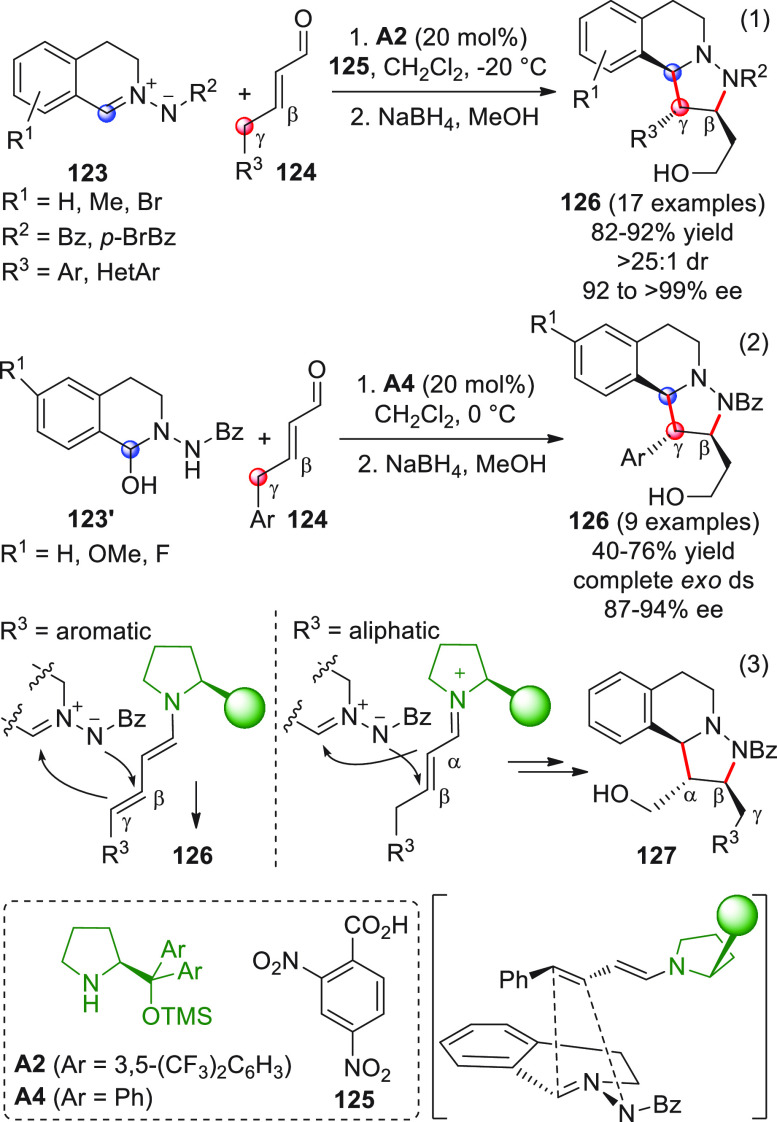


Dienamine-mediated enantioselective [3 + 2] cycloaddition reactions were independently reported in the same year by Du, Wang, et al.^106^ and Alemán, Fraile, et al.,^107^ based on the 1,3-dipolar addition of γ-enolizable α,β-unsaturated aldehydes to *C,N*-cyclic azomethine imines (Scheme 38).

In the first work,^106^ γ-aryl-substituted enals **124** reacted with *N*-aroyl azomethine imines **123** using chiral prolinol silyl ether **A2** (20 mol %) and 2,4-dinitrobenzoic acid (**125**) as an additive (Scheme 38, eq 1). After reduction with NaBH_4_, the corresponding cycloadducts **126** were recovered in high isolated yields as the sole products and with excellent enantiocontrol. The N–N bond of **126** could be easily cleaved with SmI_2_ to give precious tetrahydroisoquinoline products (not shown). The authors affirmed that products **126** were the result of a 1,3-dipolar cycloaddition (whether stepwise or concerted was not specified) involving dienamine-activated γ-carbon of **124** attacking the C=N bond and closure of the terminal nitrogen atom of the azomethine ylide to the enal β-carbon (γ/β carbon sites of the enal substrate involved, Scheme 38, eq 3). Interestingly, aliphatic α,β-unsaturated aldehydes **124** gave completely different results when treated in the same reaction conditions; LUMO-lowered iminium ion activation of **124** occurred in this case which, added to the 1,3-dipole **123** of reverted polarity, gave β/α-functionalized [3 + 2] cycloadducts of type **127**.

The same ambivalent behavior of dienamine/iminium ion activable enals and azomethine imine dipoles was experienced by Alemán and Fraile,^107^ who also annotated some discrepancies between the results of the above cited paper and their own results. Considerable efforts were devoted to finding experimental conditions capable of chemoselectively channelling the reaction paths in either direction, namely, via the dienamine path (inverse electron demand character of the 1,3 dipolar cycloaddition) toward products of type **126** or via the reverted iminium ion-driven path (normal electron demand 1,3-dipolar cycloaddition) toward products of type **127** (Scheme 38, eqs 2 and 3). Thus, the optimized protocol addressing products **126** consisted in treating **123′**, the hydrated hemiaminal form of **123**, with γ-aryl-substituted **124** in the presence of amine catalyst **A4** in CH_2_Cl_2_ at 0 °C; after reduction, targets **126** were obtained with complete *exo*-diastereoselectivity in variable yields and good enantiomeric excesses (Scheme 38, eq 2). A key role in determining the chemoselectivity of the reaction was played by the electronic/steric properties of the amine catalyst, the azomethine imine nature (dipole form vs hydrate form), and the presence or absence of additives (TBAB favoring the iminium ion path). Sustained by in-depth NMR-based and DFT calculations and the body of experimental results, the authors proposed that products **126** were formed as the result of a concerted 1,3-dipolar cycloaddition between in situ dehydrated azomethine imine from **123′** and dienamine-activated **124** thus accounting for the observed diastereo- and enantiocontrol (Scheme 38, bottom). Finally, the researchers found that the products they possessed, resulting from the iminium ion path of type **127**, had the opposite configuration of those reported by Du and Wang.

**Scheme 39:**
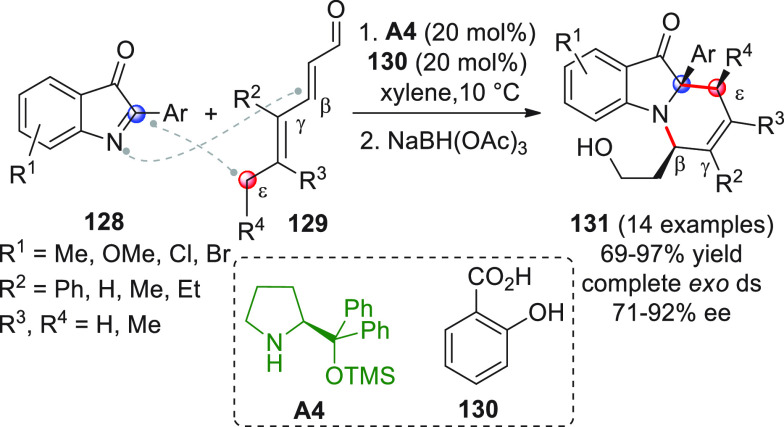


The HOMO activation strategy of ε-enolizable conjugated dienals of type **129** via trienamine organocatalysis was exploited by Chen and collaborators to perform regio- and stereoselective [4 + 2] cycloaddition reactions involving 2-aryl-3*H*-indol-3-ones **128** (Scheme 39).^108^

Using prolinol silyl ether **A4** (20 mol %) and salicylic acid (**130**) as a Brønsted acid cocatalyst, the addition reactions of different 2,4-hexadienals (and one 2,4-heptadienal) **129** to indolone derivatives **128** went to completion affording, after reduction, highly functionalized tricyclic products **131** efficiently and with good stereoselectivities. The good performance of the reaction was also due to the ad hoc-placed R^2^-R^4^ substituents of the dienal starters **129**; almost all successful examples concerned γ-phenyl-substituted hexadienals (R^2^ = Ph), while the use of unsubstituted 2,4-hexadienal or 2,4-heptadienal gave aza-Baylis–Hillman-type products (not shown).^109^ The authors referred to the overall reaction as a normal-electron-demand aza-Diels–Alder reaction, and they did not enter into details about either the concerted or Mannich-initiated stepwise nature of the mechanism.

### 3.3 Conjugate Additions to Electron-Poor C=C Bonds

Paralleling the functionalization chemistry of remote carbon sites of unsaturated aldehydes in addition reactions to C=O and C=N bonds, even in the case of additions to electron-poor alkenes, the majority of examples are associated with direct procedures (no indirect examples were indeed found in the literature over the period covered) and mainly involve the organocatalytic activation of pronucleophilic substrates via either (poly)enamine or NHC catalysis. Most of the documented studies give rise to cycloaddition products emerging from Michael type-initiated additions to the electron poor olefins, followed by intramolecular closure as testified by diverse computational and/or experimental evidence.

#### 3.3.1 Direct Procedures

##### 3.3.1.1 Acyclic Pronucleophiles

α,β-Unsaturated aldehydes may explicit their β-pronucleophilicity via NHC activation to form the corresponding vinylogous Breslow intermediates (see previous sections) which may react with electron-poor alkenes via conjugate addition (vinylogous β-donor on vinylogous β-acceptor). The fate of the resulting coupling—i.e. product type and stereochemical result—depends mainly upon the functional groups of the acceptor component and the catalyst/cocatalyst/additive used.

**Scheme 40:**
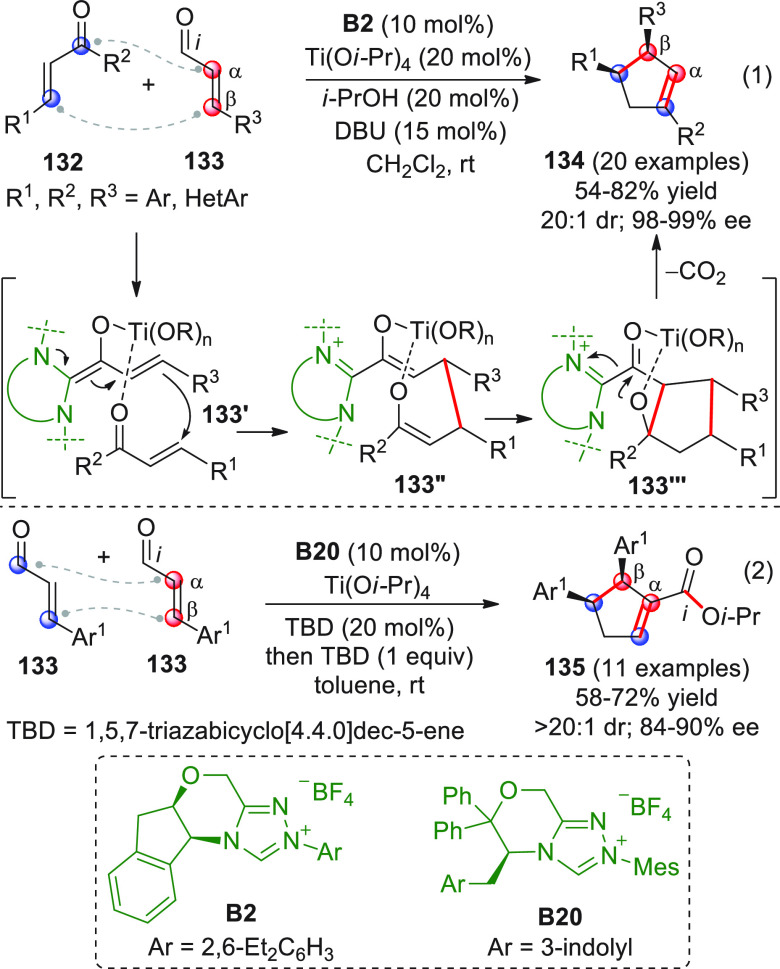


Following the fundamental work by Nair^110^ and Bode,^111^ Scheidt and collaborators discovered how β-aryl enals **133** could productively react with a series of chalchones **132**, giving *cis*-configured 1,3,4-trisubstituted cyclopentenes **134** (Scheme 40, eq 1).^92^ Key to the success of the transformation was the integrated use of NHC catalysis (chiral aminoindanol-derived triazolium salt **B2** was the best precatalyst) and Lewis acid catalysis (Ti(O*i*Pr)_4_ bearing the donating metal alkoxide ligands, proved to be the best choice) exploiting the concept of simultaneous activation of the two reacting partners via cooperative catalysis. Using the NHC/LA cocktail, together with catalytic *i*-PrOH as an addititve and DBU as the base, ensured preparation of the targeted cyclopentenes **134** in high isolated yields, excellent *cis*-diastereoselectivity, and optimal enantioselectivity. Carrying out the reaction without the Lewis acid led to the formation of the *trans-*isomers as the major products, pointing to the key role exerted by the Lewis acid during the stereodetermining step. A catalytic pathway was proposed, where the titanium-coordinated homoenolate **133′** adds via conjugate addition to the titanium-coordinated chalcone acceptor, generating the *cis*-disposed species. Protonation/tautomerization of the chalcone moiety then predisposes the second C–C bond-forming event, namely, the intramolecular aldol addition involving the α-carbon of the starting enal and the chalcone carbonyl. The cyclopentane ring **133‴** is thus forged, which quickly converts to the cyclopentene target after intramolecular acylation, with NHC release and decarboxylation (the *ipso* carbon is lost as CO_2_). A global [3 + 2] annulation occurs, involving the β/α carbon donor sites of the starting enal.^112,113^

Merging the Lewis acid activation strategy with NHC Lewis base catalysis was soon after exploited by the same research group in the enantioselective dimerization of enals of type **133** to give cyclopentene esters **135** (Scheme 40, eq 2).^114^ In this case, the challenge was to channel the reaction path toward a [3 + 2] annulation involving 1,4-conjugate addition of the β-donor homoenolate (β to β), while bypassing the competing and often prevailing 1,2-addition path (β to *ipso*). It was demonstrated that the presence of the Ti(O*i*Pr)_4_ cocatalyst was essential in driving the reaction toward the desired cyclopentenes. The reaction pathway proposed is very similar to the previous one (via **133′** and **133″**); in this case, however, the isopropoxide ligand promotes the final acylation with liberation of the NHC catalyst, while the excess of TBD base promotes water elimination to provide the alkene moiety.

**Scheme 41:**
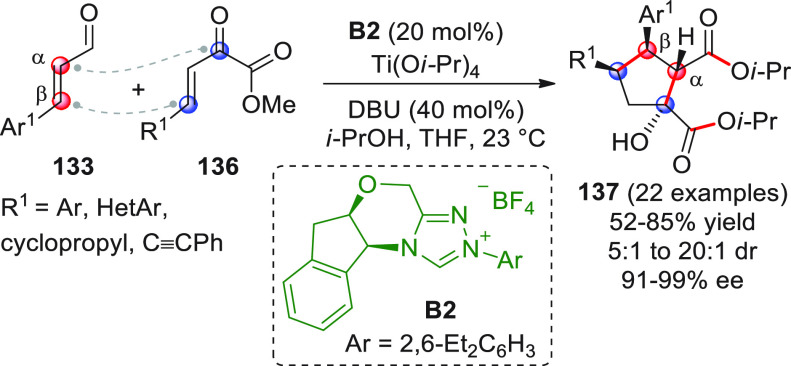


Besides chalchones and enals, another class of electron-poor alkenes could be conveniently added to NHC-bound homoenolates under cooperative Lewis acid/NHC catalysis, namely, β,γ-unsaturated α-ketoesters.^115^ As depicted in Scheme 41, several keto esters **136** were analyzed, bearing aryl, heteroaryl, cyclopropyl, and alkynyl γ-substituents, all giving the corresponding cyclopentane bis-esters **137** with good results (only alkyl and alkenyl R^1^ groups did not work, data not shown). Again, a Michael-type addition of vinylogous homoenolate from **133** to keto esters **136** occurs, followed by intramolecular aldol addition of the Cα site within **133** to the ketone acceptor (see a similar mechanism in Scheme 40, via intermediates **133′**–**133‴**). In this case, however, intermolecular acylation and transesterification by isopropanol/isoproxide ligand occurs, liberating the NHC catalyst. No intramolecular acylation nor water elimination to cyclopentene products was observed.

A highly regio- and stereoselective addition of NHC-activated enals with benzodienone acceptors was reported by Chi and co-workers, highlighting a Michael–Michael-type cascade annulation, and furnishing multifunctionalized polycyclic products (not shown).^116^

Similarly, β-NHC-activated 2-aroylvinylcinnamaldehydes were reacted with 2-aroylvinylchalchones, providing access to complex indane products (as racemates), via triple Michael-type addition and lactonization cascade.^117^ Some years later, analogous 2-aroylvinylcinnamaldehydes were added to α,β-unsaturated sulfonyl imines under asymmetric NHC catalysis (homoenolate chemistry) giving rise to controlled regio- and stereodivergent pathways depending upon the employed catalysts (not shown).^118^

**Scheme 42:**
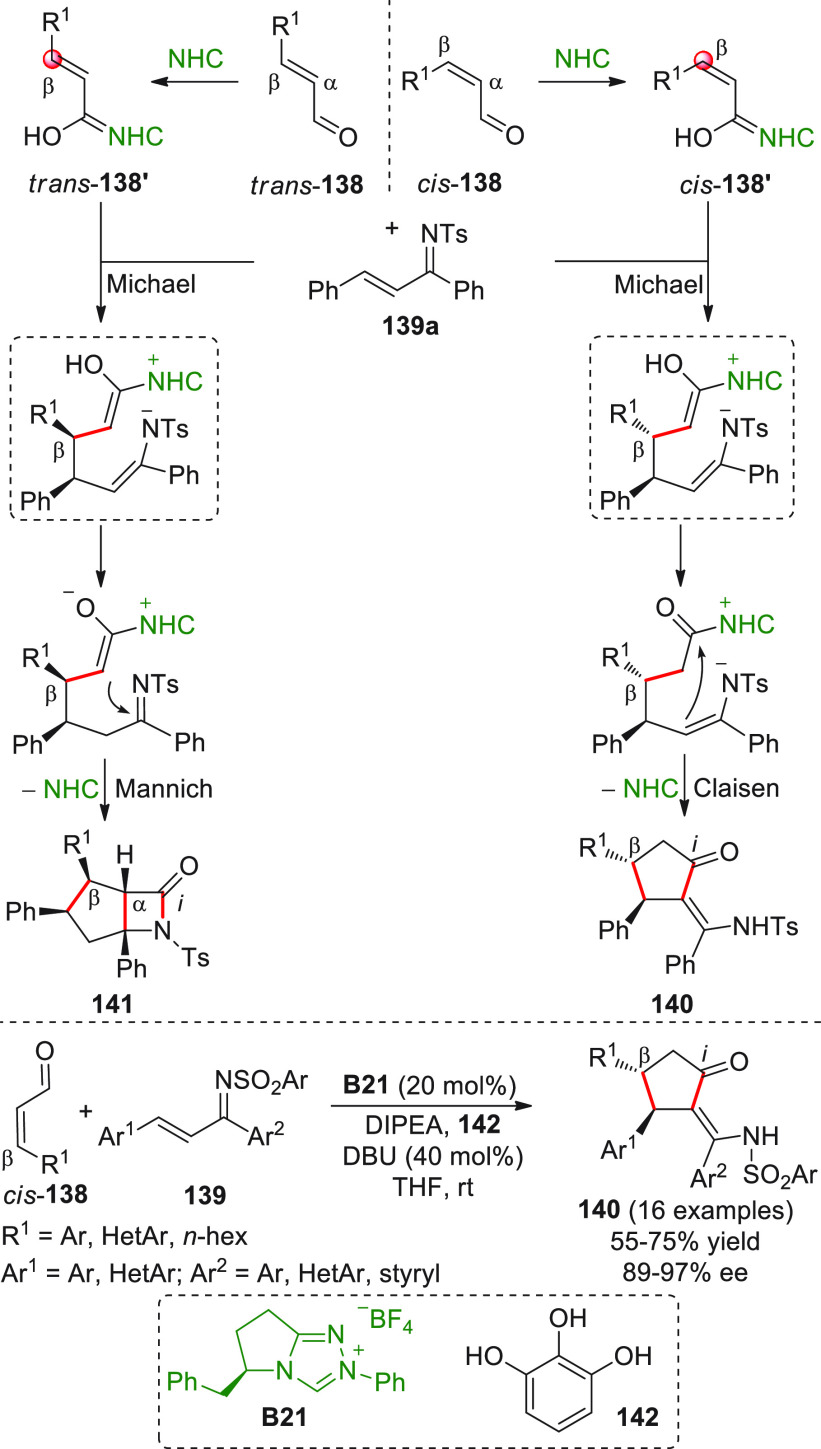


The reaction of *cis*-configured enals of type *cis*-**138** activated by NHC catalyst with α,β-unsaturated imines was thoroughly investigated by Chi et al. in 2013 (Scheme 42).^119^ It was known from previous studies by Bode,^120^ that *trans*-enals react with these electron-deficient alkenes under NHC catalysis giving bicyclic β-lactams of type **141** (Scheme 42, left side), presumably as the result of a first Michael-type addition of the β-site of the corresponding *trans-*homoenolate (*trans*-**138′**) to the conjugated sulfonyl imine and subsequent intramolecular Mannich-type closure and *N*-acylation.^120^ Accordingly, the β/α/*ipso* carbon atoms of the starting enal are inserted within the lactam bicycle.

Quite unexpectedly, in treating *cis*-**138** under optimized conditions (precatalyst **B21**, DIPEA base and triphenol additive **142**) aimed at preserving its *cis*-stereointegrity, a completely diverse reaction path was unveiled, and coupling with unsaturated sulfonyl imines **139** led to alkylidene cyclopentenone products **140** in high yields, complete diastereoselectivity, and with negligible, if any, presence of the bicyclic lactam counterparts **141**. In this case, it was proposed and partially proved by control experiments that *cis*-homoenolate *cis*-**138′** was formed, which underwent a first Michael-type addition followed by intramolecular Claisen reaction (Scheme 42, right side). This work represents a nice example of how two diastereomeric intermediates (dashed boxes in the scheme) trigger divergent reaction pathways toward different target chemotypes.^121^

In 2013, Scheidt and McCusker developed a new NHC-catalyzed formal [4 + 2] annulation reaction between enals and α,β-unsaturated imidazolidinone acceptors. In this case, however, a nonvinylogous path was triggered, where the α-position of the unsaturated enal acted as a donor site instead of the vinylogous homoenolate-linked β-position (not shown).^122^

**Scheme 43:**
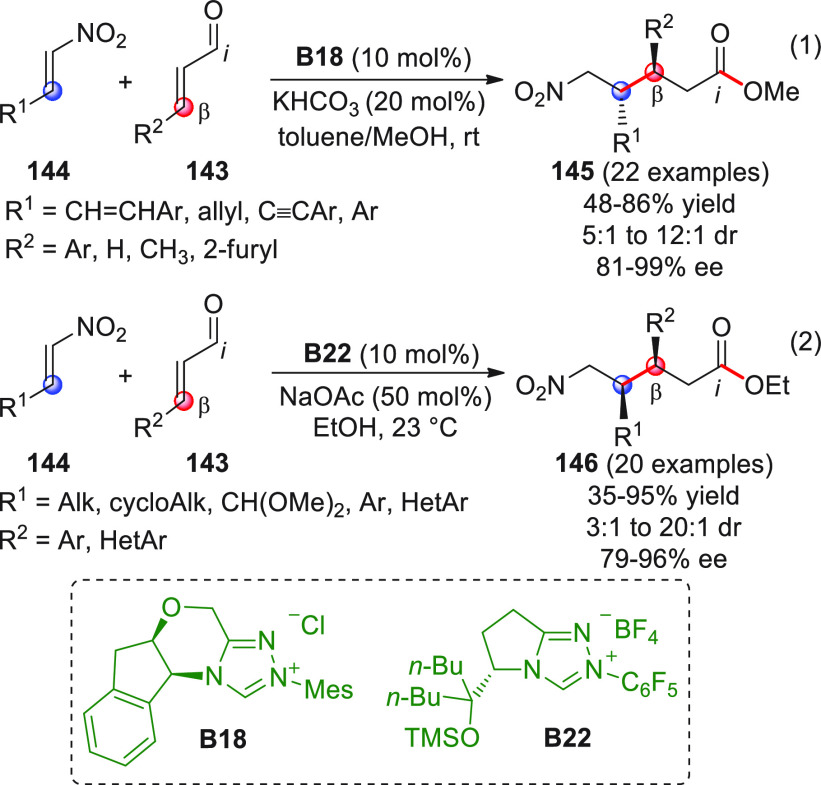


Among electron-poor alkenes, nitroalkenes were also usefully used in Michael-type addition reactions with α,β-unsaturated aldehydes via NHC-activated homoenolates, to furnish linear δ-nitro esters (Scheme 43).

Following a pioneering report by Nair who initially applied this chemistry in a racemic format,^123^ Liu and collaborators succeeded in performing this transformation in an asymmetric context.^124^ Thus, as shown in Scheme 43 (eq 1), a large series of nitroalkenes **144**, including nitrodienes, nitroenynes, and nitrostyrenes, efficiently reacted with enals **143** (mainly aromatic, but also acrolein, crotonaldehyde, and furyl-substituted enals were reported), using NHC precatalyst **B18** and furnishing, after acylation by external methanol, the corresponding nitromethyl esters **145** in generally good yields, *anti*-diastereoselectivity, and moderate to high enantiocontrol. Interestingly, the reactions proved completely regioselective (β-carbon donor on β-carbon acceptor), and no 1,6-conjugate additions (β-donor on δ-acceptor) or Stetter coupling (*ipso* donor on β-acceptor) was witnessed. As a limitation, aliphatic nitroalkenes did not prove to be suitable substrates in this transformation.

Complementary to this work were the studies by Rovis et al., who found conditions to perform the same type of transformation to deliver *syn*-configured nitro esters, while extending the scope to aliphatic nitroalkenes (Scheme 43, eq 2).^125^ Key to the success of the reaction was the rational design of the NHC-precatalyst to be used, whose stereoelectronic properties were carefully tuned in order to bestow a positive impact on the regio- and stereocontrol of the overall reaction. Thus, using bis-*n*-butyl O-TMS triazolium precatalyst **B22** in combination with sodium acetate and ethanol, aryl-substituted enals **143** were added to a large number of nitroalkenes **144** giving *syn*-disposed nitroethyl esters **146** (Scheme 43, eq 2). Enantio- and diastereoselectivities were typically greater for alkyl- and cycloalkyl nitroalkenes, though bulky derivatives such as *t*-Bu did not work (not shown). Interestingly, alkyl-substituted enals **143** were not productive and gave mainly the undesired Stetter products (not shown). In both studies,^124,125^ the nitro ester products were easily manipulated into valuable nitrogen-containing cyclic products.

α,β-Unsaturated esters were also competent electron-poor substrates to be added to β-NHC-activated enals. For example, Nair and collaborators developed a NHC-catalyzed process featuring the intramolecular Michael-initiated cascade reaction involving 2-*O*-alkenoate cinnamaldehydes and culminating in the formation of racemic coumarin derivatives (not shown).^126^

**Scheme 44:**
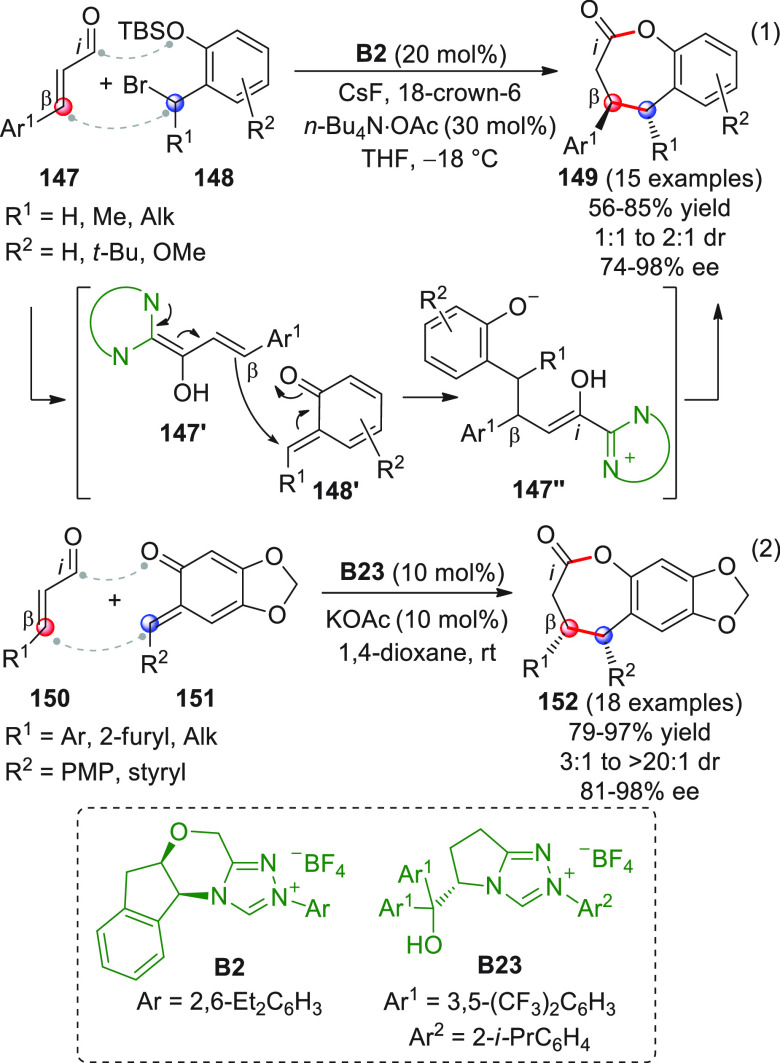


In 2013, Scheidt et al. devised a dual activation strategy where two reactive, transient species were concomitantly generated: a NHC-bound vinylogous β-donor of type **147′** and *o*-quinone methide (*o*QM) acceptor **148′** (Scheme 44, eq 1), derived from the respective precursors, enals **147** and silyl phenols **148**.^127^ The optimal reagent combination (CsF/18-crown-6 for *o*QM generation and *n*-Bu_4_NOAc as a mild base for NHC generation from precatalyst **B2**) provided formal [4 + 3] benzoxopinone products **149** in moderate/good yields and with acceptable to excellent enantioselectivities. The scope of the reaction was explored: various cinnamaldehydes **147** were well tolerated, while highly reactive acrolein gave a totally diverse [4 + 2] product (not shown). As for *o*QM precursors, both bromides and chlorides could be used as good leaving groups, and in general R^2^ electron-donating groups gave the best results. Also, prostereogenic silyl phenols precursors were exploited (R^1^ ≠ H), extending the [4 + 3] process to vicinally substituted products. In these cases, however, low diastereoselectivities were observed. Based on DFT calculations (and other data), it was proposed that the vinylogous Breslow intermediate **147′** would intercept the transient *o*QM **148′** through a Michael-type C–C-bond-forming addition in an open transition state. The formed species **147″** would then undergo tautomerization and intramolecular *O*-acylation by the phenoxide anion, thereby producing the lactone product with concomitant release of the NHC catalyst. Overall, the driving force to induce the formation of a 7-membered ring was the rearomatization of the *o*QM species. The β-vinylogous regioselectivity strictly depended upon the stability of the extended Breslow intermediate **147′**; in the absence of β-aryl substitution (e.g., acrolein, crotonaldehyde), protonation of the β-site becomes competitive and nonvinylogous [4 + 2] annulation occurs via NHC-enolate C-α conjugate addition/*O*-acylation (not shown).

Similar chemistry was developed by Ye and collaborators over the same period.^128^ In this instance, the fairly stable and electron-rich PMP- or styryl-substituted *o*QM species **151** were directly used as electrophilic substrates without the necessity of generating them in situ (Scheme 44, eq 2). These species were reacted with enals **150**, which were activated in situ by the NHC catalyst derived from carbinol **B23** and potassium acetate base. The corresponding [4 + 3] annulated products **152** were obtained, as emerging from the vinylogous conjugate addition of the β-carbon donor **150** to the *o*QM **151**, followed by intramolecular acylation (see mechanism in the same scheme, eq 1). The authors affirmed that the bulkiness of the NHC catalyst, together with its hydrogen-bonding properties, covered a role in governing the vinylogous regioselectivity and the enantiocontrol as well. Interestingly, not only β-aryl and β-heteroaryl enals but also β-alkyl enals of various chain length (R^1^ = ethyl, *n*-propyl to *n*-heptyl) could be efficiently used to give, in these last cases, remarkable diastereoselectivities and enantioselectivities.

**Scheme 45:**
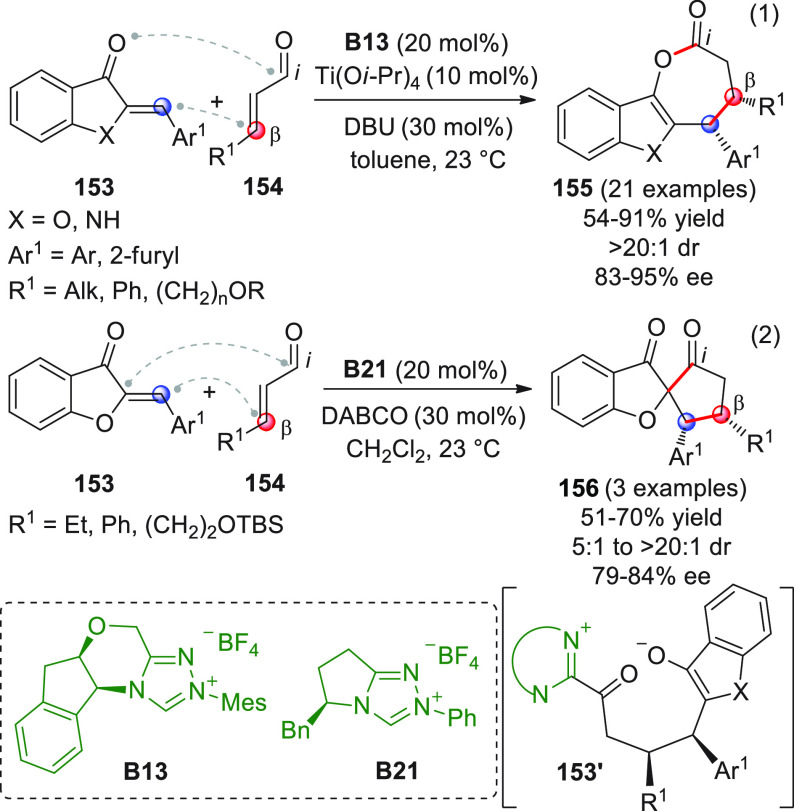


A catalyst-controlled chemodivergent reaction between α,β-unsaturated aldehydes **154** and cyclic enones **153** was developed by Zhao et al., leading to the formation of either [4 + 3] lactone products **155** (Scheme 45, eq 1) or spirocycles **156** (eq 2).^129^ In the first instance, cooperative catalysis was adopted, using NHC precatalyst **B13**/DBU and Ti(O*i*-Pr)_4_ as a Lewis acid. The substrate scope of this catalytic system was broad and included enals **154** bearing alkyl, ether-containing alkyl, and phenyl groups, while the Michael acceptor component **153** included both oxygen and nitrogen heterocycles.

In almost all cases, the corresponding coumaranone or indole-fused derivatives **155** (X = O, NH) were obtained with excellent *cis* diastereoselectivity and good chemoselectivity (**155**:**156** = 5:1 to 15:1). When the precatalyst **B21**/DABCO combination was used instead, the reaction between **153** (X = O) and **154** gave [2 + 3] annulation to furnish mainly spirocycles **156** (**156**:**155** = 4:1 to 7:1). A stepwise mechanism was proposed, according to which both targets are generated by a common intermediate precursor **153′** (the product of the conjugate addition of NHC-bound homoenolate to the Michael acceptor), which closes either to the [4 + 3] product **155** by *O*-acylation or to the [2 + 3] product **156** by *C*-acylation. The authors speculated that the backbone of the azolium catalyst played a dramatic effect on this chemodivergent path and postponed a more rigorous mechanistic discussion to future work.

Similar chemistry was cleverly developed by Glorius and co-workers, where Michael acceptors of type **153** were coupled to enals under NHC catalysis, leading to the exclusive formation of [2 + 3] products with high optical purity (not shown).^130^

In line with the chemistry disclosed in the previous sections, the direct HOMO-raising remote activation of α,β-unsaturated aldehydes by NHC catalysis may involve not only the β-site (vinylogous with respect to the *ipso* carbon) but also the γ-site (vinylogous with respect to the α-carbon), opening new synthesis perspectives toward precious carbocyclic and heterocyclic targets.

**Scheme 46:**
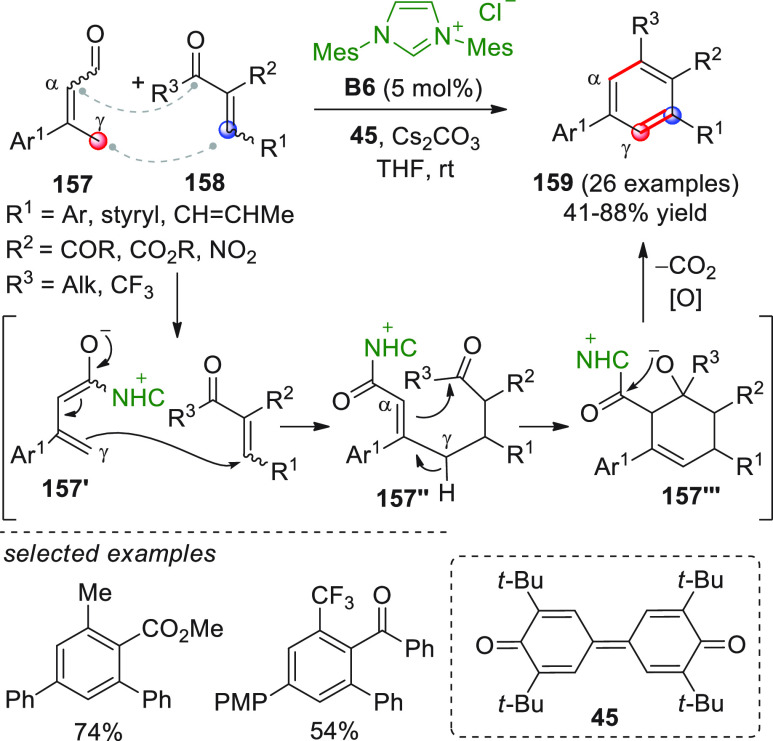


As a clever example of this concept, Chi et al. devised a very simple and efficient NHC-catalyzed formal [3 + 3] cycloaddition reaction to address multifunctionalized benzenes in one step by starting from α,β-unsaturated enals and unsaturated ketones.^131^ As illustrated in Scheme 46, a very easy procedure was implemented, according to which γ-enolizable β-aryl (or even β-heteroaryl) enals **157** reacted with a wide collection of enones **158** using achiral NHC precatalyst **B6** under oxidative conditions, directly giving benzenes **159** in useful isolated yields. The variability of the R^1^-R^3^ substituents within enones **158** granted access to a wide collection of aromatic targets ranging from alkyl, aryl, and trifluoroalkyl ketones, to esters and nitro derivatives.

Though no in depth studies on the reaction mechanism were executed, based on precedents of NHC-γ-activation (see Scheme 17), a plausible mechanistic path of this formal [3 + 3] cycloaddition was proposed. This would encompass a Michael addition step involving vinyl enolates **157′**, in turn obtained from oxidation/γ-deprotonation of the extended Breslow intermediate, and enones **158**, to furnish intermediate **157″**. Subsequent γ-deprotonation of **157″** and intramolecular α-aldol addition would lead to **157‴** which collapses to aromatic products **159** by *O*-acylation with NHC release, decarboxylation, and final oxidative aromatization. This straightforward and versatile procedure greatly outperformed the previously reported traditional syntheses of these targets, which justifies the legitimate collocation of this work in the context of the vinylogous realm, notwithstanding the fact that achiral products are obtained.

γ-Enolizable enals could also be suitable substrates for formal [4 + 2] cycloaddition reactions with suitable Michael acceptors. Along this line, Yao et al. used γ-methyl α-bromo-α,β-unsaturated aldehydes and 3-alkylideneoxindoles to perform the diastereoselective synthesis of spirocarbocyclic oxindoles under NHC catalysis (not shown).^132^

**Scheme 47:**
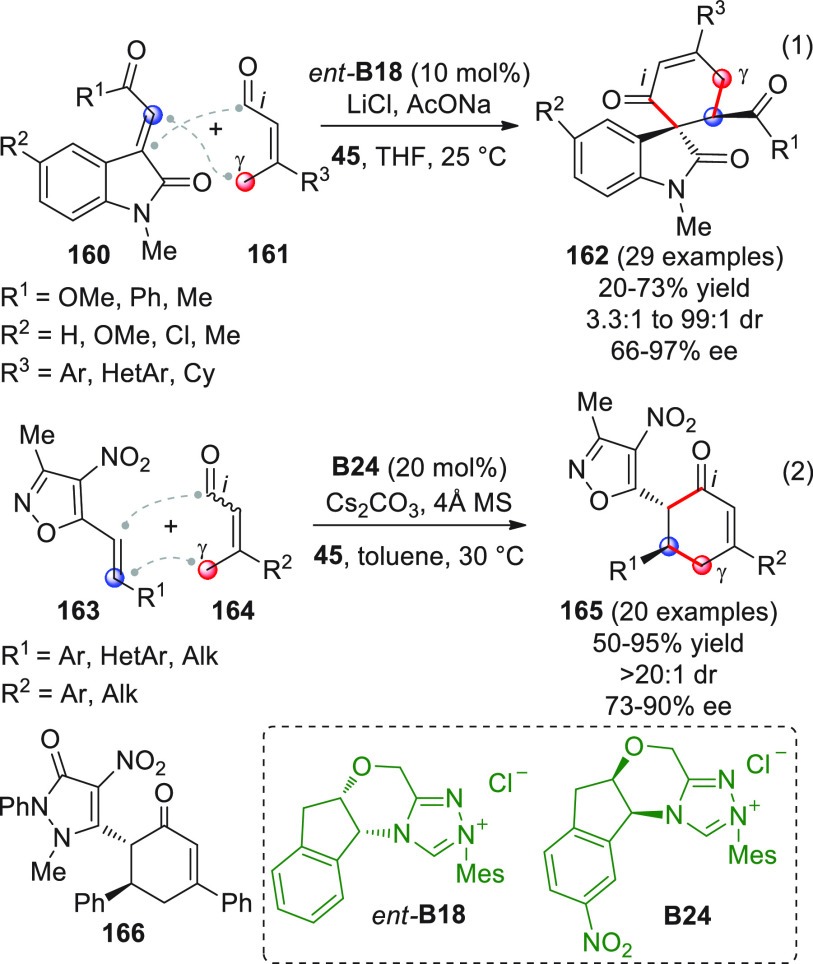


One year later, Xu, Liu, et al. documented a similar transformation in an enantioselective context (Scheme 47, eq 1).^133^ In this case, β-methyl enals **161** were reacted with oxindoles **160** using NHC catalyst from *ent*-**B18** and LiCl as a useful Lewis acid additive. With the exception of heteroaryl and cyclohexyl derivatives **161**, which gave products **162** in low yields and enantiomeric excesses, the other enal/enone substrates performed quite well, affording the corresponding all-carbon spirocyclic oxindoles **162** with good results. A plausible mechanism of this reaction entails a stepwise process: an initial γ-regioselective Michael addition of NHC-bound dienolate from **161** to **160**, followed by intramolecular closure of the emerging enolate to the C-*ipso* carbon (*C*-acylation) with the liberation of the NHC catalyst.

3-Methyl-4-nitro-5-vinyl isoxazoles **163** also served as interesting Michael acceptors in formal [4 + 2] cycloadditions with enals **164** under NHC catalysis (Scheme 47, eq 2).^134^ A varied collection of cyclohexenone products **165** was assorted in generally good yields, excellent diastereoselectivities, and moderate to good enantioselectivities. The proposed mechanism is similar to the previously disclosed examples; in this specific case, the NHC-activated γ-dienolate from **164** is engaged in a 1,6-conjugate addition to the electron-poor nitrodiene **163** (vinylogous γ-donor on bis-vinylogous δ-acceptor), and the emerging γ-nitronate attacks, again in a vinylogous sense, the NHC-bound C-*ipso* site to consign the cycloadducts **165**. The same procedure was also extended to other electron-deficient alkenes, namely, 2,4-diene from antipyrine and 2,4-dienes from malononitrile, benzoylacetonitrile, and Meldrum’s acid, affording the respective products (e.g., compound **166**).

**Scheme 48:**
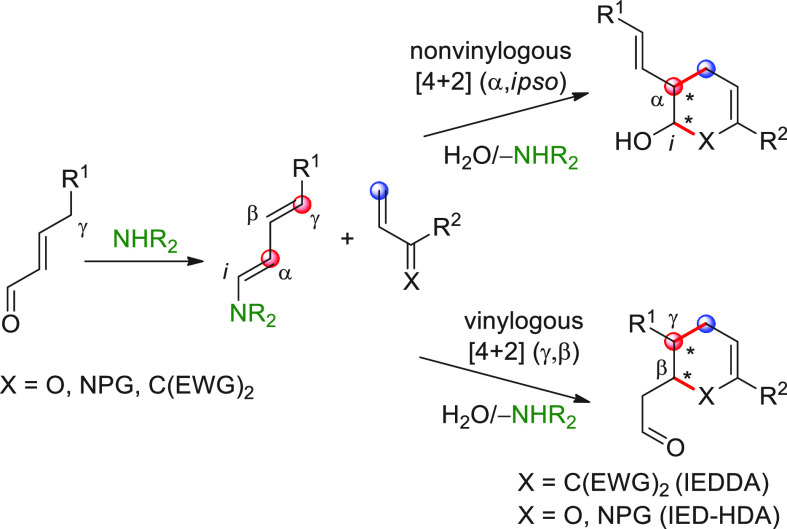


The HOMO-raised dienamine species derived from an amine catalyst and γ-enolizable α,β-unsaturated aldehydes may act as good 2π dienophile species in various [4 + 2] cyclization reactions with electron-deficient diene partners in what could be (at least formally) categorized as inverse-electron-demand Diels–Alder (IEDDA) or hetero Diels–Alder (IED-HAD) reactions (Scheme 48).

Since 2010, various strategies were proposed by different research groups, to regioselectively steer such cyclizations either toward nonvinylogous processes involving α,*ipso* carbon sites of the initial enal,^135^ or toward vinylogous processes, involving the remote γ,β carbon atoms.

**Scheme 49:**
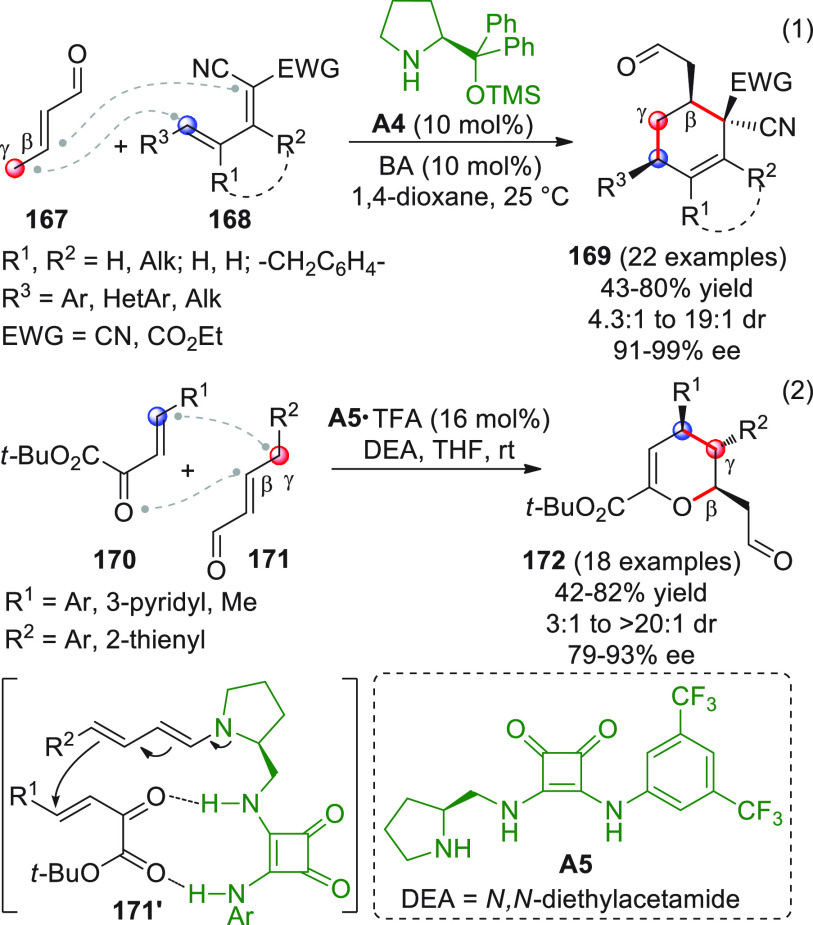


A first attempt in this latter direction was made by Chen and co-workers, who chose allylidene malononitriles or cyanoacetates **168** as 4π-component, and crotonaldehyde **167** as 2π-component in their aminocatalytic all-carbon IEDDA-type reactions (Scheme 49, eq 1).^136^ The combination of catalytic secondary amine **A4** and benzoic acid additive ensured formation of the desired cyclohexene products **169** in moderate-to-high yields and generally good stereoselectivities. In the absence of direct evidence about the actual reaction mechanism yet considering the very good enantiocontrol at the remote γ,β positions, the authors speculated that this reaction proceeded via a concerted [4 + 2] cycloaddition pathway. An *endo*-selectivity emerging from the attack of the *Si* face of the *s-cis*-dienamine intermediate from **167** to the diene **168** under steric-shielding catalyst control seemed to be responsible for the observed stereoinduction within the products. Nevertheless, as stated by the authors themselves, more investigations to elucidate the actual mechanism remained to be carried out.

In order to expand the scope of such IEDDA reactions beyond the simple crotonaldehyde substrate, the same group developed an asymmetric all-carbon IEDDA-type cycloaddition involving γ,β-functionalization of γ-enolizable β,β-disubstituted enals and chromone-fused dienes via dienamine organocatalysis. Multifunctional caged or fused heterocyclic products were obtained in high optical purity and with high efficiency as the result of a sequence encompassing an IEDDA reaction, followed by deprotonation-isomerization and vinylogous aldol closure (not shown).^137^

A remotely γ,β-regioselective and stereoselective IED oxa-DA reaction was reported by the Jørgensen group, by applying H-bond directing aminocatalysis.^138^ As shown in Scheme 49 (eq 2), various unsaturated oxoesters **170** were selected as electron-deficient components, anticipating their possible activation in H-bonding aminocatalysis. These substrates were reacted with γ-enolizable enals **171** in the presence of catalytic secondary amine-squaramide dual catalyst **A5** (16 mol %) and *N,N*-diethylacetamide (DEA), affording the desired [4 + 2] cycloadducts **172**. The generality of the reaction with respect to both reacting substrates was assayed, demonstrating that dihydropyran derivatives **172** could be assembled in various assortments with generally good efficiency and moderate-to-good stereocontrol. The rationalization accounting for the stereochemical outcome was provided. Although no calculations were performed, yet in accordance with earlier calculations in related [2 + 2] cycloadditions, a stepwise mechanism was invoked (i.e., a vinylogous Michael-initiated addition followed by *O*-closure) involving *s-trans* dienamine **171′** and H-bonding activated keto esters **170**, with possible beneficial π-stacking interactions between the aromatic moieties of the reacting partners.^139^

By employing the same bifunctional squaramide-amine catalyst, an asymmetric IED oxa-DA was developed by the same authors, who employed γ-enolizable α,β-unsaturated aldehydes and α,β-unsaturated acyl phosphonates as starting substrates. Useful dihydropyran rings were obtained in good yields and optical purity (not shown).^140^

**Scheme 50:**
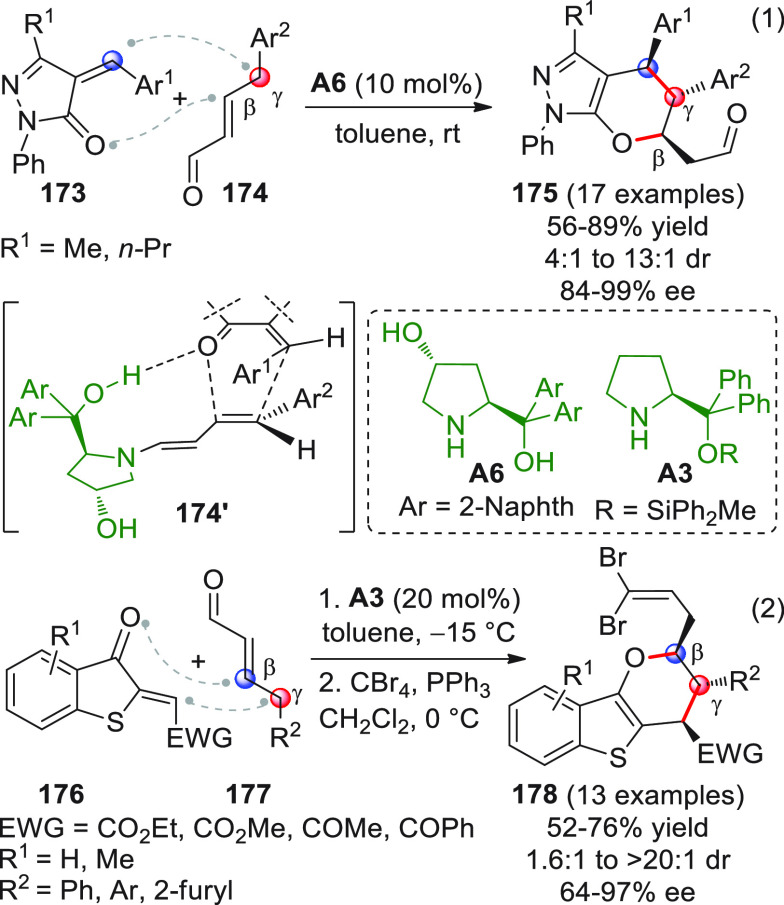


Inspired by these IED-HDA reactions using H-bond-directing dienamine-mediated strategies, Pericàs and collaborators developed [4 + 2] cycloadditions between γ-enolizable enals **174** and alkylidene pyrazolones **173** (Scheme 50, eq 1).^141^

The corresponding chiral tetrahydropyranopyrazoles **175** were produced in good yields, with high enantioselectivity and satisfying levels of diastereoselectivity. Based on previous DFT calculations, an *E,s-trans*,*E*-dienamine **174′** was postulated to be the active conformer, which approaches the heterodiene component along an *exo*-trajectory with an H-bonding between the OH of the diarylprolinol and the pyrazolone carbonyl.

A novel approach to optically active benzothiophene-dihydropyran ring-fused compounds **178** was devised by Albrecht et al., based on the γ,β-regioselective IED oxa-DA reaction between 2-alkylidenebenzothiophenones **176** and γ-enolizable enals **177** (Scheme 50, eq 2).^142^ The reaction proceeded under aminocatalytic conditions using protected prolinol **A3** (20 mol %) with the participation of a key dienamine intermediate derived by condensation/isomerization between the starting enals and the amine catalyst. The driving force of the process was judged to be the aromatization of the thiophene moiety within the targets, which were isolated as dibromoalkene derivatives after olefination of the crude cycloadducts (CBr_4_, PPh_3_).

**Scheme 51:**
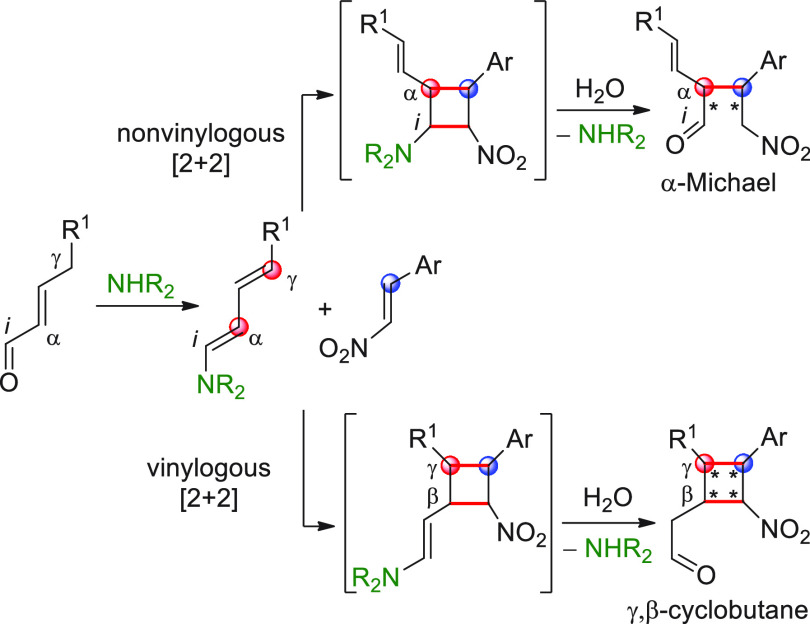


Besides [4 + 2] cyclizations, HOMO-raised dienamine species derived from γ-enolizable enals and amine catalysts may be conveniently engaged in [2 + 2] cycloaddition reactions involving electron-poor alkene partners such as nitroalkenes (Scheme 51). While the nonvinylogous α-functionalization leads to linear Michael-type products,^143^ the vinylogous γ-functionalization of such dienamines may lead to stable cyclobutane products (formal [2 + 2] cycloaddition), provided that suitable activating catalysts and product-stabilizing tricks are invented. In 2012, two research groups independently and almost simultaneously addressed this issue with success.

**Scheme 52:**
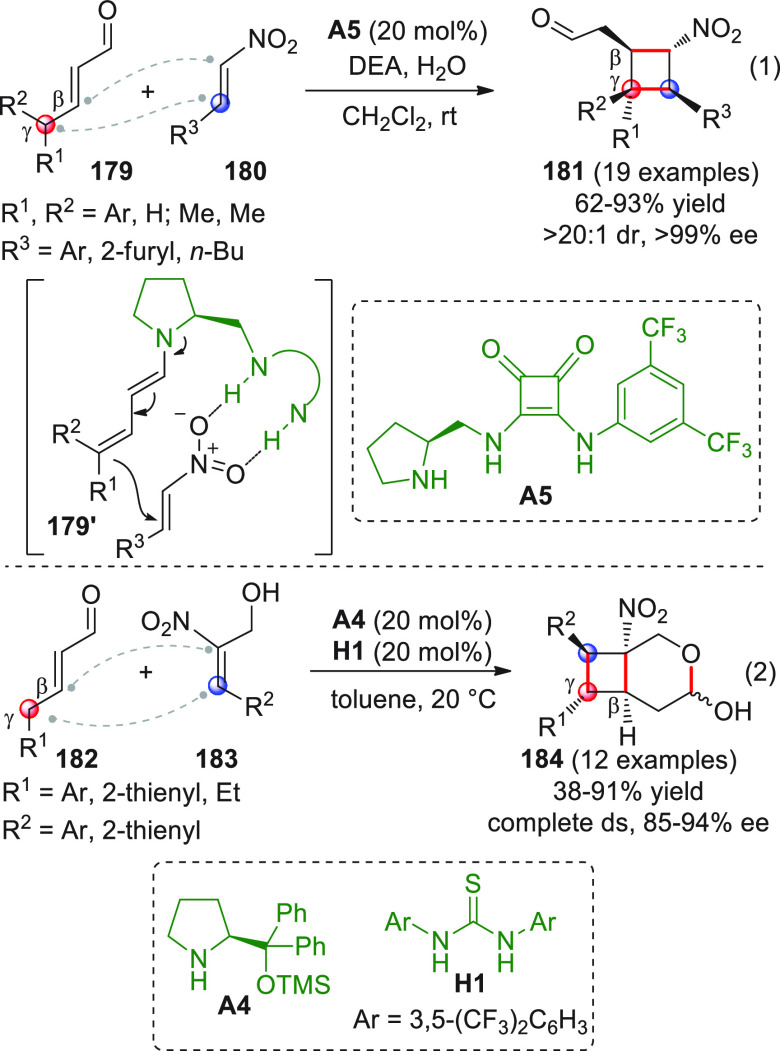


The Jørgensen group purposely designed a bifunctional organocatalyst which would be able to both efficiently activate the two reacting partners and provide an appropriate distance between them in the transition state in order to steer the reaction toward the intended, remote vinylogous functionalization (Scheme 52, eq 1).^144^ Thus, treating enals **179** and nitroalkenes **180** with H-bond directing squaramide-amine catalyst **A5** (20 mol %) in the presence of *N,N*-diethylacetamide (DEA) as catalyst-solubilizing agent and controlled quantities of water (2.8 equiv) in CH_2_Cl_2_ cleanly provided cyclobutanes **181** in good yields and exceptional levels of diastereo- and enantioselectivities. A variety of nitrostyrenes (R^3^ = Ar) and even heteroaryl or alkyl-substituted nitroalkenes were used, which were coupled with diverse enals **179** with equal success. Computational studies supported a stepwise mechanism for this [2 + 2] cycloaddition: at first, a vinylogous Michael-type addition occurs between the γ-carbon of the dienamine-activated enal **179′** and the β-carbon of the squaramide-activated nitroolefin. The nitronate intermediate, thus formed, then intramolecularly attacks the vinylogous iminium ion to furnish the cyclobutane product.

To perform similar [2 + 2] cyclizations, Vicario and collaborators started from enolizable enals **182** and hydroxymethyl nitrostyrenes **183**, and used chiral secondary amine **A4**/achiral thiourea **H1** as an efficient catalytic couple (Scheme 52, eq 2).^145^ Dual activation of both reagents occurred (covalent activation of **182** via dienamine and hydrogen bonding activation of the nitro group by the thiourea) triggering a Michael/Michael/hemiacetalization reaction cascade to the final bicyclic products **184**. Even in this case, complete diastereoselectivity was witnessed and the products were isolated in high yields (except for the case of alkyl derivatives, R^1^ = Et) and high optical purity as 1:1 mixture of anomers. Of note, the reaction of **182** with nitrostyrene under the reaction conditions failed, pointing to the conclusion that the hydroxymethyl appendage within **183** was indispensable to steer the reaction toward hemiacetal derivatives **184**, thereby providing a thermodynamic driving force for the reaction to proceed to completion.

On the basis of similar H-bond-directing dienamine activations, the asymmetric synthesis of spirocyclobutyl oxindoles was carried out by Wang et al. via formal [2 + 2] cycloaddition between enolizable enal donors and alkylidene oxindole acceptors (not shown).^146^

**Scheme 53:**
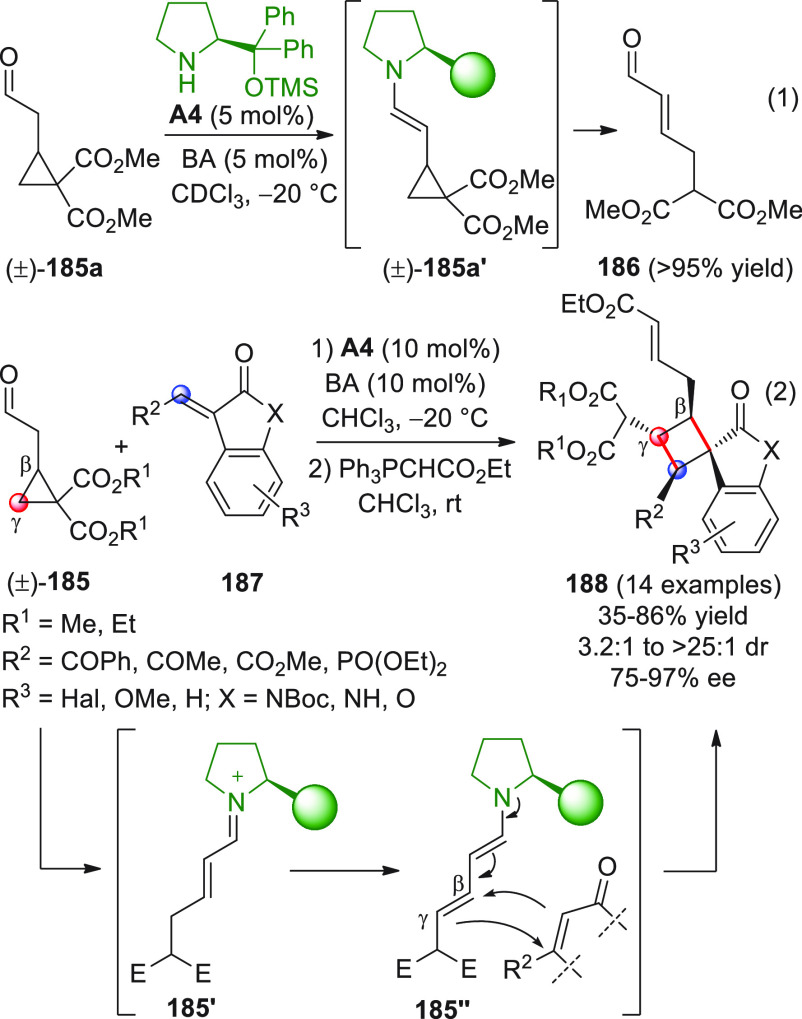


In 2015, Jørgensen and co-workers discovered a conceptually novel organocatalytic enamine-activation mode of cyclopropanes and exploited this concept in stereoselective cycloaddition reactions (Scheme 53).^147^

Based on preliminary robust computational studies, they first demonstrated that treating racemic diester-substituted cyclopropylacetaldehyde (±)-**185a** with prolinol silyl ether catalyst **A4** and benzoic acid promoted smooth ring opening to α,β-unsaturated aldehyde **186** via HOMO-activated enamine **185a′** (eq 1). Hence, they proceeded to investigate whether it was possible to intercept the organocatalytically activated cyclopropane intermediates of type **185a′** in reactions with electron-deficient alkenes. Indeed, it turned out to be the case. Cyclopropanes (±)-**177** were reacted with olefinic oxindoles (or benzofurans) **187** in the presence of amine catalyst **A4** (10 mol %) and benzoic acid affording (after one-pot Wittig-type olefination) spirocyclobutane oxindoles (or benzofuranones) **188** with good results in terms of scope generality, efficiency, and stereoselectivity. Two mechanistic pathways were proposed, namely, a [3 + 2] cycloaddition followed by ring-contracting rearrangement (unlikely) or a dienamine-mediated formal [2 + 2] cycloaddition (preferred). According to this last proposal, catalyst-induced ring opening of the cyclopropane within **185** produces iminium ion **185′** and hence dienamine **185″**, which undergoes γ,β-regioselective cyclization with the electron-poor substrate **187**. In practice, the amine-activated cyclopropylacetaldehyde substrates acted as useful dienamine surrogates to be used, as in these cases, in vinylogous processes.

HOMO-raised dienamine species obtained by condensation/isomerization of enolizable enals with amine organocatalysts could be cleverly used as 4π components in [4 + 2] cycloaddition reactions, either stepwise or concerted, with electron poor 2π alkenes^148,149^ to give precious six-membered rings. Inherent challenges in this strategy include possible double participation of the amine catalyst in activating both substrates (e.g., dienamine and iminium ion), difficulty in efficient catalyst release and recycling, and efficient catalyst-to-substrate stereoinduction. Several reports dealing with this subject were chronicled in the 2010–2018 period, which are grouped and briefly commented on in the following schemes.

**Scheme 54:**
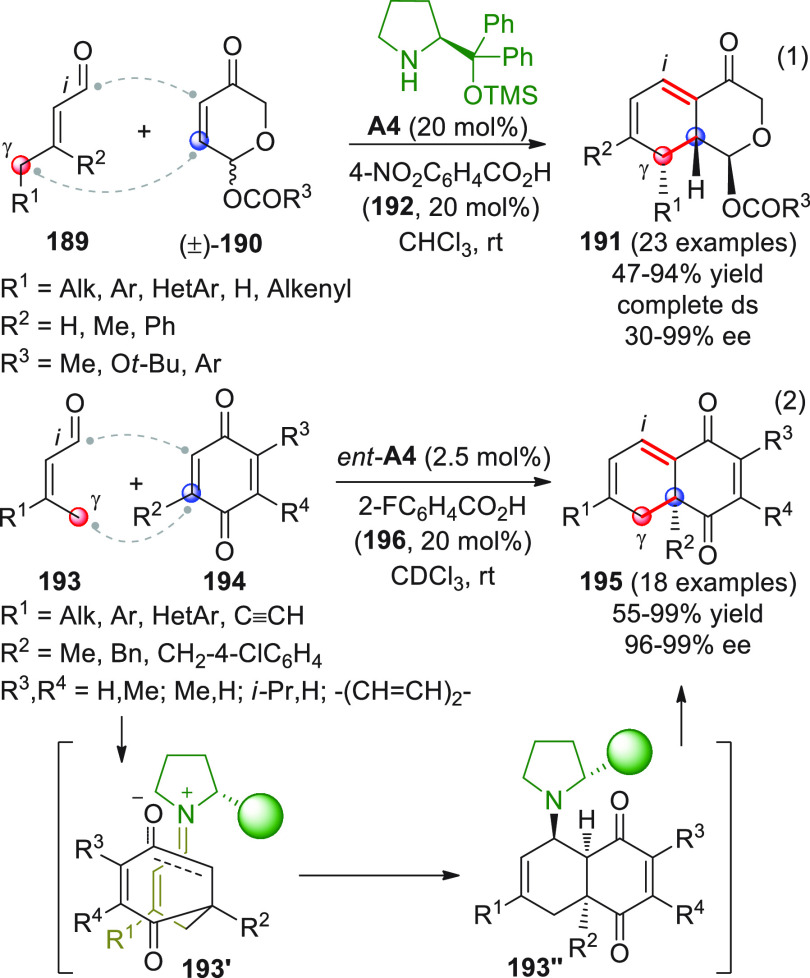


In a first example, Vicario et al. exploited the potential of dienamine catalysis (using amine catalyst **A4** and 4-nitrobenzoic acid cocatalyst **192**) to promote the coupling reaction between enolizable enals **189** and racemic acyloxy-substituted dihydropyranones (±)-**190** (Scheme 54, eq 1).^150^ Enantioenriched isochromanes **191** were obtained regioselectively as single diastereoisomers in moderate-to-good yields as the result of [4 + 2]/elimination cascade reaction in a dynamic kinetic resolution process. No mention was made of the concerted vs stepwise nature of the cyclization.

In another study by the Jørgensen group, dienamine-activated enals **193** added to 1,4-benzo- or 1,4-naphthoquinones **194**, affording dihydronaphtho- and dihydroanthraquinones **195** (Scheme 54, eq 2).^151^ Excellent levels of enantioinduction and complete regioselectivity (γ-carbon donor attacking the more hindered quinone carbon acceptor) were uniformely observed. Computational studies supported the notion that a stepwise mechanism was involved; an initial vinylogous Michael-type addition of the dienamine along an *endo* pathway, to forge the zwitterionic enolate/iminium ion intermediate of type **193′**, which is internally stabilized by favorable electrostatic interactions, after which intramolecular aldol closure to **193″** and catalyst elimination consign the targeted compounds.

**Scheme 55:**
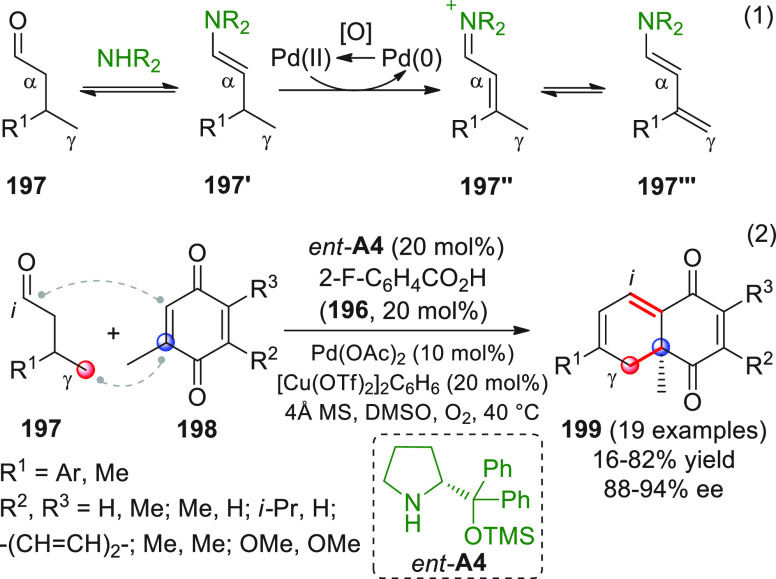


Several years later, a closely related [4 + 2] cycloaddition reaction was performed by Gong and co-workers, by adopting a completely different C–H activation strategy.^152^ The basic idea was just as simple as it was powerful: treatment of unbiased saturated aldehydes **197** (Scheme 55, eq 1) with a chiral amine catalyst would easily produce enamine **197′**, which could be converted to unsaturated iminium ion **197″** by Pd-catalyzed Saegusa-type oxidation. Clean iminium ion-enamine isomerization of γ-enolizable **197″** would then produce the active dienamine species **197‴**, which is able to participate in a plethora of asymmetric vinylogous (or nonvinylogous) coupling reactions with suitable electrophiles. A mixed metal/organo cooperative catalysis would thus enable the enantioselective functionalization of inactive C(sp^3^)–H bonds at the γ-position of saturated aldehydes by directly transforming them in situ into the corresponding HOMO-raised dienamine species. The feasibility of this concept was proven by treating different saturated β-substituted aldehydes **197** with methylquinones (or methylnaphthoquinones) **198** in the presence of catalytic Pd(OAc)_2_, chiral amine *ent*-**A4**, and acid additive **196** in DMSO under oxygen atmosphere (to ensure reoxidation of Pd(0) for the next catalytic cycle) at 40 °C (Scheme 55, eq 2). Further addition of a Lewis acid such as benzene complex of copper(II) triflate was found to be beneficial for the overall efficiency of the reaction, especially when less reactive methylnaphthoquinones substrates were involved. The expected [4 + 2] cycloadducts **199** were obtained with good optical purity in variable yields, mainly depending on the electronic properties of the R^1^-R^3^ substituents within the substrates.

Yang et al. documented an intramolecular version of dienamine-based eliminative [4 + 2] cycloadditions, by starting from bis-enal substrates to forge highly enantioenriched dihydrobenzofurans (not shown).^153^

**Scheme 56:**
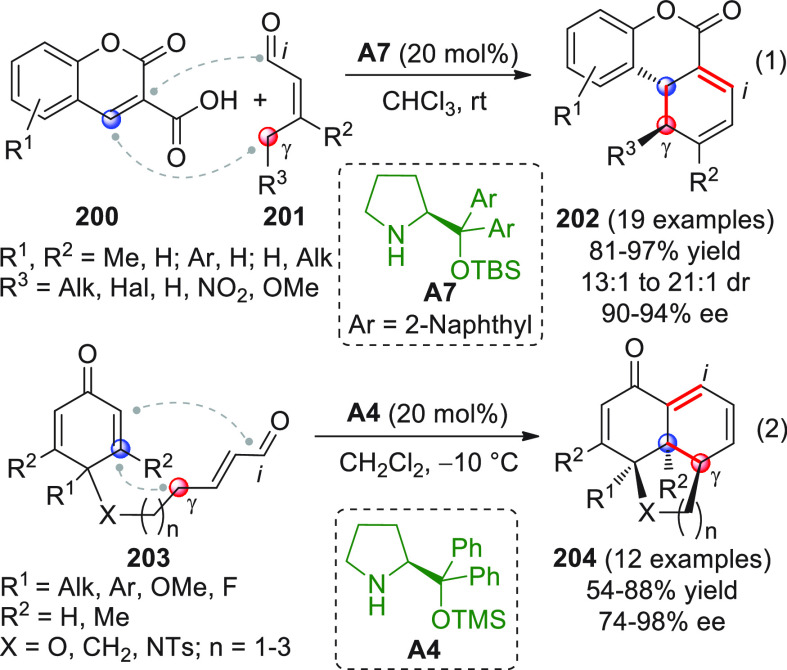


In another study, coumarin-3-carboxylic acids **200** and enolizable enals **201** were involved in a catalytic decarboxylative and eliminative [4 + 2] cycloaddition reaction to forge cyclohexadiene lactones **202** (Scheme 56, eq 1).^154^ In this case, in situ decarboxylation enabled release of the amine catalyst, allowing the transformation to proceed in a high yield and with high enantio- and diastereoselectivity. Subsequent one-pot hydride reduction of **202** and acid-catalyzed intramolecular cyclization provided access to chiral bridged tricyclic benzopyrans (not shown).

A similar aminocatalytic, decarboxylative, and eliminative [4 + 2] cycloaddition reaction was devised by Albrecht and Bojanowski for the synthesis of enantioenriched dihydroxanthones (not shown).^155^ In this instance, β,β-disubstituted γ-enolizable enals and chromone-3-carboxylic acids were used as substrates, while methyl-protected (*S*)-diphenylprolinol was employed as the amine organocatalyst.

The asymmetric synthesis of a collection of tricyclic derivatives **204** was developed by Alemán and collaborators via an aminocatalyzed desymmetrization reaction involving ad hoc prepared cyclohexadienone/enals **203** (Scheme 56, eq 2).^156^ DFT-based calculations and control experiments showed that this transformation proceeds via an asynchronous eliminative [4 + 2] cycloaddition (*endo* transition state) and not a stepwise Michael/aldol/elimination cascade reaction.

An asymmetric dienamine-mediated eliminative [4 + 2] cycloaddition between sulfone-enones (i.e., sulfonyl Nazarov reagents) and enolizable enals was developed by Díez et al. leading to highly functionalized cyclohexa-1,3-dienes (not shown).^157^

**Scheme 57:**
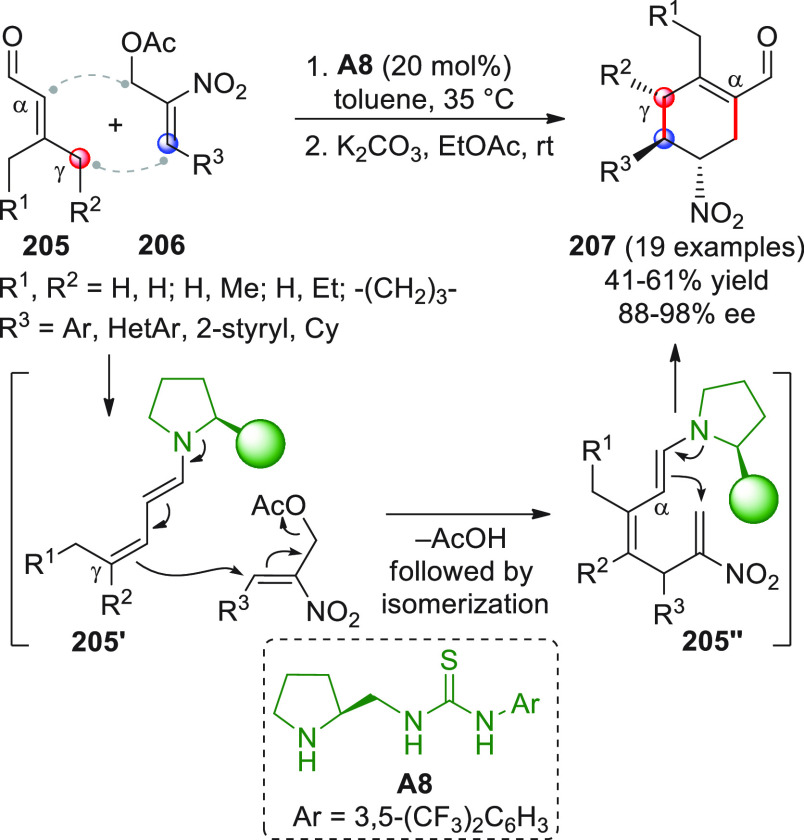


The multifaceted reactivity of dienamine species from α,β-unsaturated aldehydes toward electron-poor alkenes (e.g., γ,β-[2 + 2]-, γ,*ipso*-[4 + 2]-cyclizations, vide supra) could be extended further to include γ,α-[3 + 3] formal cycloadditions with suitable 1,3-bis-electrophilic partners (Scheme 57).^158^ When Chen and co-workers treated enolizable enals **205** with 2-nitroallylic acetates **206** using catalyst **A8**, cyclohexenal products **207** were obtained, as the result of a reaction cascade presumably comprising a vinylogous Michael addition of dienamine **205′** to the nitroalkenes, followed by a second intramolecular α-regioselective Michael addition involving **205″**. After catalyst screening, bifunctional secondary amine-thiourea catalyst **A8** was identified as the best catalyst in terms of reaction efficiency and enantioselectivity. However, due to low *cis/trans* diastereoselection in all assayed experimental circumstances, the product diastereomeric mixtures were subjected to base-promoted epimerization to afford the *trans*-isomers **207** exclusively.

**Scheme 58:**
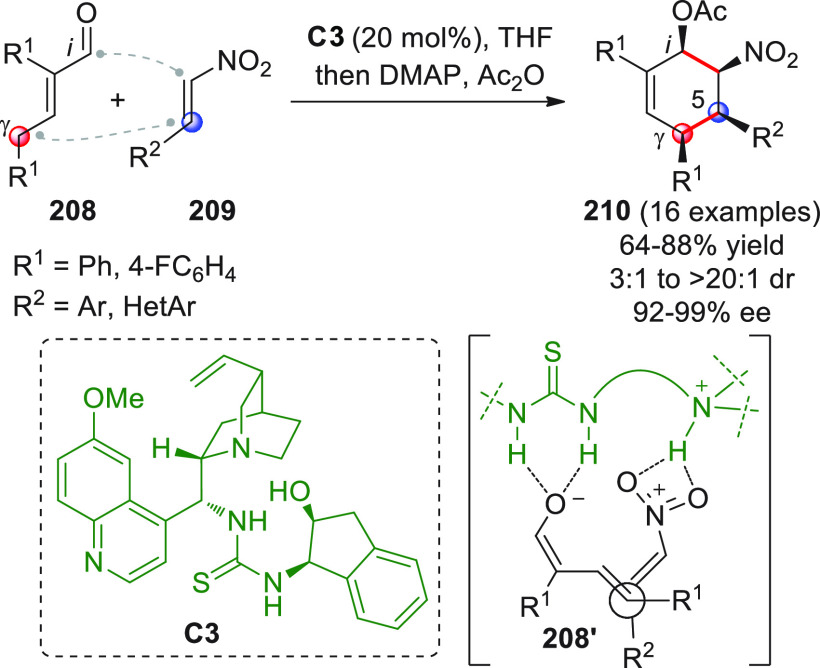


While the remote activation of enals using dienamine-mediated covalent catalysis was well established and widely exploited in synthesis, the noncovalent asymmetric and catalytic strategies based on the use of chiral Brønsted bases lagged behind, probably due to the inherent low reactivity in dienolate formation with mild bases. This task was cleverly faced by Xu et al., who succeeded in the HOMO-raising noncovalent activation of α-aryl-α,β-unsaturated aldehydes **208** by using bifunctional Brønsted base catalyst **C2** (Scheme 58).^159^

Such a catalyst would induce base-promoted enolization of the enal, while providing hydrogen-bonding activation of the reacting partners. The corresponding [4 + 2] cyclization products were obtained in high yields and with good-to-excellent enantioselectivities. During optimization studies, it was found that using catalyst **C3**,^160^*all-cis*-disposed products **210** could be accessed predominantly, accompanied by variable amounts of the corresponding C5-epimers. When an alternative squaramide-tertiary amine catalyst was used, instead, the C5-epimers of **210** were chiefly recovered, thus providing a general diastereodivergent route to the cyclohexene targets. Based on control experiments and previous reports, the authors proposed a working mode to rationalize the observed stereoinduction. Accordingly, after enal deprotonation, the ammonium ion portion of the catalyst would stabilize the nitro function of the acceptor as in **208′**, while the thiourea moiety would be able to stabilize the developing dienolate oxygen.

**Scheme 59:**
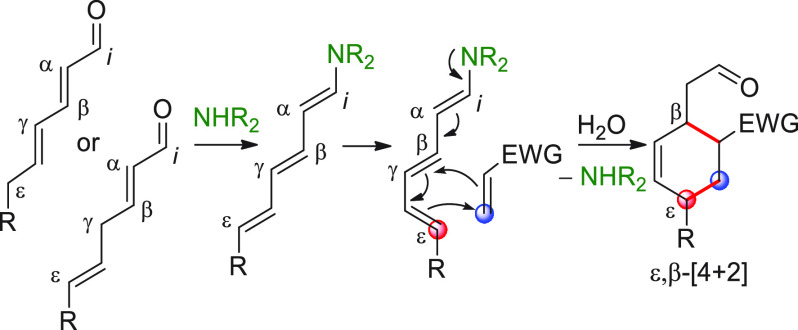


The condensation/isomerization of optically active secondary amines with enolizable fully or partially conjugated enals generates reactive trienamine species, whose intrinsic vinylogous nucleophilicity may be propagated through the π-system to the α, γ, until the remote ε-positions (Scheme 59).

In the presence of electron-deficient alkenes, these species can readily participate in ε,β-regioselective [4 + 2] cycloaddition processes as activated diene components, providing direct access to highly complex chiral cyclohexene frameworks. The viability of trienamine activation was for the first time established in a collaborative project between the Chen and Jørgensen groups in 2011,^161^ using either olefinic oxindoles or alkylidene cyanoacetates as suitable dienophile acceptors (Table 1, eq 1). On that occasion, the authors were able to prove with NMR studies the existence of the in situ generated *all-trans* trienamine intermediate as the result of the reaction between the starting polyenal substrate and the prolinol silyl ether catalyst (eq 1). The complete ε,β-regioselectivity of the [4 + 2] cycloaddition (overriding the possibly competing γ,*ipso*-[4 + 2] closure) could be rationalized by computational studies considering both the favorable rotation barrier for the formation of the reactive *s-trans,s-trans,s-cis*-trienamine conformation required for the cycloaddition and the energy of the frontier molecular orbitals set up for the interaction with the olefin. High *endo*-selectivity was explained by invoking secondary orbital interactions between the interacting partners, and face-selectivity was nicely secured by the proven ability of the chiral amine catalyst to govern the enantioface discrimination by steric factors. The authors hypothesized a plausible pericyclic Diels–Alder mechanism based on the absence of any observable Michael-addition type intermediates and high stereochemical induction indicative of a highly concerted closure.

**Table 1:**
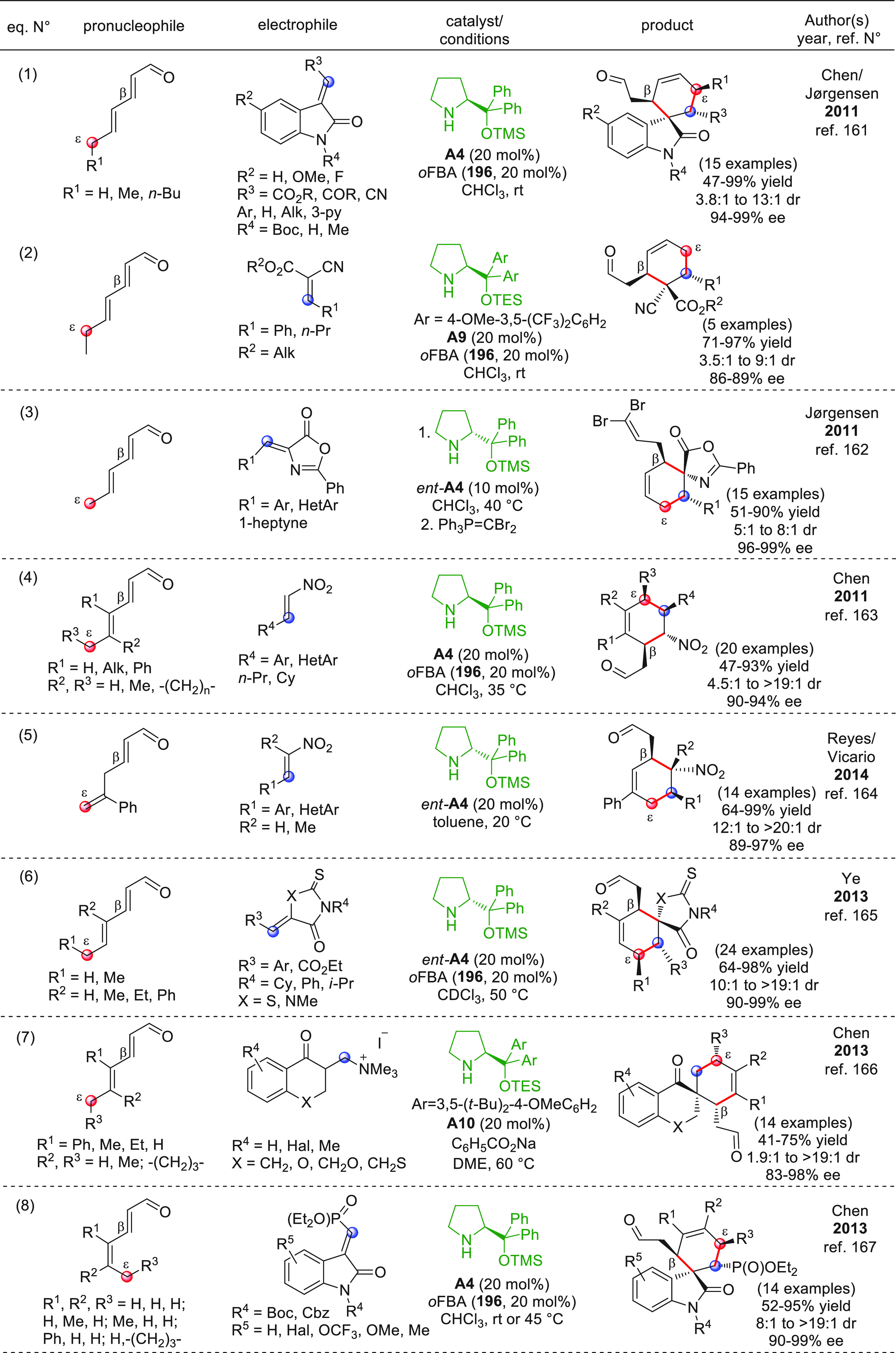

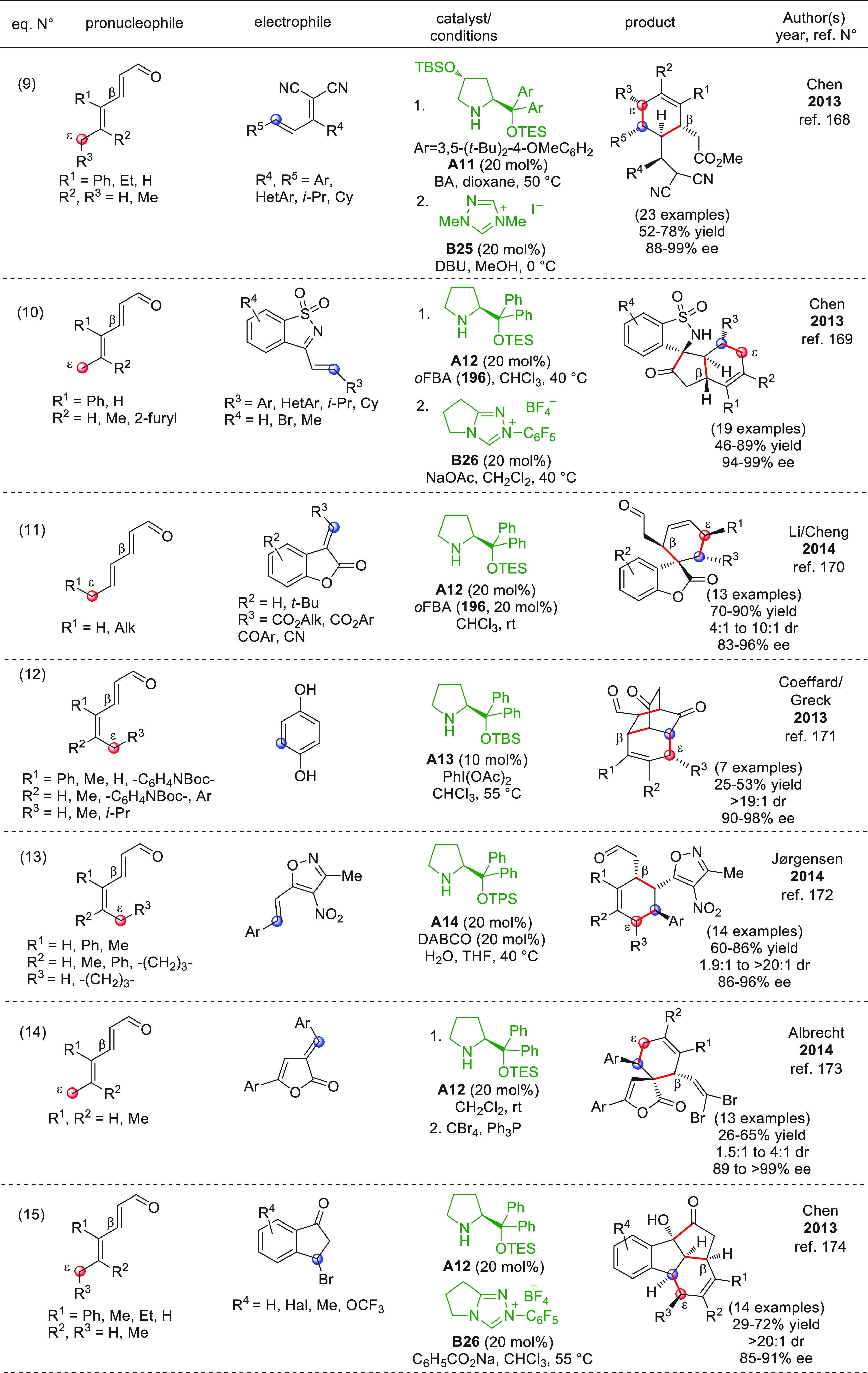

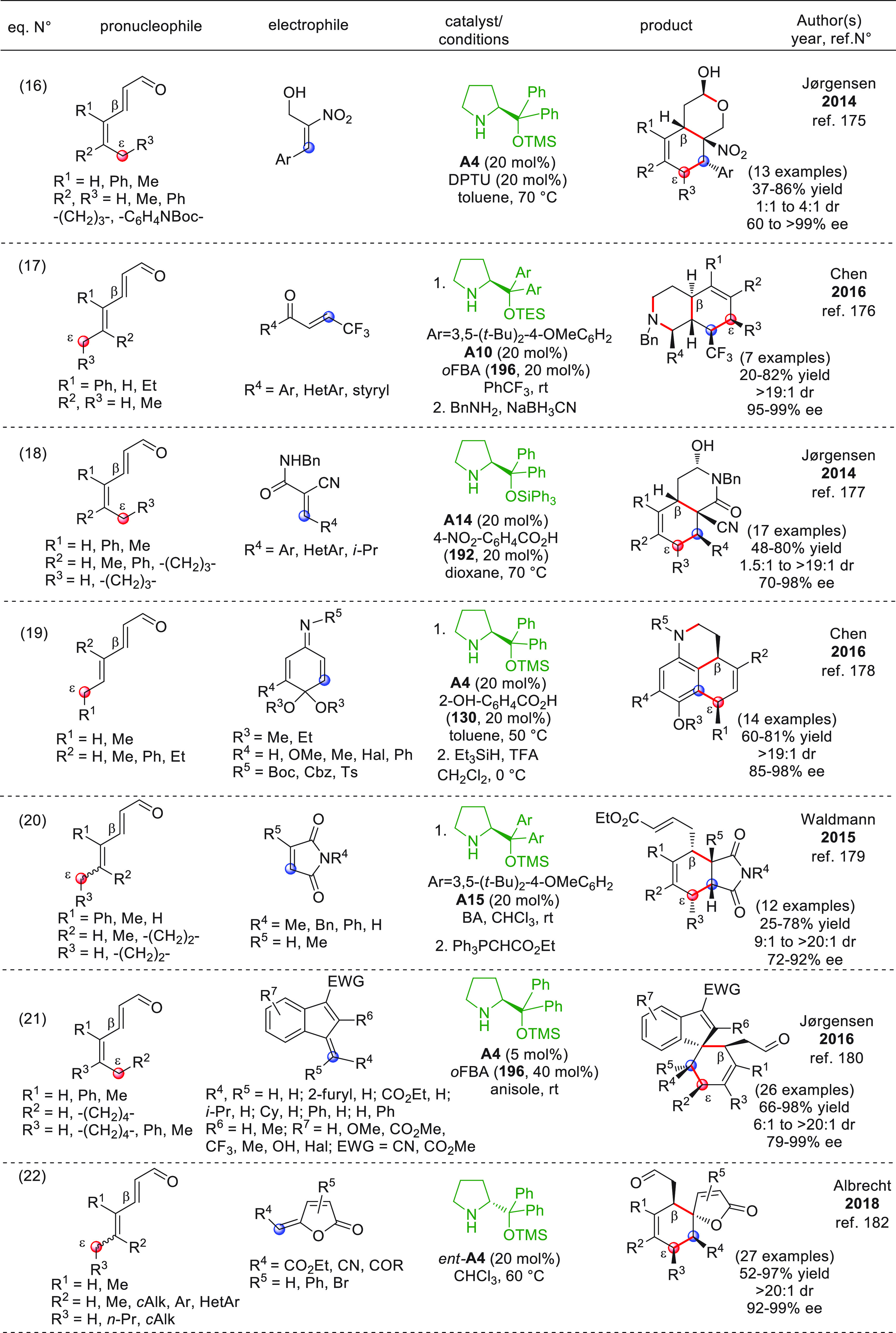

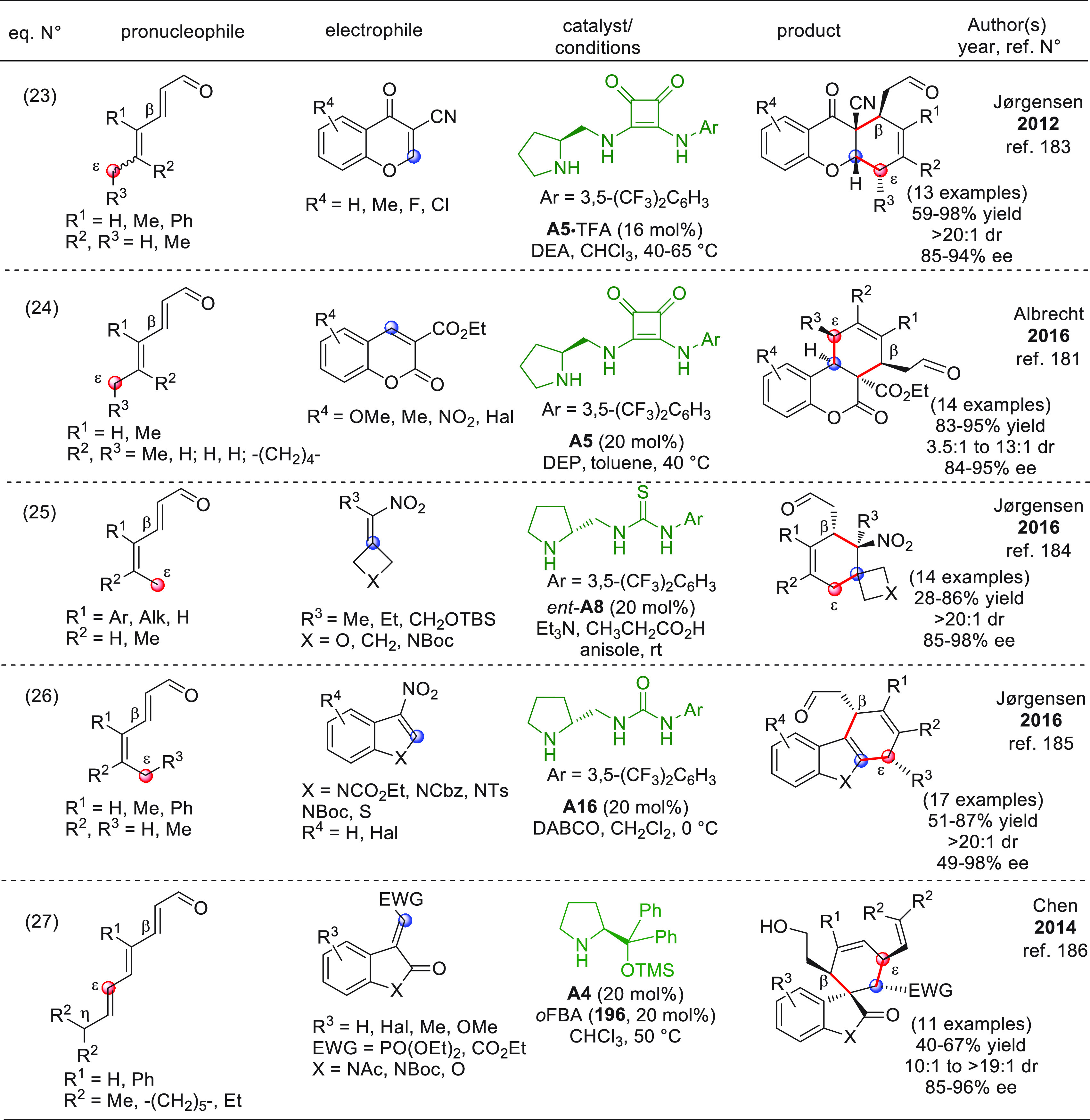
Dienals in Action with Electron-Poor Alkenes in ε,β-Regioselective Remote Cycloaddition Reactions

Henceforth, an impressive, flourishing number of reports appeared by these and other research groups, which focused on the ε,β-regioselective [4 + 2] cycloaddition between polyenals and a wide array of diverse dienophiles ranging from nitroalkenes, alkylidene heterocycles, to azadienes, enones, etc. For an immediate glance at this varied reaction panorama, a table is given (Table 1), collectively depicting these contributions.^162−186^

The richness of the cyclohexene products obtained appears evident, with hundreds of structurally and stereochemically diverse new C(sp^3^)-rich chemotypes becoming available with extraordinary simplicity and immediacy. In some cases (e.g., eq 4), ad hoc-placed electron-donating substituents at the γ,δ carbon sites of the starting enals ensured further HOMO activation while granting full ε,β-regioselectivity; in other instances (eq 5), nonconjugated polyunsaturated aldehydes were used as substrates to increase their reactivity during the condensation with the amine catalyst. The molecular diversity of the [4 + 2] products was increased even further by their transformation into secondary targets in either sequential or one-pot domino sequences, as in the case of amine/NHC cascade catalytic processes (eqs 9, 10, 15, 17, 19, and 20). In most examples, the immediate derivatization/transformation of the cycloadducts proved indispensable for product stabilization and/or isolation. Interesting examples within Table 1 also include in situ-activated electrophilic species, as in the case of methylene cyclic carbonyls from methiodide salts (eq 7), dearomatized quinones from phenols (eq 12), and indenones from 3-bromoindanones (eq 15). In one case (eq 20), the ε,β-[4 + 2] methodology was functional for accessing collections of products to be assayed in biology-oriented studies. In the majority of reports, the amine catalyst exerted its stereochemical induction via steric shielding control (eqs 1–22), while in some cases (eqs 23–26), a H-bond-directing approach was exploited using bifunctional amine catalysts.

From a mechanistic point of view, apart from the pioneering studies by Chen and Jørgensen,^161^ no experimental/computational studies were performed by the authors in order to substantiate the concerted vs stepwise nature of these [4 + 2] cyclizations; in one case (eq 14), a stepwise Michael/Michael reaction mechanism was openly postulated, while in another example (eq 26), calculations pointed to an asynchronous or stepwise mechanism. Computational work by Houk et al. indicated that, for a cyclic extended trienamine system (see later, cyclic systems), a stepwise mechanism might be operative.^187^

Lastly, a nice example of HOMO-activation strategy extended to highly conjugated enolizable 2,4,6-trienal substrates was reported, with the formation of multidentate tetraenamine intermediates under amine catalysis (Table 1, eq 27). In this case, the reaction with alkylidene oxindole acceptors gave the trienamine-type ε,β-locked [4 + 2] cycloaddition products regioselectively, as a testimony of the fact that the middle-positioned Cβ-Cε diene system of the reactive tetraenamine was involved in the coupling event. The alternative remote closure, involving the η,δ-diene, was not observed probably due to steric hindrance of ad hoc-placed substituents at the η-position.

##### 3.3.1.2 Cyclic Pronucleophiles

As disclosed in the previous sections (Scheme 22 and related text), asymmetric functionalization of benzylic C–H bonds of (hetero)aryl aldehydes or extended polyenal variants is one major focal point of the chemistry currently powering the field of vinylogy, and different remote activation strategies have been devised, especially in direct, organocatalytic approaches.

Considering the special focus of this subsection, namely, the conjugate addition to electron-poor alkenes, we opted to group the research contributions into three categories, depending of the structural features of the pronucleophilic aldehydes.

In a first scenario, the aldehyde carbonyl group is distanced from the heteroaromatic ring by one carbon–carbon double bond, which is positioned *ortho* to the enolizable benzyl site, as outlined by examples in Table 2 (eqs 1–5).^188,189^

**Table 2:**
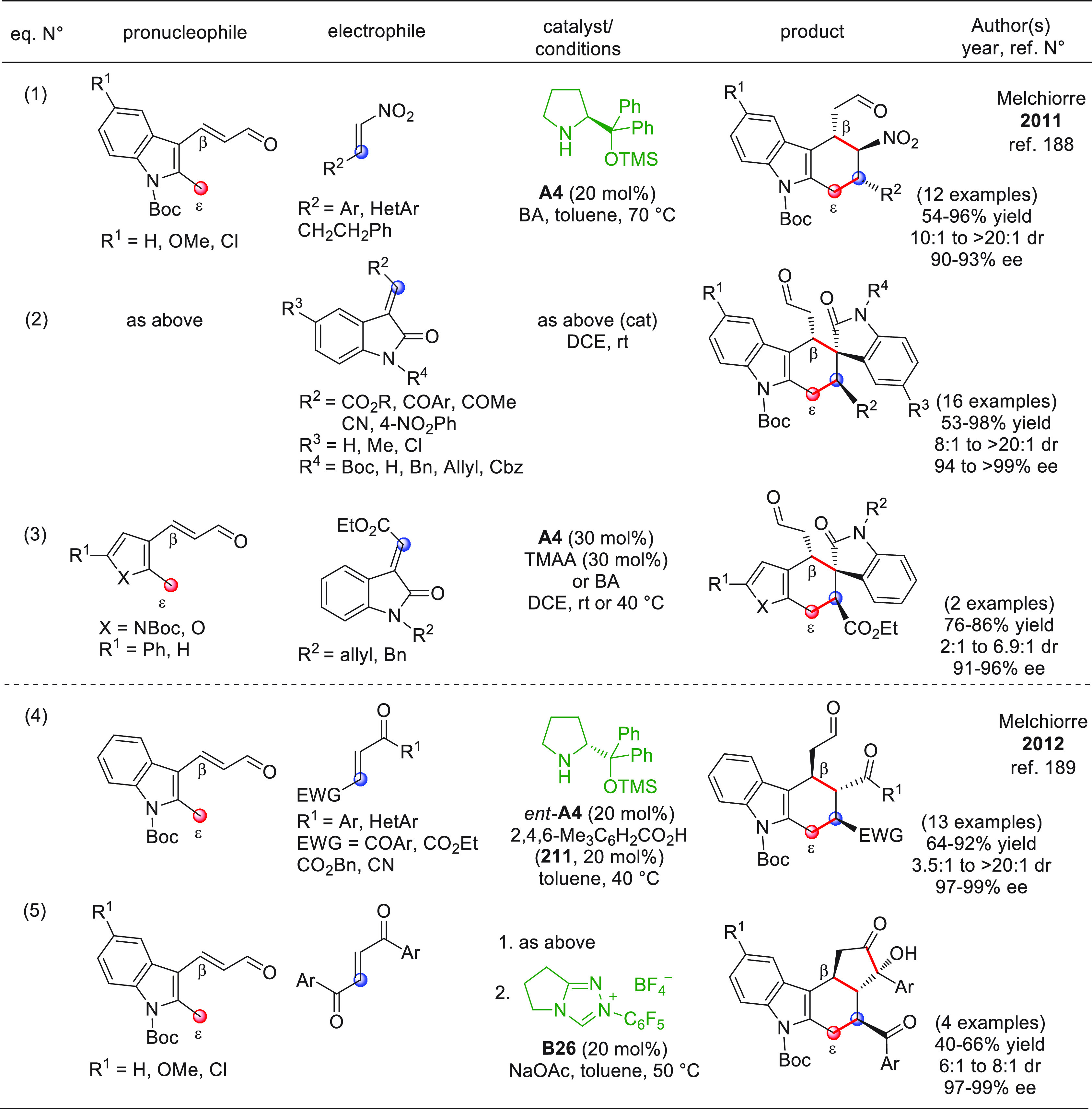
Functionalization of Benzylic C–H Bonds of π-Extended Heteroaryl Aldehydes: ε,β-Regioselective Remote Cycloaddition Reactions with Electron-Poor Alkenes

The group of Melchiorre first devised a clever strategy according to which amine-catalyzed activation of the starting pronucleophile leads to the formation of a HOMO-raised trienamine intermediate (see species **XX** in Scheme 22), a temporarily dearomatized *o*QDM species that can serve as a highly reactive *s-cis*-locked terminal diene in useful [4 + 2] cycloaddition reactions with suitable dienophiles. In the event, ε,β-closure of the starting polyenal occurs, producing highly functionalized ring-fused cyclohexene products bearing an exocyclic acetylaldehyde moiety, with the possibility of complete catalyst recovery and recycling. 2-Methyl-substituted indole, furan, and pyrrole-based enals were cleanly reacted with diverse dienophiles, including nitroalkenes, alkylidene oxindoles, and enones, affording the corresponding sp^3^-rich polycycles in generally good yields and stereoselectivities. One-pot, multicatalytic approaches could also be exploited, as exemplified by the one-pot Diels–Alder/benzoin cascade reaction reported in eq 5 (Table 2). In all instances, the regioselectivity of the [4 + 2] cyclizations was strictly dictated by the favorable frontier orbital interaction between the reacting diene/dienophiles partners, with full vinylogous transmission of the pronucleophilic character along the trienamine π-system to the remotely positioned benzylic Cε-site. The [4 + 2] cyclization was considered to be a true pericyclic Diels–Alder reaction, though no further supporting evidence was given at the time.

A second class of pronucleophilic species is represented in Table 3, where the aldehyde function is directly attached to the (hetero)aromatic ring and the enolizable benzylic site is positioned away (a sort of “*para*”-substitution) from the formyl function. The covalent activation by a chiral secondary amine catalyst would thus produce trienamine (structure **XXI**, related to eqs 1–2, Table 3) or tetraenamine (**XXII**, eq 3, Table 3) intermediates, which are temporarily dearomatized and chiralized species.

**Table 3:**
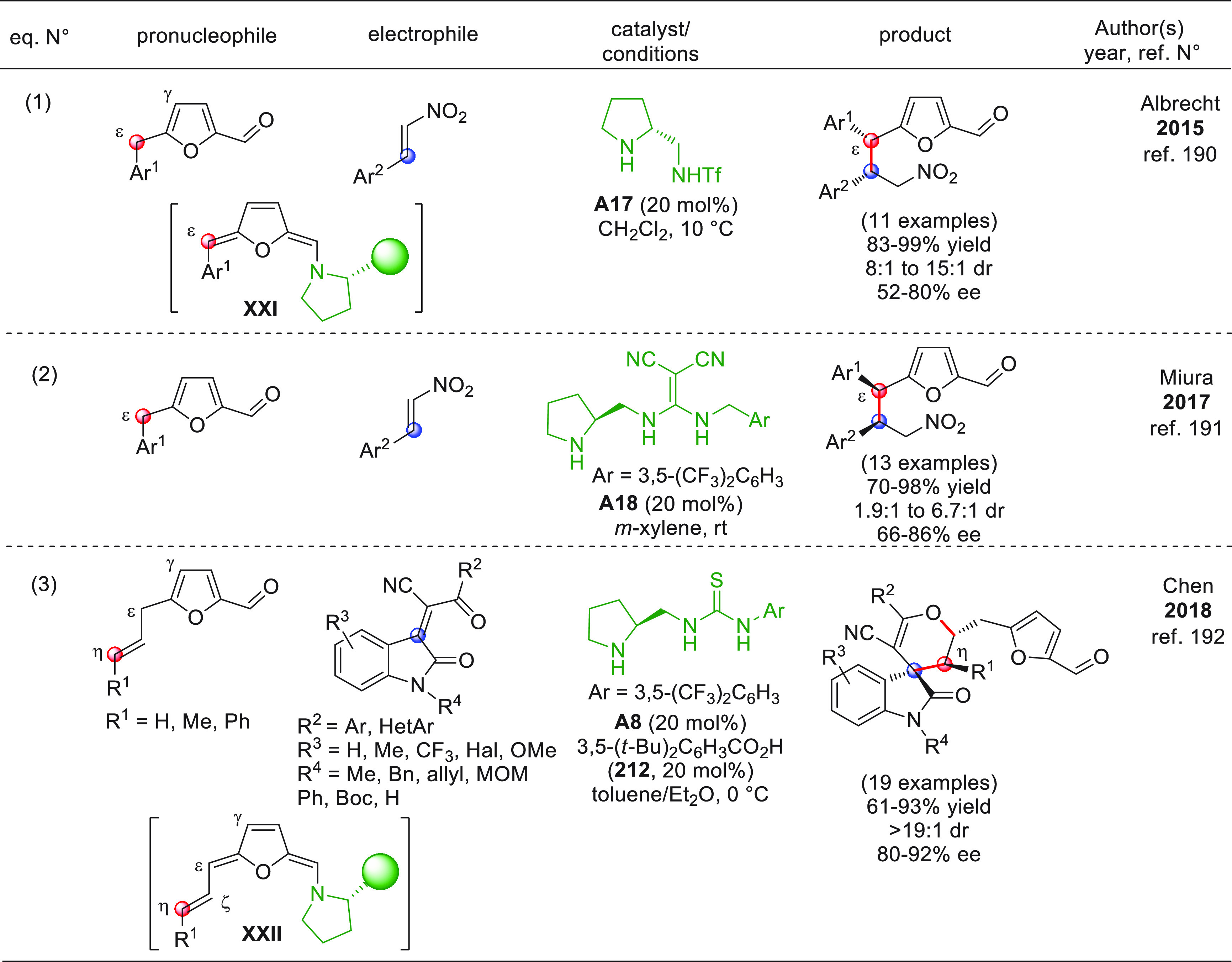
Regioselective Remote Functionalization of Benzylic or More Remote C–H Bonds of “*p*-Substituted” Heteroaryl Aldehydes with Electron-Poor Alkenes

In the first two examples, independently authored by Albrecht et al. and Miura et al. in different years (Table 3, eqs 1–2),^190,191^ a ε-regioselective bisvinylogous Michael addition occurs on nitroalkene acceptors, since the conformationally locked trienamine intermediate **XXI** prevented any pericyclic cycloadditions involving the β,ε-sites of the terminal diene.

In a third, recent example, instead (Table 3, eq 3), authored by Chen et al.,^192^ the remotely positioned ζ,η-C=C double bond of the tetraenamine intermediate **XXII** is free to act as HOMO-raised 2π-component in IED-HDA [4 + 2] cycloadditions with alkylidene oxindole oxadienes acting as 4π-partners, to furnish the corresponding spirocyclic oxindole products incorporating dihydropyran-furfural moieties. In the three works, the remote asymmetric induction, exterted by the dual catalysts, could consistently count on additional activation of the electrophile via advantageous H-bonding interactions.

A third structural scenario is given by *ortho*-alkyl-substituted (hetero)aryl aldehydes, as represented in Table 4.

**Table 4:**
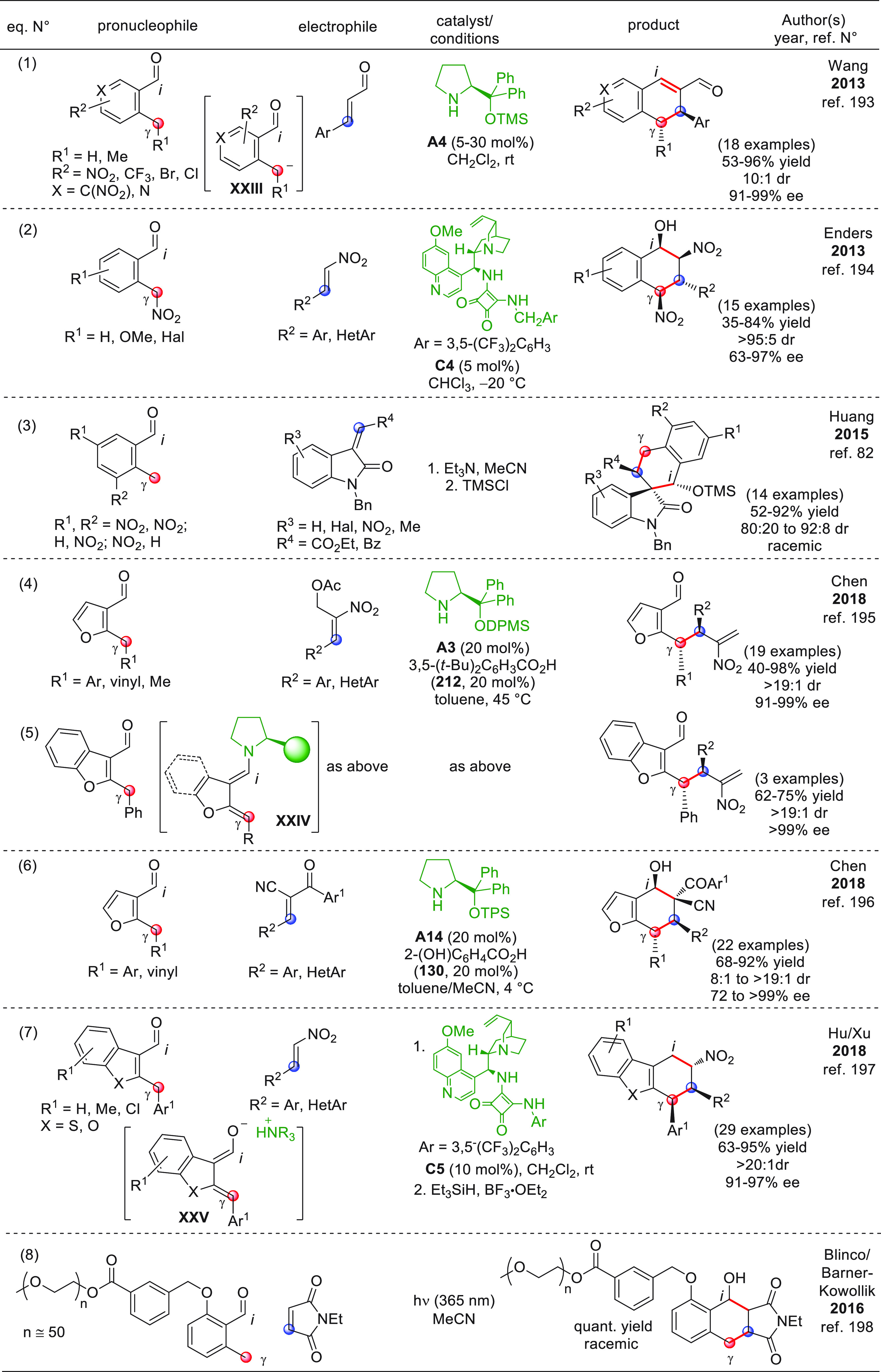
Regioselective γ- or γ,*ipso*-Remote Functionalization of Benzylic C–H Bonds of *ortho*-Alkyl-Substituted (Hetero)aryl Aldehydes with Electron-Poor Alkenes

In the independent works by Wang,^193^ Enders,^194^ and Huang^82^ (Table 4, eqs 1–3), ad hoc placed electron-withdrawing groups (e.g., nitro) within the benzene ring provided enough C–H acidity at the benzylic position for this to be easily deprotonated by mild Brønsted bases. The resulting carbanionic active intermediates of type **XXIII** (resonating as *o*QDM/nitronate species, not shown) undergo [4 + 2] cycloadditive reactions with activated alkenes, providing the corresponding six-membered carbocycles. An eliminative stepwise vinylogous Michael/aldol cascade reaction was supposed to intervene in the first case (eq 1), with the covalent activation of the electrophile component via iminium ion formation. In the second instance (eq 2), dual catalysis by the tertiary amine/squaramide catalyst ensured the concomitant activation of the coupling substrates via stepwise nitroalkene-Michael/Henry cascade. In the third work (eq 3), an achiral tertiary amine afforded the appropriate deprotonation–cyclization reaction to access the silyl-stabilized racemic cycloaldol products.

In a recent work, Chen et al. developed a rare example of covalent amine-activation of enolizable *o*-alkyl furfural (or benzofuran analogues) (Table 4, eqs 4–5).^195^ The supposed catalyst-tethered dearomatized *o*QDM intermediate **XXIV** was engaged in Michael-type addition to nitroallylic acetates to furnish, after acetic acid elimination, the benzylic alkylation products.

This research group exploited the same catalyst-bound *o*QDM activation strategy in a subsequent work (eq 6),^196^ using α-cyanochalchones as electrophilic partners. The corresponding [4 + 2] cyclohexanol products were conveniently obtained, as the result of a postulated stepwise vinylogous Michael/intramolecular aldol cascade. The stepwise nature of this addition made hydrolysis of the iminium ion intermediate possible after the first step and thus allowed for catalyst recycling.

An interesting and unprecedented strategy was devised by Hu, Xu, et al. (Table 4, eq 7),^197^ who exploited the dual tertiary amine/squaramide catalyst **C5** for the noncovalent activation of heteroaryl aldehydes. The in situ-generated *o*QDM-dienolate species of type **XXV** were intercepted by nitroalkene acceptors to afford the cyclic products via stepwise vinylogous Michael/nitroaldol cascade followed by reductive OH elimination.

Finally, a similar γ*,i-*selective [4 + 2] cycloaddition was exploited by Blinco, Barner-Kowollik, and colleagues to demonstrate how photochemically induced γ-enolization of funtionalized *o*-methylbenzaldehydes could undergo fast and chemoselective cycloaddition to maleimide, even in the presence of an amine competitor (eq 8)^198^ (for the light-induced formation of *o*QDM dienolates from carbonyl compounds, see the next section on ketone substrates). In the event, irradiation of the starting benzaldehyde at λ_max_ = 365 nm led to the quantitative conversion of the starting material to the Diels–Alder product after 7 min, whereas when the irradiation was switched off, benzaldehyde reacted exclusively, under otherwise identical experimental conditions, with the amine coreagent, giving the corresponding imine (not shown) in 23% yield. The orthogonality of the reaction path was then cleverly exploited for the selective synthesis of block copolymers.

**Scheme 60:**
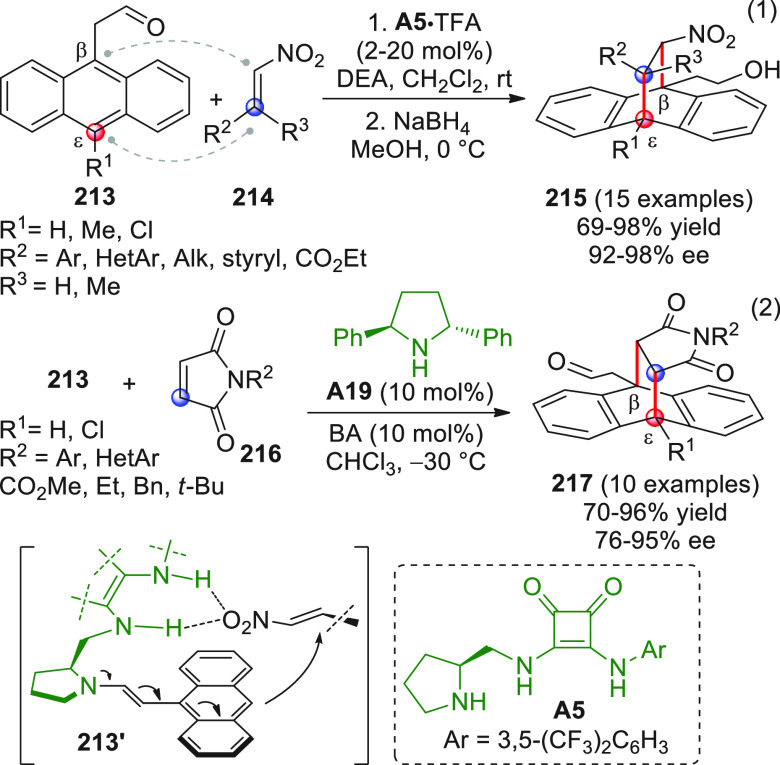


In 2012 and 2013, the group led by Jørgensen disclosed a new activation concept for anthracene derivatives by using covalent aminocatalysis.^199,200^ The basic idea was as clever as it was simple: treating α-enolizable 9-acetylaldehyde-substituted anthracenes **213** (Scheme 60) with suitable secondary amine catalyst would provide the corresponding enamine (e.g., **213′**) with direct extended conjugation to the π-system of the central anthracene ring. The aromaticity of this ring was calculated (by nucleus-independent chemical shift, NICS-method) to be less than that of the same ring in anthracene or the parent aldehyde-anthracene **213**; on the other hand, the HOMO of this special “trienamine” system was higher in energy compared to **213**. These calculations predicted that “trienamines” of type **213′** could be favorably engaged in ε,β-selective [4 + 2] Diels–Alder cycloaddition reactions with suitable electron-poor alkenes and emphasized the role of the catalyst in accelerating aromaticity-breaking toward the targets.

In the first contribution (Scheme 60, eq 1),^199^ anthracene aldehydes **213** were reacted with aryl- or alkyl-substituted nitroalkenes **214**, using bifunctional secondary amine/squaramide catalyst **A5**, affording, after carbonyl reduction, cycloadducts **215** with efficiency and high enenatioselectivities. Here, the excellent enantiofacial discrimination was dictated by the strict H-bonding control exerted by the bifunctional catalyst **A5**, as depicted in **213′**. In a subsequent report (Scheme 60, eq 2),^200^ the same anthracene aldehydes **213** were treated with maleimides **216** (and even maleic anhydride or substituted cyclopentenes, not shown) under the guidance of *C*_2_-symmetric amine catalyst **A19**. In this instance, the good stereoselectivities observed in the formation of cycloadducts **217** could be explained by both experimental and computational studies. It was observed that analogous reactions, using nonsymmetric aminocatalysts, produced lower selectivities, pointing to the notion that symmetry-breaking could be cleverly effected by *C*_2_-symmetric catalysts. DFT studies explained that the discrimination between the two possible enantiofaces of the dienophile was not the result of a steric shielding effect, but was instead due to the extended (or prevented) π-conjugation along the trienamine chain in the preferred vs disfavored transition state structures. In any case, calculations suggested that the reaction pathway was concerted and highly exothermic.

**Scheme 61:**
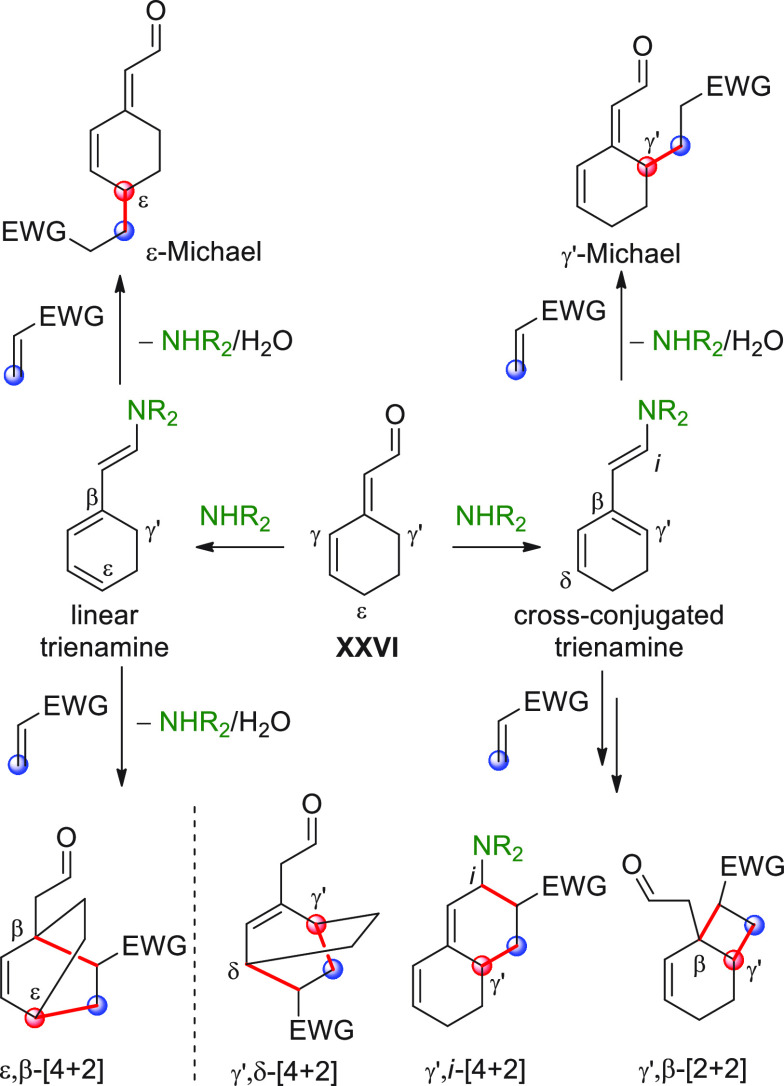


Among cyclic pronucleophilic polyenals, the class of cycloalkenylidene acetaldehydes (e.g., structure **XXVI**, Scheme 61) has enjoyed notable success, especially thanks to the studies performed by the Jørgensen group.

The covalent HOMO-raising activation of these substrates via amine organocatalysis may lead to the corresponding extended or cross-conjugated trienamines (or both) which can couple, at least in principle, to suitable electron-poor olefins during diverse and competitive reaction itineraries, including “simple” vinylogous (at γ′) or hypervinylogous (at ε) Michael-type additions, as well as ε,β-[4 + 2], γ′,δ-[4 + 2], γ′,*i*-[4 + 2], and γ,β-[2 + 2] cycloadditions (Scheme 61) or even γ′,β-[4 + 2] HAD cycloadditions (not shown). Carrying out regio- and stereocontrolled reactions in such a multifaceted scenario can thus result in a challenging venture.

**Scheme 62:**
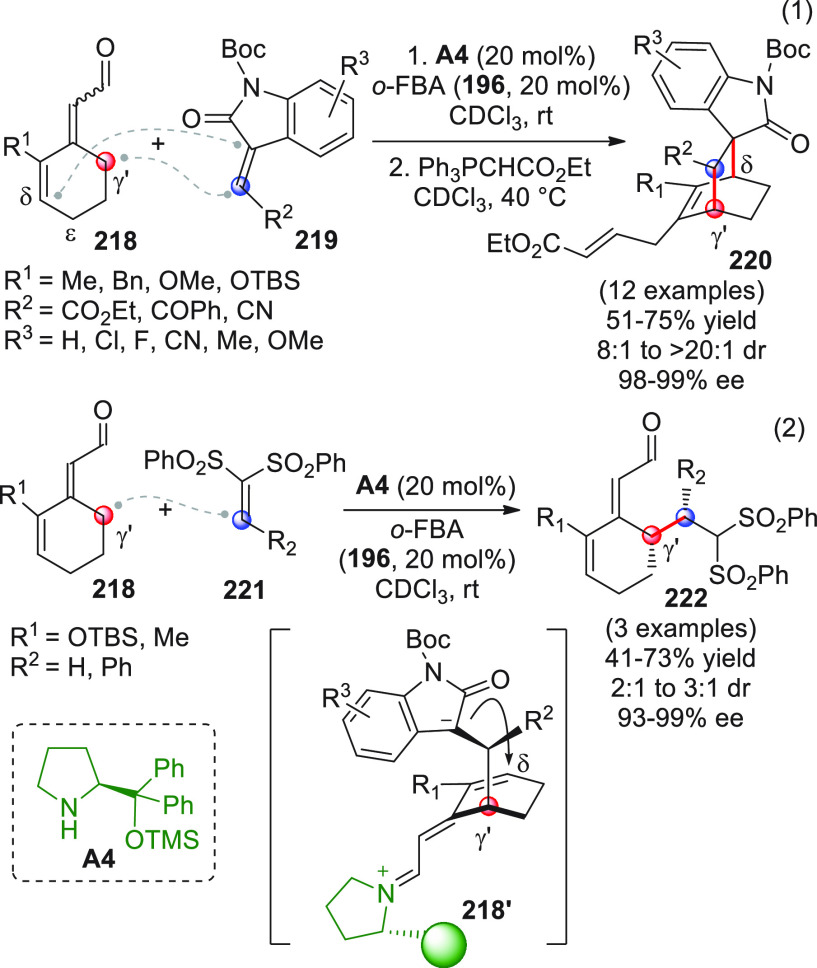


In 2012, Jørgensen and co-workers faced this task by reacting 2,4-dienals **218** with 3-olefinic oxindoles **219** under prolinol silyl ether catalysis (catalyst **A4**, 20 mol %) (Scheme 62, eq 1).^201^ The spiro-polycyclic products **220** were exclusively obtained (isolated as unsaturated esters after Horner–Wadsworth–Emmons olefination), as the result of a γ′,δ-regioselective [4 + 2] cycloaddition to the dienophiles **219** via cross-conjugated trienamine (see also Scheme 61). The generality of the reaction was proven, and wide structural variations in both the reacting partners were well tolerated, producing cycloadducts **220** in good yields and excellent stereoselectivities. The same cross-conjugated trienamine concept was successfully applied to aryl-substituted olefinic azlactone dienophiles, affording [4 + 2] products, again with exclusive γ′,δ-regioselectivity (not shown). When, instead, 1,1-bis(phenylsulfonyl)ethylenes **221** were employed as electrophiles (eq 2), γ′-substituted products **222** were isolated, as the result of γ′-regioselective vinylogous Michael addition reaction.

To clarify the reason why cycloadditions and Michael additions occurred via the cross-conjugated trienamine, a combination of both computational and NMR studies were performed. Calculations were carried out on a simplified model system related to the two possible trienamines (extended vs cross-conjugated) and showed that deprotonation at the most remote ε-position is favored, since it provides a fully conjugated, thermodynamically stable π-system. Despite furnishing the linear trienamine in higher concentrations, the authors reasoned that this reaction system would progress toward the cross trienamine-triggered γ′,δ-pathway since it would be able to give the thermodynamically more stable products **220** (thermodynamically controlled reaction) via a highly asynchronous, concerted Diels–Alder mechanism.

Having suspects about the actual concerted nature and the thermodynamic control of this cycloaddition, Houk and co-workers reanalyzed and rationalized the behavior of this reaction by employing in-depth DFT calculations.^187^ Interestingly, they concluded that a stepwise mechanism for this formal [4 + 2] cycloaddition is probably operative, entailing a first, thermodynamically and kinetically favored vinylogous Michael addition of the γ′-site donor to the olefin acceptor to produce a zwitterionic intermediate of type **218′** (Scheme 62), after which, such an intermediate would close, under thermodynamic control, to give the experimentally observed γ′,δ-cycloadducts, overriding the alternative, yet possible, competitive closures (e.g., γ′,*i*-[4 + 2], γ,β-[2 + 2] cycloadditions, Scheme 61).

**Scheme 63:**
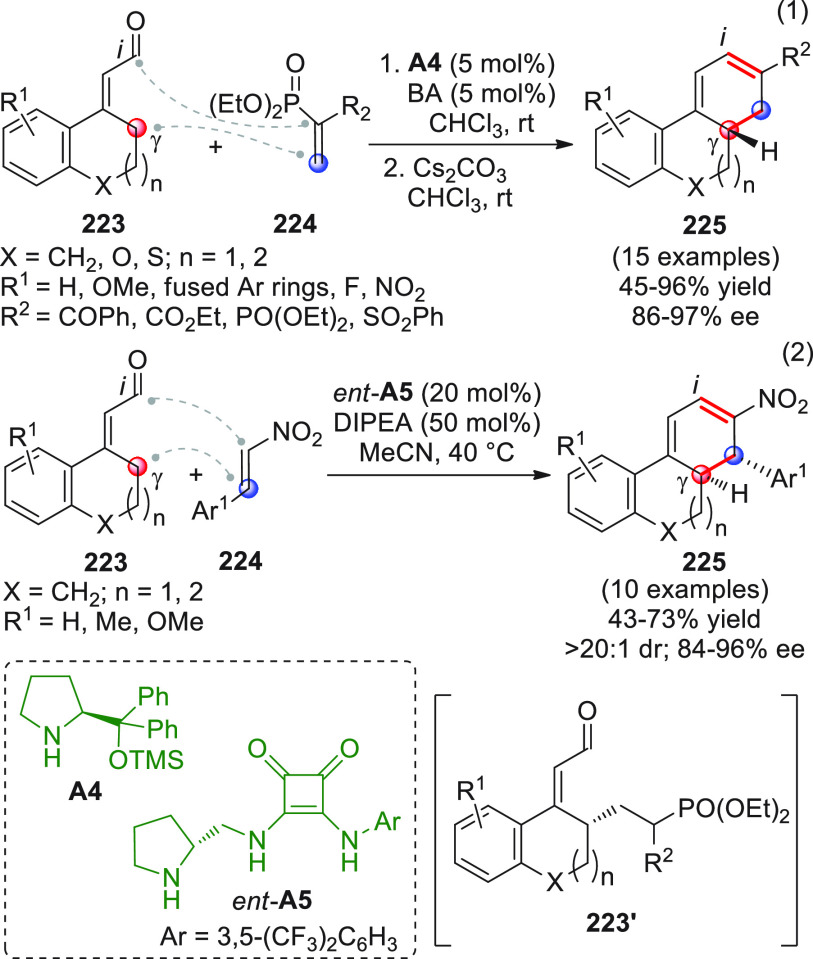


Carbocyclic and heterocyclic aromatic cycloalkylidene aldehydes **223** were the key substrates utilized by the Jørgensen group during a synthesis campaign involving amine-catalyzed coupling reactions with various electron-deficient olefin types (Scheme 63).^202^ In a first group of reactions (Scheme 63, eq 1), enals **223** were treated with phosphonate olefins **224** using catalyst **A4** and benzoic acid as an indispensable acid additive. After the one-pot addition of cesium carbonate as the base, products **225** were isolated in good yields and stereoselectivities. These products were the result of a one-pot, two-step cascade reaction entailing a first γ-regioselective vinylogous Michael addition of **223** to **224** to furnish **223′**, followed by intramolecular base-promoted HWE olefination. Furthermore, the reaction employing cyclohexene-derived enal behaved impeccably (not shown) while, as a limitation, β-substituted vinyl phosphonates did not perform well under these experimental conditions. Alternative nitroalkene acceptors were also used (eq 2) in reactions with carbocyclic donors **223** (X = CH_2_) by using bifunctional organocatalyst *ent*-**A5** and DIPEA as an additive. Again, the cyclohexadiene products **225** were obtained, derived by (at least formal) eliminative γ,*i-*[4 + 2] cycloaddition to the nitroalkenes, where the DIPEA base was thought to play a beneficial role during the E_1CB_-elimination of the amine catalyst. Of note, while in the first group of reactions (eq 1), a steric shielding effect by the catalyst was invoked to account for the observed stereoselectivity, hydrogen-bonding assistance by the dual-action catalyst *ent*-**A5** was hypothesized for the latter group of reactions (eq 2).

Cyclic dienal substrates similar to **223** were also cleverly employed in γ,*i*-regioselective [4 + 2] cycloaddition reactions with either cyclopentadiones or quinone-based dienophiles, to access important 14β-steroids and D-homosteroids, respectively (not shown).^203^ In all cases, secondary amine catalyst **A4** was employed, which ensured excellent stereocontrol via the usual dienamine intermediate.

The eliminative γ,*i*-regioselective [4 + 2]-cycloaddition of racemic 2-cyclohexenylidene acetaldehydes with benzoquinones under asymmetric organocatalysis was also exploited by the same research group to achieve dynamic resolution of the starting aldehydes (not shown).^204^

**Scheme 64:**
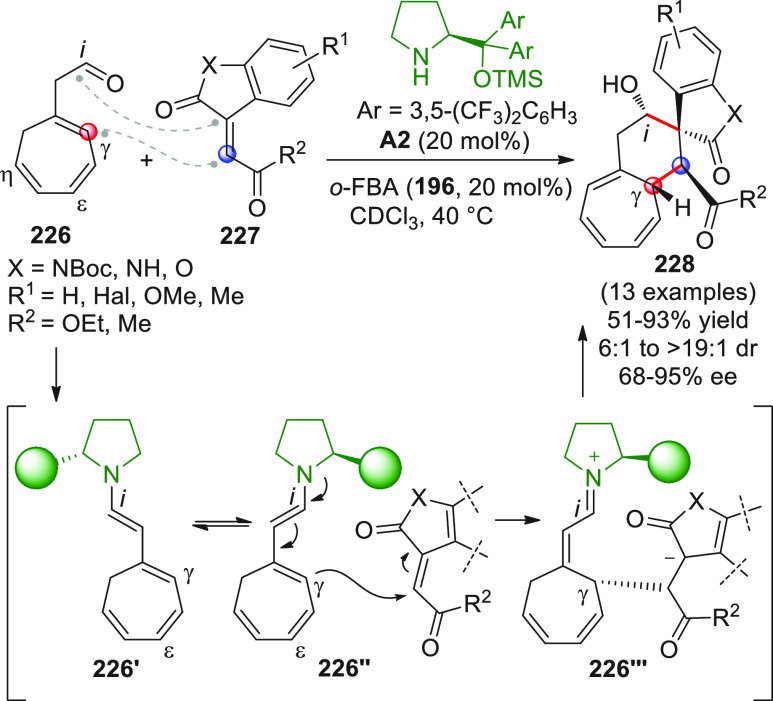


Cycloheptatrienyl acetaldehyde **226** was the carbonyl substrate chosen by Jørgensen and collaborators to demonstrate the viability of the first asymmetric organocatalyzed [4 + 2] cycloaddition via tetraenamine intermediate (Scheme 64).^205^ Treatment of deconjugated aldehyde **226** with amine catalyst **A2** led to the formation of fully conjugated tetraenamine **226′**, as demonstrated by NMR analysis. This HOMO-activated species shows multidentate nucleophilicity at the α, γ, ε, and η sites; nevertheless, when it was reacted with alkylidene oxindoles (or benzofuran analogues) of type **227**, a highly regio- and stereoselective formal γ,*i*-[4 + 2] cycloaddition occurred, giving the new class of spiroxindole products **228** incorporating fused 7/6-membered rings and four contiguous stereocenters. Though the annulation was limited to trienyl aldehyde **226**, the generality of the coupling reaction was wide with respect to the acceptor component **227**, since different R^1^, R^2^, X substituents could confer good to optimal reactivity, with production of the corresponding compounds **228**. NMR studies together with calculations and further experiments leading to the isolation of useful intermediates all sustained a stepwise mechanism entailing a first γ-vinylogous Michael addition between the reactive *s-cis* tetraenamine conformer **226″** and the acceptor **227** to provide the zwitterionic intermediate **226‴**. Subsequently, hydrolytic release of the catalyst, isomerization, and cyclization consigned the targeted compounds. Notably, intramolecular closure involving directly **226‴** could indeed occurr, which would entrap the catalyst and prevent catalytic recycle. However, this unproductive cyclization step was calculated to be reversible and therefore uninfluential to the overall efficiency of the process.

Trienamine organocatalysis was the central theme of an interesting work by Anderson et al., who reported how regioisomeric azacycle-tethered exocyclic dienals, deriving from palladium-catalyzed cycloisomerization of enynamides, could take part in ε,β-regioselective [4 + 2] cycloaddition processes with electron-poor alkenes, to give hexahydroindole products (not shown).^206^

To conclude this section, we will comment about a couple of recent, brilliant works on higher-order cycloadditions. Higher-order cycloadditions are cycloaddition reactions involving more than 6π electrons. Though they have been conceptualized and recognized for their high synthetic potential since the times of Woodward and Hoffmann more than 50 years ago,^207^ their full exploitation in asymmetric synthesis has been rising satisfactorily only over the past recent years. One of the major concerns about these reactions is the difficulty of judiciously channeling the pericyclic reaction toward a selected pathway (periselectivity) among the many competing itineraries; asymmetric aminocatalysis was envisioned to be an optimal tool to promote both peri- and enantioselectivity in this intriguing class of cycloadditions. The research groups of Jørgensen,^208^ Chen,^209^ and Hayashi^210^ contributed with excellent works to this field, and we report here only those studies that are conceptually connected to the focus of this review (for examples involving ketone pronucleophiles, see the next section).

**Scheme 65:**
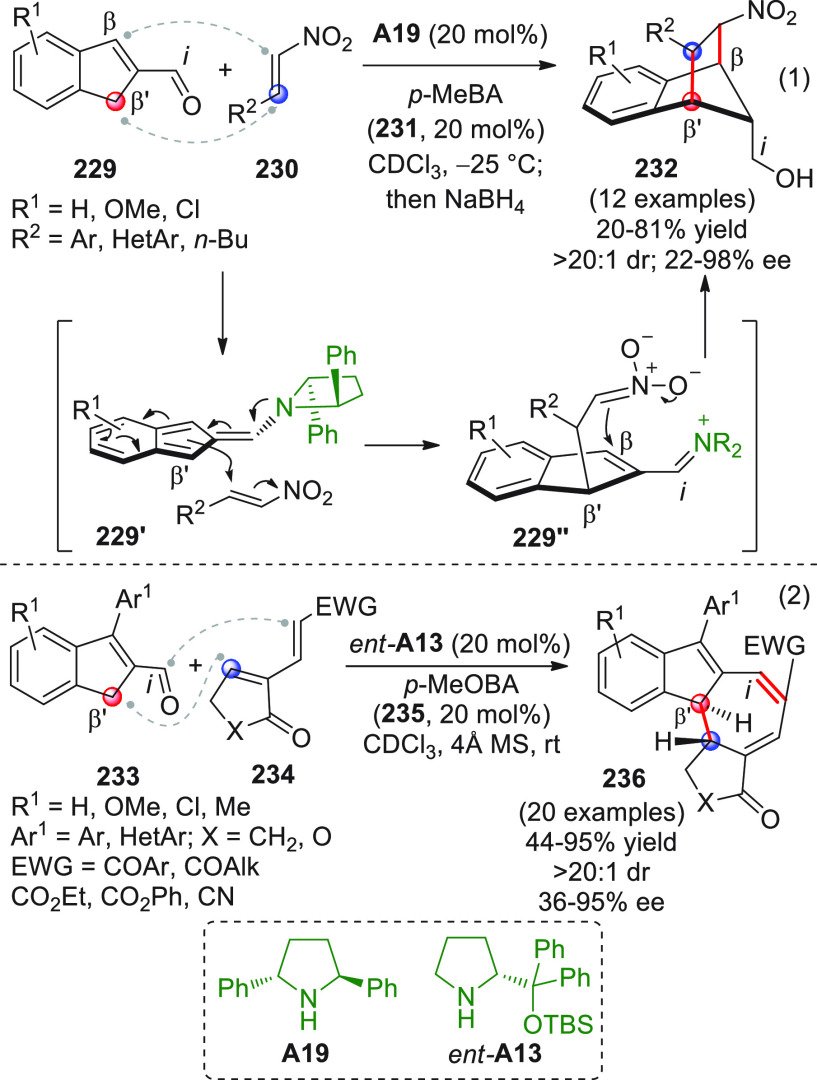


In a recent communication, Jørgensen et al. documented the first aminocatalytic [8 + 2] cycloaddition between indene-2-carbaldehydes **229** and nitroalkenes **230** (Scheme 65, eq 1).^211^*C*_2_-Symmetric 2,5-diphenylpyrrolidine **A19** (20 mol %) was identified as the best amine catalyst to promote the intended β′,β-selective [8 + 2] cycloaddition via the catalytic formation of amino isobenzofulvene intermediate **229′** acting as the 8π component. Investigation of the scope of the nitroolefins demonstrated that both aromatic and heteroaromatic substrates **230** could productively afford cycloadducts **232** in good yields and selectivities, with the exception of *ortho*-substituted aromatic moieties giving poor results. Furthermore, an alkyl-substituted derivative (R^2^ = *n*-Bu) gave poor results in terms of conversion but maintained high stereocontrol. Among the other olefins assayed, β-substituted unsaturated nitriles gave successful results (not shown), while electron-rich olefins did not work. Quantum mechanical calculations suggested a kinetically controlled, stepwise mechanism involving initial reversible formation of the zwitterionic species **229″** derived by the attack of the β′ site of semiaromatic polyenamine **229′** onto the nitroolefin. Subsequently, irreversible closure of the nitronate onto the Cβ-site provided an enamine intermediate and hence the observed product after catalyst hydrolysis (and NaBH_4_ carbonyl reduction). Of note, during calculations, an alternative [10 + 4] cycloaddition pathway was identified as a possible route (e.g., deriving by attack of the nitronate oxygen on the iminium ion within **229″**), even if no [10 + 4] adducts were observed with the employed olefins under these experimental conditions.

Intrigued by this unexplored, yet possible alternative, the same group soon after developed a strategy where such rare [10 + 4] couplings could be successfully realized in an aminocatalytic asymmetric context.^212^

As outlined in Scheme 65 (eq 2), various 3-aryl-substituted indenes **233** were used as 10π components (3-unsubstituted congeners of type **229** gave, instead, complex product mixtures), which were reacted with electron-poor cyclopentenone (or furanone) dienes **234** as 4π partners, giving rise to tetracyclic products **236** with generally good yields, excellent diastereocontrol, and appreciable enantioselectivities. Amine catalyst *ent*-**A13** was found to be the best aminocatalyst (20 mol %), together with the indispensable presence of *p*-methoxybenzoic acid additive. Experimental and computational evidence suggested that the observed stereoselectivity could derive from the kinetically controlled formation of an amino isobenzofulvene intermediate similar to **229′**, which could undergo stepwise eliminative closure to give the overall β′,*ipso*-selective [10 + 4] cycloaddition.

### 3.4 Other Reactions

The exploitation of the vinylogy concept goes well beyond the “conventional” addition reaction domain covered in the previous chapters, but it usefully includes examples of other reaction types such as alkylation (including cylopropanation, allylation), amination, nitrosation, thia-Diels–Alder cycloaddition, aziridination and protonation reactions. Indeed, we must remember that the first example of remote (γ) functionalization of enals via dienamine catalysis was an amination reaction.^213^ Most of the studies appearing in the 2010–2018 period involve the direct remote activation of vinylogous acyclic pronucleophiles, but a few examples with cyclic substrates have also been documented. To the best of our knowledge, no examples of indirect procedures were found.

#### 3.4.1 Direct Procedures

##### 3.4.1.1 Acyclic Pronucleophiles

A first group of contributions deal with the direct asymmetric γ-alkylation reaction of linear α,β-unsaturated aldehydes via dienamine, NHC, and/or metal catalysis, as outlined in Table 5.

**Table 5:**
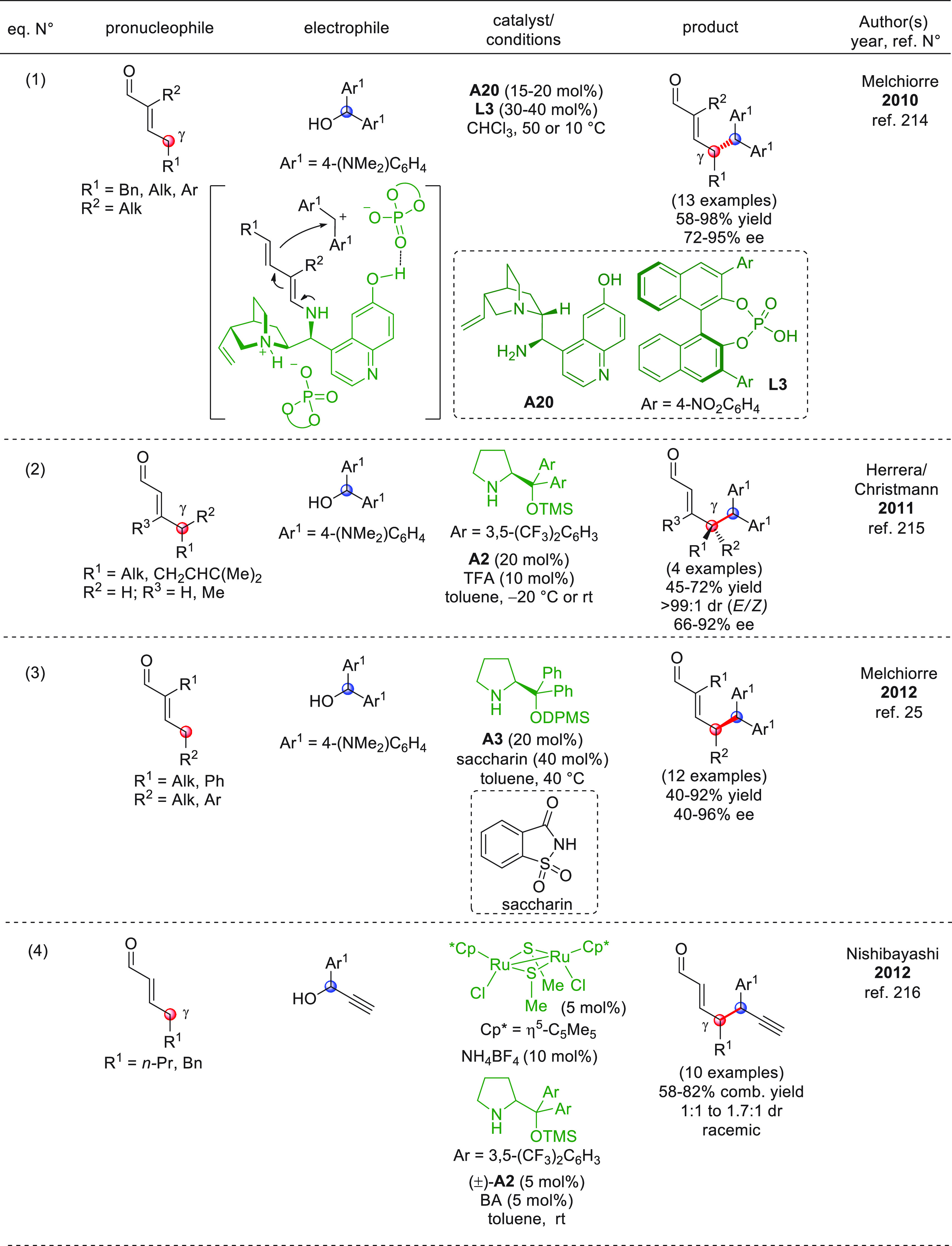

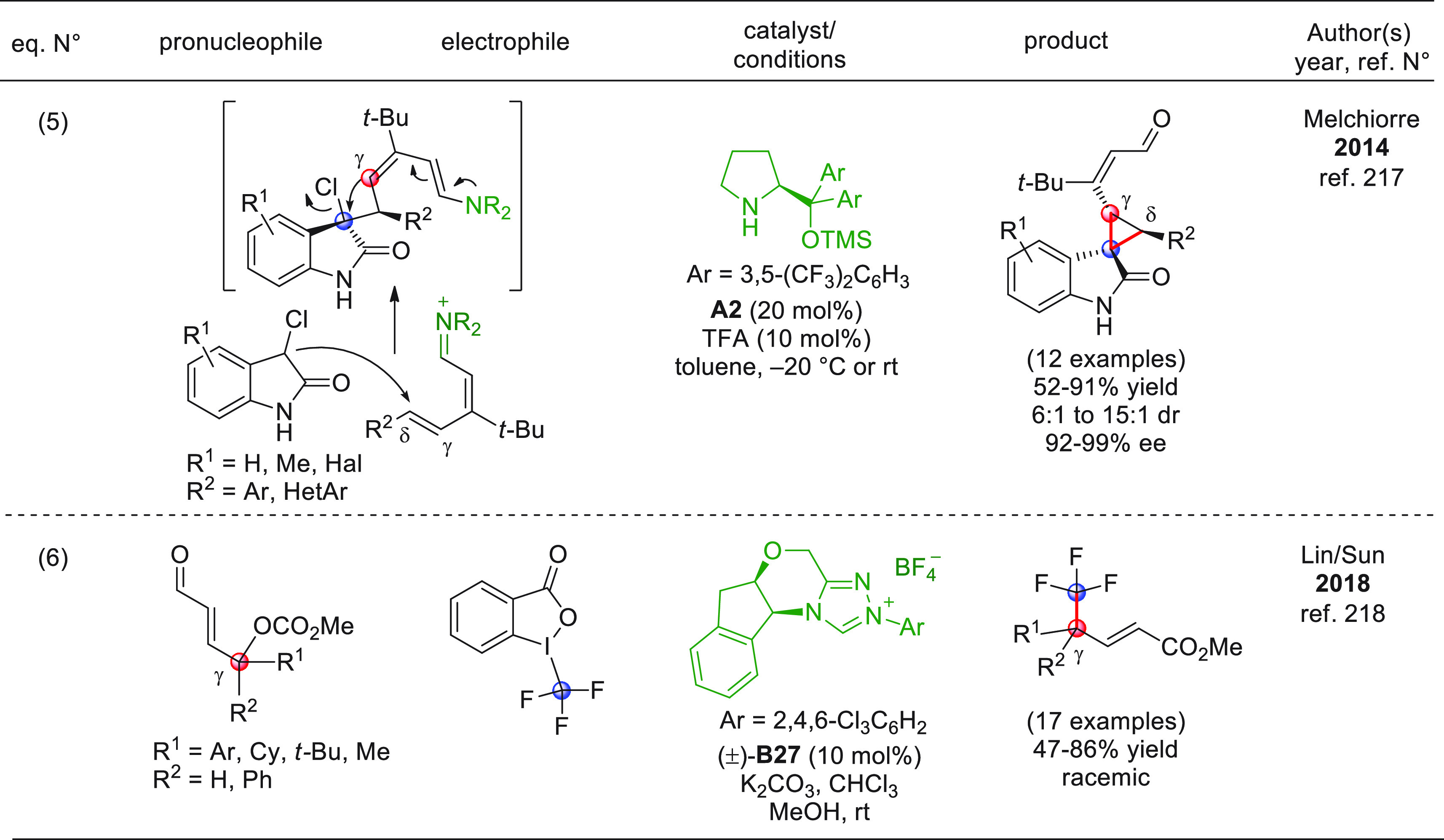
Remote Functionalization of Enals in Alkylation Reactions

Melchiorre et al. developed an asymmetric γ-alkylation process of α-substituted enals, by using bis(4-dimethylaminophenyl)methanol as the electrophilic source and exploiting the cooperative catalysis exerted by primary amine catalyst **A20** with phosphoric acid **L3** (Table 5, eq 1).^214^ Interception of the in situ generated benzhydryl carbocation under acidic conditions with dienamine intermediate from the pronucleophile gave the γ-alkylation products with generally good results (S_N_1 pathway). A tightly organized transition state was proposed (depicted in eq 1) where the chiral phosphate acts synergistically with the dienamine chiral inducer as a counterion for both the carbocationic electrophile and the protonated quinuclidine of the amine catalyst.

Soon after, Herrera, Christmann, et al.^215^ studied the same alkylation reaction starting from α-unsubstituted enals bearing diverse substitution patterns: linear unbranched and β-substituted enals favored the vinylogous alkylation path (Table 5, eq 2); γ,γ-disubstituted enals (R^1^, R^2^ ≠ H) privileged the nonvinylogous α-attack (not shown), and α-substituted enals did not work at all under these experimental conditions. Notably, the *E* vs *Z* diastereomeric ratio within the products was under thermodynamic control (exclusive *E* isomers), and the stereodefining step of the process was the final protonation of the dienamine-linked products before catalyst release by hydrolysis.

Similar γ-alkylation reactions of α-branched linear enals were carried out,^25^ using secondary amine catalyst **A3** and achiral saccharin as a Brønsted acid additive (Table 5, eq 3). Complete γ-regioselectivity and high enantioselectivities were attained in the corresponding products.

γ-Regioselective propargylation of enolizable α,β-unsaturated aldehydes was realized by Nishibayashi et al. using the cooperative catalyst system consisting of a thiolate-bridged diruthenium complex and racemic secondary amine (±)-**A2** (Table 5, eq 4).^216^ In the event, the corresponding propargyl allylated products were regioselectively obtained in moderate-to-high yields as mixtures of racemic diastereoisomers. It was proposed that the ruthenium-based catalyst activated the propargyl carbinol via the formation of an allenylidene complex (not shown), while the secondary amine would simultaneously activate the starting enal via dienamine formation. Though carried out in a racemic context, this is a quite rare and interesting example of the exploitation of cooperative transition metal- and organocatalysis in the vinylogous domain.

The asymmetric synthesis of cyclopropane spiroxindole was carried out by Melchiorre et al., featuring a highly regio- and stereoselective vinylogous organocatalytic cascade (Table 5, eq 5).^217^ This time, the dienal substrates initially act as iminium ion-activated vinylogous acceptors, which are attacked at their remotest δ-position by chlorooxindole, to furnish dienamine intermediates (between square brackets, eq 5). Intramolecular vinylogous S_N_2 of these dienamines then affords the targeted cyclopropanes with good efficiency and stereocontrol. Key to the success of the reaction was the positioning of the biasing bulky *t*-Bu group at the β-site of the starting dienals, allowing exclusive δ-regioselective 1,6-addition in the first step of the cascade.

The first example of NHC-catalyzed vinylogous trifluoromethylation reaction of α,β-unsaturated aldehydes was recently documented by Lin, Sun, and co-workers, as a general route for the remote installation of C(sp^3^)-CF_3_ bonds (Table 5, eq 6).^218^ Enals bearing a γ-leaving group were elected as NHC-activatable substrates (for the general γ-activation concept of enals under NHC catalysis, see Schemes 17 and 19 and related text), while the benziiodoxole Togni reagent was chosen as the electrophilic trifluoromethyl source. Using racemic indane-based triazolium salt precatalyst (±)-**B27**, potassium carbonate, and methanol, the efficient formation of racemic trifluoromethylated methyl ester products was obtained, with exclusive γ-regioselectivity. Control experiments supported the notion that the NHC-bound vinylogous enolate intermediate served as the actual nucleophilic species. The strict vinylogous regioselectivity could be explained by DFT calculations, indicating that the γ-pathway is both kinetically and thermodynamically favored compared to the α-pathway. In fact, beneficial electrostatic interactions were thought to be present in the transition state leading to the γ-products, involving the hypervalent iodine moiety of the trifluoromethylating reagent and the indane NHC motif; in the α-pathway, these favorable interactions would be disrupted.

In the previous sections, we discussed the prolific chemistry emerging from the (often formal) [4 + 2] oxa- and aza-Diels–Alder reactions between HOMO-raised dienamine- or trienamine-derived dienes and carbon-centered C=O or C=N dienophiles. Much rarer are examples of catalytic asymmetric thia-Diels–Alder cycloadditions, especially in the vinylogous realm. In 2014, Jørgensen et al. faced this challenge, using remotely enolizable dienals as the diene source via trienamine activation and various thioesters as the dienophile components (Table 6, eq 1).^219^ The corresponding ε,β-locked dihydrothiopyran cycloadducts were obtained with wide tolerance of substituents in both reaction partners. Of note, the regioselectivity of the reaction emphasized that the remote ε-site of the trienamine intermediate from the dienal attacked the sulfur atom and not the carbon atom of the thiocarbonyl function. To rationalize the reaction outcome, DFT calculations were performed, which pointed to a stepwise mechanism involving zwitterionic intermediates; electronic factors, particularly those exerted by the ester group adjacent to the thiocarbonyl acceptor, were considered responsible for the observed regioselectivity, while *endo/exo* diastereoselectivity is most likely to be kinetically controlled.

**Table 6:**
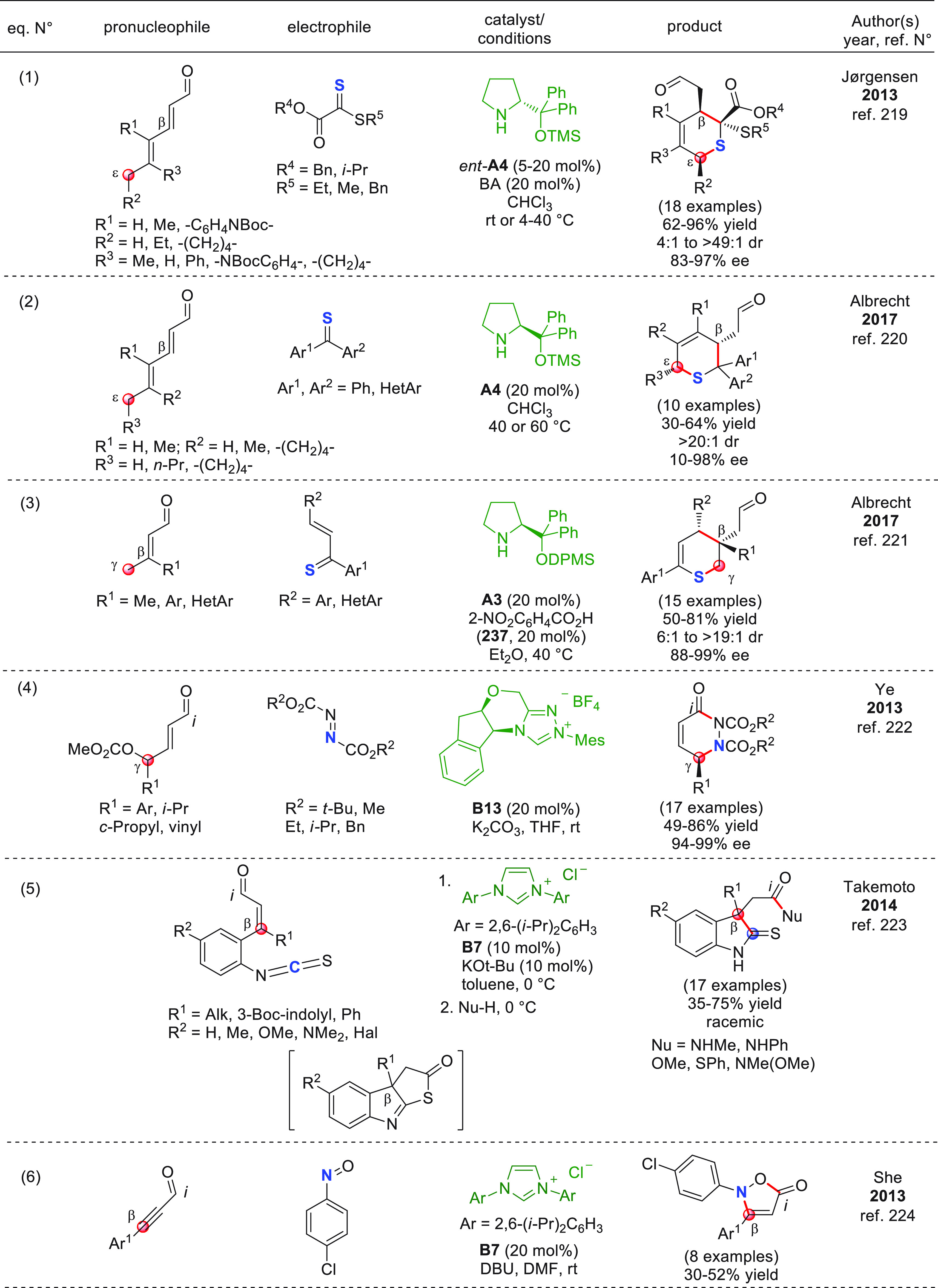

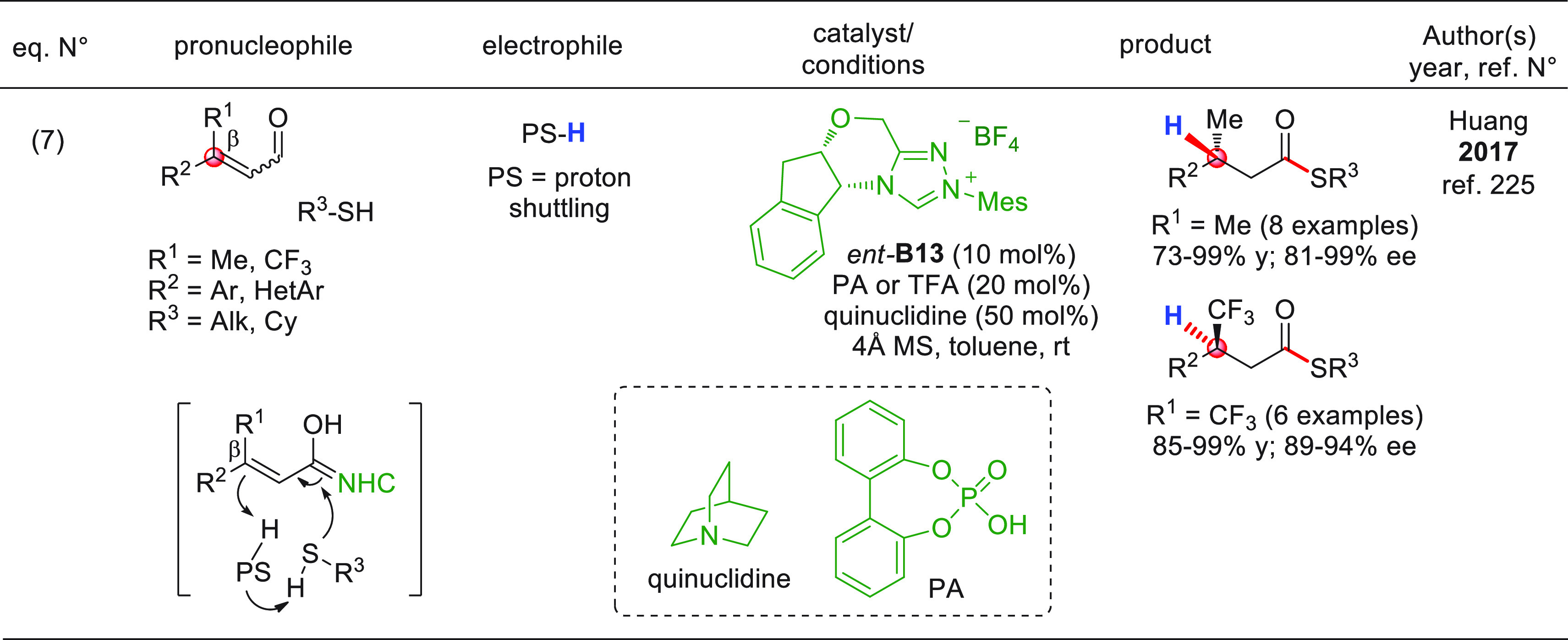
Remote Functionalization of (Poly)enals in Thia-Diels–Alder Cycloaddition, Amination, Nitrosation, Aziridination, and Protonation Reactions

Remotely enolizable dienals served also as 4π-components in ε,β-regioselective thia-Diels–Alder reactions with thioketone heterodienophiles (Table 6, eq 2).^220^ The aminocatalytic trienamine-mediated cyclization provided 5,6-dihydro-*2H*-thiopyranes, probably via either concerted asynchronous [4 + 2] cycloaddition or nonorthodox diradical process, depending upon the substituent attached to the C=S bond (aryl vs heteroaryl).

An *ortho*-regioselective IED-HDA reaction was explored by Albrecht et al. featuring regio- and stereoselective coupling between γ-enolizable enals, acting as electron-rich dienophiles, and thiochalcones, acting as heterodienes (Table 6, eq 3).^221^ The aminocatalytic [4 + 2] cycloaddition resulted in the formation of enantioenriched 3,4-dihydro-*2H*-thiopyrans.

The NHC-catalyzed γ,*ipso*-regioselective [4 + 2] annulation between α,β-unsaturated aldehydes, bearing a γ-leaving group, and azodicarboxylate acceptors was reported by Ye et al., affording highly enantioenriched dihydropyridazinone products (Table 6, eq 4).^222^ A plausible mechanistic rationale was proposed, where the NHC-bound dienolate (derived from HOMO activation of the enal) attacks the azodicarboxylate, affording an acyl azolium adduct, which finally releases the catalyst and consigns the targeted compounds.

A tandem, NHC-catalyzed reaction was developed by Takemoto and co-workers, entailing the synthesis of racemic indoline thiones starting from β,β-disubstituted α,β-unsaturated aldehydes carrying an isothiocyanate moiety (Table 6, eq 5).^223^ Initially, covalent activation of the enal by the NHC catalyst gives a vinylogous Breslow intermediate, whose β-vinylogous carbon site intramolecularly attacks the isothiocyanate to furnish a thienoindolone structure (between brackets). Subsequent nucleophilic opening of this intermediate provides the targeted products.

NHC-catalyzed formal [3 + 2] annulation involving alkynyl aldehydes and nitrosobenzenes was reported by She and collaborators in 2013 (Table 6, eq 6).^224^ Reaction conditions were found to regioselectively steer the reaction path either along a vinylogous β,*ipso*-annulation to give isoxazol-5(2*H*)ones (eq 6) or along nonvinylogous *ipso*,β-annulation to give regioisomeric isoxazol-3(2*H*)ones (not shown) (for general β,*ipso* NHC-catalyzed annulations, see also Scheme 14).

The β-nucleophilic activation of α,β-unsaturated aldehydes via NHC-catalyzed homoenolate formation was also cleverly exploited by Huang and collaborators in a quite rare example of enantioselective vinylogous protonation of enals in the absence of any directing groups (Table 6, eq 7).^225−227^ The careful choice of a Brønsted base (a chiral bridgehead tertiary amine such as quinuclidine) together with a strong Brønsted acid cocatalyst (phosphoric acid PA or TFA) provided a smart proton-shuttle system (the quinuclidinium ion, PS-H in the table, eq 7) capable of ensuring β-protonation of the NHC-bound homoenolate in a highly enantioselective manner. Since no match/mismatch was observed when using chiral quinine or quinidine with chiral NHC, it was proposed that the chiral influence came mainly from the NHC moiety, while the effect of the quinuclidine is mostly steric. Thus, using various thiol nucleophiles, the corresponding saturated thioesters with a β-stereocenter were prepared, in very good yields and enantioselectivities.

##### 3.4.1.2 Cyclic Pronucleophiles

**Scheme 66:**
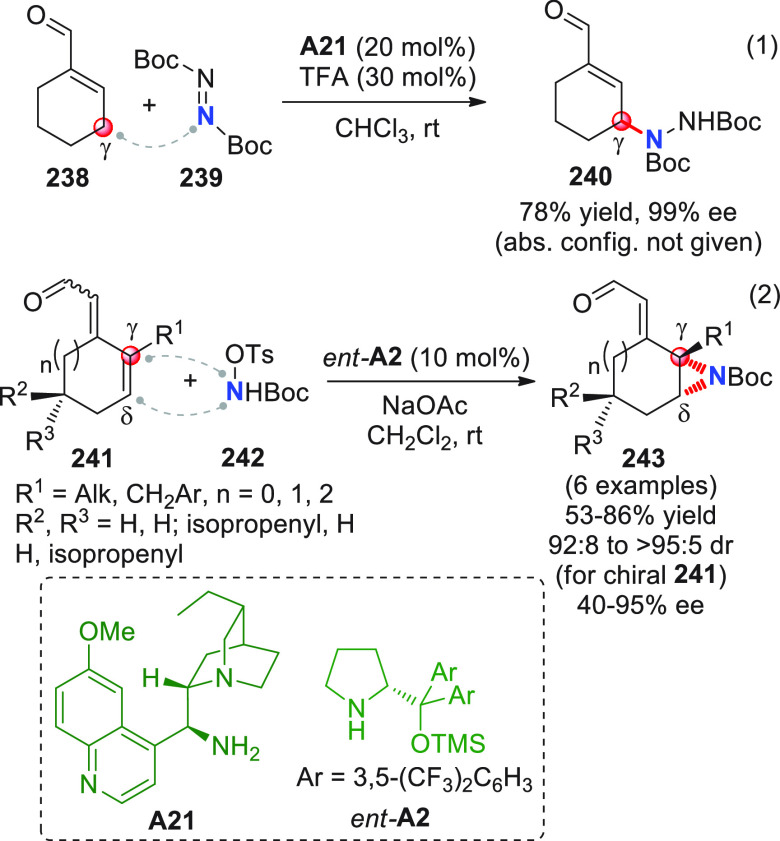


During a broad, brilliant study focusing mainly on the asymmetric dienamine-catalyzed vinylogous Michael addition of cyclic enones to nitroalkenes (see infra, section on ketone pronucleophiles), a brief exploration was also dedicated to the asymmetric vinylogous amination of a cyclic enal (Scheme 66, eq 1).^228^

Thus, using primary amine catalyst **A21**, the reaction between **238** and *tert-*butylazodicarboxylate **239** proceeded smoothly, giving aminated product **240** with exclusive γ-regioselectivity and excellent enantioselectivity.

An iminium ion-dienamine catalytic cascade was fruitfully exploited by Jørgensen et al. in a rare example of asymmetric remote aziridination reaction (Scheme 66, eq 2).^229^ Cyclic 2,4-dienals having diverse aliphatic substituents in the γ-position (R^1^ had to be strictly different from H) were treated with **242** using secondary amine *ent*-**A2** catalyst, furnishing the corresponding aziridines **243** with exclusive δ,γ-regioselectivity (>95:5 δ,γ: β,α), optimal *E*:*Z* geometric selectivity (>95:5 *E*:*Z*), and moderate-to-good enantioselectivities. The reaction mechanism features a δ-selective hypervinylogous 1,6-Michael addition of the nitrogen nucleophile **242** onto the iminium ion from **241**, followed by S_N_2 closure of the formed γ-dienamine into the nitrogen atom favored by the OTs leaving group.

**Scheme 67:**
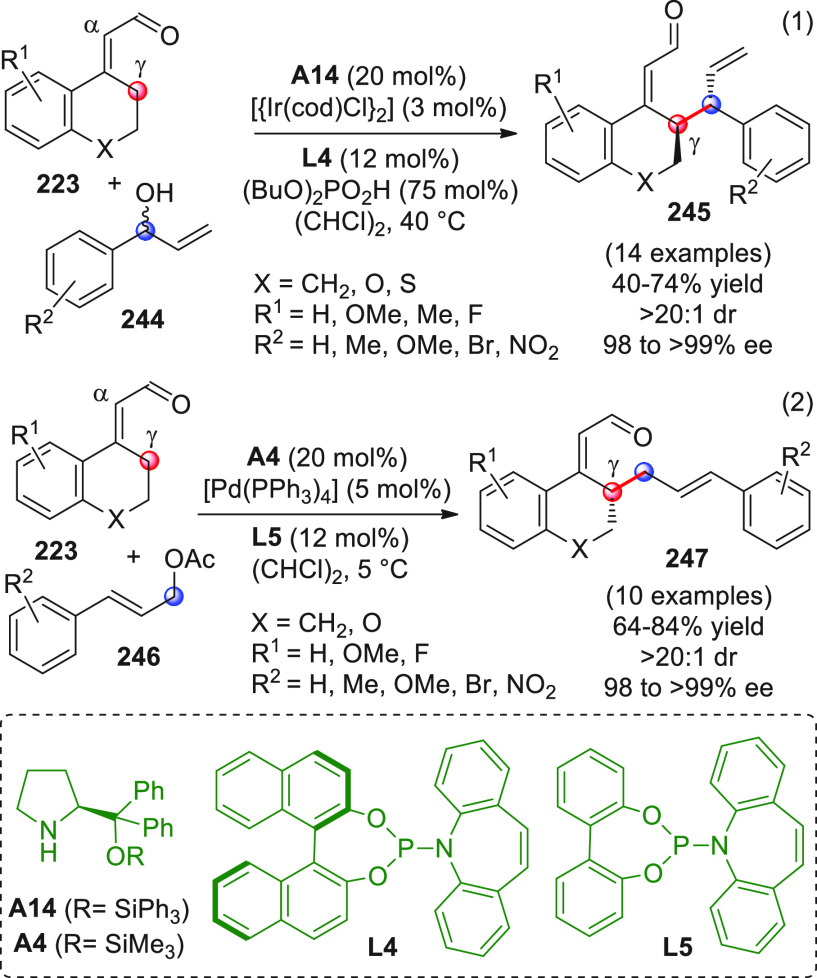


The Jørgensen group also developed asymmetric γ-allylation reactions of cycloalkylidene acetaldehydes by merging vinylogous aminocatalysis with transition metal catalysis (Scheme 67).^230,231^ The direct asymmetric allylation of enals of type **223** could pose, in principle, a series of challenges including regioselectivity issues of both the donor (α vs γ) and acceptor (branched, b vs linear, l) components, as well as stereoselectivity concerns, i.e. *E* vs *Z* olefin geometry, *syn* vs *anti* diastereoselectivity, and enantioselectivity within the products. The wise orchestration of both aminocatalytic activation of the pronucleophile **223** and electrophilic activation of the π-allyl system within either **244** or **246** allowed for the regio- and stereoselective entry to either branched allyl products **245** (eq 1) or linear allyl products **247** (eq 2).

Treatment of variously substituted enals **223** with allyl benzyl alcohols **244** using the combination of triphenylsilyl-protected prolinol **A14** (providing dienamine activation of **223**) and iridium-based catalyst having phosphoramidite ligand **L4** produced the branched γ-allyl products **245** in good yields and with excellent levels of regioselectivity (>20:1 γ/α; >20:1 b/l) as well as stereoselectivity (>20:1 *E/Z*) (eq 1). By switching, instead, to palladium-based catalyst with achiral ligand **L5** and using TMS-protected l-prolinol **A4**, enals **223** coupled to allylic acetates **246** to furnish linear vinylogous γ-allyl products **247** (eq 2), again with admirable levels of site- and stereoselectivity (>20:1 γ/α; >20:1 l/b; >20:1 *E/Z*). Of note, control experiments revealed that the branched vs linear regioselectivity exclusively depended upon the metal catalyst of choice and not on the nature of the allylic alcohol in use. By employing *ent*-**A14** instead of **A14**, *syn*-configured products were formed (not shown), thus demonstrating the possibility to selectively synthesize all six isomers of vinylogous allylated products.

**Scheme 68:**
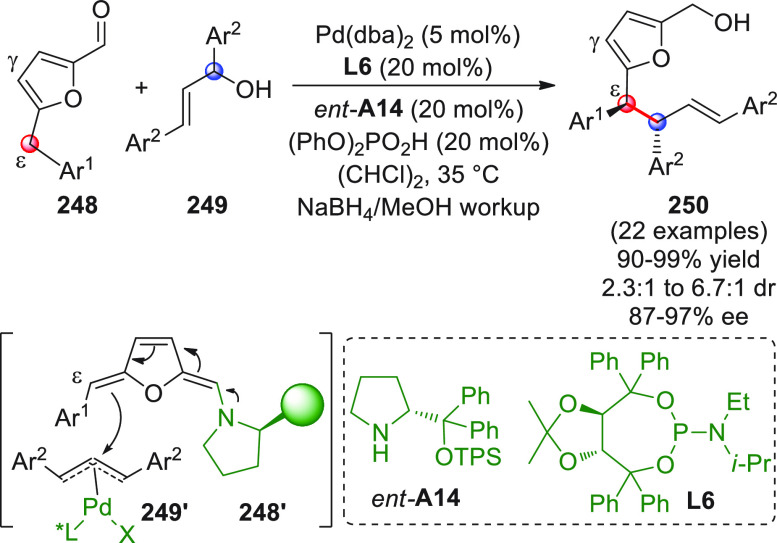


Conceptually similar synergistic combination of metal- and organocatalysis was exploited by Gong and collaborators in the hypervinylogous, ε-regioselective asymmetric allylation of furfural derivatives (Scheme 68).^232^ The focal idea was that the remote ε-site of the furfural substrates **248** would be activated via trienamine catalysis, and the palladium-ligand complex would concomitantly activate the allylic alcohols **249**. Thus, bulky secondary amine *ent*-**A14** together with palladium(II) complex using TADDOL-based phosphoramidite ligand **L6** and a phosphoric acid additive turned out to be the best “cocktail” for promoting the ε-allylation reaction between **248** and **249** and for allowing the preparation of products **250** in high yields and very good stereocontrol. Nonbenzylic ε-alkyl substituted furfurals were also examined (not shown), but they gave inferior results in terms of reaction efficiency. The use of *ent*-**L4** (under otherwise identical conditions) led to the enantiomeric products with lower stereocontrol, and this suggested that stereochemical induction was mainly dependent on the configuration of the chiral ligand of palladium, while the chiral amine catalyst played a role of assistance in stereochemical control.

## 4 Vinylogous Ketones

In comparison to the high number of documents involving catalytic γ-selective (or β-, ε-, etc.) activation of enals, reviewed in the previous section, the corresponding reactions embracing enones as γ-selective pronucleophiles are definitely encountered to a lesser extent. Controlling the γ-regioselectivity of reactions with vinyl ketones as pronucleophiles is not straightforward, because of the low electron density at the γ-position^233^ of the corresponding dienolates, which tends to favor nonvinylogous α-selective reactions^234−236^ (α vs γ regioselectivity). Moreover, ketones bearing an α′ enolizable site possess an additional pronucleophilic position,^237,238^ and this makes the regiocontrol of the reaction (γ vs α′) particularly challenging.

**Figure 2:**
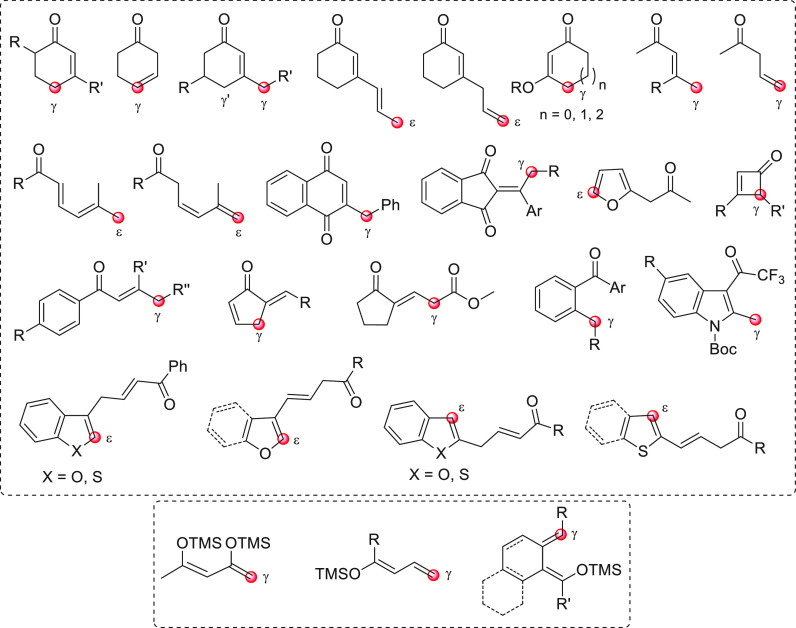
Collection of pronucleophilic ketones (above) at work in this chapter using the direct procedures. Below, the nucleophilic ketone-derived silyl dienol ethers used in indirect procedures. Red circles denote the reactive (pro)nucleophilic carbon sites.

In these years, different strategies have been adopted to overcome these issues, for example by using cyclic enones, using deconjugated ketones, or even placing a bulky group at the nonvinylogous α position, with the aim to apply to vinylogous ketones the catalytic enantioselective strategies developed for vinylogous aldehydes (Figure 2).

A limited number of examples dealing with vinylogous ketones as donors in addition to C=O and C=N bonds were reported in the last 8 years; in fact, most of the studies concern the addition to activated C=C bonds, often followed by cyclization reactions. The interest was focused on developing stereoselective procedures, and organocatalysis was the main activation strategy of pronucleophilic vinylogous ketones, often assuring high levels of diastereo- and enantioselectivity. However, even if outstanding results were reached in recent past years, the use of these vinylogous procedures in the synthesis of target molecules is still limited, and none of the reported examples utilizes enantioselective organocatalytic activation modalities.

### 4.1 Additions to C=O Bonds

#### 4.1.1 Direct Procedures

##### 4.1.1.1 Acyclic Pronucleophiles

**Scheme 69:**
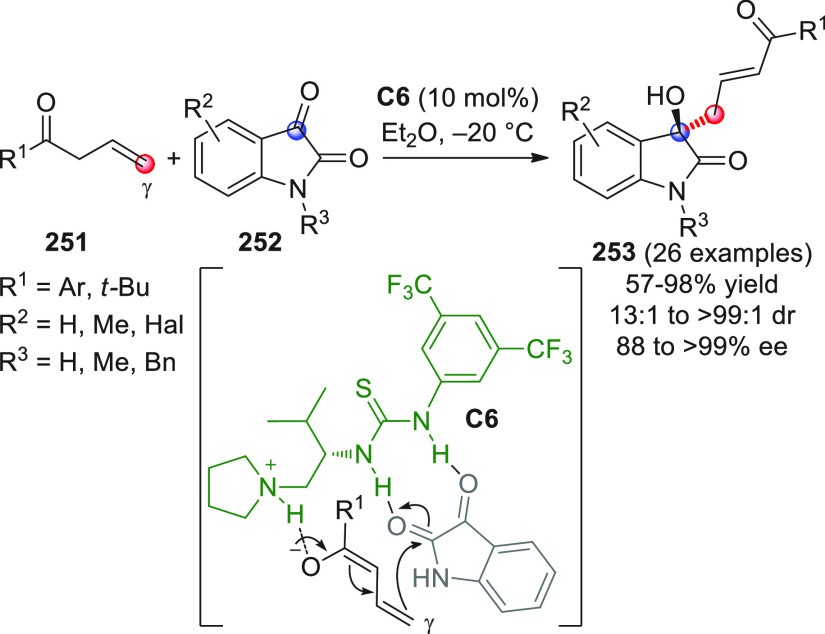


In 2013 Jiang and co-workers, inspired by previous work of Shibasaki,^239−241^ where acyclic allyl cyanides were used as donors in direct asymmetric vinylogous aldol reactions, developed the first example of application of allyl ketones in catalytic asymmetric reactions.^242^ The enantioselective direct vinylogous aldol reaction between allyl ketones **251** and isatins **252** (Scheme 69) was catalyzed by the l-valine-derived bifunctional tertiary amine/thiourea catalyst (**C6**), that is supposed to first deprotonate the allyl ketone at the α position and then bring the resulting enolate and the isatin electrophile together to form the hydrogen-bonded complex for the C–C bond formation. The reaction is highly enantio- and *E*-selective, and the best reaction outcome was achieved with unprotected isatins. Good stereocontrol was maintained even when the reaction was performed on a gram scale. The products are valuable *R*-configured 3-hydroxy-2-oxindole derivatives of type **253**. Computational studies, based on the density-functional theory (DFT), strongly supported that the observed stereochemistry, and preference for γ- over α-alkylation, is the result of favorable secondary π–π* and H-bonding interactions in the transition state.

**Scheme 70:**
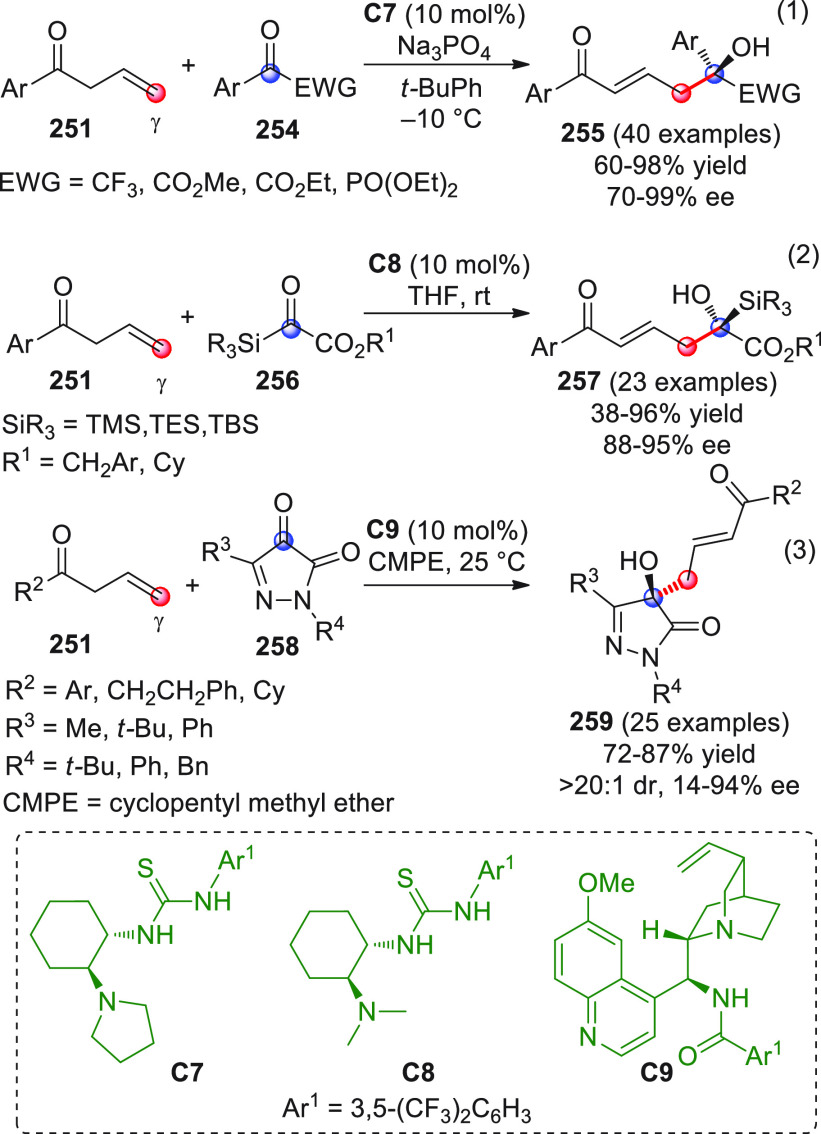


Subsequent to this report and because of their easy accessibility, allyl ketones were also employed as vinylogous pronucleophiles in addition reactions to C=C (vide infra), C=N (vide infra), and C=O. In 2016, the Jiang group reported the first catalytic, asymmetric vinylogous aldol reaction (VAR) of allyl aryl ketones **251** to activated acyclic compounds **254** (Scheme 70, eq 1).^243^ The screening of four bifunctional tertiary amine/thiourea catalysts revealed that **C7** (10 mol %) was the most efficient agent and that the addition of basic Na_3_PO_4_ (2 equiv) improved both yields and enantioselectivity. These conditions were applicable to diverse activated acyclic ketones with electron-withdrawing groups, including trifluoromethyl ketones, α-ketoesters, and α-keto phosphonates, thus furnishing compounds **255** in good yields and enantioselectivities. The authors showed how, starting from compounds **255**, it was possible to prepare, by suitable transformations, chiral EWG-(α,δ)-tertiary hydroxy-based carboxylic acids, key structural motifs in important bioactive molecules.

Two more works, where allyl ketones were used as donor components in direct VARs, were published in 2018. In the first one, Wang and co-workers reported the addition of **251** to silyl glyoxylates **256** (Scheme 70, eq 2),^244^ opening a new route for the enantioselective preparation of α-hydroxysilanes **257**. The reaction was organocatalyzed by the bifunctional catalyst **C8**, which may activate the carbonyl of **256** via strong hydrogen bonding. The steric hindrance of the silicon moiety in the acylsilane favors the attack of the vinylogous donor at the less bulky γ-position (γ-selectivity). Moreover, the reaction conditions avoided possible [1,2]-Brook rearrangement (the silyl migration from carbon to oxygen) and ensured the formation of the desired products **257** with moderate to good yields and good enantioselectivity levels. A mechanism based on DFT calculations was proposed. Interestingly, the authors verified that using the conjugated ketone 1-phenylbut-2-en-1-one as a donor, only a trace amount of the product was formed, emphasizing the importance of deconjugated allyl ketones as vinylogous precursors.

In the second study, Mukherjee and Ray reported the first example of the use of pyrazole-4,5-diones **258** as acceptors in vinylogous reactions (Scheme 70, eq 3).^245^ The VAR of **251** was catalyzed by the quinine-derived bifunctional tertiary amine/amide catalyst **C9**, a single H-donor catalyst. The reaction proceeded exclusively in γ- and *E*-selective manner to give products **259** in good yields, but with moderate to discrete enantioselectivities. The lowest enantioselections were registered with allyl ketones **251** bearing *ortho*-substituted aryl substituents, probably because of the steric hindrance of the substituent. Two examples of alkyl allyl ketones as donors were also reported, furnishing the products in good yields albeit with a moderate enantiomeric excess.

##### 4.1.1.2 Cyclic Pronucleophiles

**Scheme 71:**
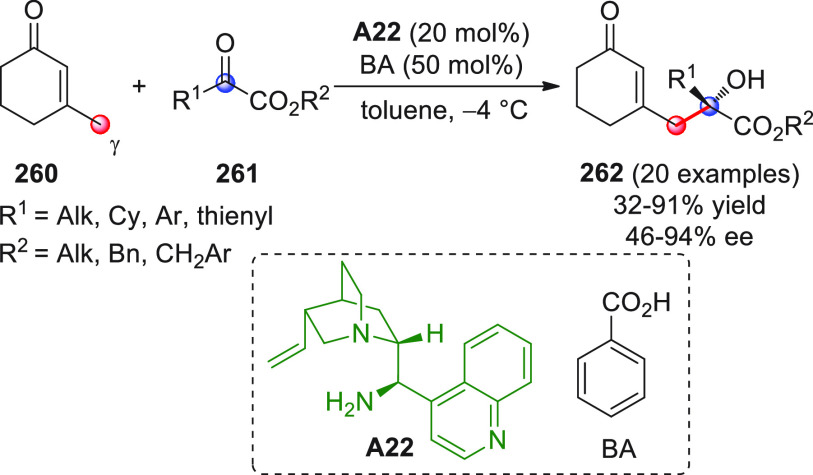


In 2013, the Melchiorre group reported the direct VAR of 3-methyl 2-cyclohexen-1-one (**260**) with α-keto esters **261** to furnish the aldol adducts **262** (Scheme 71).^246^ The reaction was catalyzed by the bifunctional primary amine-thiourea **A22**, which could simultaneously activate both the enone, by forming a nucleophilic dienamine, and the electrophilic acceptor by an H-bond-directing activation. This dual activation strategy, with the presence of benzoic acid, secured the access to aldol products **262** with good stereocontrol and perfect γ-site selectivity.

If a wide variability of R^1^ and R^2^ of the α-keto ester was well tolerated, the limitation of the system was the enone structure; in fact, with 3-methyl 2-cyclopenten-1-one, a complete loss of reactivity was registered.

**Scheme 72:**
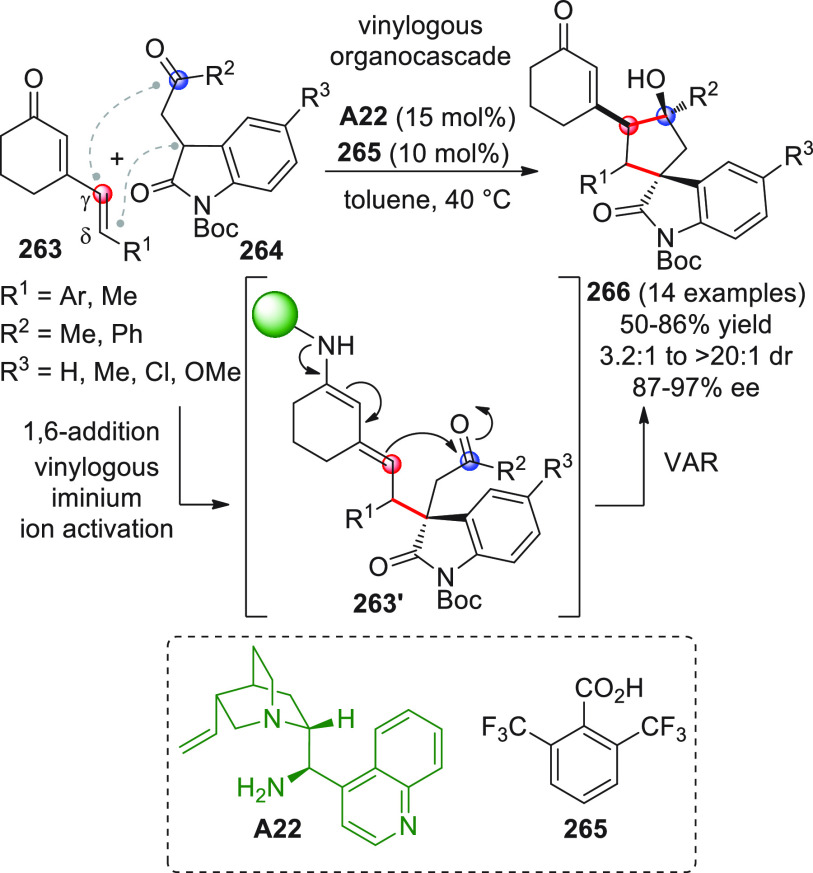


The same year, Melchiorre et al. reported the first example of a vinylogous organocascade catalysis, where a 1,6- addition/aldol sequence between cyclic dienones **263** and oxindoles **264** carried to the formation of spirocyclopentane oxindoles **266** bearing four contiguous stereocenters.^247^ The bifunctional primary amine–thiourea catalyst **A22**, in the presence of 2,6-bis(trifluoromethyl)benzoic acid **265** as cocatalyst, operates through a dual activation strategy; in fact, it activates the dienone as iminium ion that reacts at first as vinylogous electrophile to give **263′** (Scheme 72). Then, dienamine activation in **257′** allows the intramolecular VAR that eventually carries to the spirocyclic product.

**Scheme 73:**
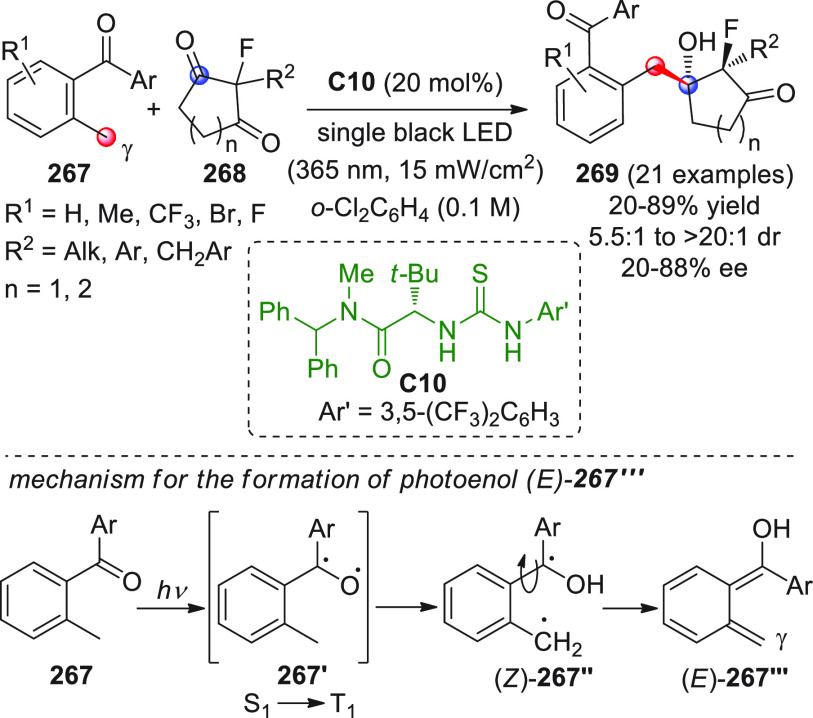


The last example of a direct cyclic VAR, again from the Melchiorre group, is the recent application of photoactivation of 2-alkyl benzophenone substrates **267** to give highly reactive hydroxy-*o*-quinodimethanes (*E*)-**267′′′**, that react with 2-substituted-2-fluorocyclopentane-1,3-diketones **268**.^248^ The photoenol generation was obtained by irradiation at λ = 365 nm. The mechanism of formation of the reactive photoenol (*E*)-**267′′′** was already in depth studied,^249^ and it is reported in Scheme 73. Irradiation of 2-alkyl benzophenone **267** triggers the formation of a singlet excited state S_1_**-267′** that, upon intersystem crossing, decays to a triplet state T_1_**-267′**. The 1,5-hydrogen transfer generates the diradical intermediate (*Z*)-**267′′**, which then undergoes rotation to give the reactive enol (*E*)-**267′′′**. The aldol acceptor was an achiral 1,3-diketone of type **268**, and good stereocontrol was achieved with the chiral amido–thiourea catalyst **C10**, which is able to activate one of the enantiotopic carbonyl groups. Overall, the process is a rare example of a light-driven organocatalytic aldol desymmetrization in which two stereocenters are simultaneously generated, one of them being a fluorine-containing quaternary stereocenter.

**Scheme 74:**
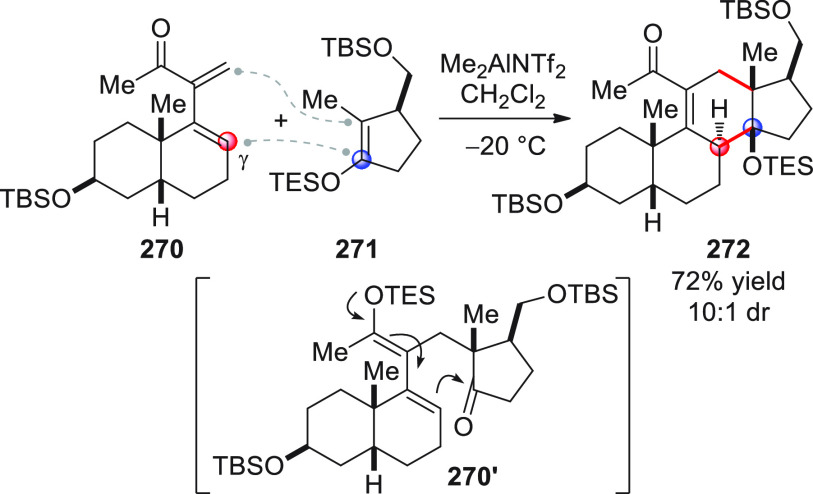


In order to assemble the tetracyclic core of rhodexin A, Jung and Guzaev developed a formal inverse electron demand Diels–Alder reaction (IEDDA) between hindered dienone **270** and silyl enol ether **271**, catalyzed by the strong Lewis acid aluminum triflimide complex, Me_2_AlNTf_2_ (Scheme 74).^250^ The authors sustained that the overall [4 + 2] process consists of a stepwise cascade with an initial Mukaiyama Michael reaction, followed by a vinylogous Mukaiyama aldol reaction, which would proceed through the intermediacy of in situ-formed silyl dienol ether **270′**.

#### 4.1.2 Indirect Procedures

##### 4.1.2.1 Acyclic Nucleophiles

**Scheme 75:**
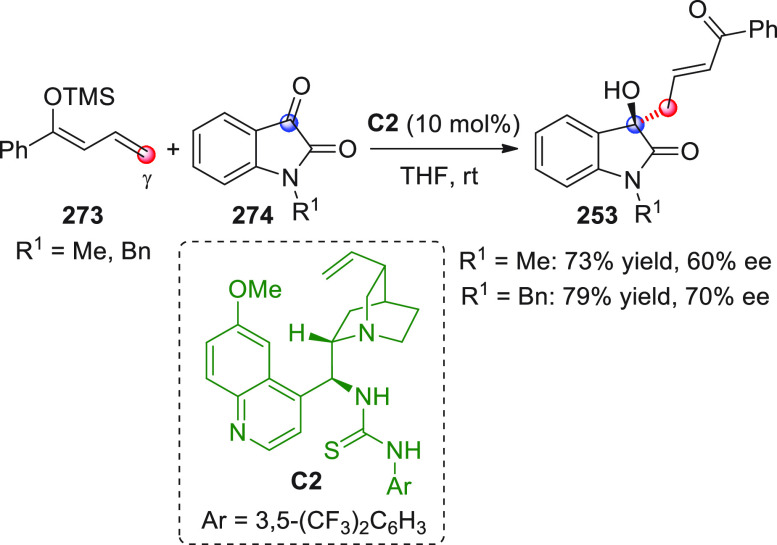


Indirect procedures, in which the vinylogous enolates of ketones are preformed as stable silyl derivatives, are almost absent in the literature of this period, probably for reasons of atom economy and the desire to develop simple and expedient synthetic procedures. A sole example was reported by the Alemán group in 2018, dealing with an enantioselective organocatalytic vinylogous Mukaiyama aldol reaction (VMAR) of silyloxy dienes and isatins (Scheme 75).^88^ The reaction was catalyzed by the bifunctional organocatalyst **C2** and gave 3-hydroxy-2-oxindole derivatives **253**. Most of the silyloxy dienes used in the paper derived from aldehydes, and the work has already been reviewed in section 3.1.2.1 (Scheme 28). However, two examples of the panel presented by Alemán are ketone-derived silyloxy dienes (**273**, Scheme 75). Interestingly, the reaction is the indirect version of the direct one presented in Scheme 69, in which Huang and collaborators used allyl ketones as pronucleophiles. The direct methodology afforded compounds **253** definitely in higher yields and enantioselectivity (86% versus 73% yield, 92% versus 60% ee for R = Me; 93% versus 79% yield, 98% versus 70% ee for R = Bn). Moreover, in the indirect procedure, the stereocontrol with *N*-unprotected isatins was modest and the methodology was developed with *N*-substituted isatins, while in the direct procedure the best stereocontrol was registered with *N*-unprotected isatins.

##### 4.1.2.2 Cyclic Nucleophiles

**Scheme 76:**
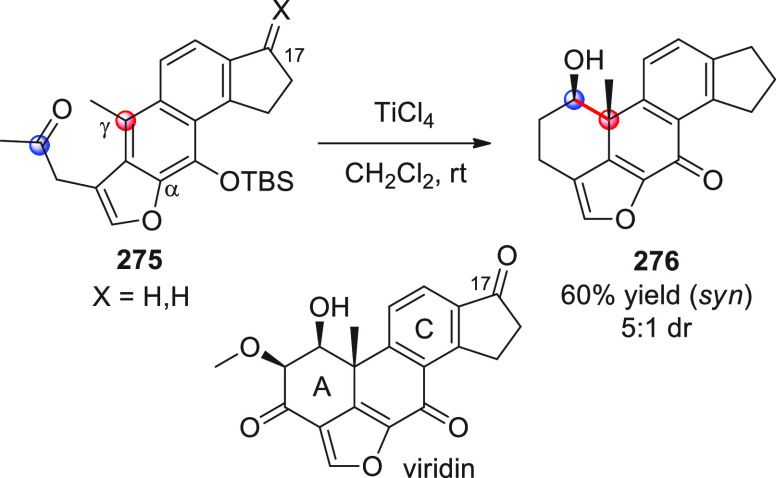


The sole example of indirect vinylogous reaction between a silyloxy diene derived from a “special” cyclic ketone (namely, silyl phenol **275**) and a carbonyl group was reported by Onyango and Jacobi,^251^ where the intramolecular vinylogous Mukaiyama aldol-type reaction was the key step in the synthesis of the core structure of viridin, a furanosteroid able to inhibit phosphatidylinositol-3-kinase (Scheme 76).

The reaction was catalyzed by TiCl_4_ and furnished the *syn*-adduct **276** preferentially, with dearomatization of the reactive silyl phenol ring. The authors found out that the substituents at C17 have a crucial impact on the cyclization. In fact, only **275**, where C17 is a CH_2_, underwent cyclization, whereas the analogous precursors with X = O(CH_2_)_2_O, H and OH, or H and OAc, did not afford any cyclized products.

### 4.2 Additions to C=N Bonds

#### 4.2.1 Direct Procedures

##### 4.2.1.1 Cyclic Pronucleophiles

**Scheme 77:**
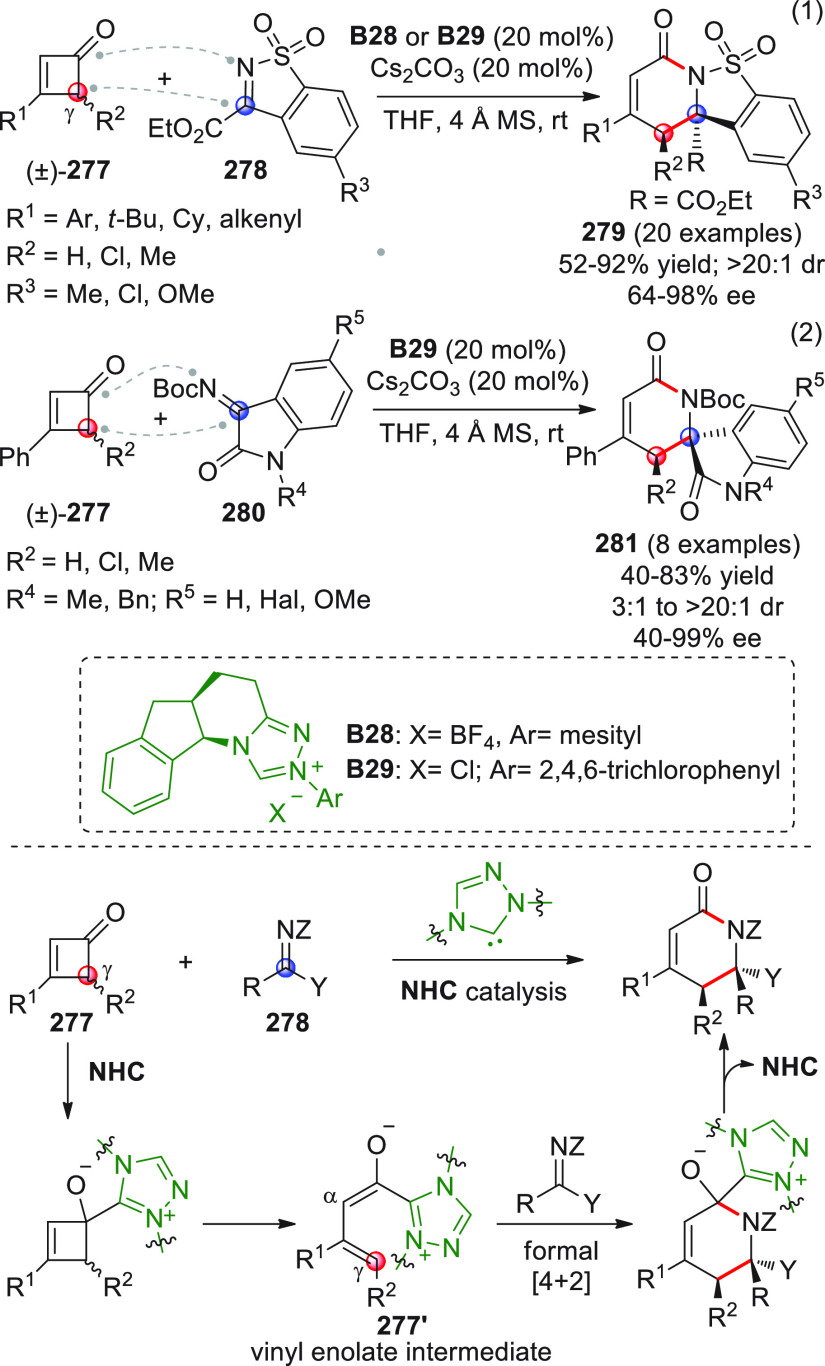


In 2015, Chi and collaborators described an organocatalytic activation of C–C bonds through the addition of an *N*-heterocyclic carbene (NHC) catalyst to cyclobutenones of type **277**.^252^ The key step is the C–C single bond cleavage, and the generation of an NHC-bound intermediate of type **277′**, a vinyl enolate, that reacts in a chemo- and stereoselective manner with an imine in a formal [4 + 2] cyclization, to form lactam products with two contiguous stereogenic centers. The reaction was reported with both sulfonyl imines (**278**, Scheme 77, eq 1) and isatin imines (**280**, Scheme 77, eq 2), while other imines, such as *N*-tosyl imine derived from benzaldehyde or aryl trifluoroacetone, did not lead to any product formation. In this strategy, all atoms of the substrates end up in the products, fulfilling the atom economy principle, and the overall reaction is redox-neutral. The reaction between **277** and **278** provided lactams **279** after 72 h in moderate to good yields, with excellent diastereoselectivities and good to excellent enantioselectivities. The stereoselectivity of the reaction was ensured by the use of aminoindanol-derived triazolium salts **B28** or **B29**. The authors disclosed how the electronic properties of both the imine substrates and NHC catalysts significantly affected the enantioselectivity of the reaction. Lactams **281** derived from isatin imines were generally obtained with lower diastereoselectivities but with high ee, apart from the case when an electron-donating substituent (R^5^ = OMe) was present on the isatin aromatic ring.

**Scheme 78:**
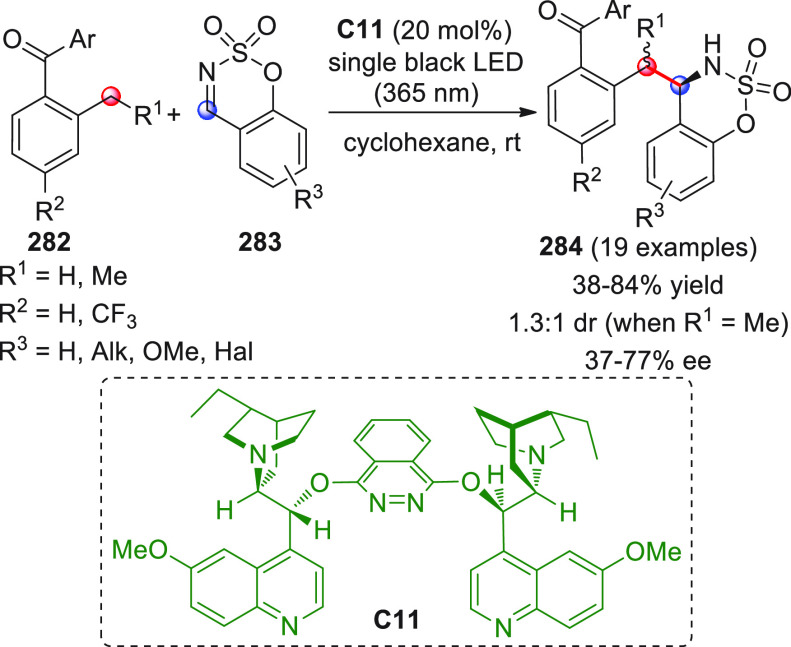


In 2016, the Melchiorre group developed a photochemical organocatalytic strategy for the direct enantioselective Mannich-type reaction of 2-alkylbenzophenones **282** and cyclic imines **283**, affording enantioenriched chiral sulfamates **284** (Scheme 78).^253^ Light irradiation (λ = 365 nm) generates the transient hydroxy-*o*-quinodimethane (see Scheme 73 for the discussion of the mechanism), that can be trapped by an imine acceptor. The reaction did not work with linear imines. A wide screening of diverse sets of organocatalysts revealed that the best stereoselection was achieved using dimeric cinchona alkaloid derivative **C11** in an apolar solvent such as cyclohexane. The authors demonstrated that the catalyst controls the stereochemical outcome of the reaction by solely interacting with the imine substrate **283** and not with the donor. However, the stereocontrol was moderate, revealing the overall difficulty of developing an efficient asymmetric organocatalytic procedure, due to the high reactivity and fleeting nature of the photoenol species.

#### 4.2.2 Indirect Procedures

##### 4.2.2.1 Acyclic Nucleophiles

**Scheme 79:**
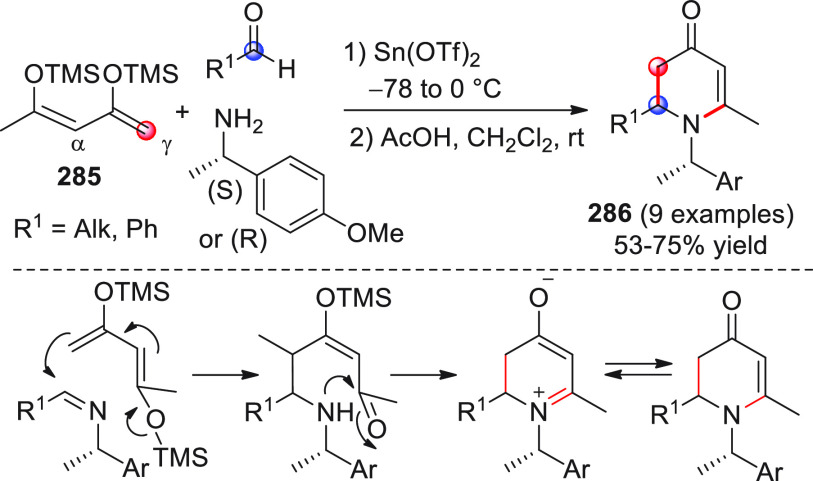


In 2015, Yang et al. reported for the first time the use of 1,3-bis-trimethylsilyl enol ether **285** as a vinylogous nucleophile in a Mannich-type reaction.^254^ They developed a stereoselective three-component vinylogous Mannich-type reaction between **285** and in situ-formed aldimines to access chiral 2,3-dihydropyridinones of type **286** (Scheme 79). The reaction was catalyzed by Sn(OTf)_2_, and the stereoselectivity was ensured by the use of the chiral α-methyl benzylamine substrate; after manipulation of the product, the auxiliary group could be removed by TFA treatment or hydrogenolysis. The authors exampled the utility of this methodology by preparing, from cyclic adducts **286**, some bioactive natural alkaloids which incorporate *cis*-2,6-dialkylpiperidine as the core structure. Beyond the synthesis of simple piperidine compounds, the method also provided a rapid route for chiral quinolizidine construction.

### 4.3 Conjugate Additions to Electron-Poor C=C Bonds

#### 4.3.1 Direct Procedures

##### 4.3.1.1 Acyclic Pronucleophiles

**Scheme 80:**
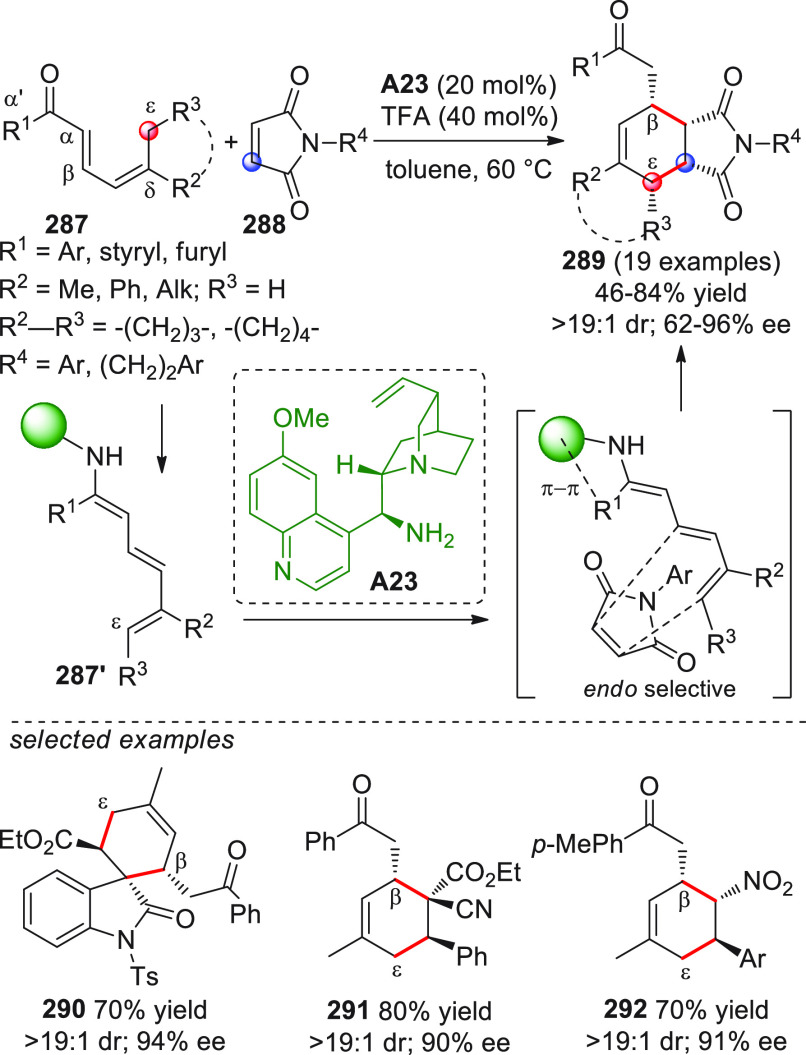


In 2012, the Chen group first applied the trienamine catalysis strategy, already developed for 2,4-dienals (see section 3.3.1.1),^161^ to 2,4-dienones in order to generate a chiral electron-rich triene system in situ, which could perform as a diene component in Diels–Alder (DA) reactions with electron-deficient dienophiles.^255^ The possibility of applying this procedure was limited to δ,δ-disubstituted 2,4-dienones, and the presence of an aryl (or 2-styryl) group at the α position was required to suppress the formation of an alternative, unreactive trienamine intermediate. Apart from these limitations, the authors developed a straightforward asymmetric DA cycloaddition reaction, catalyzed by the primary amine 9-amino-9-deoxyepiquinine **A23** and trifluoroacetic acid, in which the triene system **287′**, generated from 2,4-dienones **287**, reacted with dienophiles **288** with exclusive ε,β-regioselectivity (Scheme 80). Cycloadducts **289** were isolated as single *endo* diastereomers and with excellent enantioselectivity.

The authors did not investigate whether the mechanism was concerted or asynchronous, but they proposed a transition state to justify the observed stereoselection, in which a π–π interaction between the quinoline moiety of the catalyst and the arene ring (R^1^) of 2,4-dienone would block the *Re* face of the resulting trienamine intermediate. Then, an *endo*-selective cycloaddition would occur from the *Si* face of the triene system (Scheme 80). Finally, the generality of this methodology was demonstrated by applying it to other electron-deficient dienophiles, in particular to 3-allylideneoxindole, benzylidenecyanoacetate, and nitrostyrene acceptors to furnish multifunctional cyclohexene derivatives **290**, **291**, and **292**, respectively, with very good enantioselectivities.

**Scheme 81:**
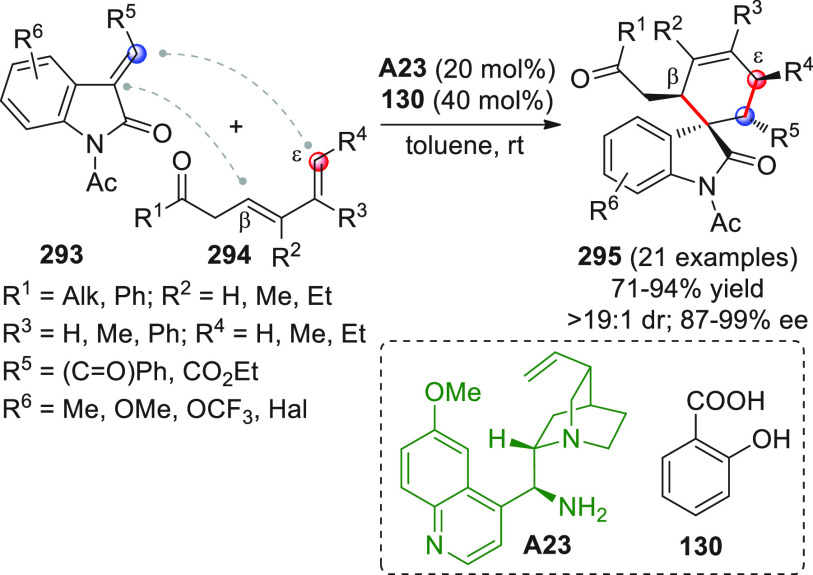


After this seminal work, where a chiral primary amine was used for HOMO-raising activation of 2,4-dienones, in 2014 the Chen group also applied this strategy to activate the remote ε-position of deconjugated linear 3,5-dienones of type **294**,^256^ by capitalizing on a recent work by themselves (see Scheme 93), in which a δ,ε-positioned C=C bond of interrupted cyclic 2,5-dienones acted as an inducing group for the formation of linear trienamines.^257^ Thus, a series of 3,5-dienones **294** were reacted with 3-alkylidene 2-oxindoles **293** via trienamine catalysis, producing spirocyclic oxindoles **295** as [4 + 2] cycloadducts in very good yields, remarkable diastereoselectivity (dr >19:1), and excellent enantioselectivity (Scheme 81). The reactions were conducted in toluene in the presence of catalytic amounts of 9-amino-9-deoxyepiquinine **A23** and salicylic acid (**130**).

**Scheme 82:**
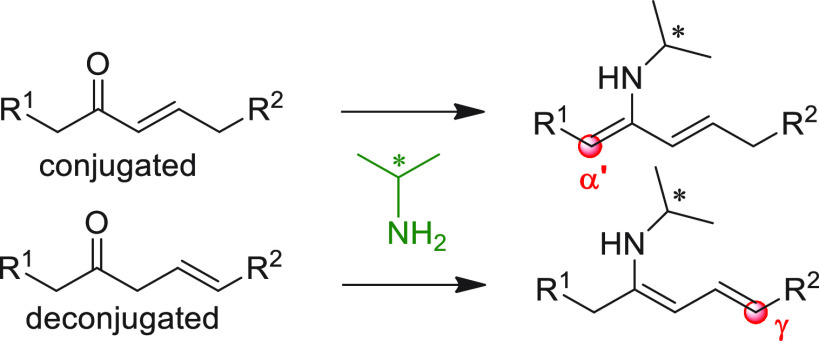


In the same year, Chen et al. applied the HOMO-raising activation strategy exerted by chiral primary amines to activate the γ-position of deconjugated ketones.^258^ Dienamine catalysis, in fact, could not be applied to activate the γ-position of linear conjugated ketones since, as shown in Scheme 82, cross-conjugated dienamines were preferably generated when the α′-CH group was present.^238,259−261^

**Scheme 83:**
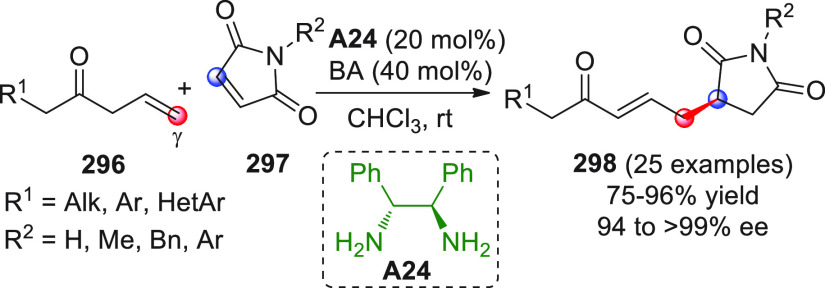


The vinylogous Michael addition of allyl alkyl ketones **296** to maleimide **297** was efficiently catalyzed by the commercially available (*R*,*R*)-1,2-diphenylethanediamine **A24**, furnishing γ-products **298** exclusively, in very good yields and with excellent enantioselectivity (Scheme 83). Linear or branched alkyl-substituted allyl ketones exhibited similar high reactivity. The procedure was simple and reliable also on a gram scale.

**Scheme 84:**
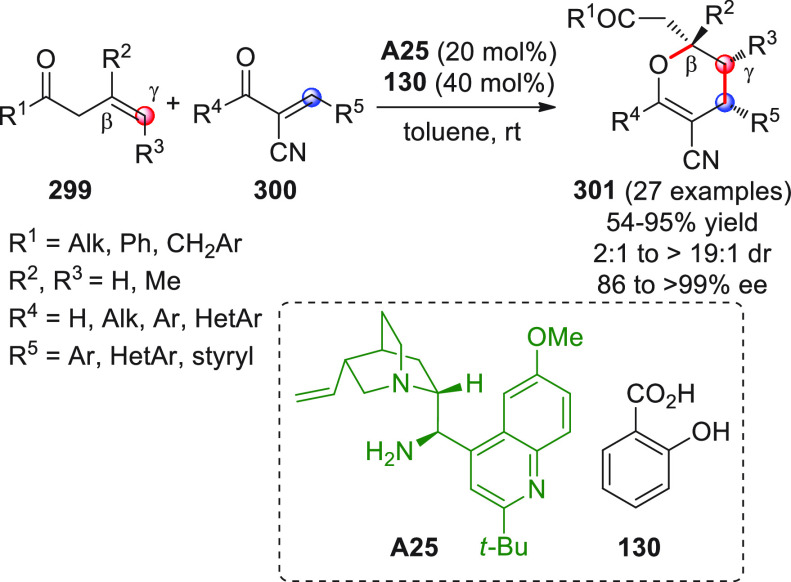


In 2016, the Chen group exploited the HOMO-raising activation by dienamine catalysis to activate the γ-position of allyl ketones in an inverse-electron-demand oxa-Diels–Alder (IED-oxa-DA) cycloaddition reaction with α-cyano-α,β-unsaturated ketones.^262^ Remote β,γ-regioselective IED-oxa-DA reactions with α,β-unsaturated aldehydes and β,γ-unsaturated-α-ketoesters through dienamine catalysis had already been developed by the Jørgensen group,^138,140^ but Chen was able to extend the strategy to linear enones. The dienophiles of the asymmetric IED-oxa-DA developed by Chen and co-workers were represented by electron-rich dienamines derived from condensation/isomerization of allyl ketones **299** with the cinchona-derived primary amine **A25**, while α-cyano-α,β-unsaturated ketones **300** were used as the diene counterparts (Scheme 84). Densely substituted dihydropyran products **301** were obtained in good yields, with excellent enantioselectivities and fair to outstanding diastereoselectivities. Oxadienes **300** with diverse β-aryl, heteroaryl, and 2-styryl groups were well tolerated, and various α′-substitutions were compatible with the reaction. The lowest dr was registered with R^4^ = CF_3_. Allyl ketones bearing diverse α′-alkyl groups showed similar reactivity, while the α′-phenyl group lowered the ketone reactivity, even if the corresponding product was obtained with excellent stereocontrol. Other oxadiene partners (not shown in Scheme 84), such as 3-benzoyl-2*H*-chromen-2-one and an α-nitro-α,β-unsaturated ketone, were assembled with allyl ketones **299**, enriching the palette of tetrahydropyran derivatives that could be prepared with this protocol.

**Scheme 85:**
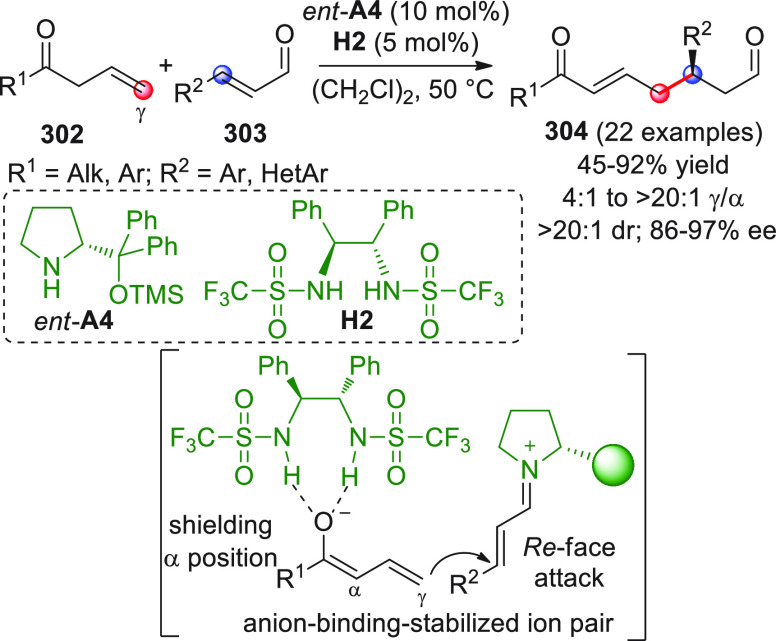


Another strategy for direct vinylogous Michael addition reactions (VMcR) of linear allyl ketones as donors to α,β-unsaturated aldehydes was proposed by Xu and co-workers in 2014.^263^ They reported the combined use of two catalysts, namely, the diphenylprolinol trimethylsilyl ether *ent*-**A4** and the bis(sulfonamide) **H2**, to trigger the VMcR between donors **302** and enals **303** (Scheme 85), by exploiting what they called “multifunctional supramolecular iminium ion catalysis” (SIC).^264^ Prolinol derivative *ent*-**A4** would activate the aldehyde by forming a conjugated iminium ion species, whose *Si* face is hindered from the attack of the nucleophile; the acidic −NHs of the cocatalyst **H2** would activate the vinylogous donor, stabilizing the dienolate by anion-binding interactions and favoring the γ-attack by shielding the α position. In the SIC strategy, the separation of the iminium–enolate ion pair by the cocatalyst **H2** increases the turnover number and allows a lower catalyst loading. The chiral 1,7-dioxo product compounds **304** were generated with good yields and excellent regio- and enantioselectivities. The reaction worked well only with α,β-unsaturated aldehyde **303** bearing aromatic substituents (both electron rich and electron poor groups). As for allyl ketones **302**, aromatic substituents bearing both electron-withdrawing and electron-donating groups were well tolerated, while aliphatic substituents furnished the products in lower yields and γ/α ratios.

**Scheme 86:**
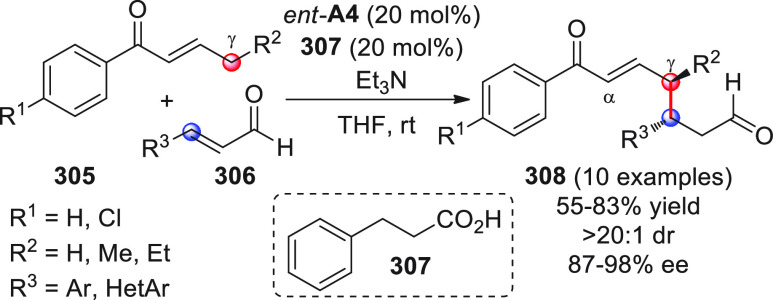


In 2016, Brenner-Moyer and co-workers published the first example of an organocatalyzed direct vinylogous Michael addition of linear conjugated ketones **305** to enals **306**, activated by the prolinol derivative *ent*-**A4** via iminium ion formation (Scheme 86).^265^ To achieve good reaction outcome in terms of both yield and enantioselectivity, the addition of Et_3_N (1 equiv) and carboxylic acid **307** (20 mol %) was necessary. With these conditions, after 3 days of reaction, the γ-alkylated products **308** were obtained. Sterically congested R^2^ groups such as a branched alkyl group hampered the reaction, while electron-withdrawing or -releasing substituents on cinnamaldehydes had minimal impact on product yield or enantioselectivity. No reaction was observed with an aliphatic enal. The authors applied these conditions to other acceptors to clarify which factors influenced the α- vs γ-alkylation, and interestingly, they could conclude that steric hindrance of the Michael acceptors, and not electronic influence, played a major role in regioselectively directing the alkylation. In fact, enals with larger R^3^ groups favored γ-alkylation, while enals with smaller R^3^ groups favored α-alkylation of these linear vinylogous Michael donors.

**Scheme 87:**
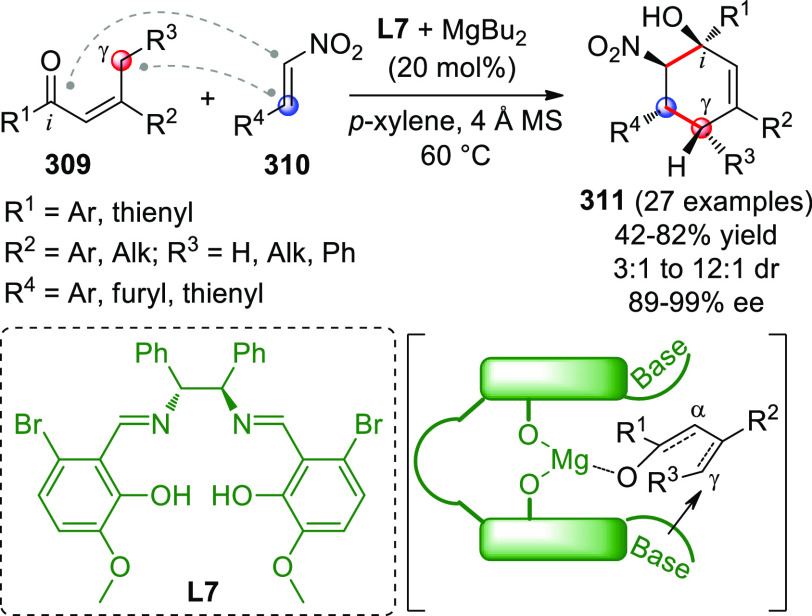


An alternative catalytic strategy for the site-selective and stereocontrolled γ-functionalization of linear enones of type **309** was reported by the Wang group in 2013 (Scheme 87).^266^ The complex **L7**-Mg, where the magnesium ion is coordinated by a salen-type chiral ligand, could direct γ-deprotonation of linear α,β-unsaturated ketones, thanks to the presence of basic groups within the ligand and contemporary hindrance of the α-position of the donor by suitable substituents. The β,β-disubstituted α,β-unsaturated ketones **309** gave a Michael-type addition to nitroalkenes **310**, followed by cyclization of the nitronate intermediate to the *ipso* carbonyl, leading to a variety of optically active cyclohexene frameworks **311**. Aliphatic nitroalkenes did not undergo this transformation, while different aryl groups at either the β- or α′-positions of the vinyl ketones were compatible.

**Scheme 88:**
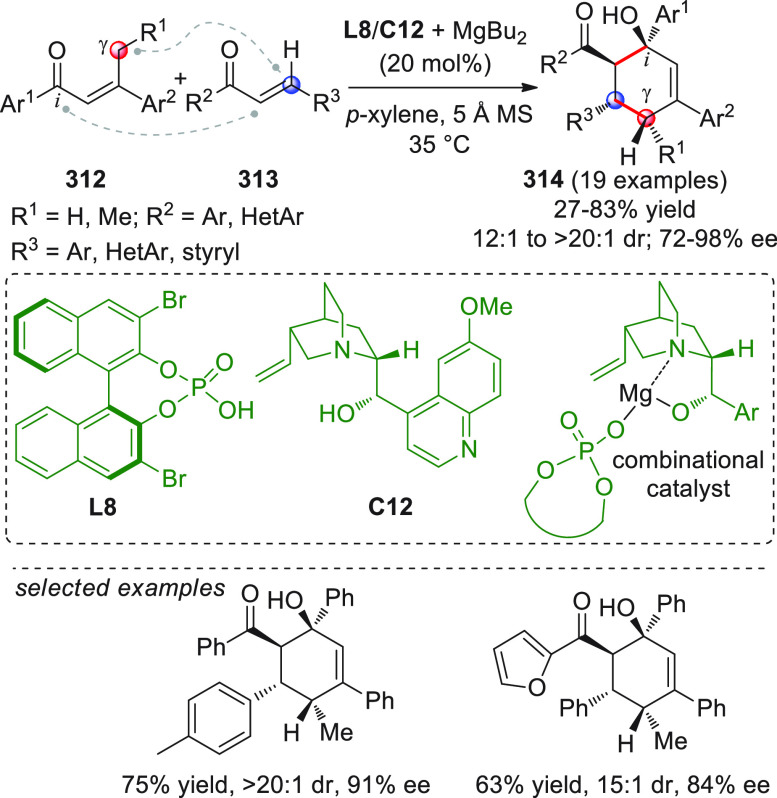


The following year, Wang et al. designed a combinational magnesium catalyst for the stereocontrolled cross reaction of enones.^267^ The stereocontrol of the reaction between **312** and **313** (Scheme 88) was particularly challenging, since the reaction partners have a very similar substitution pattern, but the use of the phosphoric acid **L8**, together with quinidine **C12** and MgBu_2_ in *p*-xylene was able to ensure the formation of γ,*ipso*-[4 + 2] cyclization products **314** in modest to good yields and high diastereo- and enantioselectivities. Nucleophilic enones **312** at first coordinate to the metal center of the preformed combinational catalyst (Scheme 88), that determines the attack direction to electrophilic enones **313**. Several control experiments were performed by the authors, concluding that the absolute configuration of the products is mainly induced by the phosphoric acid, while the cinchona alkaloid in the combinational catalysis plays the role of reaction promoter.

**Scheme 89:**
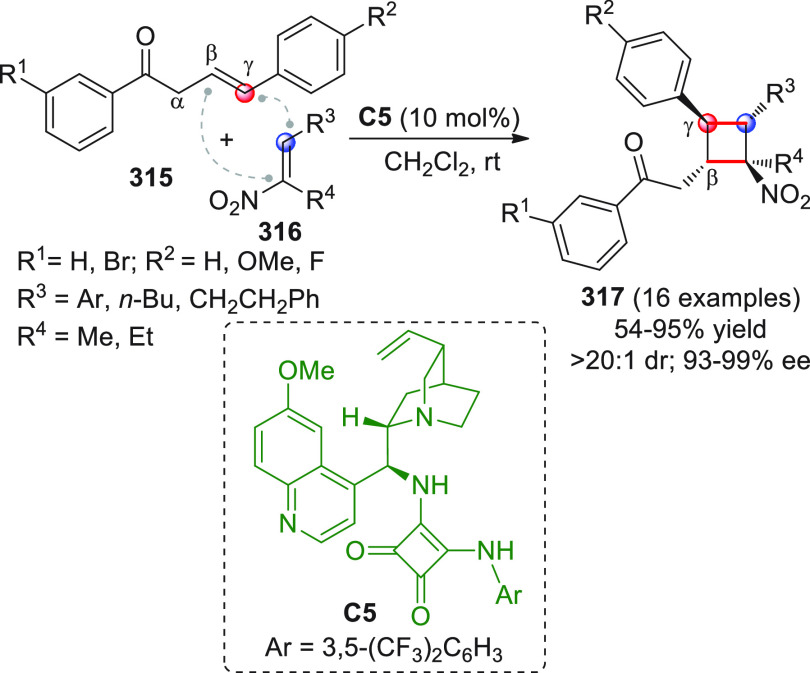


Very recently, Hong and co-workers exploited the vinylogous reactivity of deconjugated ketones **315** toward nitroolefins **316** to prepare fully substituted cyclobutanes of type **317** (Scheme 89) via a γ,β-regioselective [2 + 2] annulation.^268^ The reaction was catalyzed by cinchona squaramide catalyst **C5**, that favors the enolization of enone **315** through the squaramide hydrogen-bonding activation and deprotonation at the α-position by the quinuclidine moiety and subsequent activation of the nitroolefin by hydrogen-bonding. Cyclobutanes **317** were obtained in good yields and excellent diastero- and enantioselectivities. Lower yields were registered with nitroalkenes with aliphatic substituents on the β position. The authors underlined how the α vs γ regioselectivity was governed not only by the organocatalyst but also by the substituents on both the nitroolefins and the vinylogous donors. Small amounts of Michael-type products from the donor α-site were however formed, even under the optimized conditions.

**Scheme 90:**
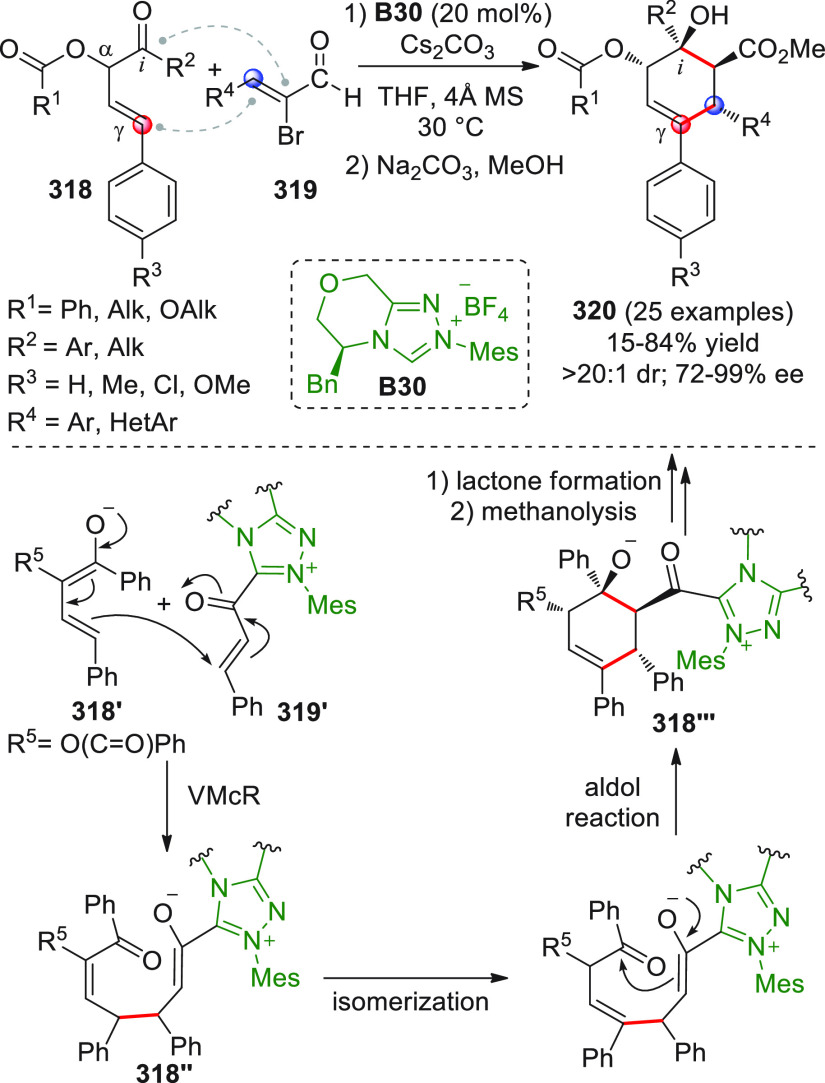


The first use of α-benzoyloxy ketones of type **318** as dienolate precursors was reported by Fang and co-workers in 2017 in an enantioselective all-carbon [4 + 2] annulation (Scheme 90).^269^ The in situ formed dienolate **318′** is able to react with α,β-unsaturated acyl azolium species **319′**, in turn in situ generated from α-bromoenals **319**(68,270,271) under NHC catalysis, giving the vinylogous Michael adduct **318′′**. Isomerization and intramolecular aldol reaction then lead to the formation of intermediate **318′′′**, that after lactonization provides a β-lactone with the release of the chiral carbene **B30**. Methanolysis of β-lactone species furnishes the cyclohexene products **320**. This strategy, thanks to an efficient isomerization process, not observed in the previous reports of intermolecular annulations,^272^ allowed the synthesis of cyclohexenes **320**, inaccessible from conventional Diels–Alder reactions, in modest to good yields, as single diastereoisomers and with good enantioselectivities. Lower yields were registered when aliphatic ketones (R^2^ = Alk), alkyl esters (R^1^ = Alk), or carbonate (R^1^ = OAlk) were used. Moreover, when R^4^ was an aliphatic group, the annulation reaction did not occur under the optimal conditions.

##### 4.3.1.2 Cyclic Pronucleophiles

**Scheme 91:**
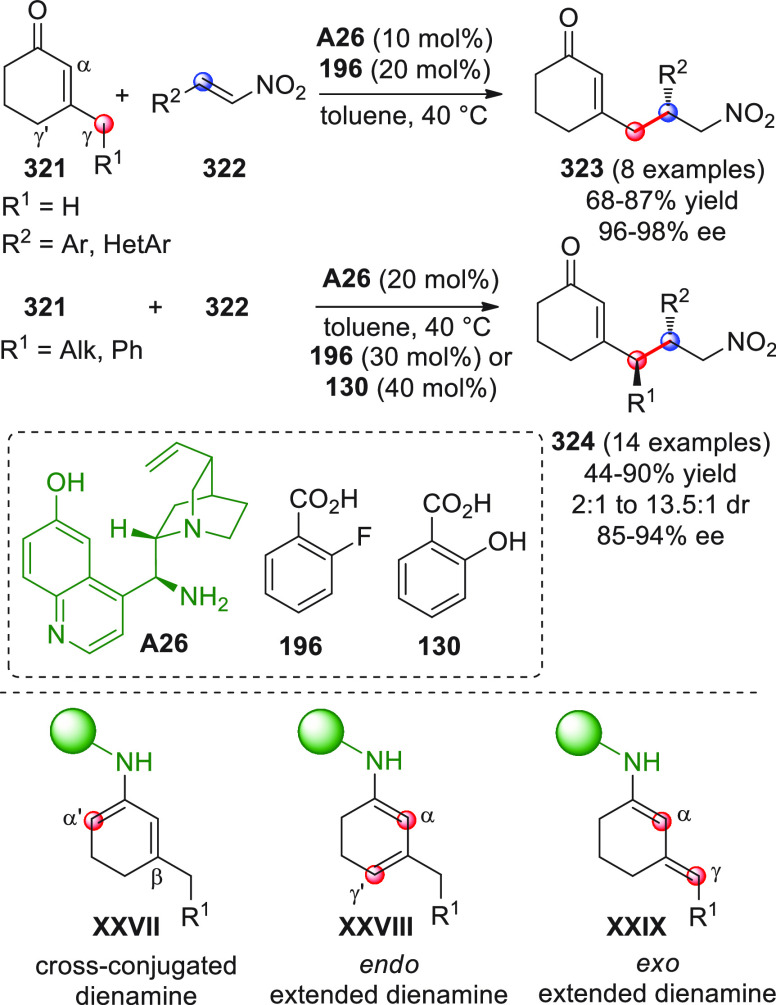


In 2010, Melchiorre and co-workers were the first who developed a procedure for a direct intermolecular vinylogous Michael addition of unmodified β-substituted cyclohexenone derivatives to nitroalkenes via dienamine catalysis.^228^ Cyclohexenones activated as dienamines have multiple potential nucleophilic sites, the α′-position in the kinetic cross-conjugated dienamine **XXVII**, the α- and γ′-positions in the *endo* extended dienamine of type **XXVIII**, and the α- and γ-positions in the *exo* extended dienamine of type **XXIX** (Scheme 91). Theoretical calculations accounted for a thermodynamically driven site-selective formation of an *exo* cyclic dienamine favored over the other two. The bifunctional *epi*-quinine-derived catalyst **A26** (10 mol %) and 2-fluorobenzoic acid (**196**, 20 mol %) as cocatalyst in toluene were the best combination to catalyze the reaction between 3-methylcyclohexenone (**321**, R^1^ = H) and nitroalkenes **322**, to give products **323** in high yields, complete γ-regioselectivity, and excellent enantiomeric excesses. Different substituents at the aromatic moiety of β-nitrostyrene derivatives were well-tolerated, regardless of their electronic properties, while aliphatic nitroalkenes, as well as a different cyclic scaffold of the nucleophilic component (i.e., 3-methyl-2-cyclopenten-1-one), resulted in a complete loss of reactivity. A modified catalyst salt combination was used to catalyze the reaction of prostereogenic **321** (R^1^ ≠ H) to give products **324** with two contiguous stereogenic centers, in good yield and enantioselectivity, with variable levels of diastereoselectivity in favor of *anti*-adducts.

**Scheme 92:**
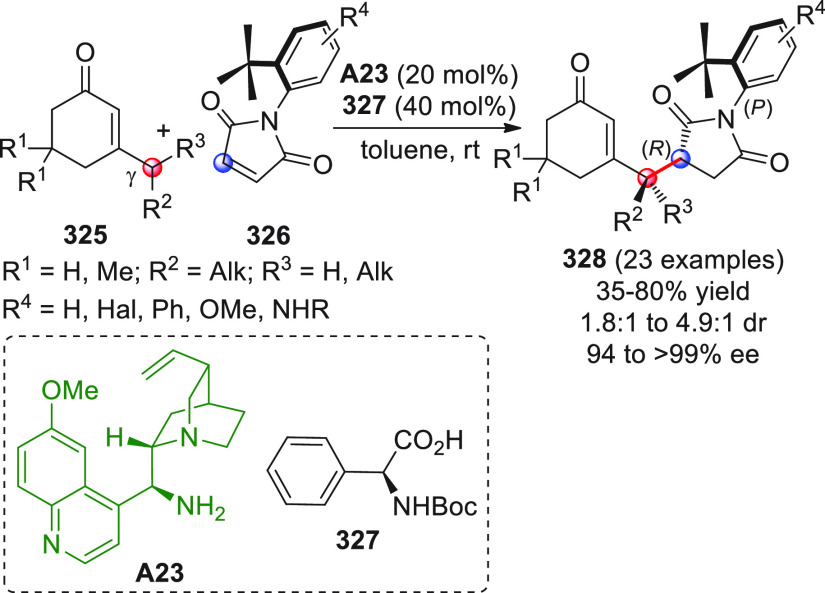


Some years later, the same activation mode was exploited by Bencivenni and collaborators to catalyze the vinylogous Michael addition of 3-alkyl cyclohexenones **325** toward *N*-(2-*t*-butylphenyl)maleimides **326** (Scheme 92).^273^ The cinchona alkaloid-derived amine catalyst **A23**, forming a dienamine intermediate, was able not only to transfer its stereochemical information to the prochiral γ-position of the donor but also to the prochiral axis of the acceptor, directing the attack from the side not shielded by the *tert*-butyl group. The authors registered an efficient control in the desymmetrization of maleimides **326**, isolating adducts **328** with low diastereomeric ratios but high enantiomeric excesses.

The reaction of cyclic enones of type **325** with alkylidene, allylidene, and alkynylidene malononitriles catalyzed by a quinidine-derived catalyst was developed by Chen and co-workers.^261^ With alkylidenemalononitriles, γ-regioselective VMcR occurred, providing the corresponding products with very low enantioselection. Instead, using allylidene or alkynylidene malononitriles, α′,β-regioselective [4 + 2] bicyclo[2.2.2]octane adducts generated by the cross-conjugated dienamine of type **XXVII** (Scheme 91) were exclusively produced. Interestingly, the authors proved that the reaction, an apparently concerted Diels–Alder cycloaddition, actually proceeded by a stepwise Michael–Michael cascade.

**Scheme 93:**
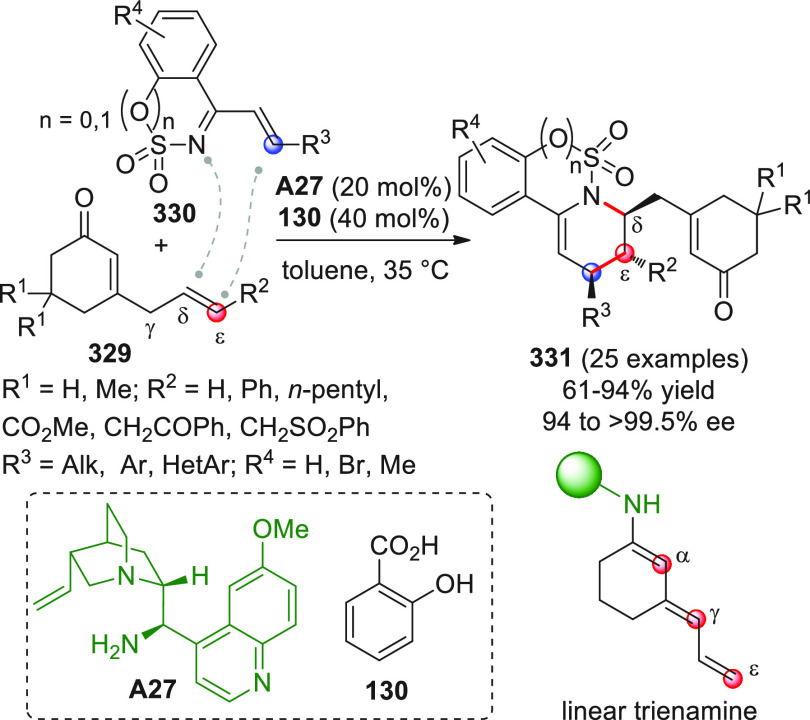


In 2013, the Chen group introduced the use of interrupted 3-allylcyclohexen-2-ones **329** as bisvinylogous donors by trienamine catalysis (Scheme 93).^257^ In fact, the use of 2,4-dienones was hampered, because of their tendency to enolize at the α′-position to give cross-conjugated trienamines. Using the deconjugated dienones **329**, instead, polyconjugated linear trienamines were formed, with the possibility of the transmission of the HOMO raising activation at the remote ε position, through the conjugated π system. Thanks to the activation exerted by the epiquinidine catalyst **A27**, and using sialic acid **130** as cocatalyst, compounds **329** reacted with electron deficient 1-azadienes **330** (in particular 3-vinyl-1,2-benzoisothiazole-1,1-dioxides (n = 0), or analogs with a 1,2,3-benzoxathiazine-2,2-dioxide motif (n = 1)) in an asymmetric inverse-electron-demand aza-Diels–Alder reaction. Cycloadducts **331** were obtained in high yields, with exclusive ε,δ-regioselectivity, and excellent stereocontrol. The reaction was efficient also on an acyclic *N*-tosyl-1-azadiene.

**Scheme 94:**
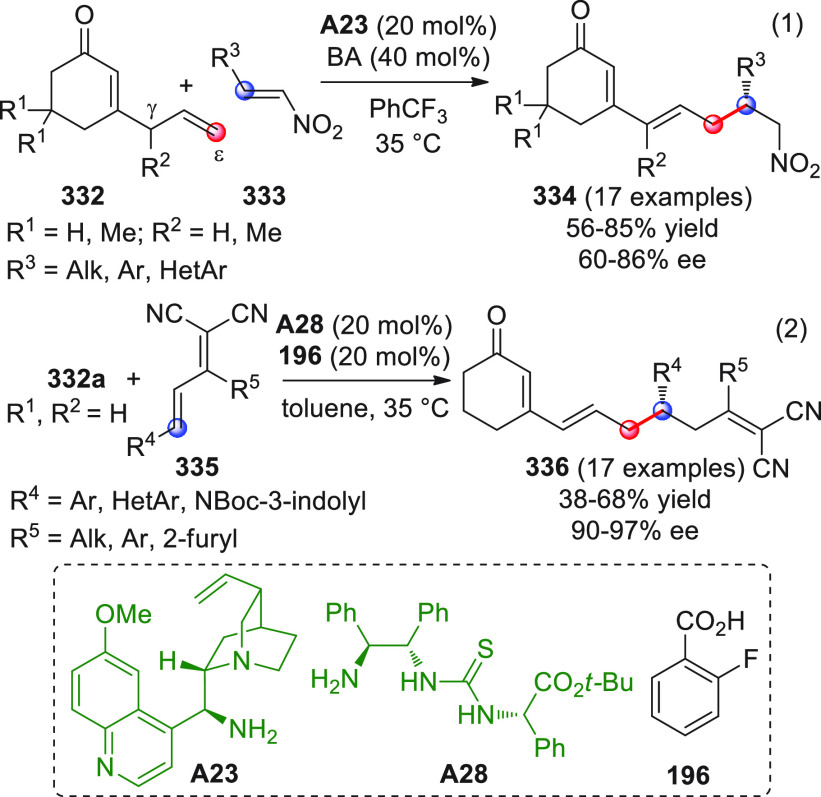


The following year, the exploitation of this trienamine catalysis-based strategy permitted to further disclose the bisvinylogous reactivity of 2,5-dienones **332** in direct enantioselective 1,4- or 1,6-additions.^274^ The reaction of **332** with nitroalkenes **333** provided linear Michael adducts **334** (Scheme 94, eq 1), with remarkable remote ε-regioselectivity, while cyclized ε,β-cycloaddition products were not observed. The enantioselectivity given by the use of the epiquinine catalyst was good, even if not excellent. Nitroalkenes bearing diversely substituted aryl or heteroaryl groups were well tolerated, while alkyl-substituted nitroalkenes gave the worst results in terms of both yield and enantioselection. The reaction could not be applied to ε-substituted 2,5-dienones, that were almost inert as donors.

Bifunctional primary amine-thiourea catalyst **A28** was necessary to favor the 1,6-addition of **332a** to α,α-dicyanodienes **335** forming adducts **336** (Scheme 94, eq 2).^275^ In fact, when cinchona-derived catalysts were tested, δ,ε-Diels–Alder cycloadducts were the major products. With the optimized conditions, linear products **336** were obtained in modest yields, but with complete ε-regioselectivity and high enantioselection. The reaction was not applicable to both α,α-dicyanodienes without β-substitution (R^5^ = H in **335**), and ε-substituted cyclic 2,5-dienones, which proved to be almost inert.

**Scheme 95:**
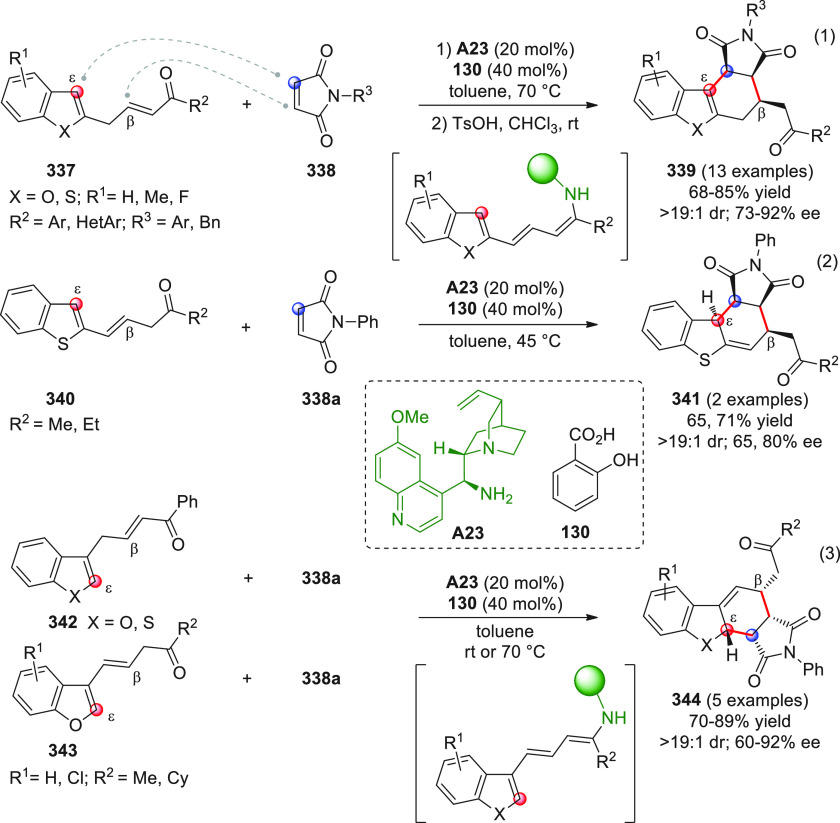


In 2014, the Chen group carried out the investigation of HOMO-activation of the remote C=C bond via trienamine catalysis and applied this strategy to other nucleophiles and reaction types. For example, this methodology was used to activate 2-vinyl heteroarenes, such as β-(2-benzofuryl)methyl enones **337** in the coupling reaction with maleimides **338** (Scheme 95, eq 1).^276^ The conditions were similar to those previously reported, i.e. 9-amino-9-deoxyepiquinine **A23** (20 mol %) as chiral catalyst and sialic acid **130** (40 mol %) as acidic cocatalyst, in toluene. Treating the reaction crude in acidic conditions, the authors isolated cycloadducts **339** in good yields and diastereo- and enantioselectivities. However, under these conditions, 2,5-dienone substrates of type **337** bearing α′-enolizable groups did not react at all with dienophile **338a**; thus the authors prepared deconjugated 3,5-dienone-type substrates of type **340**. Benzofuryl derivatives were not stable in the reaction conditions, while 2-benzothiophenyl enones **340** reacted with maleimide **338a**, directly furnishing, without acidic treatments, products **341** possessing four contiguous stereocenters (Scheme 95, eq 2). This asymmetric dearomatizative Diels–Alder protocol was also applied to 3-benzofuryl and 3-benzothiophenyl derivatives and, even in these cases, non-α′-enolizable 2,5-dienones **342** produced the corresponding ε,β-locked cycloadducts **344** (Scheme 95, eq 3), while when R^2^ in the α′-position was an enolizable alkyl group (R^2^ = Me, Et) the reaction had to be performed starting from deconjugated 3,5-dienones of type **343**. The generality of this method was proven by reacting **343** (R^1^ = H, R^2^ = Me) with other dienophiles, such as 3-alkylidene oxindoles and benzylidenecyanoacetate, obtaining the corresponding ε,β-cycloadducts in fair to good yields and excellent enantioselectivities.

**Scheme 96:**
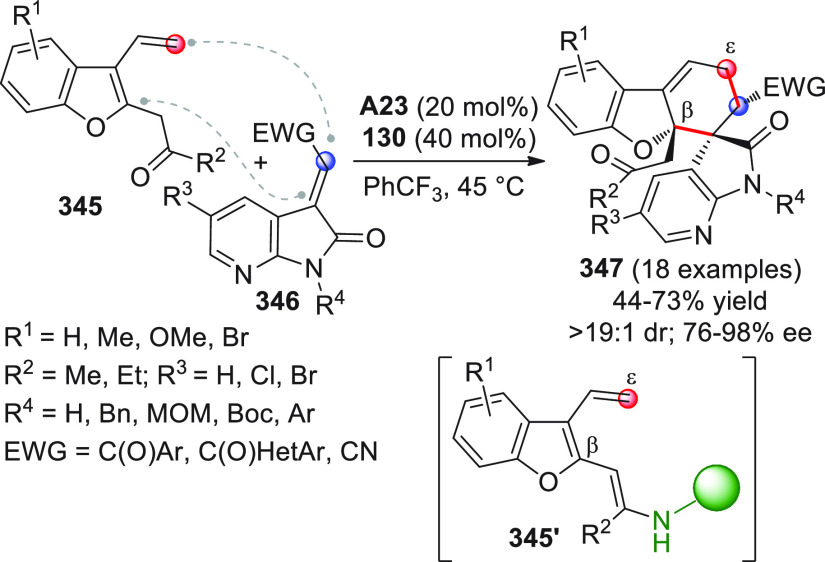


Some years later, the same asymmetric dearomatizative [4 + 2] reaction via trienamine catalysis was carried out on 2-(3-vinylbenzofuran-2-yl)ethan-1-ones **345**, activated by epiquinine **A23** to form the trienamine reactive species **345′**, and using 3-olefinic 7-azaoxindoles **346** as the dienophile partners (Scheme 96). The reaction products consisted of fused spirocycles of type **347**, bearing two vicinal tetrasubstituted stereogenic centers. They were obtained in fair yields and with good to excellent stereocontrol. Several protecting groups on the oxindole nitrogen were tolerated, and lower yield and enantioselectivity were registered with NH-free **346**.

**Scheme 97:**
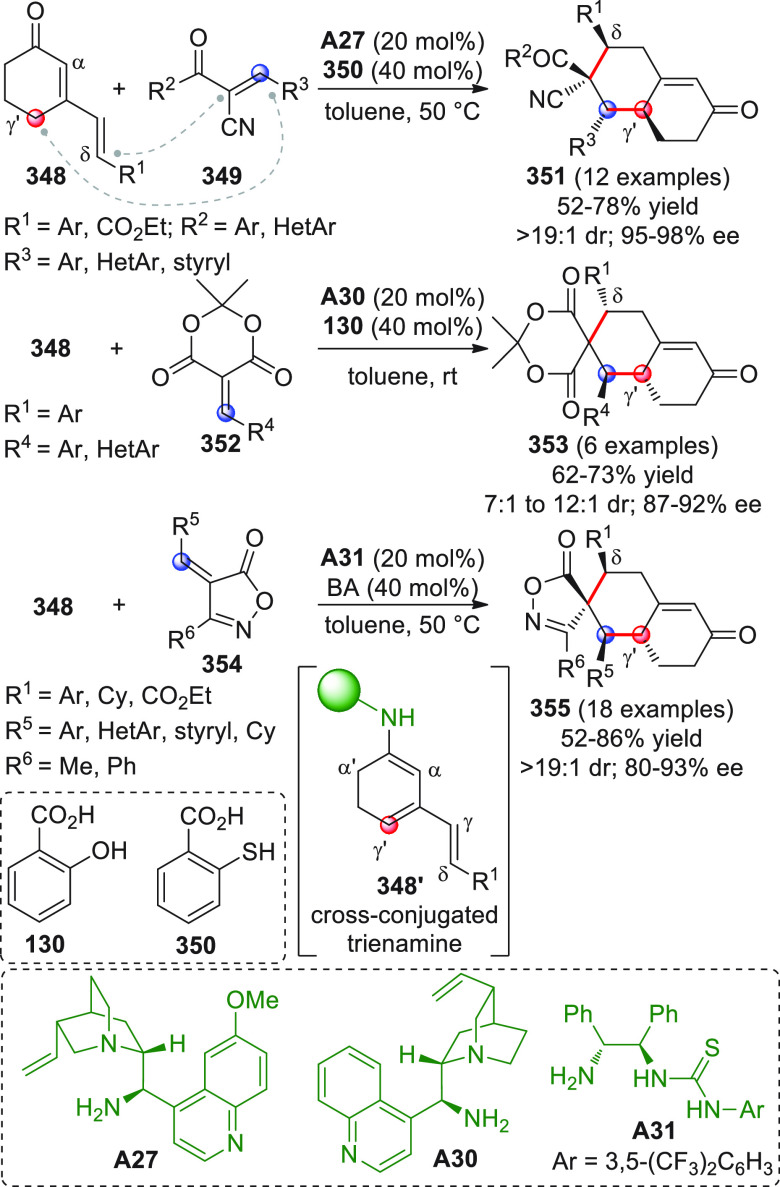


When applying the trienamine-activation strategy to 2,4-dienones **348** in coupling reactions to benzoyl-bearing activated alkenes **349**, Chen and collaborators did not isolate the expected α′,β-locked [4 + 2] cycloadducts (vide supra),^261^ but they witnessed the regioselective formation of γ′,δ-locked [4 + 2] products **351**, due to the formation of unprecedented cross-conjugated trienamine intermediates of type **348′** (Scheme 97).^277^ The combination of cinchona-derived catalyst **A27** and 2-mercaptobenzoic acid **350** secured the formation of **351** in good yields, with excellent diastereo- and enantioselectivities. Different aryl and heteroaryl groups at the β-position of acceptors **349** were compatible with this procedure, but the reaction did not furnish the desired products when R^1^ (in the donor) and R^3^ (in the acceptor) were alkyl substituents. This methodology was applied to other acceptors, such as alkenes **352** derived from Meldrum’s acid and isoxazolones **354**, obtaining good results in terms of yields, but with lower enantiomeric excesses. The analysis of the absolute configuration of the stereocenters in **355** suggested that the [4 + 2] reaction does not proceed along a concerted Diels–Alder pathway, but rather via a stepwise vinylogous Michael–Michael mechanism.

**Scheme 98:**
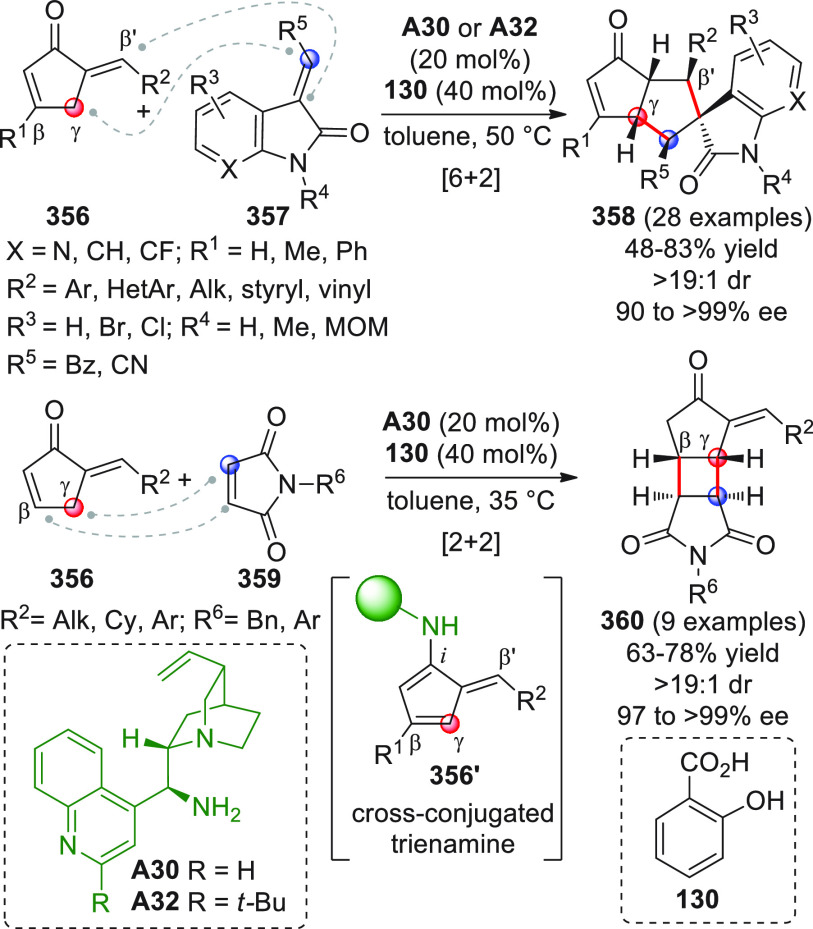


The use of α′-alkylidene-2-cyclopentenones **356** as vinylogous donors, via trienamine catalysis, was cleverly disclosed by the Chen group in 2018.^209^ Activation of **356** with 2′-*tert*-butyl-9-amino-9-deoxyepicinchonidine (**A32**) in toluene carried to the in situ formation of HOMO-raised cross-conjugated trienamine **356′** that, depending on the acceptor nature, gave rise to either an asymmetric γ,β′-regioselective [6 + 2] cycloaddition or a γ,β-regioselective [2 + 2] cycloaddition (Scheme 98). With the highly electrophilic 3-olefinic (7-aza)oxindoles **357**, under the catalysis of **A30** or **A32** and salicylic acid (**130**) in toluene at 50 °C for 72 h, the reaction furnished [6 + 2]-cycloadducts **358** with five contiguous stereogenic centers, in good yields and with high levels of diastereo- and enantiocontrol. When the same conditions were applied to the reaction of **356** with maleimides **359**, fused cyclobutanes of type **360**, derived by a [2 + 2] cycloaddition, were obtained with good yields and excellent stereoselectivities. DFT computational calculations were performed, showing that the [6 + 2] cycloaddition likely proceeds in a stepwise vinylogous Michael–Michael reaction, while the [2 + 2] cycloaddition might involve a process via an γ,*ipso*-[4 + 2]-cycloaddition, followed by a concerted ring-opening and ring-closure process, to form the products **360**.

**Scheme 99:**
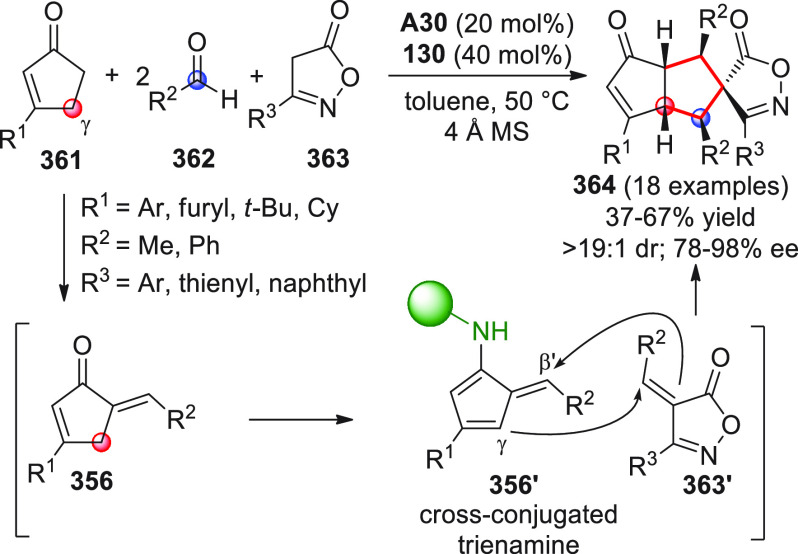


An extension of this strategy was reported the following year again by Chen and collaborators.^278^ They developed an asymmetric four-component [5 + 1+1 + 1] formal cycloaddition reaction between 3-substituted 2-cyclopentenones **361**, aryl aldehydes **362**, and 3-methylisoxazolones **363**, catalyzed by the cinchona-derived primary amine **A30** and salicylic acid (**130**) in toluene (Scheme 99). Products **364** were obtained in modest yields, but with a high level of enantioselectivity. Moderate enantiomeric excesses were registered when 3-phenylisoxazolone or thiophene-2-carboxaldehyde were used (78% and 79% ee, respectively); otherwise, ee higher than 91% was measured. The authors investigated the reaction mechanism and proposed a cascade process, in which the 3-methylisoxazolone acts also as a cocatalyst in the formation of the key dienone **356**. Next, a domino vinylogous Michael–Michael addition between **356′** and **363′** takes place carrying to products **364**. Other types of activated methylene nucleophiles different from isoxazolones **363** were used, expanding this strategy to the construction of chiral frameworks with increased structural diversity.

**Scheme 100:**
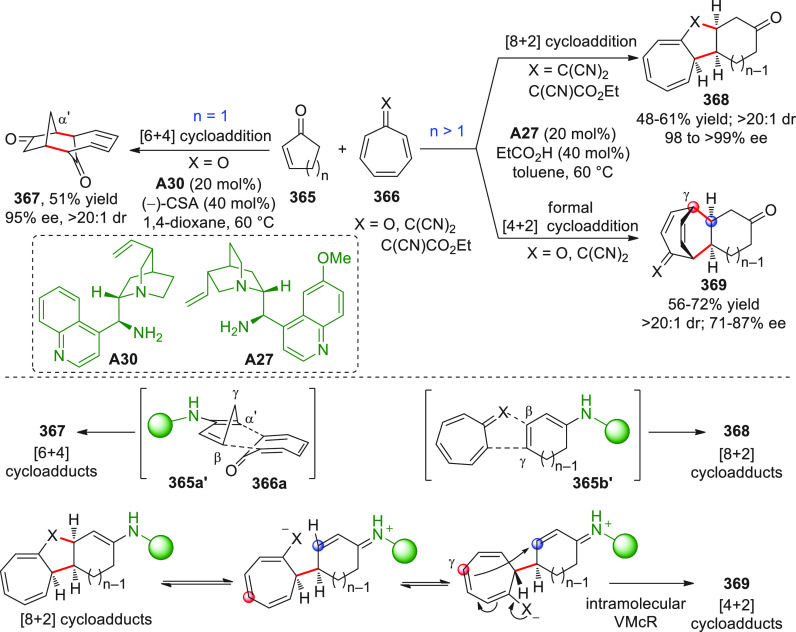


In 2017, Jørgensen and co-workers published the first example of organocatalytic [6 + 4] and [8 + 2] cycloadditions, together with a [4 + 2] pathway.^208^ Cyclic enones **365**, activated as dienamines by cinchona-based primary amine catalysts, reacted with tropone **366a** or cyanoheptafulvenes **366b** and **366c** (Scheme 100). These cycloadditions were periselective and exhibited both diastereo- and enantioselectivity, affording complex bicyclic structures **367**–**369** with up to four contiguous stereocenters. To rationalize the observed peri- and stereoselectivity, several control reactions were carried out and potential energies of all products were calculated relative to their starting compounds. The α′,β-locked [6 + 4] cycloadduct **367**, formed from cyclopentenone **365a** (n = 1), is favored when the cross-conjugated dienamine **365a′** reacts with tropone **366a** (X = O). In this case, the dienamine intermediate **365a′** reacts at the α′-position along a nonvinylogous pathway. Conversely, [8 + 2] cycloadditions to γ,β-locked products **368** are favored when linear dienamines **365b′** (*n* > 1) react with cyanoheptafulvenes **366** (X = C(CN)_2_, C(CN)CO_2_Et). The pathway to γ,β-locked [4 + 2] cycloadducts **369** was explained as a rapid rearrangement of unobserved [8 + 2] enamine intermediates, by an intramolecular vinylogous Michael type reaction (Scheme 100, bottom). Interestingly, the authors concluded that all these higher-order cycloadditions might proceed through stepwise mechanisms.

**Scheme 101:**
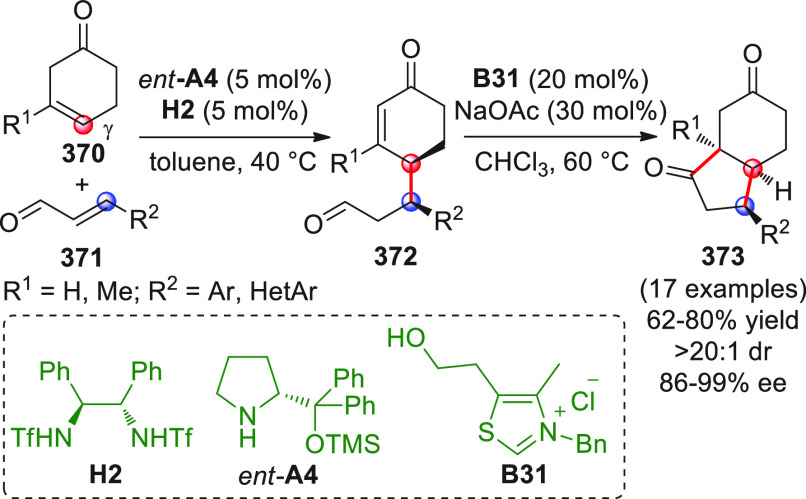


In 2017, Xu and co-workers applied the multifunctional supramolecular iminium catalysis (SIC), already optimized for linear donors (see Scheme 85),^263^ to perform the vinylogous Michael reaction of deconjugated 3-cyclohexenones **370** with α,β-unsaturated aldehydes **371** to give chiral 1,7-dioxo adducts **372** (Scheme 101).^279^ The combination of the Jørgensen–Hayashi catalyst *ent*-**A4** for the acceptor activation as iminium ion, with cocatalyst **H2** for dienolate stabilization, in a low polar solvent such as toluene, provided the almost exclusive γ-attack (γ/α 18:1), a diastereomeric ratio >20:1, and excellent enantioselection. Compounds **372** were the products of the first step of two sequential reactions. After the optimization process, the author did not separate **372** anymore, but after the evaporation of the solvent, they exploited the NHC-precatalyst **B31** in chloroform to catalyze an intramolecular Stetter reaction, obtaining products **373** with the Hajos–Wiechert ketone skeleton.

**Scheme 102:**
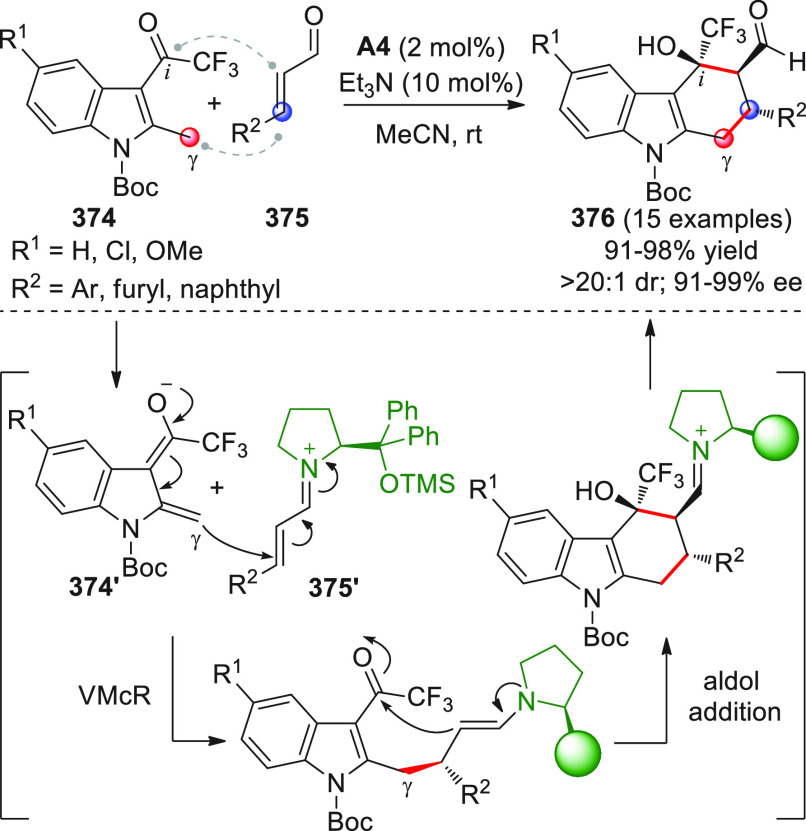


The asymmetric organocatalytic domino vinylogous Michael/aldol reaction between 3-(trifluoroacetyl)-2-methylindoles **374** and enals of type **375** was reported by the Enders group in 2018.^280^ The dienolate species **374′**, derived from **374** by γ-methyl deprotonation, regioselectively attacks the β-position of iminium ion-activated acceptors **375′** (Scheme 102). The VMcR is followed by an intramolecular aldol reaction, to afford trifluoromethylated tetrahydrocarbazoles of type **376**, bearing three vicinal stereocenters. Products **376** were produced in high yields, as single diastereomers, and with high enantiomeric excesses. Protecting groups different from Boc on the indole nitrogen (such as Bz, Ac, Me), as well as the use of unprotected indoles, were not compatible with this strategy. The reaction did not succeed also when a methyl group was present in place of a trifluoromethyl group.

**Scheme 103:**
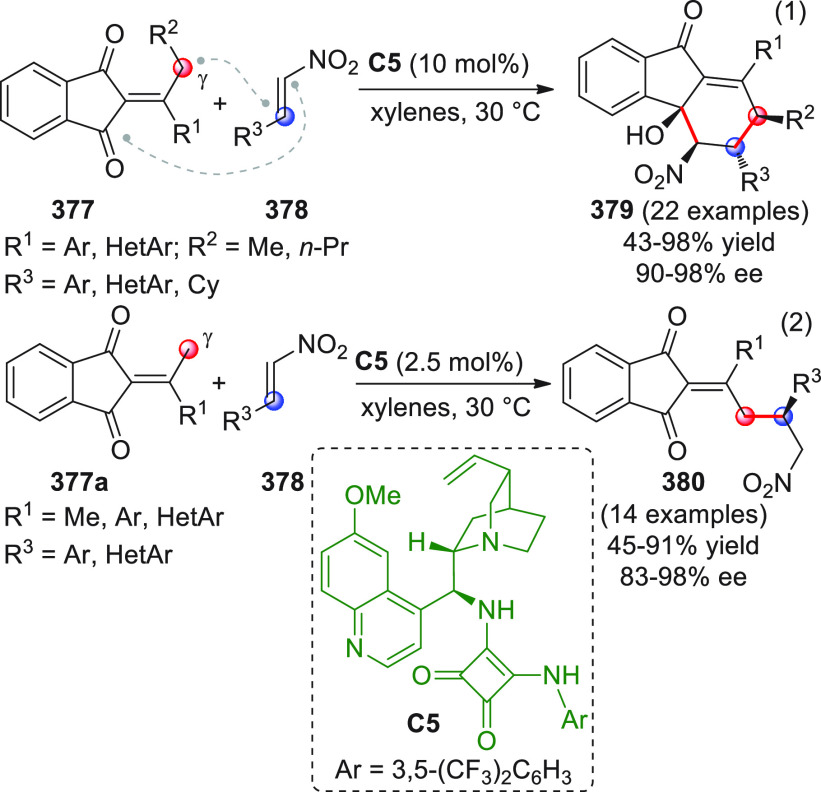


The pronucleophilic character of 2-alkylidene-1*H*-indene-1,3-(2*H*)-diones **377** in asymmetric vinylogous Michael reactions was discovered by Lin and colleagues in 2016.^281^ The high γ-proton acidity of **377**, due to the strong electron-withdrawing effect of the 1,3-indandione moiety, allows for the facile γ-deprotonation and formation of vinylogous nucleophiles able to attack nitroolefins **378**. One of the two carbonyl groups in the indandiones **377** intercepts the emerging Michael products, giving an intramolecular Henry reaction. This tandem vinylogous Michael addition/Henry reaction cascade was promoted by the bifunctional squaramide-cinchonidine catalyst **C5** in xylenes and furnished tetrahydrofluoren-9-ones **379** in very good yields and enantiomeric excesses (Scheme 103, eq 1). In each case, despite four contiguous stereocenters being formed, only one diastereoisomer is obtained. The reaction did not work with aliphatic nitroolefins, with the exception of R^3^ = *c*-hexyl, which anyway had scarce efficiency. It is noteworthy that when arylethylidene indanones were used (**377a**, R^2^ = H), the predominant formation of the vinylogous Michael addition products **380** was observed (Scheme 103, eq 2). Since the authors were confident that the overall cyclization consisted of a stepwise vinylogous Michael/Henry cascade, they explained the formation of linear Michael products **380**, by assuming that Henry cyclization did indeed take place, followed by a retro-Henry opening to the thermodynamically more stable products **380**.

**Scheme 104:**
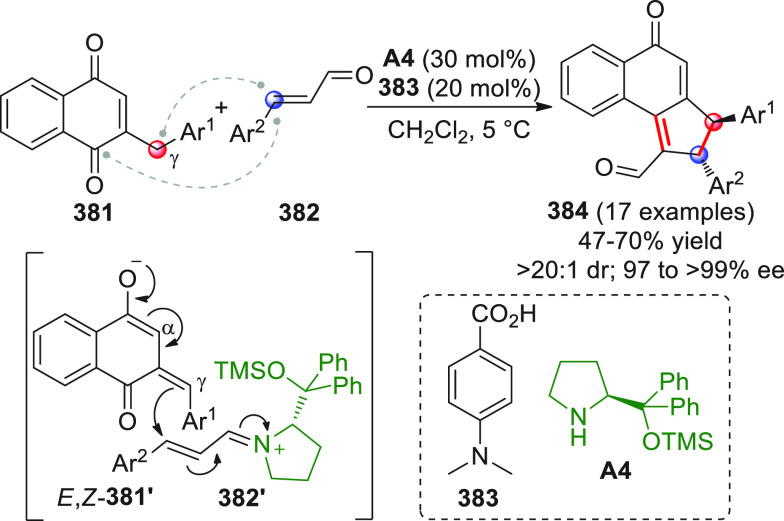


Albrecht and colleagues were the first who disclosed the vinylogous pronucleophilic character of 2-benzyl substituted-1,4-naphthoquinones of type **381**, which were used in an organocatalytic cascade reaction with nonenolizable enals **382**, to give carboannulated naphthalen-1(4*H*)-one derivatives **384** (Scheme 104).^282^ Under the optimized conditions, the dienolate species **381′** promoted a vinylogous Michael attack to enals activated as iminium ions **382′** by the diphenylprolinol trimethylsilyl ether **A4** (30 mol %), and with 4-(dimethylamino)benzoic acid (**383**) as an additive. Co-catalyst **383** favored the γ- vs α-attack and the formation of the desired product. After the VMcR, an intramolecular aldol condensation provided cycloadducts **384** in modest to fair yields, but with excellent diastereo- and enantioselectivities. Both electron-withdrawing and electron-donating groups on the aromatic substituent of enals **382** were well tolerated, while the use of aliphatic, linear enals resulted in sluggish conversion. A transition state involving *E*,*Z*-configured dienolate **381′** attacking the less hindered face of iminium ion **382′** (Scheme 104) was proposed by the authors to justify the stereoconfiguration of the products.

**Scheme 105:**
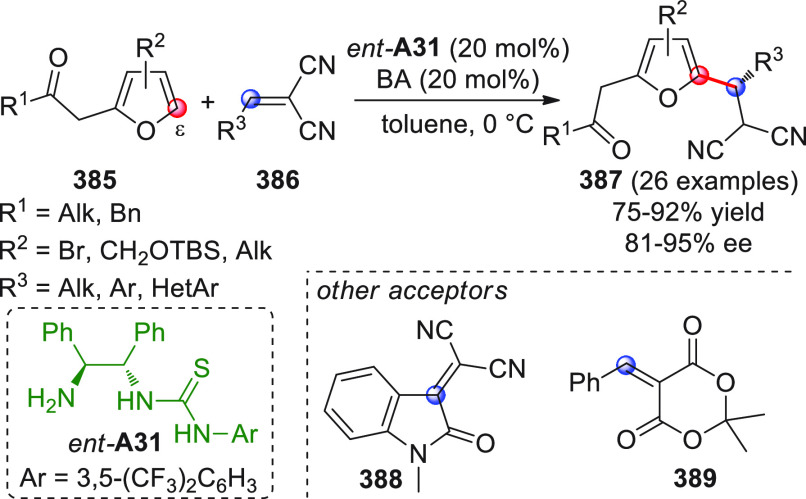


In 2014, the Chen group exploited the electronic transfer through the C=C bonds of a polyconjugated system for the HOMO activation of the C5-position (ε) of 2-furfuryl ketones of type **385** (Scheme 105).^283^ Through the formation of trienamine species with the chiral bifunctional primary amine-thiourea *ent*-**A31**, the π-system of **385** was activated for an enantioselective Friedel–Crafts (FC) alkylation. This was an alternative strategy with respect to the other catalytic asymmetric FC reactions already present in the literature, that normally proceeded by lowering the LUMO energy of electrophilic partners. The reaction was developed with alkylidenemalononitriles **386**, as electron-deficient alkene, to generate products of type **387** in high yields and enantioselectivities. The alkylation was completely ε-regioselective (C5-position of the furan system). Various aryl and alkyl groups on the alkylidenemalononitrile acceptors were well tolerated, while the lowest enantiomeric excesses were registered with heteroaryl groups. Various α′-alkyl substituents on the 2-furfuryl ketones **385** were compatible, as well as groups at the C4- and C3-positions of the furan ring. Other activated alkenes were explored as acceptor components. The reaction with compounds **388** and **389** (Scheme 105) also proceeded toward the alkylation at the 5-position of the furan, even if with definitely lower enantioselectivity, and moreover other bifunctional catalysts had to be used. Interestingly, a similar reaction with nitrostyrene gave the α-attack product, while the maleimide behaved as a dienophile in Diels–Alder cycloaddition with the furan system. These data revealed that regioselectivity in these processes is strongly influenced by both electrophilicity and structural characteristics of the alkenes.

**Scheme 106:**
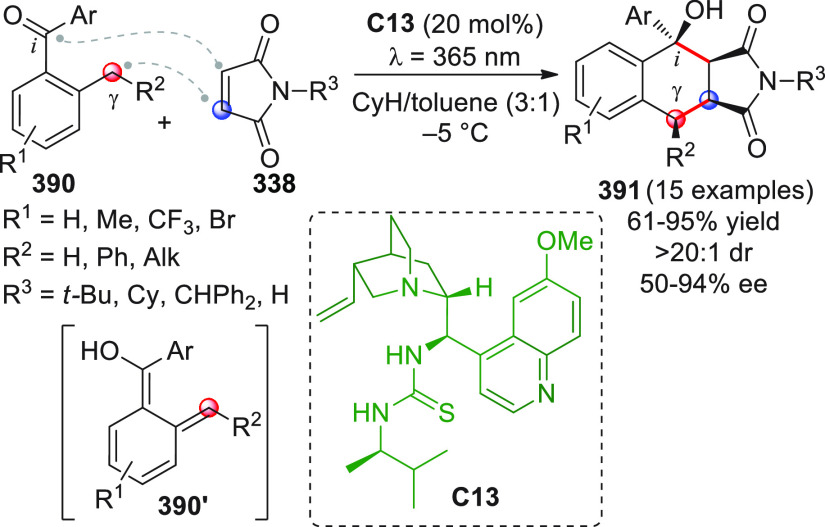


In 2016, Melchiorre et al. developed the first enantioselective catalytic variant of a Diels–Alder reaction between transient photoenol *o*-quinodimethanes **390′**, obtained by light irradiation at 365 nm of 2-alkyl benzophenones **390** (for the mechanism, see Scheme 73), and maleimides **338** (Scheme 106).^284^ The reaction was catalyzed by the bifunctional thiourea-amine **C13** in a mixture of cyclohexane and toluene at −5 °C. With these experimental conditions, excellent results were obtained, above all by considering the high challenge of performing an enantioselective catalytic version of a photoactivated reaction; tetrahydronaphthalenols **391** were formed in good yields, with high enantioselectivity and exquisite diastereoselectivity. The generality of the method was demonstrated since there was a wide tolerance for substituents on the benzophenone derivatives **390** as well as on the maleimide acceptors. Only the *N*-unprotected maleimide (R^3^ = H) afforded the product with low enantioselectivity (50% ee). The authors carried out experiments to elucidate the role of the catalyst, discovering that the quinuclidine core and thiourea moiety of the catalyst **C13** exert two opposite yet cooperative roles. The quinuclidine core interferes with the photoenolization mechanism, acting as an inhibitor of the photoenol Diels–Alder, while the thiourea moiety increases the dienophilic character of **338** upon H-bonding activation and indeed acts as a chiral catalyst, channeling the reaction toward the enantioselective pathway.

**Scheme 107:**
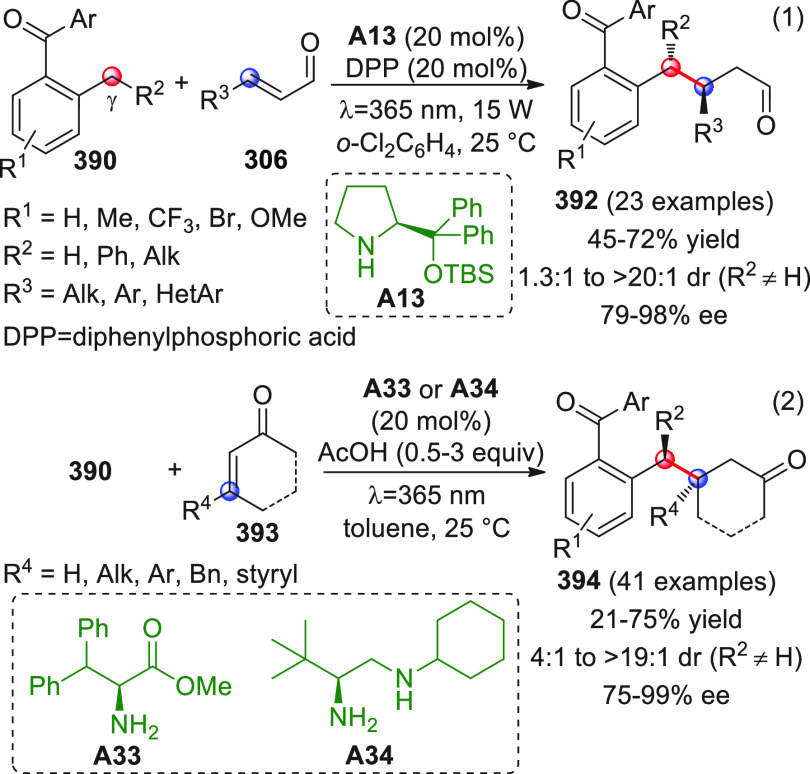


The following year, the same photoenol *o*-quinodimethanes **390′** were used as the nucleophilic components in enantioselective organocatalytic VMcRs. The Melchiorre group, in particular, developed organocatalytic β-benzylation of enals **306** (Scheme 107, eq 1),^285^ presenting this reaction as the first effective enantioselective catalytic variant of the photoenolization/Diels–Alder sequence. The enals were activated as iminium ions by the diphenylprolinol *tert*-butyldimethylsilylether **A13** in 1,2-dichlorobenzene, and the addition of diphenylphosphoric acid as acidic additive was useful to increase the reaction yield. The transformation furnished compounds of type **392** in fair yields and good enantioselectivities. Different aliphatic groups at the β-positions of the enals proved to be viable substituents, as well as different substituents on both aromatic rings of **390** were well tolerated. Interestingly, Diels–Alder adducts were never detected, so the authors carried out density functional theory (DFT) studies to explain why Michael adducts were preferred over cycloaddition products. The studies suggested the importance of a water molecule as a proton shuttle to transfer a proton by the photoenol to the iminium ion nitrogen, favoring the formation of an intermediate that selectively carries to the Michael product.

Another example of enantioselective Michael addition of photogenerated *o*-quinodimethanes **390′** (derived from benzophenones **390**) to enones **393** was reported by Ye and collaborators (Scheme 107, eq 2).^286^ The reaction was catalyzed by aminoester **A33** in toluene, using acetic acid as an additive (in the absence of acid no products were formed). With these optimized conditions, different cyclohexenones as well as linear α,β-unsaturated ketones **393** were intercepted by the photoenol species **390′**, to give adducts **394** in good yields and excellent enantioselectivities. The reaction proved quite general and was successfully applied to 3-substituted 2-cyclohexenones, providing asymmetric access to products bearing all-carbon quaternary centers. Instead, for the reaction with cyclopentenone, it was necessary to change the catalyst by using **A34**, which consigned the adducts in low yields, but with excellent enantioselectivities.

**Scheme 108:**
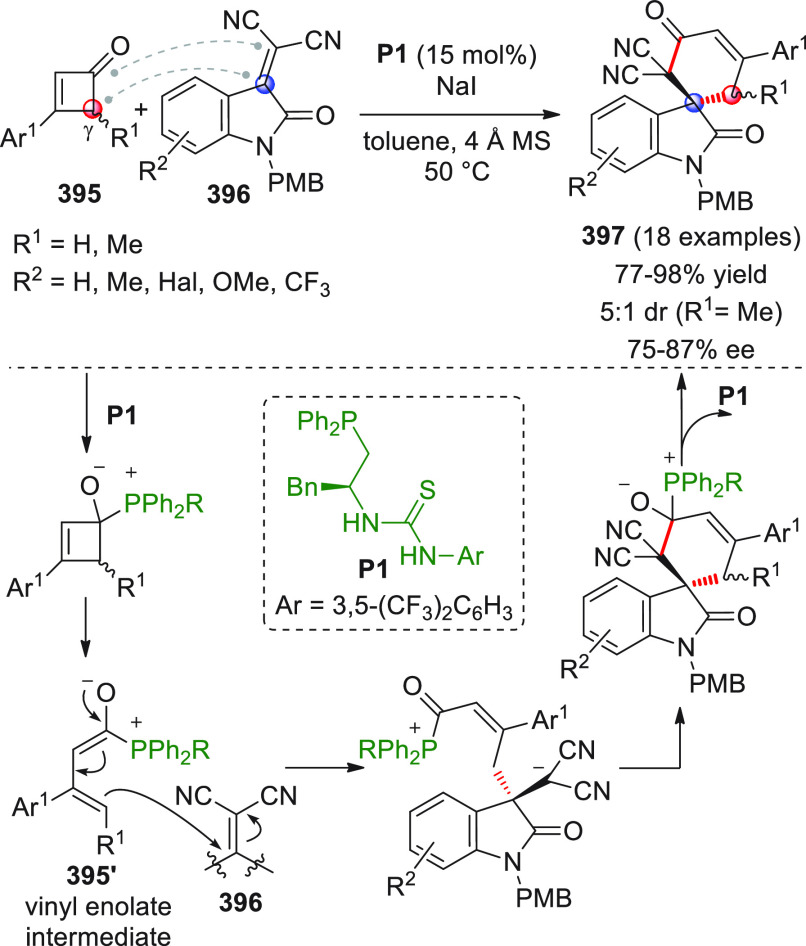


The organocatalytic activation of cyclobutenones through the addition of an organocatalyst to form a vinylenolate intermediate by cleavage of C–C bond was described in 2015 at the same time and, independently, by Chi et al. (see Scheme 77)^252^ and Zhang and co-workers.^287^ In place of an NHC catalyst, exploited by the former group, Zhang explored the use of chiral bifunctional phosphine **P1**, which in the presence of cyclobutenone **395** readily undergoes C–C bond cleavage forming the 1,4-dipolar linear intermediate **395′**, able to react as a vinylogous Michael donor on isatylidenemalononitriles **396** (Scheme 108). The reaction is a 1,4-dipolar spiroannulation that provided enantioenriched 3-spirocyclohexenone 2-oxindoles of type **397** in good yield and moderate to good enantiomeric excesses. The strong double H-bond donor moiety (3,5-di(trifluoromethyl)phenylthiourea) in the catalyst **P1** was fundamental for the good enantioselectivity; in fact, phosphine-derived catalysts with single H-bond donors were not able to give good stereocontrol. Even the NaI additive was demonstrated to be important to increase enantiomeric excess values.

#### 4.3.2 Indirect Procedures

##### 4.3.2.1 Acyclic Nucleophiles

**Scheme 109:**
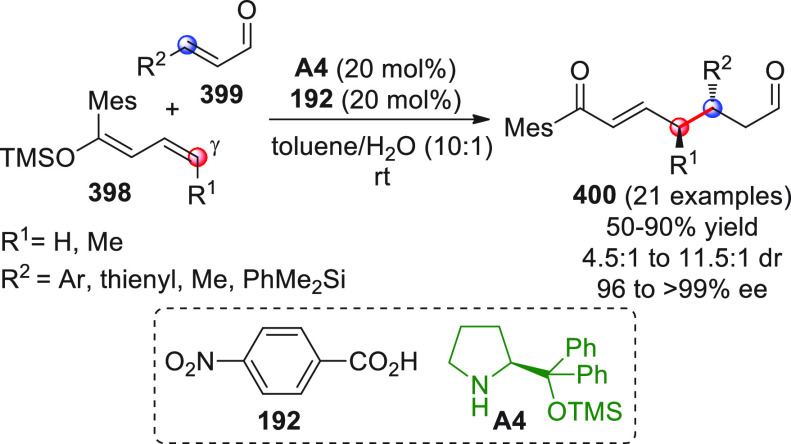


The sole example of preformed linear silyl dienol ethers derived from ketones as nucleophiles toward activated C=C bonds was reported by Schneider et al. in 2012.^288^ The reaction is a catalytic, enantioselective vinylogous Mukaiyama–Michael addition of silyl dienol ethers **398** to α,β-unsaturated aldehydes **399**, that provided chiral 1,7-dioxo compounds **400** (Scheme 109). The catalytic strategy exploited the conversion of enals into more reactive chiral α,β-unsaturated iminium ions, using prolinol-derived catalyst **A4**, together with *p*-nitrobenzoic acid **192** as cocatalyst. Compounds **400** were synthesized in good yields, and with excellent enantioselectivities, while the level of diastereoselection, measured when R^1^ = Me, was not particularly gratifying. The sterically demanding mesityl group on the carbonyl in **398** was fundamental to shield the α-position and obtain high levels of regiocontrol (γ- vs α-attack). In fact, when the phenyl was present in place of the mesityl group, high percentages of α-1,4-regioisomers were isolated.

##### 4.3.2.2 Cyclic Nucleophiles

**Scheme 110:**
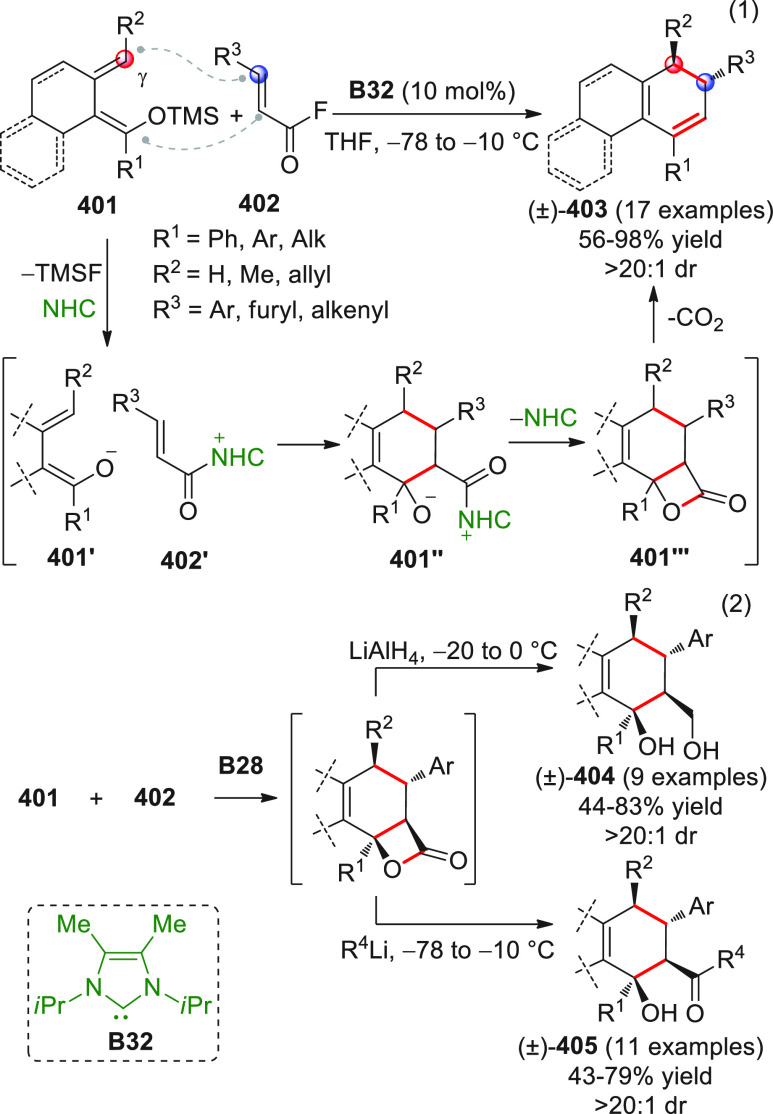


In 2011, Lupton and colleagues reported the first all-carbon NHC-catalyzed [4 + 2] cycloaddition between silyl dienol ethers of type **401** and α,β-unsaturated acid fluorides **402** to produce racemic 1,3-cyclodienes **403** (Scheme 110, eq 1), in moderate to excellent yields and with complete diastereocontrol.^289^ The NHC catalyst **B32** furnished acyl azolium intermediates **402′**, by nucleophilic substitution of the acyl fluorides, that reacted as Michael acceptors^290^ with the dienolates **401′**. The Michael addition followed by an intramolecular aldol reaction furnishes the [4 + 2] cycloadducts **401′′**, that release the NHC catalyst following lactonization reaction. Finally, the decarboxylation of intermediates **401′′′** provides the final cyclohexenes products **403**. This strategy was not applicable to aldehyde-derived TMS dienol ethers or to those derived from acyclic ketones, because in both cases *O*-acylation was favored. The studies to elucidate the mechanism reaction^291^ confirmed that the intermediate **401′′** is formed by a stepwise mechanism and not via a concerted cycloaddition. Moreover, the β-lactone intermediate **401′′′** was revealed to be stable until −20 °C and could be reductively intercepted with organolithium reagents to furnish racemic compounds **404** and **405**, respectively, bearing four contiguous stereocenters (Scheme 110, eq 2).

### 4.4 Other Reactions

#### 4.4.1 Direct Procedures

##### 4.4.1.1 Cyclic Pronucleophiles

**Scheme 111:**
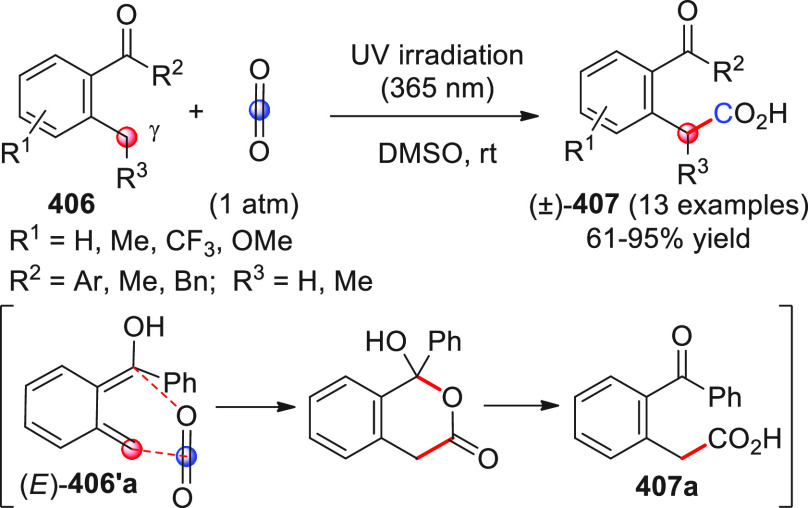


The vinylogous reactivity of photoenol (*E*)-**406′a**, generated from *o*-alkylphenyl ketones **406** via UV irradiation at 365 nm, was exploited by Murakami et al. for a vinylogous carboxylation reaction with CO_2_ (1 atm) to form *o*-acylphenylacetic acids **407** (Scheme 111).^292^ The reaction was clean since it did not need any catalyst or additional reagents but the simple irradiation of the starting materials in DMSO. The light energy that brings the formation of the highly reactive intermediate (*E*)**-406′a** is the driving force of the process. The authors proposed a mechanism in which the (*E*)**-406′a** undergoes a [4 + 2] cycloaddition reaction with the C–O double bond of CO_2_ to afford a six-membered cycloadduct, that collapses to the final carboxylic acid by a ring opening reaction; indeed the reactivity of CO_2_ as a dienophile is unprecedented.

**Scheme 112:**
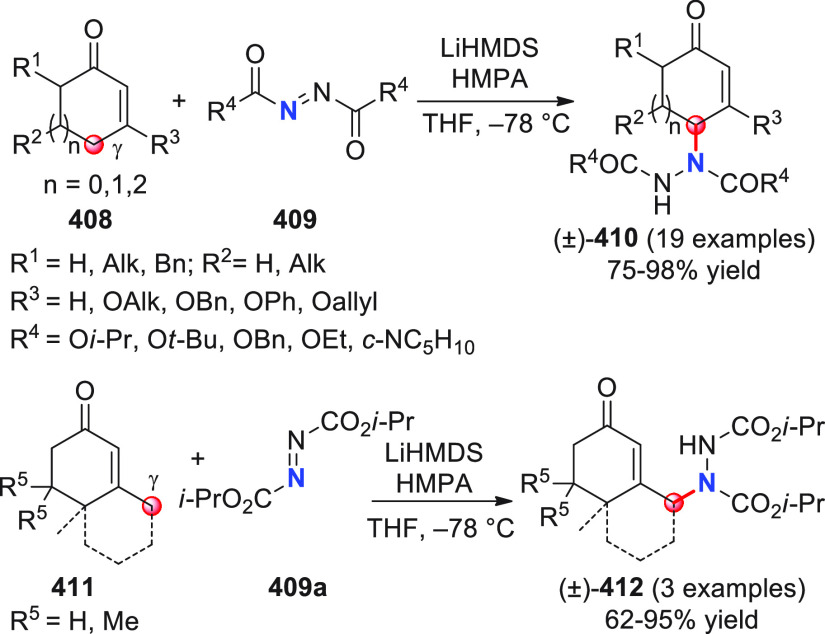


γ-C–N bond formation via dienol or dienolate is a quite uncommon strategy, even because the α-amination of dienolate intermediates is often privileged.^293^ In fact, the first example of a direct γ-regioselective amination protocol of enones was reported by Mohr and colleagues only in 2015.^294^ The reaction of conjugated ketones **408**, treated in THF at −78 °C with lithium hexamethyldisilazide (LiHMDS) to generate the dienolate, with dialkylazodicarboxylates **409** as acceptor counterparts, furnished the γ-aminated racemic products **410** (Scheme 112). A polar additive, such as hexamethylphopshoramide (HMPA), was fundamental to obtain high levels of regioselectivity, even if the reason was not fully clarified. Enones lacking the alkoxy group at the β-position (**411**) were compatible with the protocol and provided the corresponding aminated products (±)-**412** in good yields. The reaction was also successfully tested on linear enones, conjugated esters, and lactones.

## 5 Vinylogous Esters and Lactones

Chiral, enantiopure α,β-unsaturated esters, be they linear or cyclic, are one of the most widespread classes of chiral frameworks, constituting the structural core of a vast set of natural products which display an impressive range of biological activities important for the development of novel physiological and therapeutic agents.^35^ Furthermore, the possibility to easily access remotely enolizable esters and higher-order homologues by synthesis renders this compound class a preeminent source of vinylogous and hypervinylogous carbon nucleophiles to be exploited in fruitful additions to differently adorned electrophiles thus providing access to a vast plethora of chiral products. In this context, due to the high versatility of this class of compounds and the outburst of research fields such as organocatalysis and green chemistry, the past decade has witnessed, along with the development of a vast array of new asymmetric, vinylogous transformations, the application of older methodologies to the total synthesis of increasingly complex, bioactive compounds.^34^

**Figure 3:**
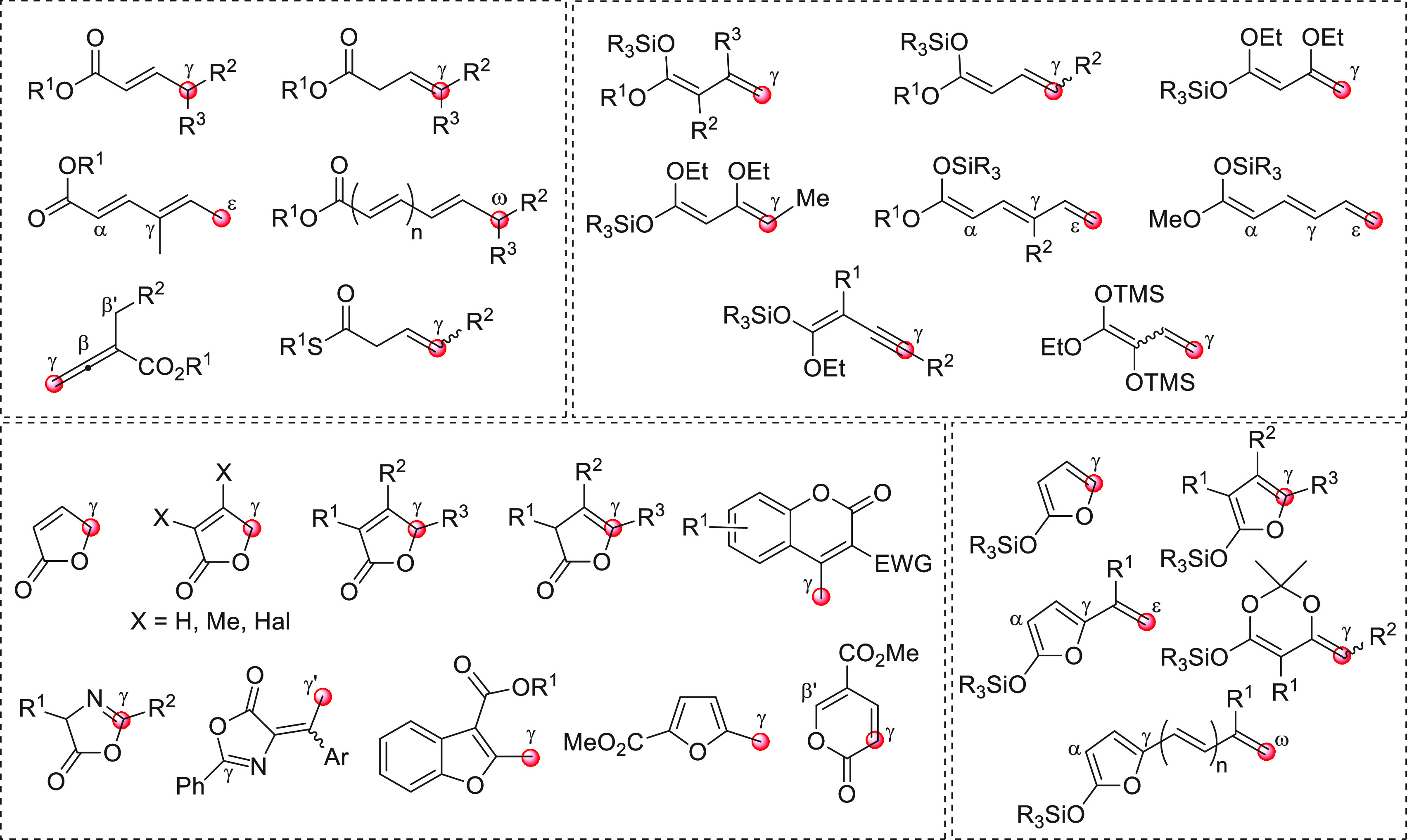
Collection of acyclic (above) and cyclic (below) (pro)nucleophilic esters at work in this section using direct and/or indirect procedures. Red circles denote the reactive (pro)nucleophilic carbon sites.

As for the previously described functionalities, a picture of the starting (pro)nucleophilic ester and lactone structures reported in this section is outlined in Figure 3, subdivided between acyclic (top) and cyclic (bottom) representatives and marked in their reactive (pro)nucleophilic remote sites.

### 5.1 Additions to C=O Bonds

#### 5.1.1 Direct Procedures

##### 5.1.1.1 Acyclic Pronucleophiles

**Figure 4:**
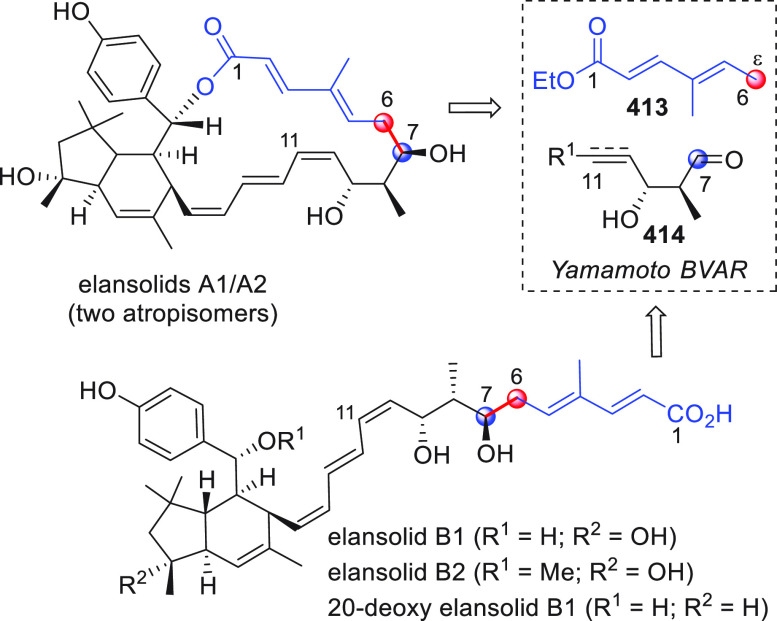
Kirschning’s disconnection of the C6–C7 linkage of elansolids, envisaging a Yamamoto bisvinylogous aldol reaction (BVAR).

Starting from 2012, as part of their total synthesis program toward several members of the antimicrobial family called elansolids (Figure 4), the Kirschning’s group developed and exploited a direct, diastereoselective, and substrate-controlled Yamamoto bisvinylogous aldol reaction (BVAR) between (2*E*,4*E*)-ethyl-4-methylhexa-2,4-dienoate (**413**) and suitable chiral aldehydes of type **414** (Figure 4).^295^ The core structure of these natural polyketides features a bicyclo[4.3.0]nonane unit embedding a chiral polyene chain resulting in a macrolactone core in elansolids A1 and A2 or its linear *seco*-acid form in elansolids B1 and B2. Concerning the construction of the chiral polyene chain, Kirschning planned to use the (2*S*,3*S*) chirality within aldehydes **414** (representing the fragment C7–C11) to control the absolute *R*-configuration of the newly formed stereogenic center at C7, with the aid of the Yamamoto protocol.^296−298^

**Scheme 113:**
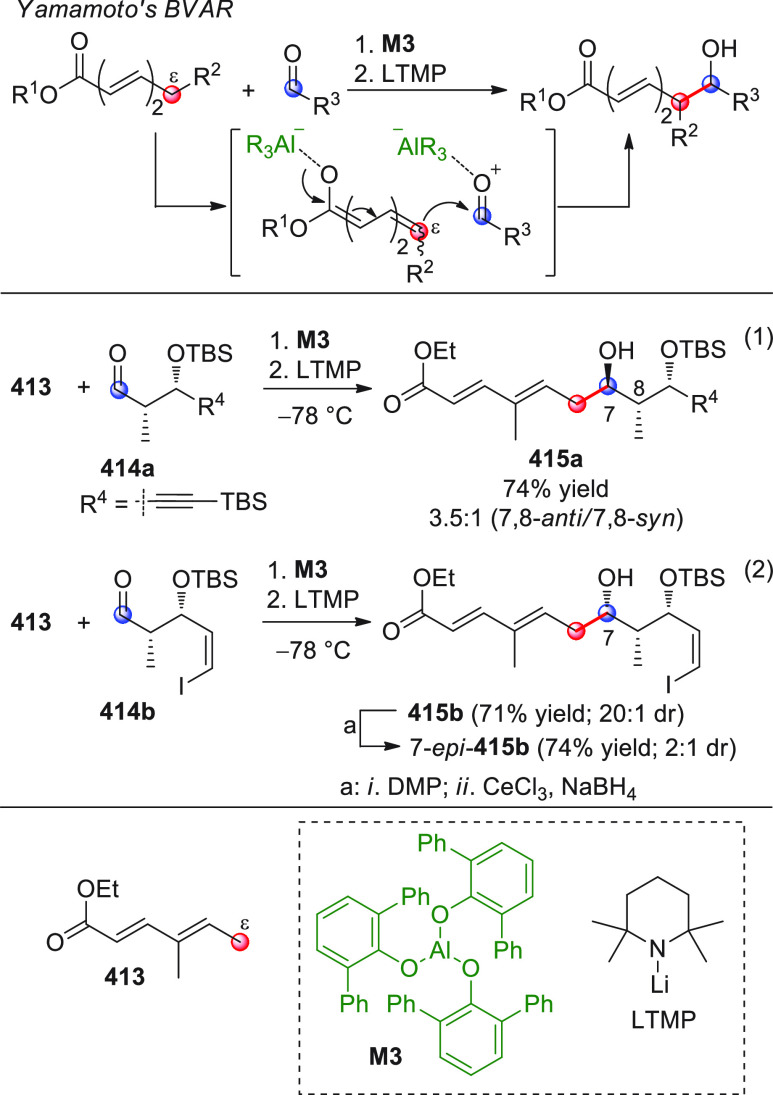


The Yamamoto bisvinylogous aldol reaction (BVAR) relies on the use of a bulky Lewis acid such as the *C*_*3*_-symmetric aluminum-tris-2,6-diphenylphenoxide **M3** (ATPH) and lithium tetramethylpiperidine (LTMP) as the base (Scheme 113). Commonly, precomplexation of both the enolizable polyunsaturated ester compound and the aldehyde with 2.2 equiv of **M3** at −78 °C is followed by the addition of an ethereal solution of stoichiometric LTMP that remotely deprotonates the ester to give an activated trienolate nucleophile that couples to the aluminum-precomplexed aldehyde via a bisvinylogous aldol reaction at the remote ε-terminus of the chain (Scheme 113, top). After aqueous work up, the expected homoallyl alcohol is provided. An extensive mechanistic study surveying differently substituted 2,3-*syn* and 2,3-*anti*-configured starting aldehydes **414** allowed Kirschning to elect silyloxy protected alkynyl (2*S*,3*S*)-**414a** as the best aldehyde candidate to directly access the 11-carbon long chiral polyene chain with the correct 7,8-*anti* relationship found in the elansolids.^295^

Indeed, the reaction between unsaturated ester **413** and (2*S*,3*S*)-**414a** under the standard Yamamoto BVAR conditions at −78 °C afforded the *anti*-Felkin adduct **415a**, with a 74% yield and a dr (7,8-*anti*/7,8-*syn*) of 3.5:1 (Scheme 113, eq 1). More recently,^297^ in the effort to construct the eastern fragment of 20-deoxy-elansolid B1 (Figure 4), the same group obtained the desired 7,8-*anti* adduct indirectly, by exploiting a highly 7,8-*syn* diastereoselective Yamamoto BVAR between dienoate **413** and a iodovinyl aldehyde (2*S*,3*S*)-**414b** (Scheme 113, eq 2), accessing the 7,8-*syn* Felkin adduct **415b** (71% yield, 20:1 dr). The C7 chiral inversion of **415b** was then afforded by Dess–Martin oxidation-Luche reduction sequence, yielding a separable mixture of diasteoisomers in favor of 7-*epi*-**415b** (2:1 ratio). The procedure was then repeated with the undesired diastereoisomer, which finally allowed them to collect the 1,3-*anti* diastereoisomer 7-*epi*-**415b** in a combined 74% yield.

**Scheme 114:**
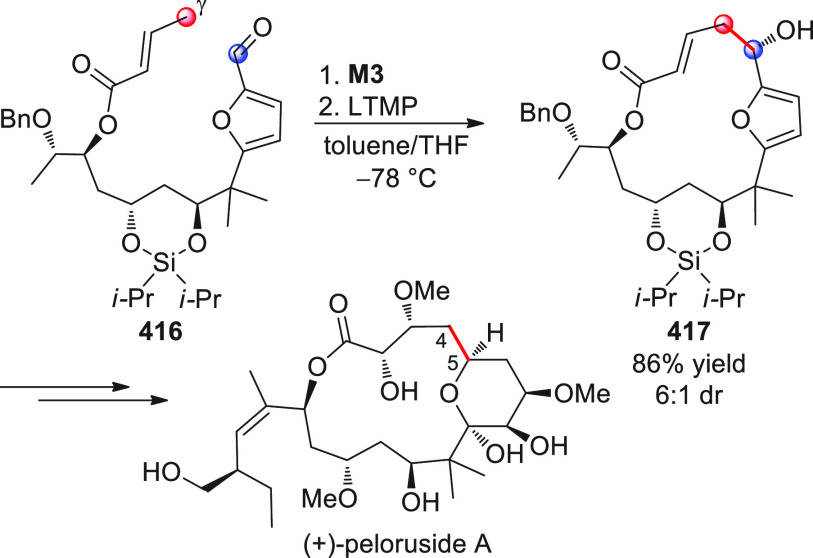


An intramolecular version of the Yamamoto VAR was exploited by Sammakia and co-workers in 2012 for the preparation of an advanced intermediate for the synthesis of (+)-peloruside A, a polyoxygenated 16-membered macrolide with potent antimicotic activity (Scheme 114).^299^ The reaction was applied to open intermediate **416** bearing pronucleophilic crotonate and nonenolizable furfural moieties that were cyclized using ATPH (**M3**, 2.2 equiv) and LTMP (2.0 equiv) in toluene/THF at −78 °C. The corresponding 16-membered macrolactone **417** was accessed in 86% yield as a 6:1 diastereomeric mixture.

**Scheme 115:**
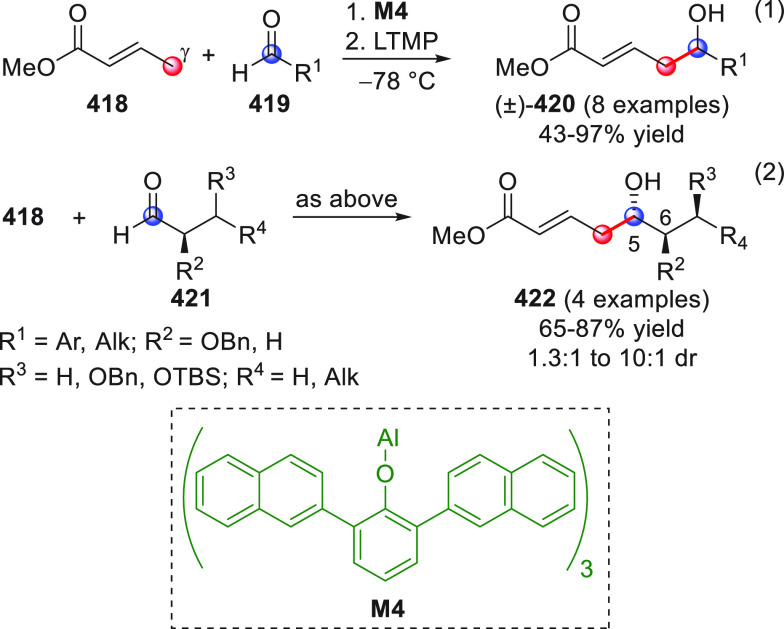


The Sammakia’s group also developed a new aluminum-based Lewis acid, namely, aluminum tris(2,6-di-2-naphthylphenoxide) (ATNP, **M4**), capable of promoting the crossed Yamamoto VAR between methyl crotonate **418** and enolizable aldehydes of type **419** and **421** (Scheme 115, eqs 1 and 2).^300^ Indeed, the bulkier 2-naphthyl groups within **M4**, blocking the α-enolization of the aldehyde component, enabled the regioselective vinylogous enolization of the crotonate ester. As described in Scheme 115 (eq 1), using 3.3 equiv of **M4**, the direct, vinylogous aldol reaction between **418** and a series of achiral aliphatic and aromatic aldehydes of type **419** afforded the corresponding γ-adducts (±)-**420** in good yields (up to 97%). Furthermore, under similar reaction conditions, addition of **418** to chiral α- and β-substituted aldehydes of type **421** (eq 2) proved also viable, providing the corresponding 5,6-*anti*-configured adducts **422** in good yields and stereoselectivity (up to 87% yield and up to 10:1 dr).

An attractive alternative to the common activation of linear unsaturated ester pronucleophiles via acid- or base-catalyzed conversion into their active (*poly*)enolate form resides in the generation of NHC-bonded dienolates (also called vinyl enolates) obtained by reacting the ester precursor with an active form of a suitable chiral *N*-heterocyclic carbene (NHC) precatalyst (vide previous sections). The annulation of these NHC-bonded vinyl enolates with substrates containing polar C–O and C–N double bonds paved new avenues to a variety of highly functionalized molecules.

**Scheme 116:**
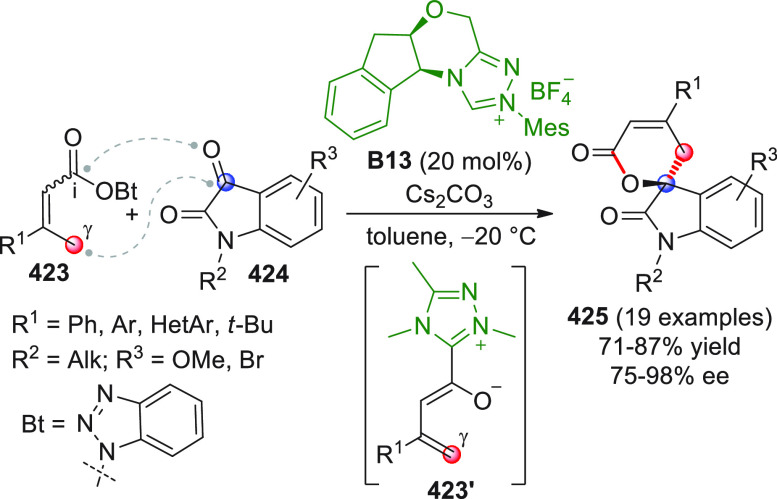


In this context, a recent example for the asymmetric synthesis of spirocyclic heterocycles was developed by Yao and co-workers in 2015 (Scheme 116).^301^

Starting from readily available, linear α,β-unsaturated benzotriazole (Bt) esters of type **423** (Scheme 116), a formal NHC-catalyzed [4 + 2] cycloaddition reaction to *N*-alkyl isatins of type **424** was devised providing a series of chiral, enantioenriched spirocyclic oxindole dihydropyranones **425** featuring a tetrasubstituted carbon stereocenter. Indeed, a suitable optimization survey elected chiral triazolium **B13** as the most effective precatalyst in promoting the reaction between **423** and **424** in toluene at −20 °C, in the presence of 1.2 equiv of Cs_2_CO_3_, providing the corresponding γ,i-locked [4 + 2] cycloadducts **425** in good yields (up to 87%) and enantioselectivities (up to 98% ee). The reaction proved to be quite general as it worked well on diverse benzotriazole 3-methylcinnamate derivatives and 5-methoxy or 4-Br-*N*-alkyl isatins.

##### 5.1.1.2 Cyclic Pronucleophiles

The enantioselective VAR involving either α,β-unsaturated (e.g., furan-2(5*H*)-one) or β,γ-unsaturated furanone (e.g., α-angelicalactone) donors and chiral/achiral aldehyde acceptors has attracted particular attention in the chemical community being one of the most efficient tools to obtain chiral, γ-substituted, and γ,γ-disubstituted butenolides.

**Scheme 117:**
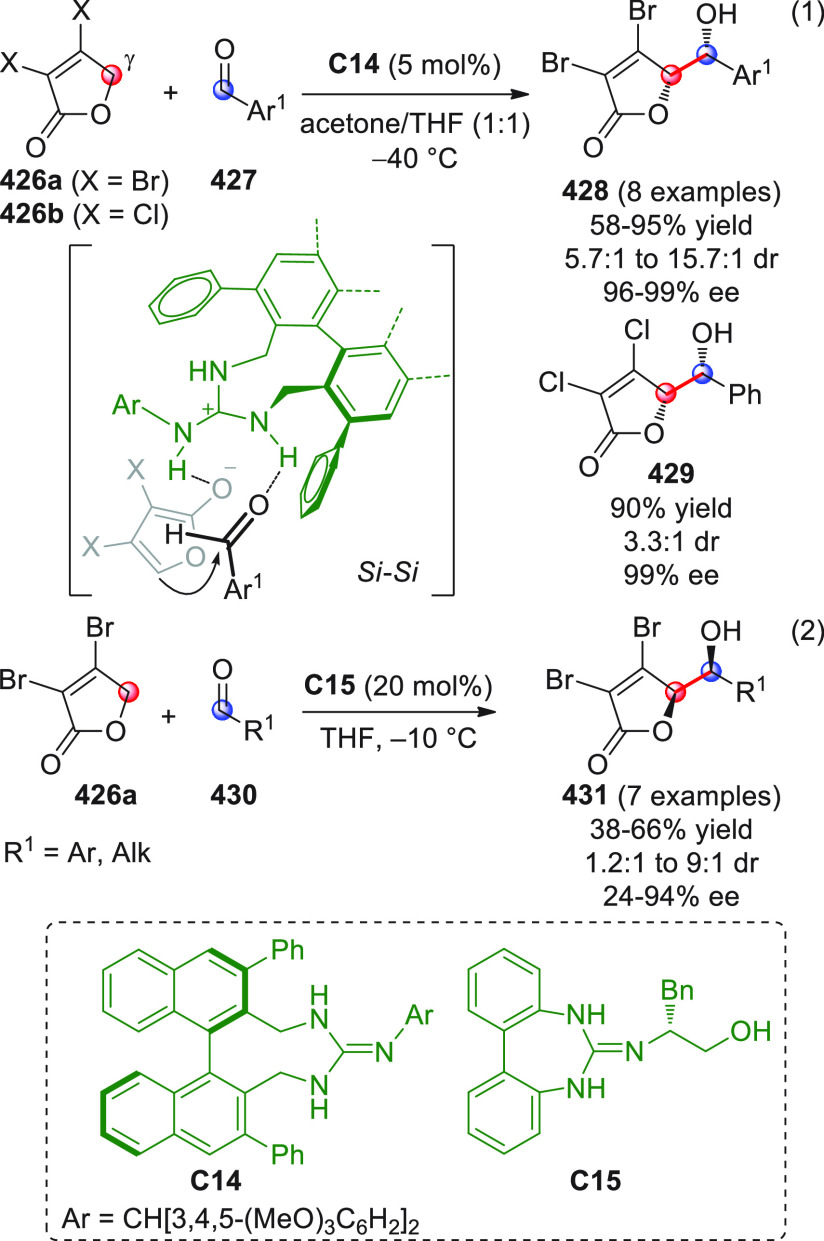


The first enantioselective, organocatalytic, and direct VAR reaction between dihalofuran-2(5*H*)-ones and aldehydes to give polyfunctionalized butenolides was reported by Terada’s group in 2010, using an axially chiral guanidine derivative as the catalyst (Scheme 117, eq 1).^302^ Catalyst **C14**, featuring a guanidine functionality (bearing a sterically demanding benzhydryl substituent Ar embedded into an axially chiral binaphthyl core), was found to be the most efficient tool to promote the enantioselective VAR between pronucleophilic dibromofuranone **426a** and several differently substituted aromatic aldehydes of type **427**. The reaction, performed in a 1:1 mixture of acetone/THF at −40 °C for at least 5 h, yielded the corresponding γ-homologated (5*S*,1′*R*)-configured products **428** with acceptable to good yields (up to 95%), and high diastereo- and enantiocontrol (up to 15.7:1 dr, up to 99% ee). Also dichlorinated furanone **426b** proved to be a viable substrate in the VAR addition to benzaldehyde, yielding the corresponding, enantiopure adduct **429** with a high 90% yield even though the diastereocontrol was somehow hampered (3.3:1 *syn*/*anti*) by the less steric bias of the chlorine atoms with respect to bromine. To account for the observed stereoselectivity, a transition state was proposed, in which the guanidinium ion orchestrated the *Si–Si* approach of the dienolate to the aldehyde by hydrogen-bonding interactions.

More recently, Dudding and co-workers^303^ reported the development of a new, chiral, computationally designed seven-membered ring guanidine **C15** as an effective organocatalyst for the asymmetric VAR between dibromofuranone **426a** and a small panel of aromatic and aliphatic aldehyde acceptors **430** (Scheme 117, eq 2). The reaction carried out in THF at −10 °C yielded optically active, dibrominated γ-butenolides **431** in low to moderate yields (up to 66%), with acceptable diastereo- and enantioselectivity in favor of the (5*R*,1′*S*)-configured isomers.

**Scheme 118:**
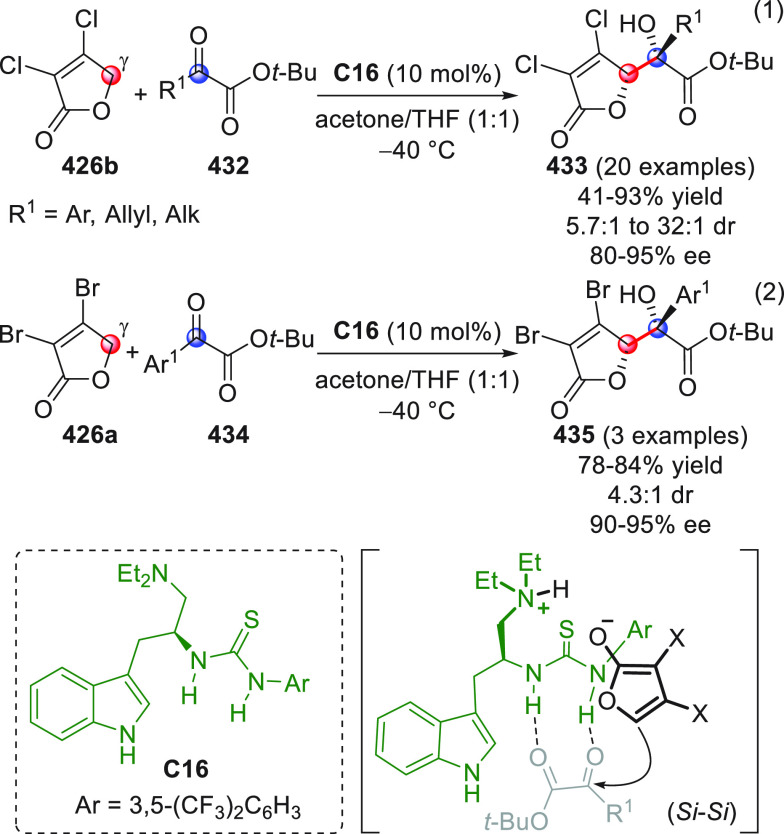


The fruitful versatility of 3,4-dihalofuranones of type **426a** and **426b** was also exploited in 2011 by Lu and co-workers,^304^ who reported a diastereo- and enantioselective, direct VAR toward α-ketoesters catalyzed by the tertiary amine-thiourea bifunctional organocatalyst **C16**, accessing enantioenriched γ-substituted butenolides with the creation of a quaternary stereocenter (Scheme 118). Indeed, after an extensive catalyst screening, the authors found that l-tryptophan-derived catalyst **C16** efficiently catalyzed the reaction between 3,4-dichlorofuran-2(5*H*)-one (**426b**) and a set of differently substituted *tert*-butyl glyoxylates of type **432** (Scheme 118, eq 1).

Consistently high diastereo- and enantioselectivities were observed for a wide range of aromatic, aliphatic, vinylic, and allyl-substituted α-ketoesters, accessing the corresponding γ-butenolides **433** in high yields with up to 32:1 dr in favor of the (5*S*,1′*S*)-configured products (up to 95% ee). Interestingly, the nonhalogenated furanone could also be used, although a much longer reaction time and a slightly lower enantioselectivity was observed (144 h; 69% yield, 10.1:1 dr, 82% ee, not shown). To account for the stereochemical outcome of the developed VAR, a transition-state was proposed (Scheme 118, bottom), highlighting the key role of hydrogen-bond interactions between the ketoester and the thiourea moiety of the catalyst orchestrating the selective attack of the C-γ of the dienolate to the *Si* face of the ketone, leading to the formation of the major stereoisomer. If the chlorine atoms were replaced by bulkier bromines as in **426a** (Scheme 118, eq 2), an overall increase in diastereoselectivity was observed, probably due to tighter ion pairing between the catalyst and the dienolate, as a result of higher electron density on the enolate oxygen atom.

**Scheme 119:**
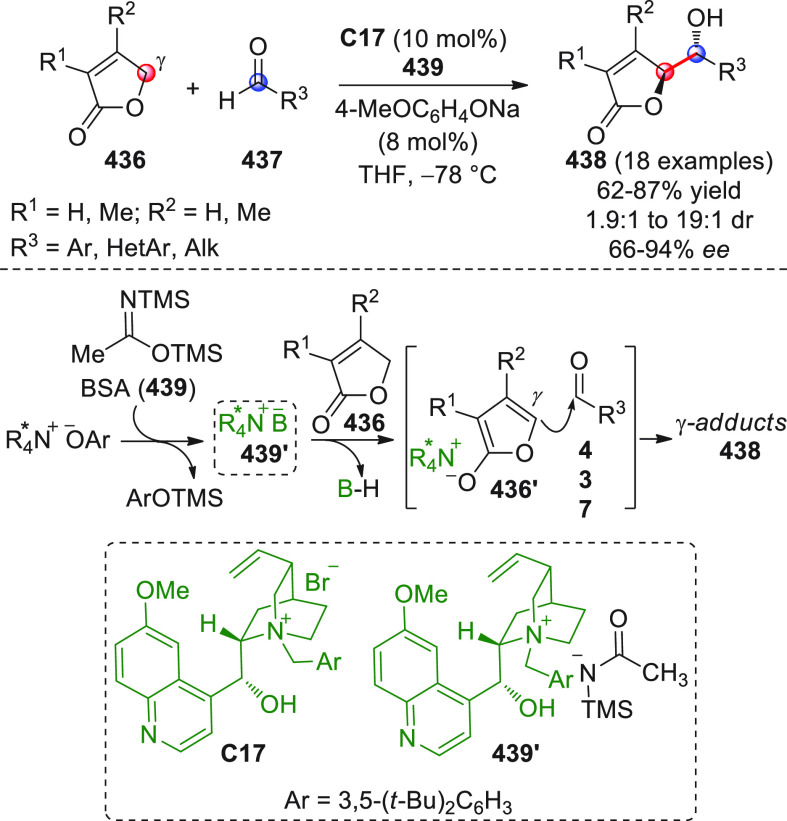


The just described direct VAR approaches involved the formation of active chiral quaternary ammonium dienolates as the key nucleophilic partners, as a result of the C-γ deprotonation of the dihalogenated (5*H*)-furan-2-ones operated by the catalyst. In this context, another attractive approach was proposed by Oudeyer, Levacher, and co-workers in 2013,^305^ who reported an asymmetric, direct, and *anti*-selective VAR of substituted (5*H*)-furan-2-ones of type **436** using a chiral quaternary ammonium aryloxide/*N,O*-bis(trimethylsilyl)acetamide (BSA, **439**) couple as promoters (Scheme 119). This strategy relies on the in situ formation of chiral salt **439′** obtained by the combination of a silylated base such as **439** with the quinine-derived chiral ammonium salt directly accessible via in solution ion methatesis between the corresponding ammonium bromide **C17** with sodium 4-methoxyphenoxide. The desired chiral quaternary ammonium dienolate **436′** is then obtained by the regioselective C-γ deprotonation of butenolide **436** which then may add to aliphatic or (hetero)aromatic aldehyde acceptors **437** to give the corresponding chiral δ-hydroxy butenolides **438** in good diastereomeric ratios (up to 19:1) and excellent enantioselectivities (up to 94% *ee*). Of note, despite the fact that unsubstituted (5*H)*-furan-2-one proved to be a viable pronucleophile giving the corresponding VAR adduct to benzaldehyde with comparable efficiency and stereoselectivity, the 4-methyl congener (not shown) reacted with benzaldehyde much less efficiently, with a poor 28% yield though with a good level of enantioselectivity (83% ee).

**Scheme 120:**
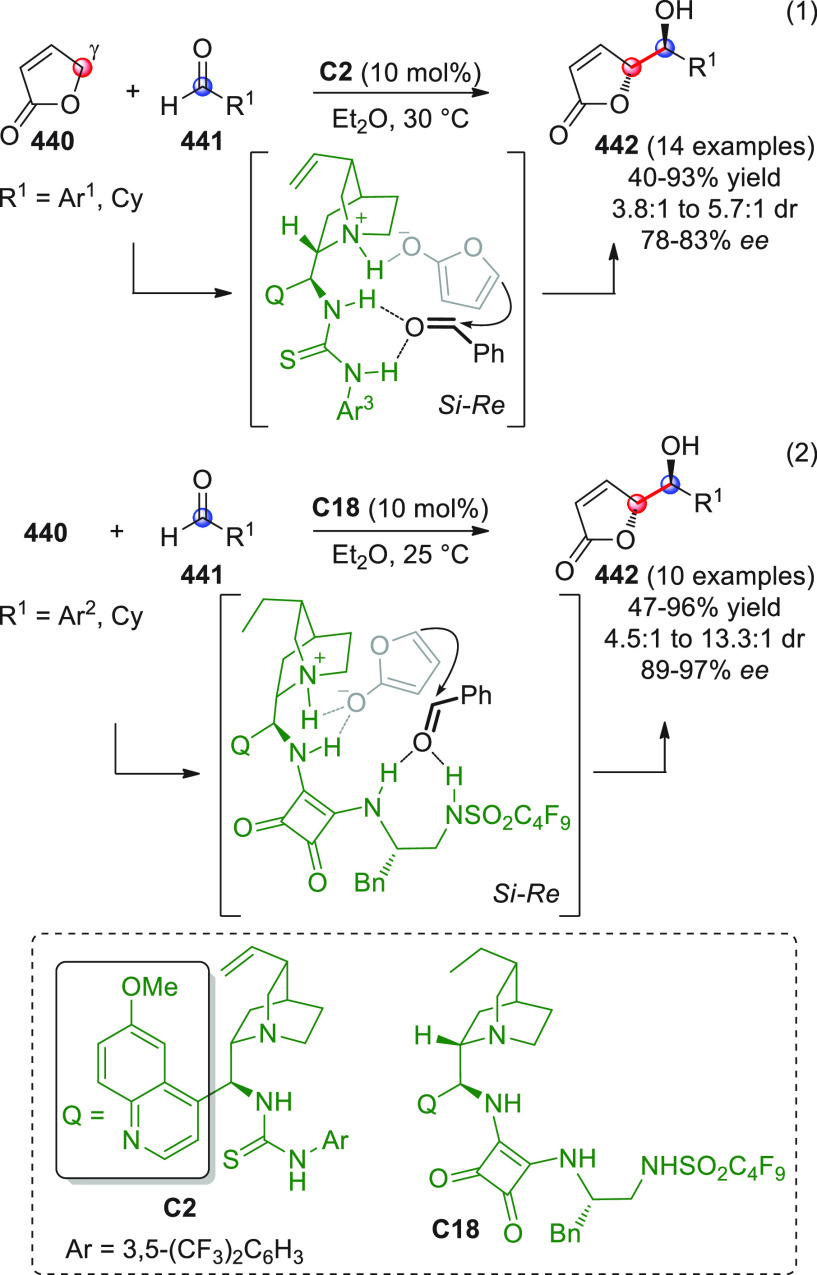


The first direct, organocatalyzed enantioselective VAR of an unsubstituted (5*H*)-furan-2-one using a cinchona alkaloid-based thiourea was achieved by Feng and co-workers in 2010 (Scheme 120, eq 1).^306^ After an extensive screening of bifunctional tertiary amine-thioureas, 9-*epi*-quinine thiourea **C2** was elected as the catalyst of choice to guide the reaction between (5*H*)-furan-2-one **440** and a panel of differently substituted aromatic aldehydes **441** (with the sole exception of aliphatic cyclohexyl carbaldehyde) in Et_2_O at 30 °C, to provide *anti*-configured butenolides (5*R*,1’*S*)-**442** with good yields (up to 93%) and satisfactory levels of diastereo- (up to 5.7:1 *anti*/*syn*) and enantioselectivity (up to 83% ee).

**Scheme 121:**
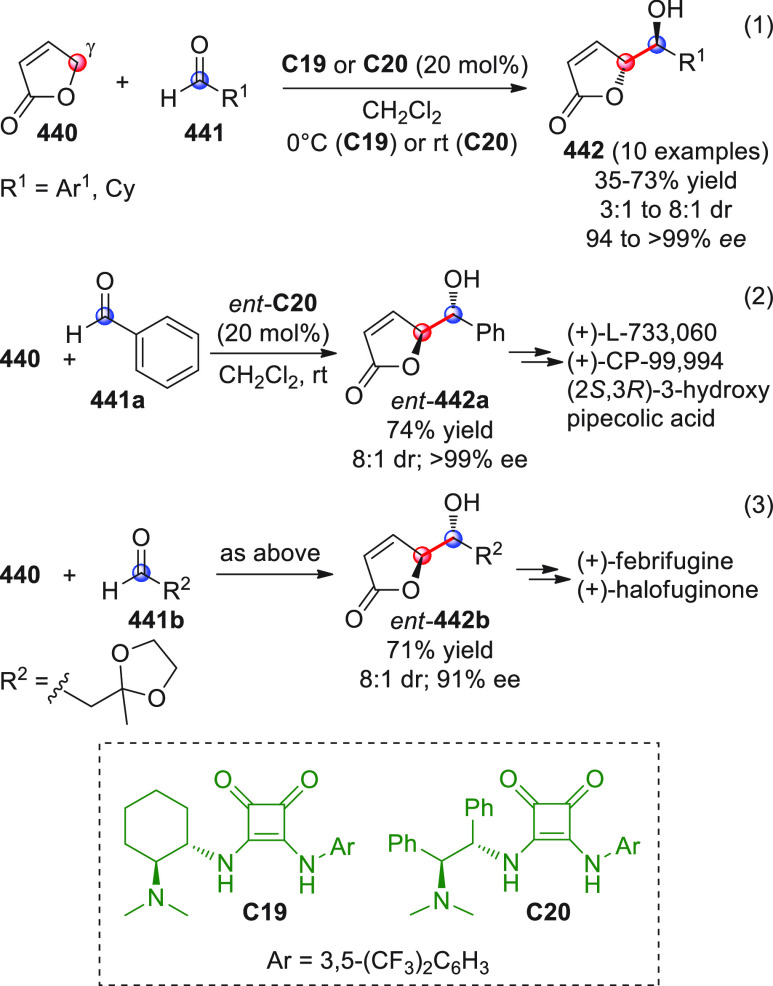


Inspired by Feng’s work, slightly better results were achieved by Hirashima and Miura in 2017 with a novel polydentate cinchona-squaramide organocatalyst **C18** bearing a chiral perfluorobutane sulfonamide group at one end (Scheme 120, bottom).^307^ Indeed, under the optimized reaction conditions (10 mol % catalyst **C18** in Et_2_O at rt), excess of pronucleophilic furanone **440** selectively added to a set of almost exclusively aromatic aldehydes of type **441**, affording the corresponding *anti*-configured-γ-butenolides **442** in high yields (up to 96%) and high to excellent diastereo- and enantioselectivities (up to 13.3:1 dr, up to 97% ee). Although the transition states of the above direct VARs are still to be completely elucidated, the rationalization of the observed high stereocontrol could be found in the H-bond networking these multidentate H-bond donor catalysts engage with both the dienolate and the aldehyde components in a stereodefined manner. In this context both groups proposed similar transition states, in which the aldehyde component is activated through a directed double hydrogen bonding operated by either the thiourea moiety in **C2** or the mixed squaramide-sulfonamide nitrogen atoms in **C18**; the quinuclidine moiety in the catalyst deprotonates furanone **440** to generate the corresponding dienolate intermediate whose selective attack to the *Re* face of the aldehyde is well orchestrated by the chiral backbone of both catalysts. The enantioselective, direct, VAR between furanone **440** and various aromatic aldehydes was also studied by Pansare and co-workers, who screened several bifunctional amino-thiourea and amino-squaramide organocatalysts to provide diastereomerically and enantiomerically enriched δ-hydroxy-5-substituted furanones of type **442** (Scheme 121, eq 1).^308^ Pansare’s survey unveiled the superior performance of the Rawal cyclohexanediamine squaramide **C19** and stilbenediamine squaramide **C20** over conventional aminothiourea catalysts, in accessing enantiopure, *anti*-configured butenolides **442** in good yields (up to 73%) and good diastereoselectivities (up to 8:1 dr). Subsequently, Pansare exploited the developed direct VAR to construct chiral, enantiopure δ-hydroxy butenolide adducts which served as the key starting materials in the total synthesis of several pharmaceutically relevant targets (Scheme 121, eqs 2 and 3).

In particular, benzaldehyde-derived adduct *ent*-**442a**, obtained from the direct VAR between **440** and benzaldehyde (**441a**) promoted by 20 mol % of squaramide *ent*-**C20** (Scheme 121, eq 2) was used in the preparation of neurokinin receptor antagonists (+)-L-733,0606, (+)-CP-99,994, as well as (2*S*,3*R*)-3-hydroxypipecolic acid.^309^ Dioxolane derivative *ent*-**442b**, instead, served as key intermediate for the synthesis of antimalarial agents (+)-febrifugine and (+)-halofuginone (Scheme 121, eq 3).^310^

Finally, the versatility of α,β-unsaturated γ-butyrolactones as powerful pronucleophiles in the direct and γ-selective aldol condensation to aldehydes was further demonstrated by several successful examples of organo- and metal-catalyzed procedures giving access to condensed γ-ylidene-butenolide frameworks: due to the nonasymmetric nature of such processes though, these examples will not be commented on in this review.^311−313,11^ Switching to deconjugated β,γ-unsaturated butenolides, only a few reports appeared with the direct, catalytic VAR to functionalized ketones, which can offer the opportunity to construct challenging tertiary alcohols with vicinal tetrasubstituted stereogenic centers.^314^

**Scheme 122:**
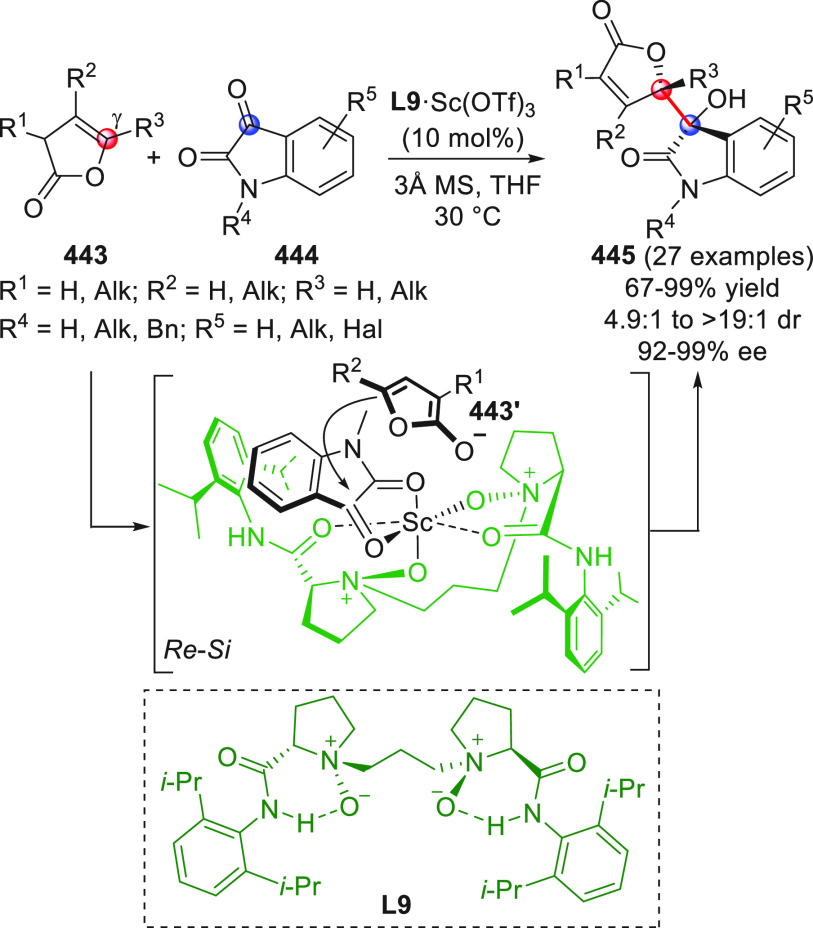


Despite the interest arisen about α-angelicalactones as vinylogous pronucleophiles,^35^ only recently Feng and co-workers reported the first asymmetric VAR of a series of β,γ-unsaturated butenolides of type **443** to polyfunctionalized isatins **444**, exploiting a *C*_*2*_-symmetric l-proline derived *N,N’*-dioxide ligand **L9** in combination of Sc(OTf)_3_ as the most suited catalytic system (Scheme 122).^315^ Indeed, the vinylogous addition between **443** and **444**, carried out in THF at 30 °C (for at least 72 h) in the presence of 3 Å MS, was perfectly orchestrated by the **L9**·Sc(III) complex, providing a series of enantiopure tertiary alcohols **445** containing not only the γ,γ-disubstituted butenolide motif but also the challenging tetrasubstituted oxindole framework. Based on the experimental investigations, a possible transition state was proposed in which **L9** binds to the central scandium ion in a tetradentate manner to form two stereodefined six-membered chelate rings (Scheme 122) that efficiently guide the *Re*-*Si* approach between the C-γ of the in situ formed dienolate **443′** to the C3 carbonyl of isatin **444**, affording products **445** with very high stereocontrol (up to >19:1 dr, and up to 99% ee).

**Scheme 123:**
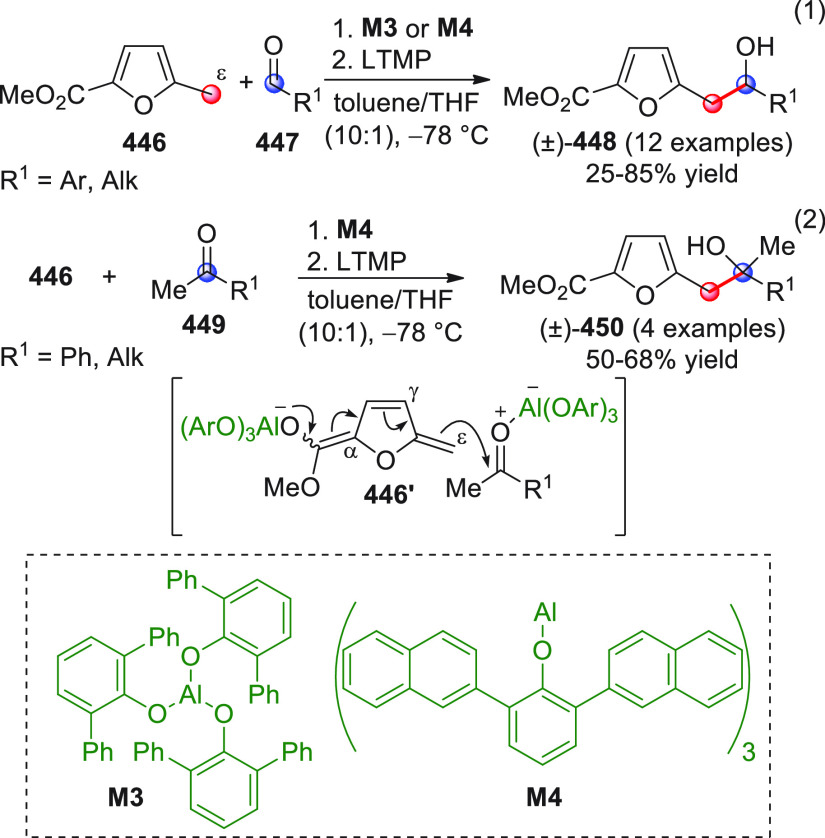


An interesting direct, bisvinylogous aldol reaction (BVAR) of methyl 5-methylfuroate (**446**) toward either aldehydes **447** or a small panel of methyl ketones **449** was developed by Sammakia et al. applying a modified version of the Yamamoto’s protocol (Scheme 123, eqs 1 and 2).^316^ Furoate **446** is a quite interesting pronucleophile, since deprotonation of its exocyclic methyl group to generate the trienolate intermediate **446′** may be hampered by the presence of the relatively stable furan ring between the methyl and the ester group. Furthermore, trienolate **446′** bearing a highly reactive 2,5-dimethylene-2,5-dihydrofuran moiety, might in principle add to the activated aldehyde or ketone at the competing α, γ, or ε position, posing the issue of the regiocontrol of the reaction. After a brief optimization survey, Sammakia found that ATNP (**M4**, 3.3 equiv) with lithium tetramethylpiperidine (LTMP, 3.5 equiv) at −78 °C in 10:1 toluene/THF, promoted the reaction between furoate ester **446** and aldehydes **447** or ketones **449**, to provide the corresponding aldol adducts (±)-**448** or (±)-**450** with moderate to high yields as the sole ε-adducts. (Interestingly, with several aliphatic, nonenolizable aldehydes, also Yamamoto’s bulky Lewis acid ATPH **M3** was an effective promoter providing the products in high yields.) Of note, the reaction proved to be viable to a wide range of functionalized carbonyl acceptors, so that aromatic and enolizable aliphatic aldehydes and methyl ketones reacted with furoate **446** under the optimized reaction conditions. β-Alkoxy chiral aldehyde acceptors were also tested (not shown), giving the corresponding aldol adducts in acceptable yields albeit with low dr.

**Scheme 124:**
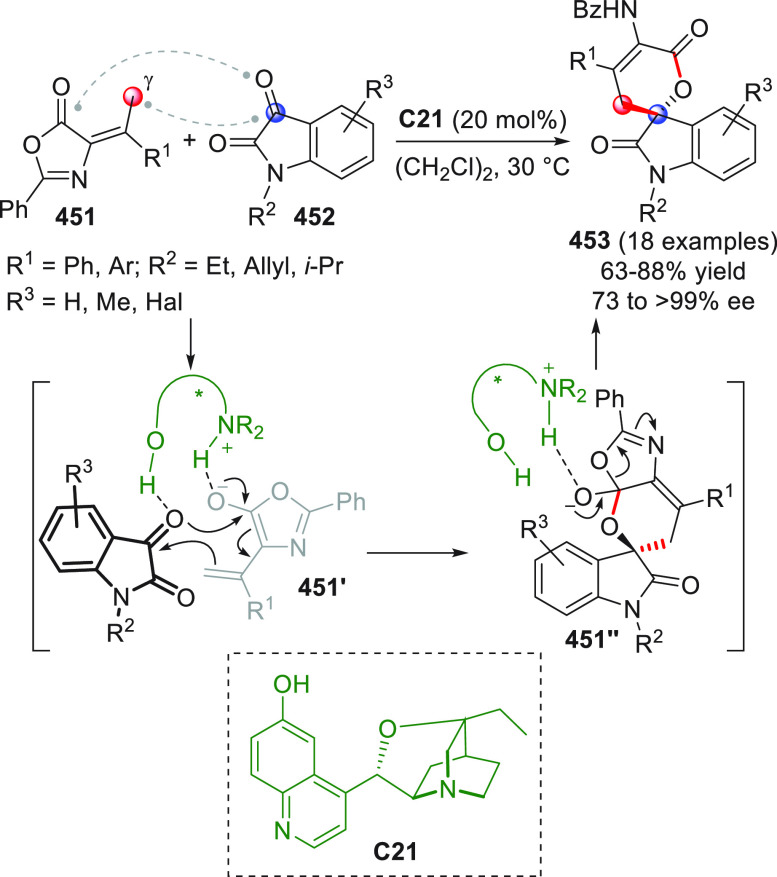


Among the varied repertoire of readily available cyclic lactone-type derivatives to be used as pronucleophilic vinylogous scaffolds in stereoselective transformations, azlactones (and in particular 4-alkylidene-oxazolones)^317^ are among the most useful starting materials for the synthesis of α-amino acids and heterocyclic scaffolds. However, until recently, the use of olefinic azlactones as pronucleophilic synthons in vinylogous aldol-type transformations remained elusive (vide infra). In this context, one of the few examples is given by the organocatalyzed, asymmetric formal hetero-Diels–Alder (HDA) reaction of olefinic azlactones of type **451** to isatins **452** achieved by Hu and co-workers via hydrogen-bond directed γ-addition (Scheme 124).^318^ This methodology provided an efficient access to *S*-configured spirooxindole dihydropyranones of type **453** in moderate to good yields (up to 88% yield) and excellent enantioselectivities (up to >99% ee). The reaction, catalyzed by bifunctional β-isocupreidine **C21** (β-ICD, 20 mol %) was carried out in 1,2-dichloroethane at 30 °C and was quite efficient irrespective of the nature and position of the substituents on both the azlactone and the isatin substrates. A mechanism was also postulated envisaging both reaction partners to be synergistically activated by the bifunctional catalyst **C21** to reach a stereodefined transition state in which a hydrogen-bonding network selectively guides the attack of azlactone dienolate **451′** on the *Si* face of isatin carbonyl. The formed aldolate adds then back to the azlactone carbonyl forming a [4 + 2] cycloadduct intermediate **451′′** which finally collapses to give the desired product **453** regenerating the catalyst.

**Scheme 125:**
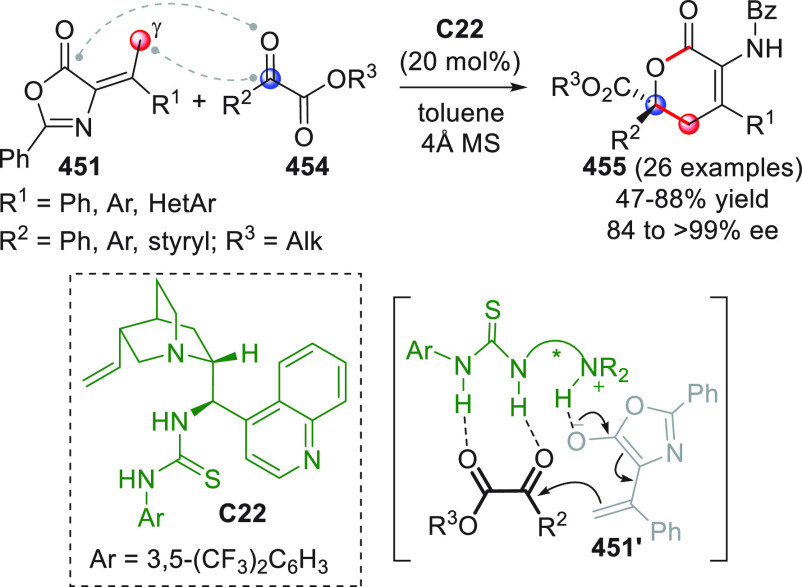


A similar protocol was reported some years later by the same group (Scheme 125),^319^ who developed an efficient asymmetric [4 + 2] annulation between alkylidene azlactones of type **451** and α-keto esters **454** using chiral cinchonine-thiourea **C22** as the bifunctional organocatalyst. Using this protocol, a wide range of the desired *S*-configured dihydropyranones **455** with a quaternary stereocenter were produced in generally good yields (up tp 88%) and excellent enantioselectivities (up to >99% ee). Here, the bifunctional thiourea catalyst enabled the synergistic activation of both reaction partners by forming a hydrogen bond-stabilized transition state in which the in situ formed dienolate **451′** was supposed to react on the *Re* face of the α-ketoesters **454** in a concerted or stepwise VAR to reach the desired product **455**.

#### 5.1.2 Indirect Procedures

##### 5.1.2.1 Acyclic Nucleophiles

**Scheme 126:**
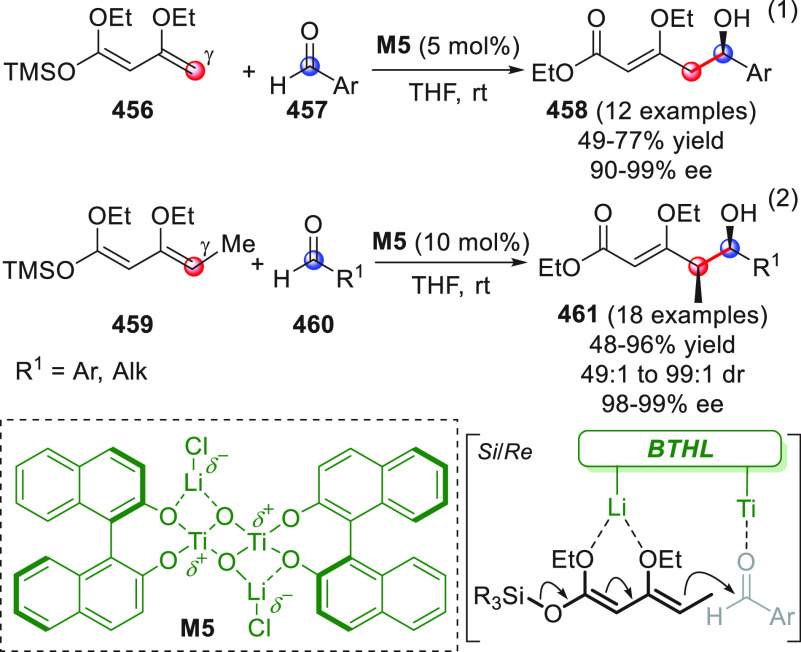


In 2010, Qu and co-workers reported the use of a novel BINOL–titanium–LiCl heterobimetallic complex (BTHL, **M5**, Scheme 126) as a useful catalyst to promote the enantioselective vinylogous Mukaiyama aldol reaction (VMAR) between Brassard’s diene **456** and a set of aromatic aldehydes of type **457** (Scheme 126, eq 1).^320^ In the optimized procedure, the catalytic system **M5** (5 mol %), generated in situ from (*R*)-BINOL, Ti(O*i*-Pr)_4_, H_2_O, and lithium chloride, carefully added in a 1:1:1:1 ratio, efficiently catalyzed the enantioselective VMAR affording the sole *Z*-configured (5*R*)-5-hydroxy-α,β-unsaturated ester derivatives **458** in good yields (up to 77%) and excellent enantioselectivities (up to 99% ee). Furthermore, VMAR addition of **456** to a more challenging aliphatic isobutyraldehyde (not shown) was also investigated, affording the corresponding adduct with 96% ee albeit with scarce efficiency (16% yield). A couple of years later,^321^ Qu exploited the same heterobimetallic catalytic system **M5** in the development of a highly enantio- and diastereoselective VMAR between prostereogenic propionyl acetate-derived diene **459** and a set of aromatic and aliphatic aldehydes of type **460** (Scheme 126, eq 2). The reaction, carried out in the presence of catalyst **M5** (10 mol %), yielded chiral, enantiopure δ-hydroxy-γ-methyl-β-methoxy acrylates of type **461** (up to 99% ee), in high yields (up to 96%) and almost complete diastereocontrol in favor of the *Z*-*syn* isomer (up to 99:1 dr). Of note, despite a strong positive nonlinear effect (NLE) being unveiled, suggesting a more complex active oligomeric titanium species in solution, a “Lewis acid assisted Lewis acid” model was proposed in which the lithium ion enhanced the Lewis acidity of the titanium center through associative interactions, by coordinating the oxygen of a μ-oxo bis-titanium complex, and allowing the metal to interact with the aldehyde carbonyl, discriminating its prochiral faces. Meanwhile, the diene would coordinate the weakly acidic lithium center in a chelating manner moving close to the aldehyde along a *Si–Re* trajectory (Scheme 126, bottom). Finally, this methodology was successfully exploited in the formal synthesis of polypropionate cystothiazole A and melithiazole C, a group of fungicidal β-methoxy (*E*)-acrylate antibiotics featuring a polypropionate chain attached to a thiazole or bis-thiazole core (not shown).

**Scheme 127:**
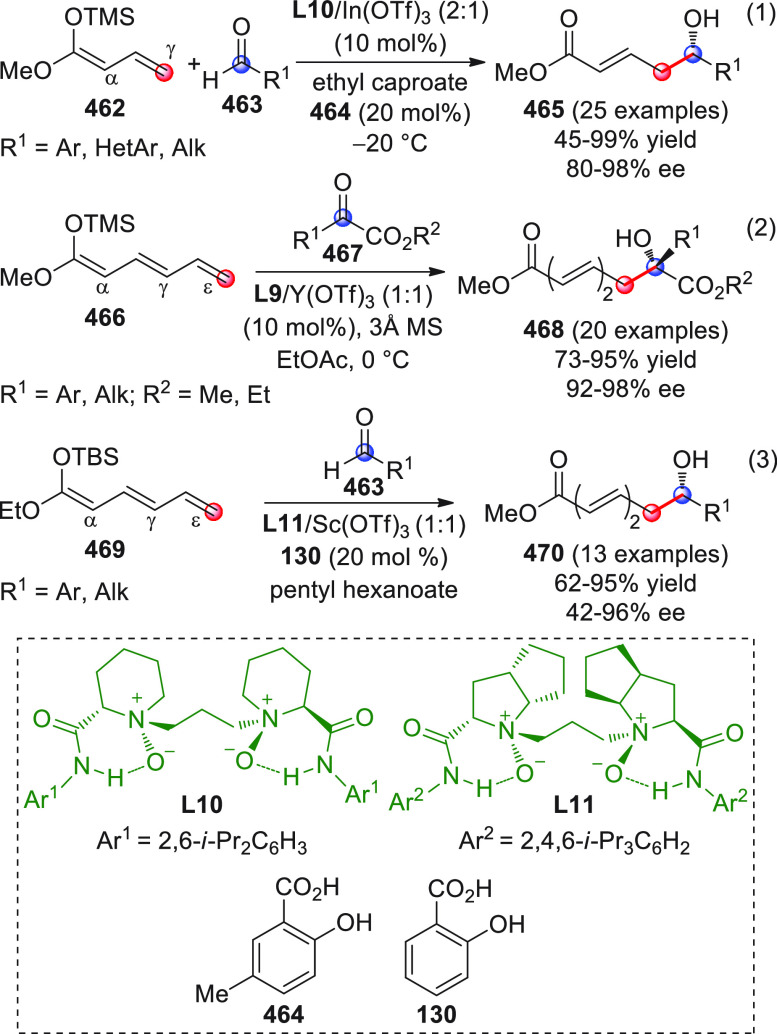


The development of catalytic, enantioselective VMAR involving linear, simple ester-derived dienol ethers such as silylketene acetal **462** (Scheme 127) is an issue of high importance in organic chemistry as it provides an efficient assembly of δ-hydroxy-α,β-unsaturated carbonyl compounds of type **465** and related polyketide frameworks, which are attractive targets for biology-driven research. Furthermore, it generally proved to be challenging since the absence of substituents in the α or β position may result in a lower α vs γ regioselectivity, or more pronounced product isomerization.

In this context, Feng’s group reported a catalytic, enantioselective VMAR promoted by a chiral *N,N’*-dioxide–indium salt complex between linear methyl crotonate-derived silyl ketene acetals **462** and differently functionalized aldehydes **463** (Scheme 127, eq 1).^322^ In particular, after an intense preliminary work, L-pipecolic acid-derived ligand **L10** complexed with In(OTf)_3_ in a 2:1 ratio was elected as the best catalyst system for guiding the reaction between **462** and **463** in ethyl caproate as solvent at −20 °C, in the presence of 5-methyl salicylic acid **464** (20 mol %), to yield the corresponding γ-adducts **465** in high yield (up to 99% yield) and enantioselectivity (up to 98% ee). Irrespective of the nature and position of the substituents, both aromatic and α,β-unsaturated aldehydes proved to be viable substrates, together with linear and branched aliphatic aldehydes (albeit with less efficiency and enantioselectivity).

More recently, Feng et al. reported the application of chiral *N,N′*-dioxide/Y(OTf)_3_ and Sc(OTf)_3_ complexes as efficient catalysts for the bisvinylogous Mukaiyama aldol reaction of linear silyl ketene acetals **466** and **469** with α-ketoesters **467** and aromatic or aliphatic aldehydes **463**, respectively (Scheme 127, eqs 2 and 3).^323^ It was found that l-proline derived ligand **L9** (Scheme 122) in combination with Y(OTf)_3_ (1:1 molar ratio), efficiently catalyzed the enantioselective and ε-selective bisvinylogous aldol addition of trienolsilyl ketene acetal **466** to a vast panel of aromatic and aliphatic α-ketoesters of type **467**. The corresponding polyunsaturated esters **468** were obtained in up to 95% yield and up to 98% ee, with very high >19:1 ε/α regiocontrol in most cases. Similarly, 10 mol % of L-ramipril derived ligand **L11** in combination with Sc(OTf)_3_ was the best choice for the aldol addition of silyl trienolate **469** to aldehydes **463** (Scheme 127, eq 3). The reaction carried out in pentyl hexanoate as the solvent at −30 °C with the addition of 20 mol % of salicylic acid **130** provided the corresponding *S*-configured aldol adducts **470** in good overall yields (up to 95%) and stereoselectivity (up to 96% ee). The reason for the observed different specificity of metals toward aldehydes and α-ketoesters was postulated to be related to the fact that the bigger Y(III) atomic radius would better coordinate the bidentate α-ketoesters, while monodentate aldehydes would better coordinate with smaller Sc(III). A transition state, resembling the one proposed for the VMAR of cyclic butenolides to isatins (Scheme 122) was also proposed accounting for the selective *Si* face attack to the aldehyde (not shown).

**Scheme 128:**
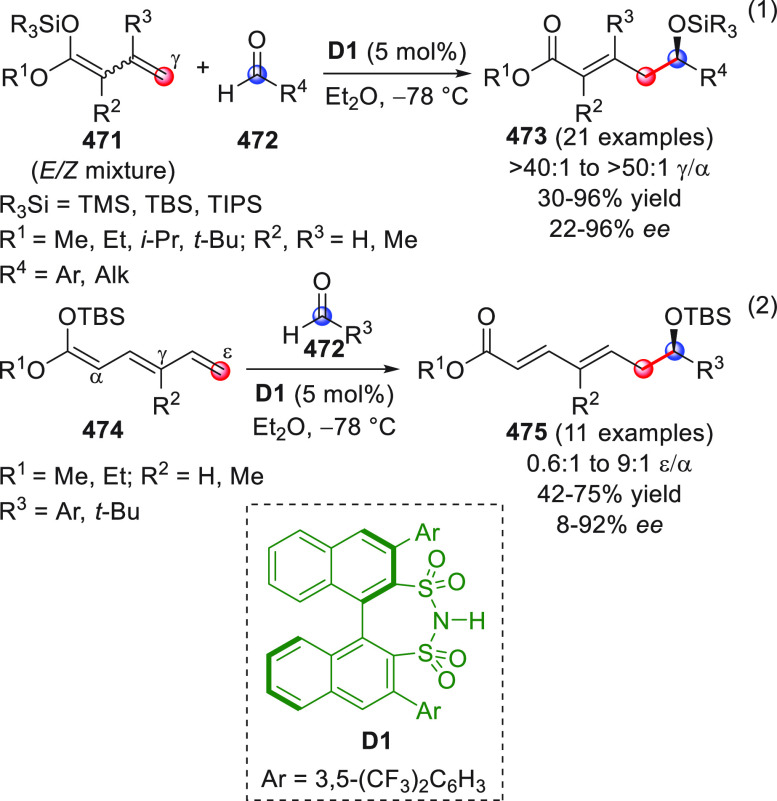


In 2011 List and co-workers demonstrated the ability of axially chiral disulfonimide DSI (**D1**) to efficiently catalyze the vinylogous and bisvinylogous Mukaiyama aldol addition of linear silyl ketene acetals **471** and extended silyl congeners **474** to a wide set of aldehydes, via an asymmetric counterion-directed catalysis mechanism (ACDC, Scheme 128).^324^ Irrespective of the dienolate geometry, *Z/E* mixtures of silyl ketene acetal **471** (eq 1) reacted smoothly with aromatic and cinnamaldehyde derivatives **472** in the presence of disulfonimide **D1** (5 mol %) in Et_2_O al −78 °C (for at least 3 days) giving almost exclusively the silylated γ-adducts **473** in good yields (up to 96%) with acceptable to high levels of stereocontrol (up to 96% ee). It was also found that aliphatic aldehydes were suitable substrates with promising reactivity, although reduced stereoselectivities were observed.

**Figure 5:**
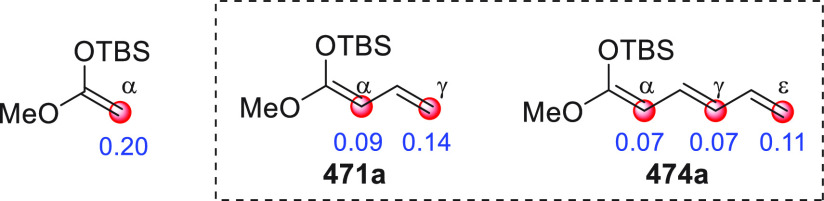
Calculated electron density values (Fukui’s numbers) of vinylogous nucleophiles as reported by List et al.

Comparable results were observed by applying a similar methodology between extended silyl trienolate derivatives **474** and aldehydes **472** (Scheme 128, eq 2). In this case, a catalytic, enantioselective, bisvinylogous Mukaiyama aldol reaction provided chiral ε-adducts **475** in acceptable yields (up to 75%) and enantiocontrol (up to 92% ee), but with a sensible drop of regioselectivity (up to 9:1 ε/α). This drop in regioselectivity was also predicted by density functional theory calculations (DFT) as reported in Figure 5: here, the reported Fukui’s values for the ε and α positions within extended trienolate **474a** vary less than for the γ and α values of the corresponding vinylogous nucleophile **471a**, envisaging a less distinct nucleophilic selectivity between the α and ε position.

**Scheme 129:**
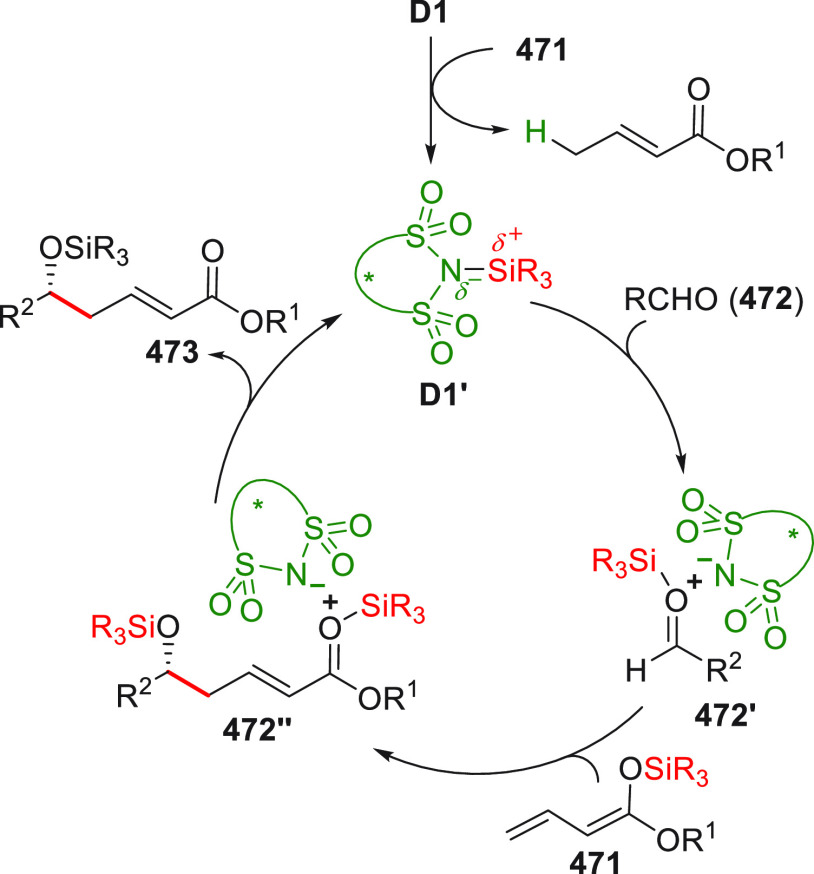


A 4-step ACDC pathway was proposed, in which chiral disulfinimide **D1** actually acted as a precatalyst. According to this catalytic cycle (Scheme 129), **D1** is initially activated by protodesilylation with the silyl ketene acetal involved in the reaction to form *N*-silyl disulfonimide complex **D1′**, that activates aldehydes **472** by *O*-silylation forming a chiral oxonium ion species **472′** coupled with the aza-anion of the chiral disulfonimide. At this point, a second molecule of silyl ketene acetal adds to chiral complex **472′** stereoselectively, to give silylated adducts **472′′** that finally release the products **473** while regenerating the silylated catalyst **D1′**.

**Scheme 130:**
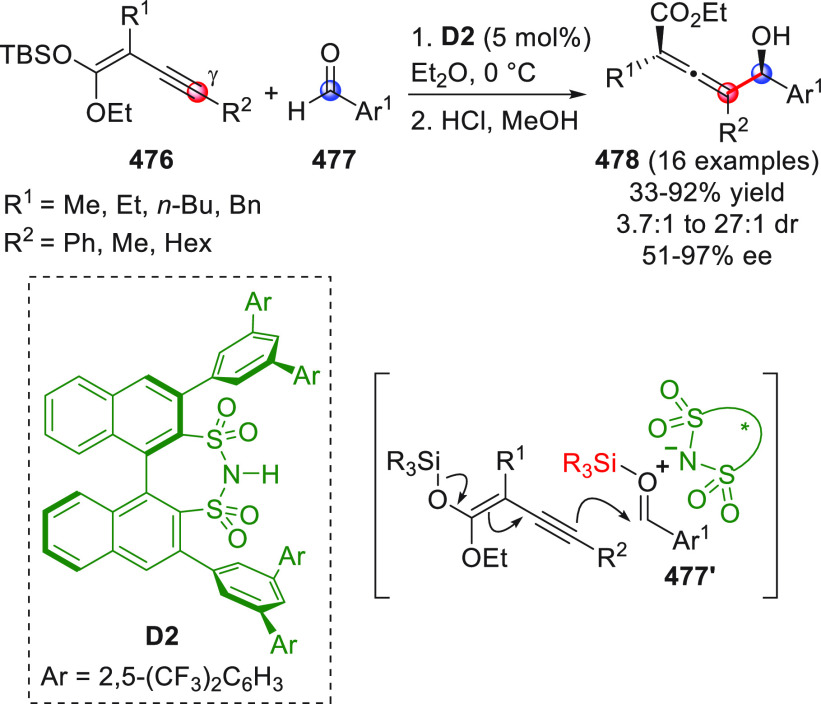


An interesting advancement of the ACDC vinylogous process highlighted above was recently reported by List’s group unveiling a novel, catalytic, enantioselective VMAR between silyl alkynyl ketene acetals of type **476** and aryl aldehydes **477** (Scheme 130).^325^ For the purpose, a newly designed chiral disulfonimide **D2** was elected as the best catalyst to deliver chiral tetrasubstituted allenoates of type **478** in high yields (up to 92%), with excellent regio- (>20:1 γ/α), diastereo-, and enantioselectivities (up to 27:1 dr, and up to 97% ee). This conceptually new “*alkynylogous*” transformation was challenging as it implied the selective γ-addition of an aldehyde acceptor through a nucleophilic enolate functionality transfer along a conjugated double and triple bond, generating a δ-hydroxy allenoate-moiety in a stereoselective manner. The developed reaction proved to be feasible to a broad range of aldehydes in combination with diverse alkynyl-substituted ketene acetals, and the obtained allenoate products were suitable substrates for a variety of further derivatizations accessing highly substituted enantiomerically enriched building blocks (not shown).

As the above methodologies demonstrate, the asymmetric VMAR is a powerful carbon–carbon bond-forming transformation with broad applicability that allows the construction of polyfunctionalized carbon chains in a convergent and stereoselective modality. Consequently, several syntheses were put forward in which the vinylogous Mukaiyama aldol reaction was successfully applied.

**Scheme 131:**
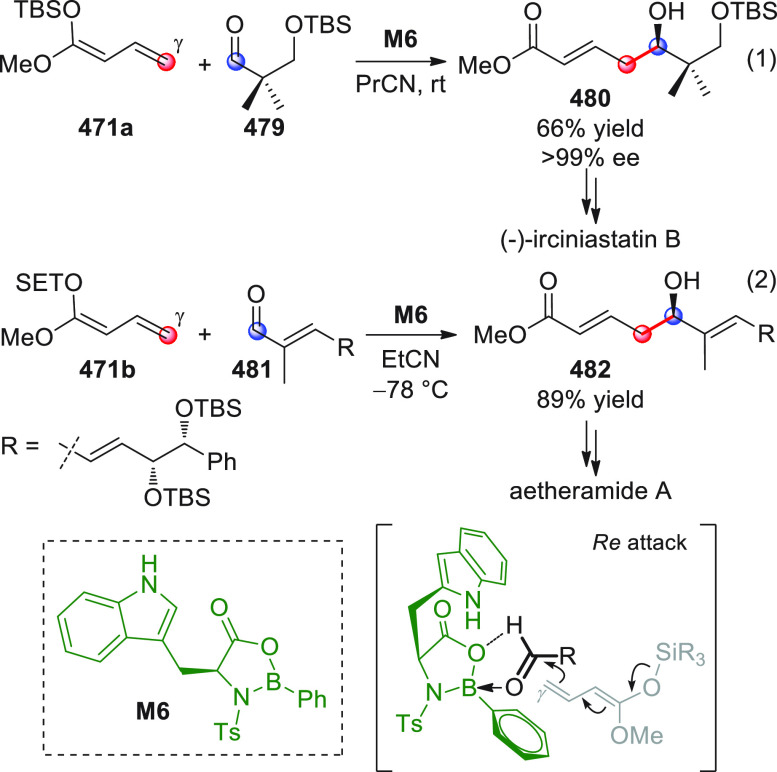


Polyketides, for example, proved to be attractive targets for biology-driven research.^34,326^ The established strategies for the total synthesis of polyketides often mimic the biosynthetic pathway by adding one propionate or acetate building block at a time. In this context, to reduce the number of transformations during a given synthesis, much effort has focused on the exploitation of vinylogous aldol or aldol-like processes which enable the stereoselective access to δ-oxy γ-homologated α,β-unsaturated ester derivatives with reliable predictability. In this context, chiral oxazaborolidinones (OXB)-promoted VMAR of linear silyl dienolates represent a good example of how a well-established asymmetric methodology may be exploited to the total synthesis of polyketide frameworks. OXB were independently developed by Yamamoto and Helmchen in 1990,^327,328^ and since then they have been applied in various asymmetric transformations including the Mukaiyama aldol reaction. Their utility in promoting enantioselective VMARs was first reported by Kalesse only in 2007 (e.g., section 3.1.2.1).^85^ In the past decade though, the usefulness of this chiral OXB-catalyzed VMAR was further assessed and exploited in natural product synthesis as demonstrated by Smith’s group in their total synthesis of (+)-irciniastatin A) and (−)-irciniastatin B, two cytotoxic secondary metabolites (Scheme 131, eq 1).^329^

To access the key tetrahydropyran precursor, the enantioselective VMAR between aliphatic α,α-disubstituted aldehyde **479** and linear silyl ketene acetal **471a** was performed using L-tryptophan-derived B-phenyl-oxazaborolidinone **M6** as the chiral promoter. Using Kalesse’s procedure (1 equiv of **M6** in butyronitrile at rt), the corresponding vinylogous adduct **480** was obtained in good 66% yield and absolute enantiocontrol (>99% ee). More recently, in 2016 Kalesse used a similar reaction in the total synthesis of the highly potent anti-HIV natural product aetheramide A (Scheme 131, eq 2).^330^ These results could be rationalized by the originally proposed transition state for OXB-catalyzed VMARs (Scheme 131, bottom),^30^ according to which the observed enantioselectivity is based on the selective attack of the silyl nucleophile to the *Re* face of the aldehyde due to the shielding of the *Si* face operated by the indole moiety of the promoter.

**Scheme 132:**
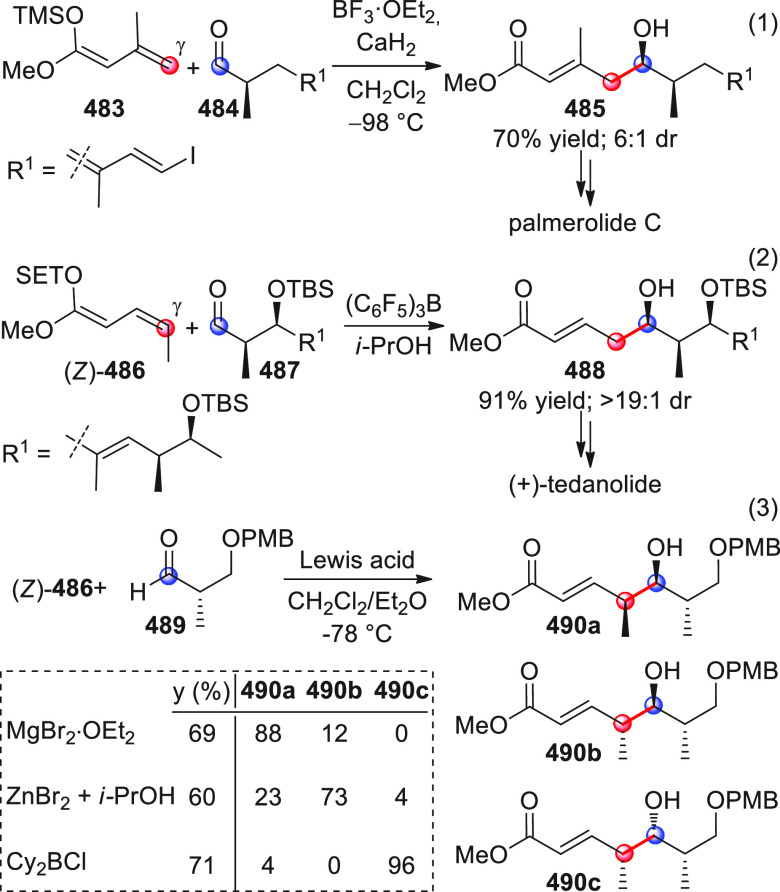


Among the plethora of stereoselective transformations involving linear silyl ketene acetals as nucleophilic components, substrate controlled, diastereoselective VMARs represent one on the most exploited transformations for the construction of chiral polyketide frameworks since simple diastereoselectivities can be easily obtained through either Felkin or *anti*-Felkin controlled additions to α-chiral aldehydes. In this context, boron-centered Lewis acids proved to be superior catalysts in promoting diastereoselective VMARs, as in the case of Florence’s synthesis of the polyketide-derived macrolide Palmerolide C (Scheme 132, eq 1).^331^ Here, the C15–C24 subunit of the target was accessed through a *syn*-selective VMAR between 3-methyl silylbutenoate **483** and chiral aldehyde **484**. Using BF_3_·OEt_2_ as a Lewis acid, the corresponding Felkin adduct **485** was obtained in a good 70% yield and 6:1 dr.

An additional level of complexity is added when nucleophiles exhibiting a terminal methyl group are employed. Such examples have been described by Kalesse et al. during the synthesis of the marine natural product (+)-tedanolide, a cytotoxic 18-membered marine polyketide isolated in 1984 (Scheme 132, eq 2). In 2012, Kalesse proposed an improved synthesis of tedanolide using a key diastereoselective VMAR between silyl ketene acetal (*Z*)-**486** and chiral aldehyde **487**, promoted by tris(pentafuorophenyl)borane B(C_6_F_5_)_3_ as the Lewis acid to afford the sole Felkin adduct **488** featuring an all-*syn* stereotriad, in 91% yield (>19:1 dr).^332^

The inherent directing effects of α-chiral aldehydes in Lewis acid-promoted diastereoselective VMARs were later investigated by Kalesse using silyl nucleophile (*Z*)-**486** and Roche aldehyde **489** as model compounds (Schema 132, eq 3).^333^ Depending on the nature of the Lewis acid employed, three different stereotriads could be obtained, namely, the chelation-controlled (*anti,syn*)-**490a**, the (*anti,anti*)-**490b**, or the (*syn,syn*)-**490c** (Scheme 132, bottom). The highest selectivities were observed for the Felkin all-*syn* product **490c** when Cy_2_BCl was used as the Lewis acid, proving again the superior ability of boron-centered Lewis acids to orchestrate the steroselective approach of the reagents.

**Scheme 133:**
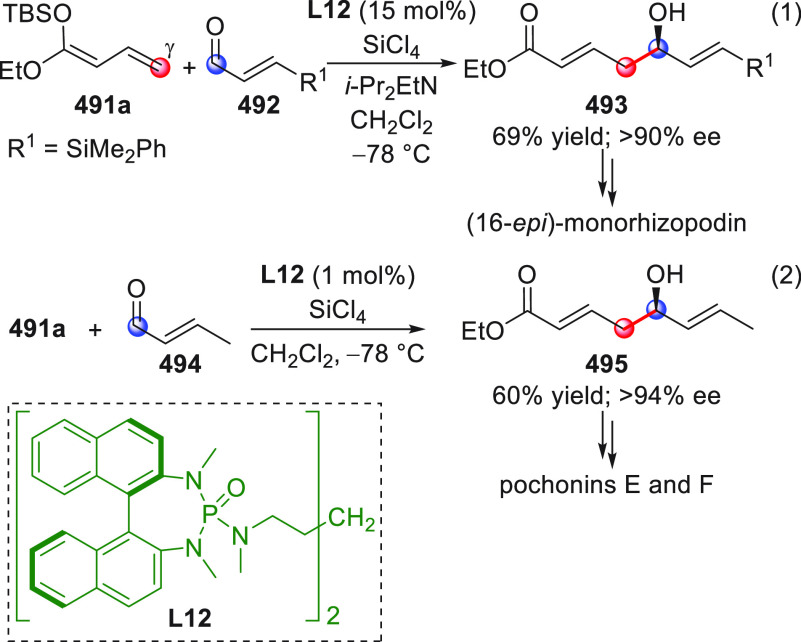


In the field of catalytic, enantioselective Mukaiyama-type transformations, the concept of *Lewis base activation of Lewis acids* developed by Denmark in 1990 has been fruitfully applied to vinylogous transformations, and it is now one of the most reliable systems to promote enantioselective VMARs.^334,335^ Denmark’s catalytic system relies on the addition of a chiral Lewis base such as bisphosphoramide **L12** (Scheme 133) to SiCl_4_ thus extending the silicon coordination sphere and generating a chiral catalytic complex with increased Lewis acidity that leads to successful activation of aldehydes while promoting for example enantioselective VMAR processes. Not surprisingly, it has found important applications in total synthesis, and few interesting examples involving the use of extended silyl ketene acetals as nucleophilic donors appeared recently.

In 2011, Nicolaou and co-workers applied Denmark’s **L12**·SiCl_4_ catalyst system during a synthetic and biological study on natural product monorhizopodin and its16-*epi*-analogue (Scheme 133, eq 1),^336^ which showed actin-binding properties without exhibiting cytotoxicity. Indeed, α,β-unsaturated aldehyde **492** treated with silyl ketene acetal **491a** in the presence of 15 mol % of bisphosphoramide **L12** and SiCl_4_ (1.1 equiv) afforded the corresponding optically pure secondary alcohol **493** in a good 69% yield and >90% ee.

Furthermore, in 2012 Winssinger and co-workers investigated a similar VMAR between dienolsilane **491a** and the challenging crotonaldehyde substrate **494** during the syntheses of HSP90 inhibitors pochonins E and F (Scheme 133, eq 2).^337^ Again, chiral bisphosphoramide **L12**·SiCl_4_ complex promoted the enantioselective formation of the advanced intermediate **495** in 60% yield and excellent enantioselectivity (>94% ee).

**Figure 6:**
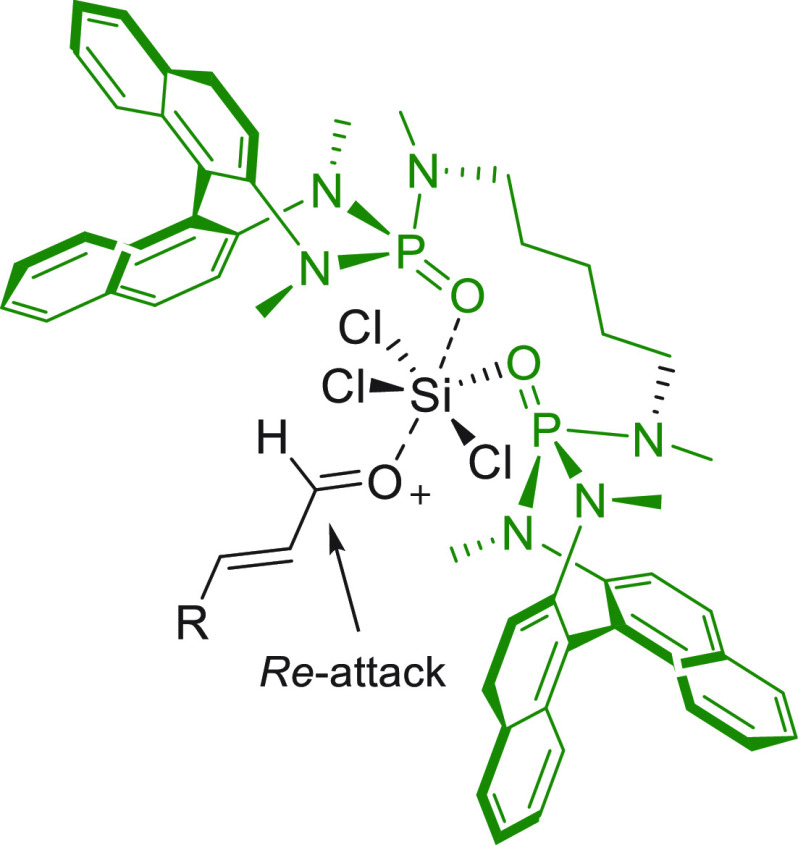
Catalytic complex involving Denmark’s SiCl_4_-bisphosphoramide **L12** and an enal.

Several studies by the Denmark group accounted for a transition state model in which the aldehyde binds to the hypervalent silicon center *trans* to one of the phosphoramides (Figure 6). This conformation places the aldehyde against one of the binaphthyl units and consequently allows exposure of the aldehyde’s *Re* face toward nucleophilic attack. Additionally, potential edge-to-face interactions between the aromatic aldehydes and the aromatic rings of the ligands can be used to rationalize the higher selectivity observed for unsaturated aldehydes as compared to simple aliphatic aldehydes.

##### 5.1.2.2 Cyclic Nucleophiles

**Scheme 134:**
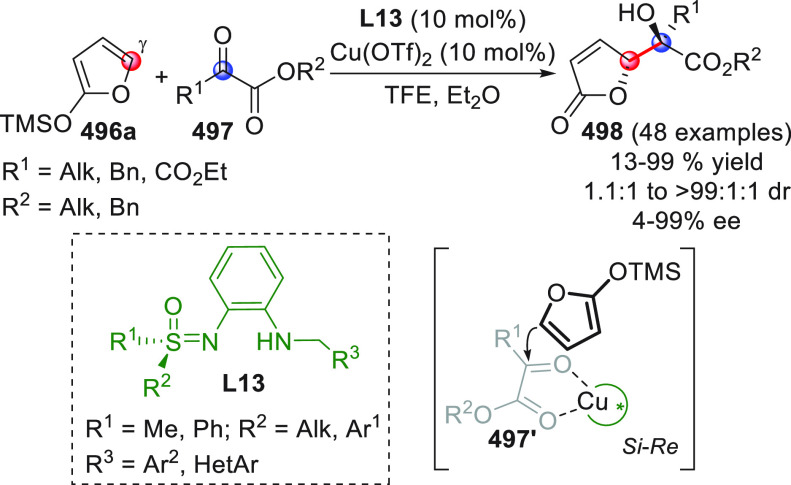


In 2010, the Bolm group reported the development of a previously introduced copper-catalyzed enantioselective vinylogous Mukaiyama-type aldol reaction between 2-(trimethylsilyloxy)furan (TMSOF, **496a**) and bidentate α-keto esters **497** affording the corresponding δ-hydroxy butenolides **498**, bearing a quaternary stereogenic center bridging the lactone core to the carboxylate unit (Scheme 134).^338,339^ After a fine-tuning of the reaction conditions and an optimization of the modularly assembled ligand structure, several *C*_1_-symmetric aminosulfoximine **L13/**Cu(OTf)_2_ couples were tested, providing the desired products with high stereoselectivities (up to >99:1 dr and up to 99% ee) and excellent yields (up to 99%). Furthermore, this catalytic, enantioselective VMAR tolerated various electrophile/nucleophile combinations, spanning from *S*-, *N*-, and *C*-analogues of TMSOF to various alkyl and benzyl substituted keto esters **497**, affording the corresponding thiobutenolide, lactam, and functionalized carbocycles in comparable yields and stereoselectivities (not shown).

The absolute configuration of the products was assigned as (5*R*,1′*R*), and the observed enantioselectivity was ascribed to a preferential attack of the *Si* face of TMSOF **496a** to the *Re* face of activated electrophile **497′** accounting for a 5-membered cyclic bidentate complex in which copper coordinates the two carbonyl oxygen atoms of the keto ester.

**Scheme 135:**
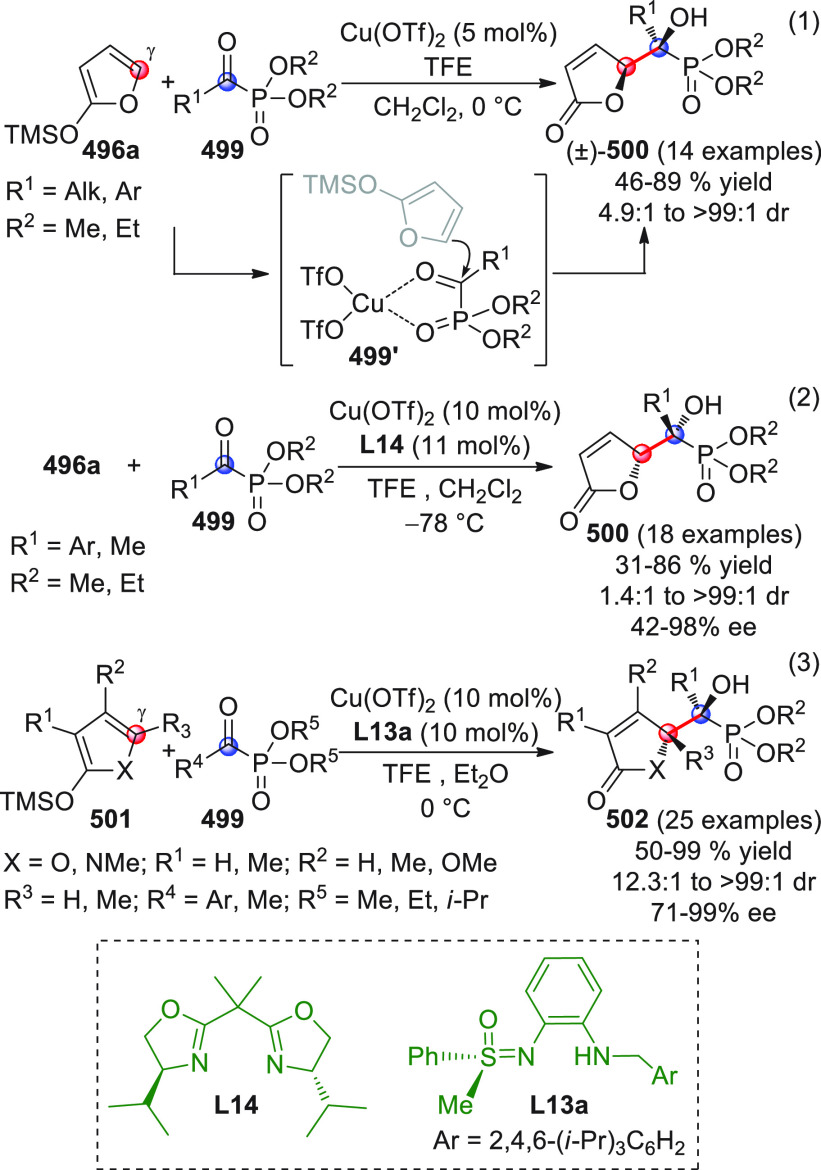


Similarly to the previously accessed chiral esters, chiral α-functionalized phosphonic acid derivatives have attracted particular attention in the past decade, due to their valuable pharmaceutical applications as anticancer and antivirus agents.^340^ The pharmaceutical potential of these compounds has stimulated the development of several methodologies for the preparation of δ-hydroxy alkylbutenolide phosphonate analogues, accessible via stereoselctive VMARs using mainly TMSOF **496a** as the lactone nucleophilic source. In this context, in 2011, Miao, Chen, et al. were the first to report a diastereoselctive VMAR between bidentate α-keto phosphonate **499** and 2-(trimethylsilyloxy)furan **496a** catalyzed by Cu(OTf)_2_ (5 mol %) in the presence of 2,2,2-trifluoroethanol (TFE, 1.2 equiv) as additive in CH_2_Cl_2_ at 0 °C (Scheme 135, eq 1).^341^ The reaction proceeded rapidly affording the corresponding (*O,P*)-*anti*-configured 5-(hydroxyarylmethyl) furan-2(5*H*)-one phosphonates (±)-**500** in high yields (up to 89%), with good to excellent diastereoselectivities (up to >99:1 dr). Similarly to the previously described work, the reaction was proposed to proceed via a staggered acyclic transition state in which Cu(OTf)_2_ coordinates the two oxygen atoms of the α-keto phosphonate (e.g., complex **499′**), creating suitable steric effects that dictate the diastereoselective approach of the reactants.

Later in 2013, the same group devised an asymmetric version of such transformation exploiting a *C*_*2*_-symmetric bis(oxazoline) **L14**·Cu(OTf)_2_ catalyst complex (Scheme 135, eq 2).^342^ Indeed, the asymmetric VMAR between α-keto phosphonates **499** and TMSOF **496a** carried out in dichloromethane at −78 °C in the presence of TFE as additive afforded the corresponding tertiary α-hydroxy phosphonates **500** in moderate to high yields (up to 86%), as well as high enantio- (up to 98% ee) and diastereoselectivities (up to 99:1) in favor of the (5*R*,1′*R*) configured isomers as assessed by X-ray crystallographic determination.

Almost in the same period, a very similar catalytic and highly stereoselective copper-catalyzed VMAR between α-keto phosphonates **499** and functionalized heterocyclic dienol silyl ketene acetals **501** was developed by Bolm and co-workers, exploiting the just mentioned *C*_*1*_-symmetric **L13a·**Cu(OTf)_2_ chiral catalytic complex (Scheme 135, eq 3).^343^ Here, (5*R*,1′*S*)-configured phosphonic γ-(hydroxyalkyl)butenolides **502** (and one *N*-Me lactam congener, not shown) were accessed with high yields and stereoselectivities showing a broad functional group tolerance for both the electrophilic and nucleophilic reactants.

**Scheme 136:**
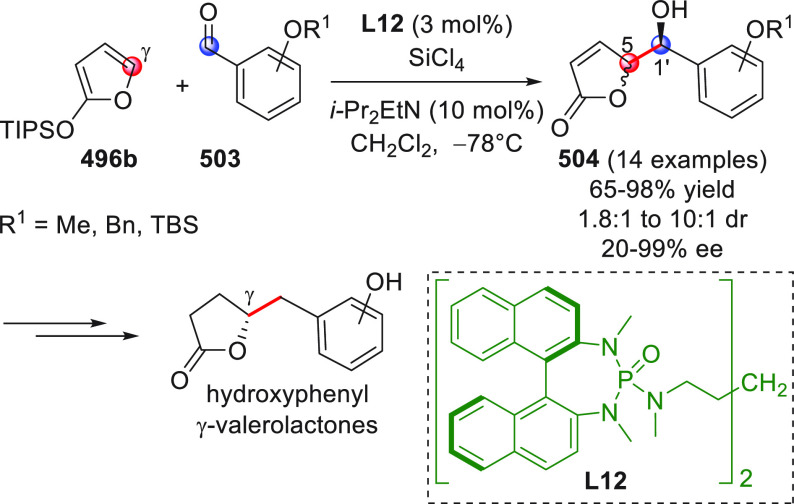


The ability of siloxyfuran nucleophiles to act as d_4_ donor reagents in vinylogous, enantioselective aldol, and related processes was demonstrated by Casiraghi’s group, who investigated the VMAR of pyrrole- and furan-based dienoxysilanes in a catalytic, asymmetric format. In this context, the previously disclosed Denmark bisphosphoramide **L12**/SiCl_4_ couple proved to be a privileged catalytic system in promoting the enantioselective VMAR between a suitable silyloxyfuran derivativative and a set of aromatic aldehydes (not shown).^344,345^ Several years later, Curti, Del Rio, et al. applied Casiraghi’s methodology to the total synthesis of hydroxyphenyl γ-valerolactones, an important class of flavan-3-ol colonic metabolites (Scheme 136).^346,347^ Starting from 2-triisopropylsilyloxyfuran **496b** and diverse alkoxy-substituted benzaldehydes **503**, Denmark’s catalytic system **L12**·SiCl_4_ enabled the formation of the corresponding δ-hydroxybutenolides **504** in high yields (up to 98%) and generally good to high enantioselectivities. Interestingly, the diastereoselectivity of the process was highly dependent upon the nature and the substitution pattern of benzaldehydes **503**; in fact, while 3′- and 4′-monosubstituted aldehydes yielded the expected *anti*-adducts **504**, 3′,4′-bisilyloxy and 3′,4′,5′-trisilyloxybenzaldehyde congeners mainly furnished the C5-epimeric *syn*-configured adducts. Unexpectedly, while the majority of butenolide products **504** possessed the expected (1′*S*) absolute configuration (resulting from an attack on the expected *Re* face of the aldehyde), a striking and unprecedented enantiofacial inversion was experienced with polymethoxy- and polybenzyloxy-substituted aldehydes, for which (1′*R*)-configured *anti*-adduct were obtained (*Si* face attack). Selected δ-hydroxybutenolides **504** were then chosen as key precursors for the chemodivergent synthesis of chiral, enantioenriched hydroxyphenyl γ-valerolactone metabolites, as well as their racemic δ-valerolactone congeners (not shown).

**Scheme 137:**
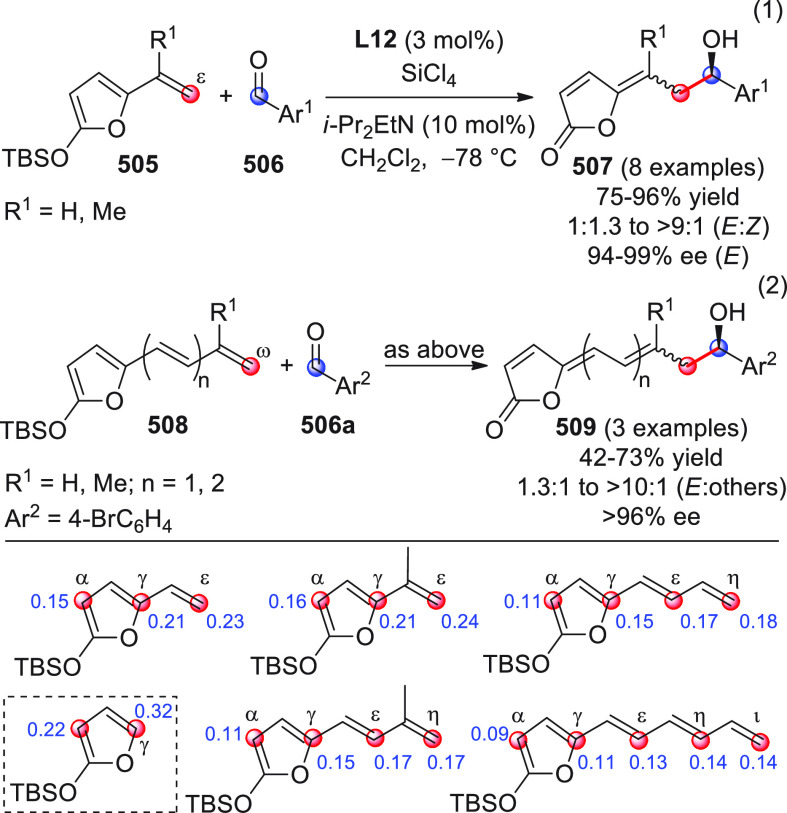


In their continuing efforts to study the reactivity of vinylogous nucleophiles such as silyloxyfuran derivatives, the Casiraghi’s group also explored more extended adaptations of the classical heterocyclic siloxydienes. Focusing on densely unsaturated butenolides, in 2011 they reported a reliable catalytic, asymmetric bisvinylogous and hypervinylogous Mukaiyama-type aldol methodology using easily available extended furan-based silyloxy polyenes of type **505** and **508** (Scheme 137).^348^ Initially, the asymmetric bisvinylogous VMAR using trienolsilane **505** and a set of aromatic aldehydes **506**, carried out in the presence of the Denmark’s bisphosphoramide **L12**·SiCl_4_ catalyst system and DIPEA (10 mol %) in CH_2_Cl_2_ at −78 °C, gave bisvinylogous 3′-hydroxybutenolides **507** with extraordinary ε-selectivity, in high isolated yields (up to 96%), and generally good to very high enantioselectivites (up to 99% ee) in favor of the (*E*,3′*R*)-configured isomer. This procedure was further applied to the more demanding VMAR between silyl polyenolates **508** and 4-bromobenzaldehyde **506a** (Scheme 137, eq 2), demonstrating a perfect relay of the enolate reactivity over up to five conjugated double bonds and named this phenomenon “*hypervinylogy*”. This novel hypervinylogous Mukaiyama aldol reactions (HVMARs) displayed complete regioselectivity at the most remote ω-nucleophilic site of the substrates (ω vs α/γ/ε), good to moderate control of the product geometries (*E* vs *Z*), and excellent enantiocontrol (>96% ee). These findings contrasted with the previously reported results by List et al.^324^ (Scheme 128), where a sensible drop of regioselectivity was observed for the DSI-catalyzed bisvinylogoua VMAR of linear silyltrienolates. To account for the observed regioselectivity, DFT and Fukui functions calculations were performed on silyl (poly)enolate nucleophiles **505** and **508** (Scheme 137, bottom). Atomic Fukui indices at the carbon atoms of the reacting polyenes were in line with the data obtained by List, foreseeing a preferential electrophilic attack of the aldehydes at the terminal carbon atom of the silyloxy nucleophiles.

Switching the attention to the role of the medium (or solvent) in promoting vinylogous transformations, the past decade has witnessed a renewed interest toward alternative and potentially “*greener*” organic chemistry methodologies based on the use of safer and environmentally friendly reagents, catalysts, and reaction media. Water represents an interesting alternative to the more classical organic solvents due to its unique structure-dependent behavior that enhances hydrophobic interactions within transition states, even when the solubility of the reactants in water is low and the reaction takes place at the solid/liquid or liquid/liquid interfaces (in a pioneering work Sharpless introduced the expression “*on-water conditions*” to denote the rate acceleration observed in organic reactions when water-insoluble reactants are vigorously stirred in water suspension).^349^

**Scheme 138:**
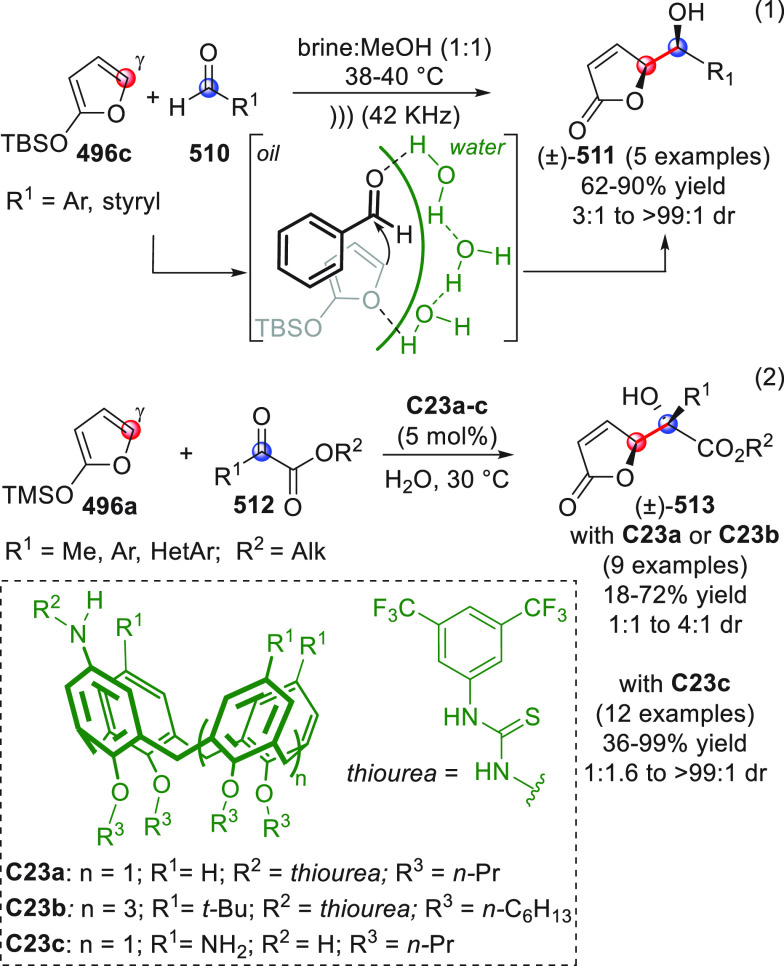


In this context, Curti et al. and, more recently, De Rosa, Palombi, and co-workers demonstrated the feasibility of a diastereoselective “on water” VMAR applied to furan-based siloxydienes **496c** and **496a** (Scheme 138). In particular, in 2010, Curti, Casiraghi, et al. reported the first, uncatalyzed VMAR carried out “on water” conditions, using a salty water/methanol mixture as reaction medium (Scheme 138, eq 1).^350^ Ultrasonic irradiation of an emulsion of TBSOF (**496c**) and aromatic and cinnamic aldehydes **510** in a 1:1 brine/methanol mixture at 38–40 °C allowed the formation of the corresponding δ-hydroxy butenolides (±)-**511** in high yields and good to excellent diastereoselectivity in favor of the 5,1′-*syn* configured adducts. Interestingly, the reaction between pyrrole-based dienoxysilane congeners and aromatic aldehydes proved also to be viable under the optimized condition, giving the corresponding vinylogous adduct in high yield but with an inverted *anti*-selectivity (see section 6). To account for the observed *syn*-selectivity, the authors speculated that, at the boundary between water and the dispersed droplets of the lipophilic reactants, water molecules worked as H-bond donor species that activate the aldehyde acceptor while controlling the mutual position of the reactants in a stacking synclinal transition state, ultimately leading to the favored *syn*-isomers (±)-**511**.^351^

Later, as part of a long-lasting venture toward unusual, environmentally friendly organocatalytic systems to promote stereoselective vinylogous transformations under solvent-free or aqueous media,^352−354^ De Rosa, Neri, and co-workers designed calixarene-based catalysts **C23a–c** (Scheme 138, bottom) to promote diastereoselective VMARs between TMSOF **496a** and α-keto esters **512** under “on water” conditions. In a first report in 2016,^355^ they found that thioureido-calix[4]arene **C23a** or its calix[6]arene congener **C23b** (5 mol %) enabled the VMAR between **496a** and a series of alkylbenzoylformates to yield the corresponding γ-adducts (±)-**513** in acceptable yields with a slight preference for the 5,1′-*anti*-configured isomers (Scheme 138, eq 2). Interestingly, the authors found that lower conversions and a switch in diastereoselectivity in favor of *syn*-adducts could be observed when the same reaction was carried out in organic solvents under homogeneous catalysis. These observations and further NMR and computational studies unveiled the basis of the supramolecular control exerted by this type of catalysts, according to which the rate acceleration of the VMAR is closely related to the hydrophobicity of the calixarene skeleton and its ability to recognize the α-keto ester via H-bonding interactions with the thiourea group. The hydrophobic amplification of weak interactions between catalyst and substrates under “on-water” conditions inspired the De Rosa group to search for new supramolecular entities able to promote a distereoselective on-water VMAR. In 2017 these efforts ended up with the design of the simple tetraminocalix[4]arene **C23c** (Scheme 138),^356,357^ bearing weak H-bond-donor NH_2_ functionalities, capable to promote the VMAR of TMSOF **496a** with polyfunctionalized α-keto esters **512**, to afford δ-hydroxybutenolides (±)-**513** in good yields (up to 99%) and acceptable diastereoselectivities.

**Scheme 139:**
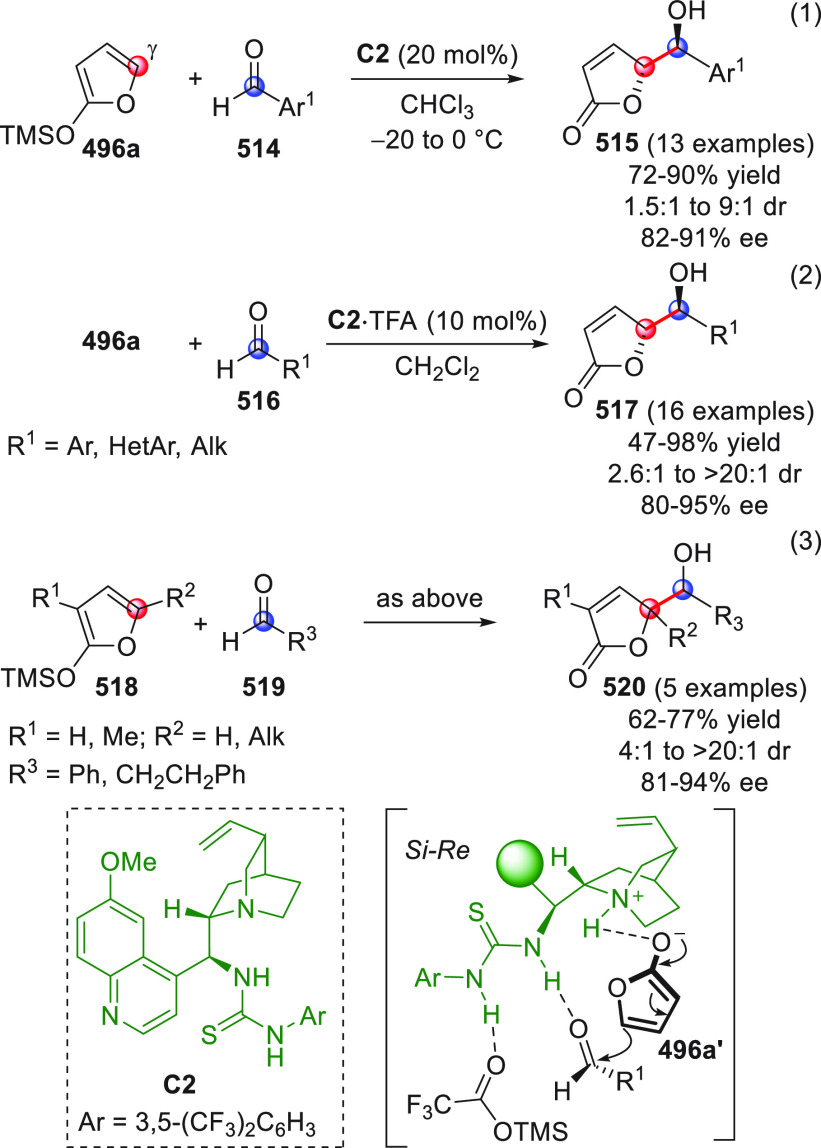


In the context of organocatalyzed asymmetric methodologies,^358^ in 2010 Ma and Wang developed the asymmetric, vinylogous addition of TMSOF **496a** to diverse aromatic aldehydes **514**, using the bifunctional 9-*epi*-quinine-thiourea **C2** as the catalyst of choice (Scheme 139, eq 1).^359^ The reaction, performed with **C2** (20 mol %) in CHCl_3_ at −20 to 0 °C afforded a panel of diverse chiral, 5,1′-*anti*-configured δ-hydroxybutenolides **515** in good yields (up to 90%), and stereoselectivity (up to 9:1 dr, and up to 91% ee).

An almost similar procedure was reported in the same year by the Deng group,^360^ who successfully exploited readily accessible organic catalysts based on a carboxylate ammonium salt such as **C2·**TFA (Scheme 139, eq 2) prepared by mixing **C2** with trifluoroacetic acid (TFA) in a 1:1 ratio. This newly developed chiral organic salt was able to effectively promote an *anti*-selective asymmetric VMAR between TMSOF **496a** and diverse aryl, alkenyl, and alkyl aldehydes of type **516**, affording the corresponding butenolides **517** with improved yields (up to 98%) and stereoselectivities (up to >20:1 dr, and up to 95% ee) even with the more challenging aliphatic aldehydes. Furthermore, catalyst **C2·**TFA was able to promote the VMAR of structurally hindered 5-substituted-2-trimethylsilyloxy furans **518** and aldehydes **519** generating chiral adducts **520** bearing adjacent tertiary-quaternary centers with comparable selectivities (not shown). To account for the ability of **C2·**TFA salt catalyst in promoting the reported *anti*-selective VMAR, a transition state was proposed based on the peculiar structure of the catalyst featuring a protonated quinuclidine unit and a carboxylate moiety linked to the thiourea through hydrogen-bond interactions (Scheme 139, bottom). The author postulated that the carboxylate ion could react with silyloxy furan **496a** to form the corresponding trimethylsilyl ester and the 2-furoxy anion **496a′** while releasing a thiourea-NH functionality that could serve as a hydrogen bond donor to activate the aldehyde acceptor. With the nucleophilic anion and aldehyde in place, the reaction between the two reactants might proceed generating the observed *anti*-adducts.

As for the previously described linear silyl ketene acetals, cyclic silyloxy dienes are invaluable tools with which a myriad of densely functionalized nonracemic molecules could be constructed. Furthermore, the vinylogous aldol-type addition of these nucleophiles to carbonyl compounds (VMAR) has become one of the main routes for the assembly of chiral, enantiopure carbon chains of polyketide and polyketide-like natural products. In this context, paralleling the above-described methodological efforts to develop new, metal- or organocatalyzed asymmetric VMARs, the past decade has witnessed the exploitation of such chemistry on chiral aldehyde substrates, for the synthesis of chiral, enantioenriched bioactive compounds.

**Scheme 140:**
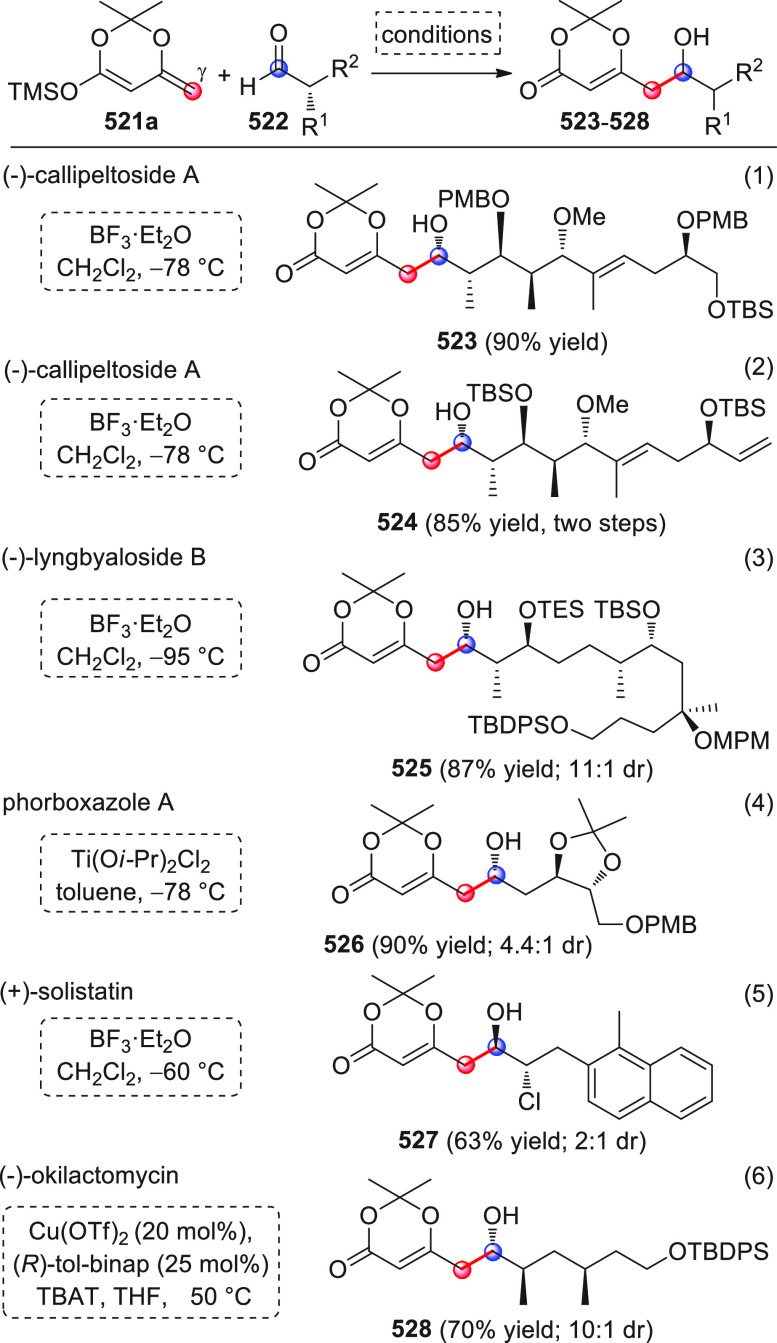


A diastereoselective BF_3_·OEt_2_-catalyzed VMAR between the dioxinone-derived dienolsilane **521a** and suitable polysusbstituted chiral aldehydes of type **522** was expoited by Hoye’s group in 2010 in the total synthesis of the cytotoxic macrolide (−)-callipeltoside A (Scheme 140, entry 1).^361^ Here, the corresponding vinylogous Felkin-adduct (5,6-*syn*)-**523** was obtained in a very good 90% yield as a single isomer. Similarly, Yadav et al. (2012)^362^ and Fuwa et al. (2015, 2016)^363,364^ applied the methodology with comparable results in the synthesis of either the C1–C14 macrolactone core of (−)-callipeltoside A (Scheme 140, entry 2), or the cytotoxic14-membered macrolide (−)-lyngbyaloside B (entry 3). A favorable 1,3-induced diastereoselective VMAR was studied by Forsyth’s group in the effort to synthesize the natural product phorboxazole A (entry 4).^365^ Indeed, during the preparation of the C31–C43 domain of the target, a highly efficient VMAR between **521a** and a 3,4-dioxygenated aldehyde acceptor was devised using a stoichiometric Ti(O*i*-Pr)_2_Cl_2_ as a Lewis acid, affording the corresponding adduct **526** in a good 90% yield and 4.4:1 dr..

An alternative diastereoselective VMAR was devised by Britton and co-workers in 2013 and applied to the total synthesis of the natural product (+)-solistatin (Scheme 140, entry 5).^366^ This strategy envisaged the use of a chiral aldehyde bearing a single chlorine atom introduced via enantioselective organocatalytic α-chlorination that acted as an easily removable achiral auxiliary. The Felkin stereodirecting influence of the chlorine atom resulted in the formation of the corresponding vinylogous adduct **527** with a good 63% yield as a 2:1 mixture of diastereoisomers in favor of the *anti*-configured isomer. Conversely, an *anti*-Felkin diastereoselective VMAR was applied by Scheidt in 2011 for the total synthesis of okilactomycin, an antitumor antibiotic agent.^367^ Using Kruger and Carreira’s copper-catalyzed VMAR^368^ between **521a** and a pseudoephedrine-derived chiral aldehyde acceptor, a key β-hydroxy dioxinone fragment **528** was assembled in 70% yield and 10:1 diastereomeric ratio (Scheme 140, entry 6).

Highly substituted dioxinone-derived silyl dienolates were also exploited in diastereoselective VMARs with chiral aldehydes demonstrating, once again, the high versatility exerted by this family of silyl nucleophiles frequently used for the stereoselective construction of complex chiral carbon chains. An important application of such versatility was assessed by Meyers in 2016 reporting a practical and convergent route to a very large set of macrolide antibiotics.^369^

**Scheme 141:**
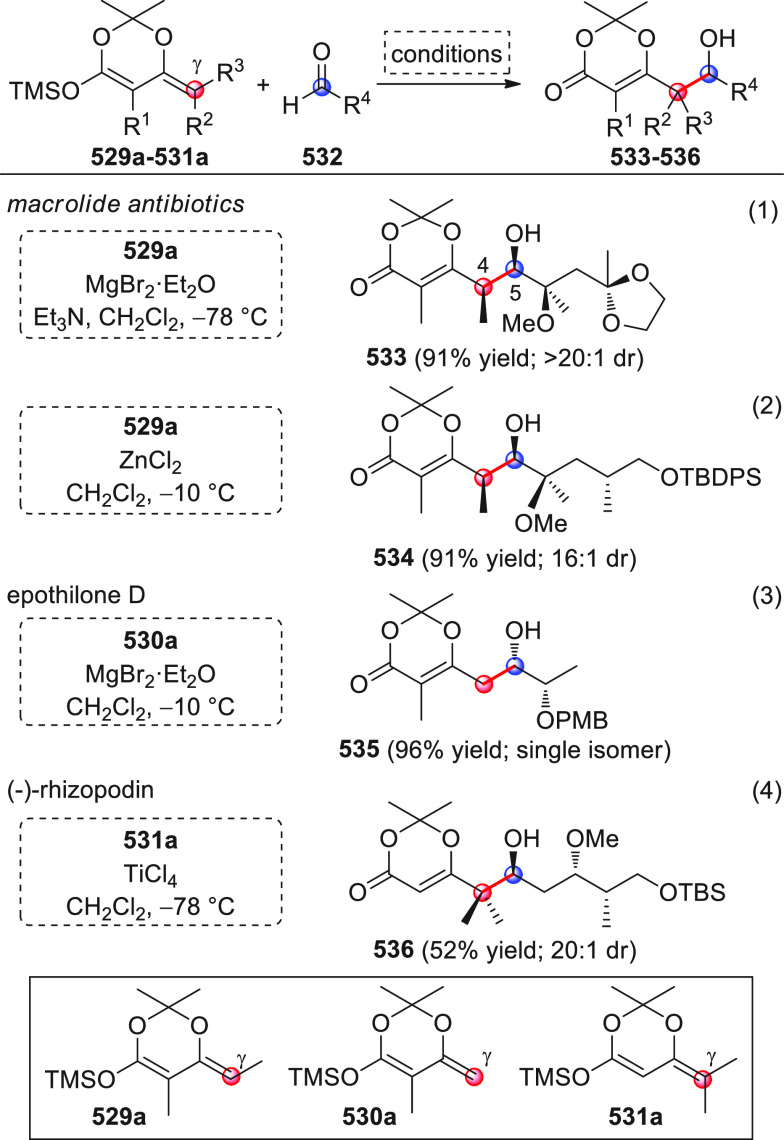


Here, two key diastereoselective VMARs between *Z*-configured dioxinone **529a** and α-quaternarized chiral aldehydes were reported, generating the 4,5-*syn* aldol adducts **533** and **534** under different reaction conditions (Scheme 141, entries 1 and 2). In fact, the use of chelating magnesium bromide etherate (entry 1) or ZnCl_2_ (entry 2) as Lewis acids both yielded the corresponding (4*S*,5*R*,6*R*)-configured stereotriad with optimal yields (91% for both) and comparable diastereoselectivities (16:1 vs >20:1). A highly *syn*-selective MgBr_2_-catalyzed diastereoselective VMAR of methyl-dioxinone **530a** was also exploited by Mulzer et al. in the total synthesis of epothilone D (entry 3).^370^ The reaction of **530a** with a suitable PMB-protected α-hydroxy aldehyde yielded the corresponding *anti*-Felkin adduct **535** as a single isomer in almost quantitative yield. Finally, a titanium-catalyzed VMAR of γ,γ-disubstituted silyl ketene acetal **531a** to a suitable 3-substituted chiral aldehyde was reported by Paterson et al. in 2013^371^ in the total synthesis of the macrocyclic polyketide (−)-rhizopodin (entry 4); a selective 1,3-chelate mechanism was here observed, allowing access to the corresponding 5,7-*anti*-configured adduct **536** with very high diastereocontrol (20:1 dr).

**Scheme 142:**
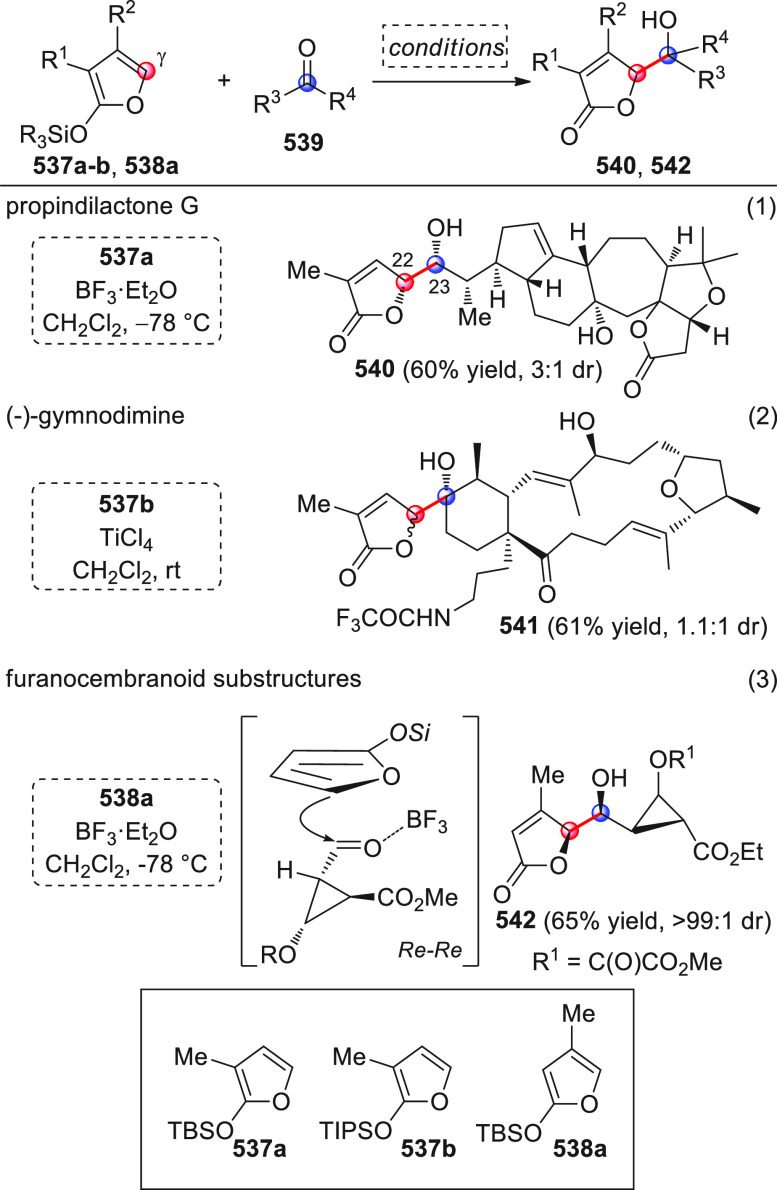


Turning to furan-based silyloxydienes, in 2016 Chen, Yang, et al.^372,373^ envisaged that the diastereoselective VMAR between 2-methyl-TBSOF **537a** and a 2-methyl chiral aldehyde could give access to the C22–C23 *syn*-δ-hydroxybutanolide framework of the bioactive nortriterpenoid propindilactone G (Scheme 142, entry 1). The reaction carried out in the presence of BF_3_·Et_2_O (1.2 equiv), afforded the corresponding γ-adduct **540** in 60% yield, as a 3:1 *syn*/*anti* diastereomeric mixture. Regrettably, the resulting Felkin-type stereochemistries of the newly generated C22 and C23 stereogenic centers were opposite to those found in the natural product, and thus an alternative strategy needs to be devised. In 2011 Romo^374^ and co-workers reported a convergent total synthesis of the marine toxin (−)-gymnodimine envisaging a late-stage VMAR between silyloxyfuran **537b** and a challenging chiral cycloexanone precursor to stereoselectively link the butenolide framework to the macrocyclic core of the target molecule. Brief exposure (ca. 1 min) of a mixture of **537b** and the ketone acceptor to TiCl_4_ led to a smooth addition reaction, to provide the corresponding δ-hydroxy lactone **541** as a 1.1:1 diastereomeric mixture in 61% yield (entry 2).^375^

A diastereoselective methodology for preparing the butenolide–butyrolactone and furan–butyrolactone units embedded in complex natural furanocembranoid diterpenes was developed by Reiser et al. in 2012 (Scheme 142, entry 3).^376^ After a brief optimization survey, a highly selective (>99:1 dr) boron-catalyzed VMAR of **538a** with an enantiopure cyclopropylcarbaldehyde yielded the corresponding *syn*-configured butenolide **542** structure in 65% yield in accordance with a proposed Felkin–Anh type model. This target served as key precursor for the synthesis of the northeastern sector of furanocembranoid bielschowskysin.^377^

**Scheme 143:**
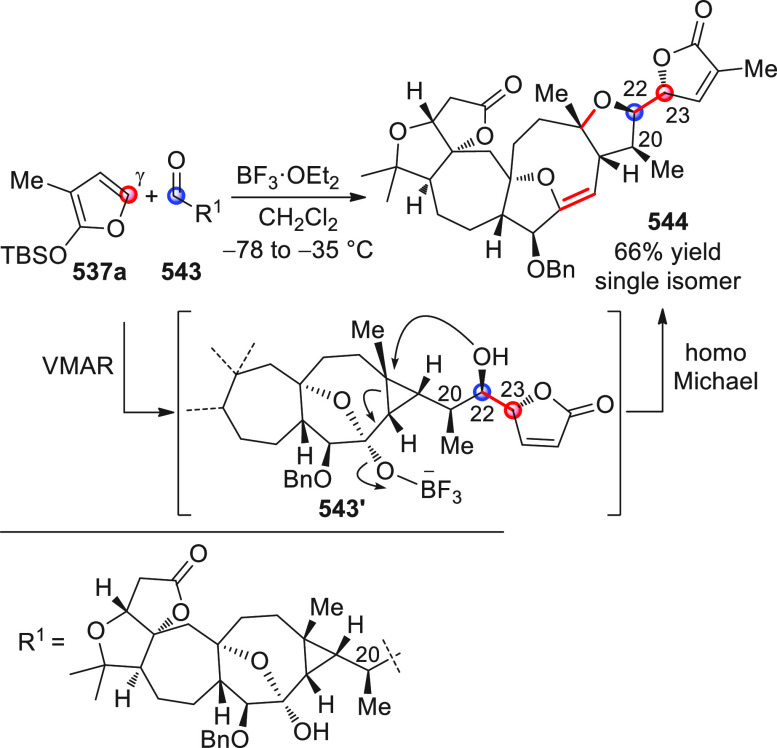


A remarkable application of a diastereoselective VMAR involving a siloxyfuran nucleophile was exploited by Han, Jiang, and co-workers in 2016 in their report on the asymmetric total synthesis of (+)-19-dehydroxyl arisandilactone A (Scheme 143).^378^ It was serendipitously found that the BF_3_·Et_2_O-catalyzed VMAR between **537a** and the strained polycyclic chiral aldehyde **543** afforded the arisandilactone A-precursor **544**, as a result of a one-pot tandem reaction in which a typical diastereoselective VMAR forming the Felkin intermediate **543′** was followed by an intramolecular oxa-Michael reaction that generated a new tetrahydrofuran ring by cleavage of the cyclopropane carbon–carbon bond. The newly generated stereocenters at C20, C22, and C23 within product **544** formed a *syn/syn* stereotriad that was later epimerized to the corresponding *anti/syn* platform found in the target, via a DBU-catalyzed isomerization of the butenolide moiety (not shown).

**Scheme 144:**
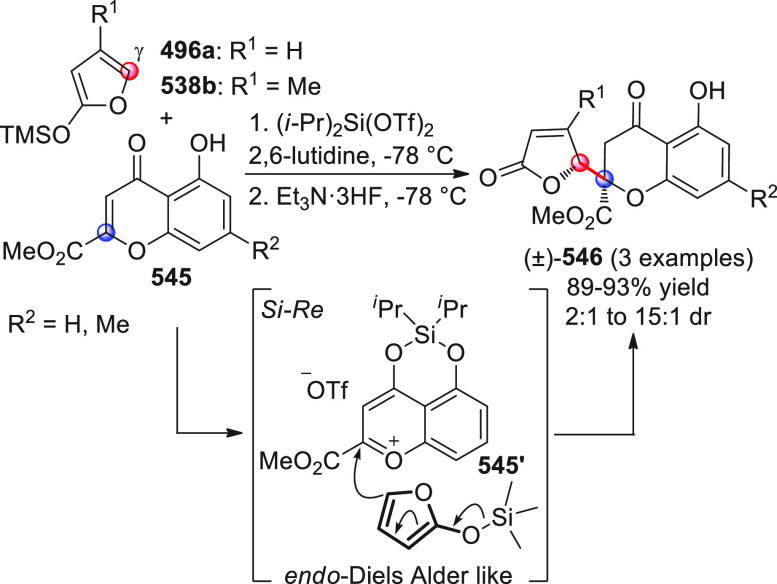


In 2011 Porco Jr. and co-workers reported the total synthesis of tetrahydroxanthone natural products blennolides B and blennolide C in racemic formats, centered on a VMAR-type addition of siloxyfurans to an in situ generated benzopyrylium ion (Scheme 144).^379^ After a concise optimization survey, treatment of hydroxy chromone **545** with diisopropylsilyl ditriflate in the presence of 2,6-lutidine led to the formation of a benzopyrylium intemediate **545’** that, treated with 2-trimethylsiloxyfurans **496a** and **538b** at −78 °C, cleanly led to formation of chromone butenolides of type (±)-**546** in good yields (up to 93%) and diastereoselectivity (up to 15:1 dr) after base-promoted desilylation. The regio- and diastereoselectivity of the vinylogous additions were probed using computational studies, which suggested the involvement of (Re*-Si*) Diels–Alder-like transition states.^380−383^

**Scheme 145:**
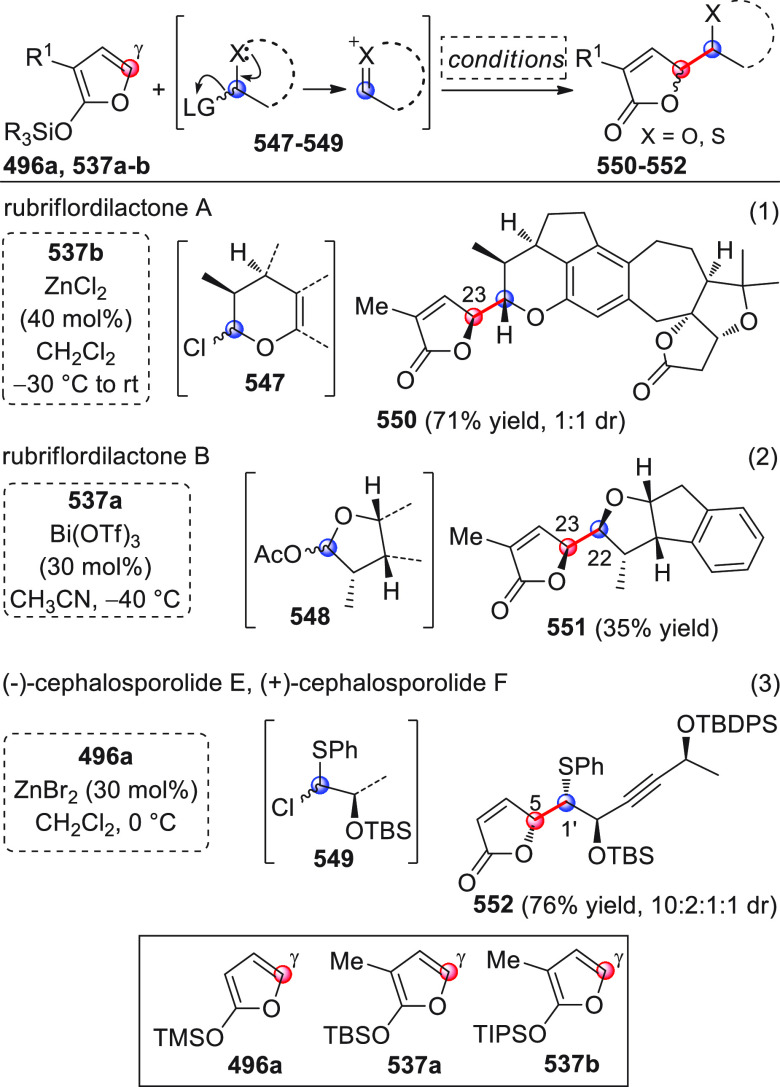


Another smart application of a VMAR-type addition of a siloxyfuran nucleophile to an in situ-formed oxocarbenium ion was introduced by Anderson and co-workers in the final step of their enantioselective total syntheses of rubriflordilactone A,^384,385^ a nortriterpenoid natural product which has attracted recent attention due to its moderate anti-HIV activity coupled with low cytotoxicity (Scheme 145, entry 1). After surveying several model systems, it was found that unstable chloropyran intermediate **547**, obtained by treating the corresponding lactol precursor with a mixture of thionyl chloride and ZnCl_2_ (not shown), smoothly reacted via formation of an oxocarbenium ion with siloxyfuran **537b** in the presence of substoichiometric ZnCl_2_ to afford a 1:1 mixture of the butenolide–tetrahydropyran framework of rubriflordilactone A **548** along with its C23-epimer in a 71% combined yield.

A related butanolide–tetrahydrofuran framework is also present in the DEFG ring system of the parent rubriflordilactone B, whose synthesis has been challenged by Peng in 2015 (Scheme 145, entry 2).^386^ Here, upon treatment of the acetate precursor **549** with catalytic Bi(OTf)_3_, the in situ generated cyclic oxonium ion quickly reacted with nucleophilic silyloxyfuran **537a** to give the addition product **550** in 35% yield with the expected (22*S*,23*S*)-*syn* configuration.

An interesting VMAR-type reaction involving the addition of TMSOF **496a** to an in situ generated sulfenium ion appeared in the total synthesis of the anti-inflammatory agents (−)-cephalosporolide E and (+)-cephalosporolide F by Raghavan and co-workers in 2016 (Scheme 145, entry 3).^387^ Treatment of a mixture of α-chloro sulfides **551** (obtained by α-chlorination with *N*-chlorosuccinimide in benzene from the corresponding sulfide, not shown) with **496a** in the presence of ZnBr_2_ afforded the corresponding γ-adduct **552** as a diastereomeric mixture of all possible diastereomers in a 10:2:1:1 ratio respectively in a combined 76% yield. Of all, the (5*R*,1′*R*)- and (5*S*,1′*R*)-configured epimeric couple was found to be the major product of the reaction in accordance to a Felkin–Ahn model in which the medium-sized alkyne substituent would eclipse the sulfenium ion, while the trimethylsiloxy furan would approach it from the *Si* face, opposite to the bulky OTBS group.

### 5.2 Additions to C=N Bonds

#### 5.2.1 Direct Procedures

##### 5.2.1.1 Acyclic Pronucleophiles

Concerning the vinylogous, nucleophilic addition to the C=N functionality, asymmetric and catalytic strategies that can directly functionalize linear, α,β-unsaturated esters at the γ-position by exploiting vinylogous Mannich-type transformations represent useful solutions in organic synthesis, since they may provide γ-homologated δ-amino-derivatives in a chiral, enantiopure format.

**Scheme 146:**
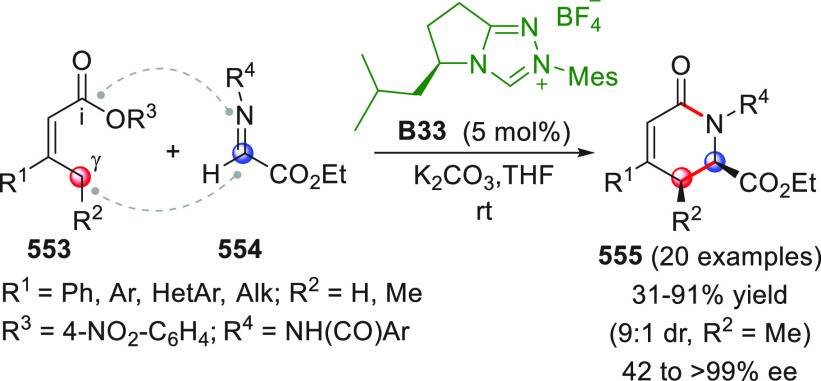


With solid background on organocatalytic ester activation, Chi, Hu, et al. in 2013 developed the first *N*-heterocyclic carbene (NHC)-catalyzed direct γ-functionalization of linear α,β-unsaturated esters of type **553**, that undergo stereoselective addition to glyoxal-derived hydrazones **554** in a highly efficient manner (Scheme 146). Following a careful optimization survey, l-leucine-derived chiral triazolium-based NHC precatalyst **B33** was elected as the most suitable tool to access a wide panel of δ-lactams **555** bearing up to two new stereocenters in good yields (up to 91%) and excellent enantioselectivities (up to >99% *ee*). The authors also proved the usefulness of such optically active lactams by transforming a lactam derivative (R^1^ = Ph and R^2^ = H) into the corresponding chiral pipecolic acid (not shown).

##### 5.2.1.2 Cyclic Pronucleophiles

**Scheme 147:**
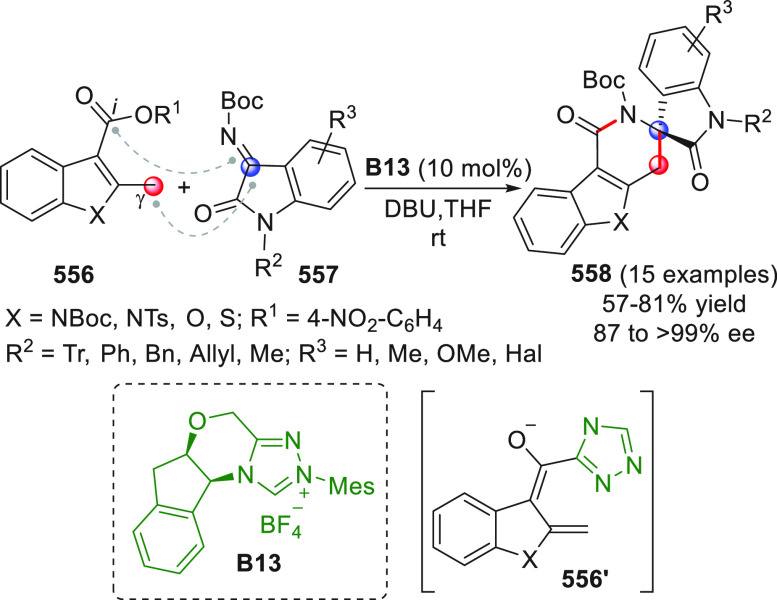


Cyclic ester pronucleophiles could be engaged in γ,ipso-selective [4 + 2] annulations similar to those witnessed for their acyclic counterparts (see Scheme 146), but this time when aromatic cyclic vinylogous esters were employed, dearomative formation of oQDM dienolate intermediates had to be faced (see section 3). In this context, in 2016 Hu et al. reported the construction of optically pure heteroarene-fused δ-lactams **558** bearing a quaternary stereogenic center (Scheme 147),^388^ facing the dearomatizative γ-enolization of 2-methyl-heteroarene-3-carboxylic esters of type **556** induced by NHC **B13** catalyst and DBU base. Highly reactive heterocyclic oQDM intermediates **556′** underwent highly enantioselective stepwise [4 + 2] annulation reactions with isatin-derived ketimines **557** to afford the constrained heteroarene-fused δ-lactams **558** in good yields (up to 81%) and high enantiocontrol (up to >99% ee).

**Scheme 148:**
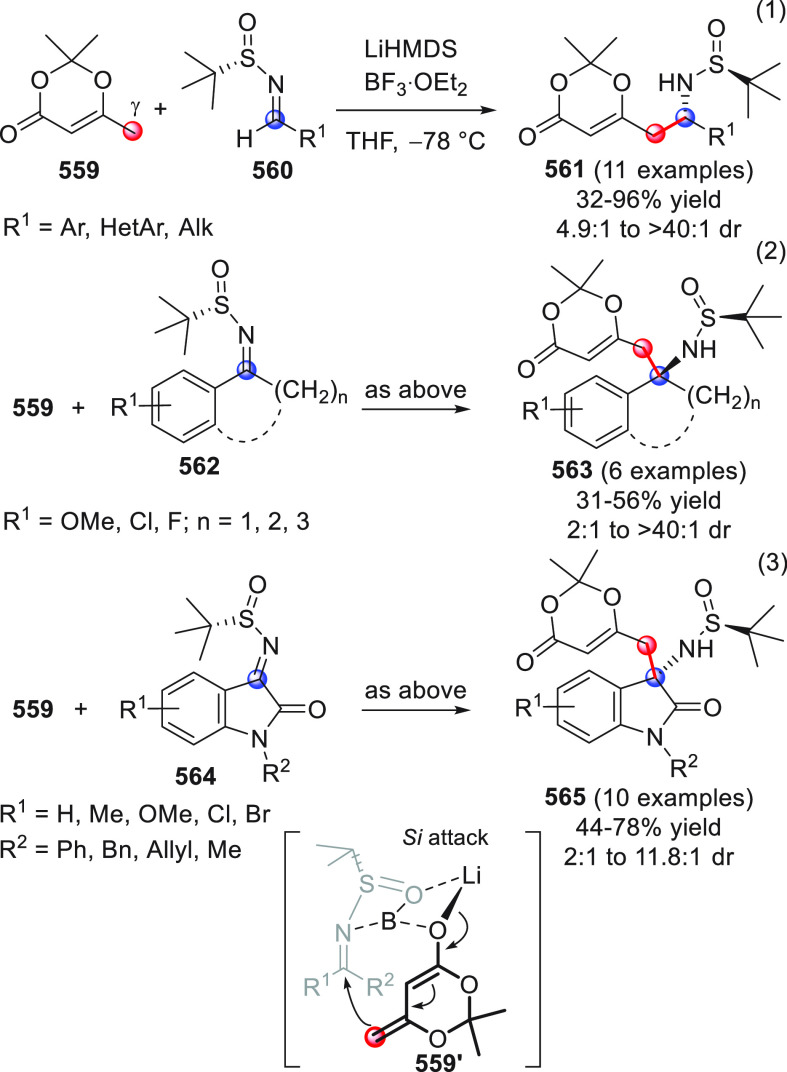


A diastereoselective, direct, two-component vinylogous Mannich reaction between chiral *N*-*tert*-butanesulfinyl imines **560**(389) and pronucleophilic dioxinone **559** was developed in 2017 by Chen, Zhang, et al. for the synthesis of δ-amino acid derivatives (Scheme 148).^390^ After a systematic screening of reaction conditions, it was found that the combination of lithium bis(trimethylsilyl)amide (LiHMDS) to generate the dioxinone-derived lithium dienolate, combined with BF_3_·OEt_2_ to activate the preformed imine component, proved to be the most efficient choice. A variety of aromatic and aliphatic aldimines of type **560** (Scheme 148, eq 1), ketimines **562** (eq 2), and isatin-derived ketimines **564** (eq 3), mostly bearing a chiral *tert*-butanesulfinyl auxiliary group at the nitrogen atom, were suitable substrates for this process, providing the corresponding adducts **561**, **563**, and **565** with varied levels of efficiency and generally with a good level of diastereocontrol.

To rationalize this BF_3_·Et_2_O-mediated vinylogous Mannich reaction, the authors provided a transition state model (Scheme 148, bottom), where the imine component, activated by the boron-centered Lewis acid, is engaged in a six-membered chairlike network with lithium ion that guides the *Si* face addition of the dioxinone-derived dienolate **559′** to the imine, leading to the *S*-configured products.

**Scheme 149:**
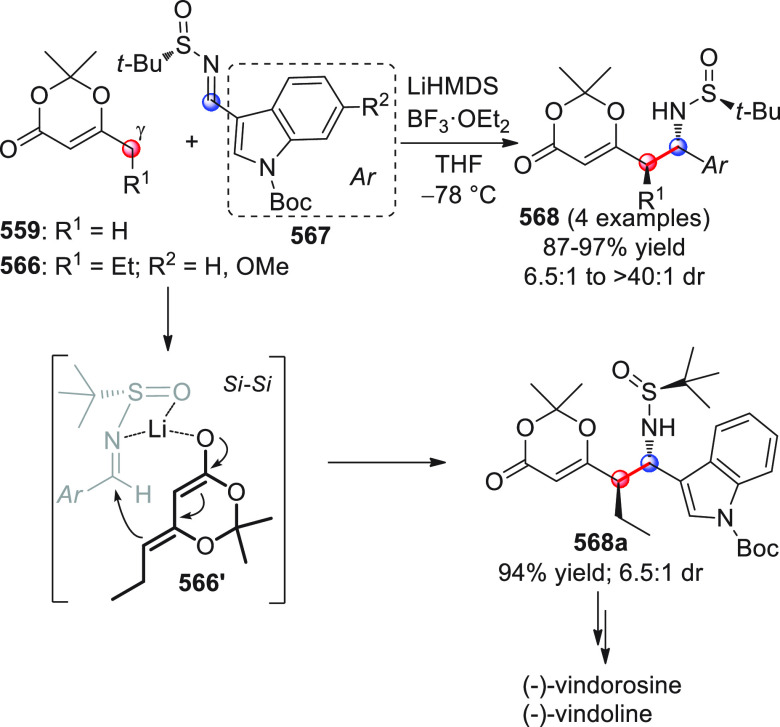


Recently, the same group reported a couple of works in which the just described direct, vinylogous Mannich-type addition to *N*-*tert*-butanesulfinyl imines was applied to the enantioselective synthesis of terpene indole alkaloids (−)-vindorosine^391^ and (−)-vindoline,^392^ whose challenging core structure is embedded in clinically important anticancer drugs such as vinblastine and vincristine (Scheme 149).

An initial study on model substrates, such as pronucleophilic dioxinones **559** and **566** and preformed indolyl *N*-*tert*-butanesulfinyl imines **567** confirmed the utility of the previously disclosed reaction conditions based on BF_3_·OEt_2_ and LiHMDS couple (Scheme 149). Indeed, treatment of **559** or **566** with lithium bis(trimethylsilyl)amide in THF at −78 °C, and subsequent reaction with indolyl-*N*-*tert*-butanesulfinylimine **567** activated by BF_3_·OEt_2_, resulted in the formation of the corresponding *N*-*tert*-butanesulfinylamines **568** on a gram scale, in high yields (up to 97%) and good to excellent diastereomeric ratios (up to >40:1). In particular, prostereogenic ethyl dioxynone **566**, coupled with unsubstituted *N*-Boc indolyl imine **567a** (R^2^ = H), gave the corresponding (4*R*,5*S*,*Ss*)-*anti*-adduct **568a** in 83% yield predominantly, accompanied by small amounts of its (4*S*,5*R,Ss*)-*anti*-diastereoisomer (6.5:1 dr). Substrate **568a** was then further elaborated to complete the total synthesis of the vindorosine and vindoline core, as planned (not shown). To account for the observed (4*R*,5*S*,*Ss*)-*anti*-selectivities resulting from a *Si–Si* approach, the authors recalled the previously described transition state (Scheme 148) adding an alternative, putative model in which the boron atom BF_3_ would not be involved (Scheme 149, bottom).

As already commented for the direct, vinylogous aldol reaction, one of the most exploited class of vinylogous pronucleophiles consists of conjugated or unconjugated furanone derivatives to access chiral, enantiopure γ-homologated butenolides. In this context, their use as vinylogous pronucleophiles in asymmetric Mannich-type addition to imine substrates affords chiral δ-amino γ-butenolides which are common motifs in a variety of natural products and pharmaceutical compounds, besides being also useful synthetic intermediates.

**Scheme 150:**
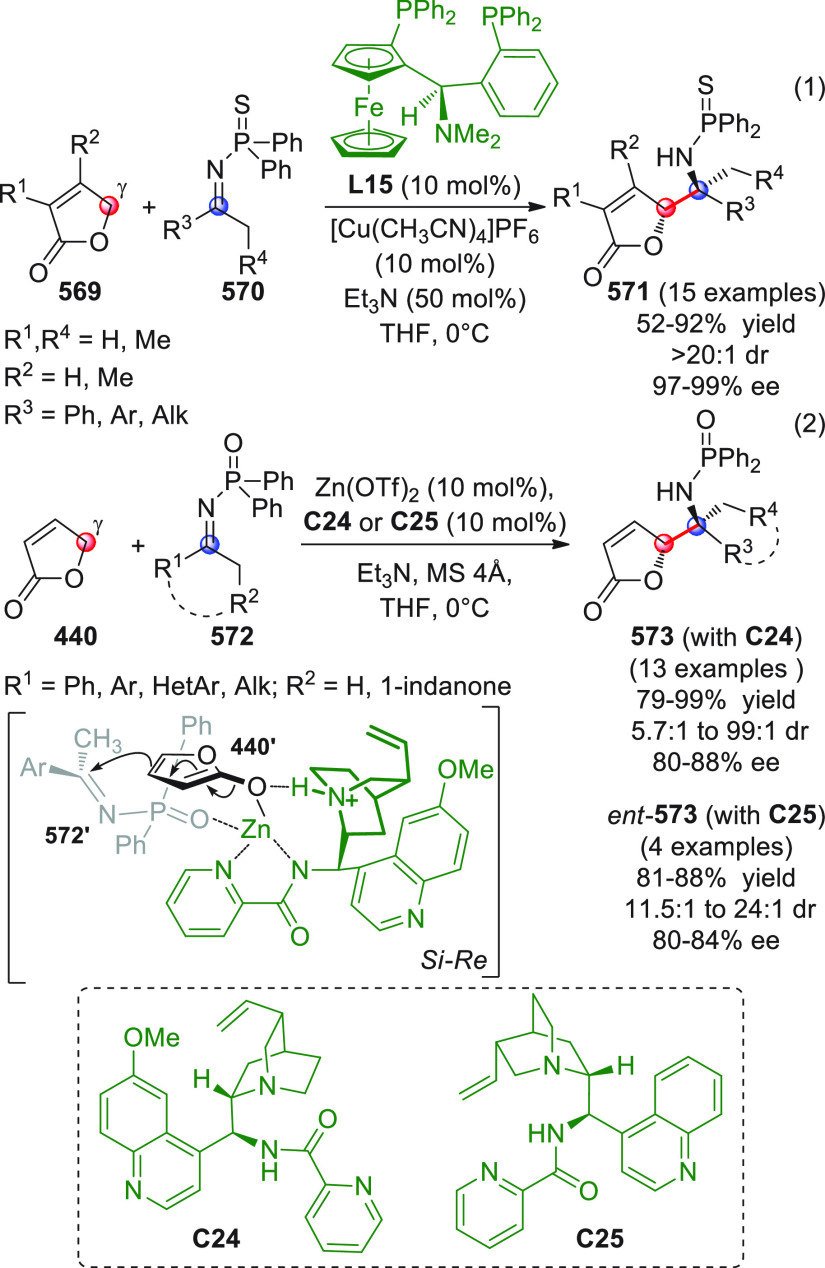


The synthetic utility of γ-butenolides led Shibasaki and co-workers to develop the first, direct, and catalytic asymmetric vinylogous Mannich reaction of furanones of type **569** with nonactivated *N*-thiophosphinoyl ketimines **570** (Scheme 150, eq 1).^393^

Capitalizing on their own previous ventures on the activation of such ketimines by soft Lewis acids through soft–soft interactions, Shibasaki envisaged that a cooperative catalytic system containing a soft Lewis acid and a hard Brønsted base would be a viable strategy to promote the catalytic generation of dienolates from the pronucleophilic γ-butenolide while simultaneously activating the ketimine component. Indeed, after a deep screening of Lewis acids and amine bases, it was found that several γ-crotonolactones of type **569** and *N*-thiophosphinoyl ketimines **570** smoothly reacted with a binary catalyst (10 mol %) composed of [Cu(CH_3_CN_4_)]PF_6_/(*R,R*_*P*_)-taniaphos (**L15**) using Et_3_N (50 mol %) in THF at 0 °C, to give optically pure, vinylogous Mannich adducts **571** in moderate to high yields (up to 92%) as the sole *anti*-isomers (dr > 20:1). The reaction proved to be quite general, so that both aromatic (acetophenone-derived) and aliphatic ketimines reacted with γ-crotonolactone and its 3- or 4-methyl congeners with almost equal efficiency.

An interesting alternative was developed several years later by Nakamura and co-workers, who reported the direct, enantioselective vinylogous Mannich reaction of unsubstituted γ-crotonolactone **440** to a series of aromatic and aliphatic *N*-phosphinoyl ketimines **572** using Zn(OTf)_2_ (10 mol %) coupled with an original quinine-derived picolinamide **C24** used in combination with Et_3_N (1.0 equiv) as the base (Scheme 150, eq 2).^394^ Indeed, the reaction of **440** with a set of cyclic and acyclic ketimines **572** gave access to the corresponding optically active δ-phosphinolylamino-butenolides **573** in high yields (up to 99%) and with excellent diastereo- and enantioselectivities (up to 99:1 dr, and up to 88% *ee*). Furthermore, using the *quasi*-enantiomeric quinidine-derived amide **C25** afforded the corresponding butenolides *ent*-**573** with comparable yields and stereoselectivities. To clarify the observed selectivity of the direct VMnR between **440** and **572**, MO calculations of the transition state were carried out, unveiling a network in the putative transition state (Scheme 150, bottom) in which the zinc(II) cation would coordinate two nitrogen atoms from the picolinoyl moiety, two oxygen atoms from the dienolate **440′**, and a phosphoryl group. Also, the proton attached to the quinuclidine moiety coordinates the oxygen atom of the dienolate. In this stacked bonding network, the *Si* face of the dienolate approaches the *Re* face of the ketimine to avoid steric repulsion of the diphenylphosphinyl group to give the observed products.

**Scheme 151:**
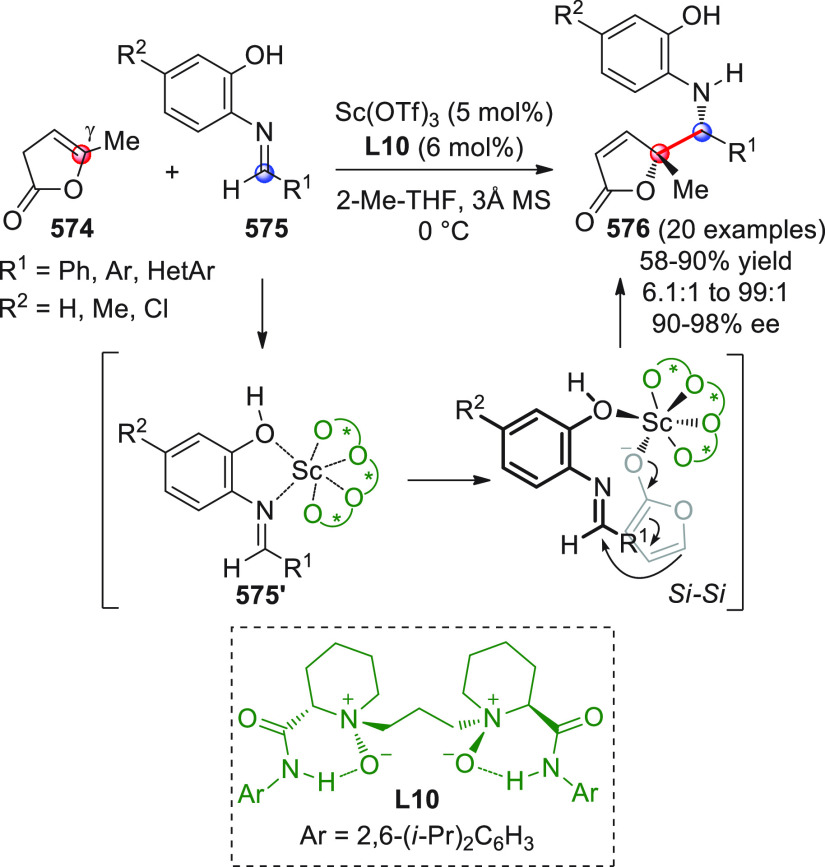


In 2011 Feng and co-workers were the first who demonstrated how deconjugated butenolides such as α-angelica lactone **574** were also viable vinylogous nucleophiles to be used in metal-catalyzed enantioselective VMnR to provide chiral δ-amino γ,γ-disubstituted butyrolactones. (Scheme 151)^395,314^ Interestingly, in contrast with previously reported methodologies in which **574** was activated to the corresponding dienolate by base-promoted α-deprotonation, Feng envisioned the possibility to activate such donors through an in situ-generated O-bond metal dienolate by the sole Lewis acid catalysis. After having scrutinized a series of metal–ligand couples, the *N*,*N*′-dioxide **L10·**Sc(OTf)_3_ complex was elected as the best catalytic system to promote the VMnR between deconjugated α-angelica lactone **574** and a set of various aromatic *N*-anisidine aldimines of type **575**, providing the corresponding δ-amino butenolide scaffolds **576** bearing adjacent quaternary and tertiary stereocenters, in good yields (up to 90%) and excellent diastereo- and enantioselectivities (up to 99:1 dr and up to 98% ee). Interestingly, the α,β-unsaturated furanone congener failed to undergo the VMnR and no product was observed under **L10**/Sc(OTf)_3_ catalysis. The electronic effects of the anisidine group were also investigated, revealing that the electronic property rather than the steric hindrance of substituents on the phenyl ring of aromatic aldimines had an impact on the enantiocontrol of the reaction; accordingly, the substrates with electron-withdrawing substituents gave higher ee values than those with electron-donating ones. A mechanism of this highly enantioselective process was proposed, suggesting that the use of the **L10**/Sc(OTf)_3_ couple is likely to generate a hexacoordinate scandium-centered intermediate **575′** from aldimine **575** After α-angelica lactone **574** is added, a new chiral scandium intermediate is generated, in which the *Si–Si* attack of the dienolate to the aldimine provided access to the targeted (5*R*,1′*R*)-configured γ,γ-disubstituted butenolides **576**.

**Scheme 152:**
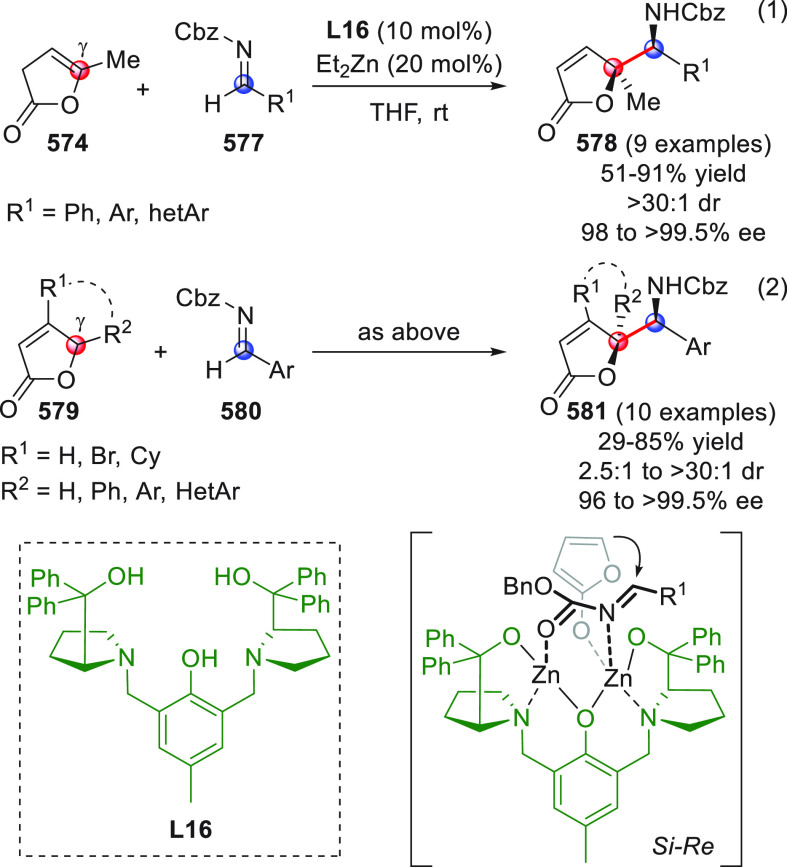


The first direct, vinylogous Mannich reaction which utilizes easily cleavable *N*-Cbz imines and either conjugated and unconjugated α,β- or β,γ-butenolides bearing a variety of substitution patterns was recently developed by the Trost group,^396^ exploiting their previously developed Zn-ProPhenol complex **L16**·Et_2_Zn whose ability in catalyzing other asymmetric transformations, including aldol reactions, had widely been proved.^397^ As described in Scheme 152 (eq 1), ProPhenol ligand **L16** (10 mol %) and Et_2_Zn (20 mol %) in THF at rt efficiently catalyzed the VMnR between α-angelica lactone **574** and a series of Cbz-protected aromatic aldimines **577**, yielding optically pure δ-amino butenolides **578** in good yields (up to 99%) and almost complete stereocontrol (up to >99.5% ee). Of note, under similar reaction conditions, this catalyst system successfully worked also with conjugated furanones **579** and aromatic aldimines **580** (Scheme 152, eq 2) affording chiral, enantiopure butenolides **581** in comparable yields and stereoselectivities. Based on previous studies on other Zn-ProPhenol catalyzed Mannich reactions, the authors proposed a transition state based on a two-point binding model in which the nitrogen and the carbamate oxygen of the imine binds to a zinc atom, generating a complex which directs the addition of the butenolide to the *Re* face of the imine (Scheme 152, bottom).

**Scheme 153:**
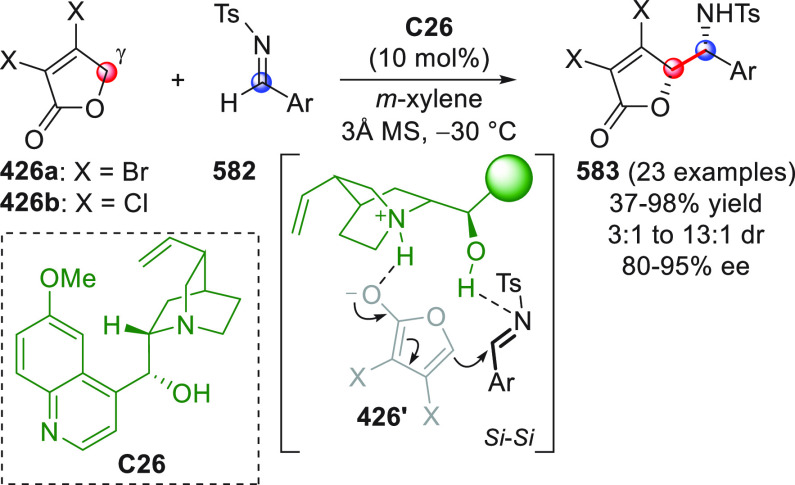


Finally, an organocatalyzed version of the direct, asymmetric, *syn*-selective VMnR of 3,4-dihalofuran-2(5*H*)-ones **426a** and **426b** with a series of *N*-tosyl aromatic aldimines **582** was developed by Xu, Wang, et al. in 2012, using natural quinine **C26** as the catalyst (Scheme 153).^398^ A series of different aldimines derived from aromatic aldehydes bearing both electron-withdrawing and electron-donating groups were suitable electrophiles for the direct VMnR with halofuranones, giving the corresponding *syn*-configured δ-amino γ-butenolides **583** in excellent yields (up to 98%) and enantioselectivities (up to 95% ee). To account for the obtained selectivity and based on previous achievements on similar organocatalyzed reactions, a transition state was proposed (Scheme 153, bottom). Accordingly, the quinuclidine moiety of quinine activates the 3,4-dihalofuran-2(5*H*)-one **426**, generating the nucleophilic dienolate **426′**, while the hydroxyl group within the catalyst would activate the imine through hydrogen bonding interaction. The chiral quinine scaffold nicely orchestrates the *Si–Si* approach of the dienolate and the imine through noncovalent interactions, promoting the formation of the targeted butenolides **583**.

#### 5.2.2 Indirect Procedures

##### 5.2.2.1 Acyclic Pronucleophiles

**Scheme 154:**
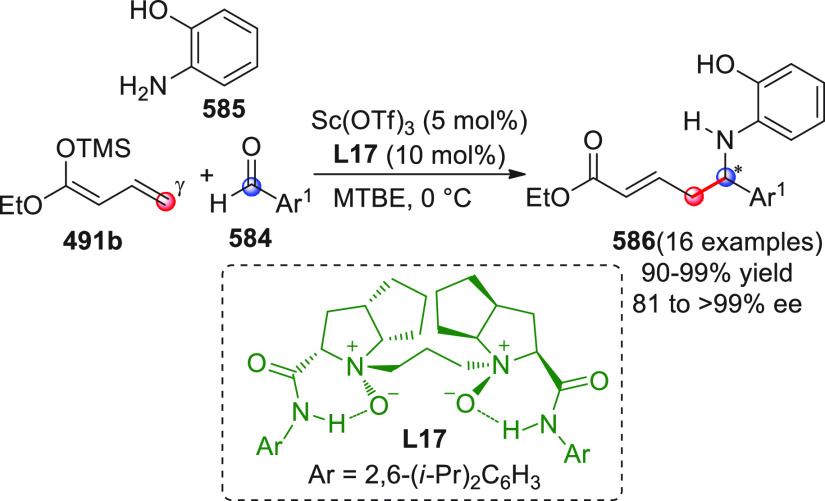


A highly selective metal-catalyzed, three-component vinylogous Mukaiyama-Mannich reaction (VMMnR) between acyclic silyl dienol ether **491b** and aromatic aldehydes **584** and 2-aminophenol **585** was accomplished by Liu, Feng, et al. in 2010, using chiral *N,N*′-dioxide **L17**·Sc(OTf)_3_ complex as the catalyst (Scheme 154).^399^ A variety of aromatic aldehydes were found to be suitable substrates for the reaction and the desired δ-amino-α,β-unsaturated esters **586** were obtained in 90–99% yields with good to excellent enantioselectivities (up to >99% ee).

**Scheme 155:**
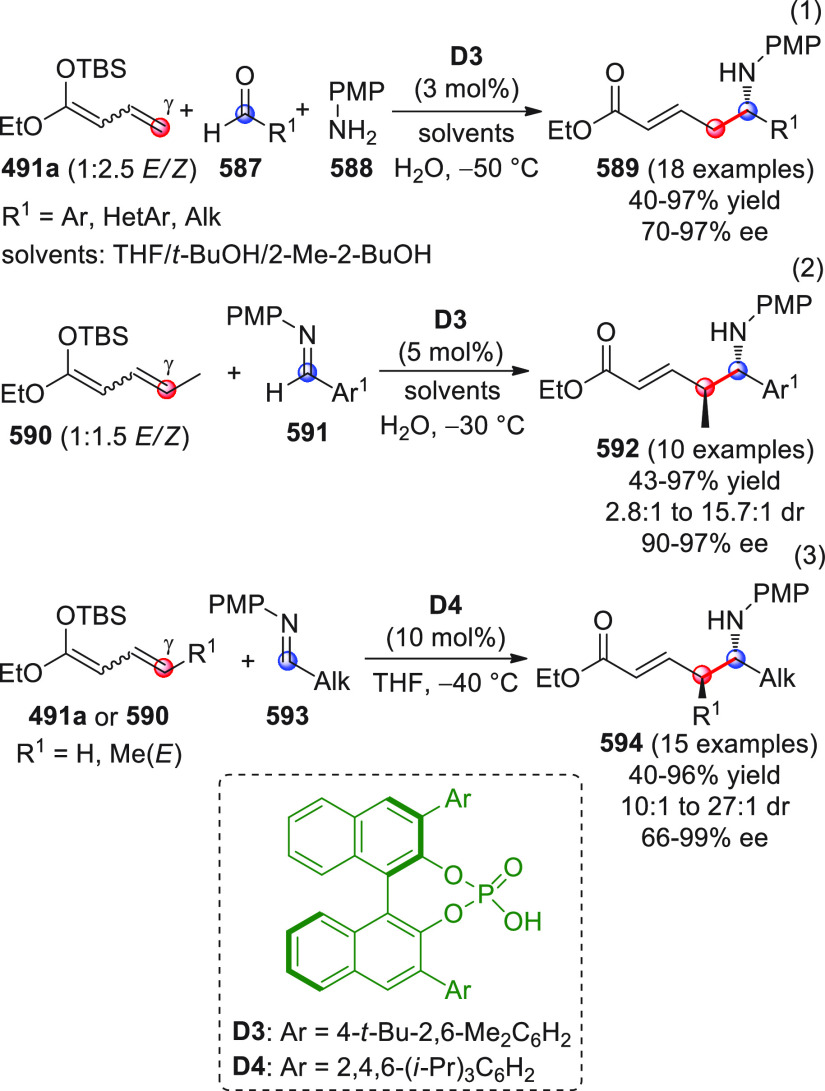


In the same year Schneider and co-workers^400^ reported the chiral Brønsted acid-catalyzed, two- or three-component enantioselective VMMnR of acyclic silyl dienolate **491a** and **590** with several aromatic and aliphatic imines, providing enantioenriched δ-amino-α,β-unsaturated esters **589** and **592** in excellent stereoselectivities (Scheme 155, eqs 1 and 2). Capitalizing on a previous report,^401^ they discovered a superior second-generation Brønsted acid catalyst such as BINOL-based phosphoric acid **D3**, in promoting a high enantioselective three-component VMMnR starting from the respective aldehydes **587**, *p*-anisidine **588**, and silyl dienolate **491a** in a solvent mixture of equal amounts of *t*-BuOH, 2-methyl-2-butanol, and THF at −50 °C, in the presence of 1 equiv of water (Scheme 155, eq 1).

Under these optimized conditions, aromatic, heteroaromatic and aliphatic aldehyde acceptors **587** were suitable substrates, generating the corresponding γ-adducts in high yields (up to 97%) and enantioselectivity (up to 97% ee). Furthermore, under similar reaction conditions when prostereogenic γ-substituted silyl dienolate **590** coupled to preformed imine **591**, a two-component version of the reaction (Scheme 155, eq 2) provided the corresponding optically enriched *anti*-adducts **592** in good yields (up to 97%) and stereoselectivities. Interestingly, the reaction of the corresponding 3*Z*-configured silyl dienolate congener (3*Z*)-**590** was much less efficient giving predominantly the *syn*-configured adduct **592** (1.5:1 *syn*/*anti*) in 41% yield and 72% ee (not shown). Mechanistic investigations including NMR spectroscopy and mass spectrometry unveiled the role of the protic reaction medium, which traps the cationic silicon species as silanols to regenerate the chiral Brønsted acid catalyst (not shown). The substrate scope of the catalytic, enantioselective VMMnR involving acyclic silyl ketene acetals was later extended to more challenging aliphatic aldimine electrophiles. In this context, a modified and highly useful protocol was developed using BINOL-based phosphoric acid **D4** (10 mol % in THF, at −40 °C) as the catalyst of choice (Scheme 155, eq 3).^402^ Either substituted or unsubstituted silyl dienolates **491a** and **590** were used in combination with a set of linear and branched aliphatic imines **593** providing the corresponding vinylogous adducts **594** in good to excellent yields (up to 96%) and high stereocontrol (up to 29:1 dr from **590**, and up to 99% ee). Ester moieties, halogen atoms, and alkynes were also well tolerated in this process.

**Scheme 156:**
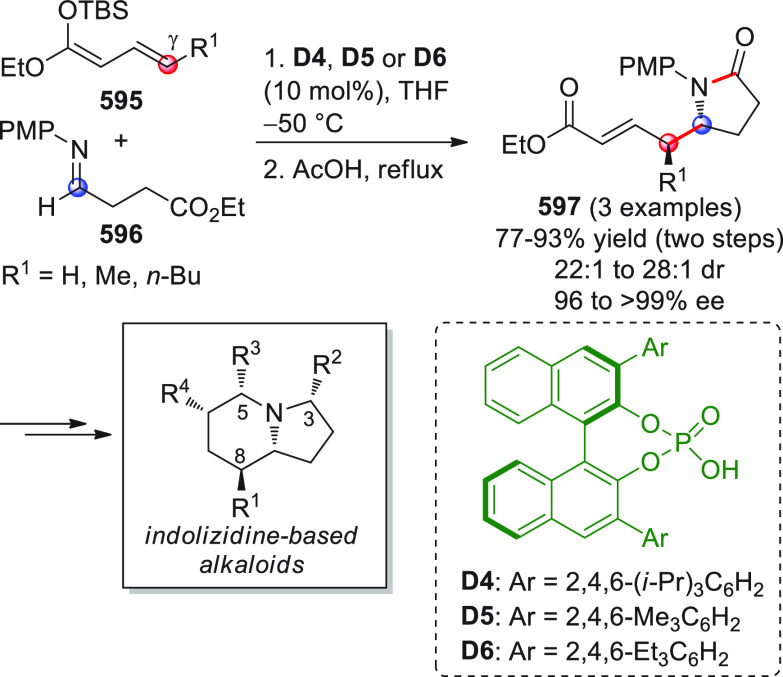


The disclosed VMMnR protocol served as the key stereoselective step in the total synthesis of 16 diversely substituted and enantioenriched indolizidine-based alkaloids (Scheme 156).^403,404^ Here, using differently substituted catalysts **D4**, **D5**, or **D6** to promote the addition between simple (R^1^ = H) and prostereogenic (R^1^ = Me) dienolates **595** to a suitable in situ-formed aliphatic aldimine **596**, the corresponding *anti*-configured γ-adduct intermediates were formed on a large scale. Upon treatment of the crude with acetic acid at reflux a subsequent cyclization step took place, providing key butyrolactam intermediates **597** in high overall yields (up to 93%) and excellent diastereo- and enantioselectivities (up to 28:1 dr and >99% ee).

**Scheme 157:**
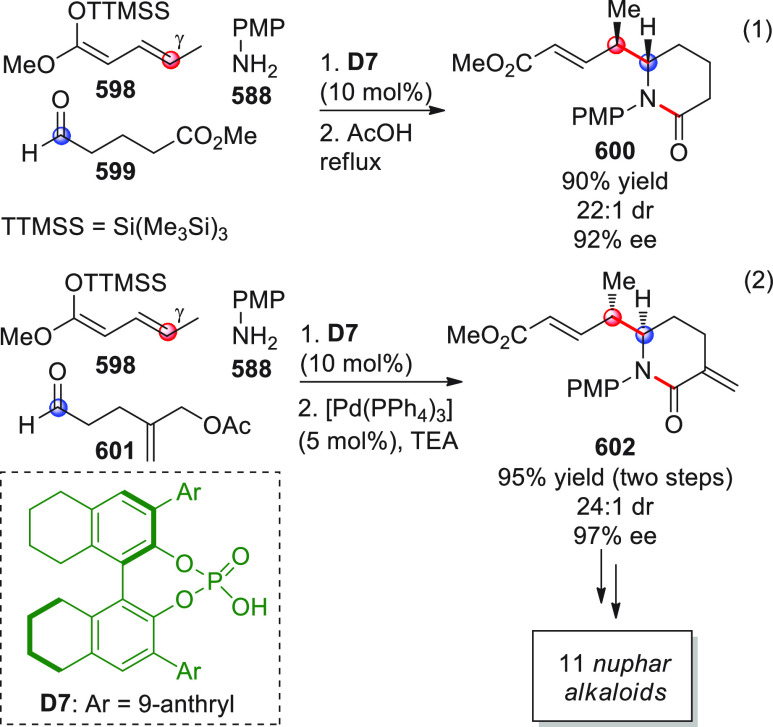


Another smart application of Schneider’s protocol was implemented by Eastman, Wu, et al. in 2016 for the enantioselective formal syntheses of diverse dimeric and monomeric sulfur-containing quinolizidine triterpenoids nuphar alkaloids (Scheme 157).^405^ The syntheses involved, as the key step, the development of highly enantioselective Brønsted acid-catalyzed three-component VMMnR between supersilyl dienol ether **598**, aliphatic aldehydes **599** or **601**, and *p*-anisidine **588** as the amine source, to provide useful γ-adduct intermediates that were easily converted into the corresponding piperidine derivatives **600** or **602** via acetic acid or Pd-catalyzed annulations (Scheme 157, eqs 1 and 2).

**Scheme 158:**
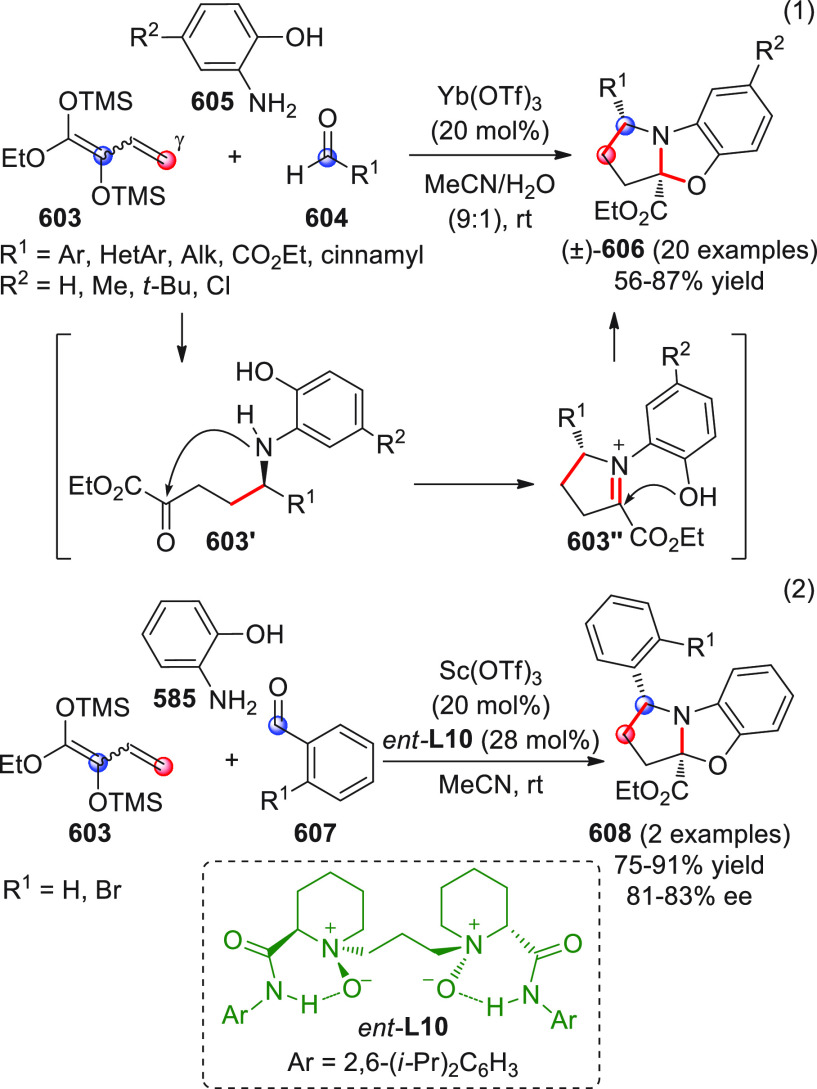


Paralleling this chemistry, Schneider and co-workers also focused on the reactivity of bissilyldienediolate **603** (Scheme 158): an α-keto ester homoenolate equivalent which may react as a 1,3-zwitterionic synthon in cascade processes providing new synthetic opportunities.

In this context, Schneider, Boomhoff et al. in 2012 reported a novel, metal-catalyzed, one-pot [3 + 2] cycloannulation process generating tetrahydropyrrolo[2,1-*b*]benzoxazoles of type (±)-**606** with two new stereogenic centers in good yields (up to 87%) as single isomers (Scheme 158, eq 1).^406−409^ In particular, the developed stepwise process consisted of a first, Yb(OTf)_3_-catalyzed three-component VMMnR between bissilyldienediolate **603**, 2-aminophenols **605**, and a set of diverse aromatic and aliphatic aldehydes **604**, yielding the corresponding γ-adduct intermediates, which underwent fast hydrolytic cleavage to give a highly reactive α-keto ester **603′**. This intermediate could then engage the imine in a cyclocondensation reaction generating the *N,O*-acetal **606** spontaneously, via formation of the cyclic iminium ion intermediate **603′′**. Furthermore, a catalytic, enantioselective variant was preliminarily attempted on benzaldehydes **607**, using the chiral Sc(OTf)_3_·*ent*-**L10** as catalyst system of choice (Scheme 158, eq 2). Under slightly different reaction conditions, the corresponding products **608** could be obtained with good yields (91–75% respectively) and acceptable enantioselectivities (81–83% ee).

**Scheme 159:**
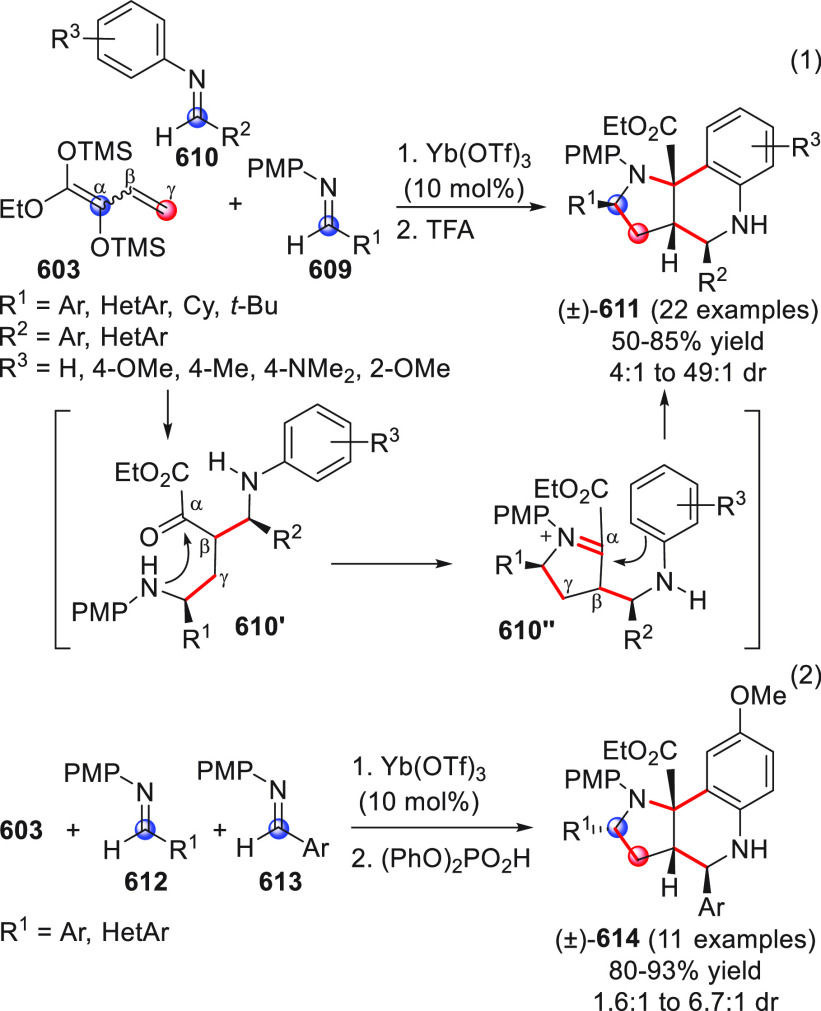


The same group used bis-silyldienolether **603** in the sequential addition to two imine substrates in a tandem, divergent vinylogous Mannich–Mannich–Pictet–Spengler process to generate chiral, polyfunctionalized hexahydropyrrolo[3,2-*c*]quinolines (±)**-611** and (±)**-614** in a one-pot operation (Scheme 159, eqs 1 and 2).^410^ Under nonaqueous conditions (DME at rt), Yb(OTf)_3_ (10 mol %) promoted a first VMMnR of **603** to a first added imine **609**, generating the corresponding silyl enol ether intermediate that, after the addition of a substoichiometric loading of a second Brønsted acid catalyst such as TFA, engaged a second imine **610** in a “normal” Mannich reaction to provide a diamino α-keto ester intermediate **610′** (Scheme 159, eq 1).

This highly reactive intermediate then cyclized to give the corresponding iminium ion **610′′**, which is finally trapped by the electron-rich anisidine moiety in a Pictet–Spengler reaction, affording the targeted pyrroloquinoline (±)**-611**. Under this protocol, a broad range of aromatic and aliphatic imines were tolerated, providing the corresponding products in good yields (up to 85% yield) and high diastereoselectivities (up to 49:1 dr).

Interestingly, a divergent option was viable by careful adjustment of reaction conditions (MeCN as solvent, at rt, using (PhO)_2_PO_2_H instead of TFA), so that **603** and added imines **612** and **613** (Scheme 159, eq 2) afforded the corresponding products (±)**-614** in high yields (up to 93%) and moderate diastereoselectivity (up to 6.7:1 dr).

**Scheme 160:**
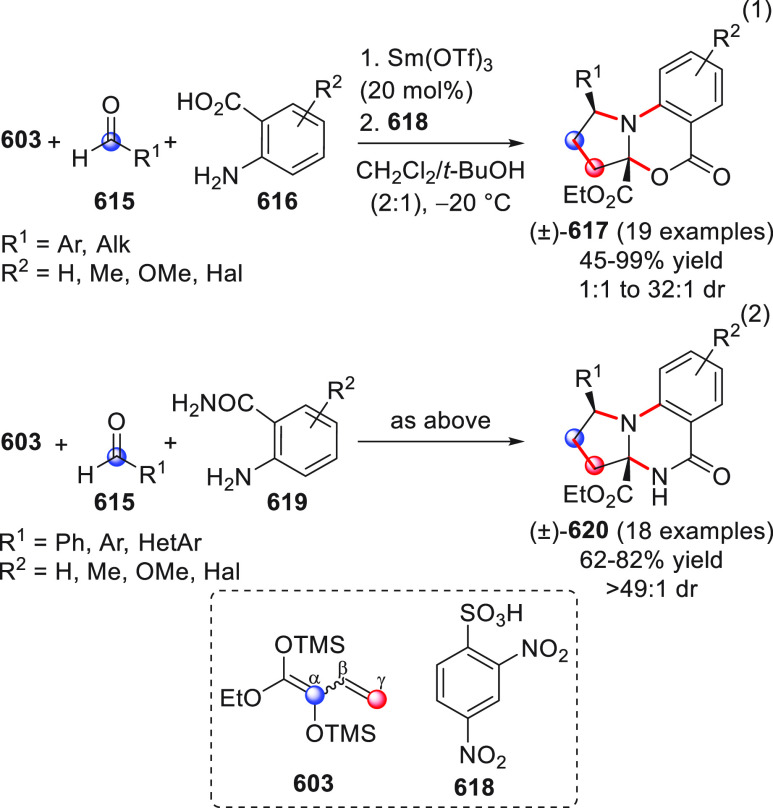


More recently, a conceptually similar stereocontrolled three-component [3 + 2]-cycloheteroannulation involving bis-silyldienediolate **603** and imines derived from 2-aminobenzoic acid and 2-aminobenzamide derivatives **616** and **620**, catalyzed by Sm(OTf)_3_ in the presence of 2,4-dinitro benzenesulfonic acid (**618**, DNBSA), furnished highly substituted pyrrolo[1,2-*a*]benzoxazinones (±)**-617** or pyrrolo[1,2-*a*]quinazolinones (±)**-620**, respectively, in good overall yields (Scheme 160, eqs 1 and 2).^411−413^

##### 5.2.2.2 Cyclic Nucleophiles

The vinylogous Mukaiyama–Mannich reaction (VMMnR) of lactone-derived silyl ketene acetals with imines or iminium ions leading to δ-amino α,β-unsaturated carbonyl compounds is a useful transformation which has become a powerful methodology for the efficient synthesis of highly functionalized heterocycles, bioactive natural products, and pharmaceuticals. In this context, the past decade has witnessed with the concomitant development of new VMMnR methodologies (particularly in the field of asymmetric metal- and organocatalyzed transformations), the exploitation of previously assessed methodologies to the total synthesis of bioactive natural products.

In this context, vinylogous additions of 2-silyloxyfurans to C=N double bonds constitute very attractive processes, allowing the incorporation of oxygen functionalities into nitrogen-containing carbon frameworks. Furthermore, both enantioselective versions using chiral catalysts and diastereoselective versions using chiraly auxyliaries have been documented and clearly reported.

**Scheme 161:**
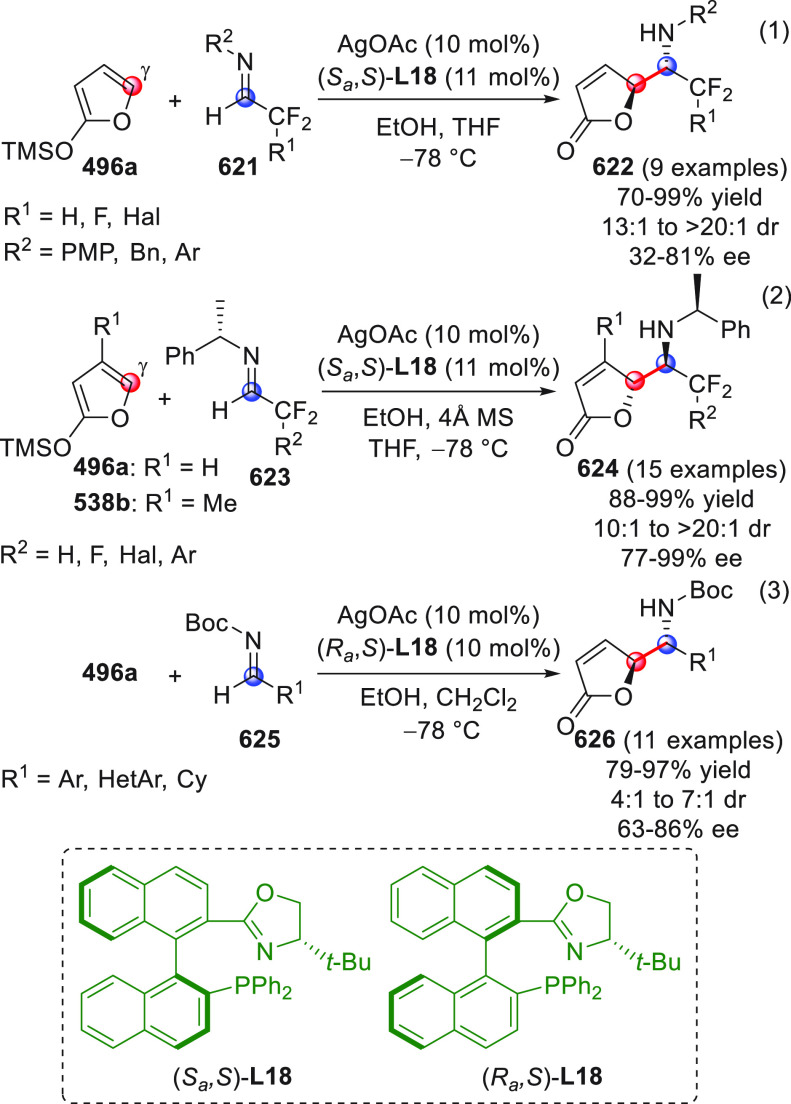


In 2010, the group of Shi and co-workers^414^ developed a silver(I)-catalyzed catalytic, asymmetric VMMnR of trimethylsiloxyfuran **496a** with a set of fluorinated aldimines **621** in the presence of a chiral phosphine–oxazoline ligand (*S*_*a*_,*S*)-**L18** derived from *S*-BINOL (Scheme 161, eq 1). The reaction, carried out in THF at −78 °C, in the presence of EtOH (1.8 equiv) as a protic, silicon scavenging additive, afforded the corresponding 5,1′-*anti*-configured γ-adducts **622** in good to excellent yields (up to 99%), along with moderate to good enantioselectivities (up to 81% ee).

An extension of such protocol was later devised by the same group, who developed an *anti*-selective VMMnR between **496a** and readily available fluorinated aldimines **623** bearing a chiral auxiliary group [(*S*)-1-phenylethyl group] (Scheme 161, eq 2).^415^ Using AgOAc-(*Sa,S*)-**L18** (10 mol %) catalytic complex, EtOH (1.8 equiv) in THF at −78 °C, a set of “*matched*” chiral, fluorinated γ-butenolides of type **624** were accessed in high yields (up to 99%), good to excellent enantiomeric excesses (up to 99% ee), and up to >20:1 dr. In addition, 4-methyl-substituted siloxyfuran **538b** was also tested as nucleophile in the VMMnR to **623**, affording the corresponding adducts in comparable yields and stereoselectivites.

A similar silver(I)-catalyzed protocol was also applied to the catalytic, asymmetric VMMnR to *N*-Boc aldimines **625** (Scheme 161, eq 3).^416^ Indeed, treatment of **496a** and imine **625** in the presence of catalytic quantities of chiral phosphine-oxazoline ligand (*Ra,S*)-**L18** and AgOAc in CH_2_Cl_2_, in the presence of stoichiometric EtOH at −78 °C, provided the corresponding 5,1′-*anti*-configured δ-aminobutenolides **626** in good to high yields (up to 97%) along with moderate to good diastereo- and enantioselectivities (up to 7:1 dr and 86% ee).^417−419^

**Scheme 162:**
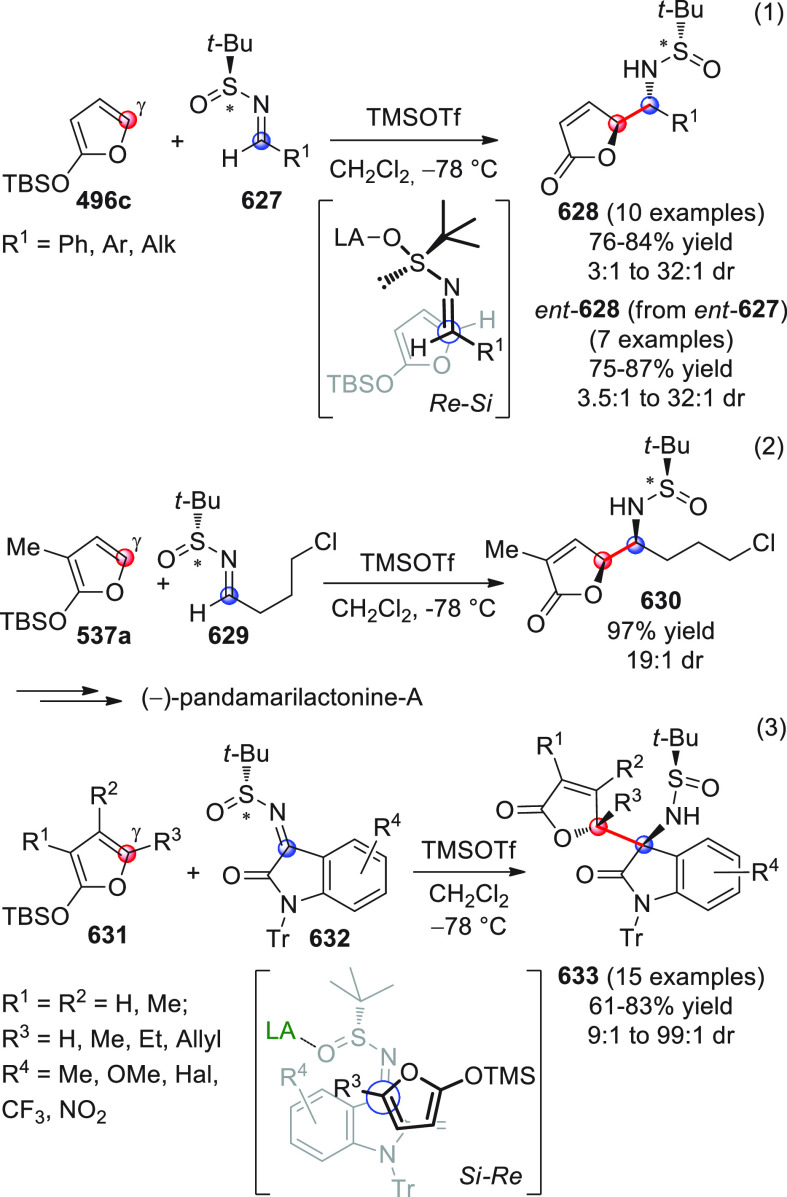


A conceptually different approach was devised by Huang et al. in 2011, who developed a diastereoselective VMMnR of cyclic sililoxyfuran **496c** with preformed, aromatic, or aliphatic chiral *N*-*tert*-butanesulfinimines (*t*-BS-imines or Ellman’s imines) of type **627** to provide chiral, enantiopure δ-aminobutenolides **628** as versatile chiral precursors to access diverse functionalized heterocycles (Scheme 162, eq 1).^420^ Under the optimized reaction conditions, TBSOF **496c** reacted in CH_2_Cl_2_ at −78 °C with either **627** or *ent*-**627** in the presence of TMSOTf to afford the corresponding 5,1′-*anti*-configured 5-aminoalkylbutenolides **628** or *ent*-**628** in good yields with dr ranging from 3:1 to 32:1. To account for the observed selectivity, a plausible transition state was described in which a monocoordinated complex between the Lewis acid and the sulfoxide moiety induced a preferred conformation of the imine which determined the favorable approach of **496c** to the less sterically hindered *Si* face of the chiral imine **627**. A similar methodology was exploited by the same group for the synthesis of diverse hydroxylated piperidine alkaloids and azasugar lactams (not shown).^421^

A rare example of *syn*-selective VMMnR involving *N*-*tert*-butanesulfinimine **629** was devised by the Huang’s group using 2-methyl-TBSOF **537a** instead of **496c** (Scheme 162, eq 2).^422^ Quite surprisingly, under the previously disclosed optimized conditions, TMSOTf (1.0 equiv) in CH_2_Cl_2_ at −78 °C afforded the optically pure, *syn*-configured adduct **630** in a high 97% yield. Butenolide **630** was then used as key intermediate in a three-pot, protecting group-free total synthesis of bioactive natural product (−)-pandamarilactonine-A.

Another highly regio- and diastereoselective TMSOTf-promoted VMMnR involving isatin-derived chiral *N*-*tert*-butanesulfinyl ketimines **632** and silyloxyfurans **631** was introduced by Singh and co-workers in 2014 (Scheme 162, eq 3).^423−425^ The method provided a clean entry to a wide range of challenging quaternary 3-aminooxindole butenolides **633** in good yields (up to 83%) and high diastereoselectivities (up to 99:1 dr). The authors proposed a *syn*-periplanar transition state where the bulky *tert*-butyl group hindered the *Si* face of the ketimine favoring the attack of the silyloxy furans on the *Re* face of **632**, producing the observed *anti*-(5*R,*1′*R*)-configured adducts **633**.

**Scheme 163:**
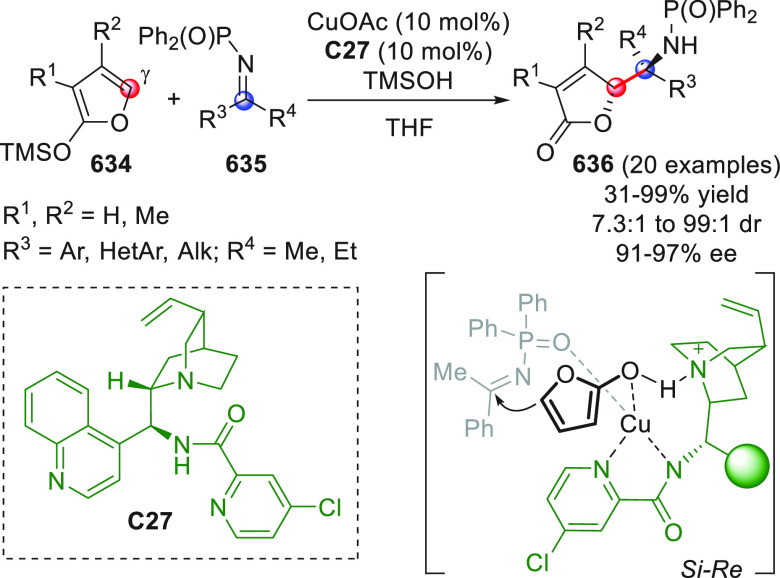


In 2013, an enantioselective VMMnR of silyloxufuran nucleophiles of type **634** and *N*- diphenylphosphinoyl ketimines of type **635** was successfully achieved by Nakamura and co-workers using Cu(II)-cinchona alkaloid picolinamide complex as the catalyst (Scheme 163).^426^

After a first optimization survey, 9-amino-9-deoxy-*epi*-cinchonidine-derived picolinamide **C27** was elected as the best chiral ligand for the CuOAc-promoted VMMnR of a set of between methylated and nonmethylated silyloxyfurans **634** and aromatic, heteroaromatic, and also aliphatic ketimines to provide the corresponding (*O,N*)-*anti*-configured δ-amino-δ,δ-disubstituted butenolides **636** in high yields (up to 99%) and excellent diastereo- and enantioselectivities (up to 99:1 dr and up to 98% ee). To account for the observed (5*R*,1′*S*) absolute configuration of **636**, a transition state was proposed in which two nitrogen atoms from the picolinamide moiety of **C27** and two oxygen atoms coordinate copper(II) ion in a square plane manner, allowing the silyloxyfuran to attack the ketimine in the coordination sphere of the copper cation. The *Si* face of the dienolate approaches preferentially the *Re* face of the ketimine, thus avoiding steric repulsion of the diphenylphosphinoyl group.

**Scheme 164:**
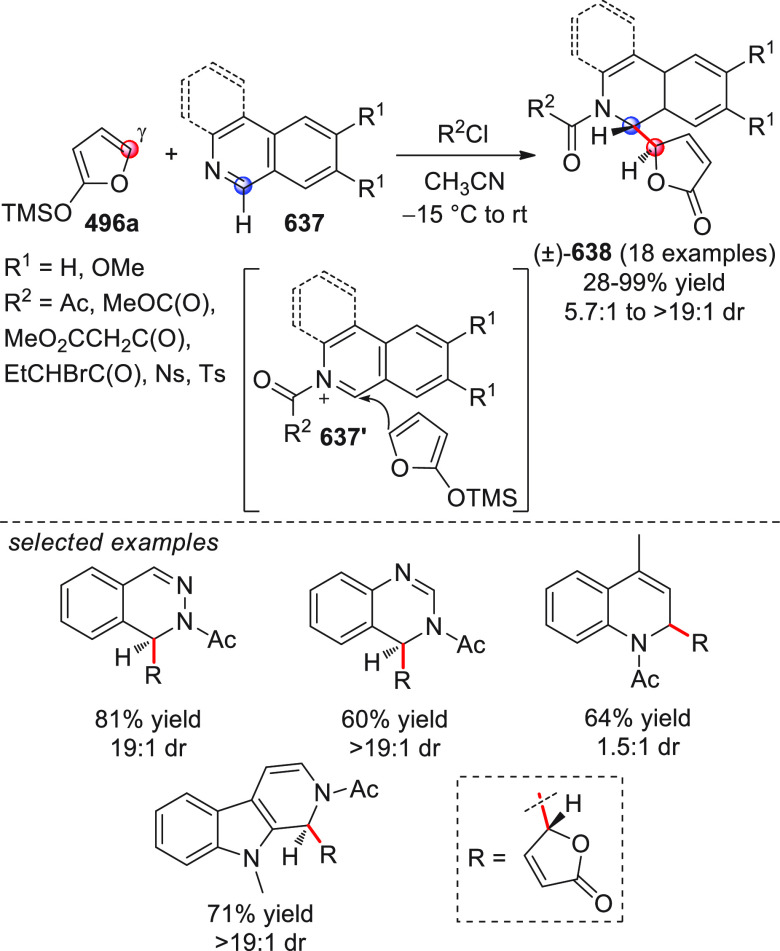


A highly versatile, diastereoselective, one-step, and three-component VMMnR involving TMSOF **496a** and a variety of nitrogen-containing heterocycles mainly of type **637** was described by Dodd and co-workers in 2014 (Scheme 164).^427,428^ Based on previous reports,^429,430^ the authors devised a highly diastereoselective procedure involving the use of acyl or sulfonyl chlorides as suitable activating agents of a plethora of different aza-heterocycles that, once acylated, generated a highly electrophilic iminium ion species of type **637′** that finally coupled to **496a** in a diastereoselective manner. The racemic reaction products were generally obtained in high yields (up to 99%) and mainly as single diastereoisomers. Several aza-heterocycles were successfully employed for this reaction, such as isoquinolines, quinoline, phenanthridine, quinazoline, phthalazine, and β-carboline, while electrophiles included acetyl chloride, methyl chloroformate, methyl chloromalonate, 2-bromobutanoyl chloride, and arylsulfonyl chlorides. Furthermore, a *N*-Boc silyloxypyrrole nucleophile was tested under the optimized reaction conditions, giving appreciable results (not shown).

**Scheme 165:**
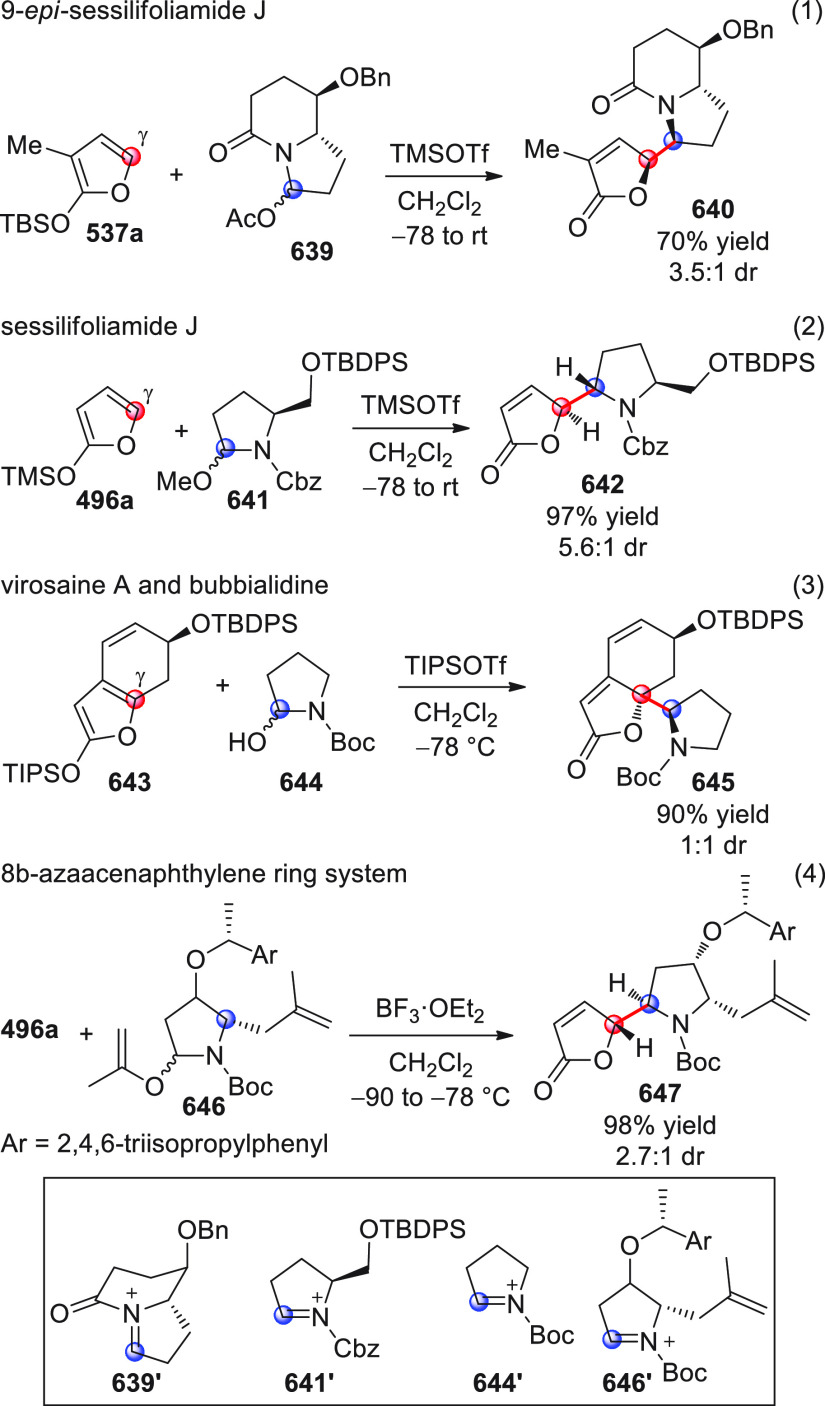


Huang and co-workers in 2011 applied a diastereoselective VMMnR between 2-methylsilyloxyfuran (**537a**) and chiral, bicyclic *N,O*-acetal **639** to access enantiopure tricyclic intermediate **640**, as a key precursor in the total synthesis of the stemona alkaloid 9-*epi*-sessilifoliamide J (Scheme 165, eq 1).^431^ Among the Lewis acids tested, TMSOTf (1 equiv) gave the best results, providing a 3.5:1 mixture of the *syn*-configured γ-adduct **640** as a major isomer in a good 70% yield. A plausible transition state was proposed, in which the in situ-formed iminium ion **639′** (Scheme 165, bottom) added to the nucleophile through a favorable Diels–Alder-like *Re–Re* approach (not shown).

Several years later, the same group used a VMMnR in the total synthesis of (−)-sessilifoliamide J (Scheme 165, eq 2).^432^ Here, starting from chiral Cbz-protected *N,O*-acetal **641**, the authors devised a two-component VMMnR with TMSOF (**496a**) as the vinylogous nucleophile. Thus, treatment of **496a** and **641** with stoichiometric TMSOTf, in CH_2_Cl_2_ at −78 °C afforded the desired γ-adduct **642** (via formation of the cyclic iminium ion **641′**) in a high 97% yield and a 5.6:1 dr in favor of the *syn*-configured isomer.

An interesting diastereoselective VMMnR in which the chiral information resides in the nucleophile was used by Gademann and co-workers, in their total synthesis of the securinega alkaloids virosaine A and the putatively related alkaloid bubbialidine (Scheme 165, eq 3).^433^ Following Busqué’s procedure,^434^ a vinylogous Mannich reaction between chiral, bicyclic dienolsilane **643** and *N,O*-acetal **644** was achieved using triisopropylsilyl triflate as a Lewis acid, generating a separable 1:1 mixture of two diastereoisomers in a combined 90% yield, from which the desired (*R,R*)-configured diastereoisomer **645** could be isolated.

More recently, Delair et al. used a BF_3_·Et_2_O-promoted, diastereoselective VMMnR between TMSOF **496a** and a readily accessed chiral pyrrolidine *N,O*-acetal **646** to access butanolide **647** en route to the uncommon [6.6.5]-tricyclic ring system of the 8b-azaacenaphthylenes alkaloids (Scheme 165, eq 4).^435^ In the event, a mixture of two lactone isomers was obtained in a high 98% combined yield and 2.7:1 dr in favor of the desired *syn*-isomer **647**.

**Scheme 166:**
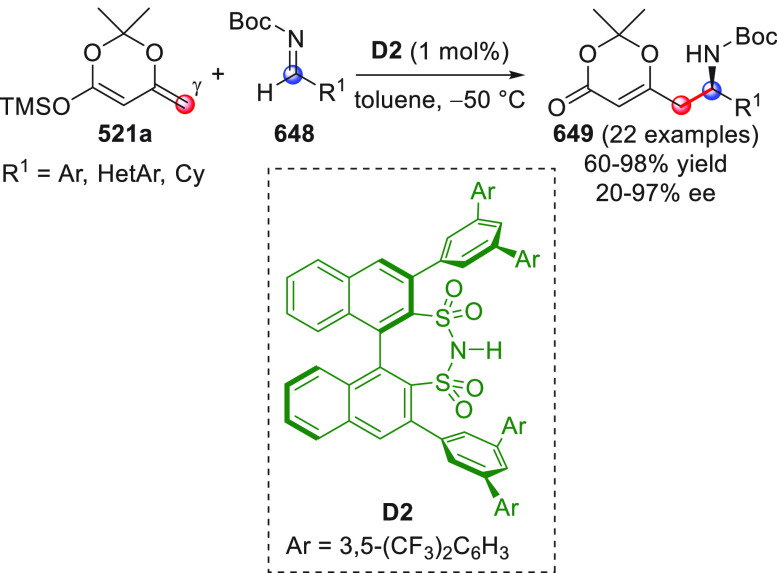


Finally, List and co-workers reported a rare, highly enantioselective approach to the synthesis of δ-amino-β-ketoesters by a disulfonimide-catalyzed VMMnR utilizing *N*-Boc aldimines **648** and dioxinone-derived silyloxydiene **521a** as reacting partners (Scheme 166).^436^ Of note, only 1 mol % of chiral disulfonimide precatalyst **D2** served as an efficient promoter for the VMMnR, carried out in toluene at −50 °C. Using readily available aromatic or heteroaromatic *N*-Boc-protected imines, a variety of polyfunctionalized γ-adducts **649** were accessed in high yields (up to 98%) and high enantioselectivities (up to 97%). Regrettably, aliphatic imines were quite inert, and only a cyclohexyl derivative could react under the optimized reaction conditions, yielding the corresponding adduct in 60% yield and 20% ee.

### 5.3 Conjugate Additions to Electron-Poor C=C Bonds

#### 5.3.1 Direct Procedures

##### 5.3.1.1 Acyclic Pronucleophiles

**Scheme 167:**
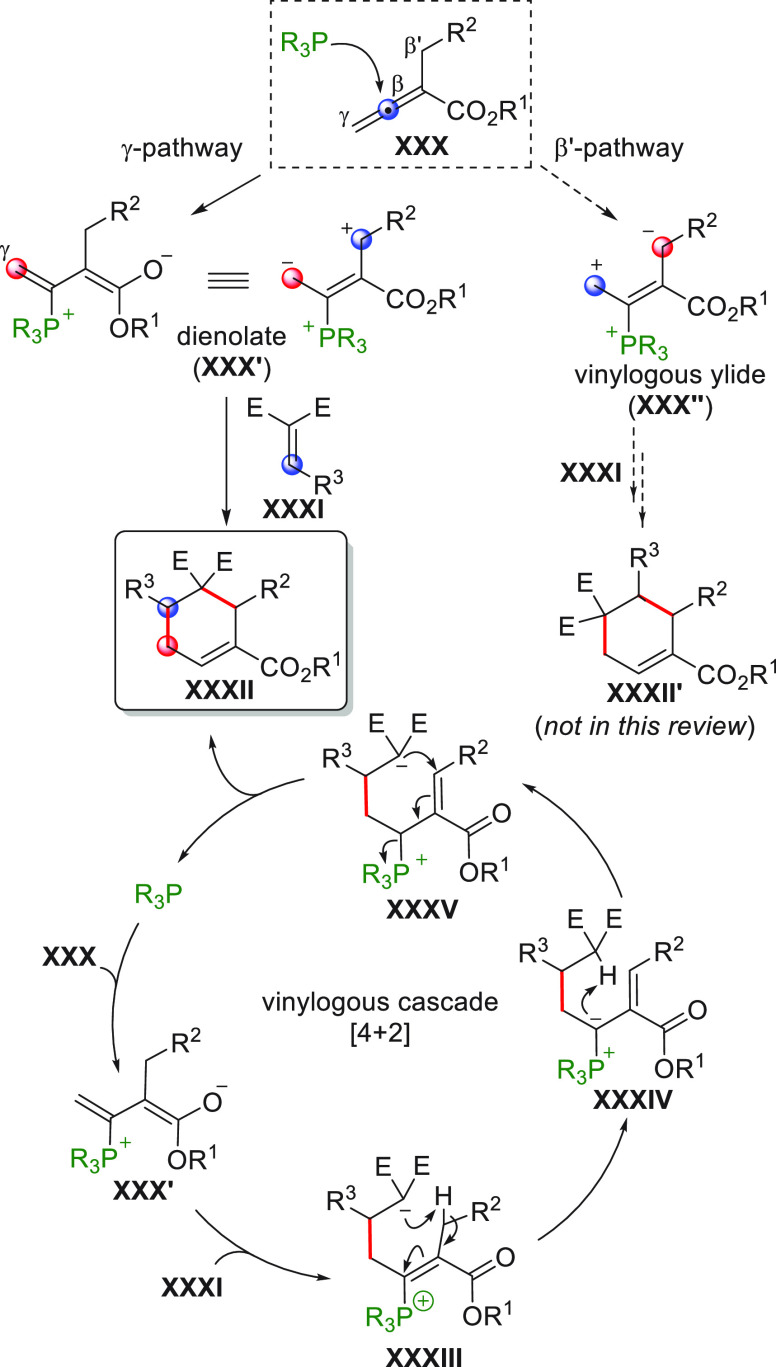


An intriguing, yet useful alternative to the classical base-catalyzed activation of α,β-unsaturated esters via dienolate-like intermediates is based on the nucleophilic addition of phosphines to the β sp-carbon of suitable allenoate species of type **XXX** (Scheme 167). Due to the unique reactivity of electron-deficient allenes, the incorporation of electrophiles such as electron-deficient C=C, C=N, and C=O bonds has been widely employed in phosphine catalysis to access polyfunctionalized carbo- and heterocycles via Diels–Alder-like reactions.^437^

Indeed, as described in Scheme 167, α-brached allenoates of type **XXX** can react with electron poor alkenes **XXXI** to form cyclohexenes **XXXII** (via formal [4 + 2] cycloaddition) under the guidance of an appropriate phosphine catalyst. Mechanistically, under suitable reaction conditions, the generation of a phosphonium dienolate **XXX′** by conjugate addition of the phosphine to the β-carbon of **XXX**, is followed by Michael addition to an activated olefin **XXXI** providing the zwitterionic intermediate **XXXIII**. A subsequent proton-transfer step facilitates tautomerization of the vinylphosphonium zwitterion **XXXIV** to an allylphosphonium zwitterion **XXXV**, which furnishes cyclohexene **XXXII** via intramolecular 6-*endo* cyclization followed by β-elimination of the phosphine (Scheme 167, γ-pathway). Alternatively, phosphonium dienolate **XXX′** can isomerize into the vinylogous ylide **XXX′′** (Scheme 167, β′-pathway), which adds to the olefin **XXXI** on its β′ carbon to provide the corresponding cyclohexene **XXXII′′** through a reaction sequence similar to the previously described catalytic cycle. Despite the profound interest raised by these and other phosphine-catalyzed transformations, as testified by the several reviews published on this issue,^438,439^ only the γ-pathway annulations (formal [4 + 2]), involving a dienolate-like intermediate, may be regarded as a vinylogous process, thus legitimately placing this type of transformations in this review. In this context, after the seminal works by Kwon’s group who disclosed achiral phosphine-catalyzed [4 + 2] annulation of allenoates with activated imines and alkenes for the expedient construction of polyfunctionalized cyclohexenes and other heterocycles,^440−442^ the asymmetric phosphine-catalyzed [4 + 2] annulations of activated alkenes with allenoates evolved more slowly until recently, when several important examples have been reported.

**Scheme 168:**
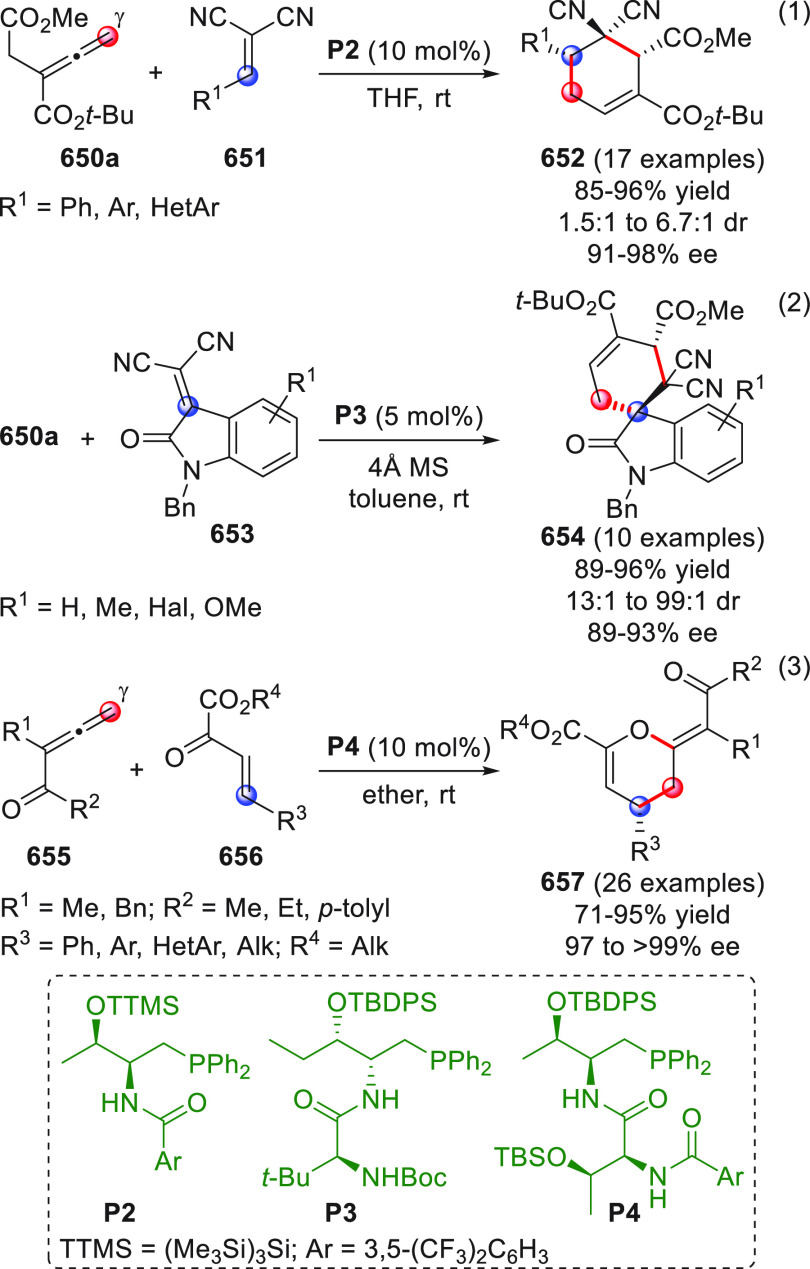


In 2012, Lu and co-workers were the first to develop a highly enantioselective [4 + 2] annulation between allenoate **650a** and a series of activated alkenes catalyzed by chiral amino acid-derived phosphines (Scheme 168, eqs 1 and 2).^443^ Their survey started by examining the catalytic effects of various amino acid-derived phosphines in the [4 + 2] annulation between allenoate **650a** and several arylidenemalononitriles **651** (eq 1). *O*-Tris(trimethylsilyl)silyl (TTMSS) l-threonine-based phosphine–amide **P2** was elected as more suitable catalyst, yielding the corresponding *cis*-configured cyclohexenes **652** in good overall yields (85–96%), moderate to good diastereoselectivity, and excellent enantioselectivity (up to 6.7:1 dr and 91–98% ee). The reaction was applied to a wide range of activated phenyl-, aryl-, and heteroarylidene malononitriles, while challenging alkenes derived from aliphatic aldehydes were found to be unsuitable for the annulation.

To further expand the scope of this asymmetric [4 + 2] annulation reaction, the authors switched to the more challenging annulation between allenoate **650a** and isatin-derived ylidene malononitriles **653** to access chiral, enantiopure 3-spirocyclohexene-2-oxindoles **654**, that are striking molecular frames of pharmaceutical interest (Scheme 168, eq 2). To this end, it was found that *O*-protected d-Thr-L-*tert*-Leu-derived phosphine **P3** effectively catalyzed the formation of a number of 3-spirocyclohexene-2-oxindoles **654** in very high yields (89–96%), and with excellent diastereo- and enantioselectivities (up to 99:1 dr and 89–93% ee). The methyl ester group at the β′ position of allenoate **650a** could also be replaced by other ester moieties or electron-poor aromatic rings, allowing the annulation to proceed smoothly to afford cyclization products in moderate yields, good dr and excellent ee (not shown).

More recently, the same group devised a phosphine-catalyzed [4 + 2] annulation process employing allenones of type **655** and β,γ-unsaturated α-keto esters **656** (Scheme 168, eq 3).^444^ By utilizing 10 mol % of the dipeptide-based bifunctional phosphine **P4**, almost optically pure 3,4-dihydropyrans **657** were obtained in high yields (71–95%). The reaction was applied to a wide range of β,γ-unsaturated α-keto esters bearing different aromatic groups, regardless of the steric and electronic properties of the substituents on the aromatic ring. Of note, different vinyl- and linear/branched alkyl-substituted α-keto esters were also tested, providing the desired products in good yields and nearly perfect enantioselectivities.

**Scheme 169:**
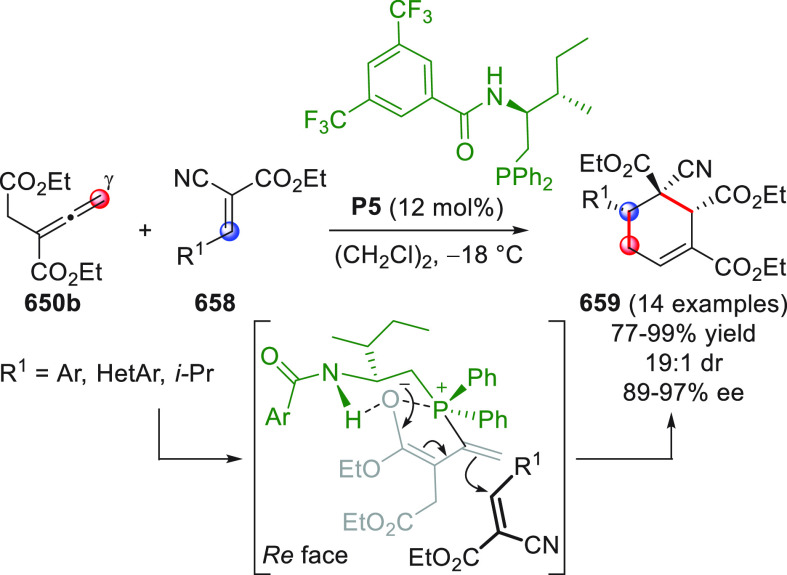


In the same year of Lu’s first work (2012), Zhao and co-workers reported the organocatalytic, enantioselective formal [4 + 2] cycloaddition between α-substituted dienoate **650b** and β-substituted (*E*)-2-cyano acrylates **658** using chiral bifunctional *N*-acyl aminophosphine catalyst **P5** (Scheme 169).^445^ γ-Regioselective access to polyfunctionalized cyclohexenes **659** was secured, bearing three contiguous stereocenters in high yield and excellent enantioselectivity.

Olefins **658** with electron-rich or electron-poor aryl substituents were well tolerated, while *ortho*-substituted derivatives, probably due to steric reasons, needed more elevated temperature (room temperature) to give satisfactory yields, albeit with a slight drop in the enantioselectivity. Interestingly, the challenging aliphatic isobutyraldehyde-derivative acrylate congener (R^1^ = isopropyl) was successfully coupled with **650b** affording the corresponding isopropyl cyclohexene product in 92% yield and 97% ee. A tentative mechanism was also proposed to explain the stereochemical output observed. A cyclic six-membered transition state was considered responsible for the enantioselectivity of the first, vinylogous addition step favoring the *Re* face attack on the activated olefins. The newly formed stereocenter would then induce the chirality of the second one formed in the final cyclization step (not shown).

**Scheme 170:**
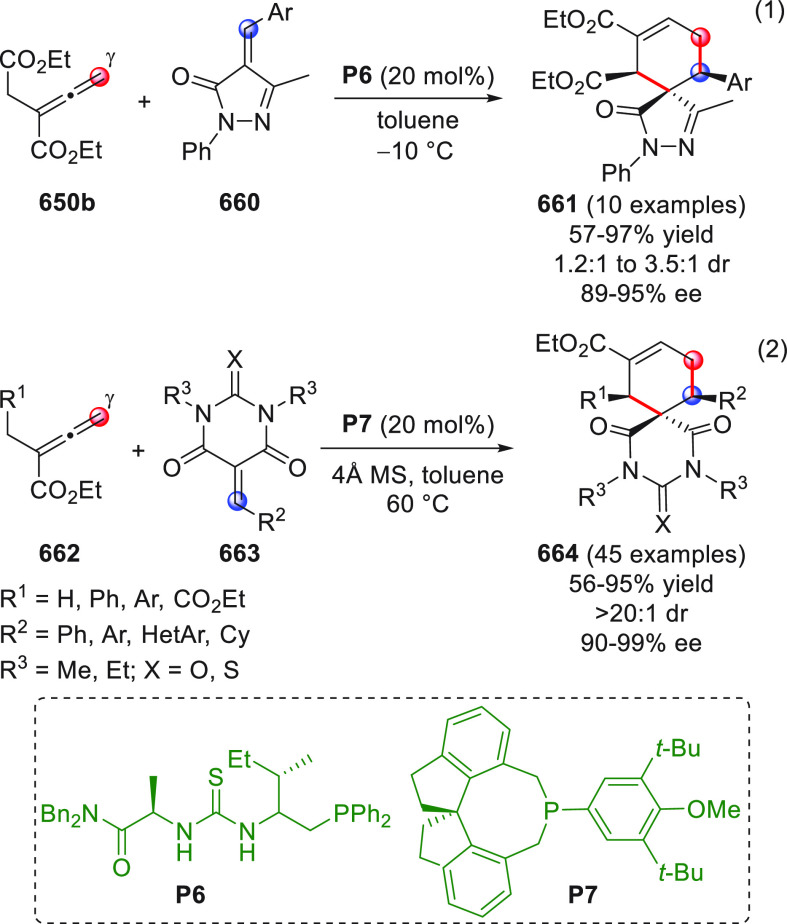


More recently, Guo et al. reported a γ-regioselective phosphine-catalyzed formal [4 + 2] cycloaddition reaction of allenoate **650b** with unsaturated pyrazolones **660** under mild reaction conditions to afford challenging polyfunctionalized spiropyrazolone derivatives **661** in both racemic and enantioselective formats (Scheme 170, eq 1).^446^ Initially, the authors examined the reaction conditions by surveying a series of achiral phosphines as catalysts (not shown) and found that moderately nucleophilic MePPh_2_ (20 mol %) efficiently catalyzed the reaction between diethyl allenoate and differently substituted pyrazolones **660** in toluene at room temperature, providing the corresponding products (±)-**661** as single 1′,5′-*syn* diastereoisomers in moderate to very high yields (49–99%). Switching to the asymmetric version of the reaction (Scheme 170, eq 1), several chiral phosphine derivatives were initially screened to catalyze the [4 + 2] annulation between **650b** and **660**, appointing thiourea-based bifunctional phosphine **P6** as the most suited catalyst for the reaction. In fact, under the optimized reaction conditions, with **P6** (20 mol %), an asymmetric variant of this [4 + 2] cycloaddition reaction was achieved, giving a set of polyfunctionalized (1′*S*,5′*S*)-configured chiral spiropyrazolone derivatives **661** in moderate to excellent yields with moderate to excellent diastereoselectivities and excellent enantioselectivities (89–95% ee). Again, a wide substitution pattern within the arylidene substituent of **660** was tolerated, with the sole exception of 3-Br and 4-Cl derivatives, which were less reactive. Interestingly, the minor 1′,5′-*syn*-configured diastereomers were nearly racemic (not shown).

The same group developed an enantioselective [4 + 2] annulation of barbiturate-derived alkenes **663** with allenoates **662** to access pharmaceutically relevant spirobarbiturates **664** as single 1′,5′-*syn* configured isomers, using a spirocyclic chiral phosphine **P7** as the catalyst (Scheme 170, eq 2).^447^ Of note, a wide range of α-substituted allenoates bearing electron-rich, -neutral, and -deficient aromatic moieties and a series of aromatic barbiturate- and thiobarbiturate-derived alkenes (irrespective of their electronic properties) were tolerated, yielding various spirobarbiturate-cyclohexenes in good to excellent yields (56–95%), with excellent diastereo- and enantioselectivities (>20:1 dr, 90–99% ee). Interestingly, both the α-methyl-substituted allenoate and the cyclohexylydene aliphatic barbiturate derivative proved to be compatible substrates for this transformation.

##### 5.3.1.2 Cyclic Pronucleophiles

**Scheme 171:**
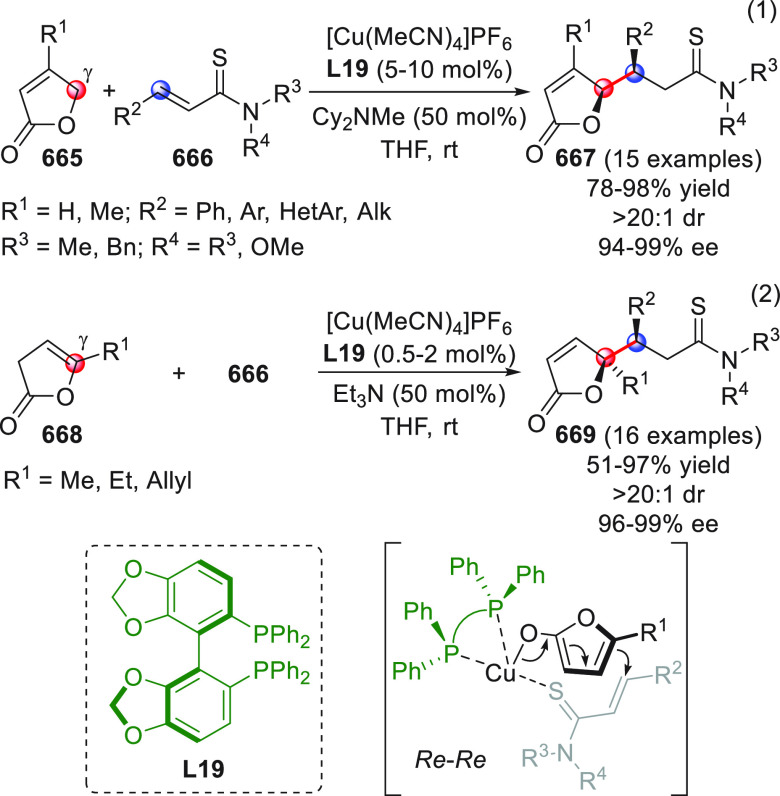


In 2014 Kumagai, Shibasaki, et al. applied the soft Lewis acid/Brønsted base cooperative catalysis to enable the direct, catalytic, asymmetric vinylogous conjugate addition of α,β- and β,γ-unsaturated butyrolactones **665** and **668** to α,β-unsaturated thioamides **666** (Scheme 171, eqs 1 and 2).^448^

The reaction afforded the corresponding γ-homologated butenolides **667** and **669** bearing two consecutive tri- and tetrasubstituted stereogenic centers in a highly diastereo- and enantioselective fashion. In particular, the cooperative action of a soft Lewis acid such as chiral copper complex [Cu(CH_3_CN)_4_]PF_6_/(*R*)-Segphos (**L19**) and a Brønsted base such as Cy_2_NMe was the most suited catalytic system to promote the vinylogous Michael reaction (VMcR) between pronucleophilic γ-crotonolactones **665** and unsaturated thioamides **666** (eq 1).

The reaction of β-aryl- or β-heteroaryl-substituted α,β-unsaturated thioamides proceeded smoothly with a 5 mol % catalyst loading, regardless of the electronic nature of the substituents, leading to the corresponding γ-adducts **667** with high yields (78–98%) and almost complete diastereo- and enantioselectivity. Of note, even β-alkyl-substituted α,β-unsaturated thioamides were effective substrates, although 10 mol % of catalyst was required to complete the reaction. Interestingly, the same reaction was tested on other electron-deficient olefins such as unsaturated esters, amides, ketones, nitriles, imides, tosylated amides, thioesters, α-vinylidene malonates, and *N*-acylpyrrole derivatives, but all failed to give the desired product in considerable yield and stereoselectivity. A very similar reaction pattern was observed when α-angelicalactone-type pronucleophile **668** was used instead of **665** (Scheme 171, eq 2). In this case, the more common Et_3_N was used to complete the reaction with a 0.5–2 mol % catalyst loading. To account for the observed selectivity, a plausible catalytic cycle was proposed, envisaging the in situ formation of a vinylogous copper dienolate by the action of the tertiary amine and the weak assistance of the Cu/(*R*)-Segphos complex. Subsequent coordination of the thioamide **666** to the tetracoordinate copper center would produce the active transition state (Scheme 171, bottom), in which a favorable *Re–Re* substrate interaction takes place to generate the observed (5*R*,1′*S*)-configured products.

The above-described work is an example of how cooperative catalysis is emerging as a valuable tool in asymmetric synthesis, fueled by the identification of more efficient catalytic systems with even increasing substrate scopes. Many endeavors have been devoted during the past few years toward developing the right catalyst pairs giving rise to powerful bifunctional systems, and many types of organic catalysts have been used in combination with appropriate metals to achieve unprecedented enantioselective processes.

**Scheme 172:**
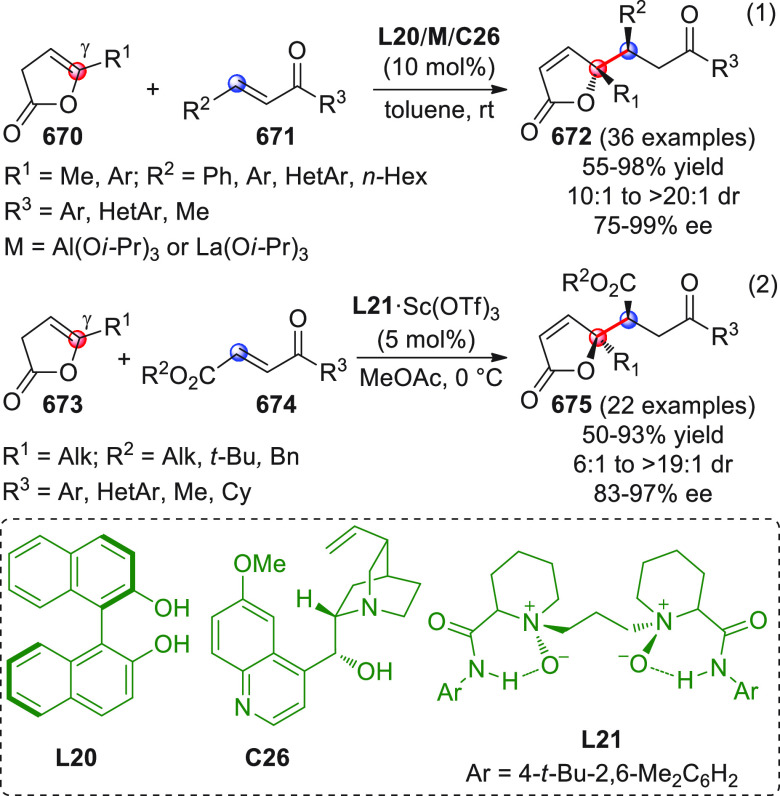


In this context, a nice example was reported by Wang’s group who devised a couple of cooperative catalytic systems that enabled the efficient, direct, asymmetric vinylogous Michael reaction of γ-aryl- and γ-alkyl-substituted deconjugated butenolides **670** to a series of aromatic and aliphatic enones **671** giving access to the corresponding chiral γ,γ-disubstituted butenolides **672** in good yields with high diastereo- and enantioselectivities (Scheme 172, eq 1).^449^ Indeed, after an initial survey to assess the viability of the reaction and the best Brønsted base/Lewis acid catalytic system, it was found that ternary (*R*)-BINOL **L20**/Al(O*i*-Pr)_3_/quinine **C26** and **L20**/La(O*i*-Pr)_3_/**C26** systems were the best choice, giving γ-adducts **672** in high yields and enantioselectivities (75–99% ee) as *anti*-configured isomers.

During the same year, a highly efficient *N,N*′-dioxide **L21·**Sc(OTf)_3_ complex has been developed by Feng and co-workers for the asymmetric VMcR of pronucleophilic γ-substituted-β,γ-unsaturated butenolides **673** to α,β-unsaturated γ-ketoesters **674**, affording the corresponding γ,γ-disubstituted butenolide products **675** in moderate to good yields (up to 93%) with high dr (up to >19:1) and ee values (up to 97%) under mild reaction conditions (Scheme 172, eq 2).^450^ Under the optimal reaction conditions (5 mol % catalyst complex, in MeOAc at 0 °C), the substrate scope of the reaction was investigated. The ester group within acceptor **674** exhibited an influence on both diastereo- and enantioselectivity, with the bulkier *tert*-butyl group (R^2^ = *t*-Bu) giving the best results. The stereocontrol of the reaction was sensitive to neither the electronic property nor the steric hindrance of aromatic and aliphatic substituents (R^3^) on **674**; however, *m*-substituted congeners generally gave lower yields than the parent *o*- and *p*-substituted derivatives. For what concerns the substituent within lactone **673**, switching from Me to Et and *n*-C_10_H_21_, the diastereo- and enantioselectivity of the reaction increased but with lower efficiency. Unexpectedly, the reaction failed with the phenyl-substituted *β,γ*-unsaturated butanolide.

**Scheme 173:**
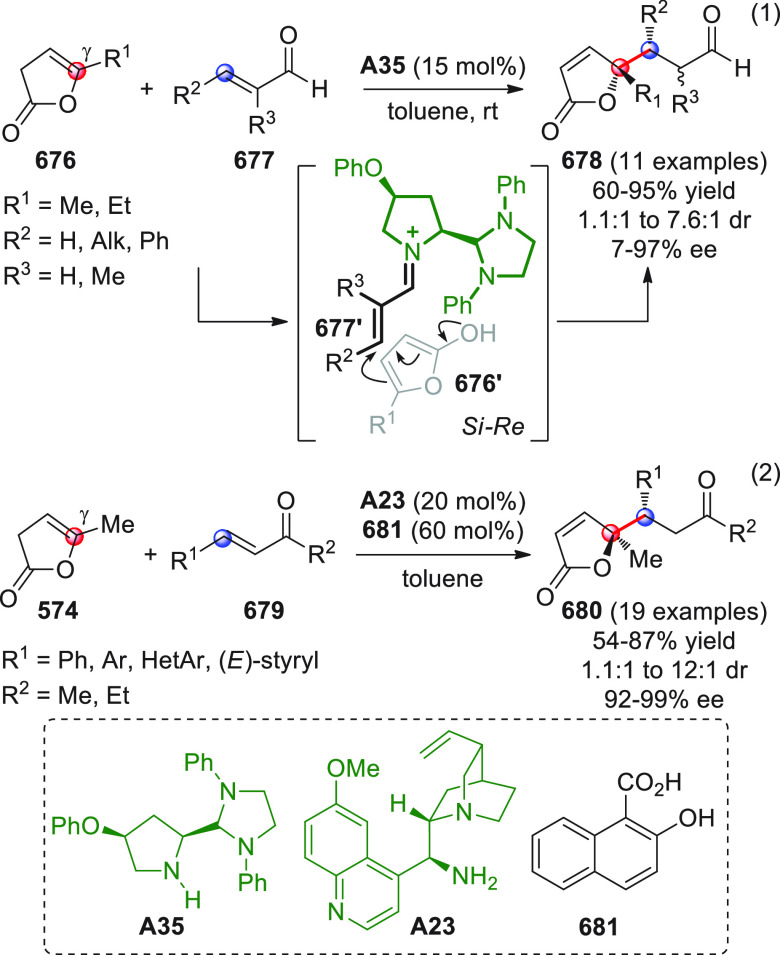


Switching to completely metal-free organocatalytic processes, the first direct, catalytic, enantioselective VMcR involving pronuclephilic deconjugated butenolides **676** was reported by the Alexakis’s group in 2011 (Scheme 173, eq 1).^451,452^ Indeed, under the optimized reaction conditions, Aminal-PYrrolidine (APY) catalyst **A35** (15 mol %) was able to promote the VMcR between γ-methyl and γ-ethyl furanones with varied enals **677** in toluene at room temperature, affording a diastereomeric mixture of the corresponding γ-homologated butenolides **678** in good to high yields (60–95%) and very good enantioselectivity (up to 97% ee). A variety of substitution patterns at the enal β-position could be tolerated, so that both alkyl and phenyl substituents worked well under the optimized reaction conditions.

A sensible drop in diastereo- and enantioselectivity was observed when α-branched derivatives such as methacrolein were used as electrophilic components. Interestingly, preliminary mechanistic investigations allowed the authors to propose a mechanism in which the catalyst has the sole role of activating the electrophile via formation of the expected iminium ion **677′**, while a thermodynamic enol equilibrium involving deconjugated butenolide **676** provides small quantities of dienol **676′** that undergoes a fast addition on the favored prostereogenic face of the electrophile not hindered by the aminal group of the catalyst (Scheme 173). More recently, the same group reported the direct, VMcR of unactivated α-angelica lactone **574** to enones **679** under iminium ion activation using the combination of chiral 9-amino-9-deoxy-*epi*-quinine **A23** along with 2-hydroxy-1-naphthoic acid (**681**) as catalytic system (Scheme 173, eq 2).^453^ The reaction led to the formation of optically pure γ,γ-disubstituted butenolides **680** in high yields (up to 87%) and moderate-to-good *anti* diastereoselectivities (up to 12:1 dr).

**Scheme 174:**
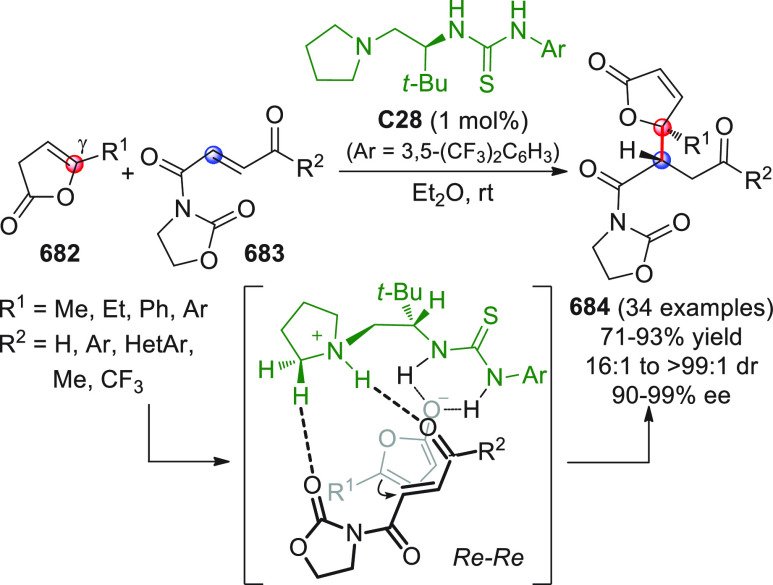


An alternative approach based on noncovalent organocatalytic activation was explored by Huang, Tan, Jiang, et al. in 2012, in the effort to synthesize chiral, enantiopure γ,γ-disubstituted butenolides from easily accessible deconjugated butenolides **682** (Scheme 174).^454^ Following previous achievements from the same group about the peculiar ability of the 2-oxazolidinone amide moiety to interact with suitable groups such as a thiourea via H-bond,^455^ the authors have successively developed the direct, asymmetric VMcR of γ-aryl- and alkyl-substituted butenolides with suitable electrophiles such as (*E*)-4-oxo-4-arylbutenamides, (*E*)-oxazolidinone enoates, and (*E*) or (*Z*)-β-trifluoromethyl oxazolidinone enoate of type **683**. Using L-*tert*-leucine-derived chiral bifunctional pyrrolidine-thiourea **C28** (1 mol %) as the catalyst, various γ,γ-substituted butenolides **684** were obtained in good to excellent yields, with excellent enantio- and diastereoselectivities (up to 93% yield, 99% ee, and dr >99:1). According to experimental observations and DFT calculations, a suitable transition state was proposed (Scheme 174) in which the catalyst interacts through H-bond interactions with the electrophilic enamide in a bidentate manner. DFT calculations unveiled a considerable interaction between the oxazolidinone carbonyl and the α-H of the pendent pyrrolium moiety of the catalyst through a nonclassical C–H···O hydrogen bonding.

**Scheme 175:**
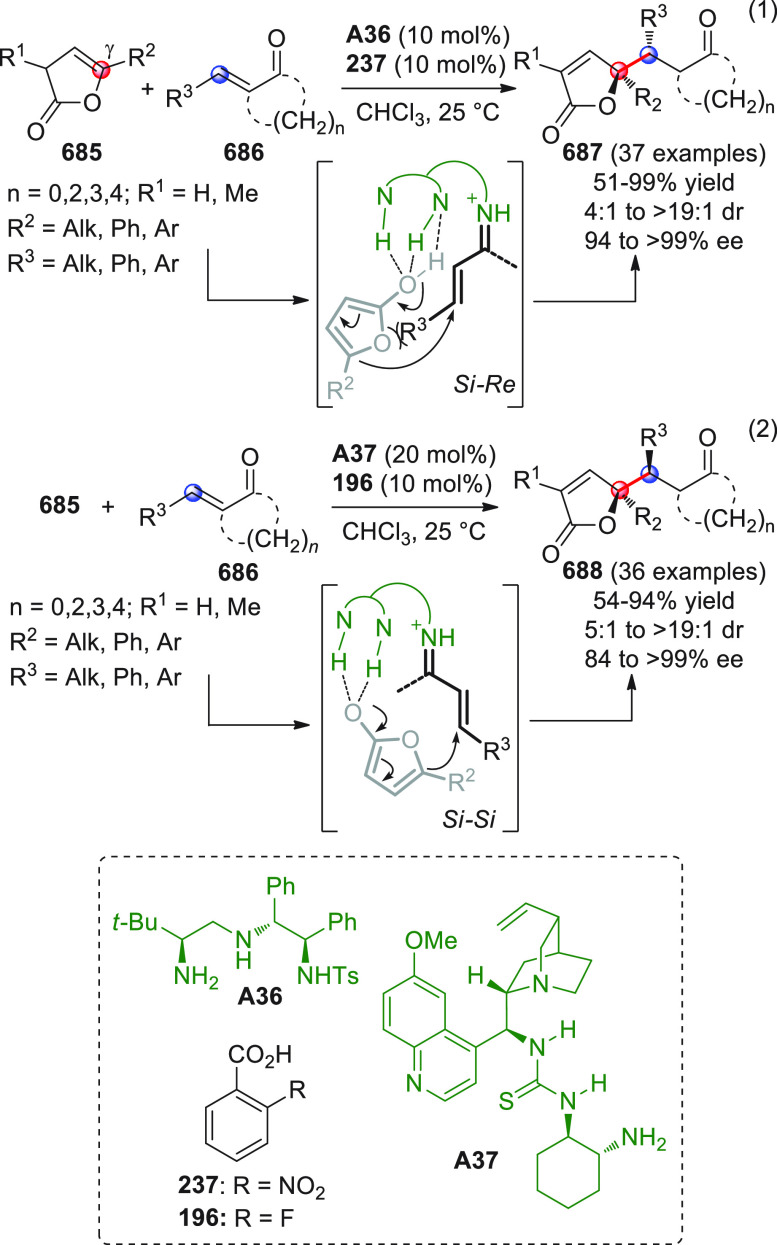


An important contribution that merges the covalent and noncovalent activation modes for the stereoselective synthesis of chiral, enantiopure γ,γ-disubstituted butenolides from prochiral precursors was reported by Dixon and co-workers in 2014 (Scheme 175).^456^ In particular, a highly diastereodivergent, asymmetric, and direct VMcR between β,γ-unsaturated butenolides **685** and α,β-unsaturated ketones **686** was devised, by employing stereochemically complementary yet nonenantiomeric primary amine catalytic systems. After an extensive optimization survey, it was found that catalyst **A36**, featuring a primary amine group, a vicinal secondary amine, and a sulphonamide moiety as a terminal hydrogen bond donor, perfectly catalyzed the reaction between α-angelica lactone derivatives **685** and enones **686** in the presence of *o*-nitrobenzoic acid **237** (20 mol %), to give almost enantiopure adducts **687** in high yields and high diastereoselectivity in favor of the *anti*-configured isomers (Scheme 175, eq 1).

Conversely, using bifunctional primary amine-thiourea catalyst **A37**, in combination with catalytic quantities of *o*-fluorobezoic acid (**196**) allowed the access to the corresponding *syn*-configured derivatives **688** with comparable results (Scheme 175, eq 2). A broad range of γ-substituted butenolides and functionalized enones, bearing both alkyl substituents and electron-rich/deficient aryl substituents, worked well under the optimized reaction conditions. Furthermore, cyclic enones were also tested with 2-cyclopentenone giving a relatively poor diastereoselectivity, as compared with 2-cyclohexenone or 2-cycloheptenone.

The observed catalyst-dependent diastereodivergence was ascribed to the different nature of polyamine catalysts **A36** and **A37** used to promote the stereoselective VMcR. In this context, the *anti* selective VMcR was envisaged to be the result of a favorable transition state in which a tight H-bonding interaction would preferentially control the position and orientation of the dienolate, overcoming the inherent steric hindrance of the reactants. Conversely, a weak and loose H-bonding interaction involving quinine thiourea catalyst **A37** would allow a transition state in which the steric bias dominates the stereoselectivity, thus favoring *syn* selectivity.

Among the large variety of asymmetric Michael reactions developed so far, nitroalkenes have been one of the most widely used Michael acceptors, since they can undergo facile β-alkylation reactions and the corresponding Michael products may be interconverted to important organic functional groups. In this context, the direct, enantioselective Michael-type addition of α-angelica lactone pronucleophiles to nitroalkenes promoted by bifunctional, noncovalent organocatalysts, which provided useful γ-homologated butenolides with at least two contiguous stereocenters, has been widely explored in the past decade by several groups as described in Table 7. The first example of a direct, enantioselective VMcR involving γ-substituted deconjugated butenolides and nitroolefins was reported by Mukherjee and co-workers in 2012 (Table 7, entry 1),^457,458^ which unveiled the ability of a new quinine-derived bifunctional tri*iso*-butyl catalyst **C29** to promote the enanatioselective synthesis of densely functionalized, *syn*-configured butenolides with contiguous quaternary and tertiary stereocenters in excellent yield and high enantioselectivity (up to 98% ee) with perfect diastereoselectivity (>20:1 dr).

**Table 7:**
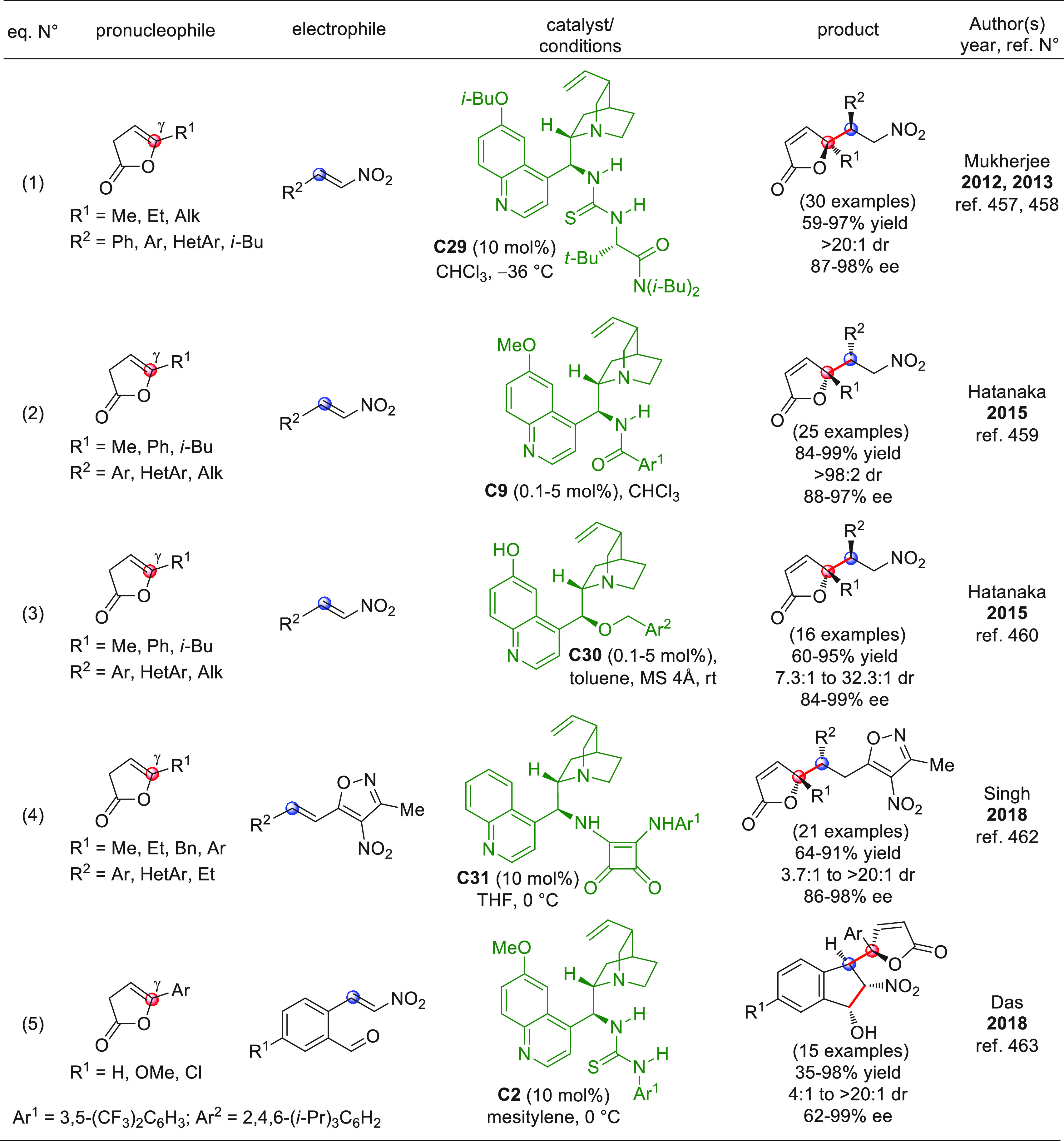
Direct, Enantioselective VMcR of α-Angelica Lactone Pronucleophiles to Nitroalkenes, Catalyzed by Bifunctional Organocatalysts

Some years later, Hatanaka’s group developed a novel bifunctional *epi*-quinine-derived 3,5-bis(CF_3_)benzamide catalyst **C9** which showed a high catalytic activity (0.1–5 mol % loadings) in the asymmetric nitro-Michael addition reaction of a series of deconjugated lactones to a wide range of aromatic and aliphatic nitroalkenes (entry 2).^459^ The corresponding *syn*-configured adducts were obtained in high yields (up to 99%) with excellent diastereo- and enantioselectivities (>98:2 dr and up to 98% ee).

Capitalizing on these results, the same group turned attention toward the more challenging *anti*-selective nitro-Michael reaction (Table 7, entry 3).^460,461^ Remarkably, the requested catalyst-controlled switching of diastereoselectivity was achieved with the use of bulky 6′-OH-*epi*-quinine catalyst **C30**, that, mirroring the high catalytic activity of **C9** in the previous reaction, promoted the addition between diverse sets of deconjugated lactones and nitroolefins, to access *anti*-configured Michael adducts in good yields (up to 95%) with excellent diastereo- and enantioselectivities (up to 32:1 dr and up to 99% ee).

More recently, highly unsaturated nitro-derivatives bearing a conjugated 4-nitro-5-vinyl isoxazole moiety were used by Singh and co-workers as electrophilic substrates for the enantioselective, vinylogous 1,6-conjugate addition between β,γ-unsaturated butanolide pronucleophiles giving access to a broad range of densely functionalized enantioenriched γ,γ-disubstituted butenolides (Table 7, entry 4).^462^ After a brief optimization survey, chiral bifunctional *epi*-quinine squaramide **C31** was the best catalyst to provide *syn*-configured adducts in high yields (up to 91%), with excellent enantio- (up to 98% ee) and diastereoselectivities (up to >20:1).

An interesting cascade approach was recently developed by Das et al. to build polyfunctionalized, enantiopure (nitro)indanol scaffolds using a VMcR between γ-substituted deconjugated butenolides and *o*-formyl-β-nitro-styrenes using bifunctional *epi*-quinine-thiourea catalyst **C2** (entry 5).^463^ The reaction, carried out in mesitylene at 0 °C, afforded the corresponding indanol products, which contain four contiguous stereocenters in good yields (up to 98%), in good diastereoselectivities (up to >20:1 dr), and with moderate to high enantioselectivities (up to 99% ee).

The high versatility of β,γ-unsaturated butenolides as γ-selective pronucleophilic species was further demonstrated by the development of a plethora of asymmetric vinylogous Michael-type addition reactions on diverse α,β-unsaturated carbonyl and sulfonyl acceptors other than nitroalkenes (Table 8). For example, in 2012 Mukherjee and Manna reported a highly diastereo- and enantioselective protocol for the direct VMcR of deconjugated alkyl-substituted butenolides to symmetrical *N*-arylmaleimides using the adamantane tertiary-amine/thiourea-based bifunctional catalyst **C32** (5 mol %, Table 8, entry 1).^464^ The reaction afforded a varied set of enantioenriched succinimide derivatives in high yields (up to 99%) and diastereoselectivity (up to 18:1 dr). The scope of this reaction was further expanded on aryl-substituted butenolide congeners by Xu, Wang, et al. using only 1 mol % of a bifunctional squaramides catalyst (not shown), providing the corresponding succinimide derivatives in comparable yields and stereoselectivity, albeit with lower diastereocontrol (up to 4.5:1).^465,466^

**Table 8:**
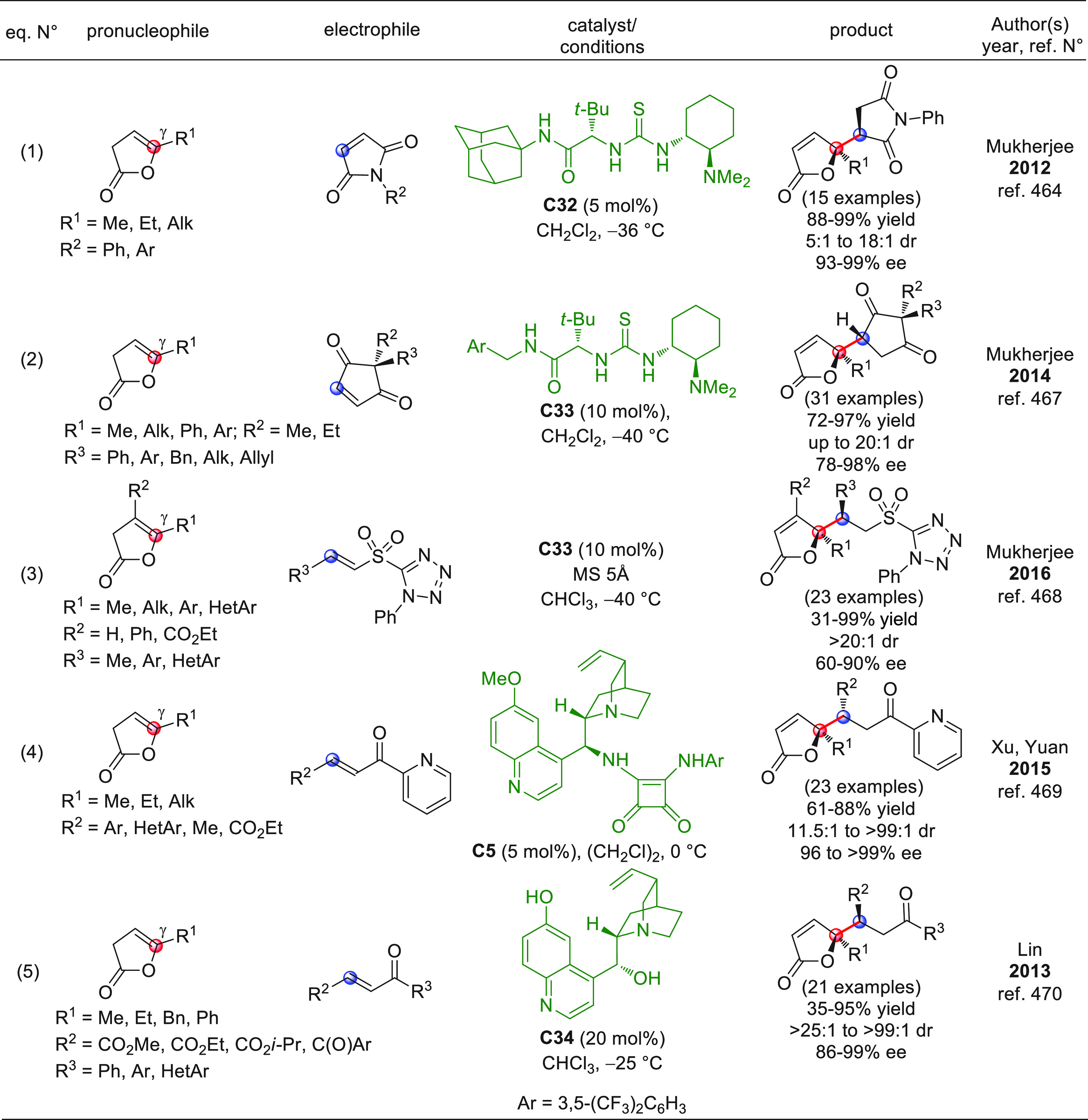
Direct, Enantioselective VMcR of α-Angelica Lactone Pronucleophiles to Differently Functionalized Michael Acceptors, Catalyzed by Bifunctional Organocatalysts

Also, Mukherjee and Manna presented an enantioselective, catalytic desymmetrization of 2,2-disubstituted cyclopentene-1,3-diones through the direct VMcR of deconjugated butenolides (Table 8, entry 2).^467^ Triggered by chiral, tertiary amino thiourea **C33** (10 mol %), derived from the “*matched*” combination of (*S*)-*tert*-leucine and (1*R*,2*R*)-diaminocyclohexane, the reaction afforded ciclopentadione products featuring two quaternary and one tertiary stereocenter in high yields (up to 97%) and diastereoselectivity (>20:1).

More recently, Mukherjee et al. exploited the same catalyst **C33** to promote a formal γ-allylation reaction of deconjugated butenolides based on a two-step sequence consisting of a catalytic diastereo- and enantioselective VMcR to vinyl sulfones followed by Julia–Kocienski olefination (Table 8, entry 3).^468^ This highly modular approach delivered densely functionalized butenolides containing a quaternary stereogenic center in excellent yields with good to high enantioselectivites.

Another interesting class of Michael acceptors used to functionalize α-angelica lactone derivatives through vinylogous transformations is represented by 2-enoylpyridines, bearing an α,β-unsaturated carbonyl moiety flanked by a pyridine core (Table 8, entry 4).^469^ In this context Xu, Yuan, et al. reported an asymmetric, *anti*-selective direct VMcR of alkyl-substituted deconjugated butenolides to 2-enoylpyridines catalyzed by the *epi*-quinine squaramide **C5**, to afford a number of γ,γ-disubstituted butenolide derivatives bearing two consecutive tri- and tetrasubstituted stereogenic centers, in acceptable yields (up to 88%) with excellent stereoselectivities (up to >99:1 dr and up to >99% ee).

As depicted in entry 5, 3-aroyl acrylates and 1,2-diaroylethylenes have been successfully used by Lin and co-workers,^470^ as Michael acceptors in a *syn*-selective organocatalytic, enantioselective, direct VMcR of deconjugated butenolides, to provide the corresponding γ,γ-dialkylated butenolides in high yields and almost complete distereo- and enantiocontrol. The reaction, promoted by bifunctional 6′-OH quinine derivative **C34** (20 mol %), was quite general, since both alkyl- and aryl-substituted nucleophiles, as well as electron-rich or electron-deficient electrophiles, were compatible substrates under the optimized reaction conditions.

As Tables 7 and 8 demonstrate, β,γ-unsaturated furanones represent highly versatile pronucleophilic scaffolds that enable access to valuable γ,γ-disubstituted butenolides in a stereocontrolled manner.

**Scheme 176:**
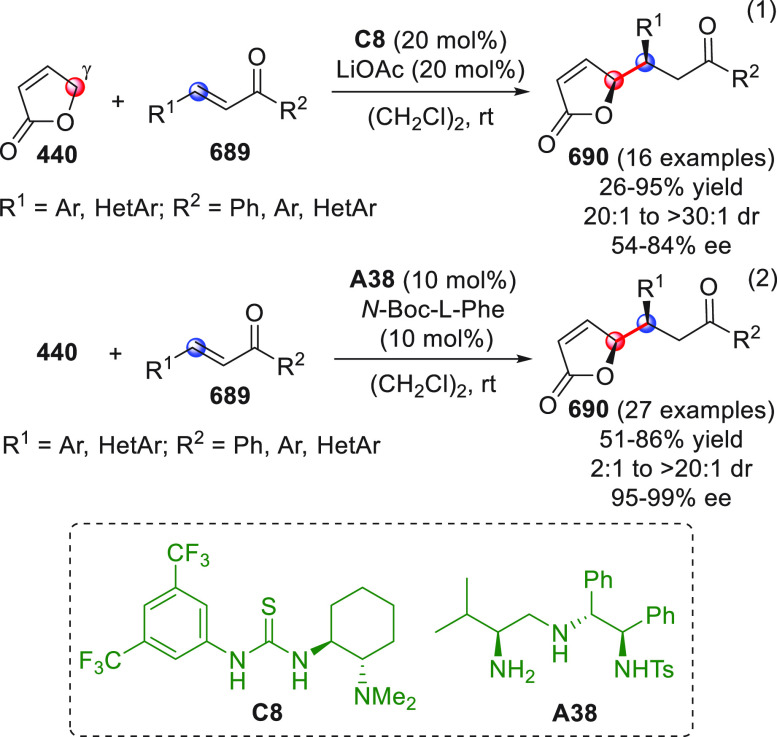


Quite surprisingly, the use of conjugated α,β-unsaturated furan(5*H*)ones as pronucleophiles in direct, catalytic, vinylogous Michael-type transformations has been much less explored. One of such few examples was published by Ji, Wang, et al. in 2010, reporting an amine-thiourea promoted direct Michael addition of γ-butenolides to chalcones to access chiral, enantioenriched γ-alkylated butenolides (Scheme 176, eq 1).^471^ Indeed, under optimized reaction conditions, Takemoto’s amine-thiourea bifunctional catalyst **C8** (20 mol %) was able to promote the VMcR between **440** and a series of aromatic and heteroaromatic chalcones **689** with the aid of LiOAc as a basic additive, to provide the vinylogous, *syn* configured adducts **690** in good to excellent diastereoselectivity and moderate to good enantioselectivities. Almost contemporarily, Liang, Ye, et al. reported a very similar transformation involving the direct, organocatalytic, asymmetric VMcR of **440** with **689** with a l-valine-derived multifunctional primary amine salt **A38** as the catalyst (Scheme 176, eq 2).^472^ The reaction, in the presence of *N*-Boc-l-phenylalanine (10 mol %) as acidic additive, resulted in the formation of the corresponding ketobutenolides **690** with satisfactory yields and high diastereo- and enantioselectivities (up to 20:1 dr and 95–99% ee). Of note, differently from the previous methodology, several aliphatic ketones proved suitable substrates for this direct VMcR.

**Scheme 177:**
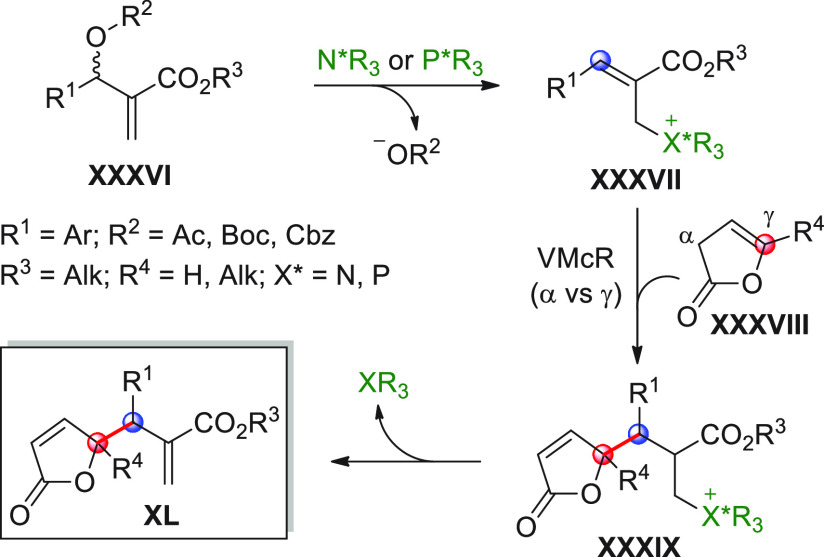


The allylic alkylation with Morita–Baylis–Hillman (MBH) adducts of type **XXXVI** (Scheme 177) through the catalysis of metal-free chiral Lewis bases has emerged as a powerful stereoselective strategy to deliver enantioenriched, multifunctional compounds. Capitalizing on the seminal works by the Krische^473^ and Shi’s groups,^474^ MBH carbonates or acetates proved to be easily activated by suitable chiral phosphines or tertiary amines via an S_N_2′-type mechanism affording a transient α,β-unsaturated carbonyl intermediate **XXXVII** (with the concomitant PGO^–^ release) that may be engaged in 1,4-conjugate addition reactions with suitable nucleophiles.

Concerning the addition of vinylogous furan-derived dienolates, limitations on the use of unsubstituted preformed 2-silyloxyfurans boosted the development of new methodologies in which pronucleophilic α-angelica lactones of type **XXXVIII** were first in situ activated by a base to the corresponding dienolates, enabling the γ-selective 1,4-addition (VMcR) to **XXXIX** that, through a second S_N_2′-type pathway would provide the γ-alkylated butenolides **XL** featuring adjacent quaternary and tertiary stereocenters.

**Scheme 178:**
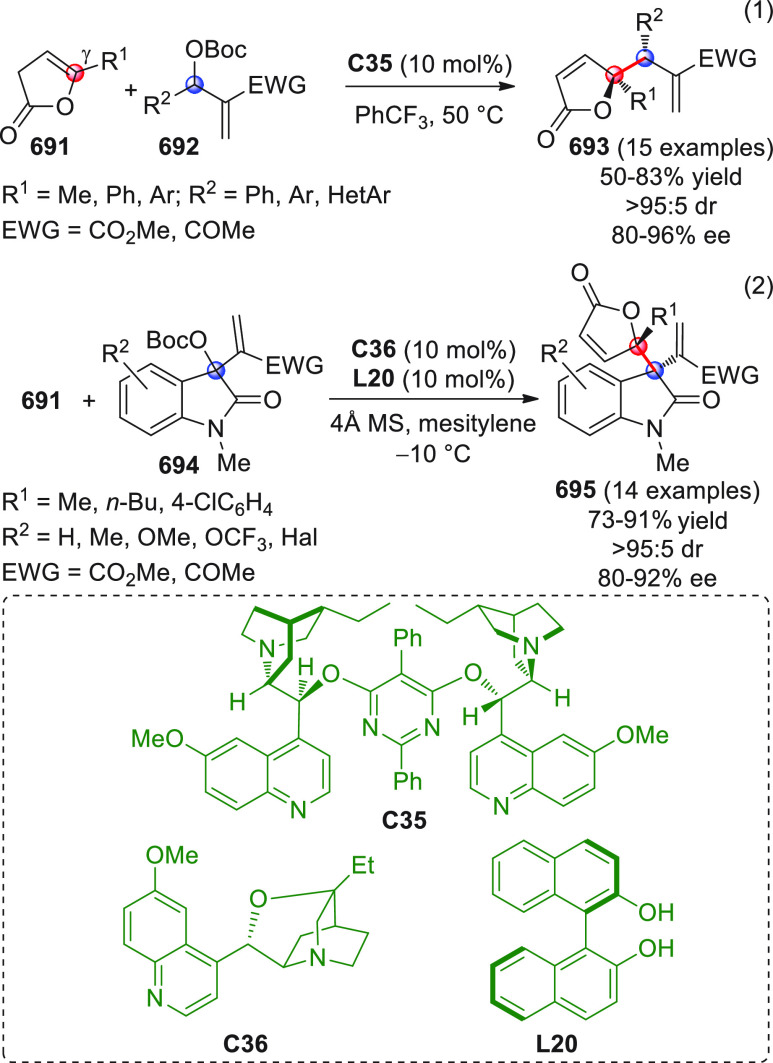


Along this line, one of the first examples of direct, enantioselective, vinylogous allylic alkylation of β,γ-unsaturated butenolides with ester- or ketone-derived MBH carbonates of type **692** to access γ,γ-disubstituted butenolides **693** was reported by Chen et al. in 2010 (Scheme 178, eq 1).^475^ After a first optimization survey, the *C*_*2*_-symmetric hydroquinidine catalyst **C35** [(DHQD)_2_PYR] was elected as the best catalyst to promote the reaction between γ-substituted lactones **691** and aromatic MBH carbonates **692** in trifluomethylbenzene at 50 °C, giving access to the corresponding γ,γ-disubstituted butenolides **693** in moderate to good yields (up to 83%) and excellent stereoselectivities (dr >95:5 and up to 96% ee).

More recently, (2015) a very similar asymmetric, vinylogous allylic alkylation between **691** and a series of MBH carbonates of type **692** was reported by Sha, Wu et al. (not shown),^476^ using a chiral, bifunctional, squaramide-phosphine catalyst. Optically active γ,γ-disubstituted butenolides **693** were accessed in good-to-excellent yields (up to 98%) and excellent stereoselectivities (up to 99:1 dr, and up to 99% ee).

Chen et al. were also engaged in the development of an organocatalytic asymmetric assembly of isatin-derived Morita–Baylis–Hillman carbonates **694** and α-angelica lactones **691** to build the structurally challenging enantioenriched oxindole frameworks **695** (Scheme 178, eq 2).^477^ The reaction, carried out in mesitylene at −10 °C in the presence of 4 Å MS was efficiently catalyzed by β-isoquinidine **C36** with the aid of slightly acidic (*R*)-BINOL additive (**L20**, 10 mol %), affording a set of multifunctional γ-adducts **695** in high yields (up to 91%) and stereoselectivities (up to 92% ee, dr >95:5).

**Scheme 179:**
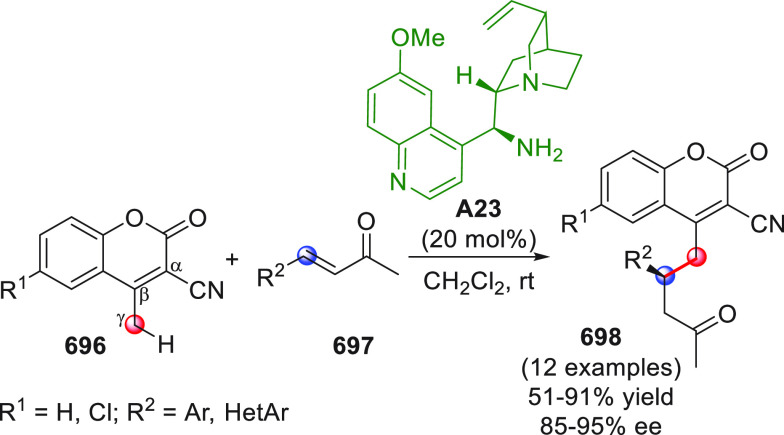


Another useful family of pronucleophiles that showed a marked versatility in direct, vinylogous transformations are the 3-cyano-4-methylcoumarins **696**, featuring an acidic γ-C(sp3)-H, which is enolizable under mild basic conditions thus enabling its γ-functionalization with suitable electrophilic partners. In this context, the first highly regio-, chemo-, and enantioselective direct VMcR of 3-cyano-4-methylcoumarin derivatives to a series of aromatic α,β-unsaturated ketones **697** was reported by Xie and co-workers in 2010, employing readily available 9-amino-9-deoxy-epiquinine **A23** as the catalyst (Scheme 179).^478^ The reaction, carried out in CH_2_Cl_2_ at rt in the presence of **A23** (20 mol %), afforded the corresponding *S*-configured, γ-homologated coumarins **698** in high yields (up to 91%) and enantioselectivity (up to 95% ee). A plausible mechanism was proposed (not shown), in which the chiral primary amine within the catalyst activated ketone **697** via formation of the corresponding iminium ion, while 3-cyano-4-methylcoumarin would be deprotonated by the quinuclidine moiety of the catalyst to generate the active dienolate species.

**Scheme 180:**
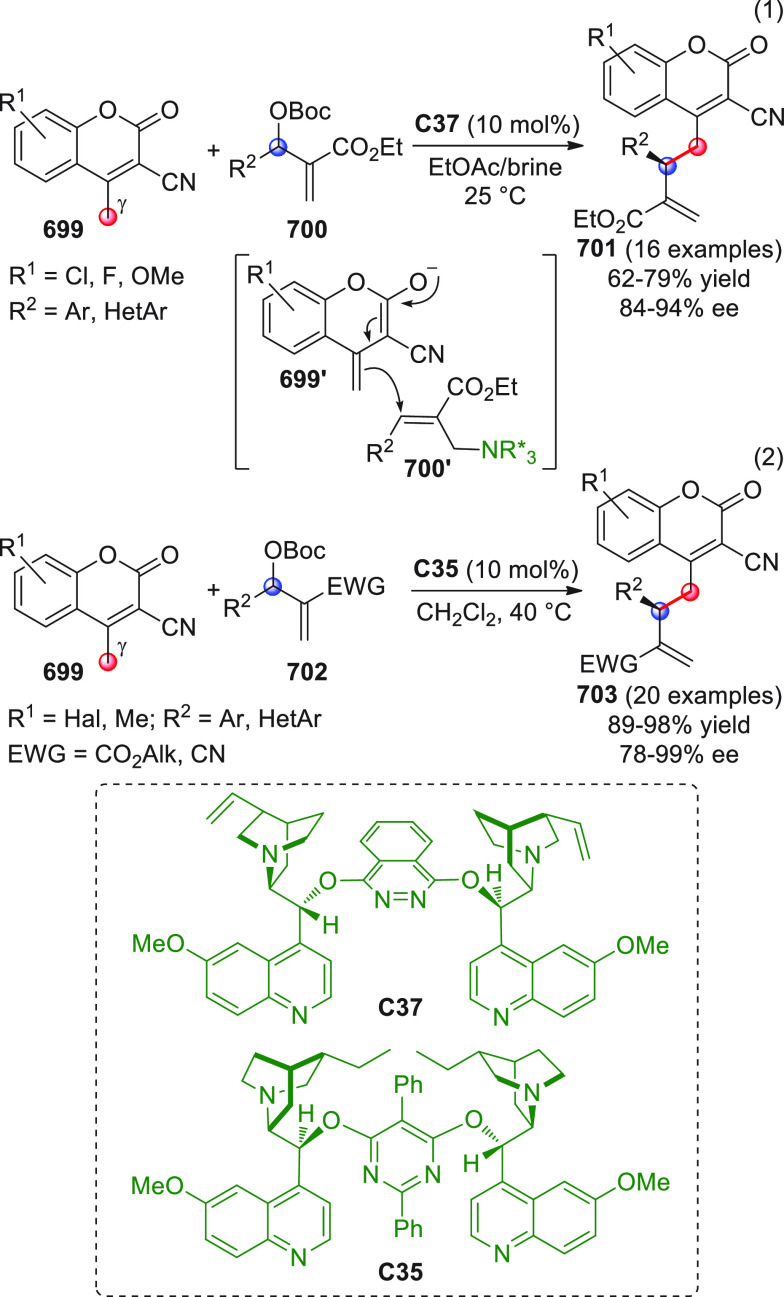


The just described application of 3-cyano-4-methylcoumarins as privileged vinylogous pronucleophiles in the enantioselective VMcR to unsaturated ketones paved the way to further applications and scope extensions. In this context, a successful organocatalyzed, enantioselective vinylogous allylic alkylation of coumarins was recently realized by Kayal and Mukherjee in 2017, using dimeric cinchona alkaloid **C37** (QD)_2_PHAL, as the catalyst of choice (Scheme 180, eq 1).^479^ Under the optimized reaction conditions, **C37** (10 mol %) promoted the reaction of differently substituted 4-methylcoumarins **699** with aromatic MBH carbonates **700** providing the corresponding γ-selective, functionalized coumarin **701** in good yields (up to 79%) and good to high enantioselectivities (up to 94% ee).

Similar results were later described by Kowalczyka and Albrecht in 2018 (Scheme 180, eq 2),^480^ who reported the allylic alkylation of ester- and nitrile-derived MBH carbonates **702** with 3-cyano-4-methylcoumarins **699** promoted by the dimeric **C35** (DHQD)_2_PYR. Here, the reaction, carried out in CH_2_Cl_2_ at 40 °C afforded the corresponding γ-adducts **703** with improved yields (up to 98%) and enantioselectivities (up to 99% ee).

**Scheme 181:**
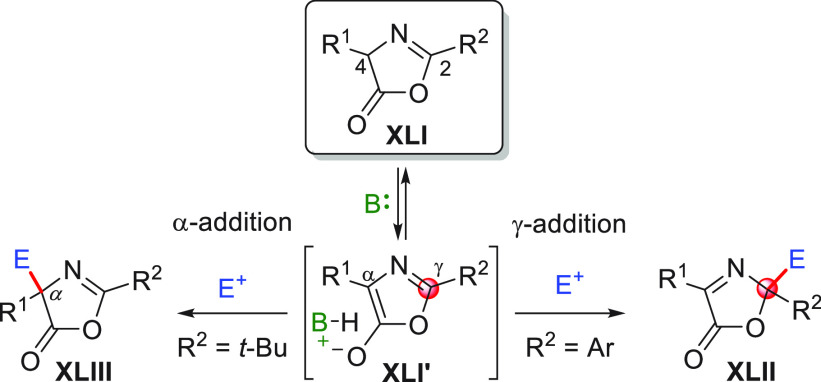


As witnessed for butenolide structures, oxazolones (azlactones) **XLI** (Scheme 181) are excellent templates for diversity-oriented syntheses of precious polyfunctionalized products, such as amino acids and heterocyclic structures.^317^ Therefore, the use of these building blocks in the field of organocatalysis constitutes an important tool in modern asymmetric synthesis. Azlactones of type **XLI** may be deprotonated at C4 by a suitable base, to provide the corresponding oxazole-dienolate **XLI′** that reacts as a bidentate nucleophile with various electrophilic partners. Interestingly, depending on the substitution pattern of the azlactone, a γ-C2 vs α-C4 addition occurs, posing the key regioselectivity issue. When 2-aryl-substituted azlactones are used, mainly vinylogous γ-addition is observed affording azlactones **XLII**, whereas 2-*tert*-butylazlactones exclusively afford the nonvinylogous α-addition regioisomers of type **XLIII** (Scheme 181).

**Scheme 182:**
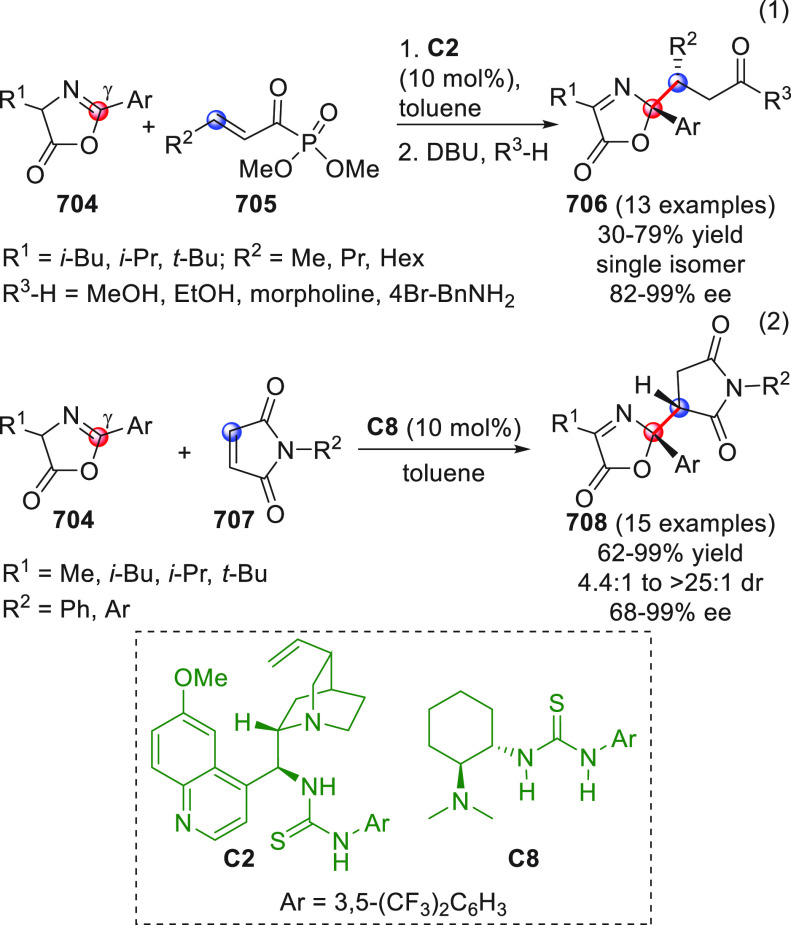


In this context, aryl-substituted oxazol-5-(4*H*)-ones (azlactones) of type **704** were exploited by Jørgensen and co-workers in 2010 as pronucleophilic scaffolds to demonstrate the excellent hydrogen-bond acceptor ability of unsaturated acyl phosphonates as electrophilic components in enantioselective, organocatalyzed Michael-type addition reactions (Scheme 182, eq 1).^481^ Indeed, it was found that bifunctional 9-*epi*-quinine-thiourea **C2** selectively orchestrated the γ-addition of azlactones **704** to a series α,β-unsaturated acyl phosphonates **705** to afford the corresponding γ-alkylated products. Overall the reaction entailed a double nucleophilic reaction, and it was shown that the acyl phosphonates could serve as masked ester or amide equivalents, which upon quenching with a second nucleophile (MeOH, EtOH, morpholine, or 4-Br-benzylamine) provided a broad spectrum of optically active conjugate adducts **706** in good yields (up to 79% yields) and high enantioselectivities (up to 99% ee). Other different nonvinylogous carbon-based nucleophiles such as indoles and 1,3-dicarbonyl compounds were tested and the obtained results served as a base to postulate a general transition state involving azlactone pronucleophile addition, in which the oxazolone dienolate reacted as an electron-rich aromatic compound, which might form π–π interactions with the electron-poor aryl group of the adjacent thiourea motif (not shown).

In the same year, Moyano, Rios, et al. developed the first highly diastereo- and enantioselective organocatalytic entry of 2,2-disubstituted-2*H*-oxazol-5-ones **708** (Scheme 182, eq 2),^482^ via γ-selective vinylogous Michael addition of aryl-substituted oxazolones **704** to symmetric *N*-aryl maleimides **707**. The C4-alkyl substitution in the pronucleophile, as well as several fluoroaryl substituents within the azlactone and maleimide scaffolds, worked well under the optimized reaction conditions, guided by Takemoto’s bifunctional thiourea catalysts **C8**, affording the corresponding adducts **708** with high yields (up to 99%) and very high regio- and stereocontrol (up to >25:1 dr, and up to 99% ee). Also, an (*S*)-isoleucine-derived chiral azlactone pronucleophile was tested, yielding the corresponding γ-adduct in 89% yield and a good 10:1 dr (not shown).

**Scheme 183:**
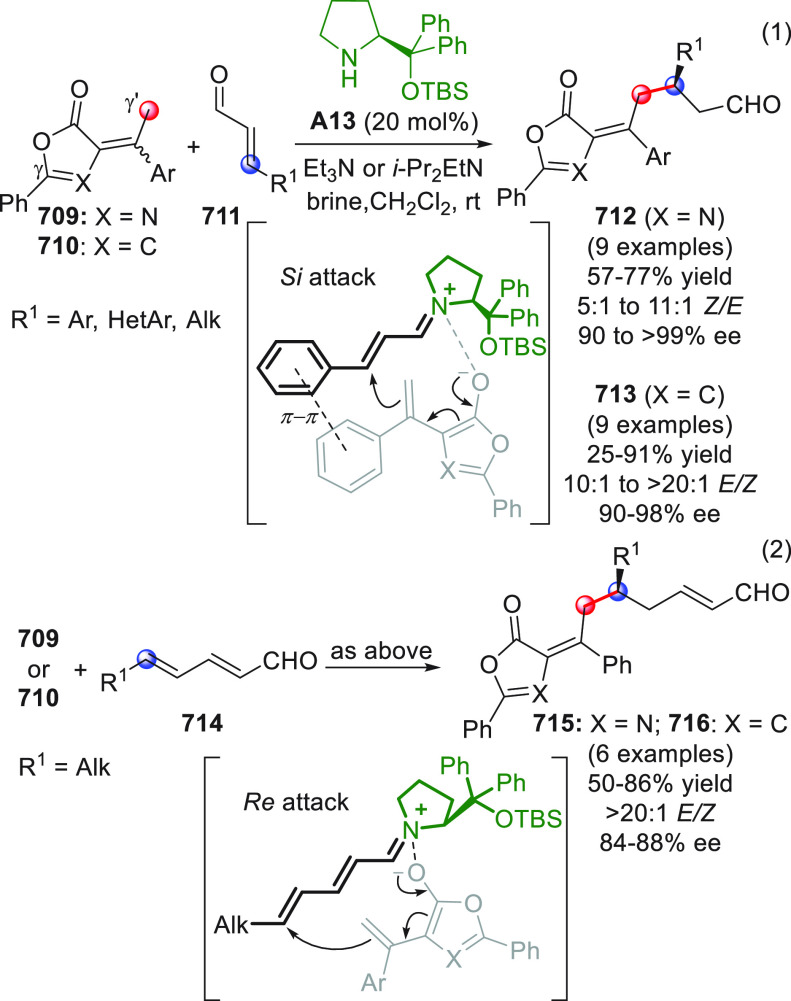


The reactivity scope of pronucleophilic azlactone and butyrolactone scaffolds in stereocontrolled vinylogous Michael-type additions was further expanded by Jørgensen and co-workers in 2013, developing a new, organocatalytic, enantioselective vinylogous 1,4- and 1,6-addition of methyl-substituted alkylidene azlactones and furanone congeners to unsaturated aldehydes (Scheme 183).^483^ Initially, the feasibility and scope of enolizable 2-phenyl-4-arylidene azlactones **709** for nucleophilic attack to both aryl- and alkyl-substituted enals **711** was tested. It was found that TBS-protected diphenylprolinol catalyst **A13** (20 mol %) in the presence of Et_3_N (20 mol %) and brine (3 equiv) in CH_2_Cl_2_ at rt were the best reaction conditions, affording optically pure γ′-adducts **712** in good yields (up to 77% yield) and almost perfect stereocontrol (Scheme 183, eq 1).

Also, methyl- and iodo-substituted aromatic systems within the ylidene chain of **709** were tolerated giving comparable performance. Similar reaction conditions were then applied to the reaction between 5-phenyl-3-arylidene butyrolactone congeners **710** and enals **711**. Here, catalyst **A13** (20 mol %) with the aid of *i*-Pr_2_EtN (20 mol %) and brine (3 equiv) provided the corresponding *S*-configured γ′-homologated adducts **713** in high yields and stereoselectivity (eq 1). Finally, in order to extend the generality and the potential of the disclosed reaction, the authors applied the developed methodology to linear 2,4-dienals **714** (Scheme 183, eq 2). Remarkably, applying the latter conditions to the reaction between phenyl-substituted azlactones **709** or furanones **710** and alkyl substituted dienals **714**, only the 1,6-addition took place, providing the corresponding enantioenriched γ′-adducts **715** and **716** with a full control of the double-bond geometry (>20:1 *E/Z* for both olefins). Interestingly, the 1,6-selectivity turned out to be dependent on the nature of the 2,4-dienal: in fact, addition of **709** or **710** to a phenyl-substitued dienal congener proceeded with lower conversions, yielding a mixture of 1,4- and 1,6-addition products (not shown). Suitable transition states were proposed by the authors to justify the observed regio- and enantioselectivities. Indeed, it was postulated that the negatively charged oxygen atom in the vinylogous dienolate species could be stabilized by the positively charged nitrogen atom of the iminium-ion intermediate, aligning the electrophilic β- or δ-carbon atom of the iminium-ion intermediate with the γ′-position of the dienolate promoting the high site-selective 1,4 vs 1,6 conjugate addition (Scheme 183). Furthermore, π–π stacking interaction between the β-aryl group of enal and the β′-aryl moiety of dienolate is proposed to be an important factor, stabilizing the transition state of the 1,4-addition, while the stereochemical outcome of the vinylogous additions toward enals and 2,4-dienals would be controlled by the shielding effect of the TBS-protected prolinol catalyst **A13**.

**Scheme 184:**
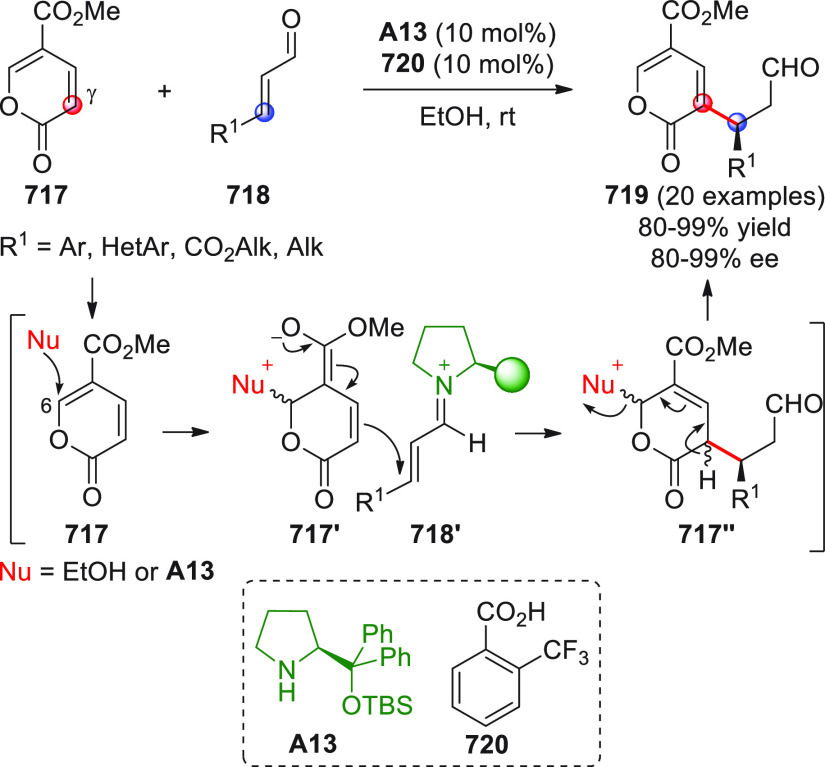


Finally, it is worth mentioning the direct, organocatalytic, enantioselective intermolecular cross- vinylogous Rauhut–Currier reaction of methyl coumalate **717** and α,β-unsaturated aldehydes **718** recently developed by Liu and Zu, in which the enals were activated via iminium ion catalysis to serve as the Michael acceptors and methyl coumalate was used as an activated diene to generate the latent enolate (Scheme 184).^484^ Indeed, prolinol silylether **A13** in the presence of 2-trifluoromethylbenzoic acid (**720**) as cocatalyst promoted the conjugate addition of pronucleophilic ester **717** to a varied repertoire of aromatic and aliphatic enals **718** to provide enantioenriched products **719** in high yields (up to 99%) and enantioselectivity (up to 99%). Several alternative pathways were proposed with a common pattern: a first nucleophilic, conjugate addition of EtOH or the prolinol to the C6 of methyl coumalate **717** would generate a transient dienolate **717′**, which could trigger the subsequent intermolecular 1,4-addition to the chiral, α,β-unsaturated iminium ion **718′**. The resulting adduct **717′′** could then undergo rapid aromatization-driven elimination to deliver the final product **719**.

#### 5.3.2 Indirect Procedures

##### 5.3.2.1 Acyclic Nucleophiles

One of the few VMMcR involving linear silyloxydienes was introduced in 2015 by Wu and Li, who developed an In(III)-catalyzed vinylogous addition of diverse γ-substituted (alkyl, aryl, benzyl) *O*- tris(trimethylsilyl)silyl vinylketene acetals **721** to 2,3-dihydro-4-pyridinones **722** (Scheme 184, eq 1).^485^

**Scheme 185:**
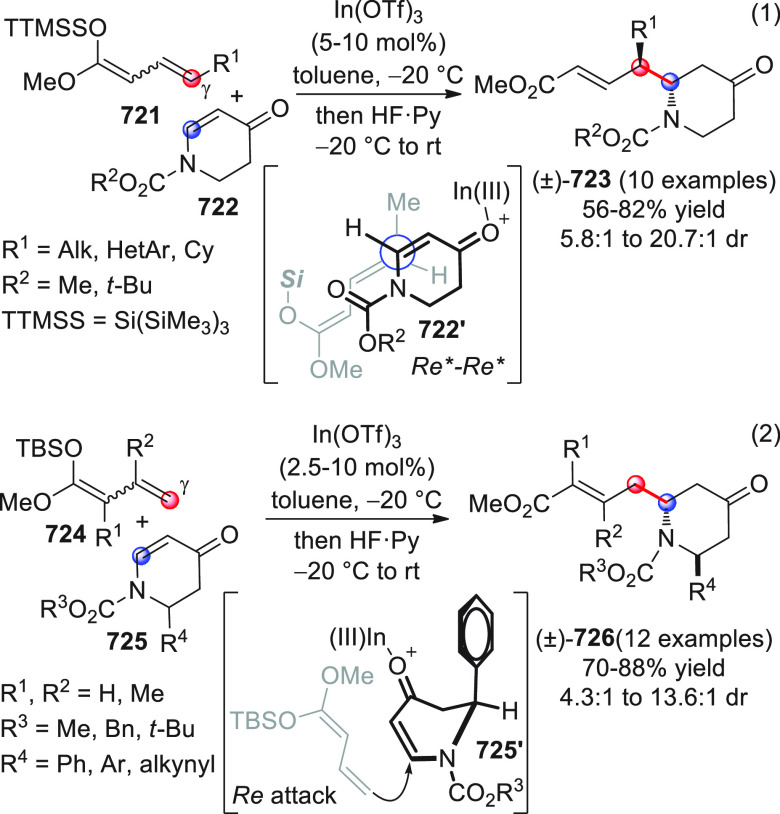


Interestingly, the supersilyl group (TTMSS) was found to be the best silyl group within the nucleophile to influence the γ vs α regiochemical control of the VMMcR promoted by In(OTf)_3_ (5–10 mol %) in toluene at −20 °C: in fact, the corresponding *anti*-configured γ-alkylated products of type (±)-**723** could be obtained in good yields (up to 82%) and high diastereocontrol (up to 20.7:1). A plausible antiperiplanar transition state was proposed, in which a secondary orbital overlap (i.e., *π–π* stacking interactions) between the vinylketene moiety of **721** and the carbamate of the activated acceptor **722′** favored a *Re–Re* approach. Furthermore, γ-unsubstituted *O*-TBS vinylketene acetals **724** were also tested as nucleophiles in the VMMcR to various 2-substituted dihydropyridinones **725** (Scheme 185, eq 2). Under the same reaction conditions, a range of *trans*-disubstituted 4-piperidinones (±)-**726** were accessed in comparable yields and diastereoselectivity, without the need of the more hindered supersilyl group. To account for the observed *anti*-facial selectivity, a combination of stereoelectronic and steric control was considered.

The author proposed a preferred half-boat conformation of activated **725′**, with the C2 phenyl group adopting a pseudoaxial position to avoid diaxial 1,3-interactions with the *N*-Boc group. This would promote the vinylogous, bottom face addition of silyloxydiene **724**, providing the observed *anti*-configured adducts.

**Scheme 186:**
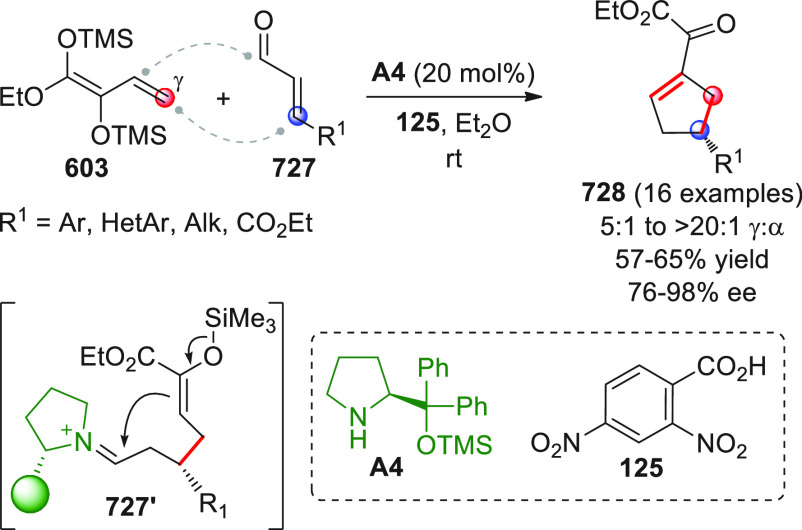


In 2015, Schneider and Nareddy disclosed a highly enantioselective access to chiral 1-cyclopentenyl-α-keto esters **728** through the implementation of an organocatalytic, domino VMMcR/intramolecular Knoevenagel-type condensation of linear bis-silyl-1,3-dienediolate **603** to a varied set of aromatic and aliphatic α,β-unsaturated aldehydes **727** (Scheme 186).^486^

Using the Hayashi–Jørgensen prolinol TMS-ether **A4** as the chiral organocatalyst, the reaction, carried out in Et_2_O under buffered conditions (pH 4), in the presence of an acidic additive such as 2,4-dinitrobenzoic acid **125** (1.0 equiv), afforded the 1-cyclopentenyl-α-keto esters **728** with high γ-regioselectivites (up to >20:1 γ:α), moderate to good yields (up to 65%), and excellent enantioselectivities (up to 98% ee). Mechanistically, this process involved a first, γ-selective intermolecular VMMcR between dienediolate **603** and the iminium ion derived by the covalent activation of the unsaturated aldehyde **727** by **A4**, generating a chiral, iminium ion intermediate **727′** that was then annulated by an intramolecular Knoevenagel-type condensation.

##### 5.3.2.2 Cyclic Nucleophiles

**Scheme 187:**
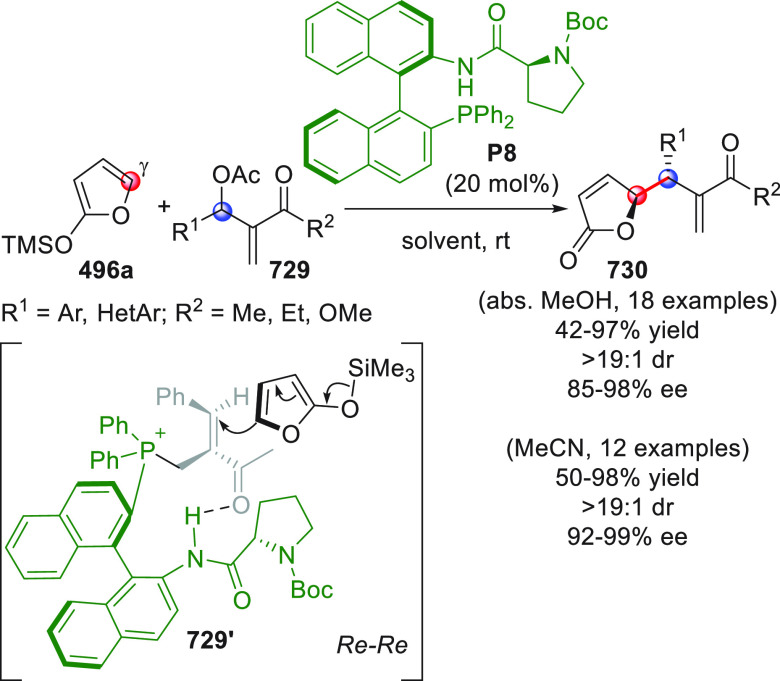


An indirect vinylogous, allylic alkylation of Morita–Baylis–Hillman (MBH) acetates **729** with TMSOF **496a** was developed by Shi and co-workers in 2011, introducing a series of multifunctional, chiral amide–phosphane scaffolds as useful organocatalysts (Scheme 187).^487^ Indeed, chiral amide-diphenylphosphane **P8** promoted the reaction of **496a** and **729** under mild reaction conditions in absolute MeOH or MeCN, giving access to the corresponding *anti*-configured γ-homologated butenolides **730** in good to excellent yields (42–98%) and high enantioselectivities (85–99%). The reaction was quite general, so that aromatic and heteroaromatic ketones and ester derived MBH acceptors **729** were good substrates for the reaction. As for the mechanism, it was proposed that a tandem S_N_2′/S_N_2′ substitution pathway could be operative (see Scheme 177), and NMR tracing experiments provided evidence for the existence of phosphonium ion intermediate **729′** being involved in the proposed Diels–Alder-like transition state (Scheme 187, bottom).

**Scheme 188:**
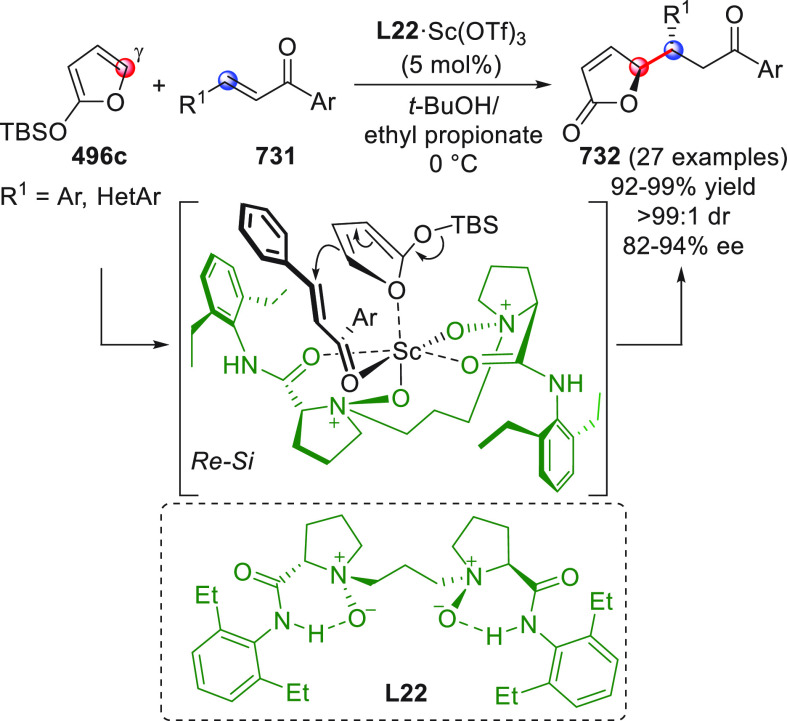


A highly efficient catalytic, asymmetric VMMcR of 2-silyloxyfuran with chalcone derivatives **731**, catalyzed by a chiral *N,N*′-dioxide–scandium(III) complex, was reported by Feng et al. in 2011 (Scheme 188).^488,489^ In particular, 2-(*tert*-butyldimethylsilyloxy)furan (TBSOF, **496c**) was coupled with a large set of differently substituted aromatic α,β-unsaturated ketones **731** in a *t*-BuOH/ethyl propionate mixture at 0 °C, under the guidance of Feng’s Sc(OTf)_3_-*N,N*′-dioxide ligand **L22**, to afford the sole 5,1′-*anti*-configured γ-adducts **732** in very high yields (up to 99%) and good to excellent enantioselectivities (up to 94% ee).

To account for the observed (5*S*,1′*S*)-stereoselectivity, a plausible transition state model was proposed in which the *N*-oxide and amide oxygen atoms of **L22** coordinated to scandium ion in a tetradentate manner to form two six-membered chelate rings. The chalcone acceptors **731** bound to scandium in a favorable equatorial position, displaying the less hindered *Si* face to the attack by the *Re* face of the nucleophile.

**Scheme 189:**
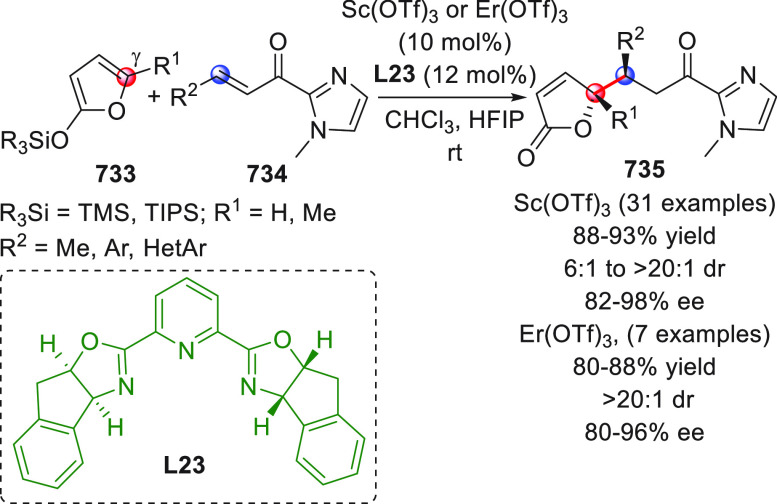


Another asymmetric, metal-catalyzed VMMcR of silyloxyfurans **733** with α,β-unsaturated 2-acyl imidazoles **734** was recently reported by Singh et al. using either chiral Sc(III) or Er(III) complexes with a chiral, *C*_*2*_-symmetric ligand **L23** (Scheme 189).^490^ In particular, the Sc(OTf)_3_·**L23** catalyzed VMMcR of silyloxyfurans **733** and a vast set of aryl and Me-substituted acyl imidazoles **734**, carried out in CHCl_3_ at rt in the presence of hexafluoroisopropanol (HFIP) as an additive, provided the expected γ-adducts **735** in good yields (up to 93%) with excellent diastereo- and enantioselectivity (up to >20:1 dr and 98% ee). Also, catalytic Er(OTf)_3_·**L23** complex worked well under similar reaction conditions, affording several γ-adducts **735** in comparable yields and selectivities.

**Scheme 190:**
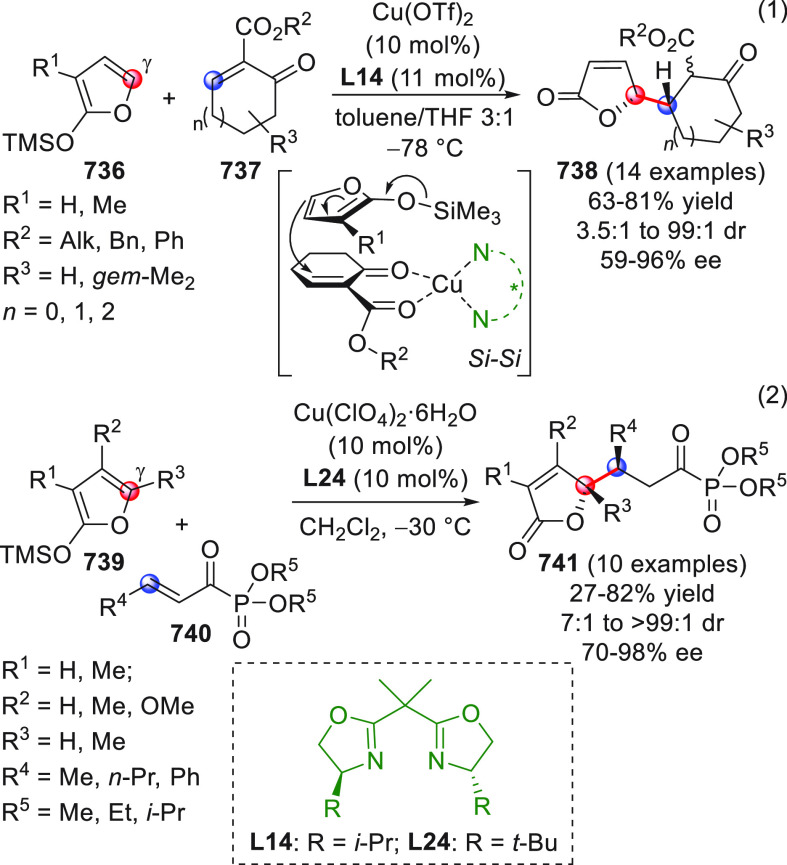


Switching to VMMcR involving cyclic electrophilic partners, the first enantioselective, Cu(II)-catalyzed asymmetric addition of 2-silyloxyfurans **736** to cyclic unsaturated oxo esters **737** was developed by Chabaud, Guillou, et al. in 2013 (Scheme 190, eq 1).^491,492^ Here, the *C*_*2*_-symmetric isopropyl-box (**L14**)·Cu(OTf)_2_ complex was elected as the best chiral catalytic system for the VMMcR involving **736** and a series of differently sized and substituted α,β-unsaturated oxo esters **737** providing the corresponding γ-adducts **738** in good yields (up to 81%) and excellent diastereocontrol (usually dr 99:1), albeit with modest to high enantioselectivities (59–96% ee), depending on the nature of the ester group and the substitution of the cyclic oxo ester.

A similar catalytic system was also exploited by Bolm and co-workers in 2015 for the synthesis of valuable enantioenriched, phosphorus-containing compounds through an asymmetric Cu(II)-catalyzed VMMcR between differently substituted cyclic dienol silanes **739** and α,β-unsaturated α-keto phosphonates **740** (Scheme 190, eq 2).^493^

Using the combination of the commercially available *C*_*2*_-symmetric bisoxazoline ligand **L24** with copper(II) perchlorate hexahydrate salt [Cu(ClO_4_)_2_·6H_2_O], the VMMcR of **739** and **740** provided a variety of γ-homologated butenolides **741** in moderate to high yields (up to 82%) and high stereoselectivities (up to 99:1 dr and up to 98% ee). Of note, when the 4-methoxy-furan derivative was used as the nucleophilic partner, a different chemoselectivity was observed, providing the 1,2 aldol adduct instead of the 1,4, in a low 22% yield, 2.3:1 dr, and 97% ee (not shown).

**Scheme 191:**
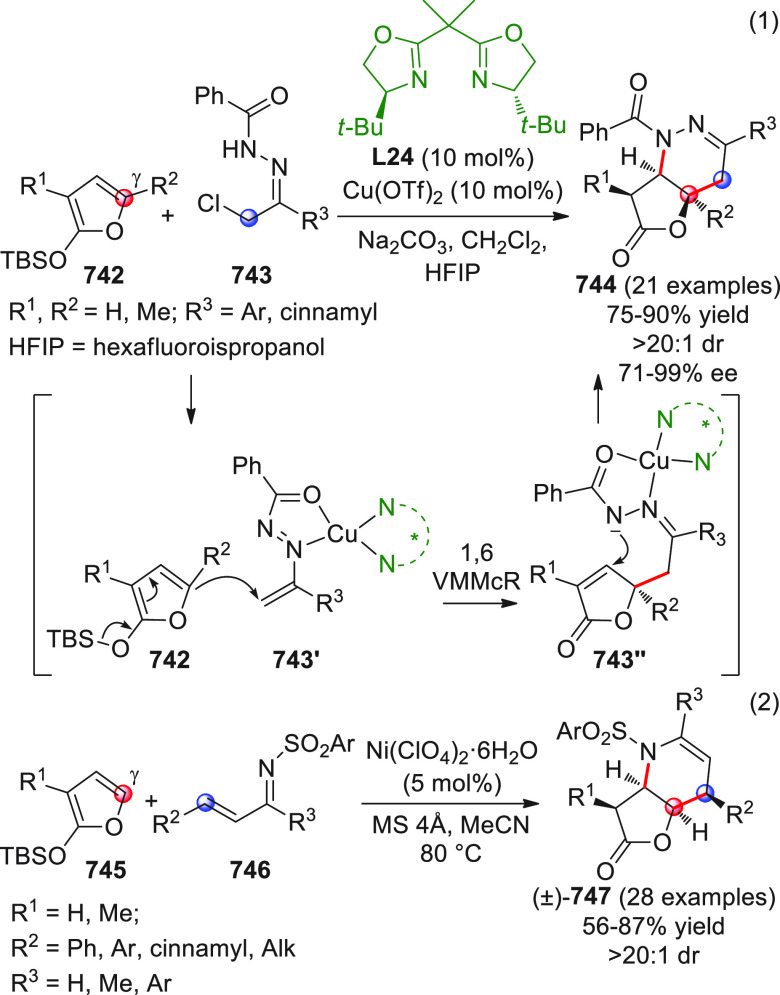


An unprecedented vinylogous 1,6-Mukaiyama Michael/*aza*-Michael cascade of 2-silyloxyfurans **742** and in situ formed electrophilic azoalkenes of type **743′** was realized by Wang et al. in 2015 (Scheme 191, eq 1).^494^ After a deep optimization survey, Cu(II)·**L24** complex was found to be the best catalytic system to enable the in situ formation of electrophilic azoalkenes **743′** from the corresponding alkyl chlorides **743** (with the aid of Na_2_CO_3_), triggering the γ-regioselective, 1,6-Mukaiyama Michael reaction of **742** to **743′**. The *aza*-anion intermediate **743′′** thus formed cyclized via intramolecular conjugate addition, to afford the final butyrolactones **744** in good yields (up to 90%) with excellent stereoselectivity (up to 20:1 dr, and up to 99% ee).

Following this strategy, the same group developed a nickel-catalyzed 1,6-VMMcR/aza-Michael cascade of 2-silyloxyfurans **745** with *N*-sulfonyl-1-aza-1,3-dienes **746**, affording the corresponding fused piperidine/lactone scaffolds (±)-**747** with excellent diastereoselectivity (>20:1) and highly functional group tolerance (Scheme 191, eq 2).^495^

**Scheme 192:**
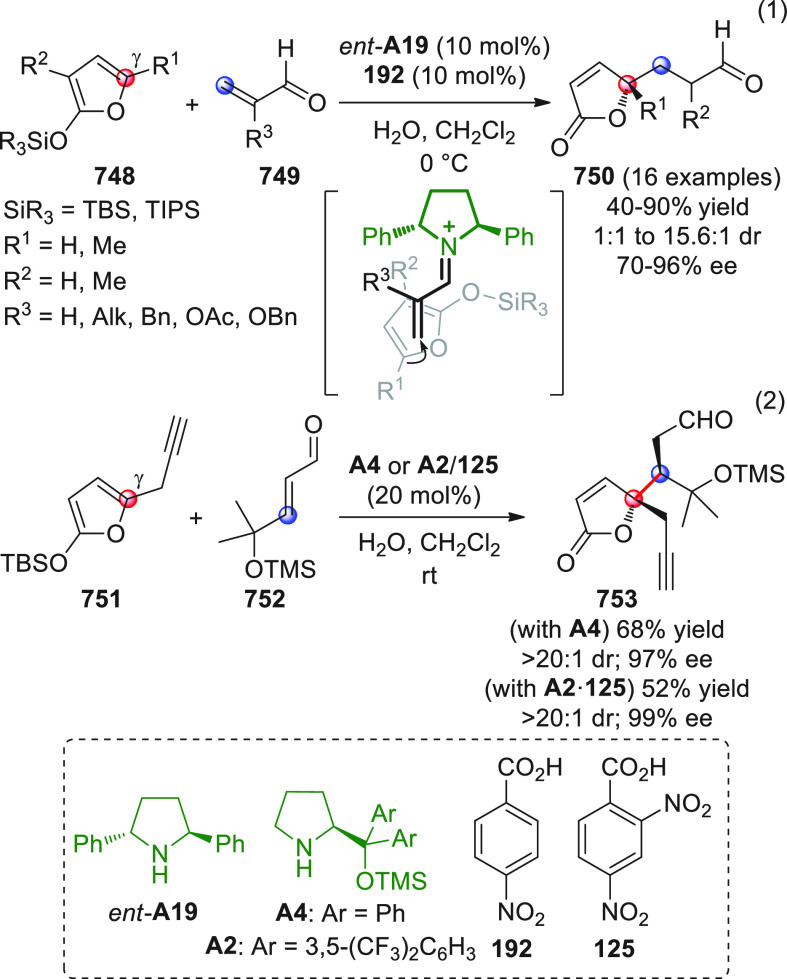


In addition to these metal-catalyzed asymmetric methodologies, a series of useful organocatalytic, enantioselective vinylogous Michael-type transformations were developed and applied to the total synthesis of valuable target molecules. Notably, enantioselective, iminium-catalyzed (LUMO-lowering) reactions of cyclic silyl ketene acetals to simple acrolein and methacrolein acceptors were introduced by Pihko and co-workers in 2012 and 2014, en route to the synthesis of the key C17–C28 segment of the cytotoxic marine natural products pectenotoxins (Scheme 192, eqs 1 and 2).^496−498^ Indeed, the authors envisaged the possibility to access key 5*S*-configured γ,γ-disubstituted butenolide intermediates **750** by implementing an asymmetric VMMcR between substituted furanone **748** and a suitable enal **749** like acrolein (R^3^ = H) or methacrolein (R^3^ = Me), promoted by a chiral secondary amine such as *C*_*2*_-symmetric 2,5-diphenylpyrrolidine *ent*-**A19**, which was the most suited for the purpose.

Although a wide range of iminium-catalyzed enantioselective reactions are known also in the field of vinylogous transformations, in the vast majority of cases the reactions have been restricted to β-substituted enals, so that enantioselective additions to α-substituted enals such as methacrolein (typically resulting an unreactive substrates) were quite challenging.^499,500^ In this context, Pihko disclosed that pyrrolidine *ent*-**A19** (10 mol %) in the presence of 4-nitrobenzoic acid **192** (10 mol %) as cocatalyst and some amount of H_2_O (2.0 equiv) was an effective catalyst in promoting the VMMcR between silyloxyfurans **748** and β-substituted enals **749** (Scheme 192, eq 1), providing the corresponding γ-homologated products **750** in good yields (up to 90%) and stereoselectivities (up to 15.6:1 dr and up to 96% ee). Interestingly, the far from obvious rationalization of the observed enantioselectivity was supported by DFT computational studies, which unveiled the presence of attractive noncovalent interactions as key factors in controlling the enantiocontrol of the reaction. On these bases, a plausible transition state was proposed in which the *Si* face of silyloxyfuran **748** attacks the iminium intermediate via a Diels–Alder-like approach.

Another iminium-ion (LUMO-lowering activation), organocatalyzed VMMcR between γ-propargyl silyloxyfuran **751** and silyl enal **752** was exploited by Xie and co-workers in 2016 for the stereocontrolled construction of the C5-*epi* ABCDE-ring system of rubriflordilactone B (Scheme 192, eq 2).^501^ Irrespective of the bulkiness of the reagents, chiral prolinol silyl ethers **A4** or **A2/125** (20 mol %) catalyzed the VMMcR in the presence of H_2_O (2.0 equiv), allowing the construction of optically pure γ,γ-disubstituted butenolide **753** in good yield as a sole isomer (>20:1 dr).

**Scheme 193:**
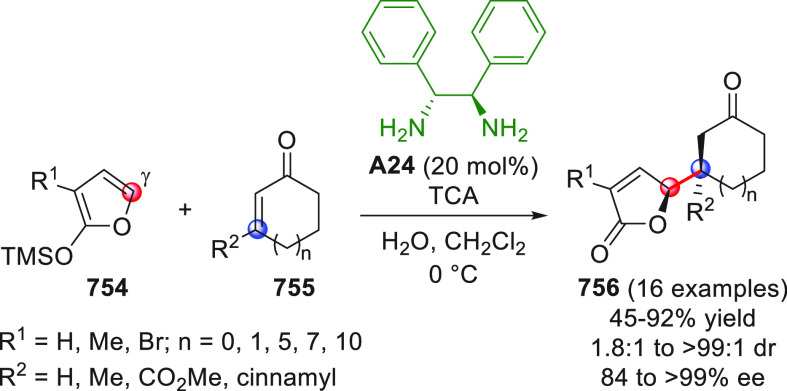


Finally, a covalently activated, asymmetric, organocatalyzed VMMcR of 2-silyloxyfurans **754** to medium and large cyclic enones of type **755** was reported by Singh and co-workers in 2015 (Scheme 193).^502^ In this report, chiral, *C*_*2*_-symmetric primary diamine **A24** (20 mol %) in the presence of trichloroacetic acid (TCA, 20 mol %) promoted the efficient *syn*-selective addition of **754** to **755**, to provide the corresponding γ-homologated butenolide **756** in good yields (up to 92%) and high stereoselectivities (up to >99:1, and up to >99% ee).^503−505^

### 5.4 Other Reactions

#### 5.4.1 Direct Procedures

##### 5.4.1.1 Acyclic Pronucleophiles

**Scheme 194:**
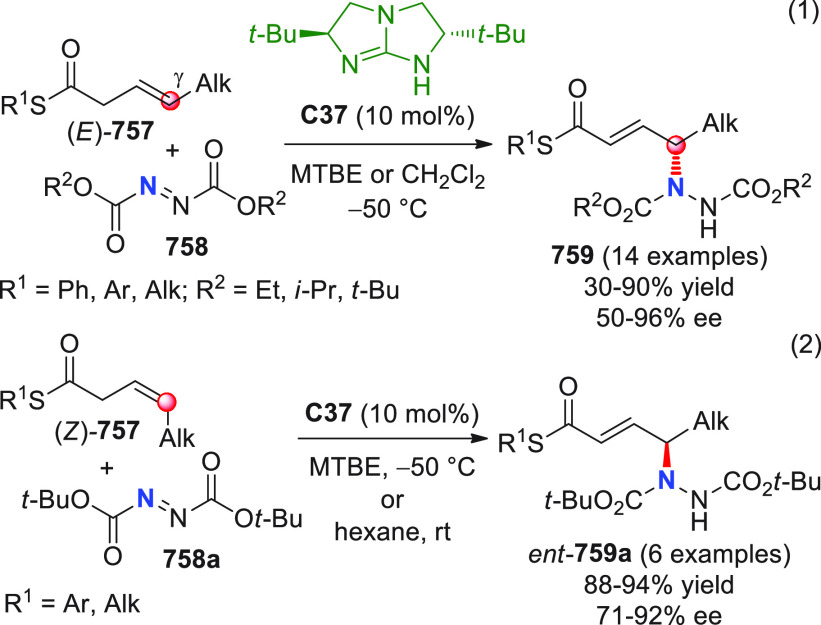


In 2012, Tan and co-workers devised a guanidine-catalyzed, enantiodivergent, vinylogous allylic amination between linear, unconjugated β,γ-unsaturated thioesters **757** and dialkyl azodicarboxylates **758** (Scheme 194).^506^ Interestingly, the reaction catalyzed by chiral guanidine **C37** and performed on (*E*)-**757** afforded the corresponding 4*S*-configured γ-adducts **759** in good yields (up to 90%) and enantioselectivity (up to 96% ee). Coversely, starting from (*Z*)-**757** and di-*tert*-butylazodicarboxylates **758a**, under similar reaction conditions, the enantiodivergent access to 4*R*-configured adducts *ent*-**759a** could be assessed with comparable efficiency and selectivity. Computational studies were also performed envisaging a side-on mechanism with the formation of an *s*-*trans* dienolate (not shown). The theoretical study agreed well with experimental results providing an intuitive explanation for the inversion of the absolute configuration.

**Scheme 195:**
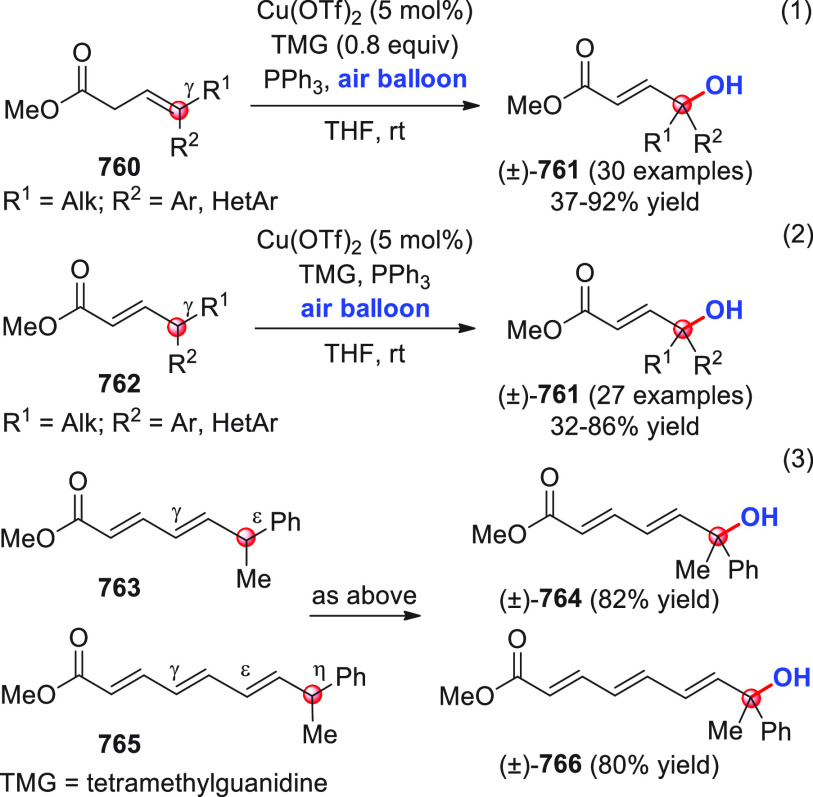


More recently, a Cu(II)-catalyzed, vinylogous aerobic oxidation of unsaturated esters with air was reported by Newhouse, Yin, et al. (Scheme 195).^507^ Under the optimized reaction conditions, a mild and operationally simple Cu(OTf)_2_-catalyzed vinylogous aerobic oxidation of β,γ-unsaturated ester **760** and the corresponding α,β-unsaturated congener **762** afforded γ-hydroxy ester derivatives (±)-**761** in good yields (up to 86–92%) as single diastereoisomers (Scheme 195, eqs 1 and 2). Furthermore, under similar reaction conditions, bisvinylogous and hypervinylogous oxidations were performed, yielding the corresponding polyunsaturated products (±)-**764** and (±)-**766** with comparable yields and selectivities (Scheme 195, eq 3). Of note, tetramethylguanidine (TMG) was found to be crucial both as a base and as a key ligand to produce the active Cu(II) catalyst.

##### 5.4.1.2 Cyclic Pronucleophiles

**Scheme 196:**
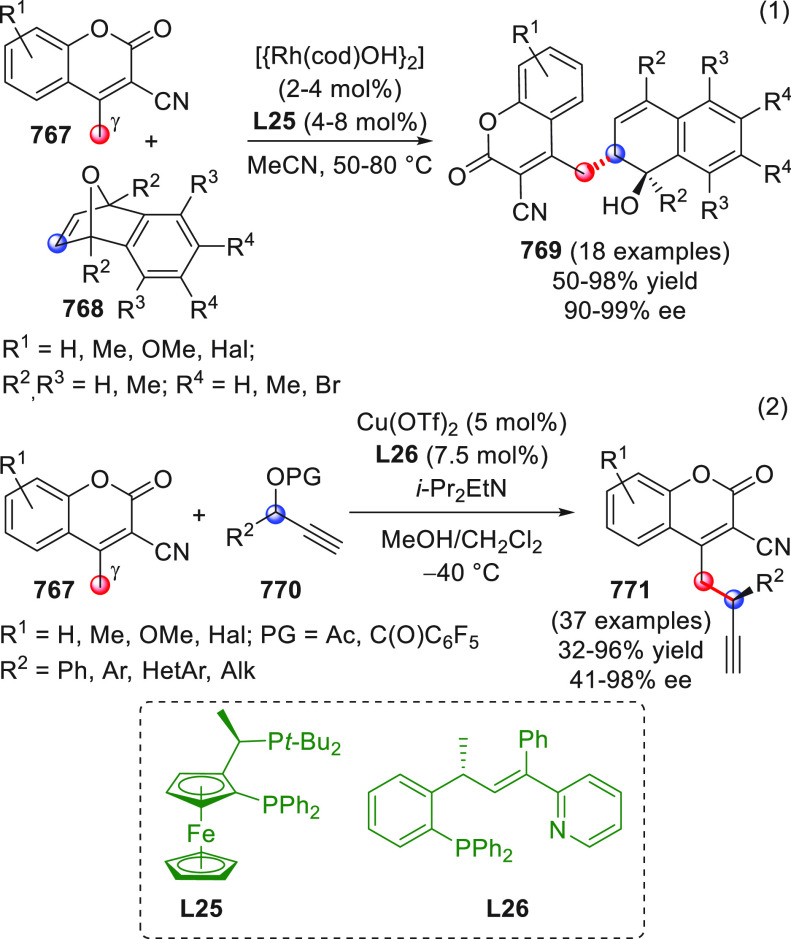


A rhodium-catalyzed, enantioselective, vinylogous epoxide-opening between methyl cyanocoumarin **767** and strained, olefinic epoxides **768** was recently devised by Lautens and co-workers, demonstrating, for the first time, how the principle of vinylogy can be applied also to the release of ring strain in polycyclic systems (Scheme 196, eq 1).^508^ Using a [Rh(cod)OH]_2_/**L25** catalytic system, a series of chiral enantiopure (up to 99% ee) γ-adducts **769** were accessed in high yields (up to 98%). In general, the devised protocol tolerates a wide spectrum of substrates with varied electronic and steric properties.

Substituted methyl-cyanocoumarins **767** were also exploited by Antonchick, Waldmann, et al. as suitable pronucleophiles in a highly enantioselective copper-catalyzed vinylogous propargylic substitution, which afforded enantioenriched propargylic coumarins of type **771**, which were also studied as autophagy inhibitors (Scheme 196, eq 2).^509^ Aromatic and aliphatic propargylic esters **770** reacted smoothly with substituted coumarins **767** under the guidance of the Cu(OTf)_2_/**L26** catalytic system, to give the desired products with excellent yields (up to 96%) and enantioselectivities (up to 98% ee).

**Scheme 197:**
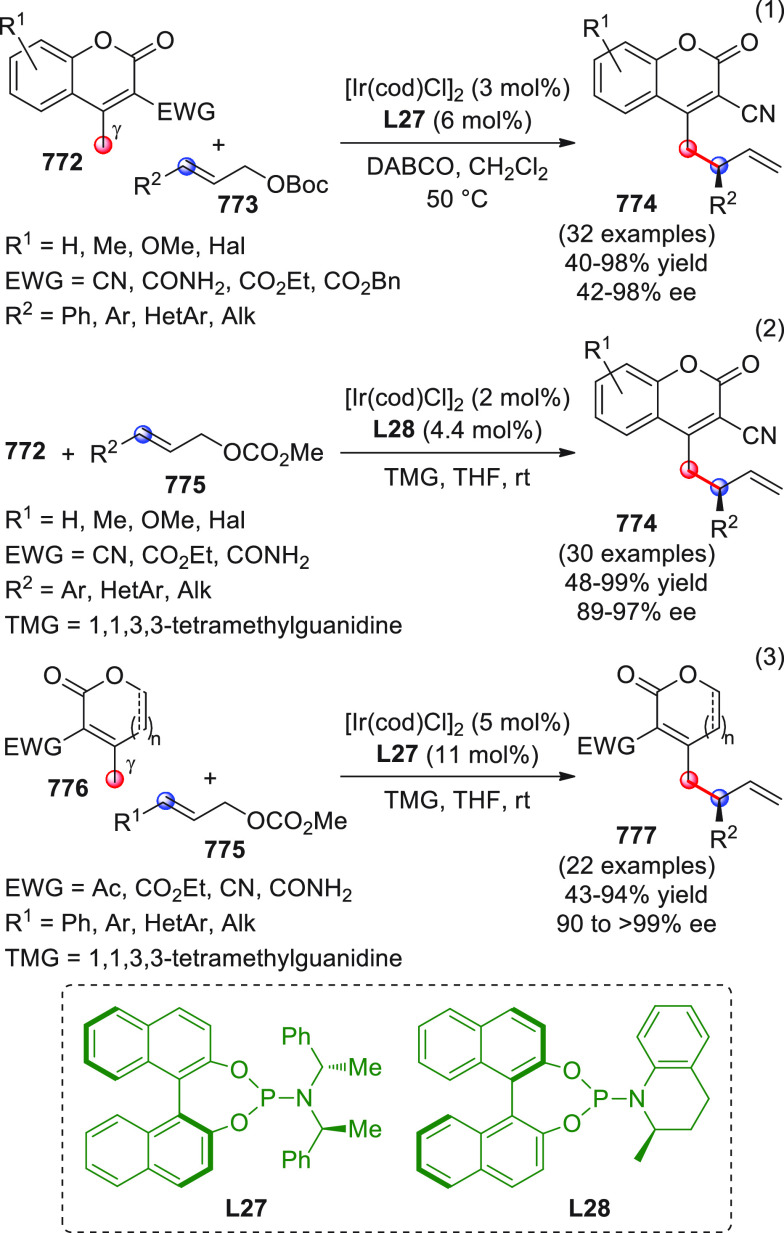


Transition metal-catalyzed, asymmetric allylic substitution reactions have emerged as an extremely powerful and versatile method for the synthesis of chiral, enantioenriched compounds from easily available starting materials through the enantioselective construction of carbon–carbon and carbon–heteroatom bonds. In this context, the first iridium-catalyzed enantioselective vinylogous allylic alkylation of coumarins has been recently reported almost contemporarily and independently by the groups of Mukherjee and Yin in 2018 (Scheme 197). In particular, using easily accessible linear allylic carbonates **773**, as allylic electrophiles (eq 1), Mukherjee and co-workers^510^ found that [Ir(cod)Cl]_2_ complexed with chiral ligand **L27** effectively promoted the vinylogous allylic alkylation of 4-methylcoumarins **772**, in an exclusively branched-selective manner (>20:1 branched vs linear), affording the corresponding γ-adducts **774** in generally high yields (up to 98%) with an excellent level of enantioselectivity (up to 98%). Similarly, the catalytic asymmetric, vinylogous allylic alkylation of 4-methylcoumarins **772** and differently shaped, α,β-unsaturated lactones of type **776** was achieved by Yin et al.^511^ using allylic carbonates of type **775** and suitable ligands such as **L27** or **L28** to be complexed with [Ir(COD)Cl]_2_ (Scheme 197, eqs 2 and 3). Several γ-functionalized adducts of type **774** and **777** were obtained, in good yields, and with excellent regio- and enantioselectivities.

#### 5.4.2 Indirect Procedures

##### 5.4.2.1 Cyclic Nucleophiles

**Scheme 198:**
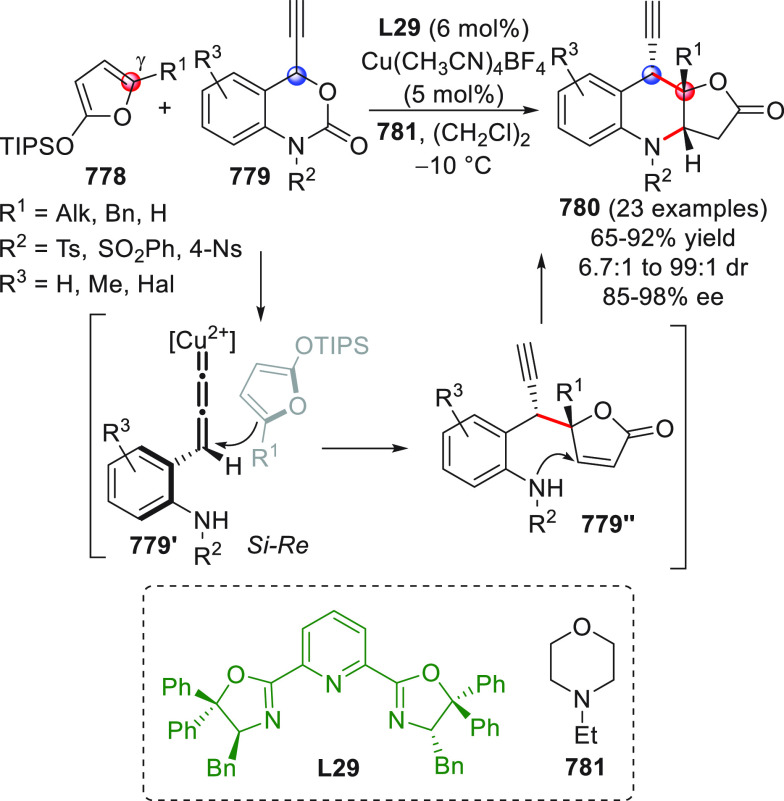


Cao, Wu, et al. introduced a novel decarboxylative formal [4 + 2] cycloaddition reaction between ethynyl benzoxazinanones **779** and 5-substituted 2-silyloxyfurans of type **778** (Scheme 198).^512^ The reaction, catalyzed by the chiral copper/**L29** complex in the presence of ethyl morpholine (**781**) as a base, provided enantioenriched tetrahydroquinolines fused with a butyrolactone moiety **780**, featuring three contiguous stereocenters, in high yields (up to 92%) and excellent diastereo- and enantioselectivities (up to 99:1 dr and up to 98% ee).

Concerning the mechanism, upon conversion of benzoxazinanone scaffolds **779** into the corresponding Cu–allenylidene species **779′**, a first γ-selective addition of **778** to the C4 of **779′** took place affording the γ-homologated butenolide intermediate **779′′**, that undergoes an intramolecular aza-Michael reaction providing the final tetrahydroquinoline targets **780**. To account for the observed selectivity, a transition state was proposed in which the *Si* face of **778** approached the relatively less sterically hindered *Re* face of the Cu–allenylidene complex **779′**.

**Scheme 199:**
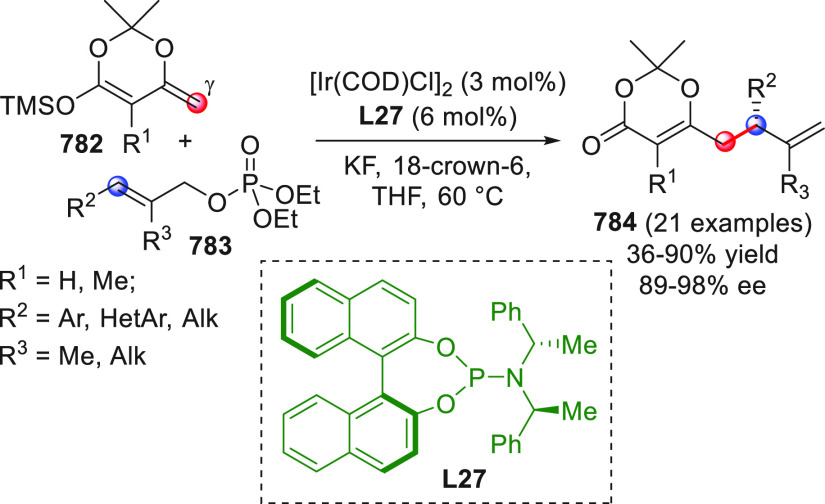


A catalytic, enantioselective, Ir-catalyzed vinylogous allylic alkylation of trisubstituted allylic electrophiles such as allylic phosphates **783** with dioxinone-derived dienoxysilanes **782** was developed by Hartwig and Chen in 2016 (Scheme 199).^513^ By surveying different leaving groups within allylic electrophiles, the authors found that trisubstituted allylic phosphates **783** were suitable electrophiles for asymmetric allylation promoted by the Ir/phosphoramidite ligand **L27** system, affording the corresponding branched allylated products **784** in good yields (up to 90%) and high stereoselectivity (12:1 to 20:1 branched vs linear; 5.1 to 8.1 γ vs α; up to 98% ee).

## 6 Vinylogous Amides and Lactams

Section 6 surveys original research appearing between 2010 and 2018 and dealing with the exploitation of α,β-unsaturated amides and lactams as vinylogous donors in asymmetric synthetic methodologies.

The ability to construct functionality-rich chiral amide- or lactam-based molecular entities in a chemo-, regio-, and stereocontrolled manner has long been the subject of extensive studies, given the widespread presence of these matrices in bioactive natural products as well as in nature-inspired synthetic intermediates and therapeutics. In this research domain, alongside the attention to the traditional and well-used aldol, Mannich, and Michael maneuvers, a growing interest is witnessed in those domino reactions where several reactive events are triggered, which comprise one or more vinylogous additions, eventually leading to cyclic or polycyclic products in an efficient and economic manner.

As already noted for other electron-rich donor classes (vinylogous aldehydes, ketones, and esters), the direct activation modalities of unsaturated amide and lactam precursors, characterized by in situ formation of di- or polyenolates, are highly represented during the period surveyed in this commentary, while the strategies based on indirect activation of these matrices (e.g., via silyl enol ether preformation) are limited in number but still used in target-oriented synthesis.

**Figure 7:**
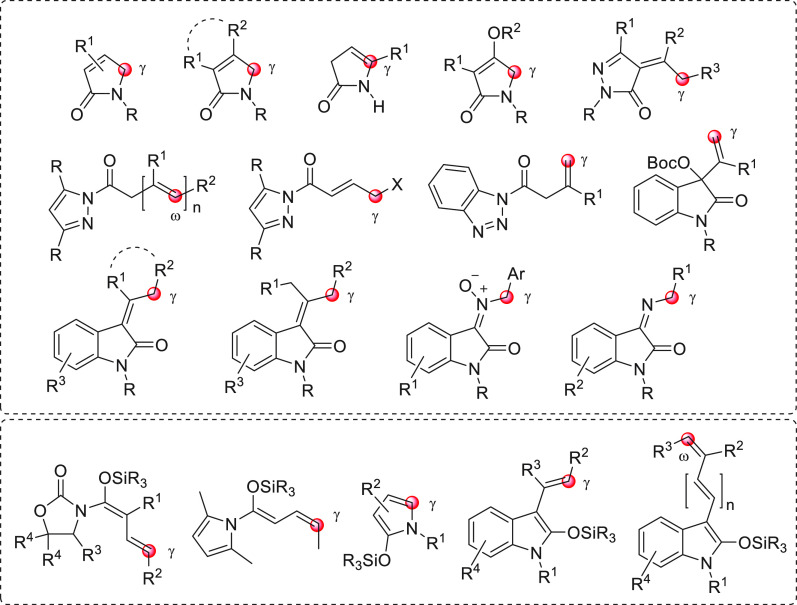
Panel of acyclic and cyclic amide pronucleophiles used in direct procedures (above) and preformed nucleophiles (silyl ketene *N*,*O*-acetals) exploited in indirect procedures (below). Red circles denote the reactive (pro)nucleophilic carbon site.

According to the main organization of this review article, acyclic and cyclic pronucleophiles displayed in Figure 7 will be sequentially discussed, where the electronic effects of the amide carbonyl system are transmitted to the remote position through conjugated double (or triple) bonds which either belong to acyclic chains or are inserted, at least partially, into a ring system.

### 6.1 Additions to C=O Bonds

#### 6.1.1 Direct Procedures

The direct catalytic asymmetric addition of vinylogous amide donors to carbonyl electrophiles (VAR) has emerged as a powerful and widely exploited strategy to assemble δ-hydroxy-α,β-unsaturated amides that represent advanced synthons in the preparation of natural product mimetics and therapeutics. The direct use of pronucleophilic molecular entities avoiding preactivation and protecting group installation processes is highly desirable for convenience and atom economy and has accordingly become the most applied strategy in catalytic asymmetric vinylogous reactions; for this reason, the studies focused on direct activation modalities of the amide precursors are much more frequent in the literature and, among these, those exploiting heterocyclic pronucleophiles have attracted the major interest.

##### 6.1.1.1 Acyclic Pronucleophiles

**Scheme 200:**
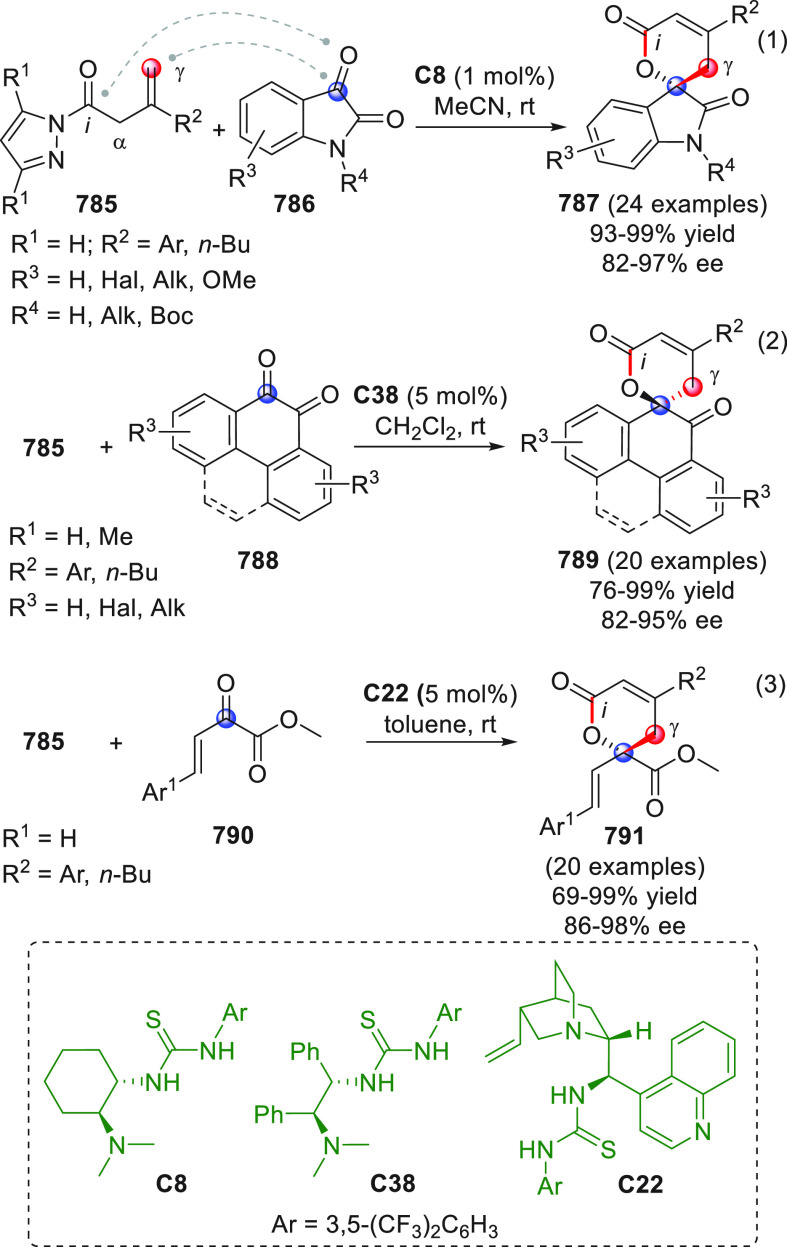


The use of simple acyclic amide pronucleophiles in direct asymmetric aldol maneuvers is of great interest, even though poorly represented in 2010–2018, likely due to the intrinsic low reactivity of the generated dienolates. In most cases, these reactions constitute the first step of a reaction cascade leading to cycloaddition products. In 2014, based on previous investigations concerning the use of pyrazoleamides as donor species in Michael and Mannich addition reactions, Sha, Wu, and colleagues developed the first direct, enantioselective vinylogous aldol addition of allyl pyrazoleamides to isatins, as the initial step of a cyclization reaction cascade producing chiral spirocyclic dihydropyran-2-ones (Scheme 200, eq 1).^514^ β,γ-Unsaturated deconjugated amides **785** were chosen as precursors of the vinylogous enolates, since the tertiary amine catalysis was ineffective in promoting γ-enolization of the corresponding α,β-unsaturated counterparts. The pyrazoleamide function within pronucleophiles **785** was selected as an activating/directing group for enhancing stereocontrol, as well as a good leaving group enabling further transformations (in the event, the spirocyclization step). Aiming at the synthesis of alkaloidal bioactive compounds, isatins **786** were selected as electrophilic components to assemble chiral spiro-oxindole scaffolds. After a careful exploration of the tertiary amine-thiourea bifunctional organocatalysts and the reaction conditions, the authors succeeded in the construction of various chiral spirocyclic oxindole dihydropyranones **787** in excellent yields and good-to-excellent enantioselectivities, by using as low as 1 mol % of the Takemoto catalyst **C8**. The absolute *S*-configuration of the newly formed quaternary stereocenter was attributed on the basis of the literature optical rotation values, whereas the authors did not investigate the role of the organocatalyst in the formation of the sole quaternary stereocenter.

The same β,γ-unsaturated pyrazole amide donors **785** were exploited, two years later, by the same authors in vinylogous aldol-spirocyclization cascade reactions with *o*-quinone acceptors **788**, in the presence of a chiral diamine-based thiourea organocatalyst, to produce spirocyclic pyranones **789** (Scheme 200, eq 2).^515^ The initial screening of the chiral promoter led to the selection of catalyst **C38**, whose H-bonding donor ability was judged crucial for the overall enantioselectivity of the process. The substrate scope was explored by reacting variously β-aryl (and β-alkyl) substituted allyl pyrazoleamides **785** with different 1,2-diketones **788** in the presence of **C38** (5 mol %): spirocyclic dihydropyran-2-ones **789** were obtained in high yields (76–99%) and enantioselectivities ranging from 82 to 95%. Control experiments confirmed the role of the tertiary amine in the initial formation of the dienolate intermediate and the selective γ-reactivity of pyrazoleamides.

In their continuing effort toward the synthesis of chiral 5,6-dihydropyran-2-ones, Wu and colleagues envisaged that the pyrazoleamides of type **785** could act, once again, as γ-pronucleophiles in the asymmetric VAR-cyclization sequence with acyclic α-keto esters **790** to form chiral dihydropyranones **791** in a highly chemo- and enantioselective manner (Scheme 200, eq 3).^516^ The use of various allyl pyrazoleamides with a variety of γ-aryl α-keto esters in the presence of organocatalyst **C22** (5 mol %) ensured the preparation of chiral cycloadducts **791** bearing quaternary stereocenters in high yields and enantioselectivities.

##### 6.1.1.2 Cyclic Pronucleophiles

The use of α,β-unsaturated γ-butyrolactam pronucleophiles as donors in vinylogous aldol reactions, in principle, offers limitless possibilities to generate chiral highly functionalized nitrogen-containing heterocycles; *N*-Boc pyrrolinone, for example, has been widely employed as the immediate precursor of the corresponding pyrrole silyl dienolates, which served in a huge number of vinylogous indirect aldol, Mannich, and Michael transformations (vide infra). During the 2010–2018 period, however, the use of this simple nitrogen heterocycle in direct versions of such pivotal addition maneuvers has been marginally explored and just one contribution concerning the exploitation of pyrrolinone dienolates was found; on the other hand, contributions exploiting direct procedures based on heterocyclic scaffolds as 3-alkylidene oxindoles as starting materials are much more represented.

**Scheme 201:**
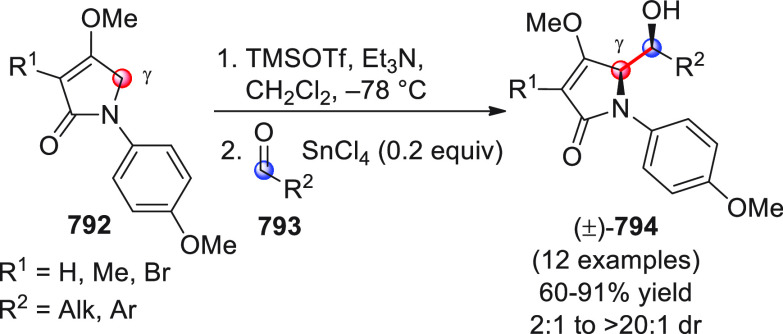


In 2014, Pettus and colleagues proposed a general diastereoselective metal-catalyzed VAR of tetramate pronucleophiles with various aldehydes (Scheme 201).^517^ Since the base-catalyzed aldol addition of tetramate-derived dienolates (obtained from **792**) gave unsatisfactory results in terms of yield and stereoselectivity, the authors set up a one pot-two step Mukaiyama-type addition protocol by treating tetramates **792** with the Et_3_N/TMSOTf system, to in situ generate silicon dienolates, followed by addition of the aldehyde acceptors **793** and the catalytic Lewis acid (SnCl_4_). *Syn*-configured aldol adducts **794** were obtained with moderate-to-good efficiency and diastereoselectivity. An open-chain transition state was hypothesized by the authors, in which the carbonyl oxygen and the aryl-substituted tetramate nitrogen were both involved in the metal-chelation, accounting for the *syn*-selective outcome of the process. Moreover, the authors observed that the nature of the R^1^ substituent markedly affected both the efficiency and stereoselectivity of the process, with bromo-derivatives affording best results, and that the reaction was quite general with respect to the aldehyde structure.

**Scheme 202:**
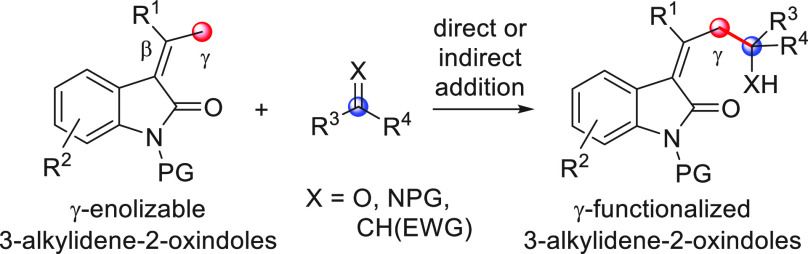


Oxindole structures are common heterocyclic motives shared by many alkaloidal natural compounds and synthetic bioactive compounds. In particular, 3-alkylidene-2-oxindole frameworks (Scheme 202) have been the subject of great interest in the search of asymmetric synthetic methodologies to build oxindole and spirooxindole derivatives;^518,519^ however, if the electrophilic reactivity at the β-position has been widely explored in many examples of Michael addition processes, the vinylogous pronucleophilic character of these scaffolds, when a γ-enolizable site is present, has drawn attention only in recent years.

The first to realize the considerable potential of 3-alkylidene oxindoles as multifunctional γ-enolizable architectures was the group of Curti, Rassu, and Zanardi, who in 2012 exploited these scaffolds as vinylogous donors in direct organocatalyzed Michael reactions, as well as in indirect aldol and Mannich additions, where the corresponding silyl ketene *N*,*O*-acetals acted as vinylogous nucleophiles (vide infra).

**Scheme 203:**
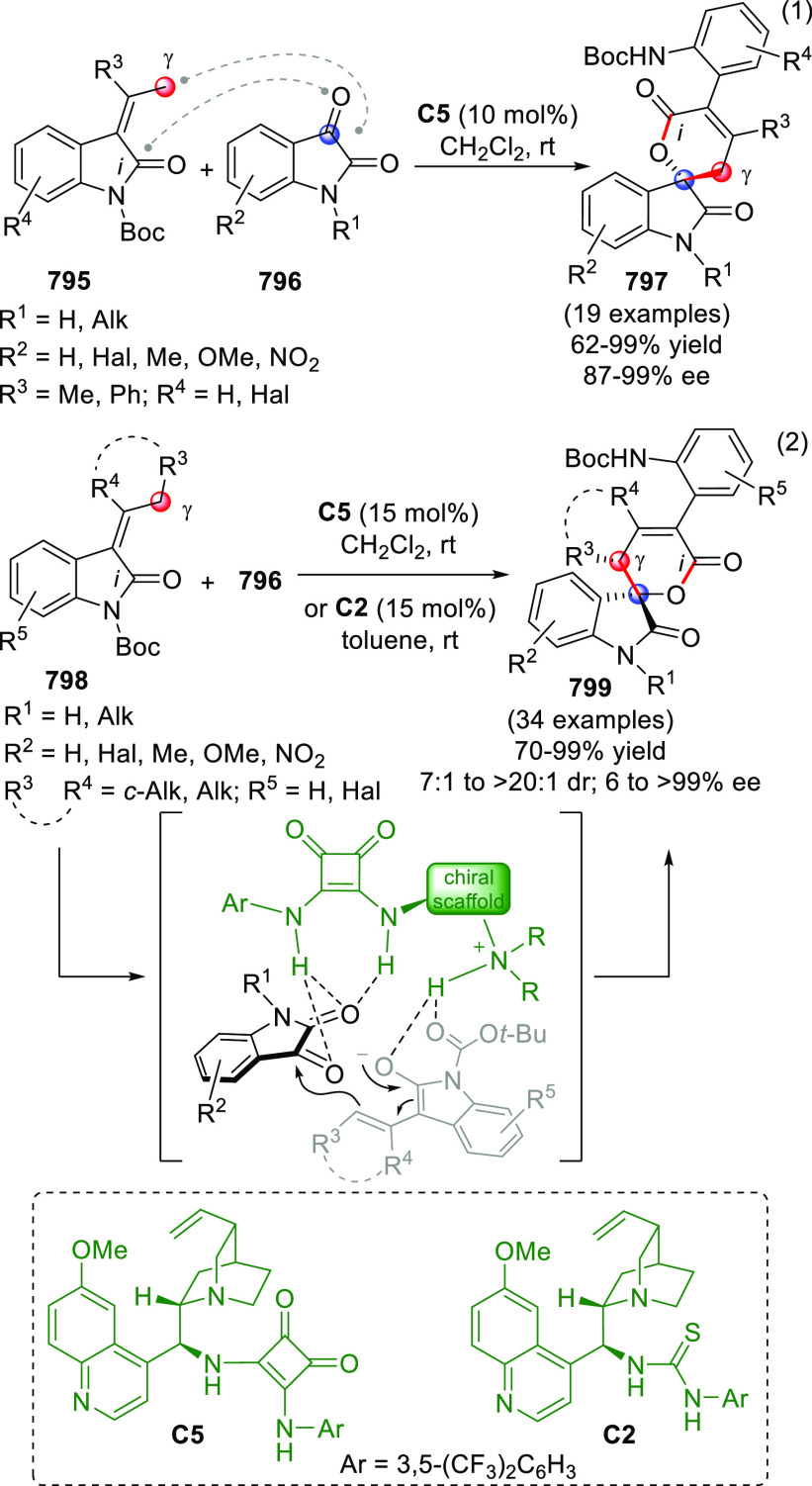


A few years later, in 2015, Han and Chang reported on the use of 3-alkylidene-2-oxindoles of type **795** as pronucleophiles in direct addition to isatins **796** (Scheme 203, eq 1).^520^ In the event, the aldol-cyclization cascade was orchestrated by the bifunctional squaramide catalyst **C5**, producing the spirocyclic dihydropyran-2-ones **797** in moderate to excellent yields and fair enantioselectivities. It was hypothesized that, after the initial γ-deprotonation of the alkylidene oxindole moiety giving an *s-cis* dienolate, the addition of the nucleophile to the isatin carbonyl resulted in the formation of an aldolate, which in turn reacted with the oxindole carbonyl, leading to the unexpected opening of the lactam ring ultimately producing unsaturated lactones **797**.

Expanding the scope of the vinylogous donor to prochiral or cycloalkylidene derivatives **798** (with R^3^, R^4^ corresponding to alkyl groups or belonging to a carbo- or a heterocycle), Chang and collaborators set up a diastereo- and enantioselective version of the aldol-cyclization reaction cascade previously described (Scheme 203, eq 2).^521^ Again, isatins of type **796** were selected as electrophilic counterpart and different cinchona-derived bifunctional organocatalysts were scrutinized. With 3-cycloalkylidene oxindoles **798** as the vinylogous donors, the best performing catalyst was the bifunctional squaramide **C5**, furnishing the bicyclic δ-lactones **799**, bearing two vicinal new stereocenters. On the contrary, the use of 3-pentylidene indolinones **798** (R^3^ = Me, R^4^ = Et) required the deployment of the thiourea **C2** and led to the enantiomeric spirooxindoles *ent*-**799** (not shown). Scheme 203 (eq 2) shows the proposed transition state accounting for the observed configuration of compounds **799**, where the ion pairing between the protonated amine catalyst and the oxindole dienolate would be operative, triggering the attack to the *Re* face of isatin; isatin, in turn, would be activated by the H-bonding network displayed by the squaramide moiety. The authors did not explain the reason for the enantioselectivity switch observed with thiourea **C2** and concluded that other possible activation modes could not be excluded since no detailed mechanistic studies entailing DFT calculations were available.

**Scheme 204:**
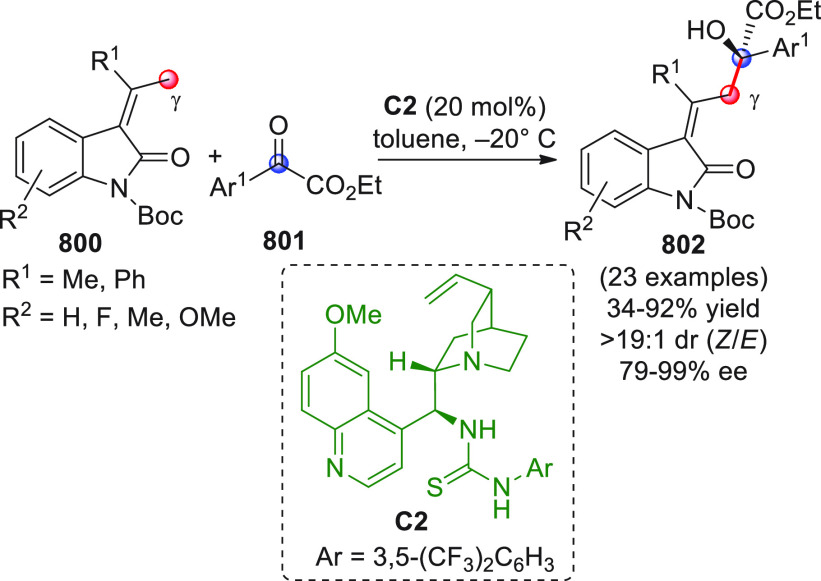


In 2017, Singh and colleagues put in place an asymmetric aldol procedure exploiting 3-alkylidene oxindoles **800** as vinylogous pronucleophiles and α-keto esters **801** as electrophilic counterparts (Scheme 204).^522^ The authors hypothesized (and proved) that the cinchona-derived thiourea bifunctional organocatalyst **C2** could be able, at the same time, to activate and orient the reactant partners leading to α,β-unsaturated aldol adducts **802** in a stereocontrolled manner. Among the tested organocatalysts, the thiourea **C2** gave the best results, affording *Z*-configured δ-hydroxy esters **802** in yields ranging from 34 to 92% and enantiomeric excesses from 79 to 99%.

**Scheme 205:**
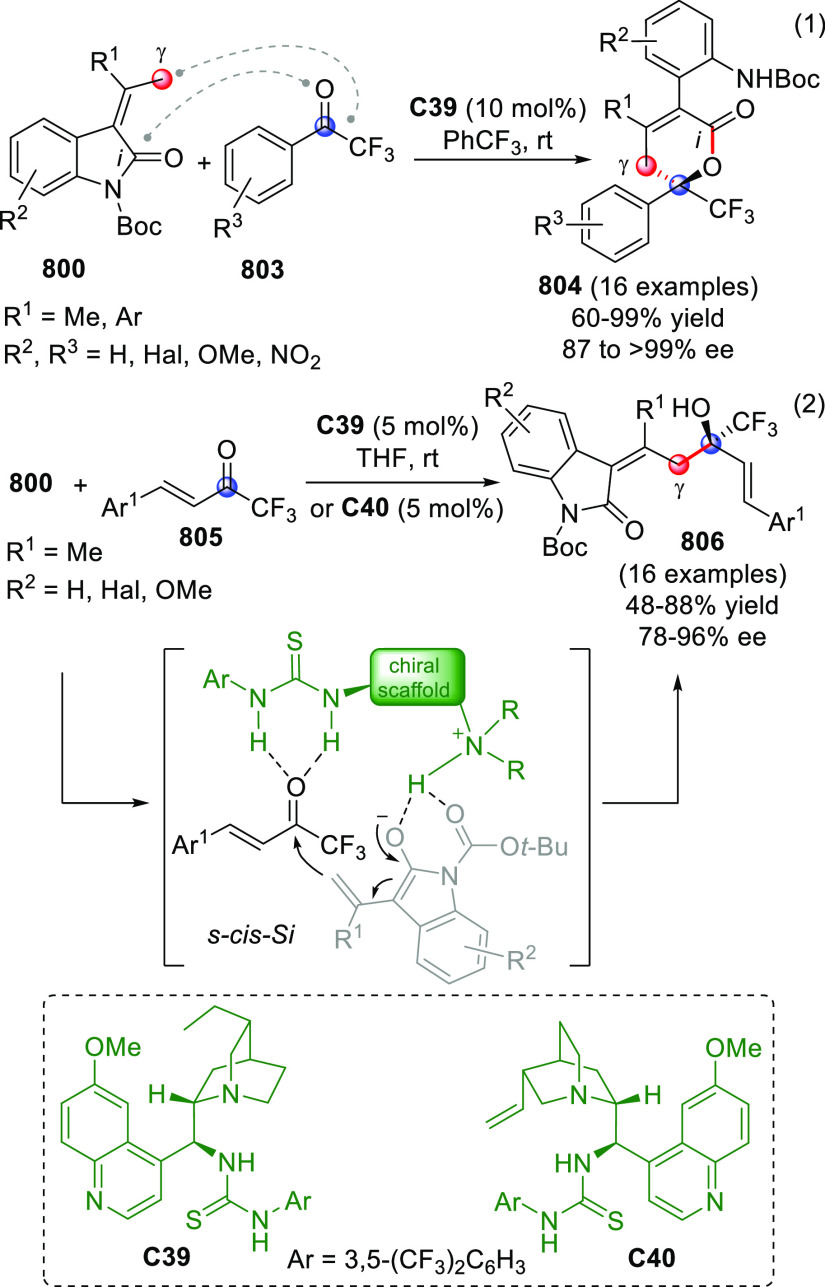


The utility of alkylidene oxindoles as vinylogous pronucleophiles in aldol addition-cyclization sequences is testified by another contribution proposed by Bencivenni and colleagues in 2018 (Scheme 205, eq 1).^523^ The authors reported on the vinylogous aldol-lactonization cascade involving alkylidene oxindoles **800** as donors and activated trifluoromethyl ketones **803** as acceptors in the presence of the bifunctional promoter **C39**. The organocatalytic aldol-lactonization proposed by Bencivenni represents a direct and atom economic strategy to access fluorinated α,β-unsaturated-δ-lactones as an alternative way to the cycloadditive methods based on NHC activation of enals reported by Chi and Song (vide infra). The authors selected the cinchona-derived organocatalyst **C39** (Scheme 205, eq 1) to produce the concomitant γ-deprotonation of the alkylidene moiety to *s-cis* dienolate, the H-bond activation of the ketone, and the directioning of both the reagent partners during the aldol-cycloaddition sequence. The trifluoro-δ-lactones **804** were obtained in moderate to very good yields and excellent enantioselectivities, together with variable amounts of acyclic aldolate adducts (not shown). Assignment of the absolute configuration of the products relied upon TD-DFT calculations of electronic circular dichroism (ECD) spectra and corroborated the transition state proposed by the authors.

The efficacy of alkylidene oxindoles in additions to vinyl trifluoromethyl ketones was also demonstrated by Bencivenni et al. in a recent study that aimed at producing enantioenriched fluorinated allylic alcohols as precursors of molecular entities of biological interest (Scheme 205, eq 2).^524^ In this work, the authors had to face the challenging issues of chemo- and regioselectivity in addition to the stereoselectivity. In fact, when trifluoromethyl vinyl ketones of type **805** are used, both the 1,2- and 1,4-addition products can be in principle formed, and the pronucleophilic γ and γ′ positions within the oxindole **800** could compete with each other leading to a mixture of *E*/*Z* alkenes. The accurate scrutiny of various cinchona-derived catalysts revealed that both thioureas **C39** and **C40** (the quasi-enantiomeric 9-*epi*-quinidine-derived) were efficient in promoting the 1,2-addition of oxindoles **800** to ketones **805** and no traces of 1,4-adducts were found. When **C39** was employed, *R*-configured allylic alcohols **806** were obtained, while the opposite enantiomers *ent*-**806** were accessible by using **C40** catalyst. To explain the remarkable stereocontrol of the process, the authors proposed a possible activation modality featuring the ion-pairing between the quinuclidine nitrogen of **C39** and the oxindole *s-cis* dienolate and the concomitant thiourea H-bond activation with exposure of the ketone *Si* face to the nucleophilic attack (Scheme 205).

#### 6.1.2 Indirect Procedures

The most common indirect procedures based on the use of amide (or lactam) donors entail the activation of the substrates as silicon-based di- or polyenolates before participating in the additive reaction. The past two decades have witnessed an extremely wide use of these Mukaiyama-type silyl derivatives as donors, especially in the vinylogous domain, to embody α,β-unsaturated carbonyl frameworks into linear, carbo- or heterocyclic scaffolds. The successful exploitation of extended silyl *N*,*O*-acetals is also due to their intrinsic propensity to privilege reactions at their remote sites, as demonstrated by many experimental pieces of evidence.^6,34,35^

##### 6.1.2.1 Acyclic Nucleophiles

**Scheme 206:**
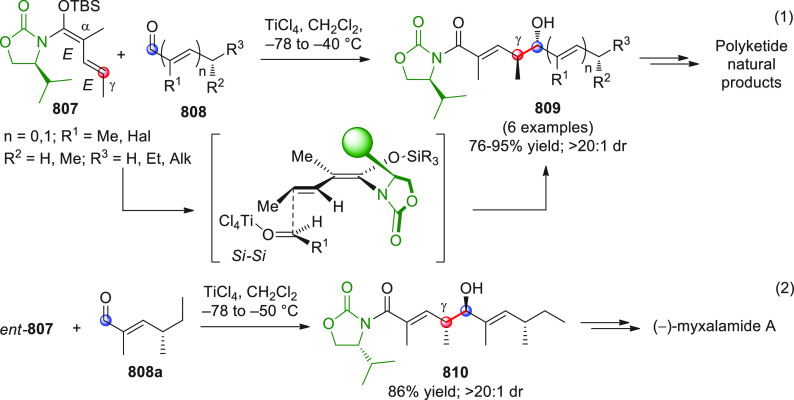


The use of chiral oxazolidinones derived from enantiopure amino acids as the auxiliaries to stereogovern the course of aldol additions (and related Mannich and Michael processes) has a long history starting in the early 1980s thanks to the pioneering works by Evans and co-workers.^525^ In 2004, Kobayashi, and later Hosokawa, successfully translated the concept of auxiliary-guided chirality transfer in the domain of vinylogous Mukaiyama-type aldol reactions, by investigating the preparation and use of vinyl ketene silyl *N*,*O*-acetals of type **807** to be used as vinylogous donors to access relevant, highly substituted polyketide units with a high level of stereocontrol (Scheme 206).^526^ In fact, the enolsilylation of α,β-unsaturated *N*-acyl oxazolidin-2-ones derived from l-valine afforded stable silyl dienolates of type **807**, whose (*E,E*) double bond configuration and the conformational arrangement were ascertained by X-ray analysis and bidimensional NMR spectroscopy. The reaction between vinyl silyl ketene *N*,*O*-acetals **807** and a variety of aldehydes (aliphatic, aromatic, α,β-unsaturated) in the presence of TiCl_4_ efficiently produced *anti*-aldol adducts of type **809** (Scheme 206, eq 1).^526^ In the event, the authors proved that the presence of an α-methyl group in the acyl chain provided stability to the dienolate and was crucial to achieving a high level of stereoselectivity. On the basis of crystallographic and NMR data and energy-minimized conformation calculations, the authors proposed the transition state reported in Scheme 206, where the α-methyl group is disposed *trans* with respect to the oxazolidinone ring which, in turn, is almost perpendicular to the dienol ether plane with the l-valine isopropyl group shielding the upper face of the dienolate donor. This arrangement would likely guide the incoming aldehyde to approach the less hindered bottom face of the donor, leading to 6,7-*anti*-configured δ-hydroxy-α,β-unsaturated adducts of type **809**.

The viability and predictability of the stereochemical outcome of this auxiliary-driven stereoselective VMAR strategy prompted the general exploitation of the Kobayashi reaction in asymmetric synthesis, as highlighted in the several publications appearing until 2010 and discussed in previous accounts.^527,528^

Over the 2010–2018 period, this fortunate trend carried on, and many other researchers besides the Kobayashi group exploited this strategy with remarkable achievements particularly in the field of natural product synthesis. In 2010, Hosokawa et al. exploited this approach to access the first total synthesis of *epi*-cochlioquinone A, a potent inhibitor of ACAT (acyl-CoA:cholesterol acyltransferase) (Scheme 206, eq 1).^529^ The vinyl silyl ketene *N*,*O*-acetal **807** was used as a donor in VMA addition to chiral (2*S*)-2-methyl butanal **808** (n = 0, R^2^ = Me, R^3^ = Et) in the presence of TiCl_4_ as Lewis acid, affording *anti*-aldol **809** in high yield and excellent control in the formation of the new stereocenters.

The viability of reactions exploiting remote asymmetric induction strategies and based on dienolate **807** was fully demonstrated in the convergent total synthesis of the antibiotic (+)-TMC-151C completed by Kobayashi and colleagues in 2011 (not shown).^530^ This compound showed a significant antitumor activity, along with an intriguing polyketide structure featuring three contiguous *anti*-homoallylic alcohol motifs that should be amenable to reiterative VMA additions of chiral oxazolidinones of type **807** to proper aldehydes. The initial attempts to reiterate the auxiliary-driven reactions of silyl dienolate **807** with the aldehyde counterparts in building up the new C–C bonds failed. Thus, the authors decided to adopt a convergent synthetic route entailing the independent synthesis of fragments C1–C6 and C7–C20 (target numbering) both featuring the intended *anti*-homoallylic alcohol motif, followed by their connection at a late stage by a silicon-tethered ring-closing metathesis reaction. In particular, l-valine-derived oxazolidinone **807** served as a donor in both the *anti*-selective VMARs giving the corresponding precursors of the C1–C6 and C7–C20 fragments of (+)-TMC-151C.

The vinyl silyl ketene *N*,*O*-acetal deriving from unnatural d-valine *ent-***807** was instead selected by Kobayashi et al. to accomplish the synthesis of the left-hand fragment (C9–C18) of the polyene antibiotic (−)-myxalamide A (Scheme 206, eq 2).^531^ The reaction of *ent-***807** with α,β-unsaturated aldehyde **808a** promoted by titanium tetrachloride efficiently produced the *anti*-aldol **810** showing the expected *anti*-configuration at the newly formed stereocenters. Moreover, the authors observed that the reaction in which a catalytic amount of water (10 mol %) was added not only produced similar yield and stereoselectivity, but it also was markedly accelerated.

Going on in investigating the exploitation of silyl ketene acetals of type **807** in asymmetric VMAR, Kobayashi et al. explored the use of α-haloenals **808** (Scheme 206, n = 1, R^1^ = Hal, R^2^ = R^3^ = H) as acceptors to overcome the low reactivity of α,β-unsaturated aldehydes such as tiglic aldehyde (**808**, n = 1, R^1^ = Me, R^2^ = R^3^ = H).^532^ According to the established protocol, dienolate **807** reacted with two equivalents of haloenals **808** and TiCl_4_ affording *anti*-aldols of type **809** endowed with versatile δ-hydroxy-ε-halo functionalities along with two versatile double bonds. As previously demonstrated, even in this case, high yields could be obtained by prolonging reaction times or by adding a catalytic amount of water.^533^

The use of tiglic aldehyde as an acceptor in the stereoselective VMAR with silyl ketene acetal **807** was also exploited by Hosokawa and colleagues in 2013, during the synthesis of septoriamycin A, an antimalarial agent of microbial origin (not shown).^534^ As proof of the concept of the so-called wide range stereocontrol, the authors realized the divergent synthesis of four 2,4,6-trimethyloctanoate diastereoisomers by combining the initial Kobayashi reaction, that installed the chirality into the central core of the adducts with a stereoselective reduction, that transferred the chirality to the “surrounding” double bonds.

The chiral vinyl silyl ketene acetal derived from d-valine *ent-***807** was selected by Prusov and collaborators in 2012 as donor component in VMARs to accomplish the synthesis, and hence the structural elucidation, of eliamid, a secondary metabolite from myxobacteria with antifungal properties (not shown).^535^ Hoecker and Gademann in 2013 proposed the enantioselective synthesis of two natural products structurally related to piericidins, JBIR-02 and Mer-A2026B.^536^ Here the installation of the two and sole C9 and C10 stereocenters within the polyenic side-chain relied on the *anti*-selective VMAR of *ent-***807** with β-bromo-acrylaldehyde in the presence of TiCl_4_ (not shown). The crystallographic analysis of the intermediate adduct allowed the assignment of the absolute configurations of both the natural and the synthetic samples. More recently, Ohashi and Hosokawa carried out the Kobayashi reaction with compound **807** and acetaldehyde as the opening move in the synthesis of stoloniferol B and penicitol A (not shown).^537^ Also in this case, the VMA addition leading to *anti*-configured products ensured the efficient construction of the stereocenters within the targeted lactone and allowed the reassignment of the structure of the analogue fusaraisochromanone.

**Scheme 207:**
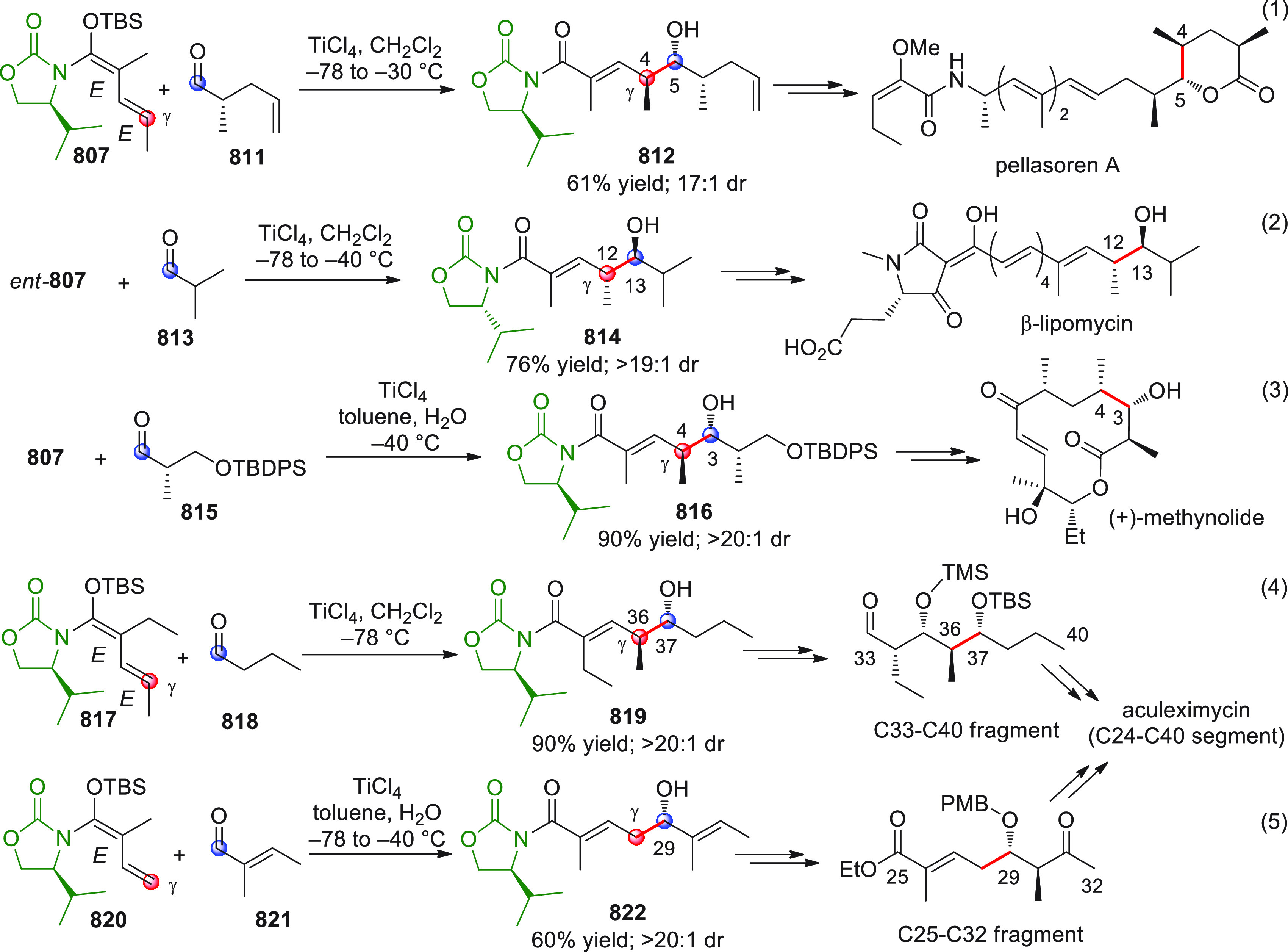


In the field of the total synthesis, structure elucidation, and biological evaluation of natural products, Kalesse and collaborators have long been active researchers, contributing to the success of the Kobayashi reactions based on the use of chiral oxazolidinones as vinylogous pronucleophiles. In 2012, they proposed the first total synthesis of pellasoren A with the aim of validating the stereochemical disposition of this natural product and establishing a model for its biosynthesis (Scheme 207, eq 1).^538^ The VMAR of (*E,E*) vinyl silyl ketene acetal **807** with chiral aldehyde acceptor **811** under optimized conditions was the key maneuver to install the *anti*-disposed C4–C5 stereocenters (target numbering) featuring the lactone moiety within the target compound.

Along this line, Hartmann and Kalesse embarked on the synthesis of the antibiotic β-lipomycin with the aim of fully validating the C12 and C13 configurations predicted by statistical methods (Scheme 207, eq 2).^539^ In the event, a Kobayashi reaction between silyl ketene acetal *ent-***807** and isobutyraldehyde (**813**) was envisaged, providing the expected (12*R*,13*S*)-configured *anti*-aldol adduct **814** in good yield and stereoselectivity. As a whole, the proposed route not only provided access to the natural β-lipomycin product and its analogues, but it also assessed the proposed statistical method as a reliable tool in predicting the absolute configurations of methyl branches and secondary alcohols within modular polyketide carbon chains.

The total synthesis of (+)-methynolide recently proposed by the Kobayashi group, once again, was grounded on the exploitation of the well-known acetal **807** in stereoselective VMAR to β-silyloxy-substituted aldehyde **815** (Scheme 207, eq 3).^540^ In particular, the bulky silyl protecting group (preventing Ti-chelation), along with the use of toluene as a solvent, and the addition of catalytic water were required to obtain good yield (90%) of the desired (3*S*,4*S*)-*anti*-adduct **816** in >20:1 diastereomeric ratio, even though with very long reaction times (4.5 days).

Finally, the stereoselective synthesis of the C24–C40 segment of antibiotic aculeximycin, proposed by Hosokawa in 2015, was likewise set on the remote asymmetric induction strategy (Scheme 207, eqs 4 and 5).^541^ The authors chose to adopt a highly convergent approach (linking the two C33–C40 and C25–C32 fragments), in which the stereocontrolled formation of the C36, C37, and C29 asymmetric carbons (target numbering) relied on TiCl_4_-promoted VMARs of chiralized acetals **817** and **820** with proper aldehydes (**818** and **821**, respectively) under the usual Kobayashi reaction conditions; all the remaining stereocenters in the polyketide C24–C40 chain were generated exploiting substrate-controlled stereoselective reactions.

The utility of γ-methyl vinyl silyl ketene *N*,*O*-acetals of type **807** in *anti*-selective VMARs has been demonstrated in the many synthetic applications discussed so far. The development of complementary *syn*-selective VMARs employing analogous chiral oxazolidinone silyl dienolates greatly broadens the potential of this strategy in natural products synthesis. *Syn*-selective VMARs were first introduced by Kobayashi in 2009, who devised a strategy according to which a switch of facial selectivity was observed for α-heteroatom-substituted aldehydes in the TiCl_4_-promoted reactions of chiral oxazolidinone silyl dienolates.^542^

**Scheme 208:**
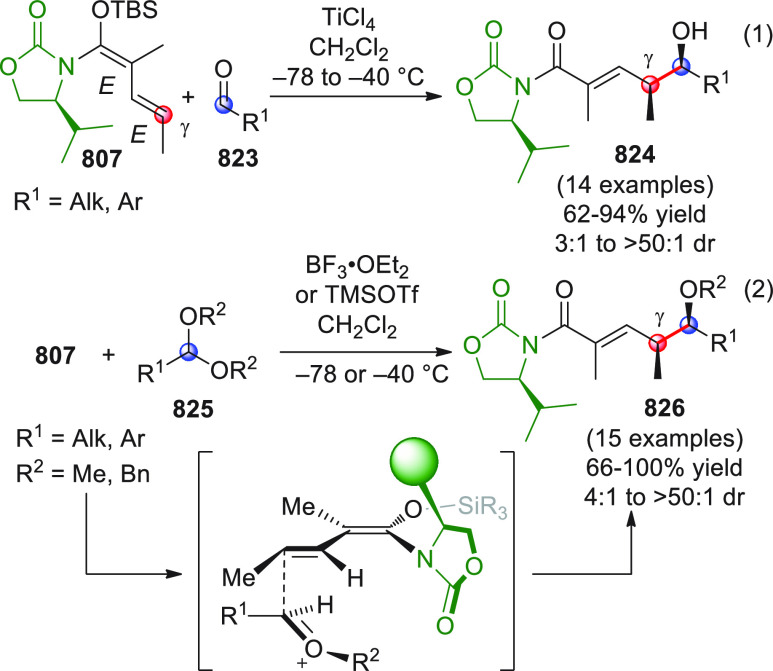


Further on in 2012, the Hosokawa group demonstrated that the stereochemical outcome of the Kobayashi reaction could depend on the amount of the added Lewis acid (Scheme 208, eq 1).^543^ In fact, the reactions employing (*E,E*)-vinyl silyl ketene *N*,*O*-acetal **807** (1.5 equiv) and TiCl_4_ (4 equiv) with a variety of both aromatic and aliphatic aldehydes **823** led to *syn*-aldol adducts **824** with an unexpected (and unexplained) switch of stereoselectivity. Although the reaction proceeded slowly with γ-oxy-aldehydes, *syn*-selectivity was observed in all cases, and aldols **824** were obtained in good to high yields and excellent levels of stereocontrol.

The utility of this methodology was demonstrated by Kanoh and colleagues during the synthesis of the C1–C18 macrolactone fragment of FD-891, a microbial macrolide that was reported to show remarkable antitumor activity.^544^ The authors exploited the VMA reaction between the enantiomer of **807** and a seven-carbon aldehyde (not shown) in the presence of an excess (5 equiv) of TiCl_4_ to form the C6–C7 bond (target numbering) and the corresponding stereocenters in high yield (95%). Unfortunately, the stereoselectivity of the aldol step was not reported, nor discussed by the authors.

The possibility to selectively access in a diastereodivergent manner to either *anti*- or *syn*-aldol products by simply varying the relative amounts of the same starting silyl ketene acetal and Lewis acid with respect to the aldehyde counterpart was fully demonstrated by Poulsen et al. during the studies on the synthesis of the enantiomer of cyclodepsipeptide BE-43547 A_1_.^545^ Starting from the same couple of substrates, a (*Z,E*)-configured vinyl silyl ketene *N*,*O*-acetal derived from l-valine and a suitable 13 carbon-long aldehyde, the reaction performed with TiCl_4_ (1 equiv) gave the corresponding *anti*-aldol in 87% yield and >20:1 diastereomeric ratio, while the reaction performed with an excess of the Lewis acid (4 equiv) gave the *syn*-aldol congener in 75% yield and >40:1 stereoselectivity, thus opening the way to the complete structural elucidation of the compounds belonging to the family of BE-43547 anticancer agents.

During their studies on remote asymmetric induction reactions, Hosokawa and colleagues discovered that the VMAR of the well-known **807** with acetals of type **825** in the presence of stoichiometric quantities of Lewis acid (BF_3_ etherate or TMSOTf) at different temperatures produced protected *syn*-aldol adducts **826** (Scheme 208, eq 2).^546^ The reactions involving acetals derived from aromatic and α,β-unsaturated aldehydes afforded the most efficient and stereoselective results, as compared to the reactions employing acetals from saturated aldehydes. This procedure turned out to be suitable for the one-pot conversion of aldehydes into the corresponding benzyl-protected *syn*-adducts (**826**, R^2^ = Bn), without erosion of yield and stereocontrol, and it was realized by adding dienolate **807** and TMSOTf (1 equiv) to a preformed mixture of the aldehyde of choice, BnOTMS and catalytic TMSOTf. According to the obtained results, the authors supposed that the reaction proceeded via an oxocarbenium ion through the preferred open-chain transition state reported in Scheme 208. Here, the largest R^1^ group of the acetal should be directed far away from the donor dienyl chain to minimize steric repulsion, while the oxonium hydrogen atom should point to the crowded area near the α-methyl group of the dienolate, thus exposing the oxonium *Re* face to the *Si* face of the donor. This transition state could also account for the variable selectivity observed depending on the bulkiness of the R^2^ protecting group.

**Scheme 209:**
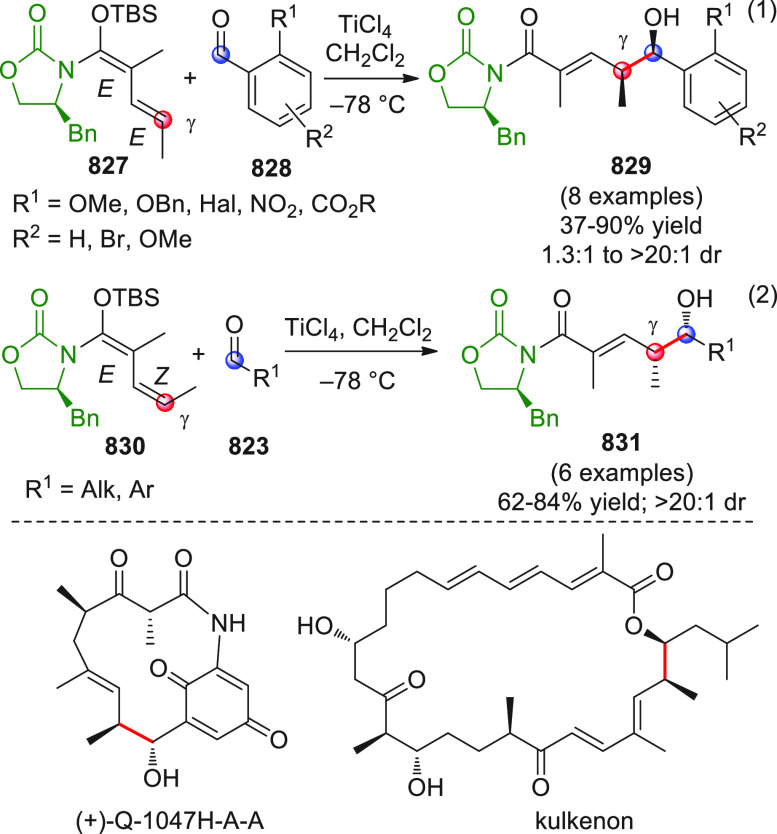


Paralleling the chemistry already disclosed by Kobayashi concerning the *syn*-selective VMARs of vinyl silyl ketene acetals with α-heteroatom-substituted aldehydes,^542^ Chen and Yang established an efficient protocol based on the exploitation of vinyl silyl ketene acetals **827** derived from l-phenylalanine and *ortho*-substituted aromatic aldehydes **828** (Scheme 209, eq 1).^547,548^ The authors proved that *syn*-aldol adducts **829** could be stereoselectively obtained via a chelation-controlled VMAR and applied the protocol to the synthesis (and structural elucidation) of both the potent immunosuppressant NFAT-68 and the ansamacrolactam (+)-Q-1047H-A-A (Scheme 209).

Another example in which chelation-controlled *syn*-selective VMAR was successfully applied to the synthesis of nature-inspired bioactive compounds was described by Jürjens and Kirschning, who exploited the silyl dienol ether **827**, a β-alkoxy-aldehyde, and Ti(O*i*-Pr)Cl_3_ to realize the synthesis of a cytotoxic ansamycin hybrid (not shown).^549^

In the field of natural product synthesis, a relevant contribution is due to Symkenberg and Kalesse, who investigated the use of chiral (*E*,*Z*)-vinyl silyl ketene *N*,*O*-acetal **830** under the Kobayashi reaction conditions to obtain *syn*-adducts **831** (showing opposite stereochemistry at the new stereocenters with respect to compounds **829**) in high yields and remarkable stereoselectivity (Scheme 209, eq 2).^550^ The authors successfully applied this protocol to the total synthesis of kulkenon (Scheme 209, bottom) and could revise and firmly assign the stereostructure of this polyketide macrolactone.^551^

The vinylogous aldol methodology leading to *syn*-configured polyketide synthons was also adopted by Dudley and collaborators to perform the enantioselective high-yielding construction of the C19–C20 bond within the side chain of palmerolide A (not shown).^552^

**Scheme 210:**
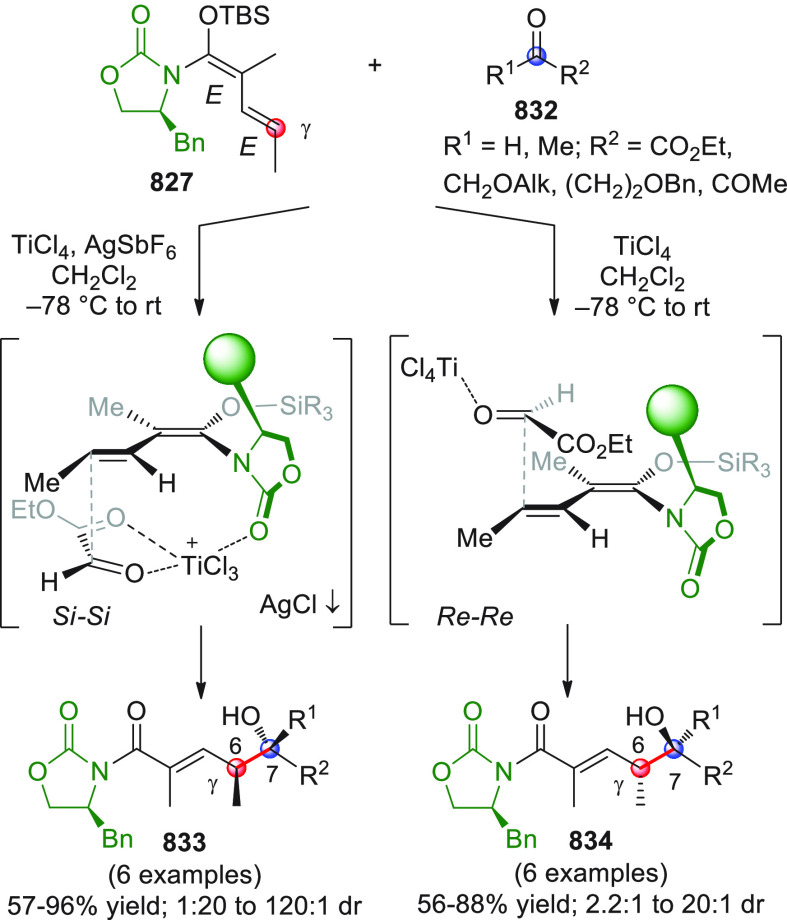


In 2010, Chen, Yang, et al. reported that the facial selectivity leading to *syn*-adducts in the Ti-mediated VMAR with dienolates of type **827** and chelating carbonyl acceptors **832** could be reversed by the addition of silver salts (Scheme 210).^553^ In fact, if the reaction was conducted with glyoxylate **832** (R^1^ = H, R^2^ = CO_2_Et) in the presence of stoichiometric quantities of TiCl_4_ and AgSbF_6_, (6*S*,7*S*)-configured *anti*-adduct **833** was produced in high yield and excellent stereocontrol. The postulated transition state is reported in Scheme 210 (left), where the presence of the silver cation likely allows the formation of a hexacoordinated complex between TiCl_3_^+^, the aldehyde, and the oxazolidinone carbonyl, favoring the *Si*-*Si* approach; on the other hand, in the absence of Ag^+^, the TiCl_4_–aldehyde complex could approach the diene according to the *Re*-*Re* trajectory, giving (6*R*,7*R*)-configured *anti*-adducts **834** (Scheme 210, right).

**Scheme 211:**
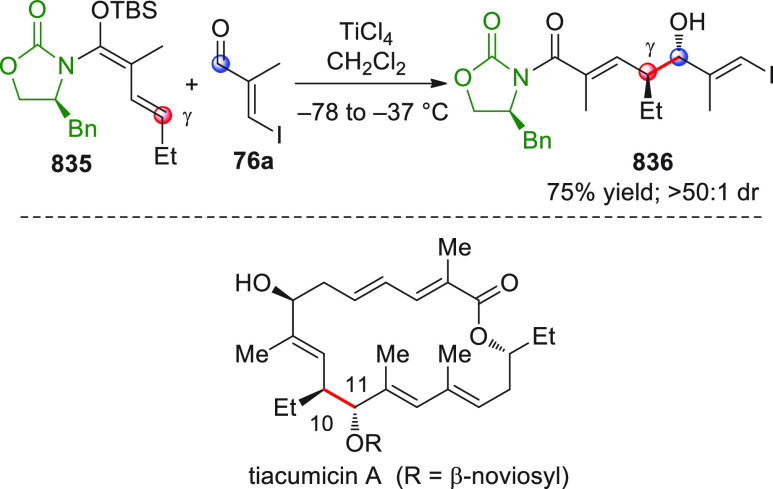


The remote asymmetric induction-based reactions employing phenylalanine-derived silyl dienolates **835** under Kobayashi conditions were adopted by Gademann and colleagues to advance the synthesis toward the macrolide antibiotic fidaxomicin, used in the treatment of resistant forms of tuberculosis, as well as an analogue, tiacumicin A (Scheme 211).^554,555^ In both cases, the convergent approach entailed the construction of the macrolactone core of the targets by joining the previously synthesized polyketide fragments. In the event, the issue of the C10–C11 bond construction (and implementation of the related stereocenters) was solved by employing the chiral vinyl silyl ketene acetal **835** that reacted with vinyl aldehyde **76a**, affording *anti*-aldol **836** with excellent levels of stereocontrol. Then, the C10–C11 *syn*-configuration, required in the target, was obtained by a Mitsunobu reaction involving the reversal of configuration at C11.

In 2013, the Nagorny^556^ and Kuwahara^557^ research groups simultaneously and independently worked on the synthesis of lactimidomycin, a polyketide antibiotic showing antitumor and antifungal activities. In both cases, the C9–C10 bond of the side chain of lactimidomycin (not shown) was formed by a VMAR of a suitable vinyl silyl ketene acetal and acetaldehyde under Kobayashi conditions.

The total synthesis of nannocystin A, a 21-membered macrocyclic depsipeptide possessing a potent antitumor activity, was completed in parallel during 2016 by the groups of Ye^558^ and Chen.^559^ Both the research teams faced the construction of the seven carbon-long C5–C11 fragment within the final macrocycle by using VMARs of vinyl silyl ketene acetals deriving from d-amino acids (d-Phe or d-Val) and (*E*)-3-iodo-2-methylacrylaldehyde under usual Kobayashi conditions, to forge the C7–C8 bond and install the C7 stereocenter; moderate yields and stereoselectivities were obtained in all cases (not shown).

**Scheme 212:**
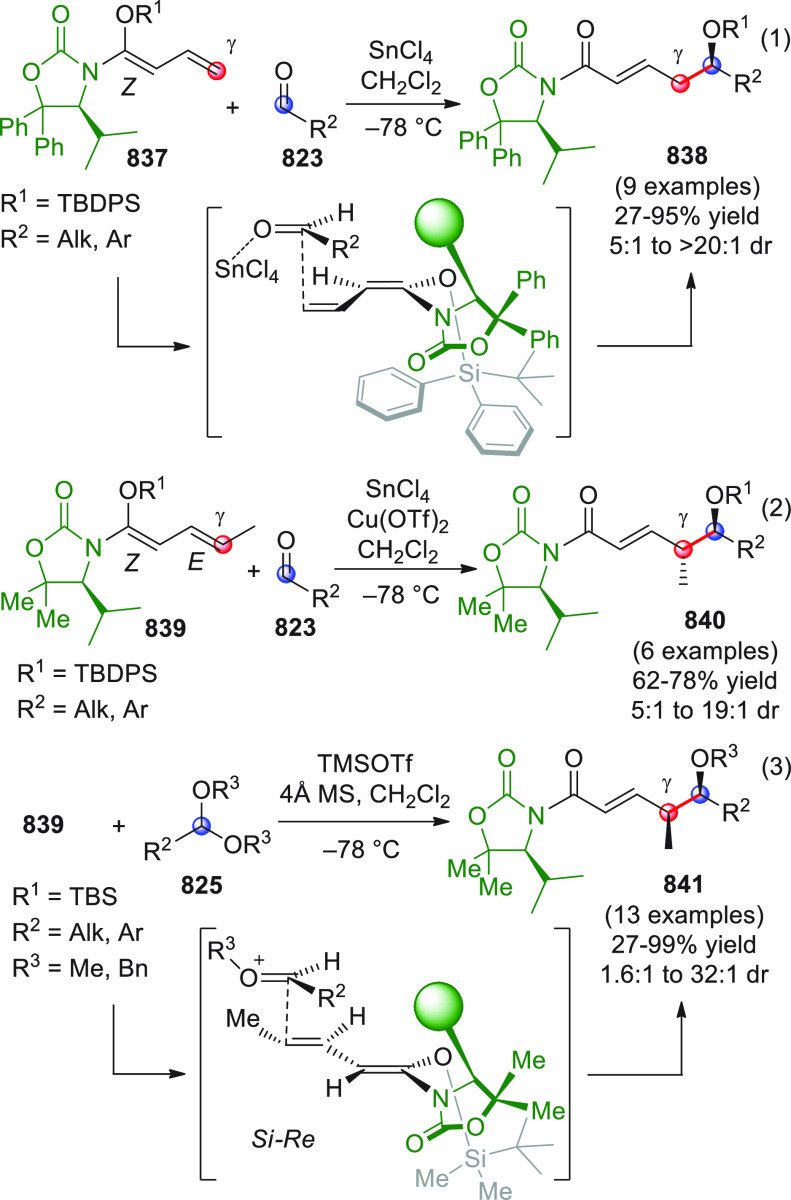


More recently, the Hosokawa group developed *Z*-configured crotonate-type silyl ketene *N*,*O*-acetals of type **837** equipped with a C5′-disubstituted oxazolidinone chiral auxiliary to address the remote asymmetric induction in aldol reactions (Scheme 212).^560^ As observed in previous studies,^526^ when α-methyl-lacking vinyl silyl ketene acetals were used, aldol adducts were obtained in moderate to low stereoselectivities, likely due to the preferred *Z*-configuration of the enolate double bond that keeps the reactive γ-site away from the auxiliary ring. The authors found that the TBDPS protecting group in **837** was crucial to improving the stability of the dienolate and that the oxazolidinone C5′ substituents were responsible for the modification of the overall dienolate conformation. Thus, the VMAR of *Z*-configured dienolate **837** with different aldehydes **823** turned out to be completely stereodivergent, affording either the 5*R*-configured *O*-silylated adducts **838** when SnCl_4_ was used (Scheme 212, eq 1) or the corresponding 5*S*-aldol products (not shown) when the reaction was performed in the presence of BF_3_·OEt_2_. The crystal structure analysis of **837** and NMR NOE experiments revealed that the substituents at C5′ in the oxazolidinone ring were actually efficient in determining the position of the valine isopropyl group and in “pushing down” the silyl group, thus directing one of the phenyl groups of TBDPS below the diene chain. Moreover, these studies revealed that, unlike BF_3_·OEt_2_, the Lewis acid SnCl_4_ promoted the isomerization of the *Z*-dienol ether to the more reactive *E* isomer, accounting for the observed stereodivergency. In order to prove the diene facial selectivity, the (*Z*,*E*)-configured γ-methyl dienolate **839** was exploited in VMARs with aldehydes **823** in the presence of tin tetrachloride and copper triflate (Scheme 212, eq 2). Also in this case, *O*-silylated *anti*-adducts **840** were obtained in good yields and variable stereoselectivity. Based on the overall results, the authors proposed a preferred transition state (Scheme 212, eq 1), where the *Re* face of the aldehyde approaches the upper face of the isomerized *E* diene. Another study by Hosokawa and colleagues analyzed the stereoselective VMA additions of TBS-protected dienol ether **839** to acetals **825** under the guidance of TMSOTf, that resulted in the formation of the δ-alkoxy-substituted *syn*-adducts **841** in good yields and stereoselectivities (Scheme 212, eq 3).^561^ The crystallographic and NMR studies corroborated the hypothesis that the chiral oxazolidinone ring was oriented 40 degrees away with respect to the diene plane and, as previously mentioned, the C5′ substituents could direct the silyl group toward the lower face of the diene (Scheme 212, eq 3); in this way the activated electrophile can approach the upper face of the donor, giving aldols **841** as the major products. The reaction could also be performed in a practical one-pot acetalization-addition procedure without decrease of both the efficiency and the stereocontrol.

The sole example of catalytic asymmetric VMAR involving silyl dienolates deriving from acyclic amides was proposed by Bolm in 2010.^562^ As previously described in the case of furan-based silyloxy dienes (vide supra), the copper aminosulfoximine complex (*S*)-**L13a**/Cu(OTf)_2_ (Scheme 135) was employed as the catalyst in the VMA addition of *N*,*O*-acetals obtained from unsaturated *N*-acyl morpholine to activated α-ketoesters. After optimization of the reaction conditions, the δ-hydroxy-α,β-unsaturated morpholino-amides were obtained, even if only moderate to good yields and no more than 91% enantiomeric excess could be achieved.

##### 6.1.2.2 Cyclic Nucleophiles

As said before, chiral γ-butyrolactams are structural motives widely encountered in many important natural and non-natural bioactive compounds. These nitrogen-containing frameworks also represent important synthetic intermediates in assembling functionality-rich nitrogen heterocyclic structures.

**Scheme 213:**
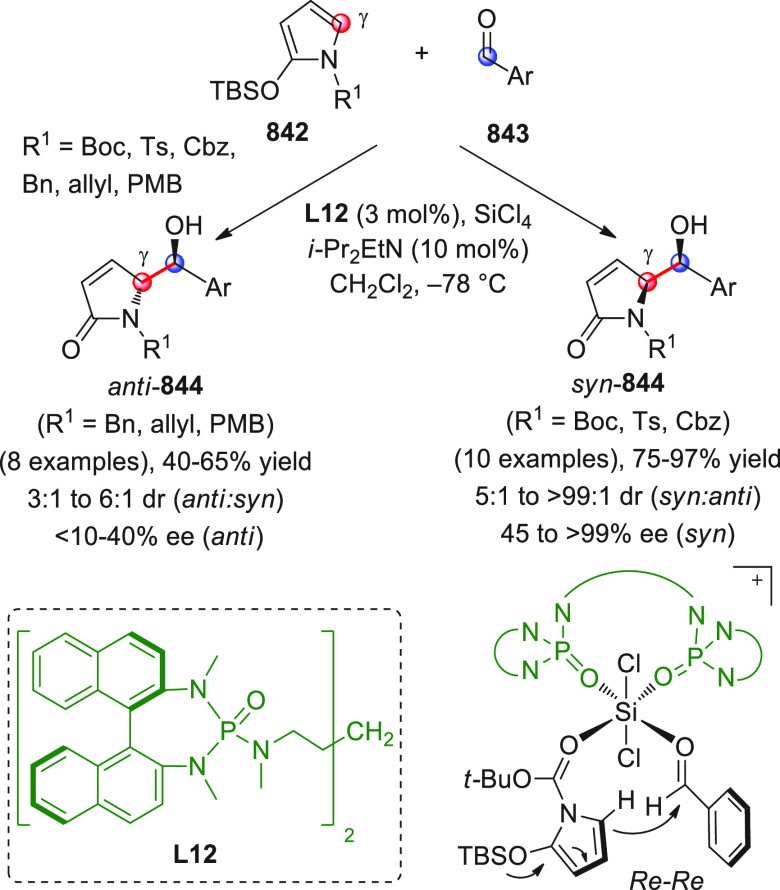


Following their longstanding experience in the use of the popular heterocyclic silyloxy dienes, in 2010 Curti, Zanardi, et al. reported a study focused on the catalytic vinylogous aldol reaction of pyrrole- and furan-based silyl dienolates (the latter being already cited in section 5) with aromatic and heteroaromatic aldehydes.^344^ After a preliminary screening of various metal-based catalyst systems, it was found that the bisphosphoramide **L12**/SiCl_4_ couple was the optimal catalyst system to promote and govern the VMAR between silyloxy pyrroles **842** and aldehydes **843**, producing the expected lactams **844** with high efficiency and stereocontrol (Scheme 213). It is noteworthy that the study revealed that the nature of the heteroatom substituents in the silyloxy diene scaffolds heavily affected the stereochemical reaction outcome; that is, the *syn*-configured adducts *syn-***844** were preferentially obtained with pyrroles bearing electron-withdrawing *N*-protecting groups (Boc, Ts, Cbz), while the use of dienes *N*-substituted with electron-donating groups (Bn, allyl, PMB) (as in the case of furans, vide supra) provided reversal of stereocontrol, giving rise to *anti*-**844** adducts, preferentially. To account for the observed reaction diastereodivergence, dictated by the nature of the heteroatom substituent, a transition state model was proposed (Scheme 213, bottom right). With silicon-coordinating *N*-protecting groups (carbamates and sulfonylamides) able to bind to hypervalent silicon atom of the chiral catalyst, engagement of the *Re* face of the aldehyde carbonyl with the *Re* face of the pyrrole γ-site resulted in the preferential formation of *syn*-disposed (5*S*,1′*S*) adducts; on the contrary, lacking supplementary coordination at silicon, as in the case of Bn, allyl, or PMB *N*-substituents (or oxygen), steric effects prevailed, favoring the involvement of the pyrrole *Si* face with the preferential generation of (5*R*,1′*S*) *anti*-configured structures.

**Scheme 214:**
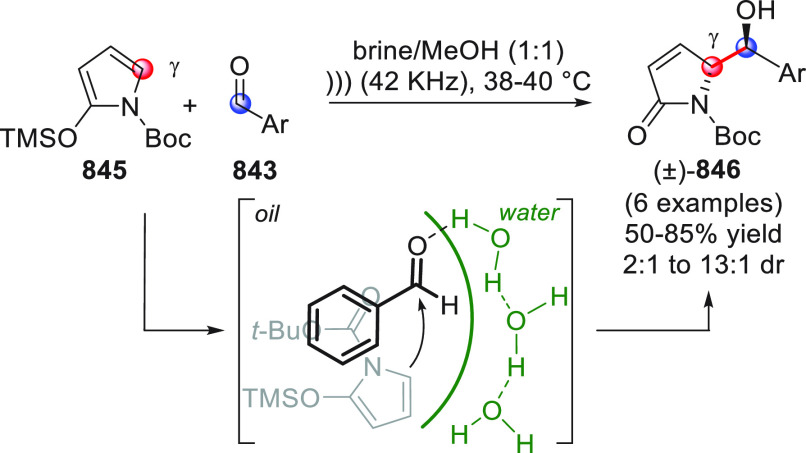


As previously described with furan-based silyl dienolates (see section 5), Curti, Casiraghi, et al. reported the stereoselective VMAR between the pyrrole counterparts and aromatic aldehydes on aqueous media giving equally successful results (Scheme 214).^350^ The uncatalyzed VMAR with silyloxy pyrrole **845** carried out in a mixture of brine and MeOH, under ultrasonic irradiation, was successfully applied to a number of aromatic aldehydes **843** leading to *anti*-configured δ-hydroxy-γ-lactams **846** in good yields, virtually complete γ-site selectivity, and moderate to good diastereoselectivity. However, a remarkable switch of diastereoselectivity was observed when passing from pyrrole (*anti*-selective) to furan silyl dienolates (*syn*-selective), highlighting, once more, how the nature of the heteroatom in the donor moiety critically affects the diastereocontrol of the reaction. On the basis of reports concerning similar *on water* processes, this reaction was postulated to occur at the boundary between water and the dispersed lipophilic phase, with critical H-bonding interactions between water molecules and the carbonyl acceptor, which likely governed the reciprocal position of the reactants in the transition state (Scheme 214); as opposite to furan nucleophiles, here the bulky, lipophilic *N*-*tert*-butoxycarbonyl group is shifted away from the water interface entering the inner space of the reactant droplets, thus reverting the diastereocontrol.

Contrary to the wide exploitation witnessed during the last 20 years of the past century dealing with the use of popular pyrrole silyloxydienes of type **842** or **845** (Schemes 213 and 214) in VMARs to chiral acceptors (according to the so-called chiron approach), in recent years the interest for these type of substrate-controlled stereoselective methodologies has gradually faded, likely due to the concomitant raising of asymmetric catalytic methodologies. Only two examples of target-oriented exploitation of the VMA reaction between silyloxy pyrroles and chiral aldehyde acceptors were found; in the first contribution by Zambrano, Battistini, et al.,^563^ the well-known vinylogous aldol addition of *tert*-butyldimethyl silyloxypyrrole (**842a**, R^1^ = Boc, TBSOP) to d-glyceraldehyde acetonide was chosen as the opening move in a short reaction sequence that led to the asymmetric synthesis of 1-deoxy-7,8-di-*epi*-castanospermine and, at the same time, served in the unambiguous reassignment of the stereostructure of this alkaloid (not shown).

The same VMAR between TBSOP (**842a**) and d-glyceraldehyde was the key step in the asymmetric total synthesis of (+)-*N*-acetyl norloline reported by Huang and colleagues in 2016.^564^ In this instance, the VMAR served to efficiently install three of the four stereogenic centers featuring the target pyrrolizidine alkaloid (not shown).

**Scheme 215:**
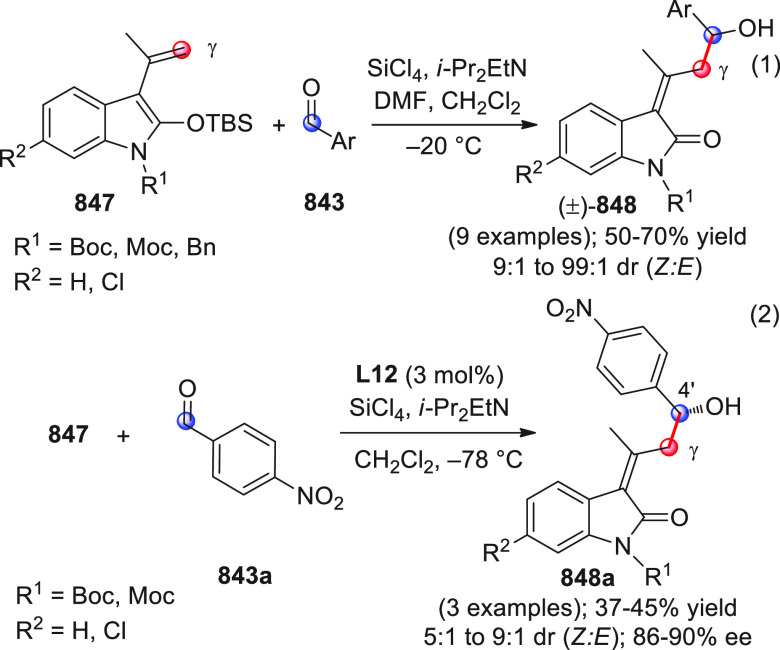


In 2012, Rassu, Curti, Casiraghi, and colleagues reported the very first example of the synthesis of 3-alkenyl-2-silyloxy indoles **847** and their use in asymmetric VMAR with aromatic aldehydes **843** (Scheme 215).^565^ A simple procedure to access *N*,*O*-silyl ketene acetals **847** was developed, using triethylamine and TBSOTf as enolizing-silylating reagents from the corresponding 3-alkylidene oxindoles; then, the addition of stable dienolates **847** to a variety of aromatic aldehydes was explored. Using silicon tetrachloride and diisopropyl ethylamine in CH_2_Cl_2_ in the presence of DMF, the corresponding racemic vinylogous aldol adducts **848** were obtained in moderate yields, with complete γ-site selectivity and excellent diastereoselectivity favoring the *Z*-configured alkenes (Scheme 215, eq 1). During an exploratory trial, the enantioselective version of the process was also discovered, using Denmark’s bisphosphoramide catalyst **L12** in combination with SiCl_4_ (Scheme 215, eq 2). Chiral nonracemic products of type **848a** eventually formed in acceptable yields, with appreciable *Z*-diastereoselectivity and promising enantioselectivity (up to 90% enantiomeric excess). Based on precedents on the use of the **L12**/SiCl_4_ catalyst system in VMARs of enoxysilanes to aromatic aldehydes, the authors hypothesized a catalytic pathway involving the attack of the indole nucleophiles to the *Re* face of the aldehyde carbonyl groups resulting in the preferential formation of 4′*R*-configured adducts.

**Scheme 216:**
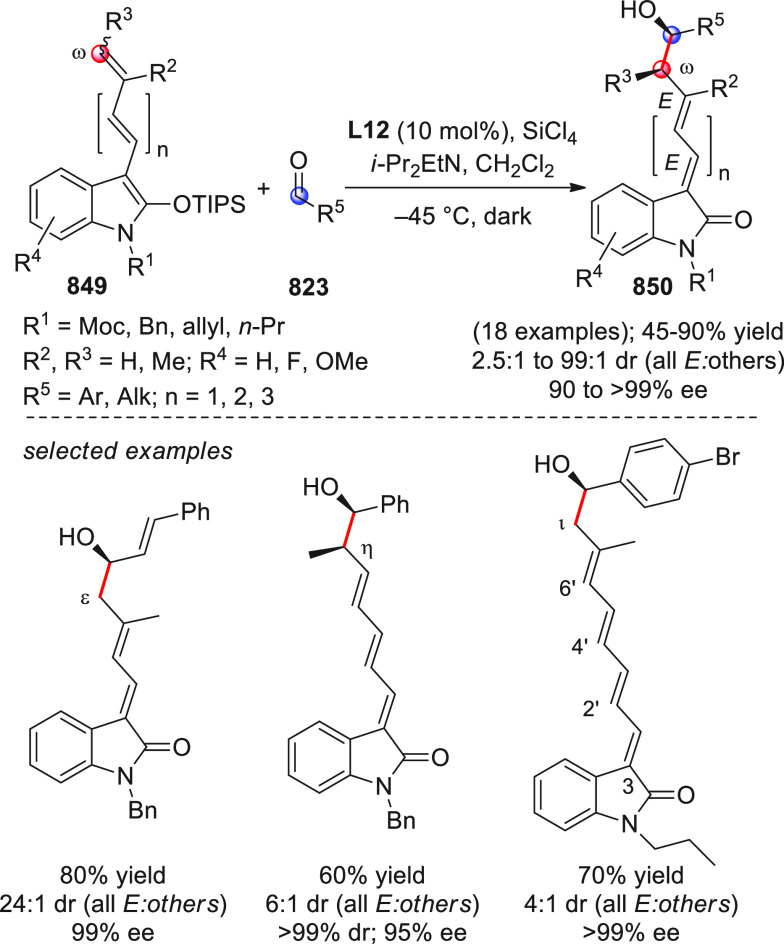


Carrying on their studies on the exploration of vinylogous reactivity of oxindole matrices, Curti, Zanardi, and co-workers reported the first example of a catalytic, enantioselective hypervinylogous Mukaiyama aldol reaction (HVMAR) involving highly unsaturated 2-silyloxindoles of type **849** as donor substrates (Scheme 216).^566^ The silyl polyenolates **849** were reacted with various aromatic and aliphatic aldehydes **823** in the presence of the previously disclosed bisphosphoramide **L12**/SiCl_4_ catalytic system, to access enantioenriched 3-polyenylidene homoallylic carbinols **850** in high yields, with excellent levels of regioselectivity, enantioselectivity, and double bond geometrical selectivity. The study was centered on the exploration of the vinylogous transmittal of the enolate lactam reactivity along the exocyclic polyene chain. Thus, 3-butadienyl silyloxyindoles and higher homologous were selected as nucleophiles with the aim of providing complete regiocontrol at the very remote position according to the concept of hypervinylogy. After careful preliminary work on variously protected 3-butadienyl derivatives **849** (n = 1) and benzaldehyde **823** (R^5^ = Ph), intended to profile the best reaction conditions, it was found that the **L12**/SiCl_4_ couple efficiently catalyzed the bisvinylogous reaction at −45 °C in the dark (to avoid the double bond isomerization) and that the nitrogen protecting group greatly impacted on the outcome of the reaction, with benzyl group giving the best results in terms of regio-, diastereo-, and enantioselectivity as compared to the Moc group. The *N*-benzyl substitution was the best choice also in the case of the trisvinylogous version of the reaction (**849**, n = 2, R^2^ = Me, R^3^ = H), while in the case of the tetravinylogous reaction, the *n*-propyl *N*-protecting group ensured the best results (**849**, n = 3, R^2^ = Me, R^3^ = H). The ^13^C NMR analysis of the chemical shifts of the remote C-ω sites and the conformational analysis carried out on selected polyenolate donor species enabled the rationalization of the obtained results suggesting that the vinylogous reactivity propagation of polyene donors was dependent on the electron-donating properties of the indole *N*-substituents as well as the coplanarity of the polyene chain, which is strictly related to the vinylogous transmittal properties.

### 6.2 Additions to C=N Bonds

During the period analyzed in the present review, the growing interest toward the asymmetric synthesis of Mannich products, that are chiral δ-amino α,β-unsaturated carbonyl derivatives, prompted the implementation of new asymmetric methodologies falling in the field of vinylogous additions to C=N bonds that, starting from either cyclic or acyclic α,β-unsaturated amides, opened the access to chiral functionality-rich vinylogous δ-aminated architectures as skillfull precursors in the synthesis of alkaloidal/aminated bioactive substances.

#### 6.2.1 Direct Procedures

##### 6.2.1.1 Acyclic Pronucleophiles

**Scheme 217:**
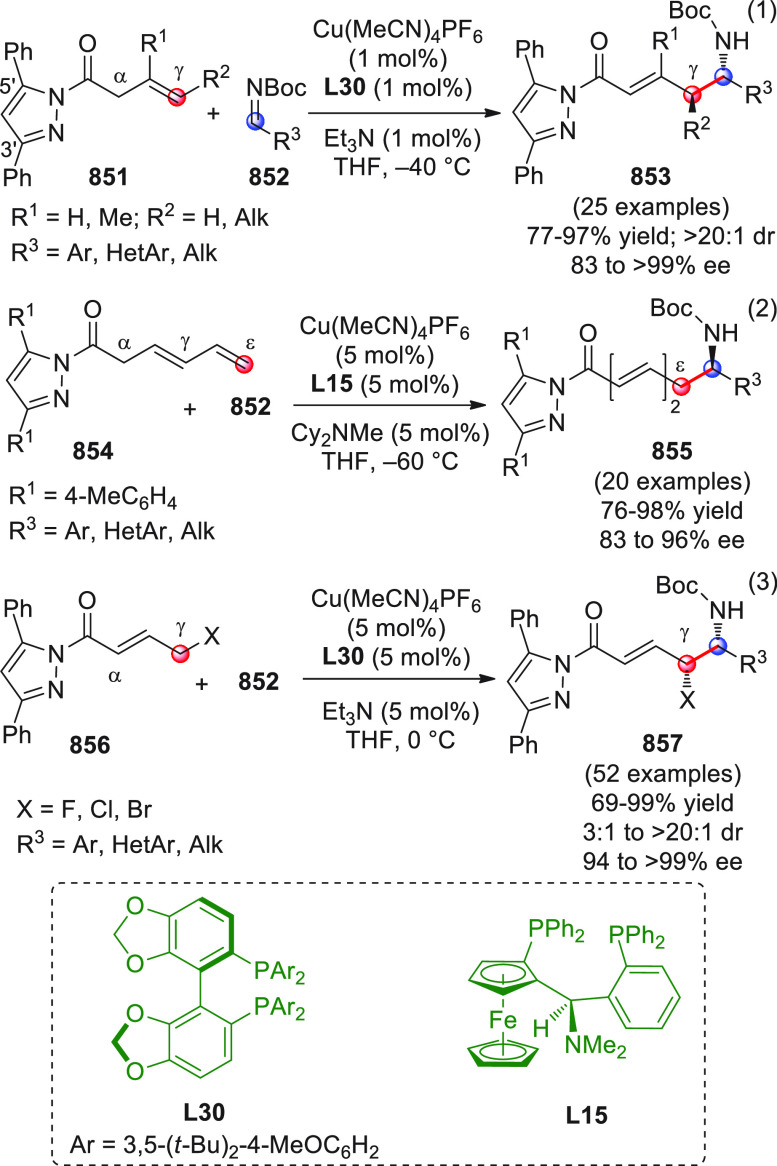


Aiming at developing direct asymmetric reactions of α,β-unsaturated carbonyl compounds to be used as vinylogous donors in Mannich additions to imine electrophiles, Yin and colleagues proposed the β,γ-unsaturated *N*-acylpyrazoles **851** and their bisvinylogous counterparts **854** as pronucleophilic substrates in direct Mannich additions to *N*-Boc aldimines **852** under the guidance of the copper(I)-**L30** (or **L15**) catalytic systems (Scheme 217).^567^

A careful exploration of the model reaction led to the finding that regioselectivity with this type of substrates could be improved by increasing steric hindrance at the pyrazole C3′ and C5′ positions and by using the bulky copper(I) bisphosphine complex based on **L30**, the so-called (*R*)-DTBM-SEGPHOS, thus favoring addition at the γ-site. The authors evaluated the substrate scope of the direct catalytic vinylogous Mannich addition by testing aromatic, heteroaromatic, and aliphatic aldimines, achieving (5*S*)-configured vinylogous δ-amino derivatives **853** (Scheme 217, eq 1) with complete γ-selectivity (γ:α > 20:1) in good to high yields and stereoselectivities, even when γ-substituted acylpyrazoles **851** (R^2^ = alkyl) were used (>20:1 diastereomeric ratio in favor of 4,5-*anti*-adducts). The authors succeeded in the development of the bisvinylogous version of the Mannich reaction by reacting acylpyrazoles **854** (Scheme 217, eq 2) and aldimines **852**; in this case, the ferrocene-based bisphosphine **L15** performed as the best ligand in the presence of Cy_2_NMe as the base, giving the ε-adducts **855** regioselectively (ε:α > 20:1) and in an enantiocontrolled manner.

The same research group, aiming at the construction of halogenated allylic carbon stereocenters, in 2018 described the application of the above disclosed asymmetric Mannich reaction to γ-halogenated α,β-unsaturated *N*-acyl pyrazoles **856** and aldimines **852** (Scheme 217, eq 3).^568^ As in the case of pyrazoles **851**, the bulky bisphosphine ligand **L30** was the key to perfectly control the regioselectivity, as well as the diastereo- and enantioselectivity. The optimized reaction conditions found in the model reaction for the fluoro-derivatives **856** (X = F), were applied to various aromatic, heteroaromatic, and aliphatic aldimines **852** giving *syn*-adducts **857** in good to excellent yields and stereoselectivities; when γ-chloro-substituted pyrazoles **856** (X = Cl) were used, copper(I)/**L30** catalyst (3 mol %) and Et_3_N were enough to catalyze the Mannich addition to aldimines with generally excellent results. The mild reaction conditions, the broad substrate scope, the good tolerance of functional groups, and remarkable regio- and stereoselectivities, all these features definitely demonstrated the robustness and versatility of the proposed methodology in the vinylogous (and bis-vinylogous) Mannich reaction domain.

##### 6.2.1.2 Cyclic Pronucleophiles

The use of cyclic amide pronucleophiles in direct vinylogous Mannich reactions has been scantly investigated; just a couple of examples are reported in the literature in which the dienolate donors belong to the alkylidene oxindole family.

**Scheme 218:**
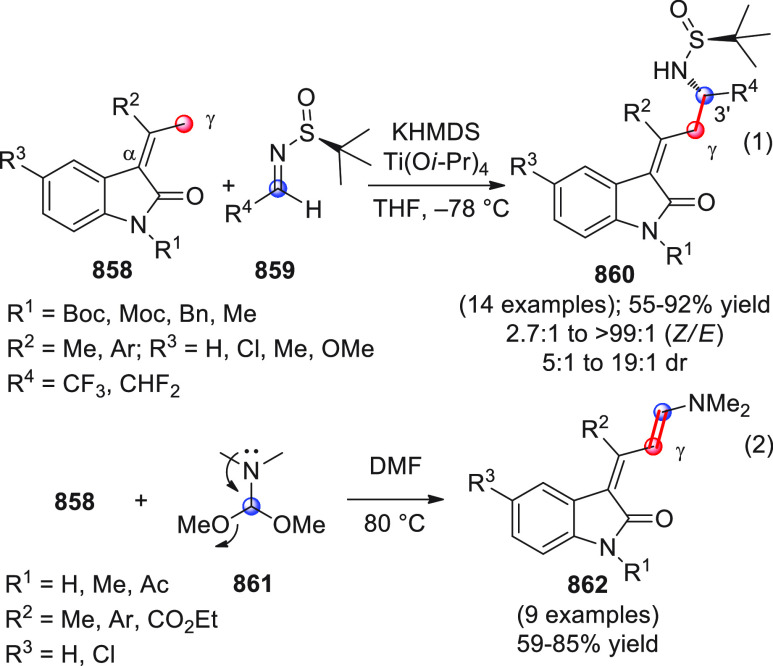


The first example concerns the vinylogous Mannich addition of alkylidene oxindoles **858** to chiral fluoroalkyl aldimines **859** disclosed by Qing et al. in 2015 (Scheme 218, eq 1).^569^ The reaction was carried out in the presence of the strong base KHMDS to promote the γ-enolization of oxindoles **858** at −78 °C, while sulfinyl imine **859** was activated by means of Ti(O*i*-Pr)_4_ as the Lewis acid. The addition resulted in the formation of α-alkylidene-δ-amino-δ-fluoromethyl oxindoles **860** in moderate to good yields and complete γ-regioselectivity (γ:α > 99:1). The degree of diastereselection was good in terms of *Z/E* double bond preference and substrate facial control, with (3Z,3′*S*)-configured isomers formed as the major products. Substitution at C5 of the oxindole ring with both electron-donating and electron-withdrawing groups was well tolerated, as well as the various *N*-protecting groups having different electronic properties and steric hindrance. The authors explained the substrate-controlled stereoselectivity of the addition through a nonchelated transition state model in which the sulfinyl oxygen coordinates the titanium isopropylate thus sterically shielding the *Re* face of the imine acceptor (not shown). In the same study, the authors also reported the reaction of an alkylidene benzofuranone, a scantly exploited vinylogous pronucleophile, with a chiral trifluoromethyl sulfinyl imine that produced a *Z*-adduct with comparable efficiency and stereoselectivity as the nitrogen counterpart.

In the second contribution authored by Kim and co-workers, the alkylidene oxindoles of type **858** were reacted with *N*,*N*-dimethylformamide dimethyl acetal **861** as the electrophilic partner, which at the same time generated the iminium ion acceptor and the methoxide ion acting as the base in deprotonating the oxindole γ-methyl group (Scheme 218, eq 2).^570^ The reaction was performed in DMF at 80 °C resulting in (*E,E*)-configured Mannich adducts 3-dimethylamino-2-propenylidene oxindoles **862** quite efficiently, after methanol elimination.

#### 6.2.2 Indirect Procedures

##### 6.2.2.1 Cyclic Nucleophiles

The vinylogous asymmetric Mukaiyama-type Mannich reactions of pyrrole-based silicon dienolates with imines provide an effective and straightforward way to construct rare α,β-unsaturated γ,δ-diaminocarbonyl frameworks, which are crucial motives in the synthesis of many natural and synthetic compound classes. In the past 2010–2018 period, the use of linear acyclic silyl ketene *N*,*O*-acetals as vinylogous donors in Mannich-type processes has been almost completely disregarded, while a limited number of studies involving silyloxy pyrroles (or oxindoles), classified as cyclic silyl ketene acetals, was found and commented on here.

**Scheme 219:**
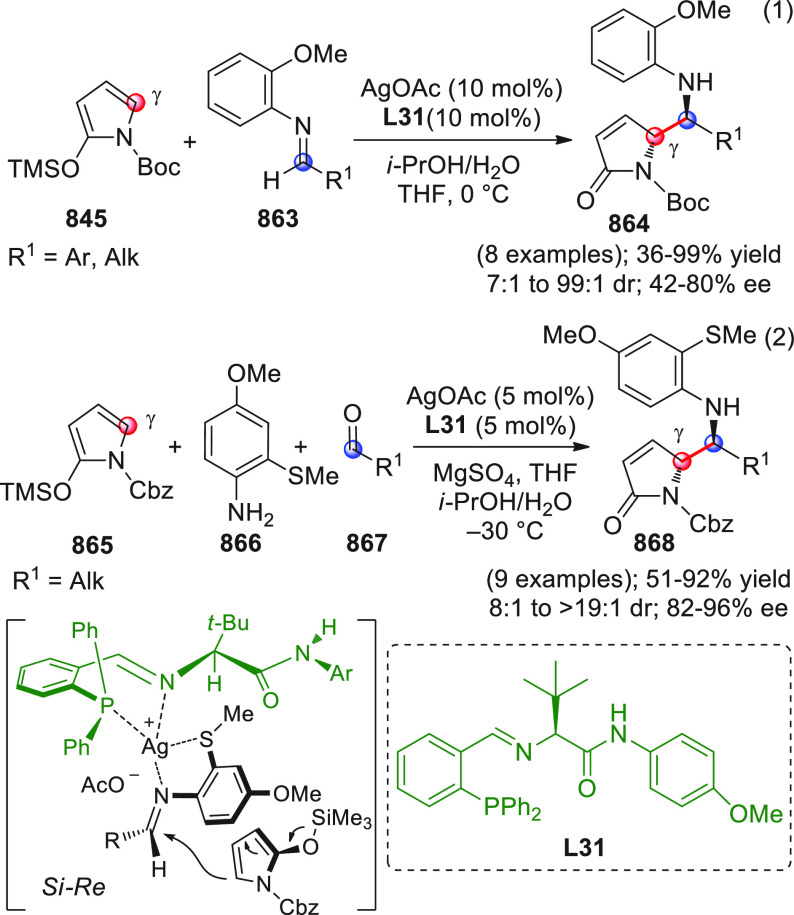


In this research domain, Zanardi, Curti, and co-workers developed *anti*-selective, catalytic asymmetric Mannich addition protocols to access δ-aminated entities, by exploiting siloxy pyrroles as donors and preformed or in situ generated aldimine acceptors (Scheme 219).^571,572^ Excellent results in terms of regio-, diastereo-, and enantioselectivity were achieved utilizing, as the catalyst of choice, *tert*-leucine-derived ligand **L31** in complex with silver(I) acetate, a system ideated and exploited by the Hoveyda and Snapper group a few years ago.^573−575^ As shown in Scheme 219, two different optimal protocols were elaborated, according to the nature of aldimine acceptors. When aromatic aldehyde substrates were involved, the vinylogous Mannich addition of *N*-Boc protected pyrrole **845** and preformed *N*-aryl imines **863** with ligand **L31** and AgOAc (10 mol % each) performed well, while the reaction of alkyl (and hydroxyalkyl) aldehyde substrates **867** entailed a three-component sequential addition protocol, where the imine acceptors were formed in situ (from aldehydes **867** and *o*-thiomethyl-*p*-anisidine **866**) prior to the addition of the catalyst and the *N*-Cbz candidate **865**. Invariably, both protocols performed well, returning the expected *anti*-configured Mannich adducts **864** and **868** in high yields, excellent diastereomeric ratio, and moderate to good enantioselectivities. The synthetic versatility of the unsaturated lactam adducts was demonstrated by the transformation of a hydroxylated lactam, deriving from the reaction between pyrrole **865**, d-glyceraldehyde and amine **866**, into an unprecedented bicyclic furopyrrolone product, reminiscent of the structure of the naturally occurring (+)-goniofufurone (not shown). Based on the pioneering studies by Hoveyda and Snapper on silver-catalyzed asymmetric VMMnR of furan silyl dienolates, the authors proposed a transition state (Scheme 219, bottom left) accounting for the observed *anti*-configuration of the Mannich products. In the event, the *o*-thiomethyl group and the nitrogen atom within the imine acceptor participate in the tetracoordinated silver complex, thus exposing the imine *Re* face to the *Si* face of the incoming silyloxy pyrrole, with the catalyst amide carbonyl acting as a silicon scavenger; the tightly organized complex was judged responsible for the Lewis acid activation of the substrate and the Lewis base activation of the enolsilane, while determining the relative orientation of the reacting partners with the imine *anti*-disposed with respect to the bulky *tert*-butyl substituent within the catalyst ligand.

A few years later, following their longstanding interest in the exploitation of amino acid-based chiral phosphine catalysts, Hoveyda, Snapper, and co-workers applied the vinylogous Mukaiyama–Mannnich addition protocol to the same pyrrole **845** with preformed *o*-methylthio-*p*-anisidine imino-derivatives in the presence of silver(I) acetate and isoleucine-derived phosphine catalyst (not shown).^419^ The process turned out to be viable and enantioselective with aryl, alkyl, and alkynyl imines, even in the case of in situ formation of the Mannich acceptor.

**Scheme 220:**
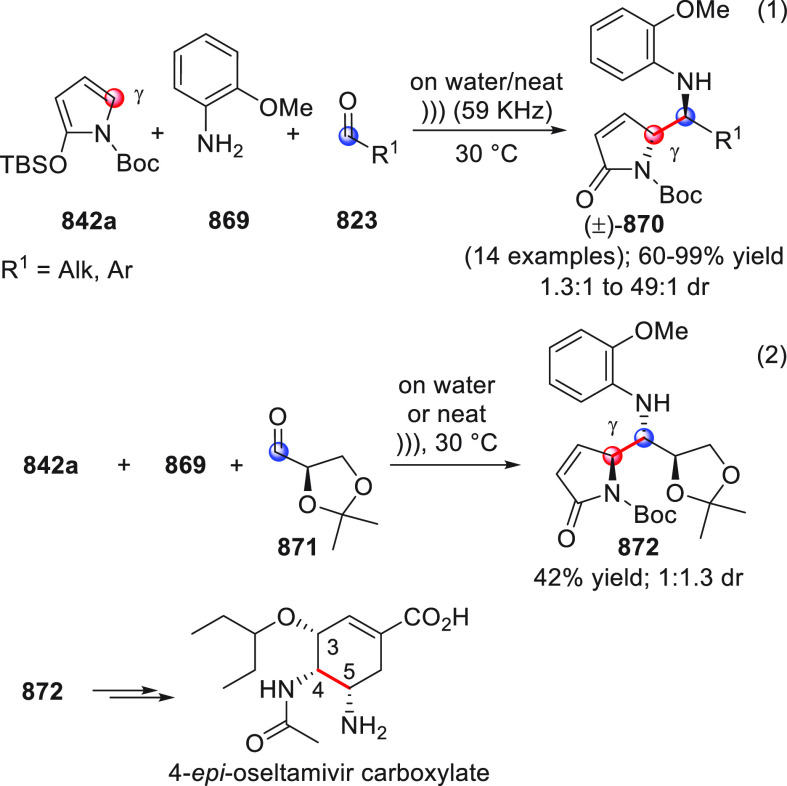


The development of reliable and efficient chemical methodologies that fulfill such representative keywords as water, solvent-free, environment awareness, practicality, and atom economy is one of the most challenging issues of contemporary organic synthesis. Along this line, Zanardi et al. presented the first catalyst-free three-component vinylogous Mukaiyama–Mannich reaction (VMMnR) employing pyrrole silyl dienolates of type **842a**, *o*-anisidine **869**, and diverse aldehydes **823**, which performed well in both aqueous and solvent-free environments (Scheme 220).^576^ Both lipophilic and hydrophilic, aromatic and aliphatic aldehydes proved to be competent substrates giving access to a wide collection of racemic α,β-unsaturated δ-aminolactams **870** in high isolated yields, with excellent levels of γ-site selectivity and chemoselectivity, and moderate to high diastereoselectivity in favor of *anti*-configured adducts. Of note, aqueous and solvent-free protocols were particularly suited for protected alkoxy aldehydes and highly hydrophilic substrates, leading to the corresponding adducts with promising yields and favorable *anti*-diastereoselectivity. As for the role exerted by water in this vinylogous Mannich reaction, it was recognized to be a critical ingredient acting as both the proton donor, to activate the in situ generated imine, and the silicon scavenger, to sequestrate the silicon ion into an inactive R_3_SiOH species, the sole byproduct in these environmentally benign transformations. Soon after, the same authors envisaged taking full advantage of this three-component vinylogous Mannich reaction in planning the stereodivergent synthesis of certain stereoisomers of the anti-influenza agent oseltamivir carboxylate.^577^ The initial VMMnR of siloxy pyrrole **842a**, d-glyceraldehyde acetonide **871** and amine **869** (Scheme 220, eq 2), which installed the entire carbon skeleton and heteroatom substituents of the target, was carried out according to the previously disclosed one-pot three-component protocol in both aqueous and solvent-free conditions, and afforded the expected adducts in high global yield (on-water, 95% yield; neat, 96% yield) as a mixture of the three separable diastereoisomers, namely, the *anti,anti*-configured product **872** (Scheme 220, eq 2), along with minor amounts of *syn,anti*- and *syn,syn*-diastereoisomers (not shown). The most abundant isomer **872** was then converted into the target 4-*epi*-oseltamivir carboxylate (14 steps, 20.2% overall yield), an unprecedented stereoisomeric variant of the antiviral drug, whose inhibitory activity toward influenza A virus neuraminidase was assayed.

**Scheme 221:**
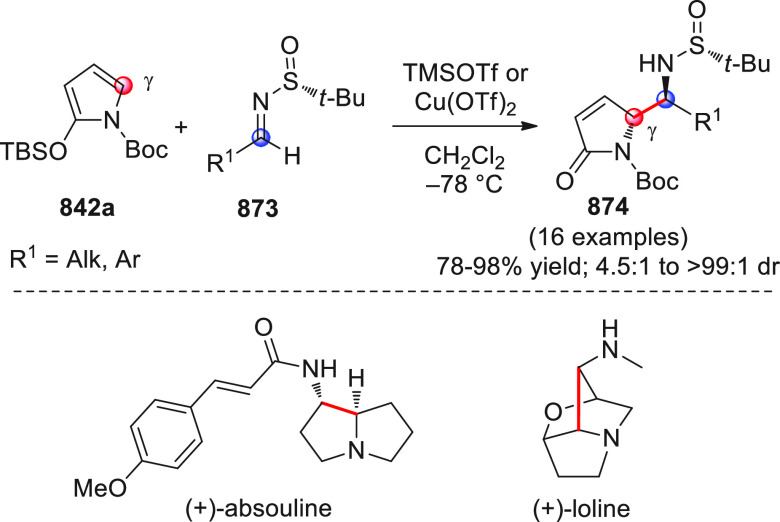


The utility of the vinylogous Mannich addition of pyrrole **842a** to chiral *N*-*tert*-butanesulfinimines **873** according to a classical substrate-controlled approach was demonstrated by Ye, Huang, et al. in the total synthesis of the amino-pyrrolizidine alkaloid (+)-absouline and a precursor of (+)-loline, both characterized by vicinal *anti*-diamine motives (Scheme 221).^578,579^ During a preliminary work, the authors explored the efficiency of the TMSOTf-promoted Mannich additions to diverse *N*-*tert*-butanesulfinimines **873** prepared from (*R*_*S*_)-*tert*-butanesulfinamide and aliphatic or aromatic aldehydes, affording the corresponding *anti*-adducts **874** in good to excellent yields and remarkable stereoselectivity. In the event, the imine of TIPS-protected 3-hydroxypropanal was used as starting material to prepare the 1-aminopyrrolizidine alkaloid (+)-absouline. In the second contribution, the same authors exploited the previously disclosed VMMn addition of pyrrole **842a** to the sulfinyl imine of l-glyceraldehyde aiming to directly access the vicinal diamino motif present in the (+)-loline.

**Scheme 222:**
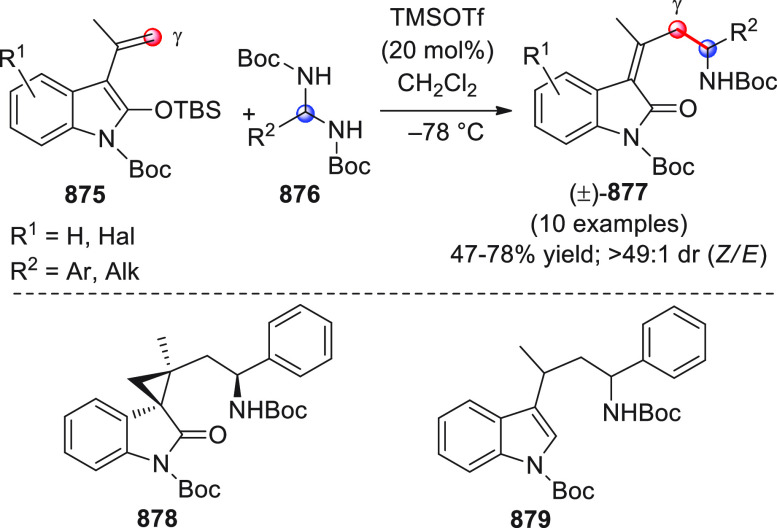


Following their interest in the full exploitation of γ-enolizable alkylidene oxindoles as extended carbon nucleophiles, Sartori, Zanardi, et al. explored the use of the oxindole *N*,*O*-silyl ketene acetals **875** in VMMnRs involving imine precursors **876** (Scheme 222).^580^ The propenyl silyloxy indoles **875**, already utilized in vinylogous aldol maneuvers (vide supra), were coupled to di-Boc aminals **876** with catalytic TMSOTf (20 mol %) which triggered the in situ formation of the imine electrophiles. The reaction proved to be quite efficient (47–78% yields), giving the corresponding chiral δ-amino-2-oxindoles **877** in a racemic format with complete γ-selectivity (γ:α > 49:1) and excellent diastereoselectivity in favor of *Z*-adducts. The protocol worked well with both aromatic and aliphatic aminals, and a representative δ-amino-2-oxindole deriving from benzaldehyde aminal (R^2^ = Ph) served as a precursor in the preparation of indole-based architectures including spirocyclopropaneoxindole **878** and homotryptamine analogue **879** (Scheme 222).

### 6.3 Conjugate Additions to Electron-Poor C=C Bonds

Asymmetric vinylogous reactions enabling the straightforward access to amide structures decorated with electron-deficient appendages (e.g., carbonyl, nitro, or cyano groups), double bonds, and stereocenters at their γ- (or more remote) positions are of wide interest in synthetic chemistry, as they can represent key intermediates in the diversity-oriented synthesis of natural and non-natural compounds of biological interest. In this context, many efforts have been devoted to the development of catalytic asymmetric methodologies based on vinylogous 1,4 additions to activated double bonds which provide chiral high-value α,β-unsaturated δ-substituted amide scaffolds bearing additional carbonyl, nitro, or cyano substituents at their ε-position.

#### 6.3.1 Direct Procedures

##### 6.3.1.1 Acyclic Pronucleophiles

**Scheme 223:**
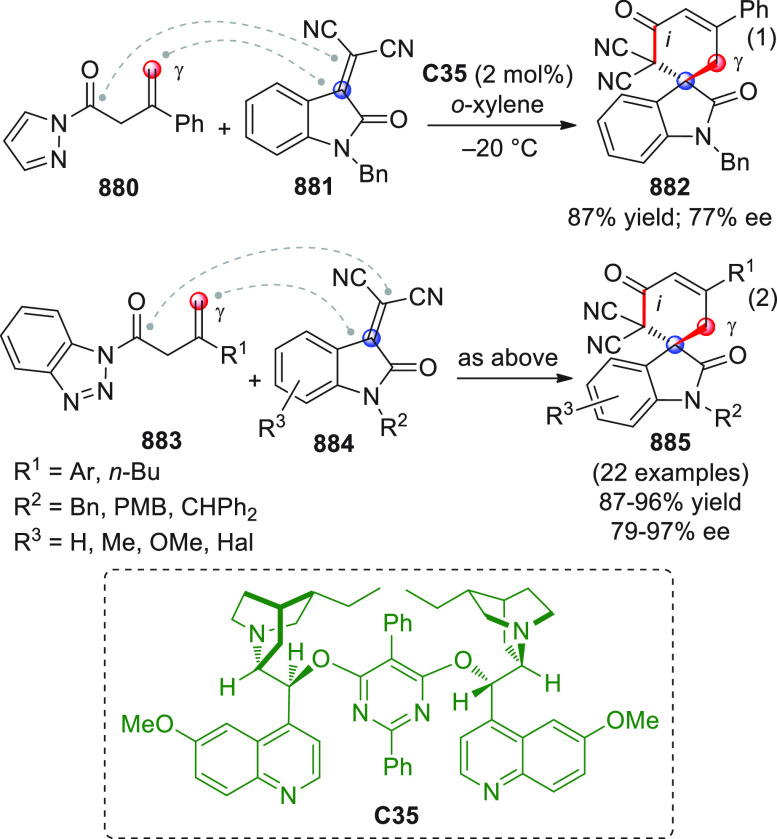


The elaboration of asymmetric synthetic methodologies based on vinylogous addition–cyclization cascades to directly access spirocyclic oxindoles has recently attracted growing interest, as already mentioned for the aldol domain (vide supra). As a continuation of their efforts toward the synthesis of spirooxindoles as privileged structural motives in bioactive compounds, Sha, Wu, and colleagues developed a catalytic enantioselective vinylogous Michael addition–cyclization sequence involving acyclic β,γ-unsaturated amides of type **880** or **883** as vinylogous pronucleophiles and isatylidene malononitriles **881** or **884** as electrophilic partners (Scheme 223).^581^

Initially, the authors explored the reaction between pyrazole amide **880** and alkylidene malononitrile **881** (Scheme 223, eq 1) as a model to screen for the best organocatalyst and reaction conditions. The reaction worked well in the presence of cinchona-derived catalyst **C35** [(DHQD)_2_PYR, 2 mol %], affording the spirocyclic adduct **882**. According to the postulated reaction mechanism, the dienolate formed by deprotonation of the quinuclidine base reacted at its γ-position with the isatylidene malononitrile acceptor; the resulting carbanion intermediate attacked the amide carbonyl providing, after elimination of the pyrazole unit, the spiro-compound, thus completing the reaction cascade. During the evaluation of the substrate scope, the authors found that the use of 1*H*-benzotriazole allyl amides **883** as pronucleophiles gave the best results in terms of both yields and enantioselectivities; moreover, the *N*-protecting group and the substitution pattern at the aromatic ring of the oxindole acceptor **884** were examined giving generally good yields and good to excellent enantioselectivities (Scheme 223, eq 2).

**Scheme 224:**
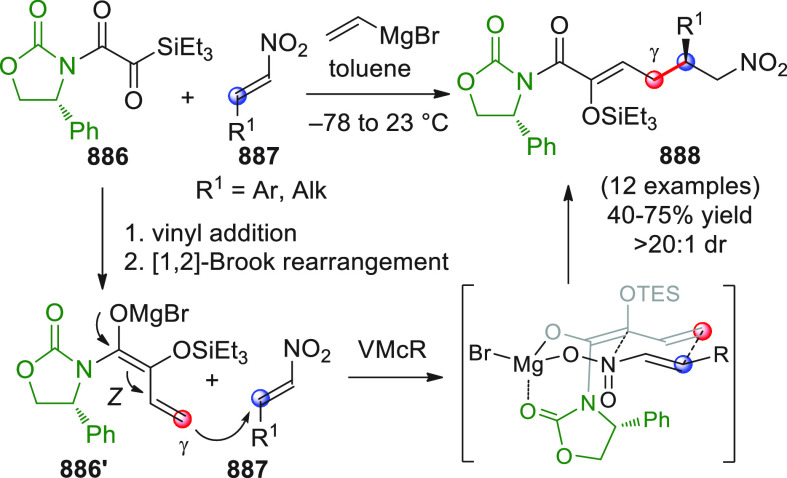


In 2016, Boyce and Johnson reported an example of stereoselective auxiliary-driven vinylogous Michael cascade exploiting silylglyoximide **886** as latently chiralized pronucleophile and nitroalkenes **887** as the acceptor partners (Scheme 224).^582^ The in situ formation of the *Z* magnesium dienolate **886′** was initiated by vinylation of acyl silane **886**, followed by [1,2]-Brook rearrangement (the carbon-to-oxygen silyl migration); then the dienolate **886′** coupled to nitroalkenes **887**, thus terminating the vinylogous Michael cascade. The three-component reaction sequence yielded functionality-rich *Z*-silyl enol ethers **888** with complete chemo- (only traces of Grignard addition to nitroalkene were detected), regio- (only γ-adducts were observed), and facial selectivity (>20:1 diastereomeric ratio). Even though the efficiency of the process was poor, with yields ranging from 40% to 75%, the scope of the nitroalkene was broad, tolerating aryl, heteroaryl, alkyl, and alkenyl substituents. Of note, the nitrodiene **887** having R = styryl exhibited 1,4-selectivity instead of the potentially competitive 1,6-addition, providing evidence for the possible transition state accounting for such a high level of stereoinduction. In fact, supported by DFT calculations, a highly organized “*trans*-decalin” model was proposed, where the magnesium ion strongly coordinates both the auxiliary carbonyl oxygen and the nitro group, driving the approach of the dienolate to the *Si* face of the nitrolefin having the R substituent in a favorable pseudoequatorial position. The developed methodology provided direct and stereoselective access to densely functionalized α-heterosubstituted nitro-derivatives **888** whose *Z*-enol ether configuration prevented the Henry cyclization, commonly observed with similar intermediates.^266^

##### 6.3.1.2 Cyclic Pronucleophiles

Among the cyclic amide pronucleophiles, the *N*-Boc pyrrolinone has been considered and still represents a valuable heterocyclic building block; it is prone to quite easy γ-enolization, and it may be used as a versatile d_4_ synthon in direct, catalytic vinylogous Michael addition reactions with electron-poor alkenes, giving access to highly functionalized γ-substituted pyrrolinone scaffolds in an efficient and atom economical manner.

In this research field, Chen and co-workers in 2010^583^ reported the first direct organocatalytic conjugate addition of *N*-Boc-pyrrolinone to α,β-unsaturated aldehydes under iminium ion activation and, in the same year, Shibasaki and colleagues described the direct catalytic asymmetric Michael (and Mannich) addition of unsaturated γ-butyrolactams to nitroolefins (and aromatic aldimines) in the presence of a dinuclear nickel complex, achieving outstanding results in both cases (not shown).^584,585^

**Scheme 225:**
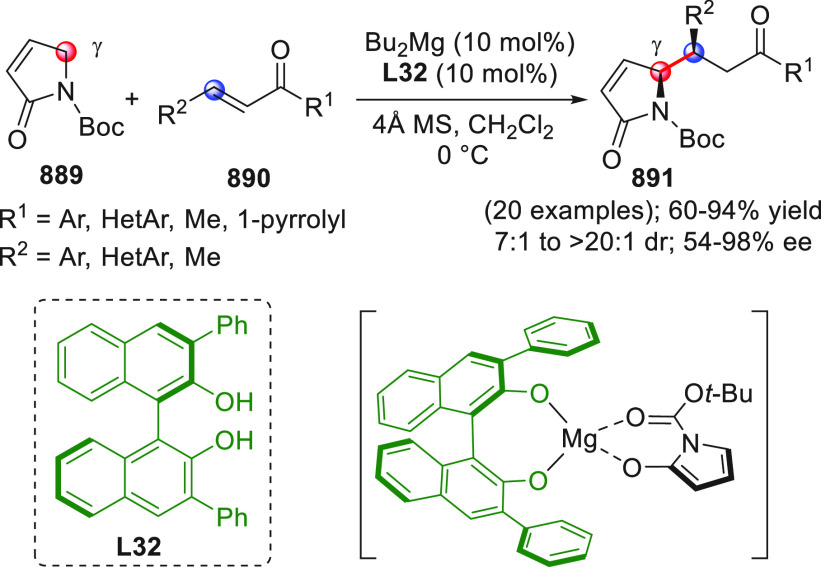


The organometallic catalysis in asymmetric vinylogous Michael additions was applied shortly after by Wang et al. in a work appearing in 2011 (Scheme 225).^586^ The approach devised by these researchers to direct the addition of *N*-Boc pyrrolinone **889** with a series of chalcones and related derivatives **890** entailed the use of the magnesium/3,3′-Ph_2_-BINOL **L32** complex as the optimum catalyst system, which was prepared in situ by reacting the ligand **L32** with dibutyl magnesium. The addition to a variety of enones afforded *syn*-configured products **891** in high yields, high diastereoselectivities, and variable enantioselectivities (54–98% enantiomeric excess). The selectivity of the reaction was slightly affected by the R^1^ substituent and highly dependent on the nature of the R^2^ group. Bidentate chelation between the in situ-generated *N*-Boc pyrrole dienolate and the chiral BINOL/magnesium complex was supposed to activate the nucleophile, while providing the chemical environment for high stereocontrol (Scheme 225, bottom).

**Scheme 226:**
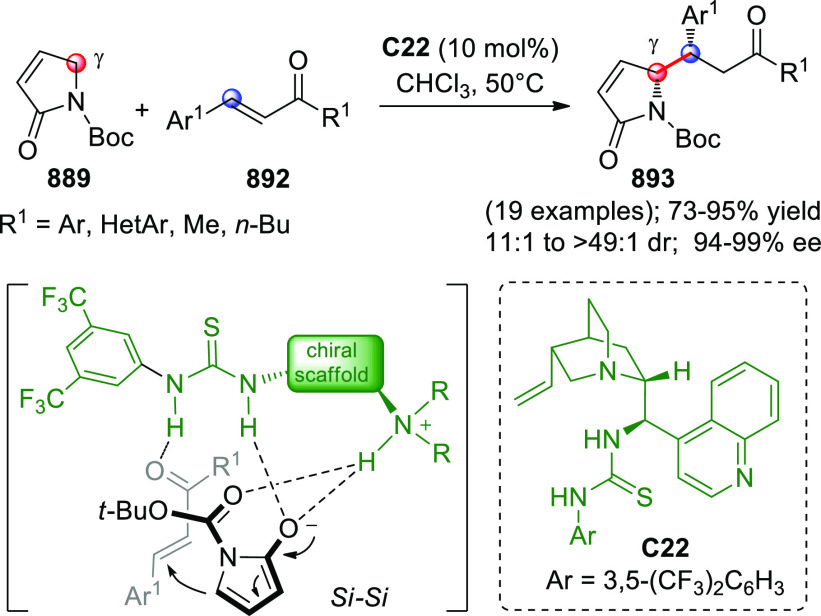


On the other hand, Wang and co-workers adopted a different approach to govern the vinylogous Michael addition between pyrrolinone **889** and diverse chalcones **892**, based on the use of the popular bifunctional cinchona thiourea catalyst **C22** (Scheme 226).^587^ As shown in Scheme 226, the respective *syn*-configured Michael adducts **893** were accessed in high yields, with very high margins of diastereo- and enantioselectivity. The generality of the reaction was quite large, tolerating either electron-donating or electron-withdrawing enone substituents; heterocyclic systems were also reliable substrates, while alkyl-substituted chalcones failed to give appreciable results.

Based on previous investigations on the thiourea-tertiary amine catalytic mechanism,^588−590^ in a subsequent paper the same researchers postulated different possible models for the interaction of the bifunctional catalyst **C22** with the reacting donor and acceptor partners and performed in-depth studies on the same reaction displayed in Scheme 226 by means of a combination of both experimental (NMR) and theoretical (DFT) approaches.^591^ In the event, the authors demonstrated a new dual activation pathway. The key feature of the proposed transition state is that the carbonyl group of the chalcone is activated by the more acidic NH of the thiourea moiety, while the ammonium nitrogen of the catalyst and the second NH of the thiourea simultaneously activate the *N*-Boc lactam nucleophile via hydrogen bonding, thus favoring the *Si*-*Si* approach that accounted for the observed *syn*-configuration of the products.

Many other researchers explored the utility of pyrrolinone **889** as the pronucleophilic substrate in organocatalyzed asymmetric direct VMcR to various acceptors such as nitroolefins, alkylidene malonates, enones, dienones, enoylpyridines, to cite but a few. The results are condensed in Table 9.

**Table 9:**
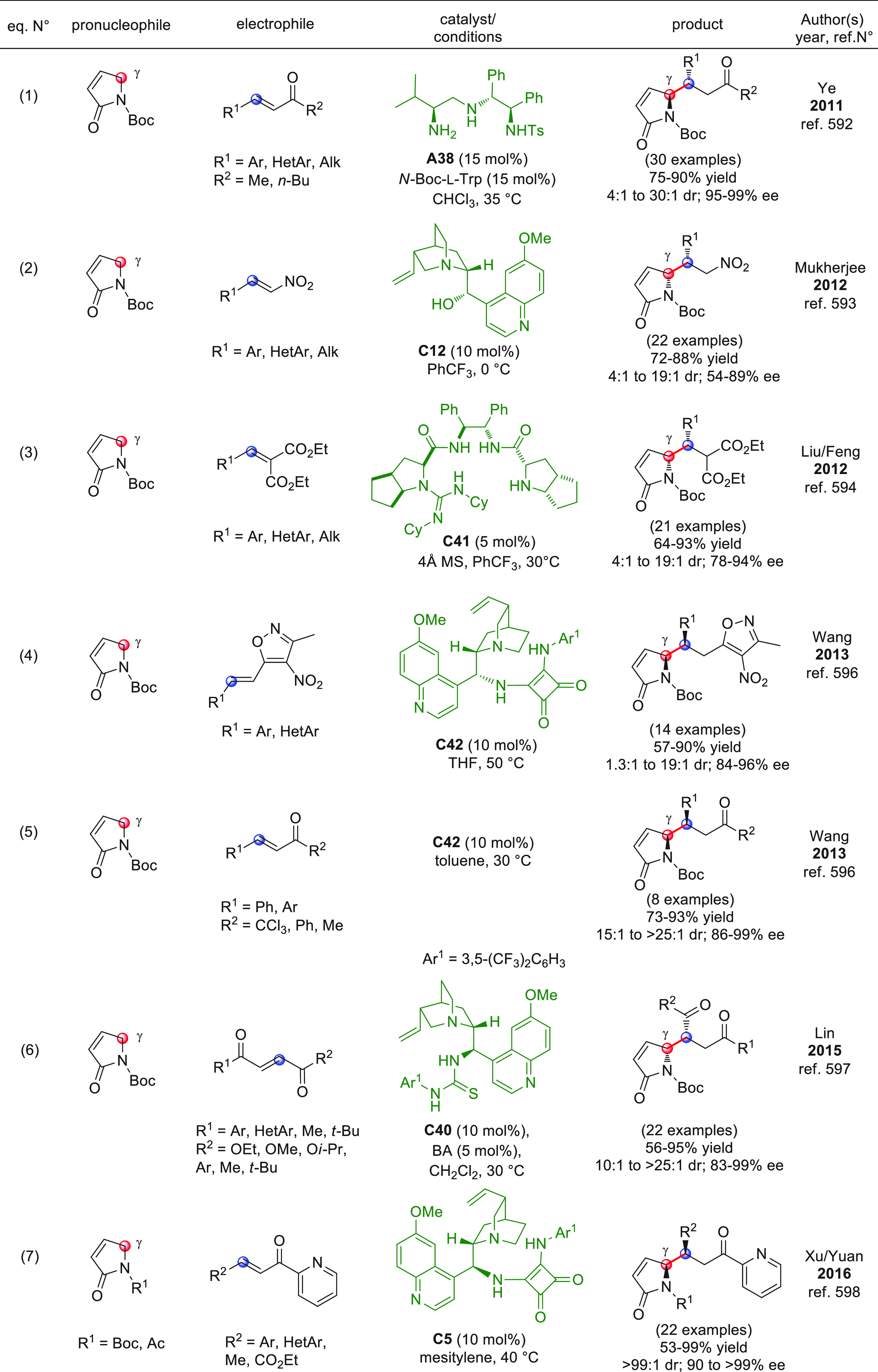

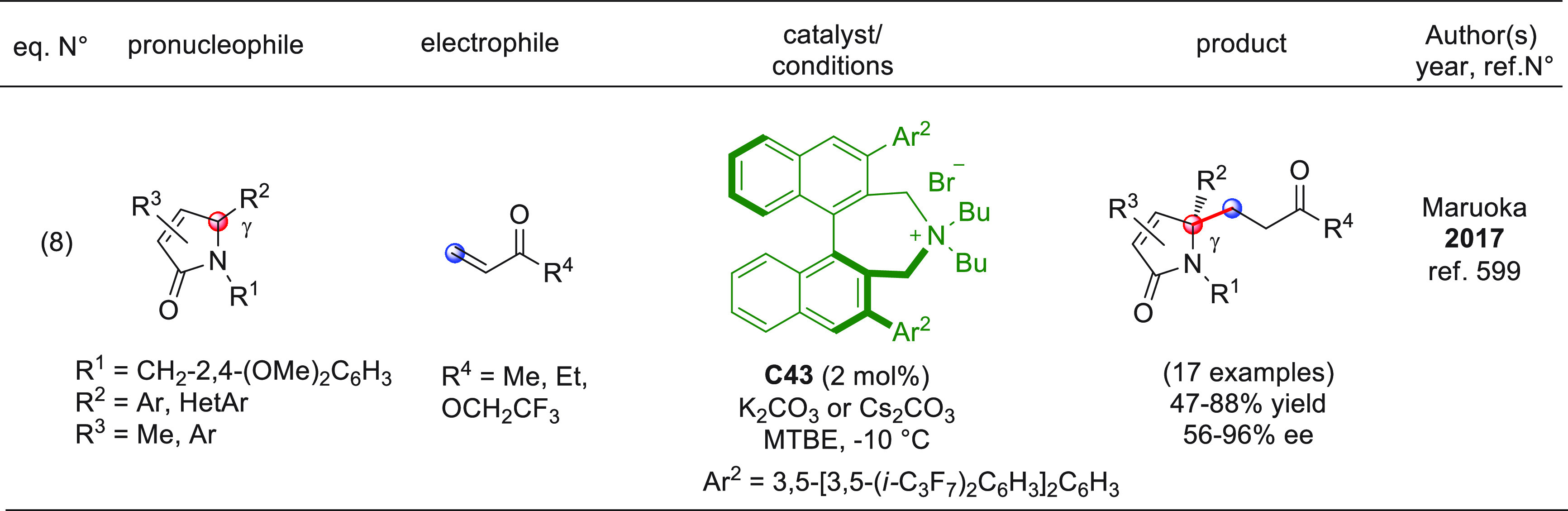
Organocatalyzed Direct Vinylogous Conjugate Additions of *N*-Boc-pyrrolinone to Various Michael Acceptors

The *trans*-stilbene-derived chiral triamine catalyst **A38** and *N*-Boc-L-tryptophan as an additive formed the catalyst system with which Ye et al. investigated the vinylogous Michael addition of *N*-Boc pyrrolinone to rather inert enone acceptors (Table 9, eq 1).^592^ The reaction scope proved to be large, encompassing aryl, heteroaryl, alkyl, and cycloalkyl enone substituents. In any case, uniformly high yields, diastereomeric ratios, and enantiomeric excesses were witnessed for the *anti*-configured pyrrolinone products. In the proposed transition state, LUMO-lowering iminium ion covalent activation of the acceptor by the primary amine of the catalyst, as well as HOMO-raising noncovalent activation of the dienolate donor via H-bonding network were simultaneously operative, along with the participation of the bulky amino acid L-tryptophan via ion pair interaction.

Mukherjee investigated the reaction of *N*-Boc pyrrolinone with a series of aryl, heteroaryl, and alkyl β-nitroolefins under the catalysis of quinidine derivative **C12** (Table 9, eq 2).^593^ S*yn*-configured products were consistently obtained in good yields, excellent diastereoselectivity, and moderate enantiomeric excesses; on the other hand, the enantiomeric products could be accordingly prepared by using a dihydroquinine-derived organocatalyst. As for the mechanism, the authors postulated the activation of the nitroolefin by the quinidine hydroxyl group via H-bonding, whereas the tertiary amine moiety of the catalyst provided for the activation of the lactam pronucleophile.

Feng, Liu, et al. analyzed the behavior of the reaction involving *N*-Boc pyrrolinone and alkylidene malonate acceptors using the unsymmetrical guanidine-secondary amine multifunctional catalyst **C41** (Table 9, eq 3).^594^ Optimal conditions were found using catalyst **C41** (5 mol %) in combination with 4 Å molecular sieves in trifluoromethylbenzene. Reactions proved efficient and selective as far as diastereo- and enantioselectivity were concerned. A mechanistic rationale was proposed, based on control experiments and previous investigations.^595^ Thus, the basic guanidine in the catalyst provided γ-deprotonation of pyrrolinone to form the active dienolate; the *N*-Boc protecting group contributed in supplementary H-bonding to the amide on the same side of the guanidine moiety; meanwhile, the alkylidene malonate was activated through a network of hydrogen bonds with both the secondary amine and the second amide moiety of the catalyst.

The access to chiral substituted γ-butyrolactams by means of direct catalytic asymmetric Michael reactions was developed by Wang et al. by using α,β-unsaturated pyrrolinone as donor and 3-methyl-4-nitro-5-alkenyl-isoxazoles as bisvinylogous Michael acceptors (Table 9, eq 4).^596^ The quinine-derived squaramide **C42**(160) was identified as the best catalyst to govern the 1,6-Michael addition to various aryl nitro-isoxazoles, giving *syn*-adducts in high yields and remarkable diastereo- and enantioselectivity. The same bifunctional organocatalyst proved equally efficient in promoting the conjugate addition of *N*-Boc pyrrolinone to α,β-unsaturated trichloromethyl ketones (Table 9, eq 5).^596^ Excellent diastero- and enantioselectivities were achieved also in the case of chalcone having R^1^, R^2^ = Ph and with less reactive enone having R^2^ = Me.

α,β-Unsaturated γ-butyrolactam was also exploited as a pronucleophile in organocatalyzed vinylogous Michael additions to β-acyl acrylates and ene-diones by Lin and co-workers (Table 9, eq 6).^597^ The use of quinidine-derived bifunctional thiourea **C40** in the presence of benzoic acid enabled the synthesis of the corresponding *syn*-configured butyrolactams in acceptable chemical yields; good-to-excellent diastereo- and enantioselectivities could be achieved with β-aroyl derivatives having R^1^ = aryl or heteroaryl, while with aliphatic keto esters (R^1^ = alkyl) the diastereoselectivity decreased.

Based on their previous studies on the organocatalyzed vinylogous conjugate additions of butenolides to enoylpyridines (vide supra, Table 8, eq 4), Xu, Yuan, et al. demonstrated the efficacy of the same quinine-derived squaramide **C5** as the catalyst in Michael additions exploiting γ-butyrolactams as pronucleophilic reagents (Table 9, eq 7).^598^ The reaction was broad in scope concerning the nature of the aromatic ring substituents within the electron-poor acceptors, producing the corresponding adducts in high yields and excellent levels of stereocontrol; the *N*-acetyl protected lactam substrate gave efficiently the corresponding adduct, while with the *N*-methyl counterpart the reaction failed and no desired products were found. Based on experimental results and previous experience, the authors proposed a possible transition state model, emphasizing the role of the catalyst tertiary amine in activating the pyrrolinone dienolate and the role of squaramide NH groups in coordinating both the carbonyl and the pyridine nitrogen of the acceptor via H-bonding, thus precisely governing the stereoselective attack.

Aiming to the development of a catalytic enantioselective method to provide γ,γ-disubstituted γ-lactams, Maruoka and colleagues proposed the exploitation of chiral phase-transfer catalysis to promote the vinylogous Michael addition of γ-monosubstituted pyrrolinones to vinyl ketones (or esters) (Table 9, eq 8).^599^ During these studies, any attempt to selectively alkylate the substrates with benzyl bromide failed, while the conjugate addition to α,β-unsaturated carbonyl derivatives in the presence of chiral ammonium salt **C43** worked well and stereoselectively led to unsaturated lactam products bearing a quaternary stereocenter at the γ-position. The introduction of aryl groups at the α-position did not compromise the reactivity and stereoselectivity, indicating the generality of the protocol. Moreover, the strategy was applied to trifluoroethyl acrylate acceptor (R^4^ = OCH_2_CF_3_) with just a modest erosion of the efficiency and selectivity.

**Scheme 227:**
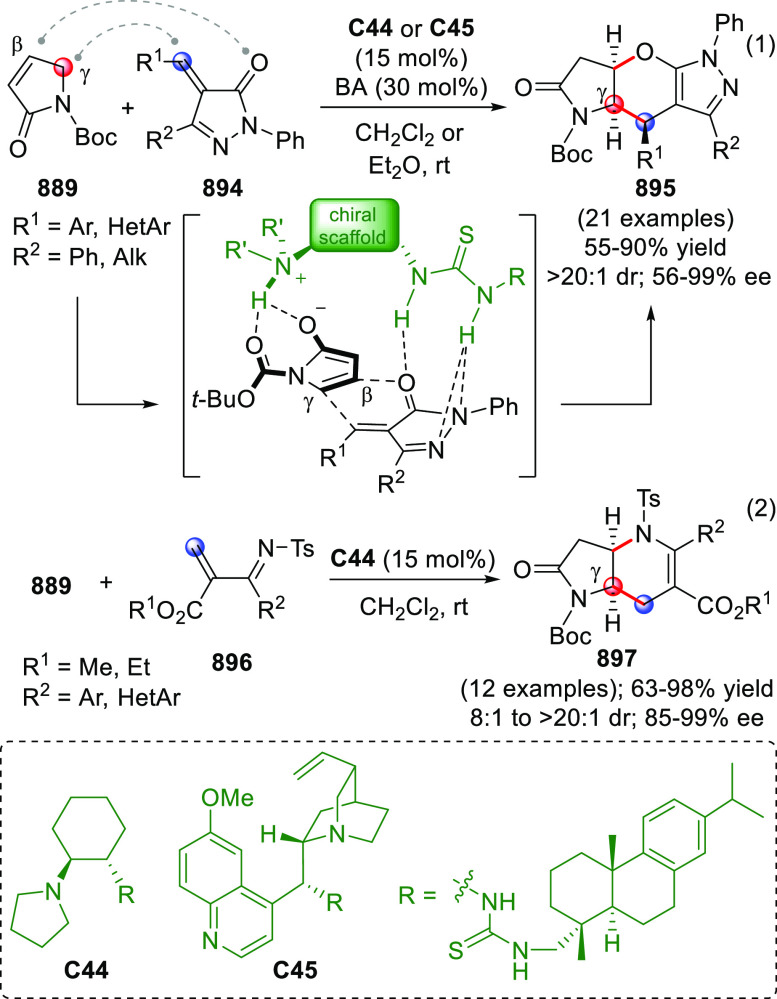


The potential of unsaturated γ-butyrolactams as key precursors in asymmetric organocatalyzed synthetic strategies has been explored by Wang and collaborators, who proposed a direct approach to functionalize the pyrrolidinone scaffolds in β,γ-selective IEDDA [4 + 2] annulations (Scheme 227).^600^ The researchers set out to exploit unsaturated pyrazolones **894** or α,β-unsaturated imino derivatives **896** as electrophilic reagents and chiral bifunctional thioureas **C44** or **C45**(160) as the organocatalysts. In light of their previous investigations,^601^ they envisaged that the dienolate deriving from lactam **889** might serve as electron-rich dienophile undergoing IEDDA annulations with proper electron-poor dienes of type **894** or **896**; in fact, the reaction of **889** with phenyl-substituted pyrazolones **894** worked well in the presence of thiourea **C44**, while the alkyl-substituted counterparts required catalyst **C45** in combination with benzoic acid, affording in one step tricyclic dihydropyranopyrrolidin-2-ones **895** in good to high yields and stereoselectivities (Scheme 227, eq 1). Wishing to explore the substrate scope, the protocol using catalyst **C44** was extended to acyclic imino derivatives **896**, providing a series of chiral aza-analogue architectures **897** in comparable efficient and stereoselective manner (Scheme 227, eq 2).

The authors hypothesized a transition state model (Scheme 227) accounting for the observed stereochemistry of the annulated products, in which a dual HOMO*_dienofile_*/LUMO*_diene_*-activation was operative with the catalyst tertiary amine promoting the in situ dienolate formation and the thiourea moiety activating the pyrazolone acceptor. Although the experimental results were perfectly consistent with the hypothesis, a stepwise mechanism involving a sequential vinylogous Michael/oxa- or aza-Michael reaction cascade could not be ruled out.

**Scheme 228:**
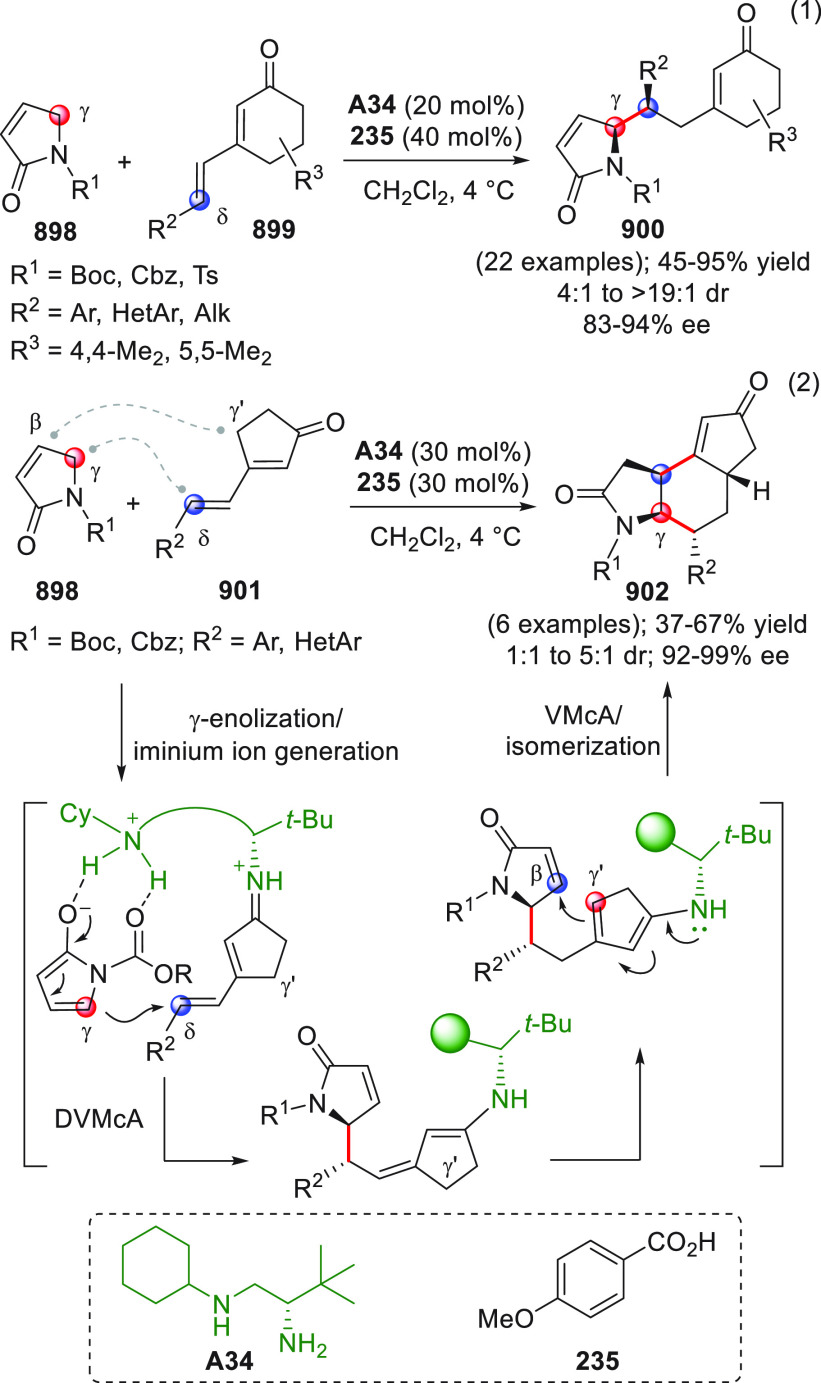


A few years later, the issue of β,γ-functionalizing the pyrrolidinone scaffold was faced by Ye, Dixon, et al., who developed an unprecedented organocatalyzed reaction cascade initiated by a doubly vinylogous Michael addition (DVMcA) between γ-butyrolactams **898** and cyclic 2,4-dienones of type **899** or **901** (Scheme 228).^602^ The challenging simultaneous activation of the vinylogous donor **898** and the rather unresponsive dienones **899**/**901** acting as vinylogous Michael acceptors was obtained by the bifunctional diamine catalyst deriving from l-*tert*-leucine **A34**. When the challenging γ-to-δ 1,6-addition of protected γ-butyrolactams **898** involved the sterically congested β-substituted cyclohexenones **899**, Michael adducts **900** were obtained with high regio- and stereoselectivity in the presence of **A34** and *p*-anisic acid **235** (Scheme 228, eq 1). The remote transmission of the stereochemistry through the conjugate double bonds was ensured by the catalyst diamine functionalities responsible for the simultaneous covalent iminium ion activation of dienones and strong hydrogen-bonding interactions with the deprotonated butyrolactam. On the other hand, when 3-alkenyl 2-cyclopentenones **901** were used as substrates, the initial DVMcA was followed by a vinylogous Michael addition/isomerization cascade involving the γ′-position of cyclopentenone and the β-carbon of the lactam, thus affording tricyclic lactams **902** with four newly formed stereocenters (Scheme 228, eq 2).

**Scheme 229:**
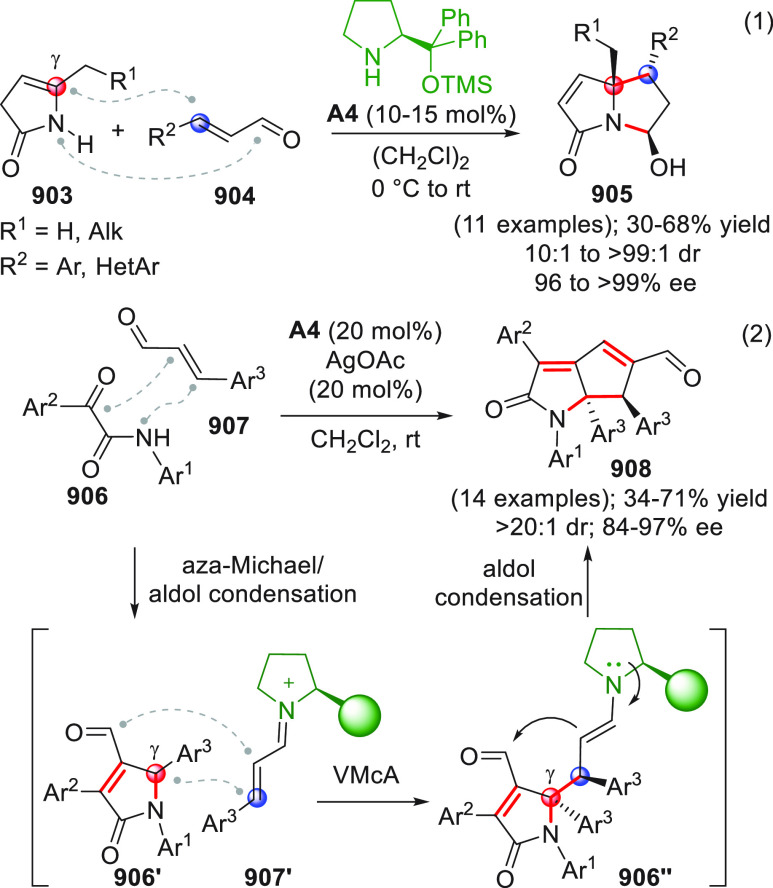


The competence of β,γ-unsaturated γ-lactams in participating in [3 + 2] annulation reactions as multidentate nucleophiles was explored by Kalaitzakis, Vassilikogiannakis, et al. in 2018 during the asymmetric synthesis of a collection of pyrrolizidine bicyclic lactams (Scheme 229, eq 1).^603^ The authors set up a strategy to accomplish asymmetric and site-selective annulations with unconjugated γ-alkyl pyrrolinone **903** and α,β-unsaturated aldehydes **904** under silyl prolinol organocatalysis.

In light of the results, the authors hypothesized that the vinylogous Michael attack of the dienolates deriving from **903** to iminium-ion activated enals **904** was followed by the intramolecular *N*-hemiaminalization, thus providing bicyclic adducts **905** with remarkable levels of stereoselectivities, albeit in fair chemical yields. They postulated that the stereochemistry of **905** resulted from an ion-paired transition state (not shown) that forces the *Si* face of the lactam to attack the *Si* face of the activated enals in the first step of the reaction cascade; the aldehyde group is then trapped in the hemiaminal ring-closure.

The potential of covalent organocatalytic protocols in combination with tandem reactions was demonstrated some years before by Enders and co-workers in the rapid synthesis of a series of highly substituted 2-aza-bicyclooctadienones (Scheme 229, eq 2).^604^ These researchers developed a three-component quadruple reaction cascade of aryl α-ketoamides **906** and enals **907** in the presence of (*S*)-diphenylprolinol TMS ether **A4**. The cascade proceeded via an initial aza-Michael addition of the ketoamide nitrogen to iminium ion-activated enal, followed by the enamine-promoted aldol condensation forming the γ-butyrolactam intermediate **906′**; this last compound participated as vinylogous donor in the Michael addition reaction with a second enal unit, forming the two stereocenters within the γ,γ-disubstituted pyrrolinone **906″** in a highly stereocontrolled manner; the final intramolecular aldol condensation via enamine catalysis completed the target bicyclic compounds **908**. The authors noted that the high stereocontrol in the generation of the two stereocenters depended on the facial selectivity in the key vinylogous Michael addition step; here, the iminium ion-activated α,β-unsaturated aldehyde was attacked on its *Re* face by the dienolate thus generating both the new stereocenters and placing the two Ar^3^ substituents in a *trans* orientation. The whole process was orchestrated by the chiral amine organocatalyst **A4** and produced in good yields complete diastereoselectivity and high enantioselectivity polysubstituted azabicyclooctadienones **908** (Scheme 229, eq 2).

As already noted, 3-alkylidene-2-oxindoles are among the most studied vinylogous heterocycles in Michael additions, which present their enolizable γ- (ε- or ω-) sites out (or far) from the cyclic indole core. Other useful heterocyclic scaffolds of this type are their aza-analogues (aza-alkylidene oxindoles and nitrone ylides deriving from isatin) along with alkylidene pyrazolinones.

It is worth pointing out here that most of the strategies taking advantage of the vinylogous nucleophilicity of alkylidene oxindoles entail direct executions in Michael-type addition reactions.

In the realm of racemic approaches based on alkylidene oxindoles, two contributions are due to the Shanmugam research group, the first of which appeared in the literature in 2010.^605^ Here, the authors reported an example of phosphine-catalyzed direct VMcA involving bromomethyl alkylidene oxindoles as vinylogous nucleophiles and maleimide or methyl acrylate as electrophilic partners (not shown). In the second example,^606^ the same researchers described the diastereoselective synthesis of a series of oxindole-appended vinyl cyclopropanes based on the use of sulfur ylides from bromoallylidene oxindoles and activated styrenes (not shown).

**Scheme 230:**
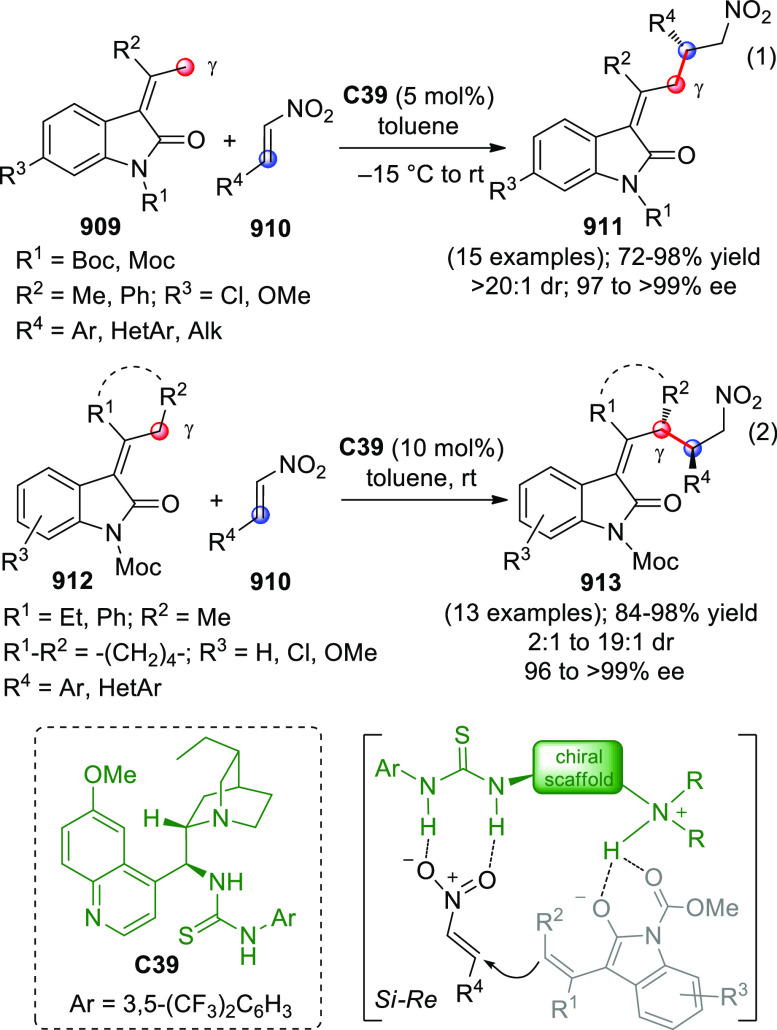


Going through the asymmetric versions of the conjugate additions involving alkylidene oxindoles, the first example of direct organocatalytic vinylogous Michael addition to nitroolefin acceptors was reported by Curti, Casiraghi, et al. in 2012 (Scheme 230, eq 1).^607^ The authors investigated the potential of 3-alkylidene-2-oxindoles **909** as viable γ-enolizable nucleophiles in conjugate additions to a variety of β-nitrostyrenes **910**. The reaction was orchestrated by the bifunctional cinchona alkaloid thiourea **C39** and delivered almost enantiopure γ-substituted *Z*-configured alkylidene oxindoles **911** with excellent levels of regio- (γ:α > 99:1), diastereo- (>20:1 diastereomeric ratio), and enantioselectivity (>99:1 enantiomeric excess). Provided that *N*-carbamoyl protecting groups within the oxindole substrates were used (Boc, Moc), the reaction scope and generality were remarkable, regardless of the presence of neutral, electron-withdrawing, or electron-donating substituents on the two reaction components. The authors demonstrated that the cooperativity between the basic and the acidic moieties in the bifunctional catalyst was a prerequisite for an efficient chirality transmittal.

One year later, in 2013, the same researchers reported another example of direct vinylogous Michael addition in which the acceptors were again nitroolefins **910**, while the donors were prochiral alkylidene oxindoles of type **912**, thus enabling the simultaneous installation of two stereocenters within the adducts **913** (Scheme 230, eq 2).^608^ Even in this case, the best results in terms of yield and diastereo- and enantioselectivity were obtained with thiourea **C39**, and the reaction furnished only *Z*-configured *anti*-configured products **913**. The investigation of the substrate scope revealed that this vinylogous reaction was general, with the sole limitation that unprotected and *N*-alkyl substituted indoles did not give appreciable results. Based on the experimental evidence and several precedents with bifunctional organocatalysts,^590,609^ two possible models of the dual activation were proposed, in which both the nucleophilic and the electrophilic reaction components were activated by means of the bifunctional catalyst: the thiourea unit could activate the nitroalkene by double hydrogen bonding, while the quinuclidine base deprotonated the oxindole to afford the active dienolate species; an additional hydrogen bond between the carbonyl of the indole *N*-protecting group and the protonated quinuclidine base of the catalyst further contributed to the stabilization of the transition state (Scheme 230), thus ensuring stereocontrol and chirality transmittal from the catalyst to the product. An alternative transition state model involving electrophile activation by the protonated amine group of the catalyst and nucleophilic activation by the thiourea moiety could not be excluded.

Since these contributions, which launched the γ-enolizable alkylidene oxindoles as useful vinylogous donor matrices in asymmetric synthesis, a number of studies appeared dealing with the exploitation of these scaffolds in organocatalyzed vinylogous Michael additions to nitroalkene or α,β-unsaturated carbonyl acceptors which are listed in Tables 10 and 11, respectively.

**Table 10:**
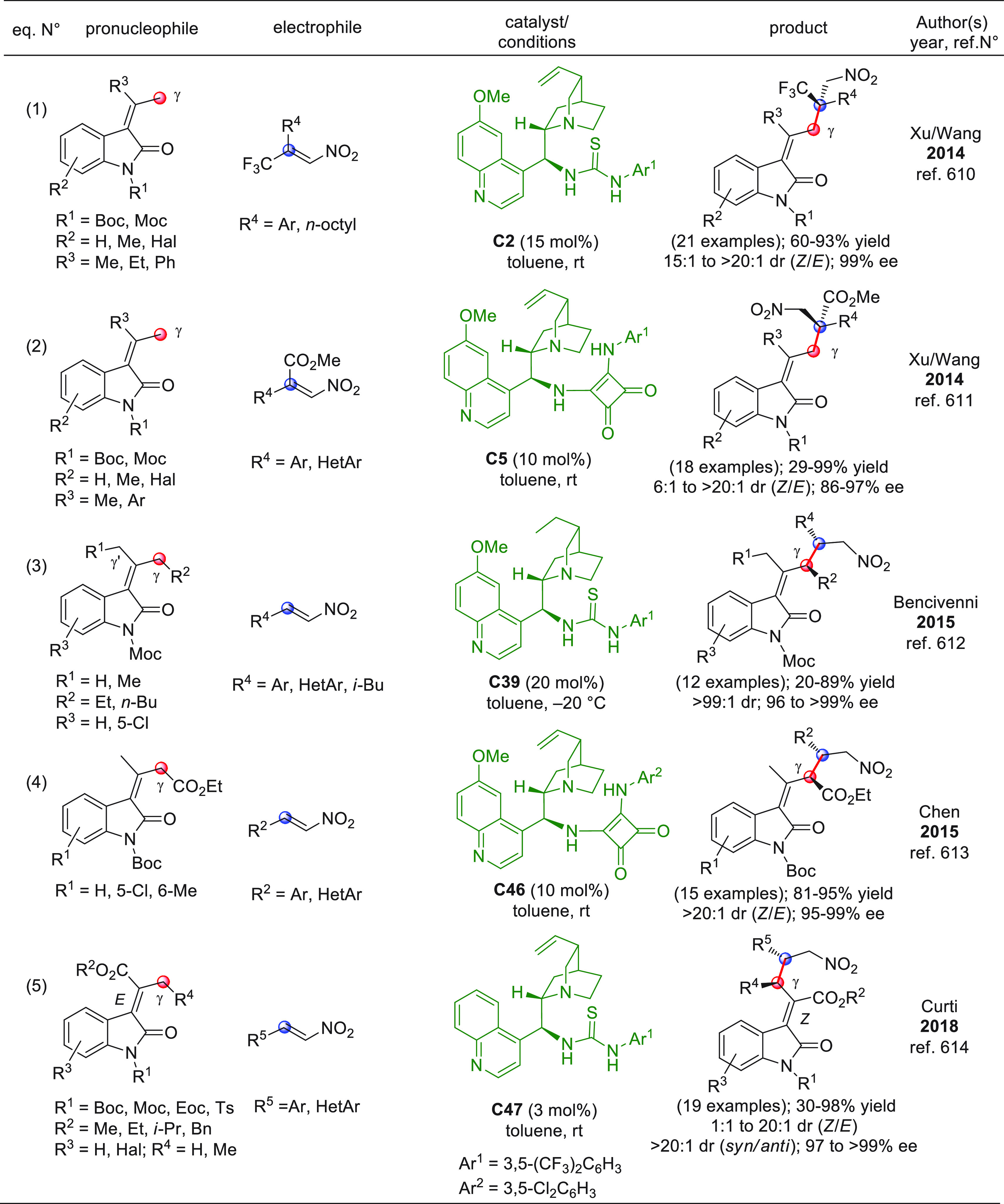
Asymmetric Vinylogous Conjugate Additions of γ-Enolizable 3-Alkylidene Oxindoles to Nitroalkene Acceptors

**Table 11:**
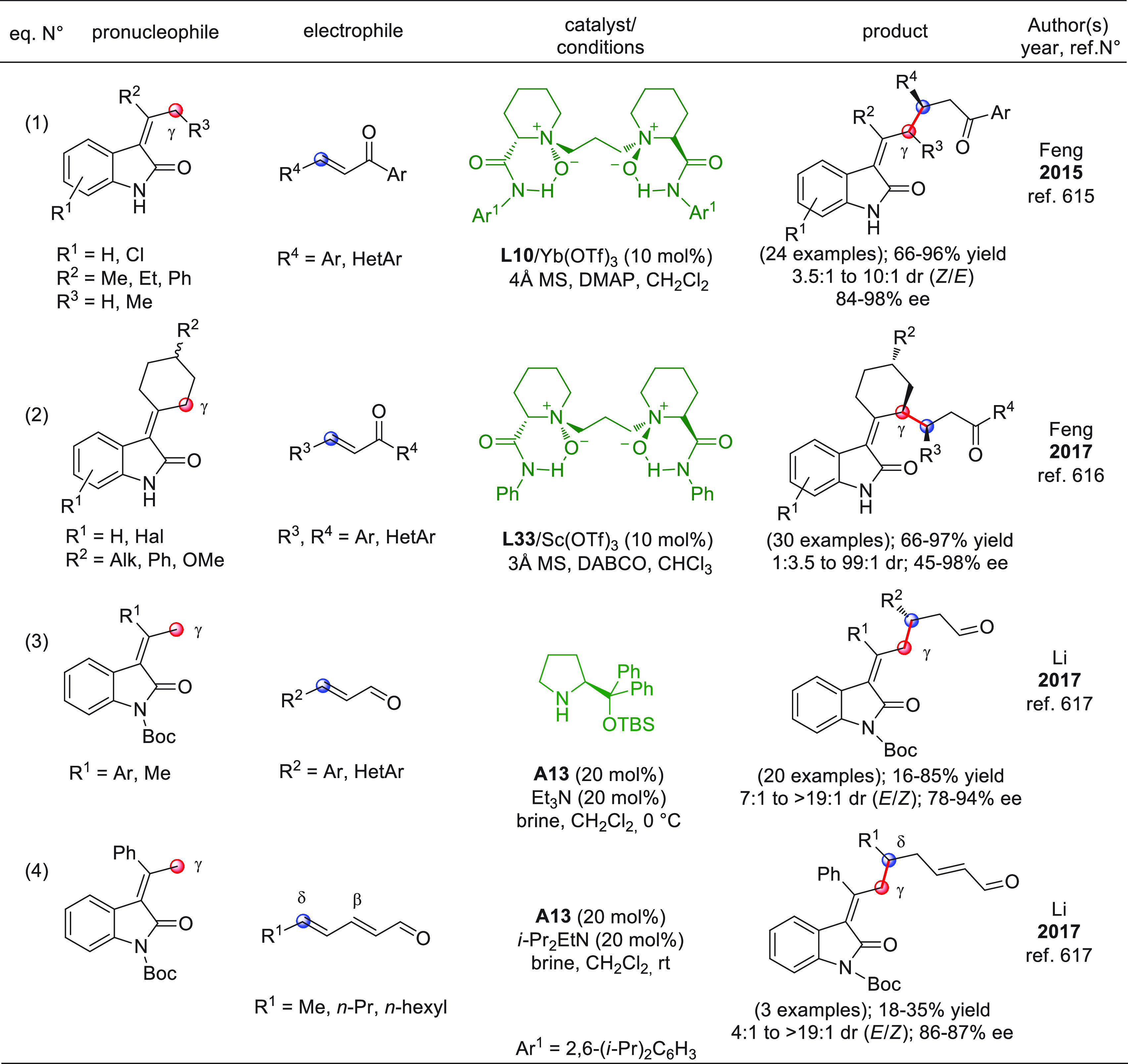
Asymmetric Vinylogous Conjugate Additions of γ-Enolizable 3-Alkylidene Oxindoles to Unsaturated Carbonyl Acceptors

In 2014, Xu, Wang, et al. reported the vinylogous Michael addition reaction between alkylidene oxindoles and β,β-disubstituted nitroolefins catalyzed by bifunctional quinine thiourea **C2** (Table 10, eq 1).^610^ A series of chiral oxindoles bearing trifluoromethylated all-carbon quaternary stereocenters was obtained in good yields, excellent *Z/E* ratio, and enantioselectivity.

The same authors published a further development of the protocol, in which the Michael addition of oxindoles to α-substituted β-nitroacrylates gave oxindole products with a quaternary stereocenter (Table 10, eq 2).^611^ The reaction required the use of quinine-derived bifunctional squaramide **C5**, which was postulated to simultaneously link via H-bonding the nitroalkene moiety and generate the oxindole dienolate by means of the tertiary amine unit.

Another example of direct, vinylogous Michael addition to β-nitroolefins was presented by Bencivenni and collaborators who studied the γ-functionalization of nonsymmetric alkylidene oxindoles having R^1^ ≠ R^2^ (Table 10, eq 3).^612^ The reactions were carried out at −20 °C to suppress the interconversion between *E* and *Z* isomers, and the organocatalyst of choice was the 9-*epi*-hydroquinine thiourea **C39** being able to selectively deprotonate the oxindole γ-position (and not the γ′-site), as confirmed by isotope-effect experiments. The reactions involving *Z*-configured pronucleophiles proved to be completely regiocontrolled and extremely stereoselective, affording the corresponding products as single *Z*-diastereoisomers with excellent enantiocontrol. The chemical yields were generally good, with the exception of the reaction exploiting an aliphatic nitroalkene as acceptor. Similar results were achieved with *E*-configured substrates, providing Z-adducts, although in such cases regio- and stereoselectivity were decreased.

In 2015 Chen and co-workers proposed the dichloro-substituted squaramide catalyst **C46** to promote the asymmetric Michael addition reaction of oxindolinylidene butanoates to nitroolefins (Table 10, eq 4).^613^ The use of catalyst **C46** afforded the best results in terms of yield and diastereo- and enantioselectivity, and the reaction turned out to be efficient and selective with a variety of substituted nitrostyrenes.

Continuing on this theme, recently Curti and colleagues introduced (*E*)-3-(alkoxycarbonyl-2-alkyliden)-2-oxindoles as γ-enolizable pronucleophiles to be used in organocatalyzed Michael additions to nitroolefins (Table 10, eq 5).^614^ After extensive optimization studies, the thiourea **C47** was selected to generate multidentate γ-dienolates that add to nitroalkenes and deliver vinylogous *Z*-configured products with excellent levels of site-selectivity and diastereo- and enantioselectivity. It is noteworthy that the reaction showed an unprecedented *Z*-selectivity, providing chiral alkylidene oxindoles with geometrically inverted C3–C2′ double bonds (the newly formed C–C bonds and the related stereocenters are oriented *trans* to the oxindole carbonyl), as opposed to most of the γ-homologated alkylidene oxindoles mentioned so far. The authors supposed that the ester handle within the oxindole pronucleophiles could play an active role in determining the geometry of the products and reported a mechanistic pathway supported by DFT calculations and accounting for the observed stereochemical outcome. According to their assumption, the alkoxycarbonyl group may function as an additional anchoring point with the catalyst quinuclidine portion stabilizing the *s-trans* conformation of the oxindole dienolate, while the thiourea would activate the electrophile by hydrogen bonding. Moreover, the synthetic utility of the Michael adducts was demonstrated by chemical transformations leading to a quaternary oxindolyl proline analogue and a chiral spyrocyclic furoindolone scaffold.

In 2015, Feng and co-workers reported the first example of direct, asymmetric VMcR of alkylidene oxindoles to α,β-unsaturated carbonyl acceptors (Table 11, eq 1).^615^ The reaction between unprotected oxindoles and chalcones was catalyzed by the ytterbium(III)-*N*,*N*′-dioxide chiral complex **L10**/Yb(OTf)_3_ in the presence of DMAP and molecular sieves. The yields of the reactions were generally high and the enantioselectivity was good, though the *Z*/*E* ratio for many adducts was not impressive. Two years later, the same authors envisaged exploiting a similar *N*,*N*′-dioxide chiral metal complex in vinylogous Michael additions to chalcones aiming at the deracemization of axially chiral cyclohexylidene oxindole compounds (Table 11, eq 2).^616^ In the event, the **L33**/Sc(OTf)_3_ complex served as the catalytic system in the one-step dynamic resolution of the starting unprotected racemic oxindoles, giving Michael products as the only *Z*-isomers in good yields and with high levels of diastereo- and enantiocontrol.

The asymmetric iminium ion activation of enals as electrophilic partners in vinylogous conjugate additions of alkylidene oxindoles was explored by Li and colleagues in 2017 (Table 11, eq 3).^617^ The TBS-protected diphenylprolinol **A13** was chosen as the covalent organocatalyst to access the corresponding chiral Michael products in moderate to good yields and high enantioselectivities. Interestingly, the reaction of phenyl-substituted oxindole to 2,4-dienals resulted in 1,6-addition with complete δ-site preference and acceptable enantioselectivity, although yields turned out to be modest (Table 11, eq 4).

**Scheme 231:**
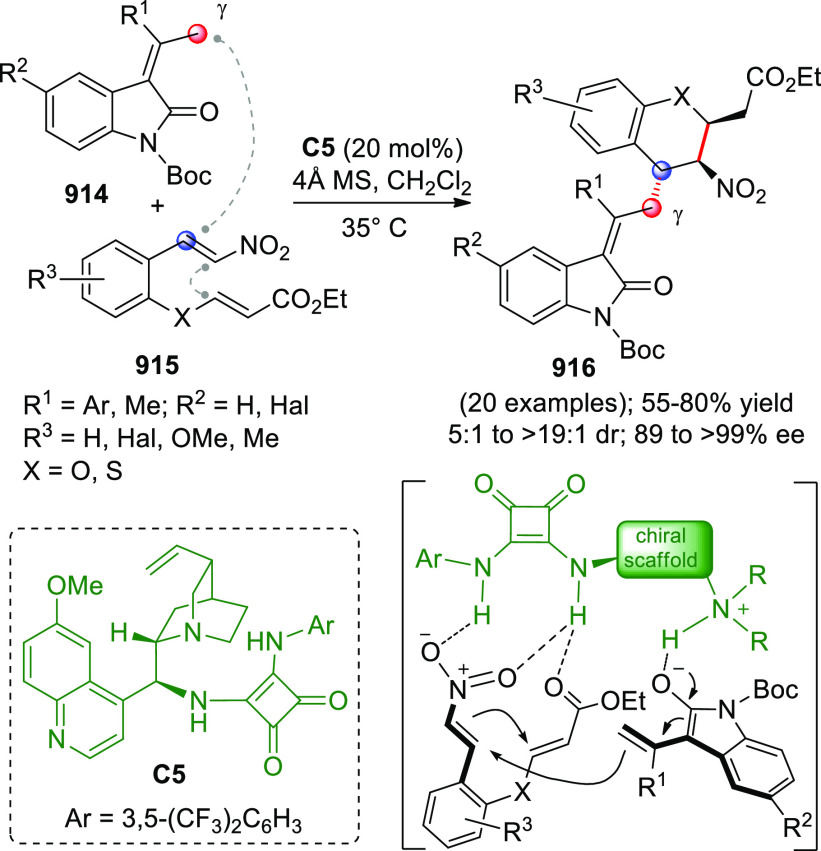


The sole example of an asymmetric annulation cascade involving 3-alkylidene oxindoles **914** and nitroolefin enoates **915** was reported by Feng and Li in 2017 (Scheme 231).^618^ The proposed reaction entailed a sequence of two Michael reactions, a first vinylogous conjugate addition to a nitroalkene moiety, followed by an intramolecular closure on the enoate portion. The procedure led enantioselectively to oxindoles **916** bearing three contiguous stereocenters on the newly formed chroman framework (X = O). The reaction cascade was orchestrated by the bifunctional squaramide **C5**, which was able to simultaneously generate the oxindole dienolate and activate the nitroolefin and the enoate moiety via H-bonding, according to the transition state proposed by the authors (Scheme 231, bottom).

Continuing on the theme of vinylogous indolinone chemistry, the ketimines and nitrones deriving from isatins can be regarded as aza-analogues of 3-alkylidene oxindoles (often these reagents are indicated as aza-alkylidene oxindoles) as they own a C=N bond which behaves as an inherently unsaturated π-system and participates in transferring the electronic properties of the indolinone core to the remote γ-site. Along this line, such isatin derivatives have found applications as precursors of aza-dienolates in vinylogous Michael additions to electron-poor olefins or in reaction cascades involving conjugate addition reactions. Apart from one example reported by Trivedi et al.^619^ in which the benzylimines deriving from isatin produced spirooxindole-pyrrolidines in racemic format (not shown), the remaining examples fall in the field of asymmetric organocatalyzed processes.

**Scheme 232:**
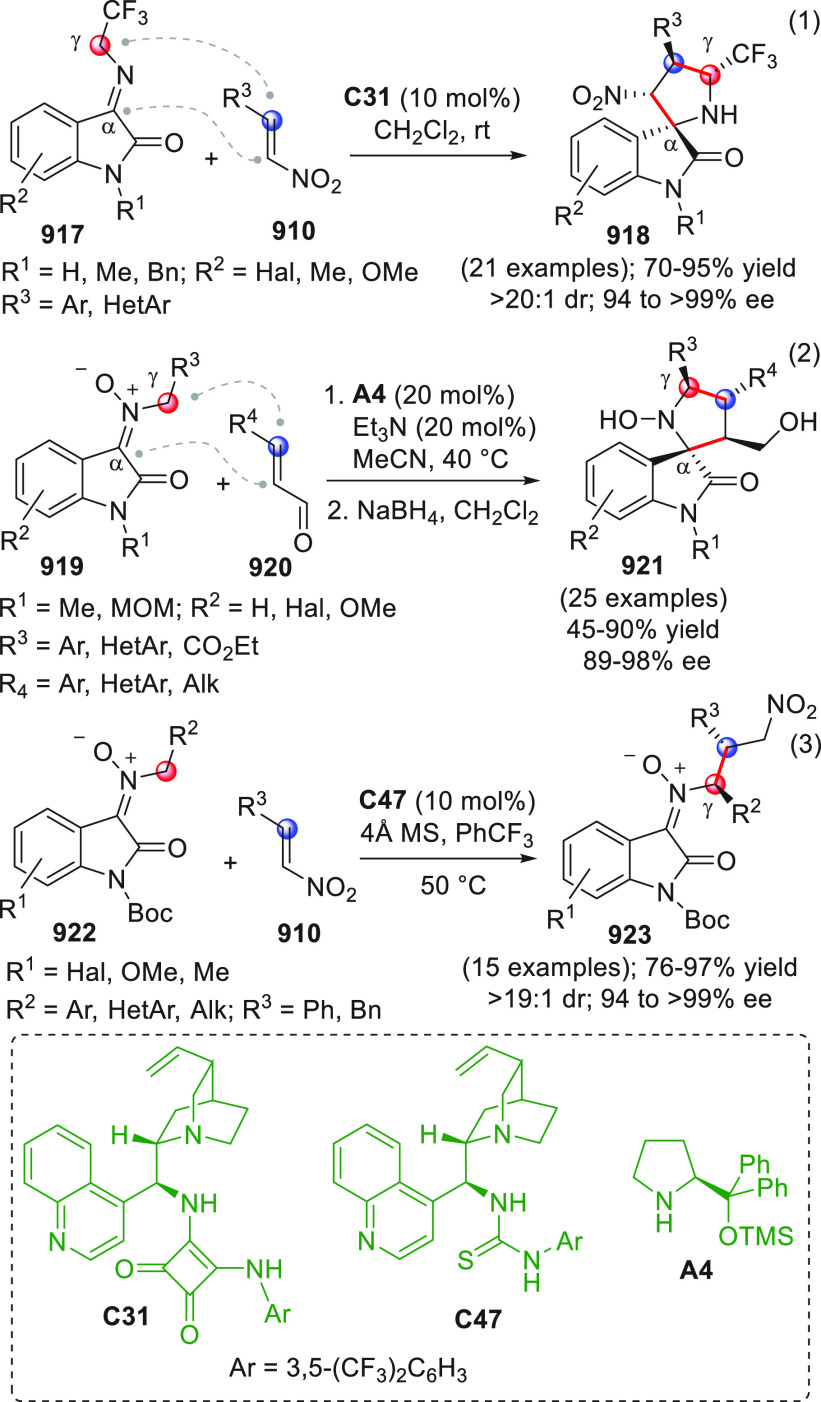


In 2015, Wang and colleagues disclosed that *N*-2,2,2-trifluoroethyl isatin ketimines **917** could be γ-deprotonated by means of bifunctional squaramide **C31** to afford azomethine ylides (azadienolates) and take part to formal γ,α-regioselective [3 + 2] cycloadditions with nitroalkenes **910** (Scheme 232, eq 1).^620^ The scope of both substrates was explored and the reactions produced trifluoromethylated spiropyrrolidine oxindoles **918** in high yields and excellent enantio- and diastereoselectivities. The authors supposed the involvement of the tertiary amine catalyst in the formation and H-bonding of the azomethine ylide and the concomitant activation of the nitroalkene via the squaramide H-bonding, thus ensuring high stereocontrol.

On the other hand, Du, Chen et al. exploited stable and readily available *N*-benzyl nitrones of isatins **919** as precursors of the corresponding ylides in catalytic asymmetric γ,α-regioselective [3 + 2] cycloadditions to enals **920** (Scheme 232, eq 2).^621^ The reactions proceeded smoothly under the assistance of TMS-protected diphenyl prolinol **A4** and a catalytic amount of trimethylamine, giving rise, after reduction with NaBH_4_, to *N*-hydroxy-pyrrolidinyl derivatives **921** in moderate to good yields and considerable enantioselectivity. The detection in the reaction of a Michael adduct intermediate from *N*-benzyl nitrone ylide (R^1^ = Me, R^3^ = Ph) and cinnamaldehyde confirmed the supposed stepwise mechanism for this cycloadditive process. Interestingly, a fine regioselectivity switch toward nonvinylogous α-conjugate addition could be obtained with crotonaldehyde by adding catalytic amounts of suitable metal salts.

Shortly after, the same authors set up an organocatalyzed protocol to perform aza-vinylogous-type Michael additions of nitrones **922** to nitroalkenes **910** (Scheme 232, eq 3).^622^ The reactions were run in the presence of bifunctional cinchonidine thiourea **C47** and molecular sieves, and provided the vinylogous Michael products **923** under almost complete diastereo- and enantiocontrol. It is noteworthy that the Mannich-type cyclization step affording spiropyrrolidine scaffolds did not occur, contrary to the above procedures involving ketimines **917** and nitrones **919**.

As previously mentioned, the Morita–Baylis–Hillman (MBH) carbonates belong to a group of valuable compounds that have been widely exploited as electrophiles in many examples of metal-free asymmetric allylic alkylations^623^ and other substitution reactions with various nucleophiles (vide supra). Although these procedures in most cases lead to overall alkylation products, they indeed proceed through Michael-type additions to electron-poor double bonds of MBH-intermediates, and therefore examples of this chemistry are judged to be nice examples of vinylogous processes and they are legitimately listed in and commented on in this section.

**Scheme 233:**
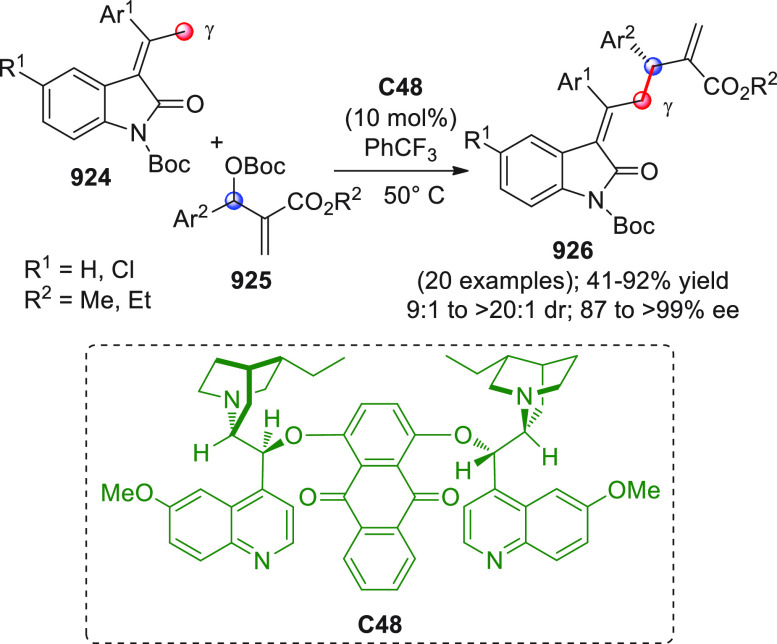


Li and colleagues reported the first example of an asymmetric allylic alkylation involving oxindoles **924** as the pronucleophilic reagents and MBH carbonates **925** as the electrophilic counterparts (Scheme 233).^624^ The reaction was promoted by the chiral bis-cinchona catalyst **C48** activating the MBH carbonates, while the γ-deprotonation of oxindole donor relied on the in situ generated *tert*-butoxy anion released by compounds **925**. The reaction scope was wide, and a series of γ-allyl-substituted alkylidene oxindoles **926** were prepared in moderate to good yields, with excellent enantioselectivity and *Z/E* selectivity.

**Scheme 234:**
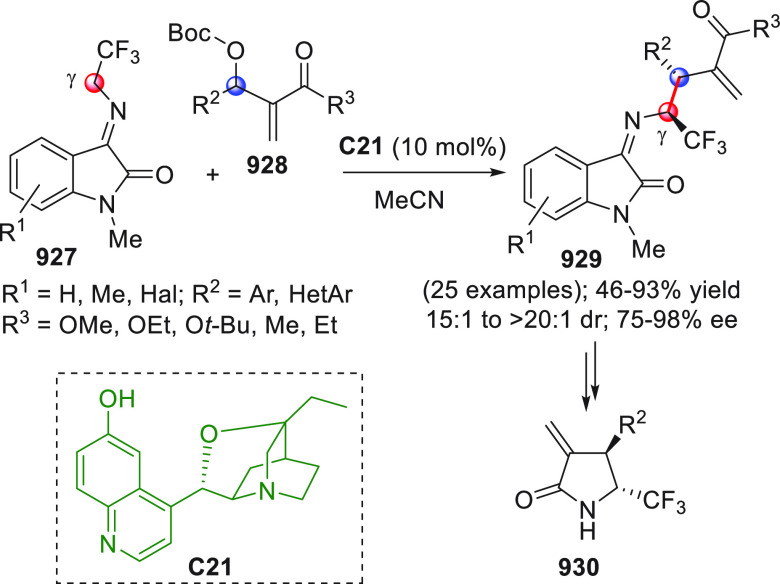


The MBH carbonates **928** also served as competent substrates in the asymmetric allylic alkylation of trifluoroethylisatin ketimines **927**, documented by Wang and collaborators in 2016 (Scheme 234).^625^ The best catalyst promoting the process was the β-isocupreidine **C21** acting as a chiral Lewis base in the generation of the activated electrophiles from compounds **928**; the products **929** were isolated in moderate yields and with remarkable diastereo- and enantioselectivities as a result of the nucleophilic attack of isatin ketimine dienolates to activated double bonds of MBH intermediates. The authors speculated on the possible reaction mechanism describing the process as a sequence of two S_N_2′-type reactions, that ultimately led to the chiral imines **929**, useful precursors of biologically relevant fluorinated α-methylenelactams **930**.

The umpolung of the MBH derivatives toward a nucleophilic reactivity can be obtained by using proper chiral tertiary phosphines or amines as Lewis bases able to form P or N ylide reagents. As demonstrated by Lu and co-workers, the in situ formed ylides from isatin MBH derivatives are capable to participate as 1,3-dipolar synthons in asymmetric [3 + 2] annulations and other cycloaddition maneuvers.^626^

**Scheme 235:**
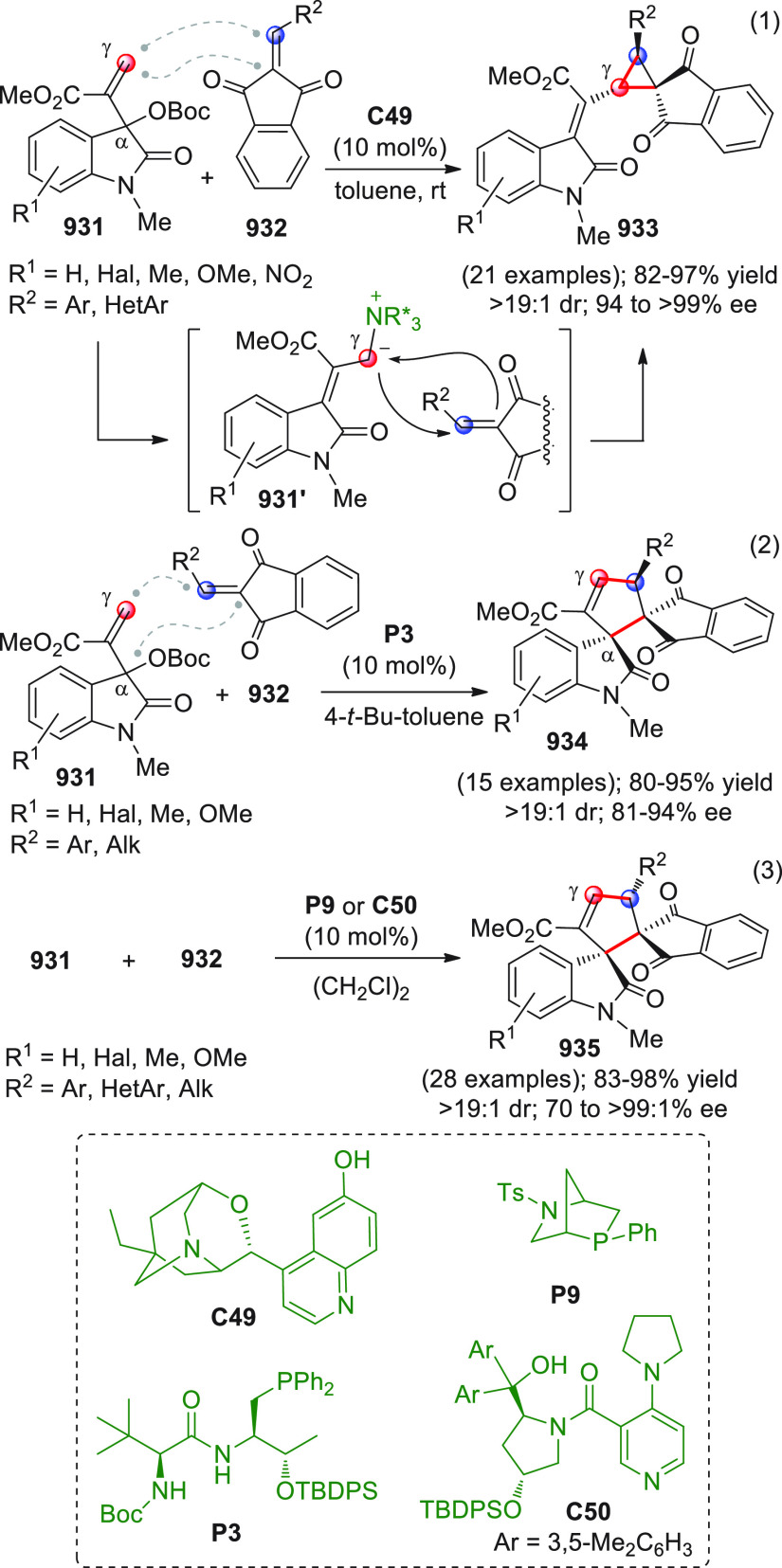


In particular, the potential of MBH carbonates deriving from isatins (and acrylates or acrylonitriles) as vinylogous pronucleophiles in Michael-initiated cycloadditions has been explored by Ouyang, Chen, et al. in 2016 (Scheme 235).^627^ These researchers found that MBH carbonates **931** could alternatively undergo chemo- and stereocontrolled [2 + 1] (Scheme 235, eq 1) or [3 + 2] (Scheme 235, eq 2) annulations with activated alkenes **932**, depending upon the nature of the Lewis base used. The reactions performed in the presence of α-isocupreine **C49** as bifunctional catalyst efficiently provided cyclopropane derivatives **933** (or their enantiomers, if β-isocupreidine catalyst was employed) with high enantiomeric purity as a result of the [2 + 1] annulation (Scheme 235, eq 1). When bifunctional chiral phosphine **P3** was used with the same couple of starting reagents **931** and **932**, a switch in chemoselectivity was observed, and the [3 + 2] annulated spirooxindoles **934** were obtained in excellent yields and good to high enantioselectivity (Scheme 235, eq 2). The diastereoisomeric spirocycles **935** were prepared by using either bicyclic phosphine **P9** or chiral base **C50**, with the latter showing better catalytic activity (Scheme 235, eq 3). DFT calculations were performed to rationalize the different annulation mechanisms and the related stereochemical outcomes; in every case, the first step was recognized as a vinylogous Michael addition of ylides **931′** to alkylidene diones **932**, followed by an S_N_2 attack to the γ-position for the [2 + 1] cyclization (Scheme 235, eq 1); on the contrary, a second Michael reaction involving the oxindole α-position occurred to accomplish the [3 + 2] annulation process.

**Scheme 236:**
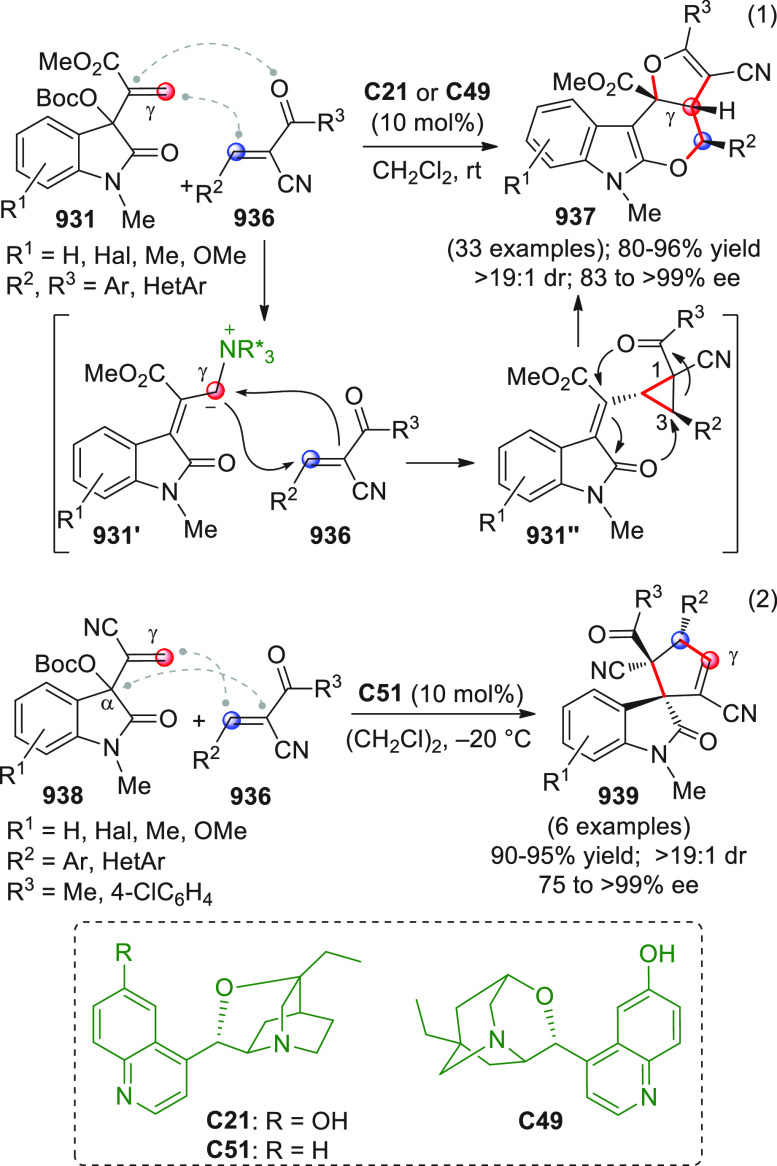


The same researchers also described an unexpected organocatalyzed domino process involving, once more, the isatin-derived MBH carbonates as pronucleophiles with unsaturated α-cyano ketones as activated alkene acceptors (Scheme 236).^628^

In the event, the highly electrophilic α-cyanoketones **936** were reacted with MBH carbonates **931** in the presence of β-isocupreidine **C21**, resulting in fused tetrahydrofuro-pyranoindoles **937** in enantiopure form (Scheme 236, eq 1). On the basis of previous experience with isatin ylides **931′**, the authors supposed the initial generation of cyclopropane intermediates of type **931″** (not detected during the reaction) that subsequently underwent ring-opening, through O-attacking to the cyclopropane C3, followed by intramolecular oxa-Michael ring-closure, ultimately leading to the formation of the polyheterocyclic structures **937**. During the exploration of the substrate scope, it was disclosed that the annulation pathway could switch to a conventional [3 + 2] version producing spirooxindoles **939** (Scheme 236, eq 2) when the MBH carbonates **938** were used under amine **C51** catalysis. The DFT computational calculations suggested that a concerted mechanism for the ring-opening/ring-closure domino reactions was operative, accounting for the diastereocontrolled formation of polycyclic structures **937**; regarding the sequential γ,α-Michael addition cascade, the authors concluded that the introduction of a stronger electron-withdrawing group at the C=C bond was the key to obtain the regioselective formation of spirooxindoles **939**.

**Scheme 237:**
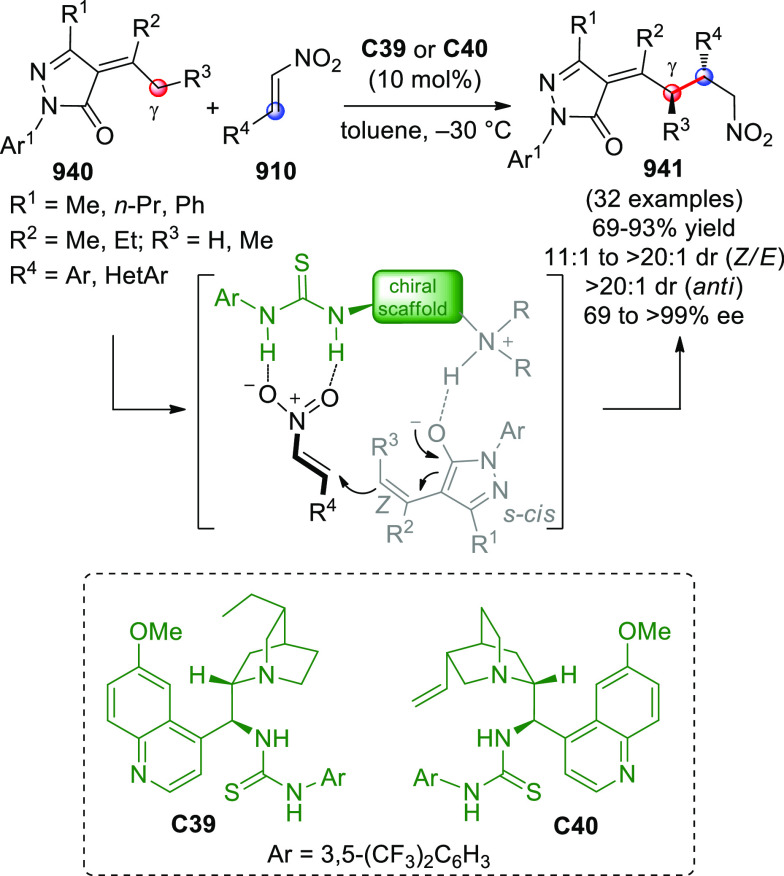


Prompted by the success of 3-alkylidene oxindoles as heterocyclic nucleophilic synthons in asymmetric vinylogous conjugate additions, in 2014 Zanardi, Rassu, and colleagues developed a new asymmetric strategy to functionalize α-alkylidenepyrazolinones by means of organocatalyzed 1,4-additions to nitroalkenes (Scheme 237).^629^ The reaction of variously substituted pyrazolinones **940** with aromatic nitroolefins **910** was orchestrated by quinine-based thiourea organocatalyst **C39** and yielded to the corresponding γ-adducts **941** in high yields, with remarkable levels of enantio- and diastereoselectivities and geometrical selectivities. The pyrazolinones belonging to the enantiomeric series were efficiently accessed by using the *quasi*-enantiomeric quinidine-derived thiourea **C40**. Of note, prochiral pyrazolinone donors **940** bearing different enolizable γ and γ′ positions were competent substrates in the addition reaction giving the *Z-*configured regioisomers, irrespective of the geometry of starting ylidenes **940**.

On the basis of the Michael adduct stereochemistry and chemical correlation experiments, and capitalizing on previous studies on related organocatalyzed vinylogous Michael reactions, the authors proposed a plausible mechanistic rationale entailing the γ-deprotonation of the pyrazolinone by the tertiary amine catalyst and the ion-pair interaction with the resulting dienolate; meanwhile, the thiourea moiety activated the electrophile by H-bonding. Thus, the observed *Z* versus *E* preference would be dictated by the *s-cis* conformation of the dienolate, while the *anti/syn* selectivity would be imparted by the *Z*-geometry of the donor double bond in the stereodetermining step.

**Scheme 238:**
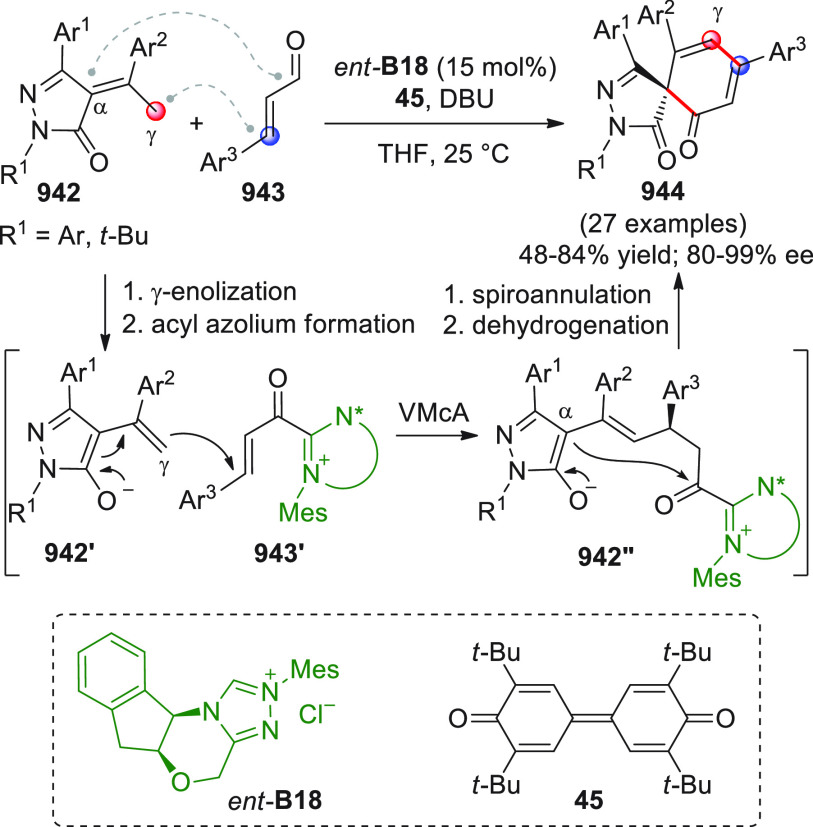


The potential of alkylidene pyrazolinones **942** as vinylogous reagents in conjugate additions was also explored by Biju et al. during a study on the enantioselective synthesis of pyrazolone-fused spirocyclohexadienones **944** under oxidative NHC organocatalysis (Scheme 238).^630^ The authors demonstrated that a variety of pyrazolinones **942** reacted as dienolate donors **942′** with acyl azoliums **943′** generated from NHC salt *ent*-**B18** and variously substituted cinnamaldehydes under oxidative conditions. The reactions were conducted in the presence of DBU as the base and quinone **45** as the oxidant and furnished the spirocyclic adducts **944** bearing an *S*-configured all-carbon quaternary stereocenter in moderate to good yields and good to excellent enantiomeric excess values. A plausible mechanism was postulated for this formal [3 + 3] spiroannulation, which involved a vinylogous Michael addition–spirocyclization–dehydrogenation reaction sequence. Accordingly, the γ-deprotonation of pyrazolone **942** by DBU was followed by the vinylogous intermolecular Michael addition of dienolate **942′** to the chiral acyl azolium ion **943′** leading to an intermediate (not shown) which, upon proton transfer, formed dienolate **942″**; this last, in turn, reacted intramolecularly at its α-position by giving the *C*-acylation. The resulting spiro-compound intermediate formed the spirocyclohexadienone target **944** in the presence of the oxidant.

**Scheme 239:**
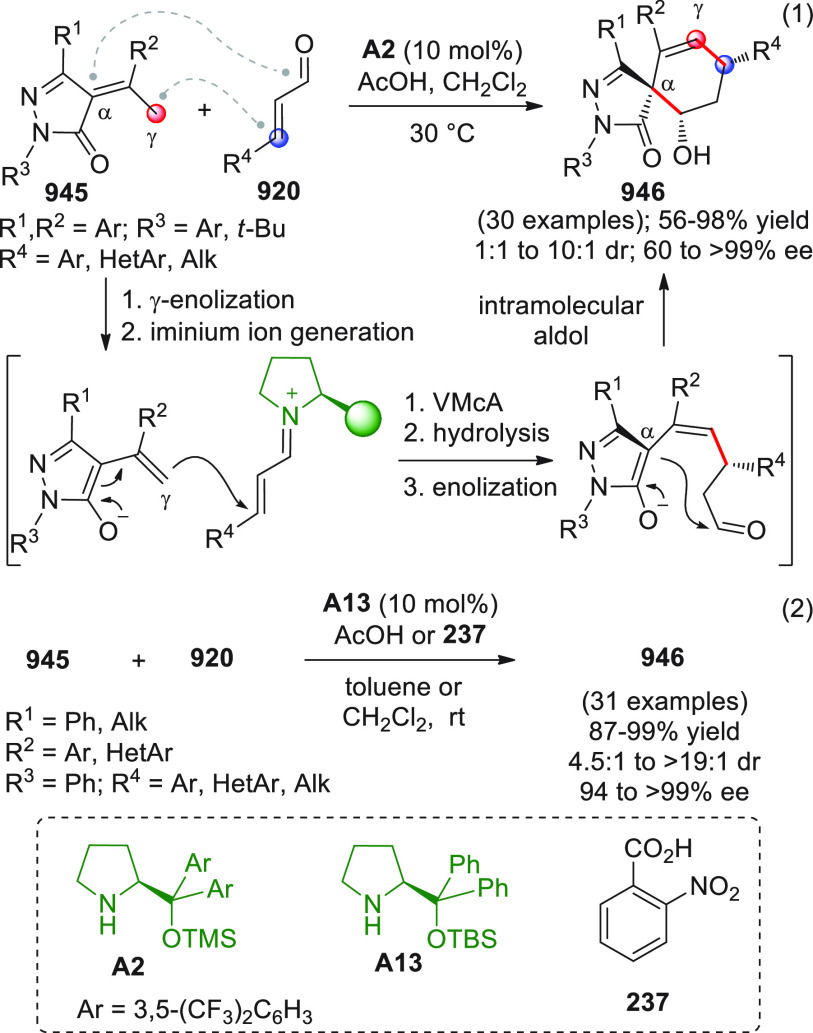


Inspired by the successful exploitation of arylidene pyrazolinones as bisnucleophile species in the synthesis of spirocyclic compounds, the same researchers ventured into the development of a one-pot organocatalyzed synthetic strategy to directly access pyrazolone spirocyclohexenols **946** (Scheme 239, eq 1).^631^ Biju and colleagues envisaged that the dienolate formed from pyrazolinones **945** would intercept the enals **920** activated as iminium ions by the chiral secondary amine **A2**; an intramolecular aldol maneuver would follow, giving rise to the spirocyclohexenols **946** (Scheme 239, eq 1). The authors succeeded in their plan and accomplished the synthesis of spirocyclic pyrazolinone targets in high yield and enantioselectivity, even though with moderate to good diastereoselectivities. A few months later, the Han and Li research group reported a very similar asymmetric γ,α-regioselective [3 + 3] cyclization involving the same pyrazolinones **945** and enals **920** via secondary amine organocatalysis (Scheme 239, eq 2).^632^ They adopted a protocol based on the use of prolinol **A13** as the catalyst, in the presence of acetic acid or *o*-nitrobenzoic acid (**237**) achieving exactly the already reported cyclohexenols **946**, even if with better yields and stereoselectivities.

**Scheme 240:**
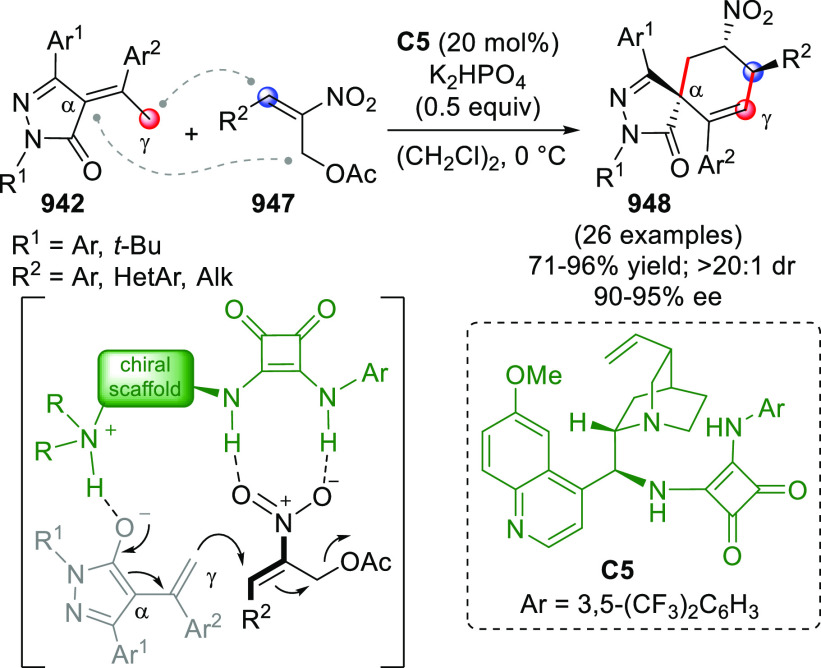


The asymmetric synthesis of spirocyclohexene pyrazolones **948** bearing a nitro-substituent was the aim of the research performed by Xu et al., who exploited the bifunctional squaramide **C5** to catalyze the γ,α-regioselective [3 + 3] cycloaddition reaction (Scheme 240).^633^ The well-known α-arylidene pyrazolinones **942** were selected as the γ,α-binucleophilic synthons to be used in the vinylogous Michael/Michael addition cascade with (*E*)-2-nitroallylic acetates **947**. A series of spirocyclohexene pyrazolones **948** having a quaternary stereocenter were prepared in good yields and excellent stereoselectivities, thanks to the synergistic activation of the vinylogous pyrazolones and the α,β-unsaturated nitroacetates, as illustrated in Scheme 240. According to the proposed catalytic cycle, the Michael attack by the dienolate species forming the first stereogenic center was concurrent to the release of AcOH, and after tautomerization, a second intramolecular Michael reaction provided the final cyclohexene derivatives.

**Scheme 241:**
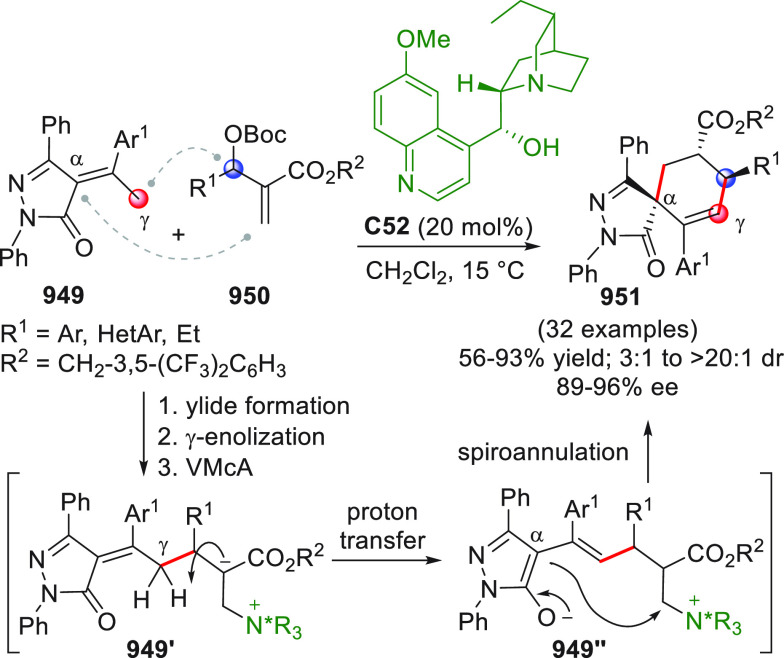


The research group of Guo also developed an asymmetric Lewis base-catalyzed γ,α-[3 + 3] cyclization strategy to access spiropyrazolone scaffolds **951**, based on the exploitation of arylidene pyrazolones **949** as vinylogous donors and MBH carbonates **950** as the electrophilic precursors (Scheme 241).^634^ Here, the *N*-ylides formed from MBH carbonates **950** and the chiral Lewis base **C52** could efficiently γ-deprotonate the pyrazolinones **949** and simultaneously generate the electrophilic Michael acceptors, thus avoiding the need of an additional base. Regarding the substrate scope, various aryl and heteroaryl substituted carbonates **950** proved competent in the reaction and high yields of the products along with good stereoselectivities were observed in most cases. The possible reaction mechanism consisted of the ylide formation, followed by the dienolate generation and the intermolecular vinylogous 1,4-attack affording intermediate **949′** (Scheme 241); after proton transfer, the resulting α-enolate **949**″ completed the ring closure and regenerated the chiral catalyst.

#### 6.3.2 Indirect Procedures

##### 6.3.2.1 Acyclic Nucleophiles

**Scheme 242:**
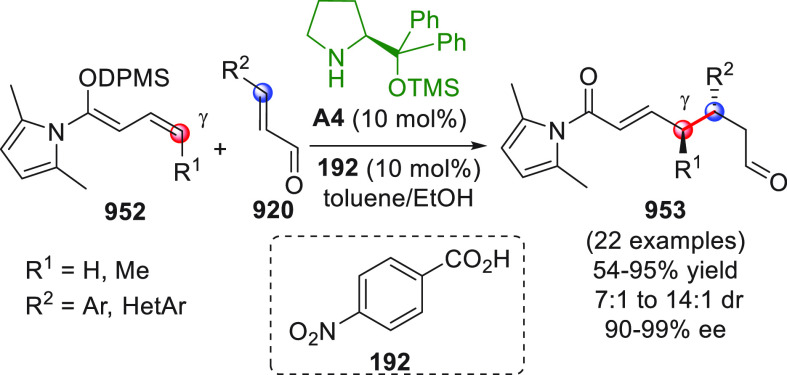


In the period covered by this review the sole example demonstrating the utility of silicon dienolates from acyclic α,β-unsaturated amides in conjugate additions was proposed by the group of Schneider in 2014 (Scheme 242).^635^ These researchers profited from the *Z*-configured vinylketene silyl *N*,*O*-acetals **952** as vinylogous nucleophiles in the conjugate addition to enals **920**. When pyrroles **952** were reacted with enals in the presence of diphenylprolinol silylether **A4**, variable mixtures of α- and γ-regioisomeric products were obtained; however, under optimized conditions, the best γ-regioselectivity was obtained when bulky diphenylmethylsilyl (DPMS)-substituted dienolates **952** were added to a range of enals **920**; the vinylogous adducts **953** were obtained in generally high enantioselectivities when nonprochiral donors were used (**952**, R^1^ = H), while the presence of a γ-methyl appendage within the silyl dienol ethers resulted in moderate diastereopreference in favor of *anti*-stereoisomers.

##### 6.3.2.2 Cyclic Nucleophiles

**Scheme 243:**
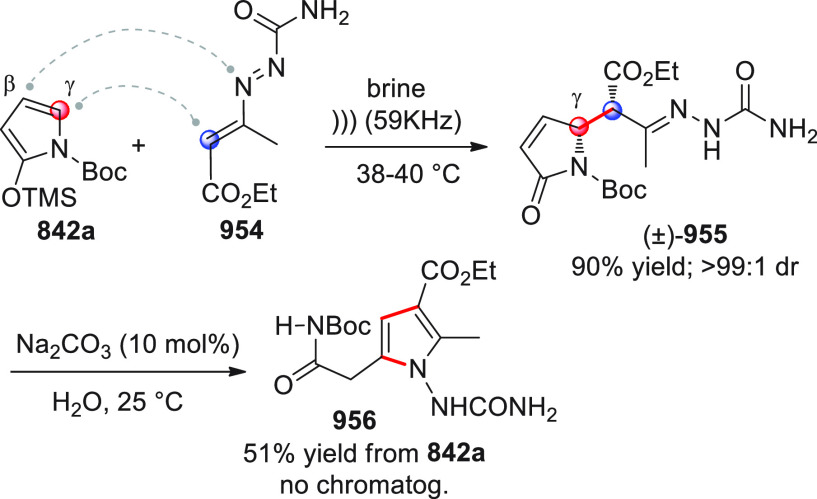


Among the many different transformations centered on the use of the popular silyloxypyrrole **842a** as cyclic nucleophile in Michael reactions, it is worth mentioning the conjugate addition to 1,2-diaza-1,3-dienes of type **954**, reported by Battistini, Zanardi, and colleagues during the synthesis of highly functionalized pyrrole-carboxylates **956** (Scheme 243).^636^

The one-pot three-step reaction cascade leading to the targeted pyrrole was performed in aqueous medium and consisted of an initial water-mediated diastereoselective vinylogous Mukaiyama-Michael coupling of pyrrole **842a** to diazadiene **954**, followed by a base-catalyzed intramolecular aza-Michael attack/ring-opening/aromatization sequence yielding the unsoluble pyrrole-carboxylate **956**. The opening vinylogous Mukaiyama–Michael reaction was tested in either traditional organic solvents (in the presence of Lewis acid) or aqueous media; the crucial role exerted by water as both reaction environment and promoter was demonstrated by the remarkable results obtained in terms of reaction rate, stereoselectivity, efficiency, and general applicability of the protocol. Of note, this on-water procedure was conveniently extended to oxygen- and sulfur-containing nucleophiles with yields and diastereomeric ratios comparable to the nitrogen counterparts (not shown).

**Scheme 244:**
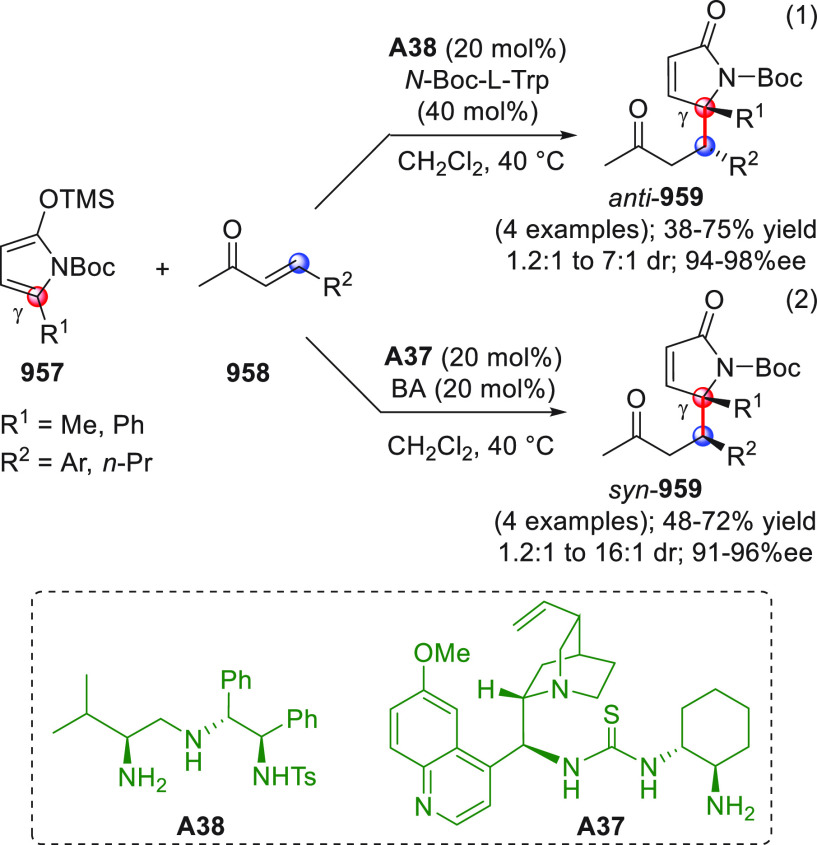


During an extensive study on the diastereodivergent synthesis of γ,γ-disubstituted butenolides by means of organocatalyzed vinylogous Michael reactions (vide supra), Dixon and co-workers examined the possibility to apply this catalyst-controlled stereodivergent strategy to the Mukaiyama–Michael reaction of pyrrole derivatives **957** (Scheme 244).^456^ The authors selected the Boc-protected γ-methyl (or γ-phenyl) silyloxypyrroles **957** as the vinylogous donors and various enones **958** as the acceptors; when the reactions were performed with chiral amine **A38** as the catalyst, *anti*-configured lactams **959** were obtained in moderate yields and excellent enantiomeric excess, albeit with poor diastereoselectivities. The use of thiourea **A37** as the catalytic system provided instead *syn*-configured lactams **959** as the major stereoisomers, with equally good results as for the efficiency and stereocontrol were concerned.

**Scheme 245:**
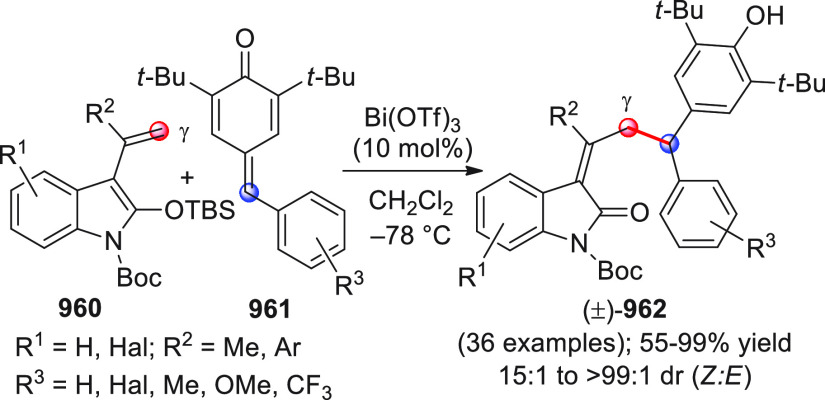


Finally, Li et al. reported the metal-catalyzed 1,6-conjugate addition of silyloxyindoles **960** to *para*-quinone methides **961** (Scheme 245).^637^ The reaction was performed in the presence of catalytic bismuth triflate, providing racemic α-alkylidene-δ-diaryl oxindoles **962** in excellent yields, with complete γ-site selectivity and preference for *Z*-configured isomers.

### 6.4 Other Reactions

In this section examples of vinylogous reactions are grouped which do not enter in the previously discussed additions to C=O, C=N, or activated C=C bonds and involving vinylogous, linear or cyclic, α,β-unsaturated amides (or lactams). Among these contributions, a couple of papers regarded asymmetric γ-selective aminations, alkylation, or acylation procedures, and only one example dealt with a stereoselective halogenation.

#### 6.4.1 Direct Procedures

##### 6.4.1.1 Acyclic Pronucleophiles

**Scheme 246:**
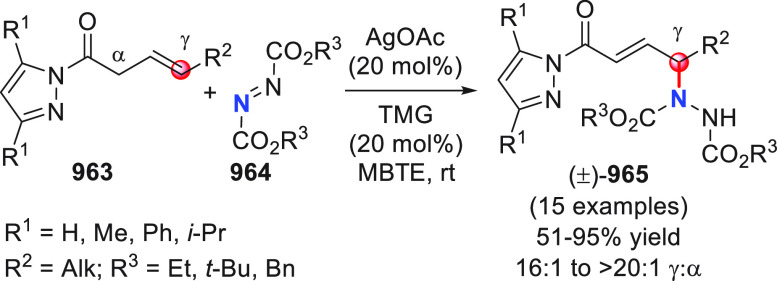


In a recent study, Huang, Zhang, et al. elaborated a metal-catalyzed protocol to regioselectively accomplish the γ-amination of *N*-acylpyrazoles **963** as acyclic pronucleophiles with azodicarboxylates **964** (Scheme 246).^638^ The authors noticed that the choice of the metal catalyst (silver versus zinc) was critical to control the regioselectivity; in fact, the use of a silver acetate/TMG system ensured the formation of racemic γ-aminated products **965** in moderate to good yields, while the alternative use of zinc acetate as catalyst allowed the access to the α-aminated analogues.

##### 6.4.1.2 Cyclic Pronucleophiles

**Scheme 247:**
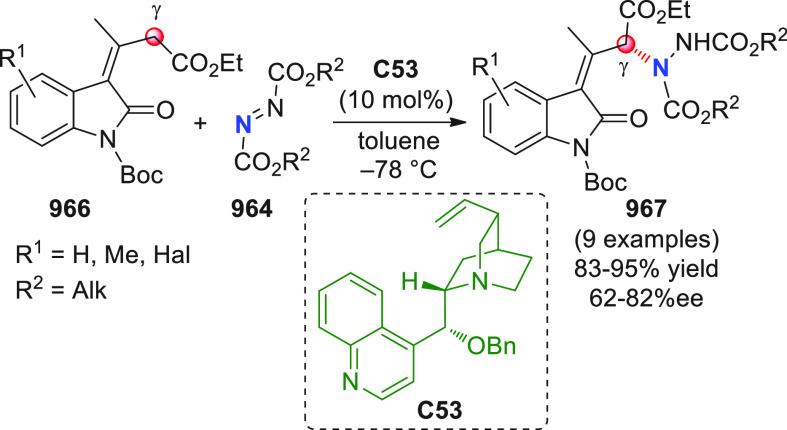


The asymmetric amination of oxindole-derivatives **966** with azodicarboxylates **964** was obtained by Chen and colleagues in 2015 by reacting butanoates **966** with aminating reagents **964** under the assistance of organocatalyst **C53** in toluene (Scheme 247).^639^ The products **967** were prepared in good chemical yields, albeit with moderate enantioselectivities.

#### 6.4.2 Indirect Procedures

##### 6.4.2.1 Acyclic Nucleophiles

**Scheme 248:**
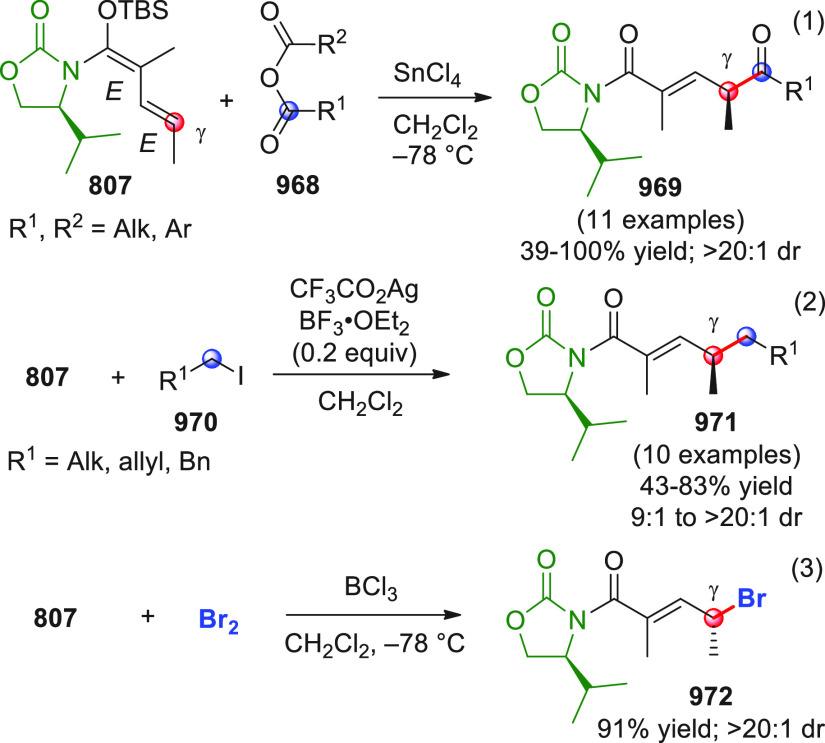


As described in section 6.1.2, the *E,E*-vinylketene silyl *N,O*-acetal **807** was used as the key reagent in *syn*- or *anti*-selective vinylogous Mukaiyama aldol reactions by a large number of researchers. The remote asymmetric induction strategy exploiting this type of reagents was also applied by the Hosokawa group to acylation,^640^ alkylation,^641^ and bromination^642^ reactions (Scheme 248), and the advanced intermediate adducts were successfully utilized in the total synthesis of various natural products.

##### 6.4.2.2 Cyclic Nucleophiles

**Scheme 249:**
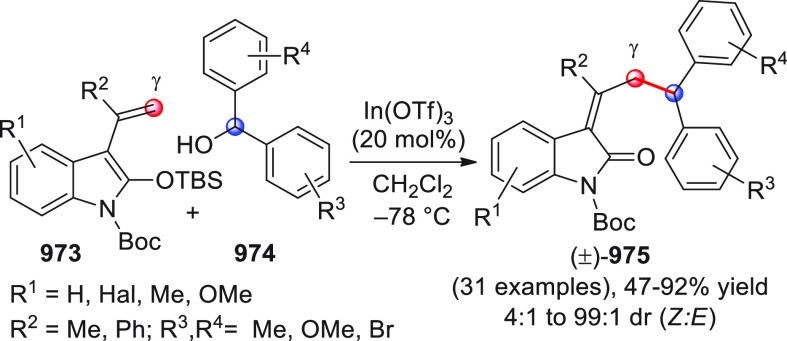


The synthesis of α-alkylidene-δ-diaryl-2-oxindoles **975** was accomplished by Singh et al. through metal-catalyzed vinylogous nucleophilic substitutions of diarylmethanols **974** with alkylidene silyloxyindoles **973** (Scheme 249).^643^

The reactions were catalyzed by indium triflate and proceeded efficiently giving the *Z*-configured γ-alkylated compounds **975**, exclusively. The authors explained the observed *Z*-selectivity by the vinylogous attack of the *s-cis* conformer of silyloxyindoles on the diarylmethane cation intermediates, while steric concerns were invoked to account for the remarkable γ-selectivity.

## 7 Vinylogous Nitriles

**Figure 8:**
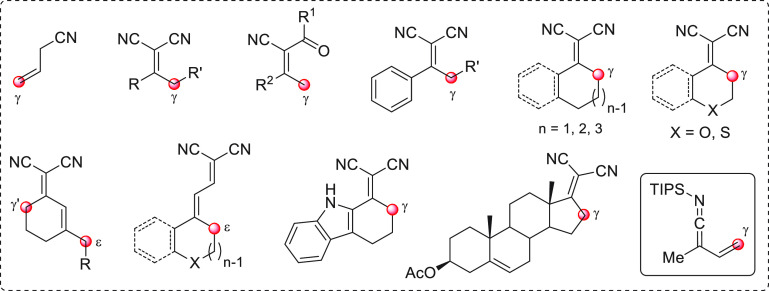
Collection of linear and cyclic pronucleophilic nitriles at work in this chapter using the direct procedures. In the plain box the sole type of nucleophilic nitrile-derived silyl dienol ether used in indirect procedures. Red circles denote the reactive (pro)nucleophilic carbon site.

As can be observed in Figure 8, most of the vinylogous pronucleophile nitriles are γ-enolizable-α,α′-dicyanoalkenes and they have long been exploited in catalytic asymmetric reactions.^28,644^ They are easily prepared by Knoevenagel-type condensation of carbonyl compounds and malononitrile, and the strong electron-withdrawing effect of the two nitrile moieties increases the acidity of the γ-protons, allowing a facile γ-enolization under very mild conditions. For this reason, they are generally more reactive than the corresponding carbonyl precursors and show a remarkable γ-selectivity in vinylogous addition reactions. Moreover, the γ vinylogous addition products may be prone to further manipulation, including cyclization, tandem processes, or even elimination, offering broad maneuvers for the synthesis of cyclic (or polycyclic) diversely functionalized structures via cascade reactions.

In this section, the separation between cyclic and acyclic (pro)nucleophiles is not always sharp, because in many cases methodological studies were carried out on both kinds of structures. The discussion of these works and the corresponding scheme has taken into account the prevailing type of structure in the paper.

### 7.1 Additions to C=O Bonds

#### 7.1.1 Direct Procedures

##### 7.1.1.1 Acyclic Pronucleophiles

**Scheme 250:**
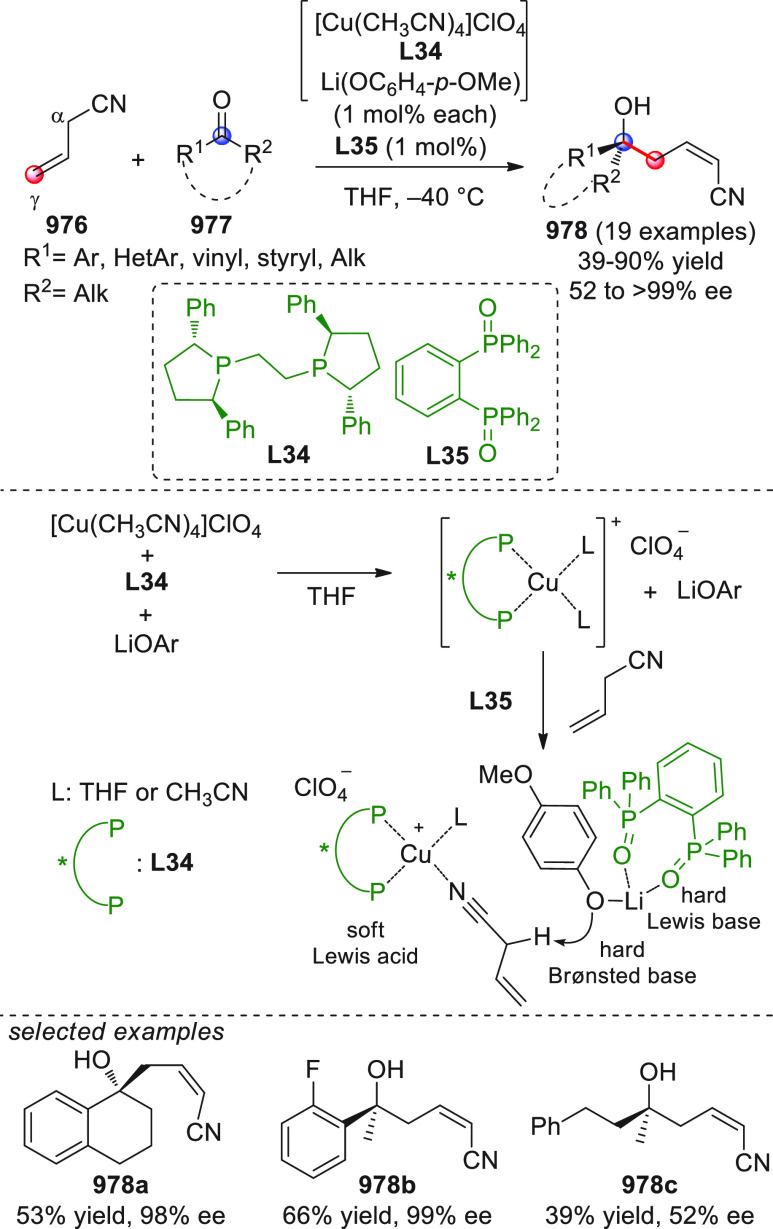


Already in 2002, allylic cyanides were identified as viable pronucleophiles,^645^ bearing an α-proton with an acidity (p*K*_a_ 21.1 in DMSO) suitable for achieving catalyst turnover via proton transfer. α-Selective additions to aldehydes,^645^ aldimines,^646^ and ketimines^647^ were reported, which exploited different activation strategies. However, only in 2009 Shibasaki et al. disclosed the vinylogous nucleophilicity of allylic cyanides **976**, by developing the first direct catalytic and asymmetric addition to ketones, with a complete γ-regioselectivity.^239^ They developed a chiral soft Lewis acid/hard Brønsted base catalytic system consisting of [Cu(CH_3_CN)_4_]ClO_4_/**L34**/LiOAr, to synthesize alcohols with asymmetric tetrasubstituted carbon centers in good yields and enantioselectivities. The following year, the same authors developed a second generation catalytic system, with the addition of a hard Lewis base (**L35**) as a third catalytic component.^240^ The reaction between **976** and ketones **977** (Scheme 250) provided *Z*-configured alcohols **978** in good yields and with generally very high enantiomeric excesses. The bidentate bis(phosphine oxide) **L35** substantially improved the catalytic performance, ensuring higher enantioselectivity in most cases with only 1 mol % catalyst loading. [Cu/**L34**]ClO_4_ serves as a soft Lewis acid and activates **976** through a soft–soft interaction, accelerating deprotonation. The deprotonation step is likely the rate-determining step and Li(OC_6_H_4_-*p*-OMe) is the active Brønsted base used to remove the α-hydrogen. The hard Lewis base **L35** predominantly coordinates Li cations thanks to the hard–hard interaction (P=O···Li^+^) and accelerates the overall reaction rate by enhancing the Brønsted basicity of LiOAr. This cooperative catalysis could be applied only to allylic cyanides without substituents in β- or γ-positions; in fact, the steric factor is critical in preventing the addition to ketones, possibly because of increased steric demands in the transition state. When the ketone substrate possessed two alkyl substituents, the product was obtained in low yield and enantioselectivity (see product **978c**), while when R^1^ was an aryl or a vinyl group, excellent enantiomeric excesses were observed.

Some years later, Shibasaki et al. applied this catalytic strategy to the addition of allylic cyanide to aldehydes.^241^ In this case, variable *anti*/*syn* mixtures of α- and γ-attack products were obtained and with lower enantiomeric excesses. The nature of the aldehyde, the hard Brønsted base, and the reaction time influenced the α/γ ratio. The conditions were optimized and the reaction was applied to the enantioselective synthesis of a key intermediate of fostriecin,^648^ a naturally occurring molecule with antitumor and antibiotic activity. As a corollary of these studies, in 2014 the same authors published a paper in which substrates of type **978** were directly converted to δ,δ-disubstituted unsaturated δ-valerolactones via a one-pot three-step sequence (not shown).^649^

##### 7.1.1.2 Cyclic Pronucleophiles

While cyclic α,α-dicyanoalkene pronucleophiles have been largely used as vinylogous donors in Michael addition reactions (vide infra, section 7.3.1.2), their reactivity in aldol additions has been poorly investigated. Moreover, the two examples reported in these years are nonasymmetric procedures toward racemic compounds.

**Scheme 251:**
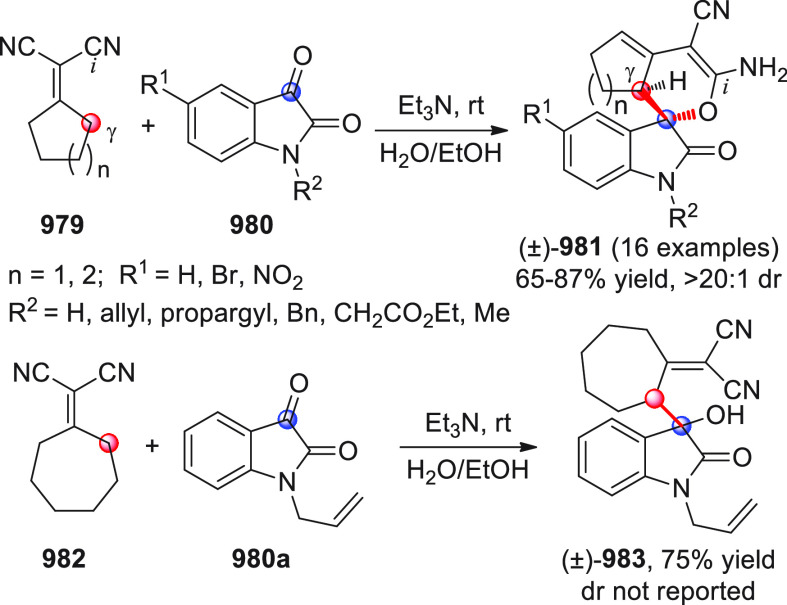


**Scheme 252:**
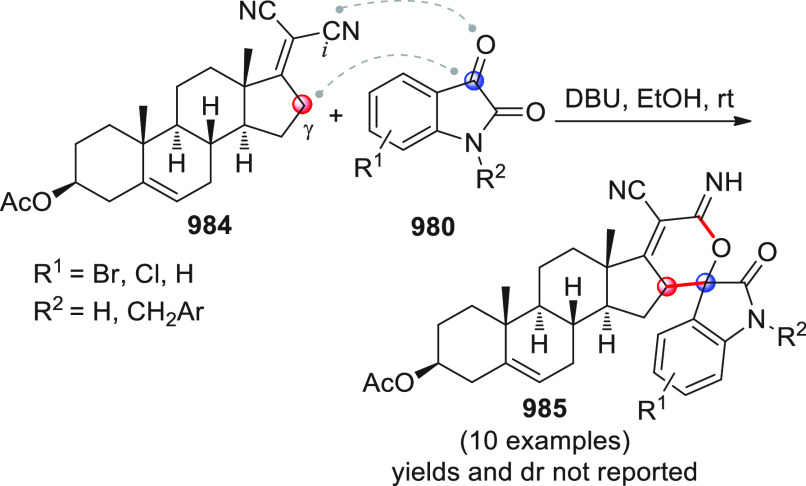


In 2010, Perumal et al. described the one-pot synthesis of functionalized spirooxindoles **981** via base-promoted vinylogous aldol addition/cyclization of cycloalkylidene malononitriles **979** with isatin derivatives **980** (Scheme 251).^650^ Products **981** were isolated in good yields as single diastereoisomers. Instead, when the seven-membered dicyanoolefin **982** was used, the reaction provided the aldol product **983** as a diastereoisomeric mixture, which did not undergo cyclization. This methodology was used by Shan et al. to prepare novel steroidal pyran-oxindole hybrids of type **985** (Scheme 252),^651^ with the aim of obtaining potent and selective cytotoxic agents.

#### 7.1.2 Indirect Procedures

##### 7.1.2.1 Acyclic Nucleophiles

**Scheme 253:**
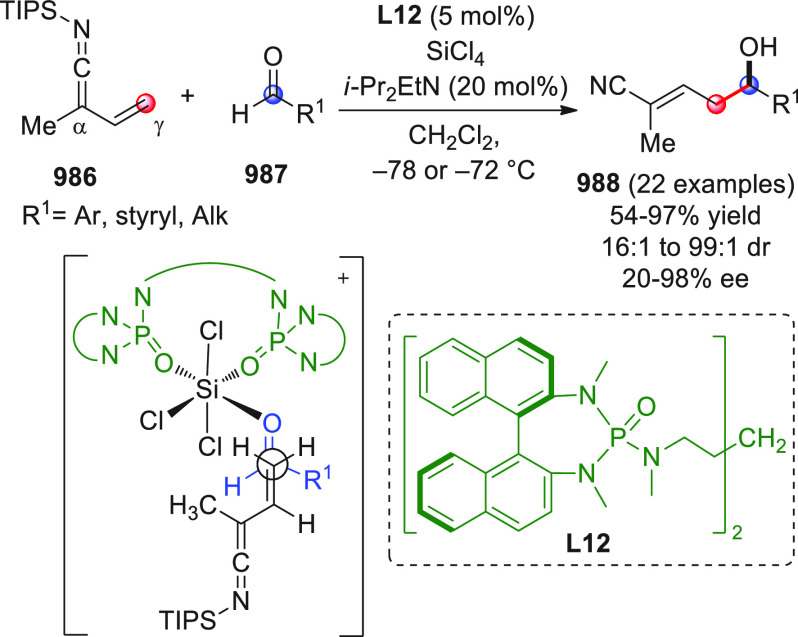


Vinylogous additions of allylic nitrile to aldehydes were reported for the first time by Denmark and Wilson in 2012.^652^ Aliphatic aldehydes can readily undergo base-mediated self-condensation reactions, limiting the possibility to use basic reaction conditions, normally used in direct reactions to generate the enolate species. To overcome this problem, Denmark proposed the alternative strategy to preform the nucleophile species, the *N*-silyl vinylketene imines of type **986** (Scheme 253), by selective *N*-silylation of allylic nitrile anions. Compounds **986** were used as nucleophiles in enantioselective vinylogous aldol reactions with aldehydes **987**, to generate δ-hydroxy α,β-unsaturated nitriles **988**. The reaction was catalyzed by the Lewis base **L12**/SiCl_4_ complex and furnished *E*-configured products with high γ-regioselectivity (up to >97:3 γ:α), in moderate to good yields and with good to excellent enantioselectivities. With aromatic aldehydes, the catalyst loading could be lowered to 2.5 mol %. The γ-attack selectivity was mainly ascribed to the less steric encumbrance of the γ-site with respect to α-carbon atom of the ketene imine **986**, while the absolute and relative configurations of the products were explained by the addition of the *N*-silyl vinylketene imines **986**, in the *s*-trans conformation, to the *Re* face of the aldehyde (Scheme 253, left).

### 7.2 Additions to C=N Bonds

#### 7.2.1 Direct Procedures

##### 7.2.1.1 Acyclic and Cyclic Pronucleophiles

**Scheme 254:**
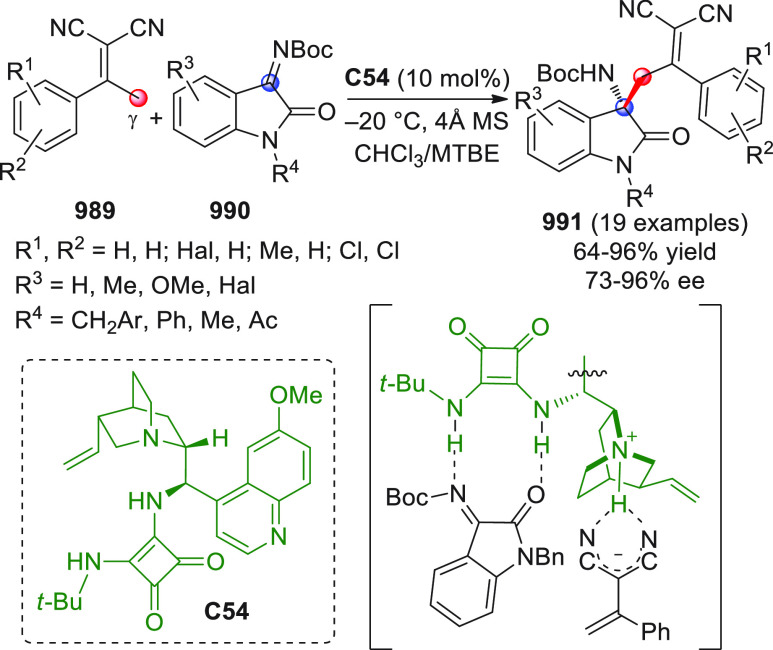


Even if an organocatalytic, asymmetric vinylogous Mannich reaction (VMnR) of α,α-dicyanoolefins to aldimines was successfully realized by Chen in 2007,^653^ the analogous reaction with ketimines was developed only in 2016 by Meng, Li, and co-workers (Scheme 254).^654^ Ketimines, in fact, are more stable than aldimines, and this makes their use as acceptors more challenging. Isatin *N*-Boc ketimines, however, are still good electrophiles because of the electron-withdrawing *tert*-butoxycarbonyl group, and they have been largely used in synthetic procedures, even because they furnish the oxindole backbone, an important structural motif in biologically and pharmacologically active compounds. The reaction between α,α-dicyanoolefins **989** and ketimines **990** was catalyzed by squaramide derived catalyst **C54**, to provide chiral 3,3′-substituted oxindoles **991** in good yields and good to excellent enantioselectivities. Different *N*-protecting groups (R^4^) were tolerated, even if the best results were obtained with benzyl groups. Finally, two cyclic α,α-dicyanoolefins were also investigated, and they afforded the desired products in good yields and enantioselectivities, but with almost no diastereoselection. A drawback of this strategy is the long reaction times, 3 to 8 days depending on the substrates. A potential transition-state structure was proposed by the authors (Scheme 254), in which the tertiary amine of the catalyst deprotonates the α,α-dicyanoolefin which holds, through coordination, the enolate-like structure in close proximity to the isatin *N*-Boc ketimine, in turn activated by N–H binding by the squaramide moiety.

**Scheme 255:**
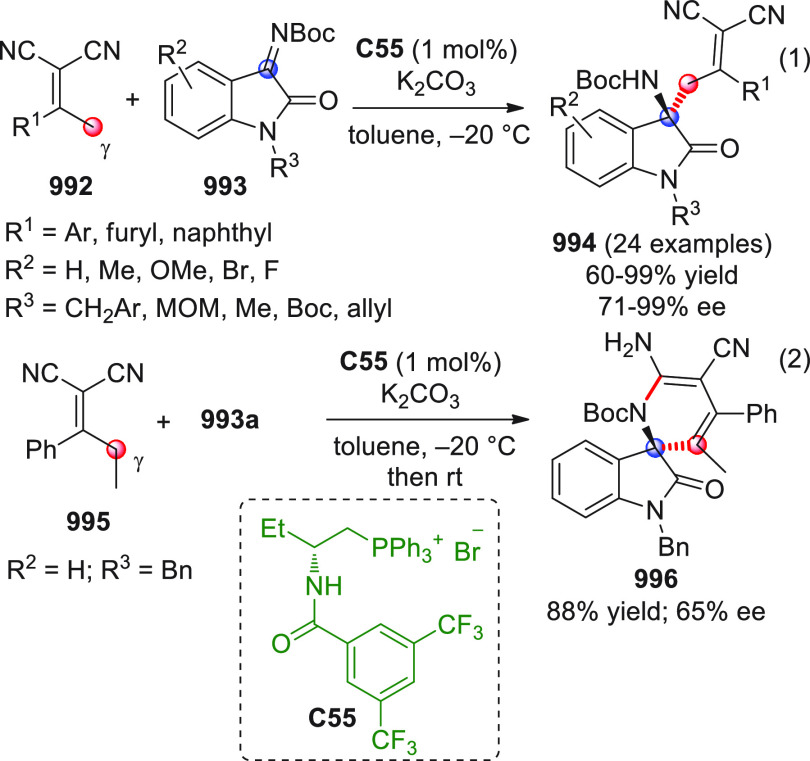


Some months later, Chen et al. developed the same asymmetric VMnR between α,α-dicyanoolefins **992** and *N*-Boc isatin imines **993**, with a different catalytic activation mode (Scheme 255, eq 1).^655^ The reaction was performed in toluene at −20 °C, with bifunctional amide phosphonium salt **C55** (1 mol %) as a phase transfer catalyst and K_2_CO_3_ as a base. In these conditions, adducts **994** formed with very good yields and good to excellent enantiomeric excesses. Interestingly, the authors optimized the conditions for the removal of the malonic nitrile by treatment of the products **994** with KMnO_4_, obtaining the corresponding ketones; for this reason, they proposed these α,α-dicyanoolefins as surrogates of less reactive aryl methyl ketones. When α,α-dicyanoolefins derived from propiophenone (**995**), methyl *tert*-butylketone, or tetralone (not shown) were used, the reaction led to cyclized products of type **996** via a VMnR/intramolecular cyclization cascade sequence (Scheme 255, eq 2).

**Scheme 256:**
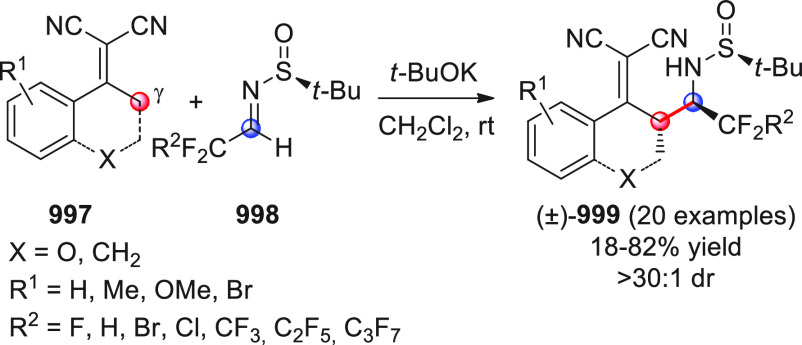


The last example of an asymmetric VMnR with α,α-dicyanoolefins as vinylogous nucleophiles was reported by Fustero, Pozo, et al. in 2018.^656^ Linear and cyclic pronucleophiles **997** reacted with chiral enantiopure fluorinated sulfinyl imines **998** under basic conditions to furnish compounds **999** as single diastereomers in low to good yields (Scheme 256). To account for the observed relative configuration of the products, the authors assumed that the reaction proceeds through a chelated transition state, where the potassium counterion in the enolate would form a chelate with the imine nitrogen atom in a chairlike modality, thus facilitating the attack of the nucleophile to the *Re* face of the imine.

### 7.3 Conjugate Additions to Electron-Poor C=C Bonds

#### 7.3.1 Direct Procedures

##### 7.3.1.1 Acyclic Pronucleophiles

**Scheme 257:**
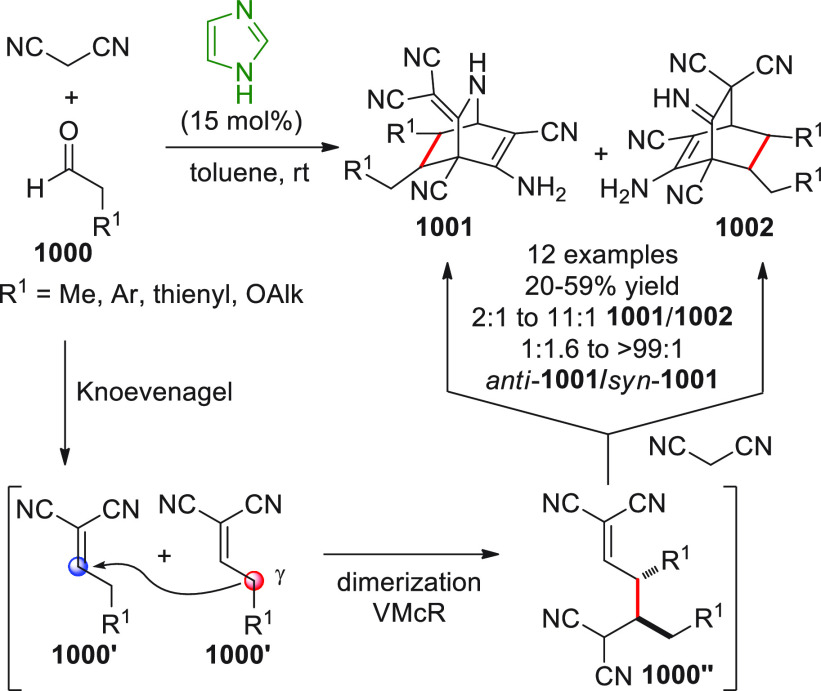


In 2016, Tsogoeva et al. reported the unexpected discovery of an atom-economical metal-free domino transformation, which employed malononitrile and enolizable aldehydes **1000** in the presence of imidazole (15 mol %) and led in a single operation to complex compounds such as isoquinuclidine derivatives **1001** and their isomeric carbobicycles **1002**, bearing an exocyclic imine group (Scheme 257).^657^ A first Knoevenagel reaction furnished α,α-dicyanoolefins **1000′**, that underwent dimerization via VMcR giving intermediate **1000′′**. The reaction of **1000′′** with the excess of malononitrile could follow two pathways, well elucidated by the authors, to provide either compounds **1001** or **1002**, in low to moderate yields. When R^1^ in the aldehyde was an aromatic ring, the major products **1001** were isolated with excellent diastereoselectivities (>99:1), while when R^1^ was the methyl group, the lowest yield and diastereoselection of the panel compounds were registered.

**Figure 9:**
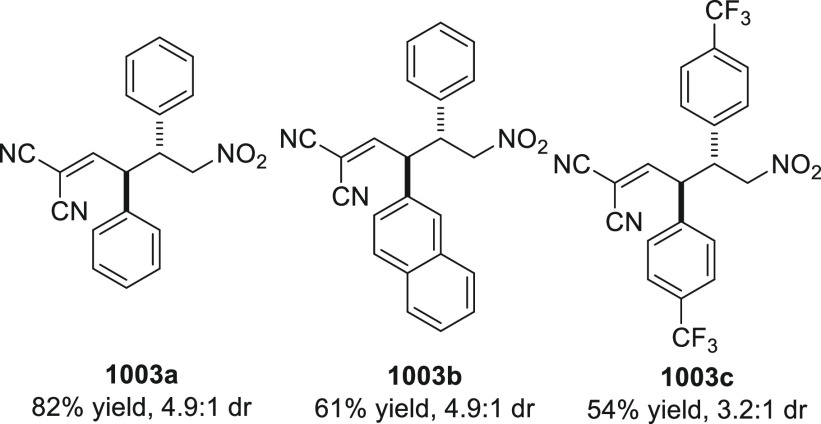
Representative compounds prepared via three-component Knoevenagel/vinylogous Michael domino reaction between arylacetaldehydes, malononitrile, and *trans*-β-nitrostyrenes.^658^

Tsogoeva and colleagues exploited the same imidazole-catalyzed procedure for a three-component Knoevenagel/vinylogous Michael domino reaction of arylacetaldehydes, malononitrile, and *trans*-β-nitrostyrenes.^658^ Nitrostyrenes were able to intercept the in situ generated α,α-dicyanoolefins **1000′** to form the corresponding Michael adducts (such as **1003a**–**c** in Figure 9) in good yield but with modest diastereoselection.

**Scheme 258:**
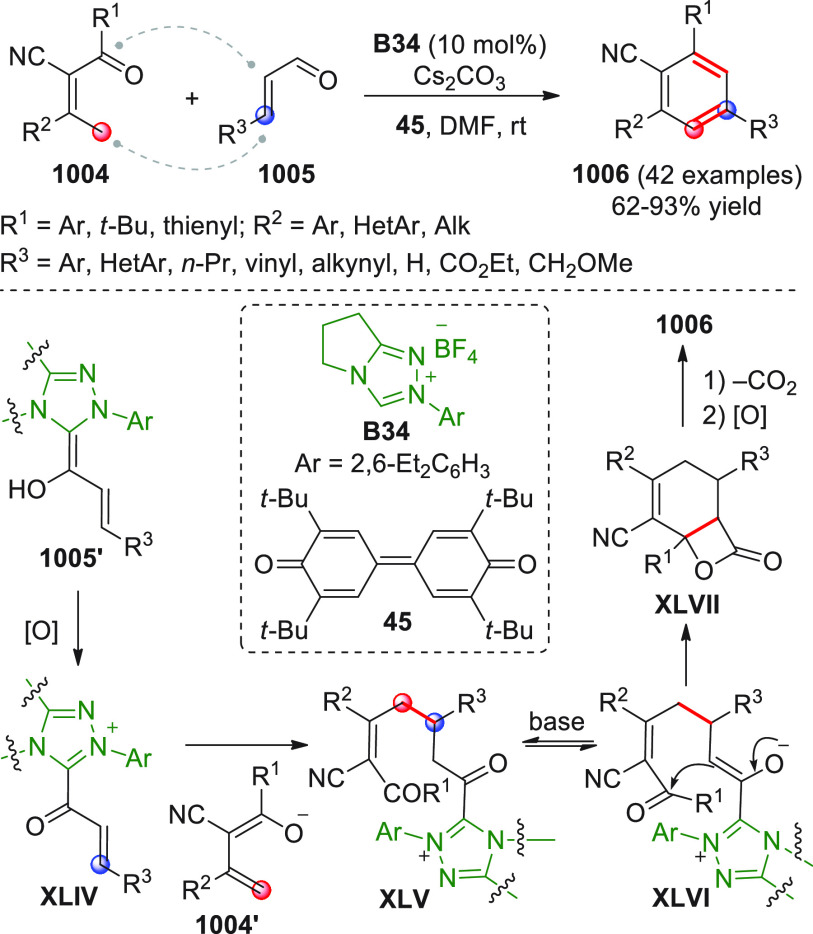


**Scheme 259:**
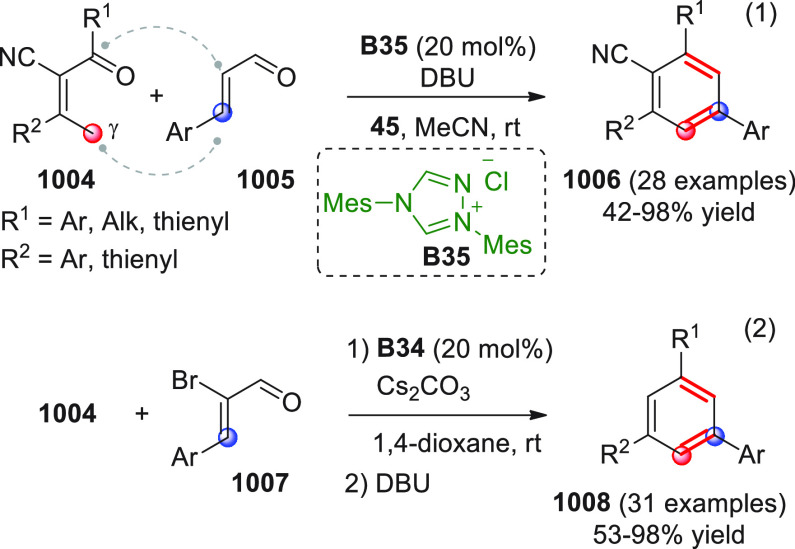


In 2016, the Wang and Ye groups, independently, developed a mild and convenient strategy, an NHC-catalyzed formal [4 + 2] benzoannulation, for the synthesis of multisubstituted benzonitriles **1006** (Scheme 258 and Scheme 259 eq 1, respectively).^659,660^ The reaction mechanism is described in Scheme 258. The addition of the NHC catalyst to enal **1005** followed by deprotonation provides intermediate **1005′**, which is oxidized by diphenoquinone **45** to intermediate **XLIV**. Vinylogous attack of γ-deprotonated α-cyano-β-methylenone **1004′** to **XLIV** furnishes intermediate **XLV** that, after an intramolecular aldol reaction and β-lactonization, yields the bicyclic compound **XLVII** and liberation of NHC catalyst. Decarboxylation of **XLVII**, followed by spontaneous oxidative aromatization affords benzonitriles **1006**. The protocol is tolerant toward a wide range of substituents at the R^2^ position, while decreased, but still acceptable, yields were registered when R^1^ was an alkyl group.

A variant of this reaction was developed using bromoenals **1007** in place of enals (Scheme 259, eq 2).^661^ The reaction was performed without the presence of an oxidant such as **45**. In fact, the formation of the intermediate of type **XLIV** (Scheme 258) was assured by the bromide elimination, while after the decarboxylation of intermediate of type **XLVII**, the DBU-promoted elimination of cyanide furnished 1,3,5-trisubstituted benzenes **1008** in good to excellent yields.

**Scheme 260:**
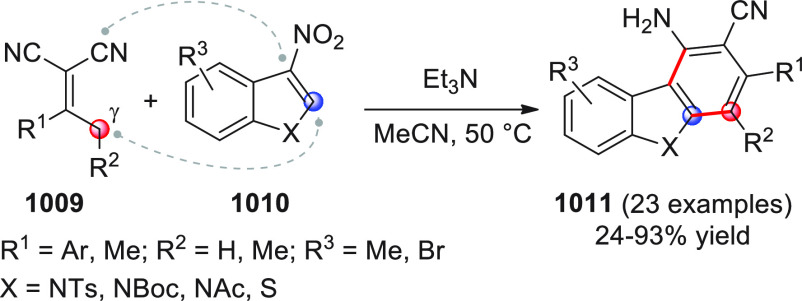


The methodology reported by Yang and co-workers for the construction of substituted carbazol-4-amine derivatives **1011** exploited the vinylogous addition of alkylidene malononitriles **1009** to 3-nitroindoles **1010**, followed by cyclization/isomerization/elimination reactions (Scheme 260).^662^ This base-activated one-pot procedure provided carbazolamines **1011** in moderate to good yields. The reaction was performed in an achiral environment because the aromatization step leads to the loss of the stereogenic centers.

**Scheme 261:**
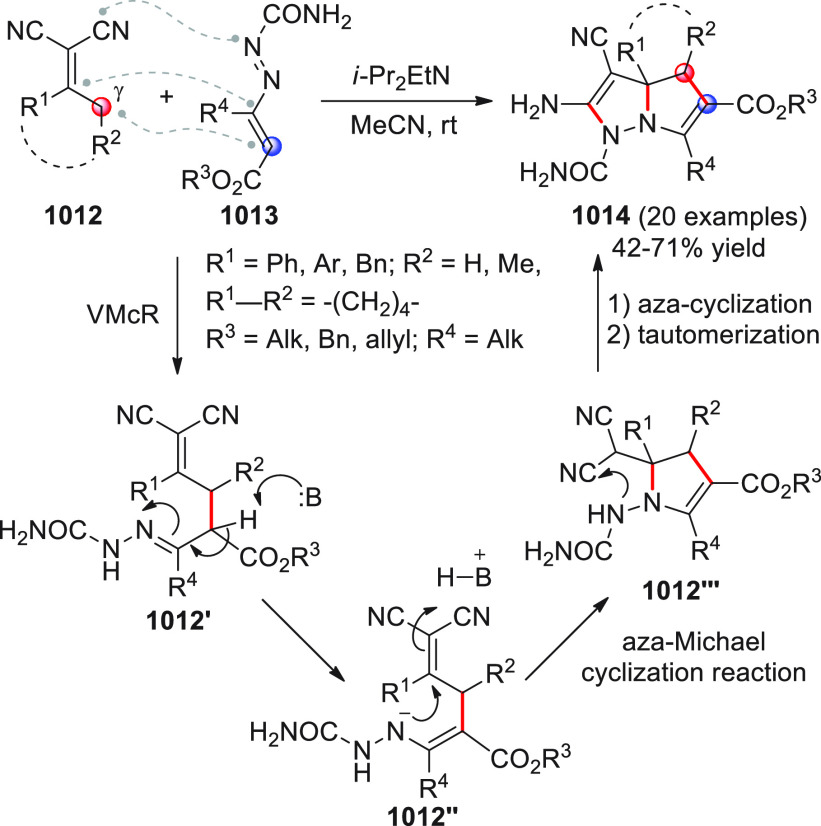


Another example of a base-mediated procedure in an achiral environment is the synthesis of densely functionalized pyrrolo-pyrazole systems **1014** via domino reaction of both linear and cyclic vinylmalononitriles **1012** with 1,2-diaza-1,3-dienes **1013** proposed by Favi and collaborators (Scheme 261).^663^ A plausible mechanism was provided by the authors. The overall transformation would involve at first a VMcR of **1012** to diazadienes **1013** to give intermediates **1012′**. The aza-Michael cyclization of the azaallylic anion intermediate **1012′′**, followed by an aza-cyclization and subsequent imine-enamine tautomerization would furnish adducts **1014** in moderate to good yields with high levels of chemo- and regioselectivity. An equal number of linear and cyclic alkylidene malononitriles were tested.

##### 7.3.1.2 Cyclic Pronucleophiles

**Figure 10:**
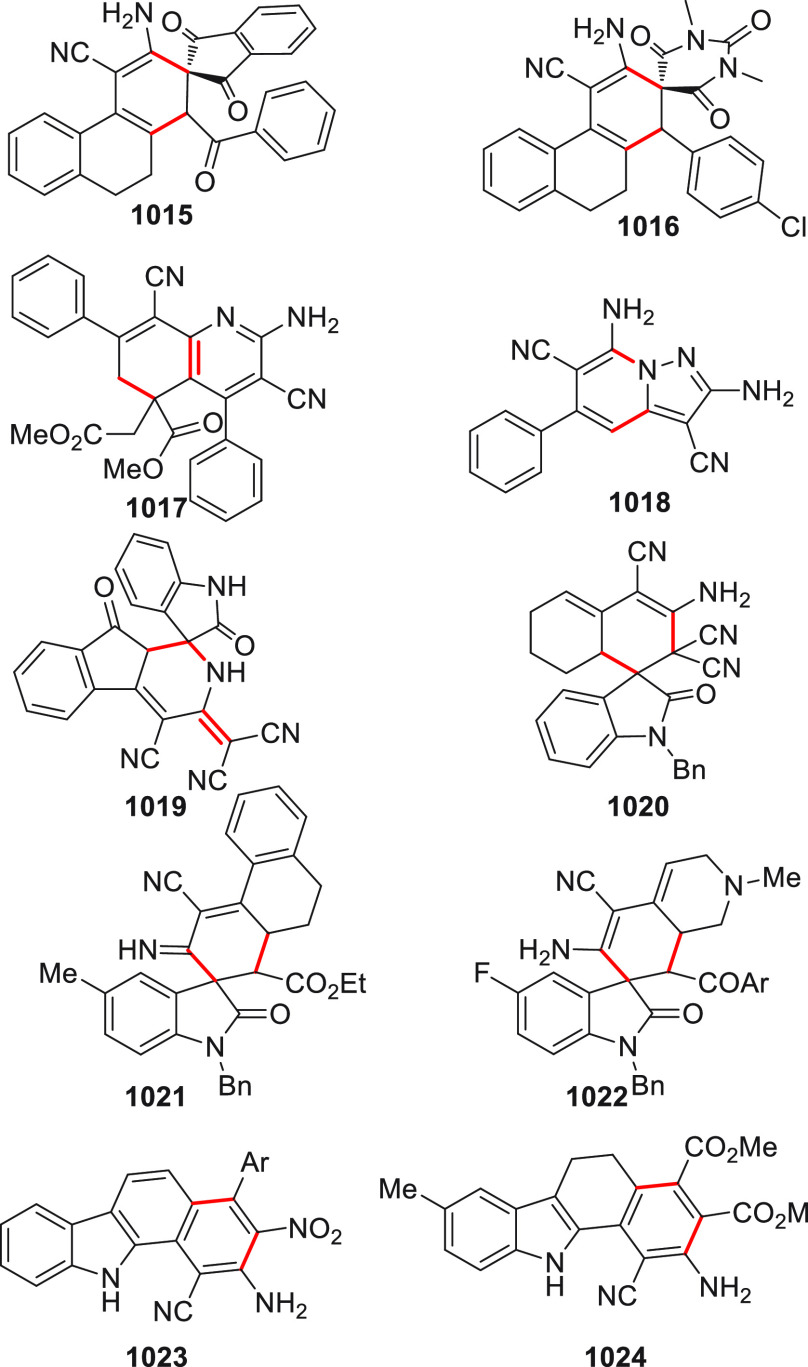
Polycyclic structures obtained through base-activated domino reactions between cyclic α,α-dicyanoalkene pronucleophiles and Michael acceptors.

As stated before, the vinylogous adducts deriving from addition reactions of α,α-dicyanoalkene pronucleophiles to electrophiles are prone to further manipulation including cyclization, tandem processes, or even elimination. Most of these elaborations carry to aromatic products with the loss of the newly formed stereocenters. These works are less interesting from a methodological point of view but have their strength in the straitghforward formation of uncommon aromatic structures or substitution patterns. Some examples are reported in Figure 10, dealing with diversely functionalized products formed by domino reactions of cyclic α,α-dicyanoalkene pronucleophiles with Michael acceptors, through base activation in an achiral environment. As can be observed, the variety of the targeted chemotypes is wide and includes phenanthrene derivatives (**1015** and **1016**),^664^ 5,6-dihydroquinoline motif (**1017**),^665^ pyrazolo[1,5-*a*]pyridines (**1018**),^666^ indenopyridine-fused spirocyclic systems (**1019**),^667^ spirocyclic oxindoles (**1020**, **1021**, and **1022**),^668−670^ and benzo[*a*]carbazole derivatives (**1023** and **1024**).^671,672^ Some of the compounds reported in Figure 10 still bear stereogenic centers, but they were isolated as racemic mixtures since the reactions were catalyzed by achiral bases (commonly Et_3_N, DIPEA, or DBU).

**Scheme 262:**
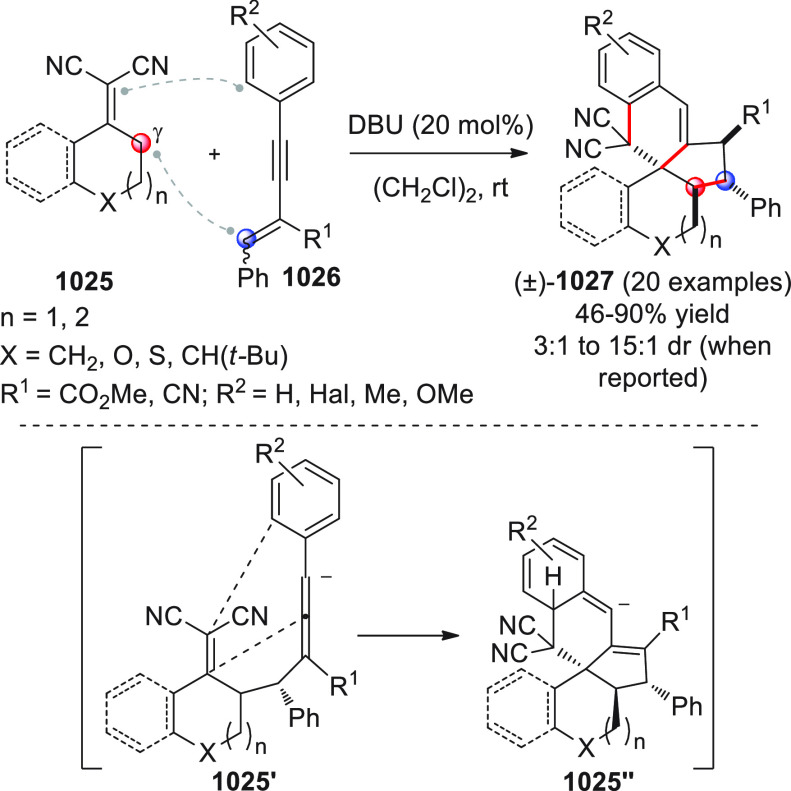


A similar base-promoted procedure was developed by Zhang and Zhang for the synthesis of angularly fused polycycles **1027** (Scheme 262).^673^ The reaction of cycloalkylidene malononitriles **1025** to enynes **1026** was catalyzed by DBU (20 mol %) in dichloroethane at room temperature and gave products **1027** in reasonable to very good yields and appreciable diastereocontrol. The first step of this one-pot tandem reaction is the vinylogous Michael addition of deprotonated **1025** to the electron-deficient enyne acceptors **1026**. The allene intermediate of type **1025′** undergoes formal dehydro-Diels–Alder reaction to the target **1027**, via stepwise anionic pathway (**1025′′**).

**Scheme 263:**
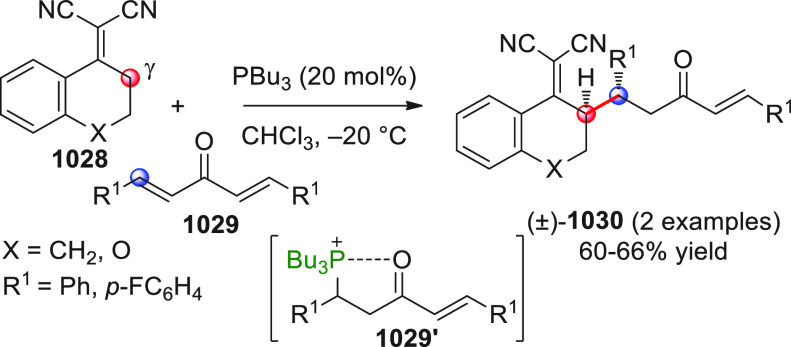


In a study of phosphine-catalyzed [4 + 2] annulation reactions between 1,4-dien-3-ones (**1029**) and α,α-dicyanoalkenes, He and co-workers reported also two examples of vinylogous addition reactions of cycloalkylidene malononitriles **1028** to dienones **1029**, which provided *anti*-configured, racemic products **1030** (Scheme 263).^674^ The work represents a rare example of phosphine catalyzed VMcA, in which the enolate from **1028** reacts with the phosphine activated intermediate **1029′**. The versatility of this procedure proved however limited since it could be applied just to dienones as acceptors, while a similar reaction between **1028** and chalcone (a monoenone) did not provide any addition product.

**Scheme 264:**
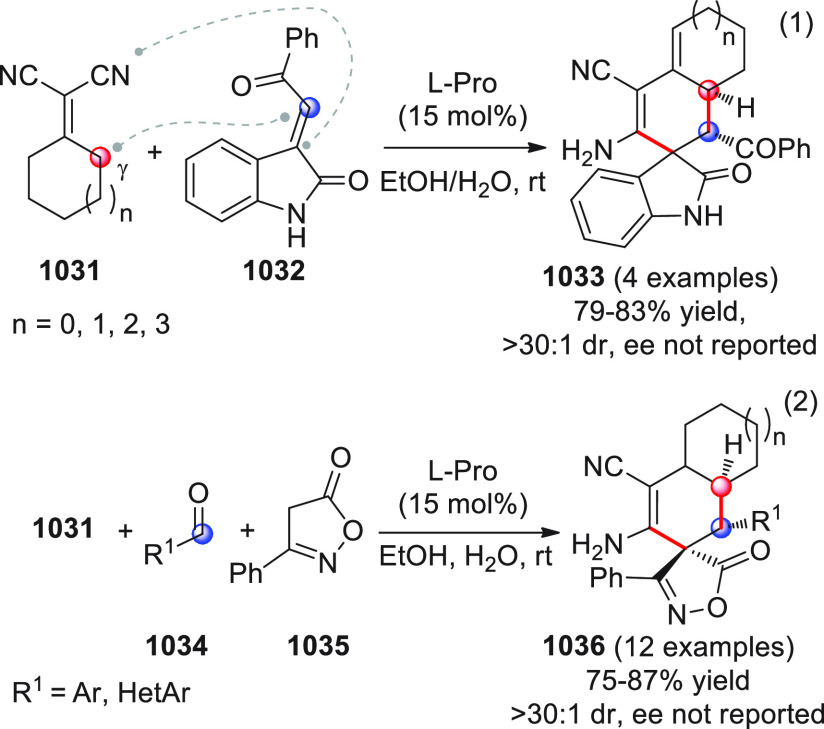


The organocatalyzed synthesis of polyfunctionalized spirooxindoles of type **1033** was reported by Perumal et al. featuring a domino reaction between vinylogous malononitriles **1031** and isatin-derived chalcone **1032** in the presence of l-proline (15 mol %) as the catalyst (Scheme 264, eq 1).^675^ The products were obtained in high yields as single *trans* diastereoisomers, but regrettably, no mention of their enantiomeric purity was made in the paper. In the same work, this procedure was also applied to the multicomponent reaction of **1031** with aromatic and heteroaromatic aldehydes **1034** and isoxazoles **1035** (Scheme 264, eq 2) to prepare spiroisoxazolones **1036**, but again no information about the enantiomeric excesses of the products was reported.

**Scheme 265:**
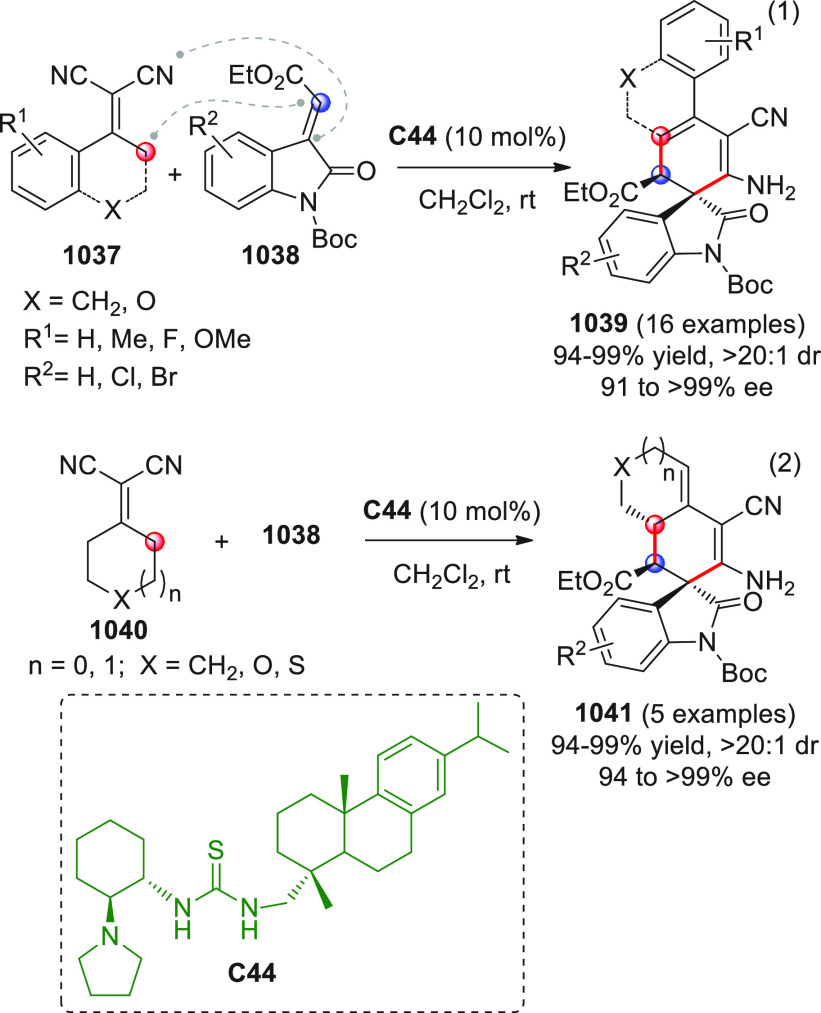


The first organocatalyzed, enantioselective vinylogous Michael/cyclization reaction cascade of α,α-dicyanoalkenes with 3-alkylidene oxindole acceptors for the synthesis of enantiopure spirooxindoles was reported by Wang et al. in 2013 (Scheme 265).^676^ The authors presented the reaction using both acyclic and cyclic dicyanoalkenes. The rosin-derived bifunctional thiourea catalyst **C44**, bearing a pyrrolidine group, triggered the reaction between dicyanoolefins **1037** and electron-poor alkylidene oxindoles **1038**, to provide spirooxindoles **1039** in high yields and excellent diastereo- and enantioselectivities (Scheme 265, eq 1). The same conditions applied to the more flexible monocyclic dicyanoolefins **1040** furnished spriooxindoles **1041** with equally optimal results (Scheme 265, eq 2), thanks to an unexpected final tautomerization process.

**Scheme 266:**
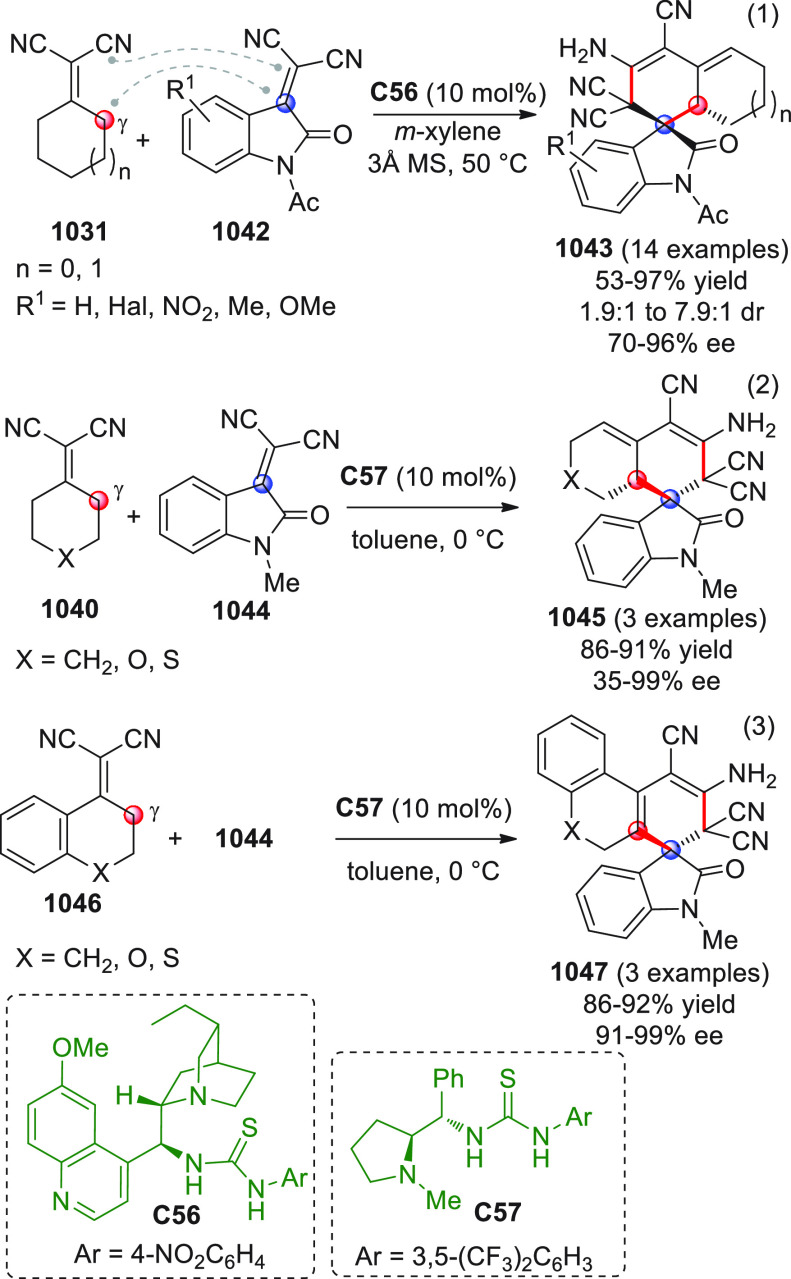


Structurally similar spirooxindoles of type **1043** were prepared in good yields, acceptable diastereoselectivities, and good to excellent enantioselectivities by Wang and co-workers, by exploiting the VMcR between dicyanoolefins **1031** and isatylidene malononitriles **1042** triggered by bifunctional thiourea catalyst **C56** (Scheme 266, eq 1).^677^ The organocatalyst worked as both a Brønsted base to generate the γ-carbanion from **1031** and an activating unit of **1042** by hydrogen bonding between the thiourea moiety and the C=O group of the oxindole core.

One year later, in 2015, a similar vinylogous Michael addition–cyclization reaction between dicyanolefins **1040** or **1046** and *N*-methyl-protected isatylidene malononitrile **1044** (Scheme 266, eqs 2 and 3) was reported by Kesavan and collaborators for the access to spirooxindoles of type **1045** or **1047**.^678^ The reaction was organocatalyzed by l-proline derived bifunctional thiourea **C57** under mild reaction conditions (0 °C in toluene). Interestingly, a one-pot three component procedure, using dicyanoolefins **1040** as nucleophiles with *N*-unprotected isatin and malononitrile as Michael acceptors, was carried out, obtaining very good results in terms of yields and enantioselectivities.

**Scheme 267:**
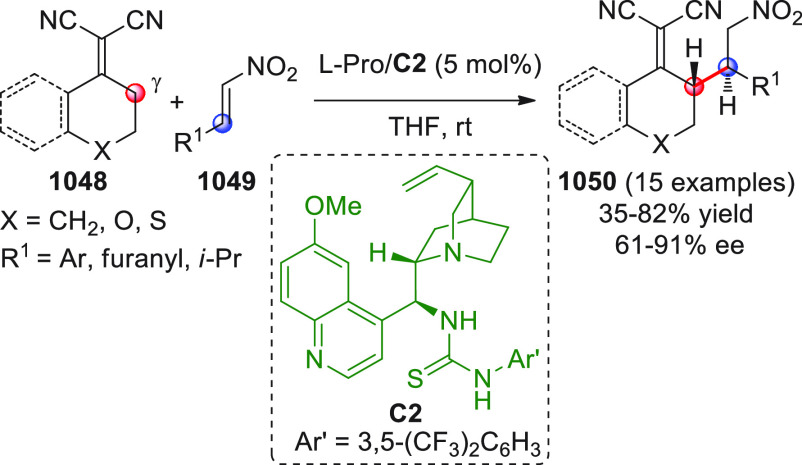


The first direct asymmetric procedures of vinylogous Michael additions of cycloalkylidene malononitriles of type **1048** to nitroolefins **1049** were independently published by Deng et al. in 2005,^679^ Jørgensen et al. in 2006,^680^ and Chen et al. in 2007,^681^ with good results in terms of yields and enantioselectivities; in 2012, Chen and collaborators reported a new study of this reaction catalyzed by self-assembled organocatalytic systems (Scheme 267).^682^ As outlined in Scheme 267, formation of products **1050** was ensured by the combination of l-proline and thiourea-tertiary amine **C2** (5 mol % each). However, this strategy did not give better results in terms of yields and enantioselectivities of the products with respect to previous publications.

**Scheme 268:**
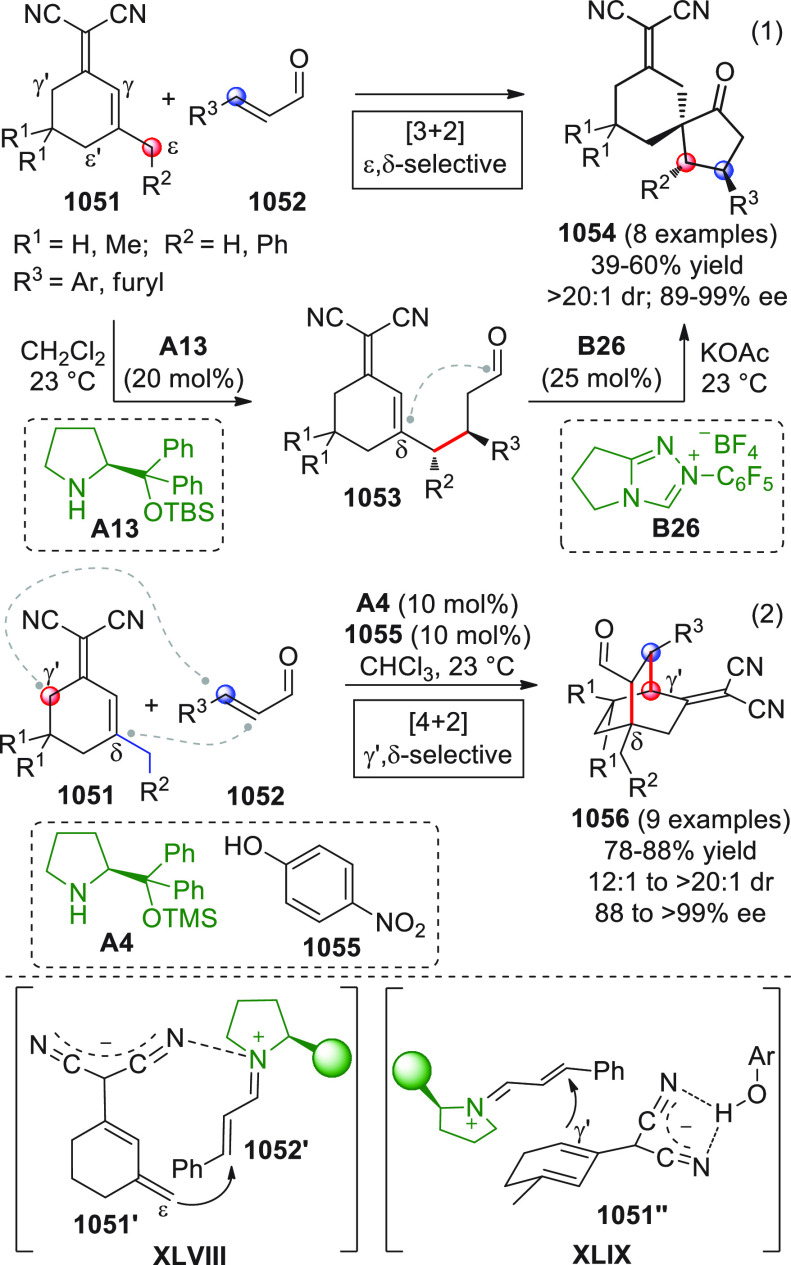


In 2014, Zanardi and co-workers reported the first use of cycloalkylidene malononitriles of type **1051** as vinylogous pronucleophiles.^683^ These compounds can be in principle deprotonated at different positions (e.g., ε, ε′, and γ′-positions), offering multiple pronucleophilic sites and making the regioselectivity control of the reaction a challenging issue. The reaction of **1051** with α,β-unsaturated aldehydes **1052**, triggered by prolinol-derived catalyst **A13**, led to the formation of isolable, ε-selective vinylogous Michael addition products **1053** (Scheme 268, eq 1). When the reaction solution was added with NHC-precatalyst **B26** and KOAc base, a 1,6-Stetter closure consecutive to the first hypervinylogous Michael addition provided [3 + 2]-spiroannulated products **1054** with moderate to good yields, excellent diastereoselectivity, and very high levels of enantioselectivity. Interestingly, under similar reaction conditions (catalyst **A4** instead of **A13**, CHCl_3_ instead of CH_2_Cl_2_) and using *p*-nitrophenol (**1055**) as cocatalyst, the reaction between **1051** and enals **1052** proceeded along a regiodivergent pathway, giving γ′,δ-selective [4 + 2] annulated products **1056** in high yields and excellent enantioselectivities (Scheme 268, eq 2). In this case, the vinylogous attack of the γ′ position of **1051** to iminium ion-activated **1052** was favored, followed by intramolecular attack of the enamine intermediate at the δ-site of **1051**. Several control experiments indicated that the regiodivergence toward either ε,δ-[3 + 2] or γ′,δ-[4 + 2] products (eq 1 vs eq 2) was dictated by the presence or not of *p*-nitrophenol cocatalyst. When it is absent, a transition state of type **XLVIII**, carrying to compound **1053**, is favored because of Coulombic interactions between the extended enolate **1051′** and the positively charged nitrogen atom of **1052′**; on the other hand, when the phenol cocatalyst is present, a transition state of type **XLIX**, where the cross-conjugated enolate **1051″** approaches the iminium ion **1052′** along an *endo* Diels–Alder-like trajectory, is favored, thanks to the stabilization of **1051″** by hydrogen bonding with donor **1055**.

**Scheme 269:**
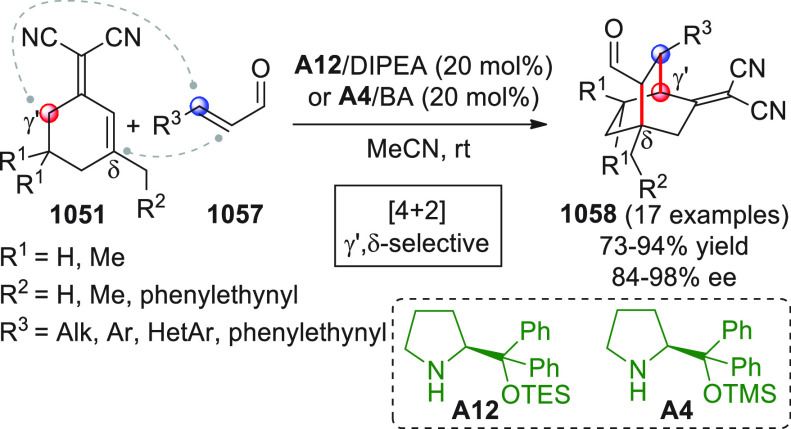


An analogous formal [4 + 2]-cycloaddition between cyclohexenylidene malononitrile **1051** and enals **1057** under secondary amine organocatalysis for the access to bicyclooctanes **1058** was reported almost concurrently and independently by Chen and co-workers (Scheme 269).^684^ In this case, the authors optimized the reaction conditions not only for aromatic-group substituted enals (R^3^= Ar, HetAr) but also for alkyl-group substituted acroleins (R^3^ = Alk). In the first case, the reaction was catalyzed by TES-protected prolinol **A12** and DIPEA, while in the second case the TMS-protected prolinol **A4** in combination with benzoic acid was the best catalytic system to provide the products in good yields and with high enantiomeric excesses.

**Scheme 270:**
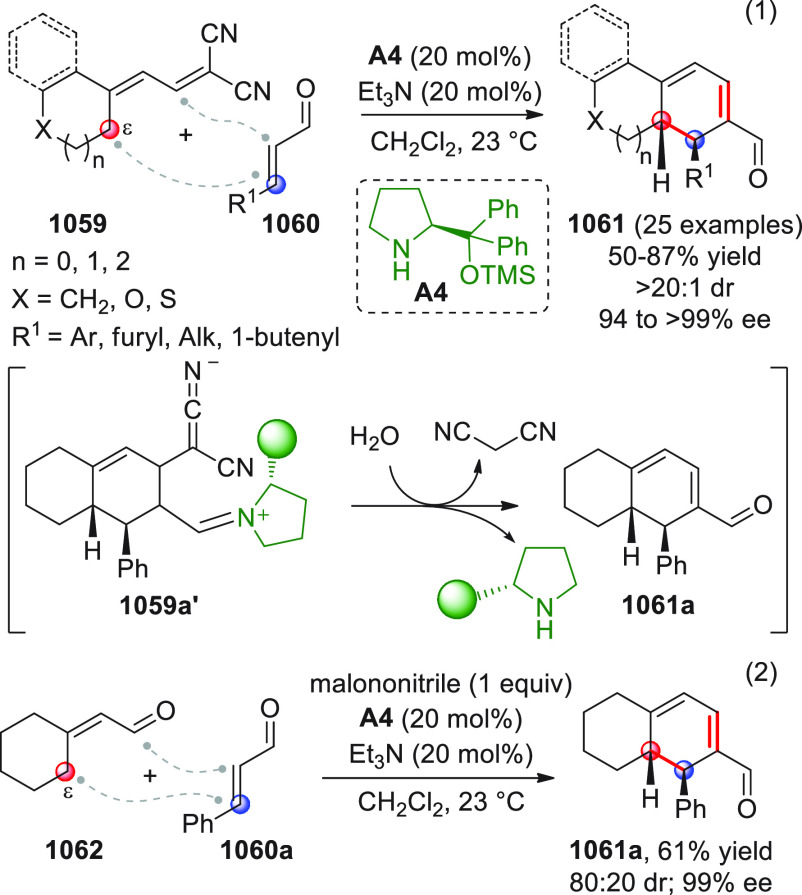


The following year, Zanardi and co-workers reported the use of allylidene malononitriles of type **1059** (Scheme 270, eq 1) as vinylogous donors, whose π-system conjugation propagates the nucleophilic character to the remote ε-position on the cycle.^685^ The reaction with both aliphatic and aromatic enals **1060**, catalyzed by prolinol-derived catalyst **A4**, provided a series of fused polycycles **1061** in good yields, complete *trans*-diastereoselectivity, and very high enantioselectivities, via a formal [4 + 2]-eliminative cycloaddition reaction. The authors proposed a plausible mechanism, describing the reaction as a stepwise domino process involving a bis-vinylogous Michael/Michael/retro-Michael organocascade. After the first bisvinylogous Michael addition of **1059** to the iminium ion-activated acceptor **1060**, an enamine intermediate is formed, which undergoes intramolecular Michael addition to the cycloadduct **1059a′**. Finally, catalyst hydrolysis and malononitrile elimination (retro-Michael) deliver product **1061a**. It is noteworthy that the authors performed also a one-pot procedure (Scheme 270, eq 2), using aldehyde **1060a** in the presence of a stoichiometric amount of malononitrile for the in situ formation of allylidene malononitrile **1059a** via Knoevenagel reaction, isolating the desired product **1061a** in good yield and excellent enantiomeric excess, even if with lower dr with respect to the two-step procedure.

**Scheme 271:**
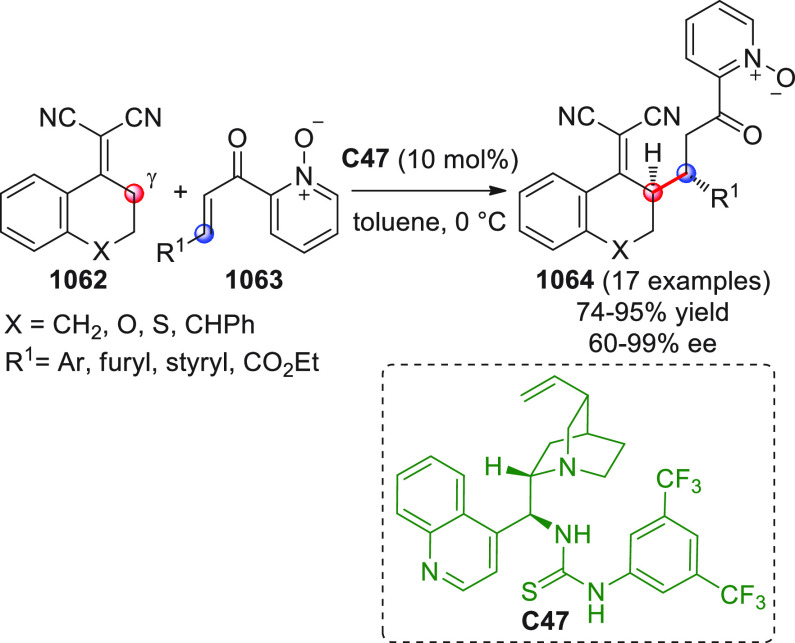


The first asymmetric and direct VMcR of α,α-dicyanoalkenes **1062** to 2-enoylpyridine *N*-oxides **1063** with a bifunctional thiourea/amine organocatalyst **C47** was disclosed by Singh et al. in 2014 (Scheme 271).^686^ The products **1064** were obtained in good yields and selectivity. Two acyclic α,α-dicyanoalkenes proved equally successful.

**Scheme 272:**
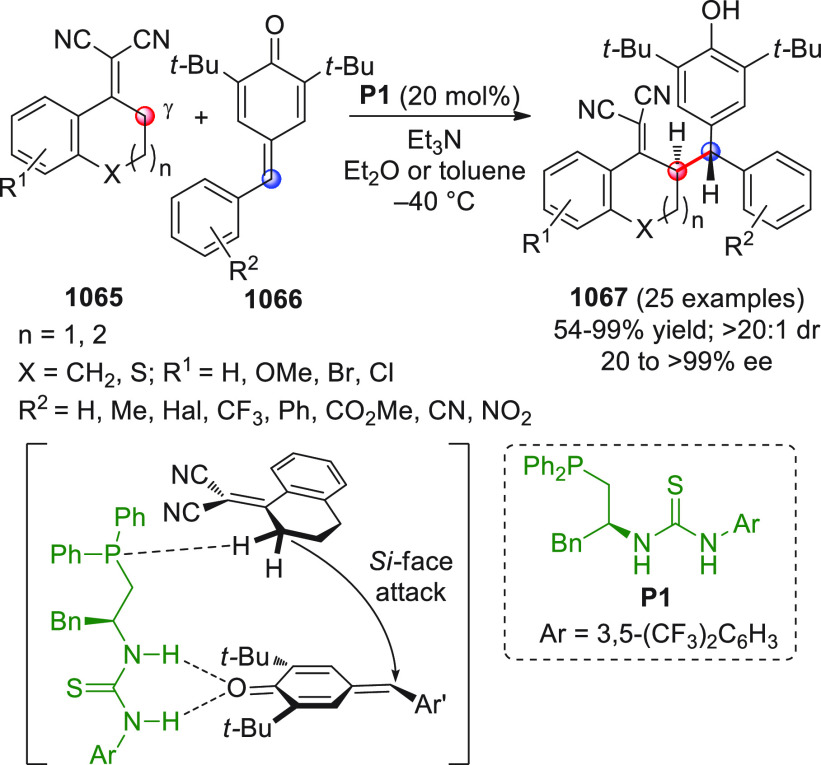


In 2016, Yao et al. reported the direct vinylogous 1,6-conjugate addition of alkylidene malononitriles **1065** to *p*-quinone methides of type **1066** (Scheme 272).^687^ The reaction was catalyzed by chiral phosphine-thiourea organocatalyst **P1** with triethylamine in diethyl ether or toluene and provided diarylmethine products **1067** in good yields, with high diastereocontrol and low to excellent enantioselectivities. Very low results in terms of enantioselectivity were obtained using cyclohexanone-derived dicyanoolefin (not shown). The role of the phosphorus atom in the catalytic cycle was unclear, but it was thought to play a role as a Lewis base on dicyanoolefin. The authors proposed the activation mode shown in Scheme 272, where the remote stereocontrol was achieved through intermolecular hydrogen-bond interaction between the thiourea catalyst and the *p*-quinone methides, while dicyanoolefin was more prone to attack the *p*-quinone methides from the *Si* face.

## 8 Other Vinylogous Pronucleophiles

### 8.1 Direct Procedures

#### 8.1.1 Acyclic and Cyclic Pronucleophiles

This section groups in tabular format a selection of illustrative pieces of research in which quite unconventional pronucleophiles including α,β-unsaturated acyl chlorides,^688,689^ carboxylic acids,^690−692^ alkylnitroisoxazoles,^693−698^ vinylphenols,^699−705^ nitrotoluenes,^706−708^ and allylsulfones^709^ act as leading players in asymmetric vinylogous transformations. All the reported examples deal with the in situ generation of the reactive polyenolate-type donor species (direct activation modalities) which couple to suitable C=O, C=N, and activated C=C bonds at their vinylogous sites.

The first reported example deals with the use of γ-enolizable α,β-unsaturated acyl chlorides as vinylogous donors in tertiary amine-catalyzed [4 + 2] cycloaddition reactions with activated aldehydes as chloral (Table 12, eq 1).^688^ The authors demonstrated that the transformations proceeded through the formation of zwitterionic ammonium enolates of type **L** (eq 1), generated in situ from the starting acyl chlorides by use of the nucleophilic amine catalyst **C59** and Sn(OTf)_2_ as Lewis acid cocatalyst. Enantioentriched δ-lactones were forged in variable yields, depending upon the nature of the R^1^ substituents within the donors. While this procedure proved quite limited as for the electrophilic scope, the authors identified in the same work an alternative catalyst combination (e.g., Er(OTf)_3_ in complex with chiral aliphatic β- or γ-amino alcohols), which was able to trigger the [4 + 2] cycloaddition of unsaturated acyl chlorides with unactivated aldehydes as benzaldehyde (not shown).

**Table 12:**
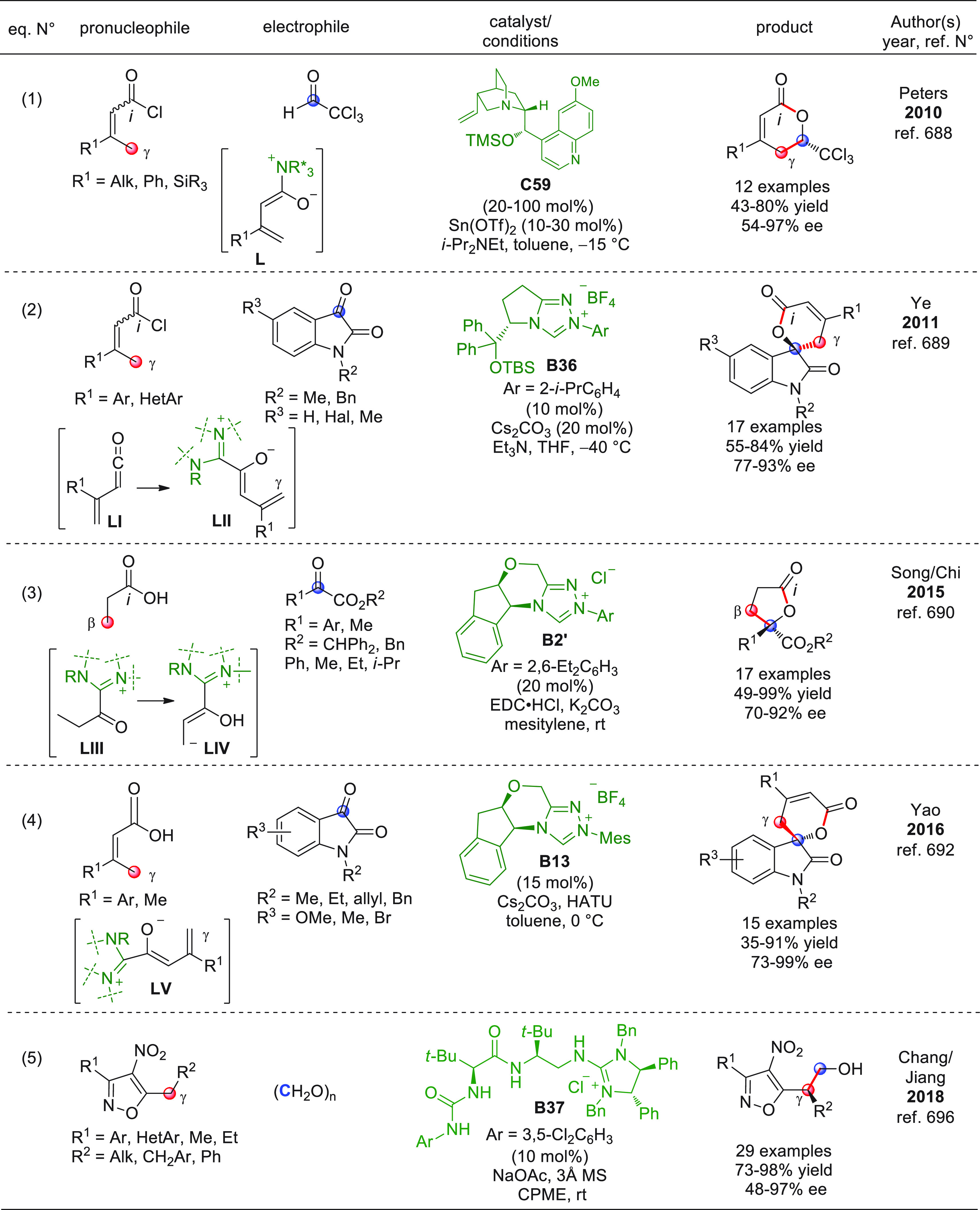
Asymmetric Vinylogous Additions of Unconventional Pronucleophiles to C=O Bonds

α,β-Unsaturated β-methylacyl chlorides were also employed in NHC-catalyzed [4 + 2] cyclization reactions with activated ketones such as isatins, which gave the corresponding spirocyclic unsaturated δ-lactones in good yields and enantiomeric excesses (Table 12, eq 2).^689^ It was proposed that NHC-bound vinyl enolates **LII** were operative, which were likely obtained in situ from vinyl ketenes **LI**, in turn generated by dehydrohalogenation of the starting acyl chloride with Et_3_N base. As in the previous case, the authors could not precisely define whether the δ-lactone products were the result of a concerted HDA cyclization or stepwise VAR followed by intramolecular closure.

Direct β-activation of propionic acid by NHC catalysis was developed by Song, Chi, et al. to be exploited in formal [3 + 2] cycloaddition with keto esters (Table 12, eq 3).^690^ Mechanistically, condensation of the three carbon-long acid with a coupling reagent (EDC) forms a carboxylic anhydride that reacts with the NHC catalyst **B2′**, to furnish the activated acyl intermediate **LIII** and hence the β-donor homoenolate **LIV**, ready for the coupling reaction with the carbonyl acceptor.

γ-Enolizable carboxylic acids (either α,β-unsaturated or saturated) were convenient γ-donor substrates to be exploited in NHC-catalyzed [4 + 2] cyclizations with isatins (Table 12, eq 4)^692^ or acylhydrazones (Table 13, eq 1).^691^ Treatment of the starting carboxylic acids with a condensing agent (HATU), NHC catalyst **B13** or **B38**, and a base could release the corresponding activated intermediates, namely, dienolate **LV** (Table 12, eq 4) or dienolate **LVII** after oxidation of enolate **LVI** (Table 13, eq 1), which were ready for the asymmetric assemblage of the respective δ-lactone or δ-lactam products.

**Table 13:**
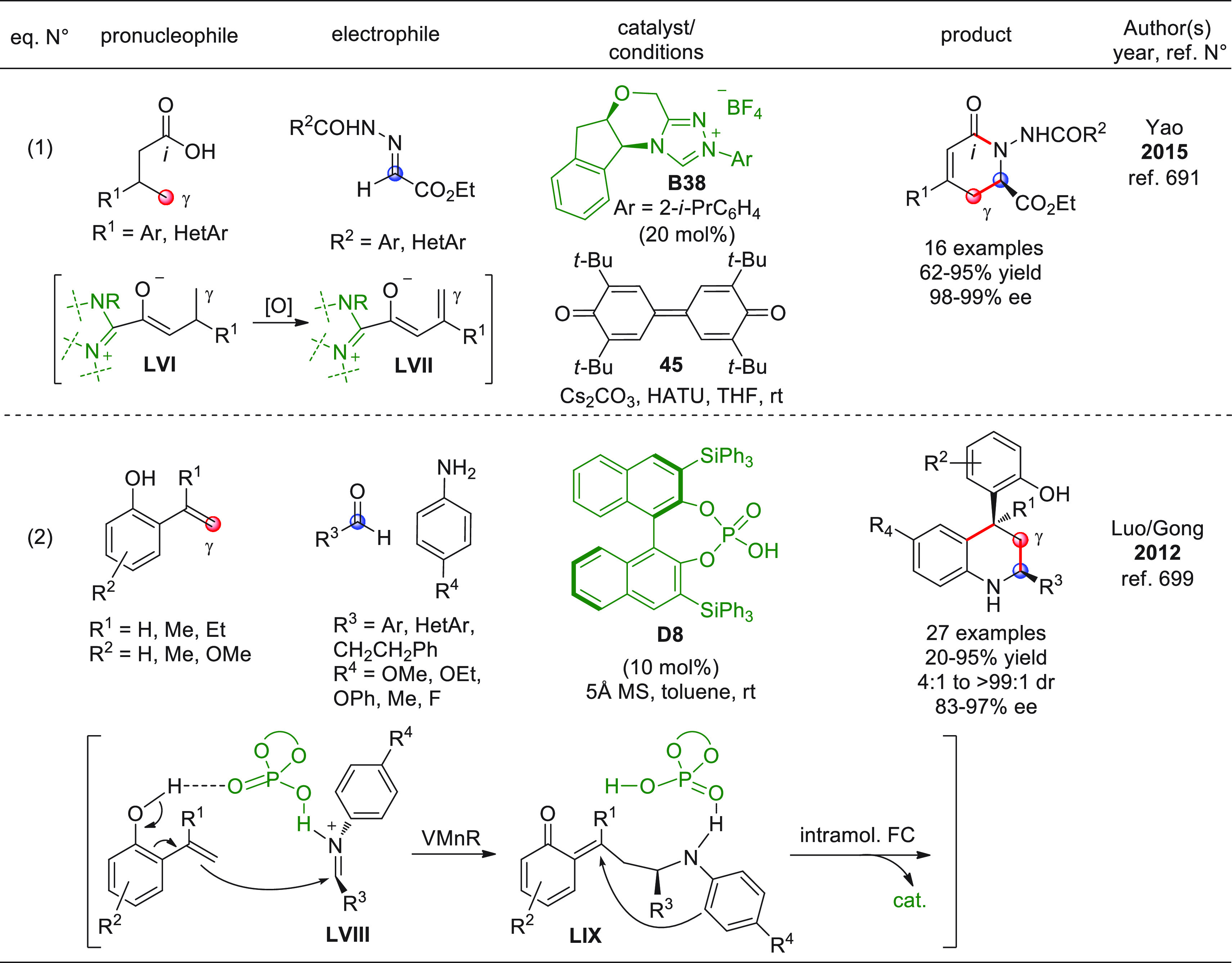
Asymmetric Vinylogous Additions of Unconventional Pronucleophiles to Multiple C–N Bonds

Direct asymmetric addition reactions of alkyl-substituted benzenes or heterocycles such as 5-alkyl-4-nitroisoxazoles could be effectively carried out by introducing nitro groups at *ortho*- and/or *para*-positions of the aromatic ring. In fact, the enhanced acidity of the C(sp^3^)-H benzylic protons induced by the presence of the nitro functionality could be exploited in either addition reactions to carbonyl compounds (Table 12, eq 5) or conjugate additions to electron-deficient alkenes (Table 14, eqs 1, 2, 3, and 6) under organocatalytic conditions. In other words, the leading functional group responsible for the pronucleophilic reactivity at the remote conjugated benzylic position is not a carbonyl function (as, for example, in Scheme 26, or Table 4, eqs 1 and 2) but rather the nitro function, according to vinylogous Henry-type nitroaldol or conjugate addition reactions involving the π-system of the (hetero)aromatic ring. Thus, for example, nitrotoluenes were employed in conjugate additions to iminium ion activated enals (Table 14, eqs 1 and 2)^707,708^ to afford the corresponding alkylated toluenes in high enantiomeric excesses. Using “elongated” nitrovinyl-nitrotoluenes such as those reported in Table 14, eq 3, efficient preparation of highly enantioenriched hexahydrophenanthrenes was at hand.^706^ Using, instead, 5-alkyl-4-nitroisoxazoles as heterocyclic vinylogous pronucleophiles, the VAR to paraformaldehyde (Table 12, eq 5) and VMcA to enals (Table 14, eq 6) were successfully realized.^696,698^

**Table 14:**
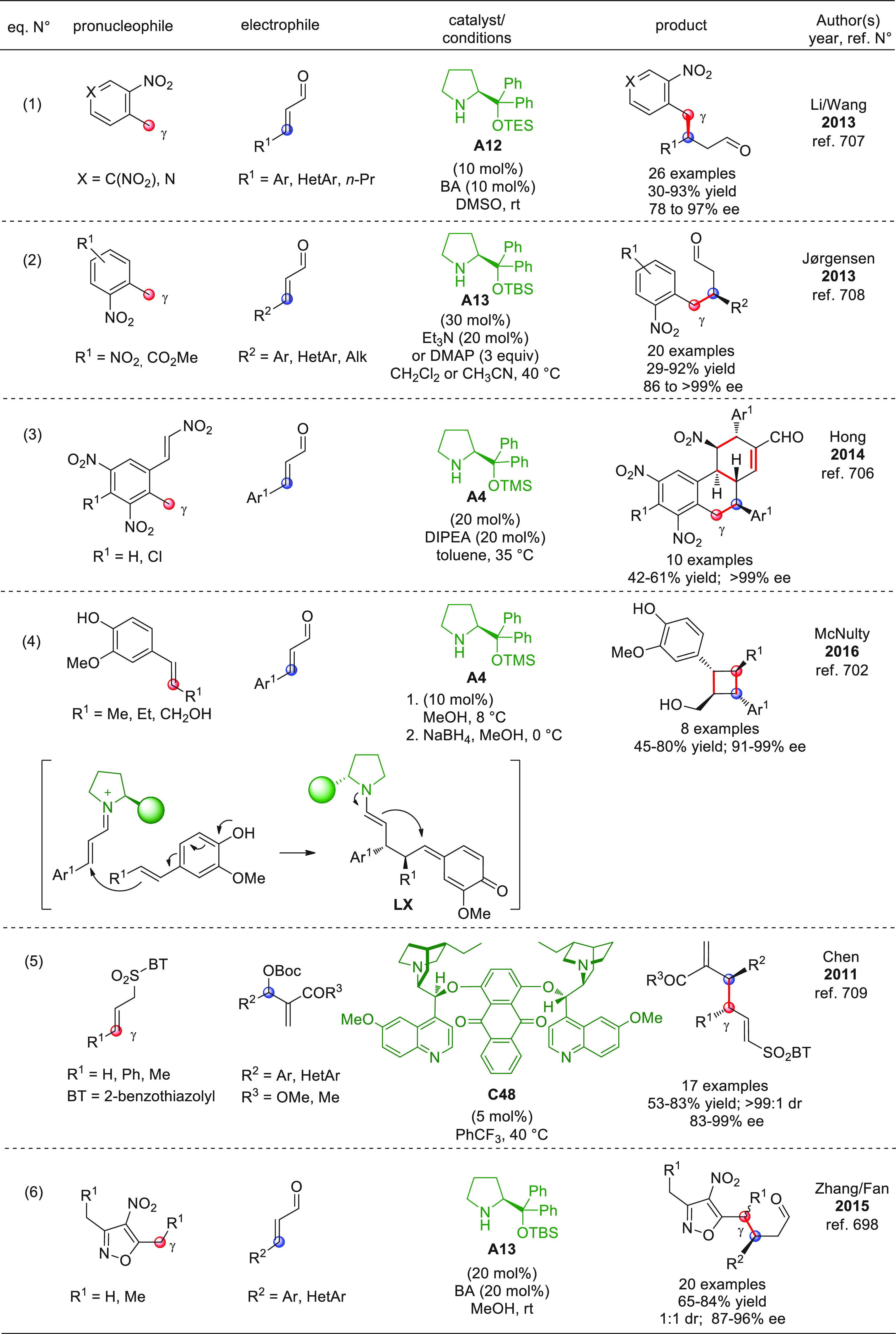
Asymmetric Conjugate Additions of Unconventional Pronucleophiles to Activated C=C Bonds

Another unconventional vinylogous pronucleophilic system is given by *ortho*- or *para*-substituted vinylphenol. Vinylphenols, together with vinylindoles,^14^ vinylpyrroles, and other π-extended electron-rich (hetero)aromatic substrates^710,711^ have been widely exploited in recent asymmetric synthesis programs as useful pronucleophilic synthons in remote Friedel–Crafts-type reactions. However, to stay focused on the subject of this review, we here restrict the field to *o*-vinyl- or *p*-vinylphenols, since their topology is strictly connected to dienol or polyenol species. Among these, we document just a few examples, where the presence of the OH group within the vinylphenol moiety proved to be essentially important to guide reactivity, exocyclic remote regioselectivity, and enantioselectivity.

In a first example, a Brønsted acid-catalyzed three-component Povarov reaction involving 2-hydroxystyrenes was developed, which provided an efficient method to access highly enantioenriched tetrahydroquinolines (Table 13, eq 2).^699^ The authors demonstrated that the phosphoric acid catalyst **D8** acted as a bifunctional species activating both vinylphenol and the in situ-generated imine by hydrogen bonding interactions (species **LVIII**, eq 2). A two-step reaction pathway was operative, according to which a vinylogous Mannich reaction between vinylphenol and the imine acceptor occurred (**LVIII** to **LIX**), followed by intramolecular Friedel–Crafts reaction to the target compounds.

Secondary amine-catalyzed [2 + 2] cycloaddition between *p*-vinylphenols such as isoeugenol and cinnamaldehydes was developed by McNulty et al. in 2016, which afforded the corresponding tetrasubstituted cyclobutanes in good yields and enantiomeric excesses (Table 14, entry 4).^702^ Even in this case, it was found that the phenol group was crucially required to transmit the donor properties to the remote position with a postulated stepwise mechanism encountering a first attack of the vinylphenol to the aldehyde acceptor (activated as iminium ion by the amine catalyst **A4**), followed by intramolecular closure (via **LX**).

## 9 Concluding Remarks

In nearing to an end, this journey through the scientific publications dealing with the generation and use of π-extended enolate-type nucleophiles over the period of 2010–2018 calls for several final considerations.

The way in which vinylogous reactions were traditionally exploited has definitely been increased multifold or even surpassed by new concepts and new methods. Hence, the conventional generation of dienolates, via γ-deprotonation of unsaturated carbonyl compounds (with possible silyl enolate trapping), has been progressively supplemented with several ingenuous modalities where the starting pronucleophilic substrates are more complex in structure topology, chain length, substitution, and reactivity. A rapid glance to the general formulas of the (pro)nucleophilic species, reported at the beginning of each main chapter, confirms this. HOMO-raising activation modalities with chiral catalysts especially applied to π-extended carbonyl compounds (dienamine-, trienamine-, tetraenamine-activation, NHC-activation, noncovalent ion paired polyenolate activation, etc.) have resulted in hundreds of methodology-oriented papers revealing previously unexplored, remote C–C and C–X connections in an asymmetric context. *Nonetheless, it is still hard for such innovative opportunities, involving aldehyde and ketone substrates, to find applications in target-oriented asymmetric syntheses, and we expect this issue to be addressed in the near future*. On the other hand, the activation and use of π-extended ester/amide substrates are somehow more consolidated, and ample choice in indirect silyl ketene acetal-based procedures allowed many research groups to venture into successful target-oriented synthesis programs. A restricted area of vinylogy is occupied by the “other pronucleophiles”, including unconventional unsaturated acyl chlorides, carboxylic acids, alkylnitroisoxazoles, vinylphenols, nitrotoluenes, and allylsulfones, *whose exploitation as vinylogous substrates in asymmetric synthesis is quite a novelty and is expected to fill the agenda of future research*. *As a further promising future direction, we expect that the photocatalytic generation of polyenolates will offer an interesting yet challenging opportunity to expand the arsenal of the ways multidentate carbonyl-related donor species are produced and exploited*. Compared to our previous vinylogy-related review articles, we definitely noticed that researchers have been paying an increasing amount of attention, especially over the past 4–5 years, to the formulation of mechanistic hypotheses backed-up by solid calculations and experimental evidence; *we expect this trend to become more established in the near future*.

Finally, the extraordinarily wide spectrum of all the vinylogous products synthesized (which can be increased by postsynthesis transformations), vividly portrayed in the multitude of schemes and tables in this review article, bears witness to the fact that, in the context of contemporary organic synthesis, the application of vinylogy represents an excellent opportunity of selectively addressing molecular diversity beyond the boundaries of Nature.

## Abbreviations Used

Ac: acetyl
ACDC: asymmetric counterion-directed catalysis
Alk: alkyl
Ar: aryl
ATNP: aluminum tris(2,6-di-2-naphthylphenoxide)
ATPH: aluminum-tris(2,6-diphenylphenoxide)
B: base
BA: benzoic acid
BB: Brønsted base
BINOL: 1,1′-bi-2-naphthol
Bn: benzyl
Boc: *tert*-butoxycarbonyl
BSA: *N,O*-bis(trimethylsilyl)acetamide
Bt: *N*-hydroxybenzotriazole
BT: 2-benzothiazolyl
BTHL: BINOL-titanium-LiCl heterobimetallic complex
*t*-Bu: *tert*-butyl
BVAR: bisvinylogous aldol reaction
Bz: benzoyl
cat.: catalyst
Cbz: benzyloxycarbonyl
COD: 1,5-cyclooctadiene
CPME: cyclopentylmethyl ether
CSA: camphorsulfonic acid
Cy: cyclohexyl
CyH: cyclohexane
DA: Diels–Alder
DABCO: 1,4-diazabicyclo[2.2.2]octane
DBU: 1,8-diazabicyclo[5.4.0]undec-7-ene
DCE: 1,2-dichloroethane
DEA: *N,N*-diethylacetamide
DEP: *N,N*-diethyl-3-pyridinecarboxamide
DFT: density functional theory
[DHQ]_2_PHAL: hydroquinine 1,4-phthalazinediyl diether
[DHQD]_2_PYR: hydroquinidine-2,5-diphenyl-4,6-pyrimidinediyl diether
DIPEA: diisopropylethylamine
DMF: dimethylformamide
DMP: Dess−Μartin periodinane
DMAP: 4-dimethylaminopyridine
DMSO: dimethyl sulfoxide
DNBA: 2,4-dinitrobenzoic acid
DNBSA: 2,4-dinitrobenzenesulfonic acid
DPMS: diphenylmethyl silyl
DPTU: diphenyl thiourea
DPP: diphenylphosphoric acid
dr: diastereomeric ratio
DSI: chiral disulfonimide
DTBM-SEGPHOS: 5,5′-bis[di(3,5-di-*tert*-butyl-4-methoxyphenyl)phosphino]-4,4′-bi-1,3-benzodioxole
DVMcA: doubly vinylogous Michael addition
ee: enantiomeric excess
E1cB: elimination unimolecular conjugate base
EDC: 1-ethyl-3-[3-(dimethylamino)propyl]carbodiimide
Eoc: ethoxycarbonyl
equiv: equivalent(s)
EWG: electron-withdrawing group
*o*-FBA: 2-fluorobenzoic acid
*o*-FBZA: 2-fluorobenzaldehyde
FC: Friedel–Crafts
Hal: halogen
HATU: 1-[bis(dimethylamino)methylene]-1*H*-1,2,3-triazolo[4,5-*b*]pyridinium 3-oxide hexafluorophosphate,
HDA: hetero-Diels–Alder
Hex: hexyl
HetAr: heteroaryl
HFIP: hexafluoroisopropanol
HMDS: hexamethyldisilazide
HOMO: highest occupied molecular orbital
HVMAR: hypervinylogous Mukaiyama aldol reaction
HWE: Horner–Wadsworth–Emmons
ICD: isocupreidine
IEDDA: inverse electron demand Diels–Alder
IED-HDA: inverse electron demand hetero-Diels–Alder
LA: Lewis acid
LDA: lithium diisopropylamide
LTMP: lithium tetramethylpiperidine
LUMO: lowest unoccupied molecular orbital
MBH: Morita–Baylis–Hillman
Mes: 2,4,6-trimethylphenyl (mesityl)
MS: molecular sieves
MTBD: 7-methyl-1,5,7-triazabicyclo[4.4.0]dec-5-ene
MTBE: methyl *tert*-butyl ether
nd: not determined
NHC: N-heterocyclic carbene
NLE: nonlinear effect
Ns: 4-nitrobenzenesulfonyl
Nu: nucleophile
OXB: oxazaborolidinone
PCC: pyridinum chlorocromate
PG: protecting group
Ph: phenyl
Phe: phenylalanine
PMB: 4-methoxybenzyl
PMP: 4-methoxyphenyl
*i*-Pr: isopropyl
Pro: proline
ProPhenol: 2,6-bis[2-(hydroxydiphenyl)-1-pyrrolidinylmethyl]-4-methylphenol
PTSA: *p*-toluenesulfonic acid
*o*QDM: *ortho*-quinodimethane
[QD]_2_PHAL: quinidine 1,4-phthalazinediyl diether
rt: room temperature
TANIAPHOS: (1-[(*R*)-(dimethylamino)[2-(diphenylphosphino)phenyl]methyl]-2-(diphenylphosphino)-(2*R*)-ferrocene)
TBAF: tetra-*n*-butylammonium fluoride
TBAT: tetrabutylammonium difluorotriphenylsilicate
TBD: 1,5,7-triazabicyclo[4.4.0]dec-5-ene
TBDPS: *tert*-butyldiphenylsilyl
TBS: *tert*-butyldimethylsilyl
TBSOF: *tert*-butyldimethylsilyloxyfuran
TCE: trichloroethanol
TES: triethylsilyl
TFA: trifluoroacetate, trifluoroacetic acid
TFE: trifluoroethanol
THF: tetrahydrofuran
TIPS: triisopropylsilyl
TIPSOF: triisopropylsilyloxyfuran
TIPSOTf: triisopropylsilyl trifluoromethanesulfonate
TMAA: tetrabutylammonium acetate
TMAH: tetrabutylammonium hydroxide
TMG: 1,1,3,3-tetramethylguanidine
TMS: trimethylsilyl
TMSOF: trimethylsilyloxyfuran
TMSOTf: trimethylsilyl trifluoromethanesulfonate
TPS: triphenylsilyl
Tr: triphenylmethyl
Trp: tryptophan
Ts: 4-methylphenylsulfonyl
TTMSS: tris(trimethylsilyl)silyl
VA: vinylogous aldol
VAR: vinylogous aldol reaction
VMAR: vinylogous Mukaiyama aldol reaction
VMcR: vinylogous Michael reaction
VMMcR: vinylogous Mukaiyama Michael reaction
VMMnR: vinylogous Mukaiyama Mannich reaction
VMnR: vinylogous Mannich reaction
