# Supplementary figures and images for: New Developments of the Principle of Vinylogy as Applied to π-Extended Enolate-Type Donor Systems
Source: Chem Rev. 2020 Feb 10;120(5):2448–612. doi: 10.1021/acs.chemrev.9b00481 (PMC7993750; doi:10.1021/acs.chemrev.9b00481)

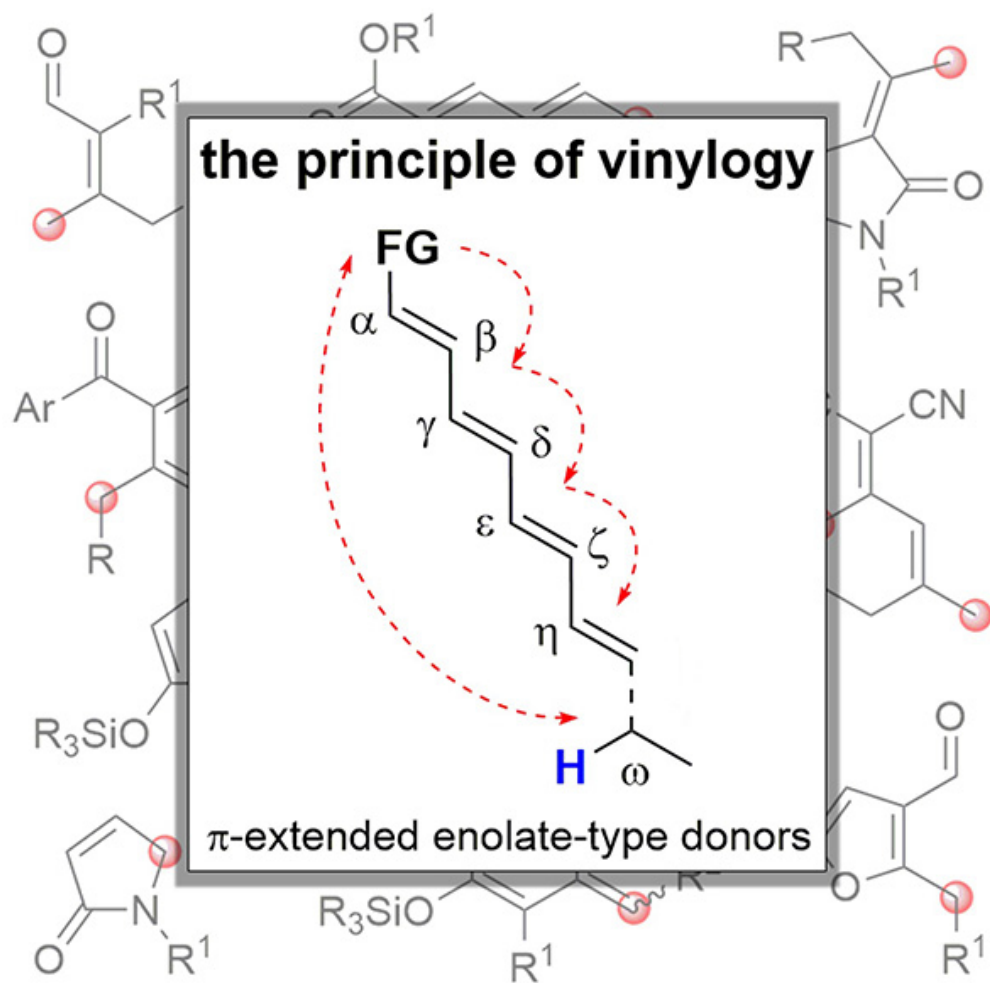

Supplement: Supplementary file 1 — cr9b00481_si_001.pdf [file cr9b00481_si_001.pdf]
